# Supplementary material for: E3 ligase AREL1 controls perinuclear localization of lysosomes and supports Purkinje cell survival
Source: EMBO J. 2025 Dec 2;45(3):655–91. doi: 10.1038/s44318-025-00654-3 (PMC12864862; doi:10.1038/s44318-025-00654-3)
Supplement: Supplementary file 7 — Movie EV4 [file 44318_2025_654_MOESM7_ESM.zip › Movie_EV4.docx]

**Movie EV4**

Representative video showing hindlimb clasping of a 12-month-old *Arel1*^-/-^ male mouse in the tail suspension test.
