# Supplementary figures and images for: E3 ligase AREL1 controls perinuclear localization of lysosomes and supports Purkinje cell survival (part 1 of 4)
Source: EMBO J. 2025 Dec 2;45(3):655–91. doi: 10.1038/s44318-025-00654-3 (PMC12864862; doi:10.1038/s44318-025-00654-3)

|               |  |    |    |       |       |       |       |       |       |             |     |
|---------------|--|----|----|-------|-------|-------|-------|-------|-------|-------------|-----|
| AREL1-myc     |  | WT | WT | G706A | T707A | G708A | D709A | I710A | S711A | D709A/I710A |     |
| ATP6V0A3-FLAG |  | -  | +  | +     | +     | +     | +     | +     | +     | +           | kDa |

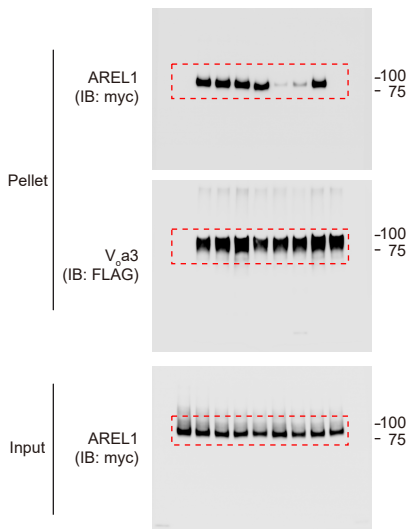

IP: FLAG

Supplement: Supplementary file 8 — Source data Fig. 1 [file 44318_2025_654_MOESM8_ESM.zip › Figure 1/1O/1O.pdf]

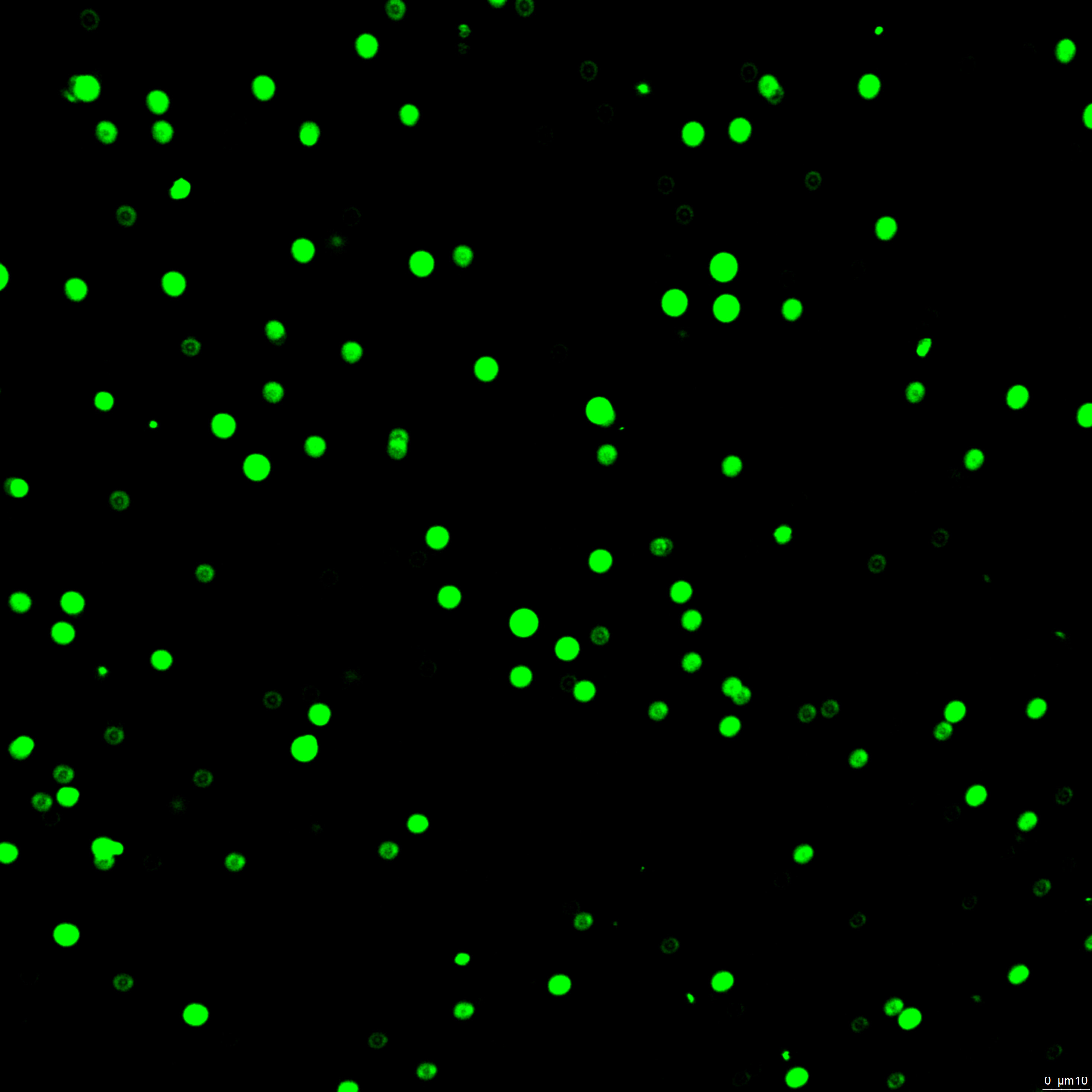

Supplement: Supplementary file 8 — Source data Fig. 1 [file 44318_2025_654_MOESM8_ESM.zip › Figure 1/1G/1G-3-mEGFP-LCR 50mM NaCl.tif]

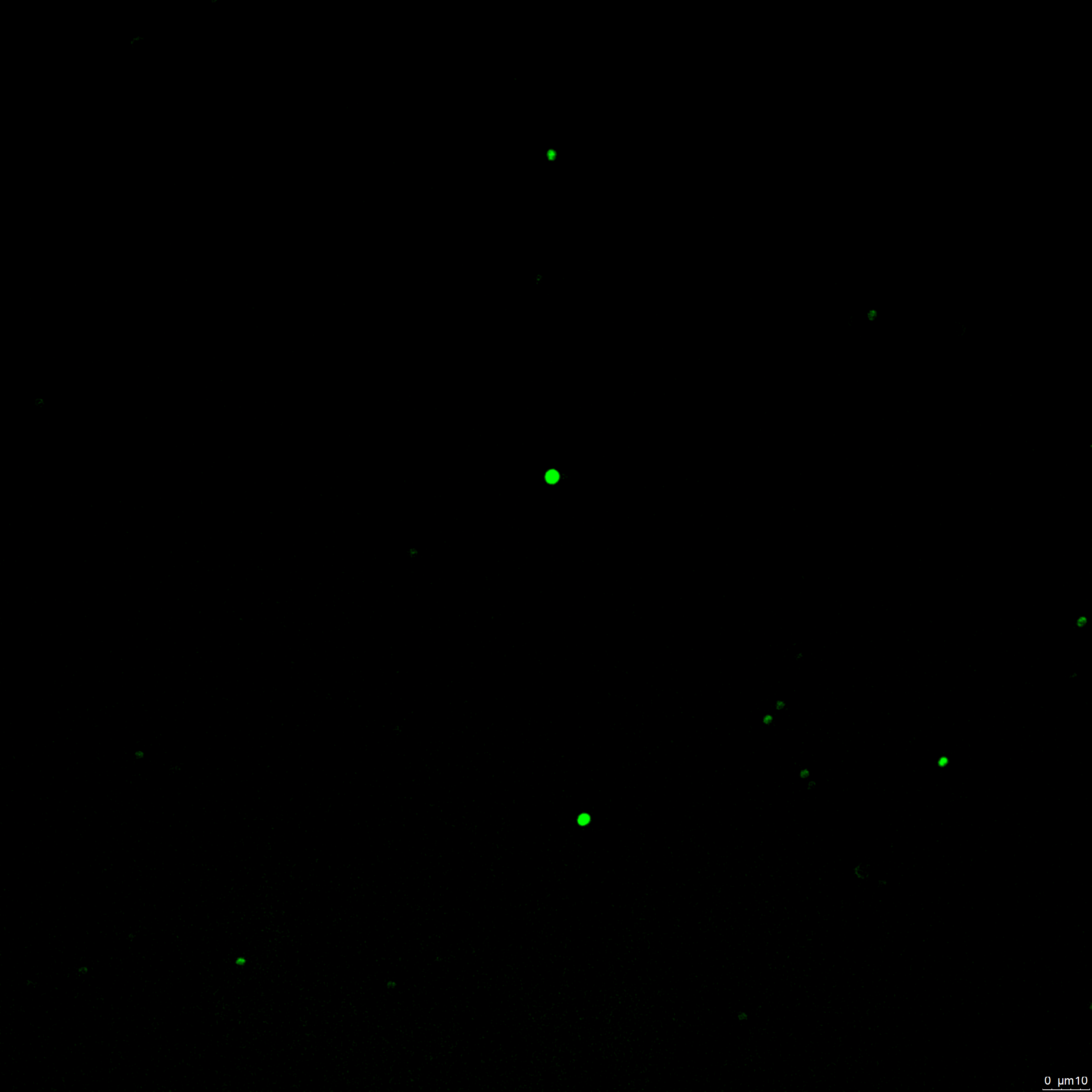

Supplement: Supplementary file 8 — Source data Fig. 1 [file 44318_2025_654_MOESM8_ESM.zip › Figure 1/1G/1G-5-mEGFP-LCR 200mM NaCl.tif]

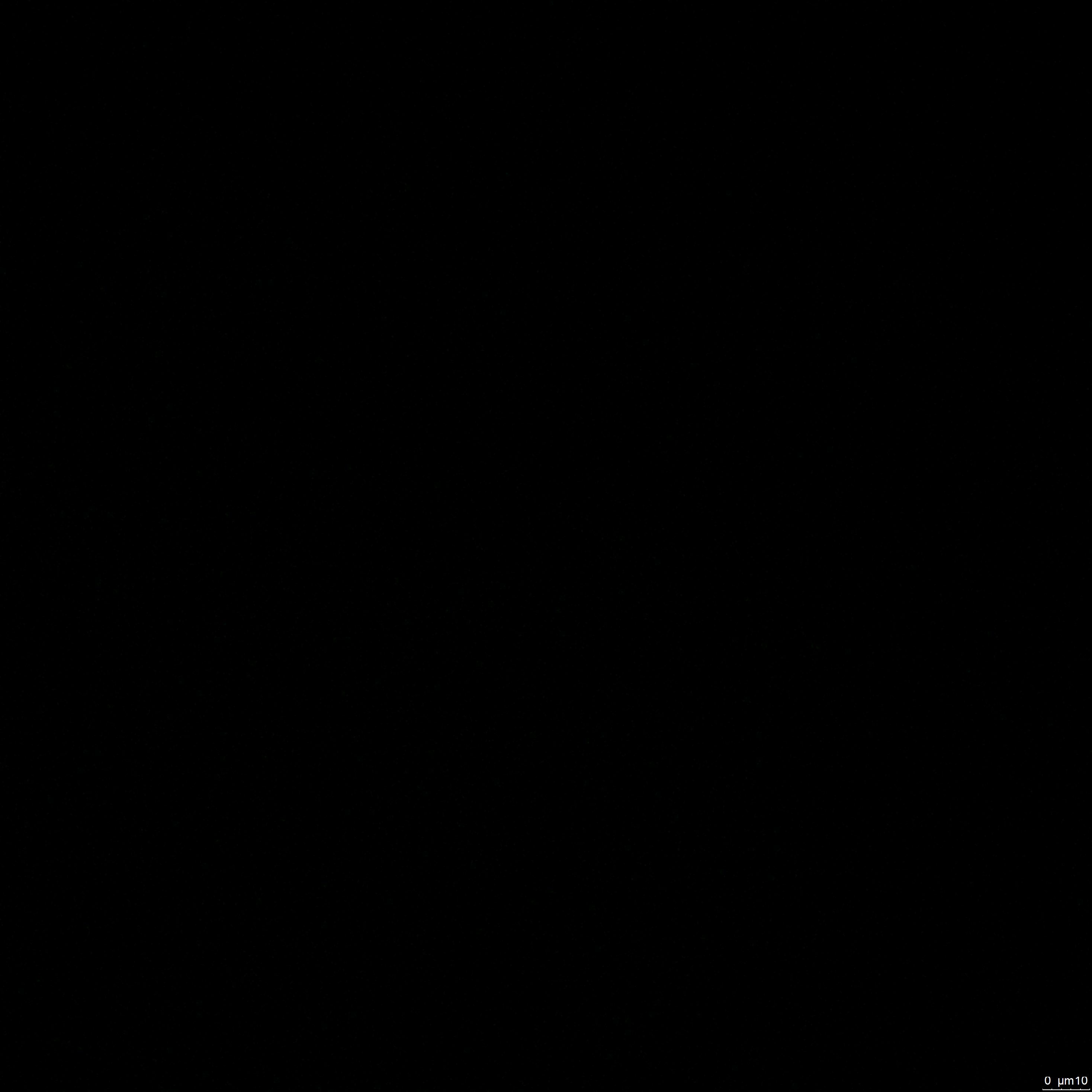

Supplement: Supplementary file 8 — Source data Fig. 1 [file 44318_2025_654_MOESM8_ESM.zip › Figure 1/1G/1G-1-mEGFP 50mM NaCl.tif]

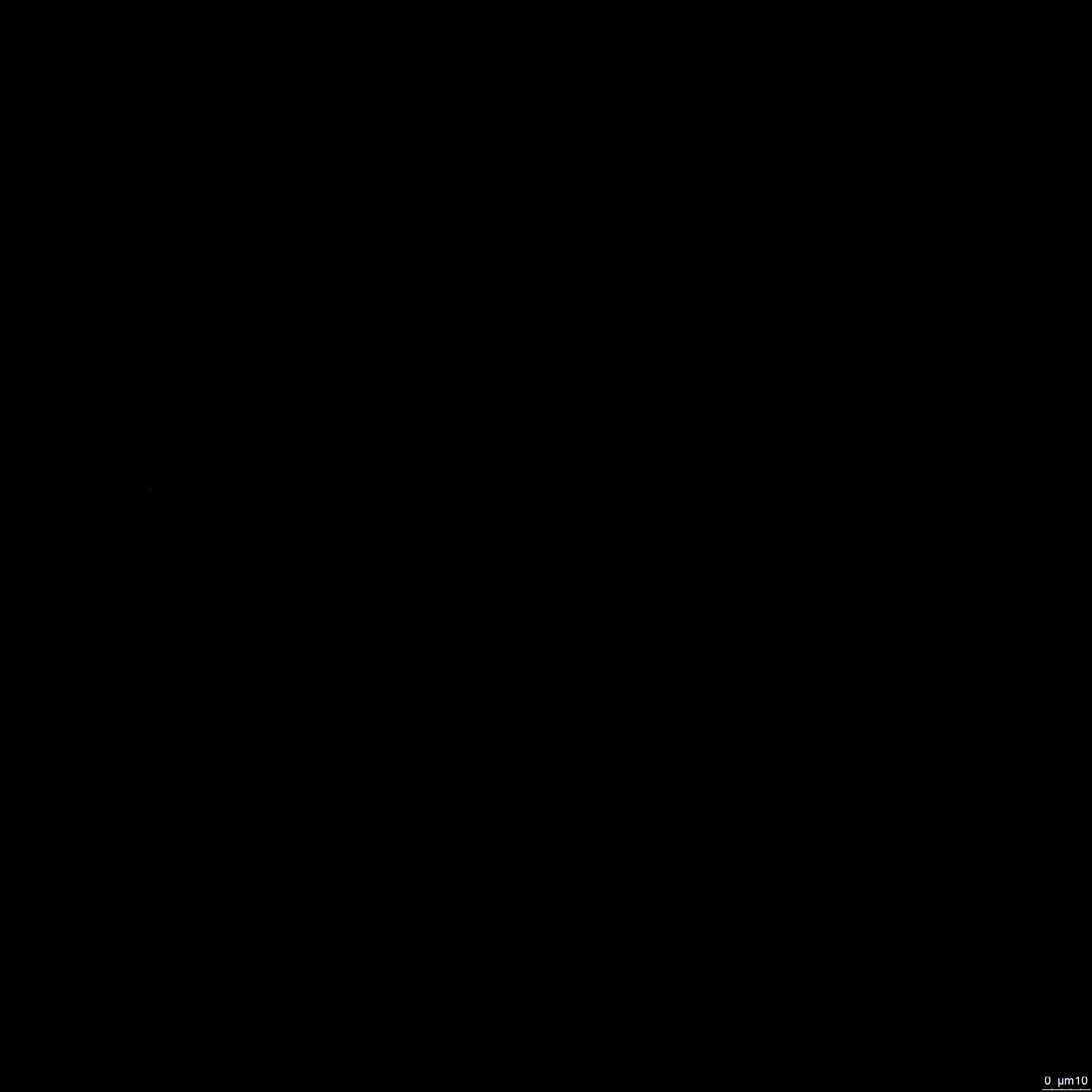

Supplement: Supplementary file 8 — Source data Fig. 1 [file 44318_2025_654_MOESM8_ESM.zip › Figure 1/1G/1G-2-mEGFP-IDR 50mM NaCl.tif]

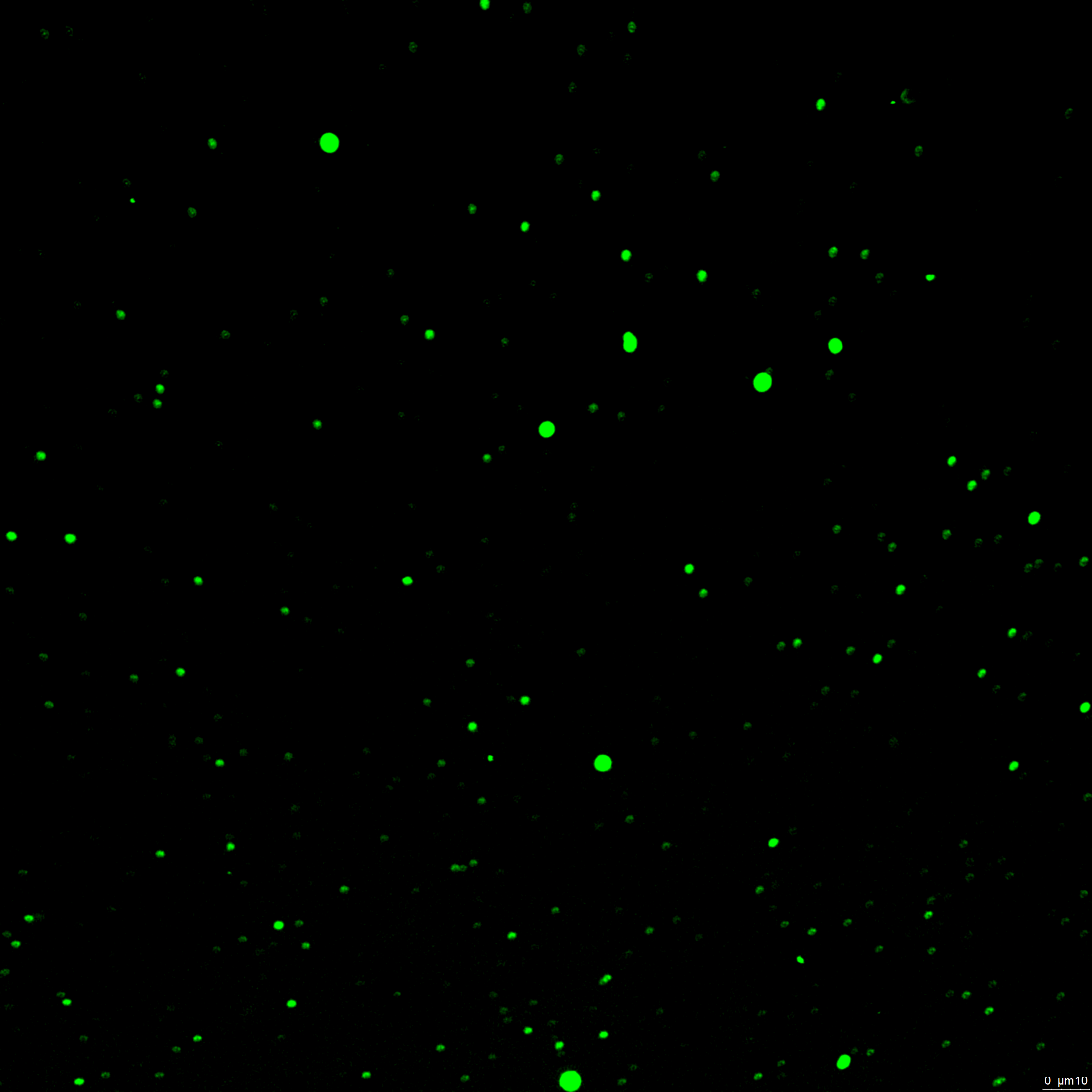

Supplement: Supplementary file 8 — Source data Fig. 1 [file 44318_2025_654_MOESM8_ESM.zip › Figure 1/1G/1G-4-mEGFP-LCR 100mM NaCl.tif]

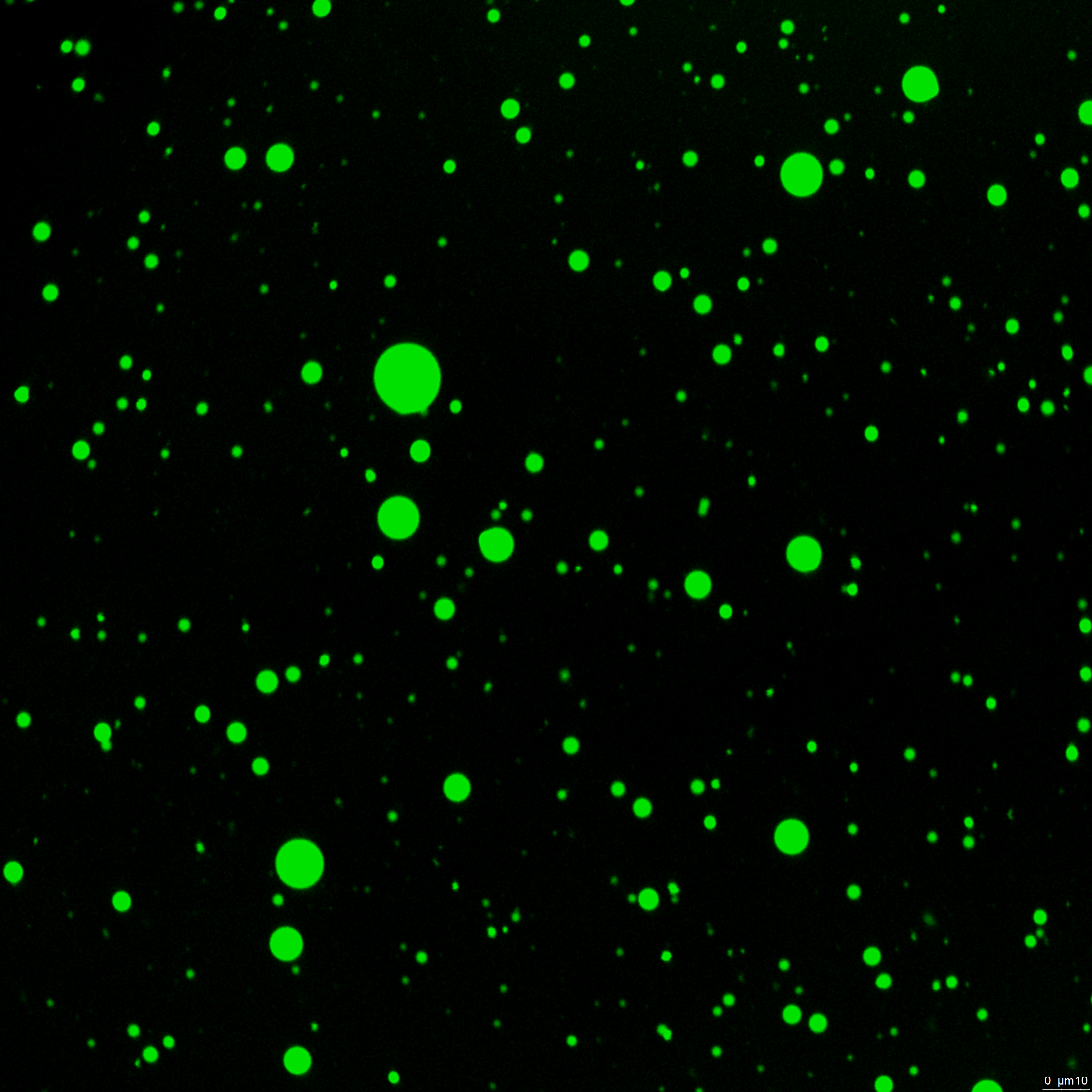

Supplement: Supplementary file 8 — Source data Fig. 1 [file 44318_2025_654_MOESM8_ESM.zip › Figure 1/1I/1L-4-mEGFP-LCR Y354W+Y356W.tif]

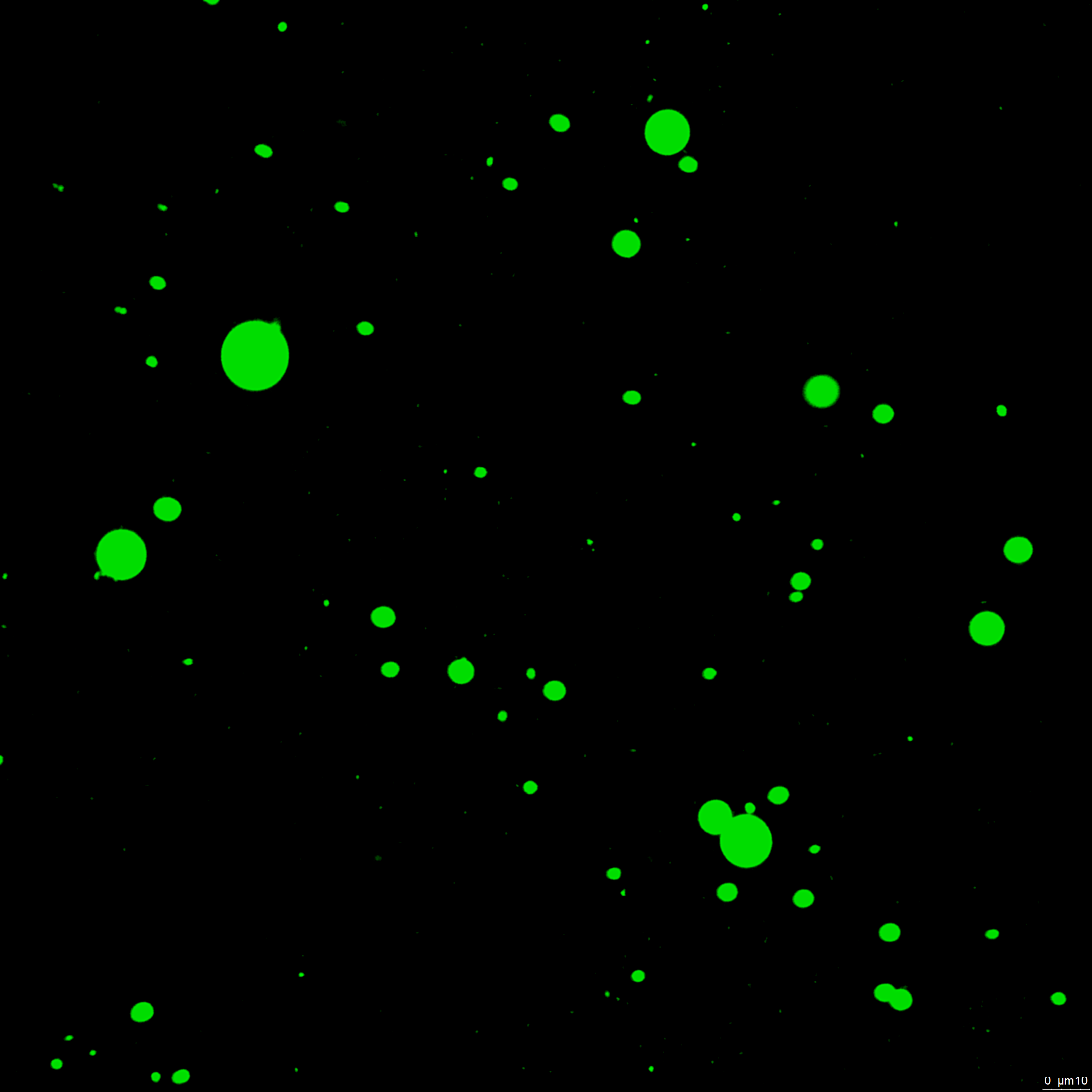

Supplement: Supplementary file 8 — Source data Fig. 1 [file 44318_2025_654_MOESM8_ESM.zip › Figure 1/1I/1L-5-mEGFP-LCR Y354F+Y356F.tif]

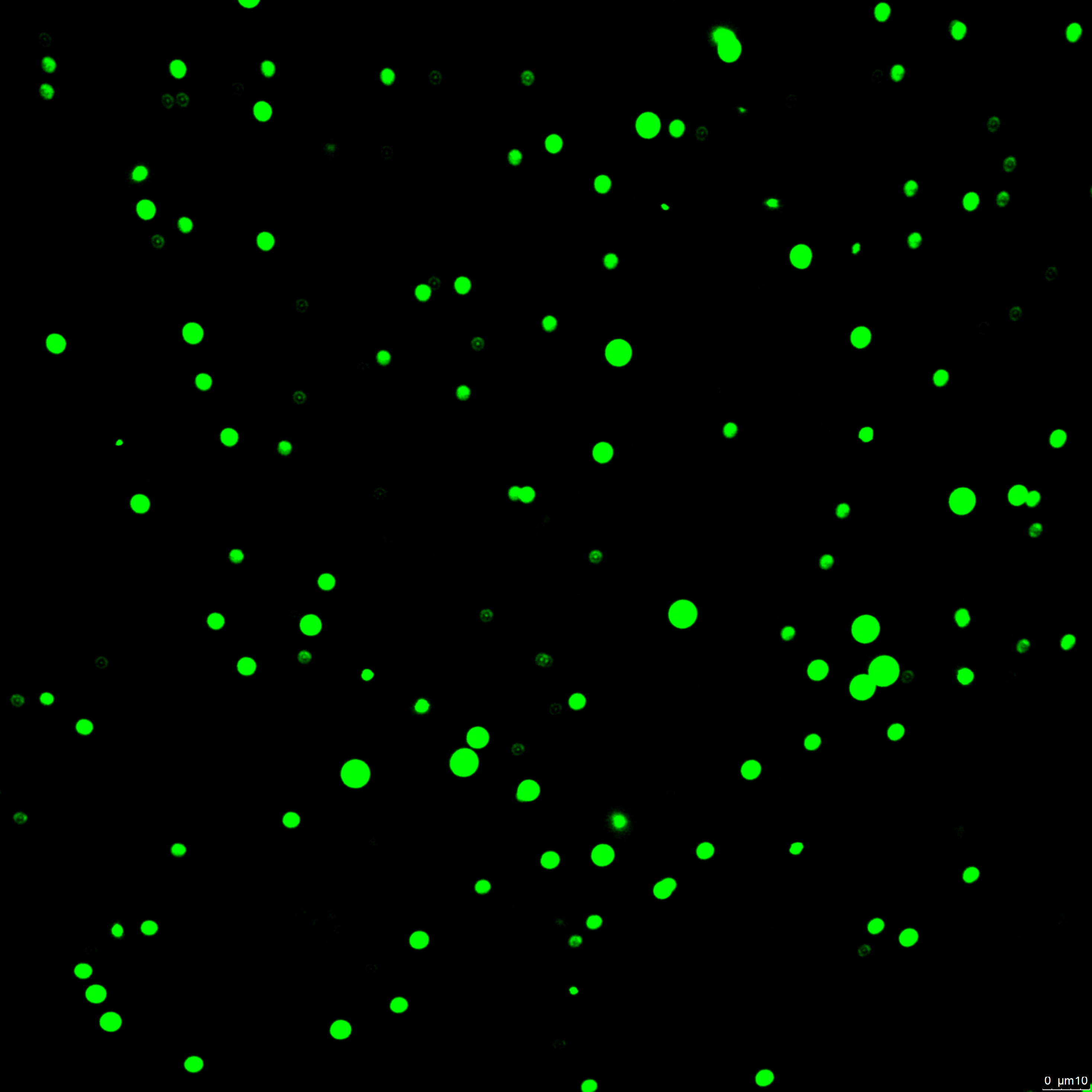

Supplement: Supplementary file 8 — Source data Fig. 1 [file 44318_2025_654_MOESM8_ESM.zip › Figure 1/1I/1L-1-mEGFP-LCR WT.tif]

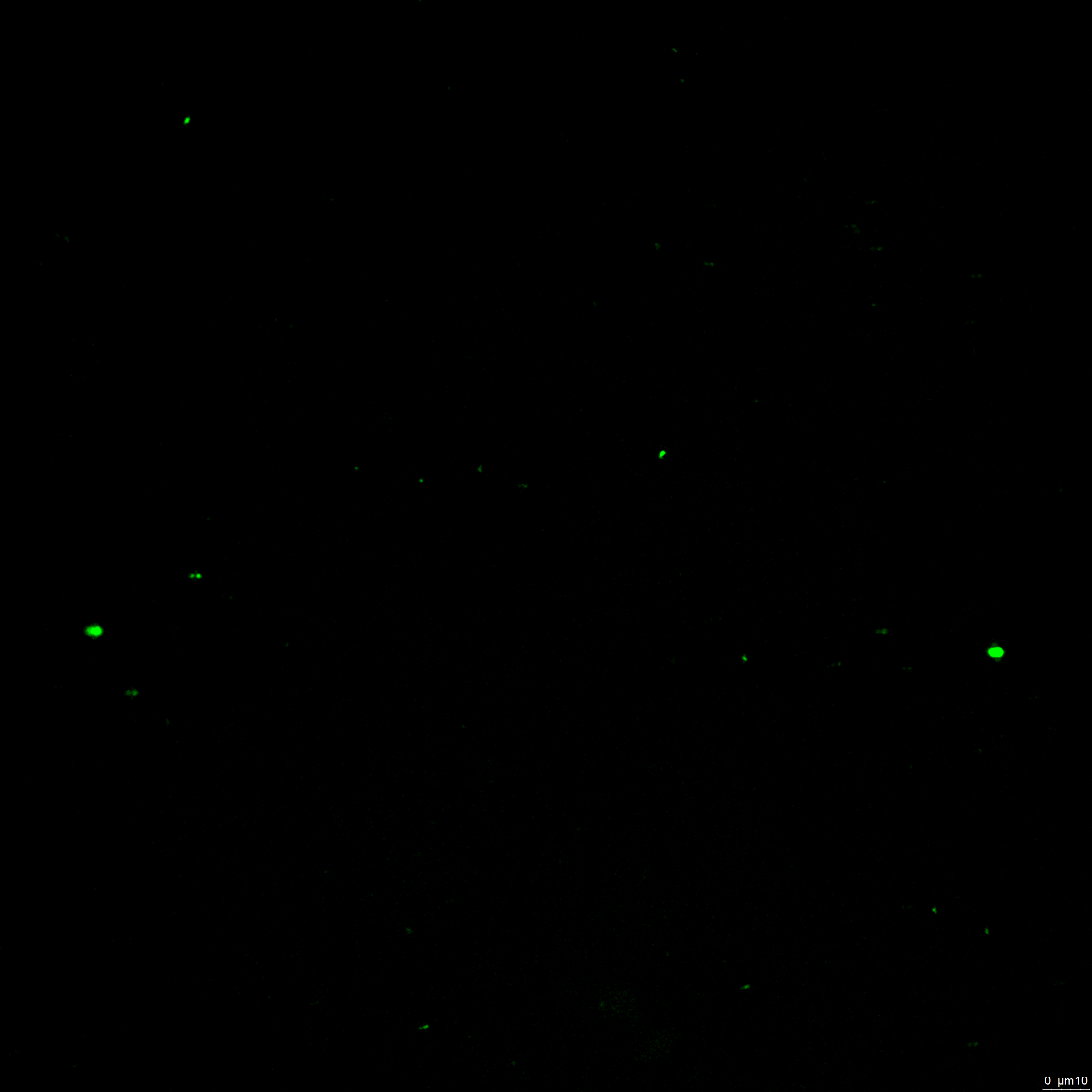

Supplement: Supplementary file 8 — Source data Fig. 1 [file 44318_2025_654_MOESM8_ESM.zip › Figure 1/1I/1L-3-mEGFP-LCR Y354A+Y356A.tif]

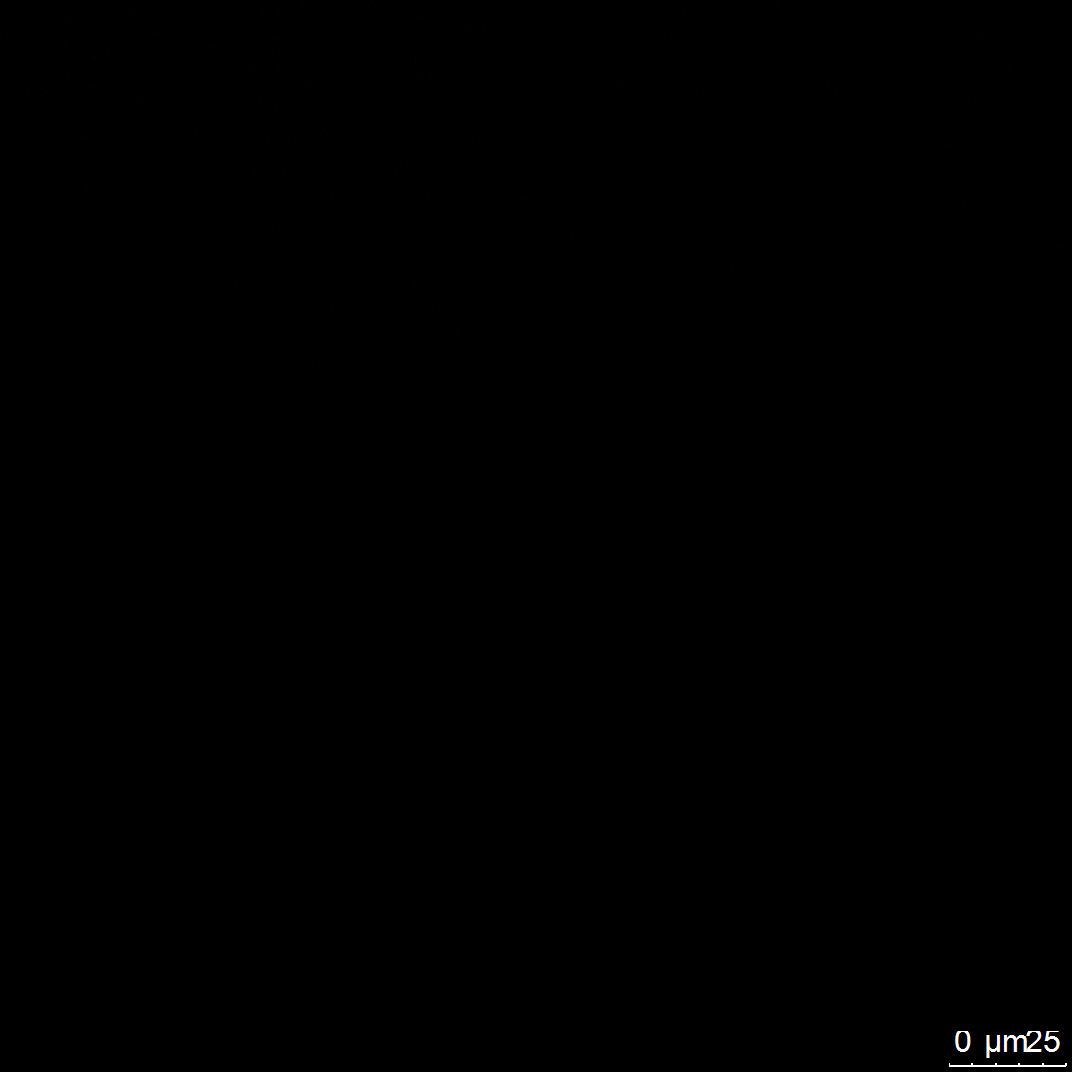

Supplement: Supplementary file 8 — Source data Fig. 1 [file 44318_2025_654_MOESM8_ESM.zip › Figure 1/1I/1L-2-mEGFP-LCR ╬ö5k.tif]

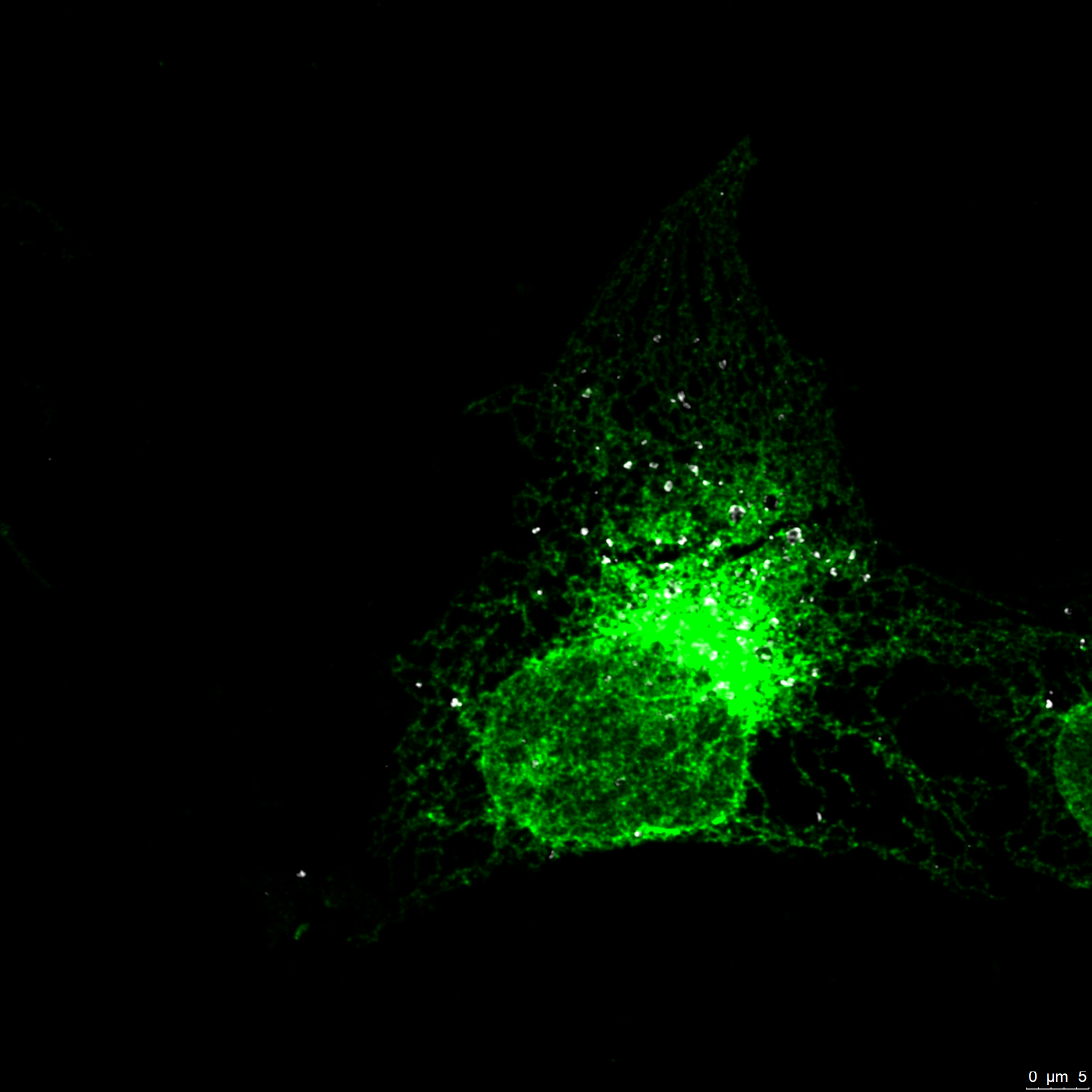

Supplement: Supplementary file 8 — Source data Fig. 1 [file 44318_2025_654_MOESM8_ESM.zip › Figure 1/1K/1K-5-U2OS AREL1 KO-AREL1(Y354A+Y356A)-EGFP+PLA merge.tif]

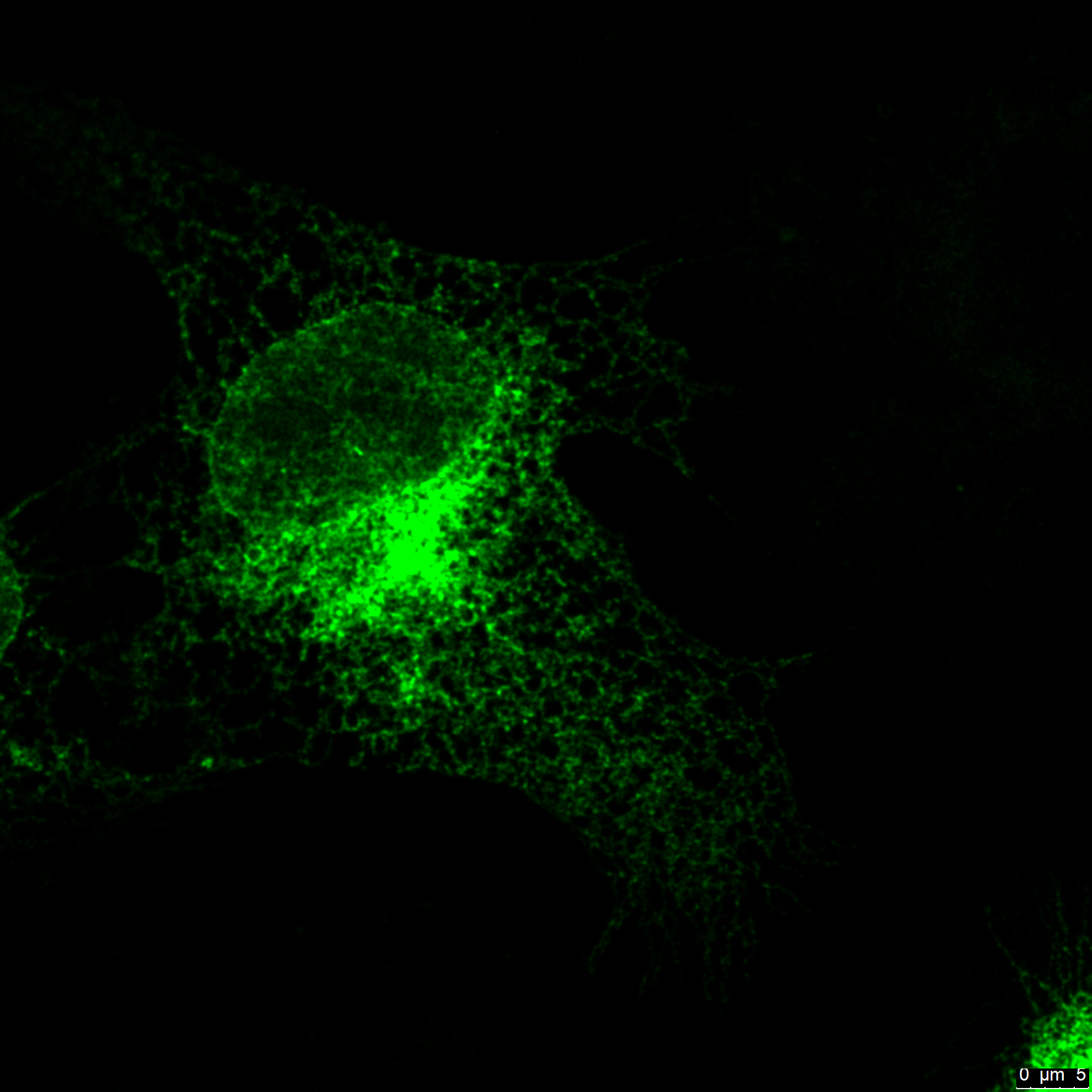

Supplement: Supplementary file 8 — Source data Fig. 1 [file 44318_2025_654_MOESM8_ESM.zip › Figure 1/1K/1K-4-U2OS AREL1 KO-AREL1(╬öLCR)-EGFP.tif]

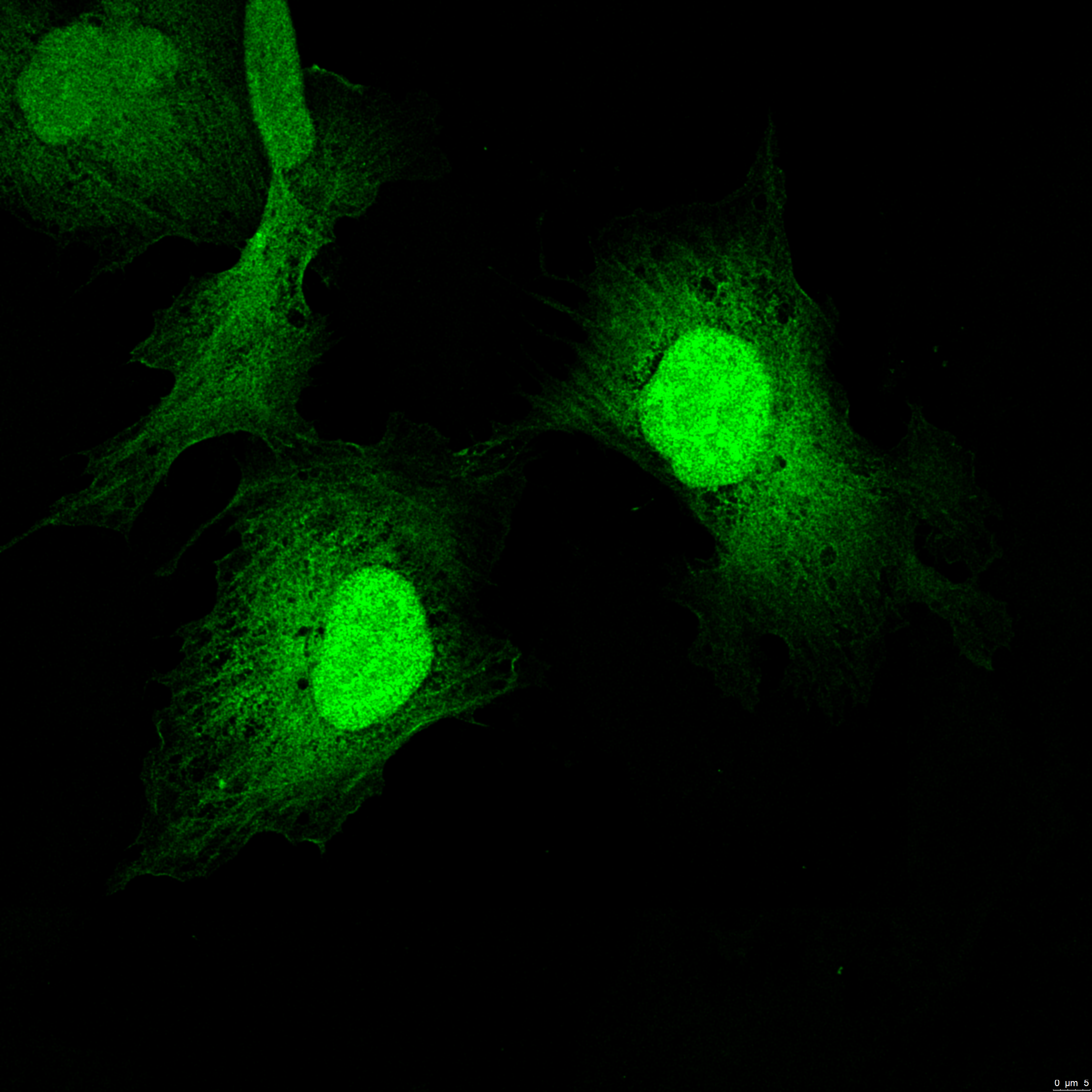

Supplement: Supplementary file 8 — Source data Fig. 1 [file 44318_2025_654_MOESM8_ESM.zip › Figure 1/1K/1K-2-U2OS AREL1 KO-EGFP.tif]

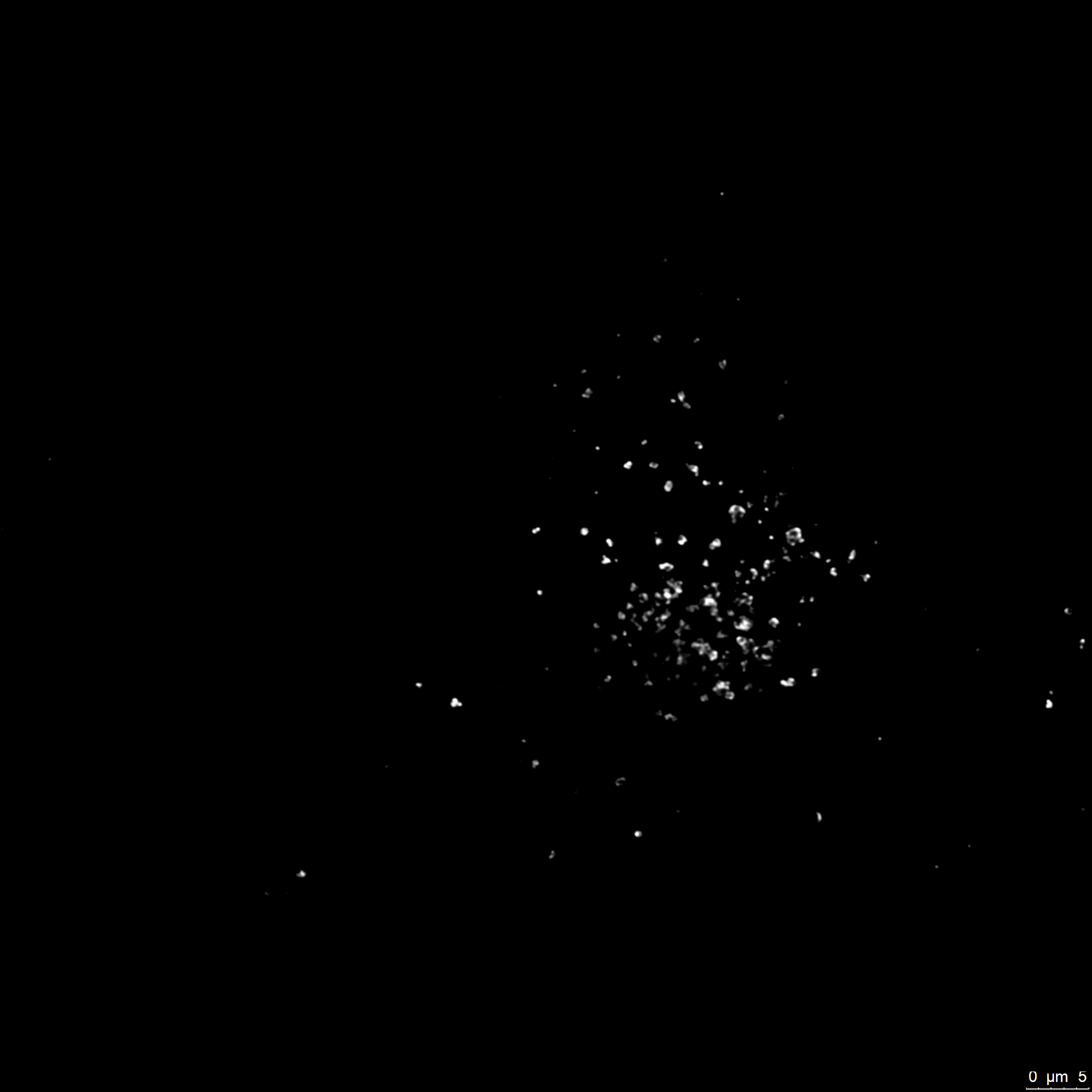

Supplement: Supplementary file 8 — Source data Fig. 1 [file 44318_2025_654_MOESM8_ESM.zip › Figure 1/1K/1K-5-U2OS AREL1 KO-PLA.tif]

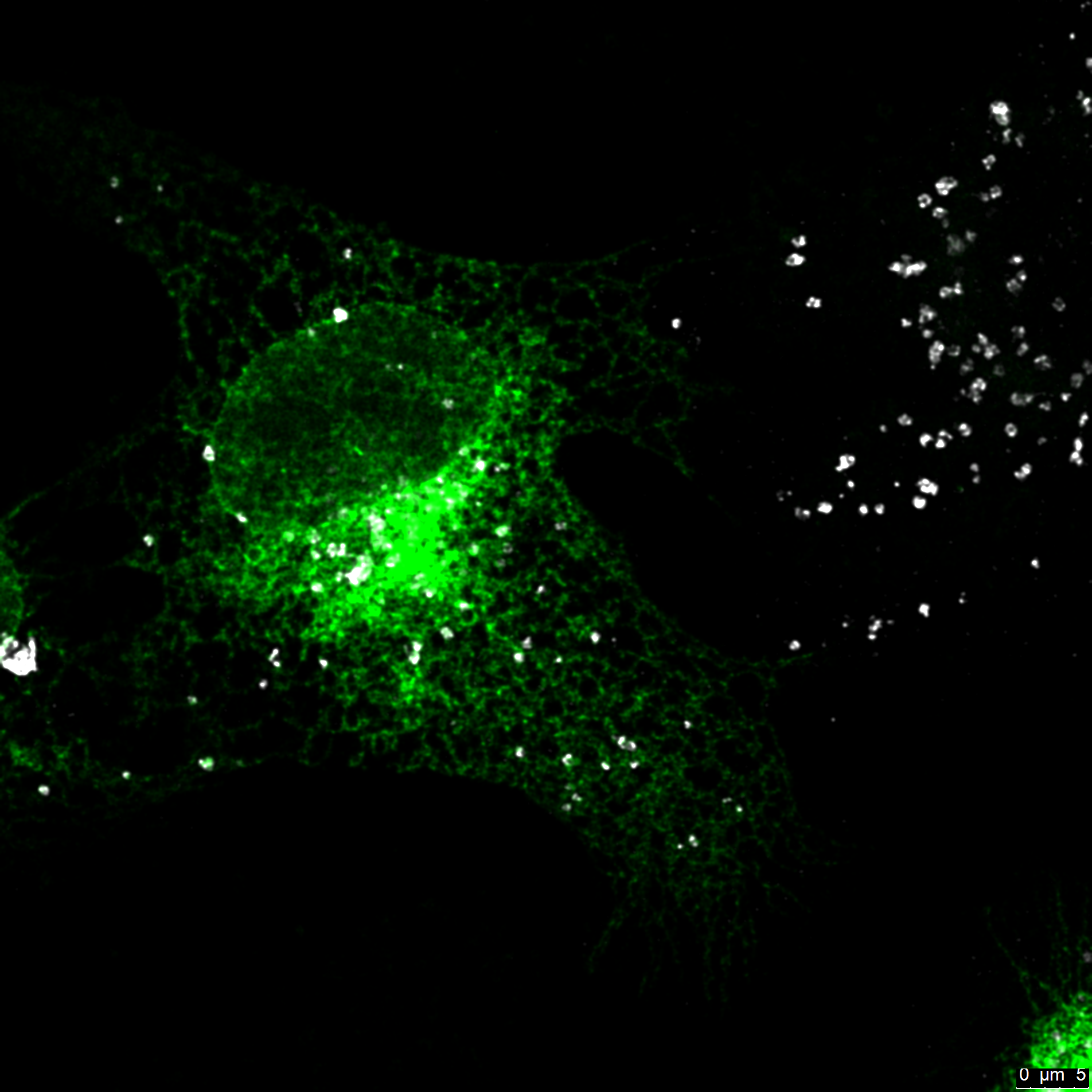

Supplement: Supplementary file 8 — Source data Fig. 1 [file 44318_2025_654_MOESM8_ESM.zip › Figure 1/1K/1K-4-U2OS AREL1 KO-AREL1(╬öLCR)-EGFP+PLA merge.tif]

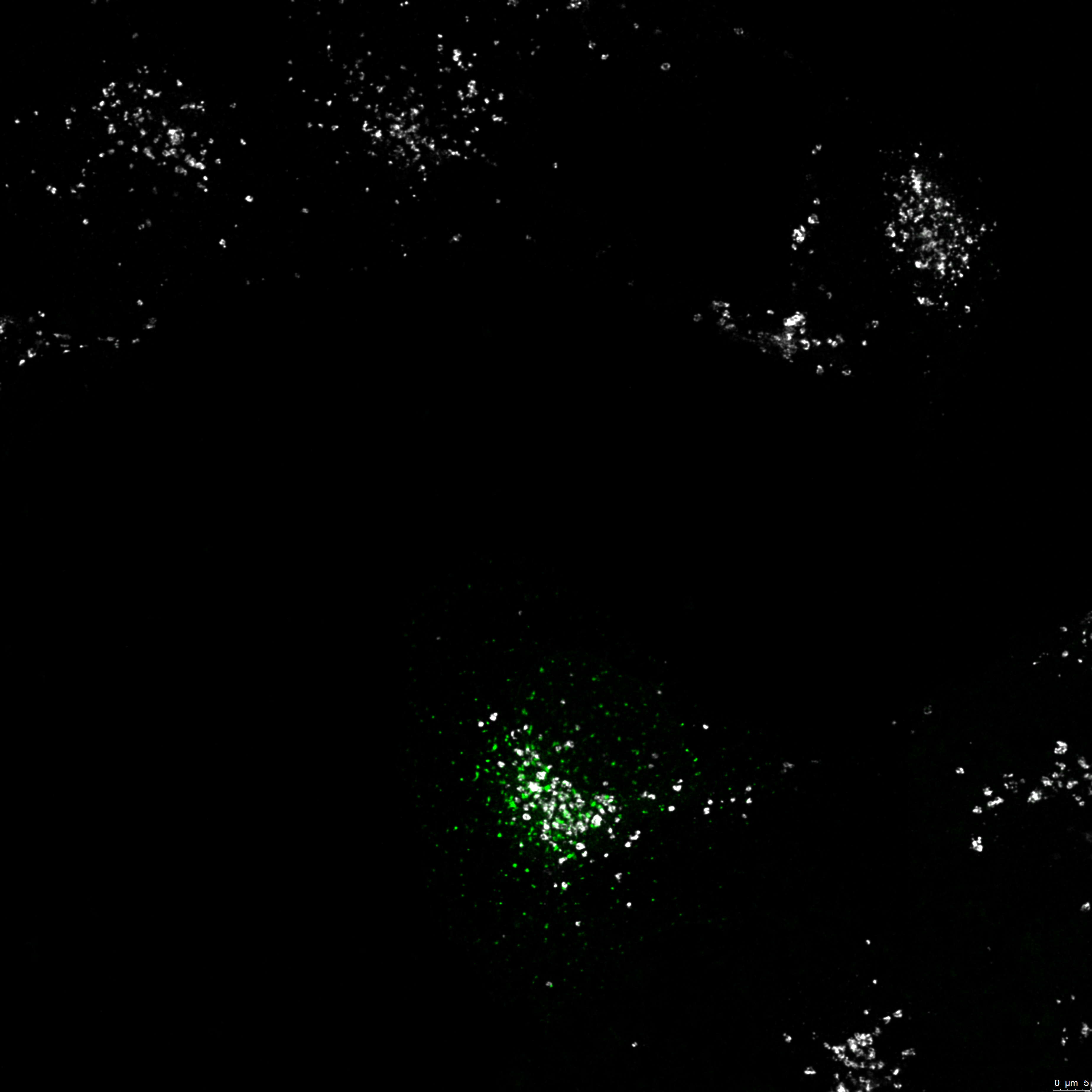

Supplement: Supplementary file 8 — Source data Fig. 1 [file 44318_2025_654_MOESM8_ESM.zip › Figure 1/1K/1K-3-U2OS AREL1 KO-AREL1(WT)-EGFP+PLA merge.tif]

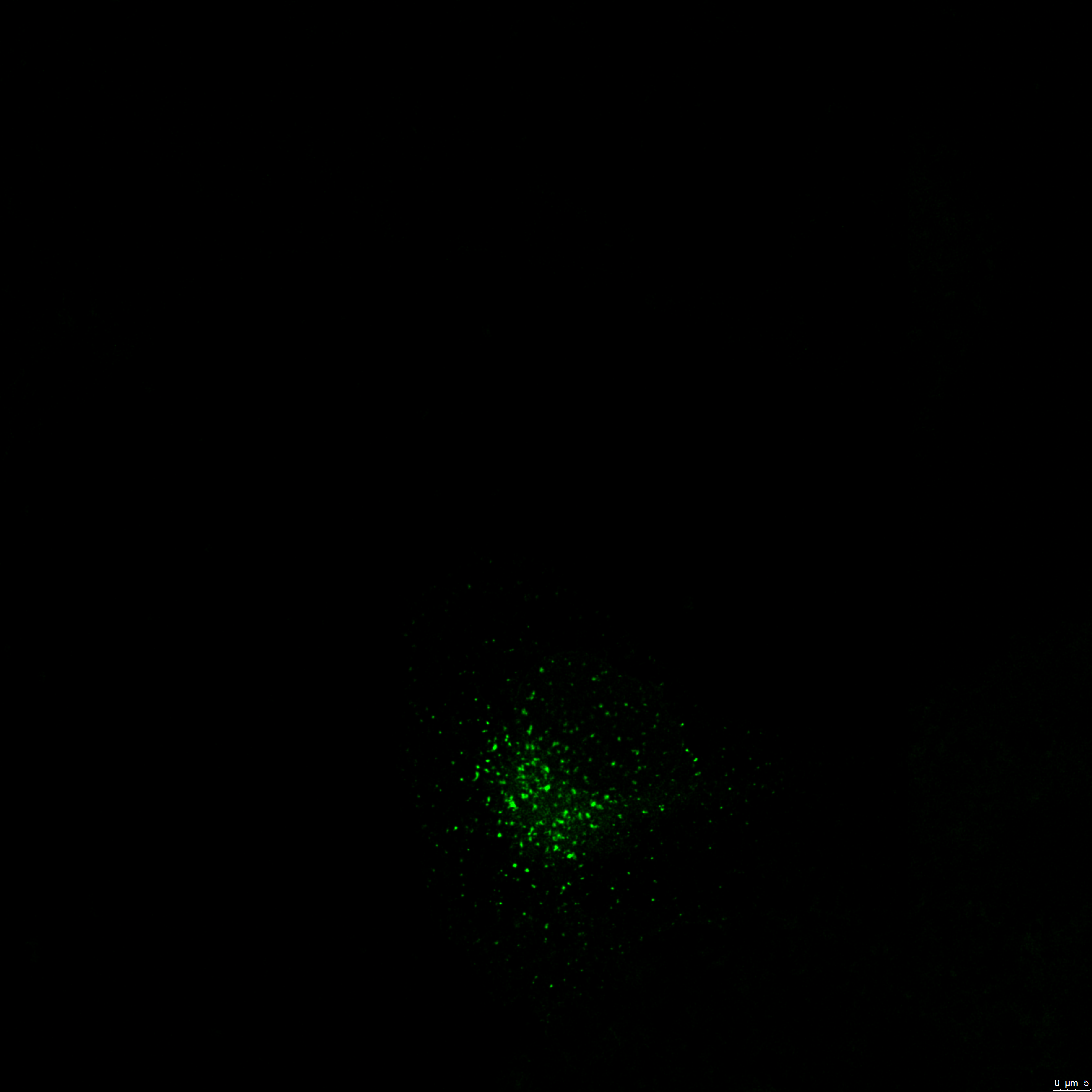

Supplement: Supplementary file 8 — Source data Fig. 1 [file 44318_2025_654_MOESM8_ESM.zip › Figure 1/1K/1K-3-U2OS AREL1 KO-AREL1(WT)-EGFP.tif]

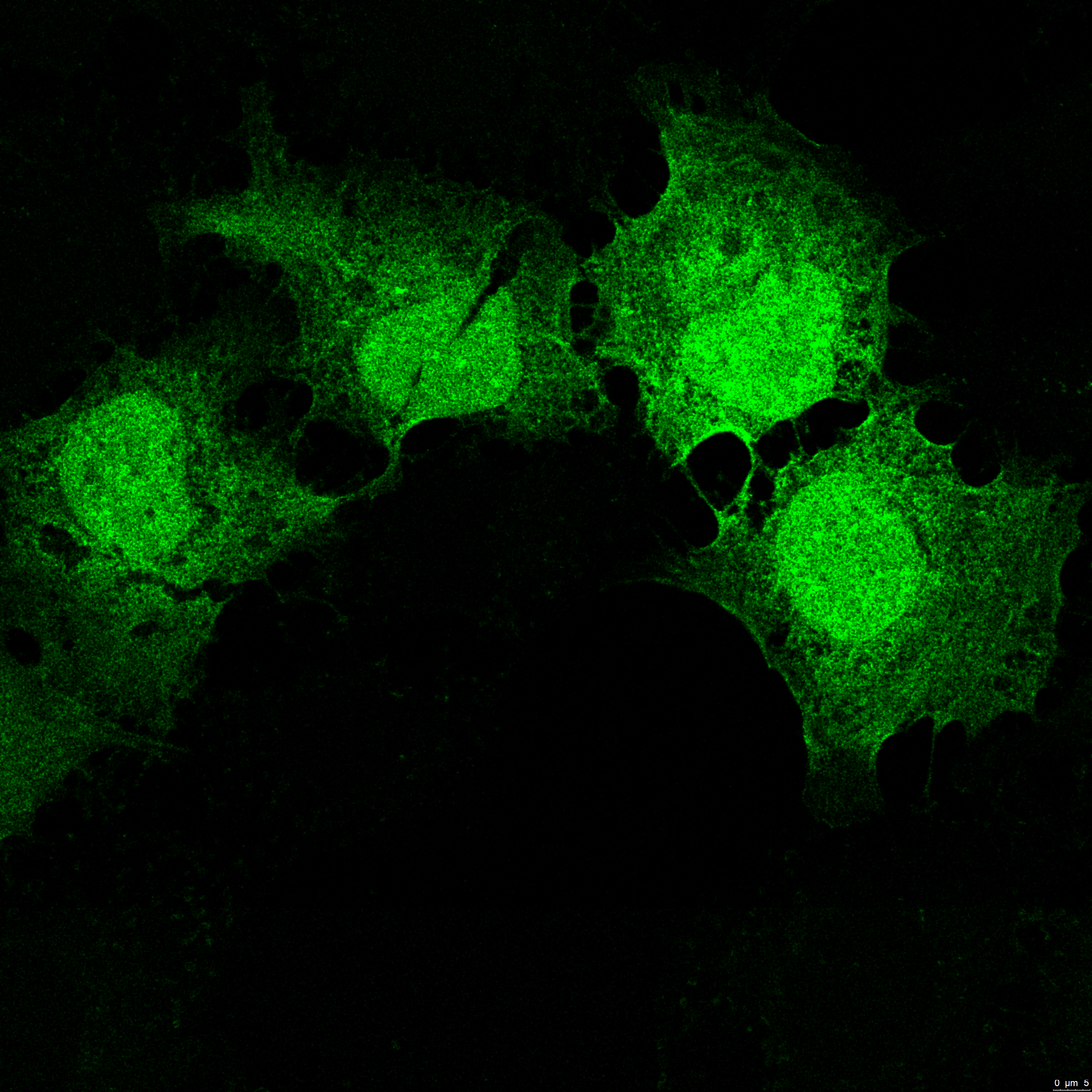

Supplement: Supplementary file 8 — Source data Fig. 1 [file 44318_2025_654_MOESM8_ESM.zip › Figure 1/1K/1K-1-U2OS WT-EGFP.tif]

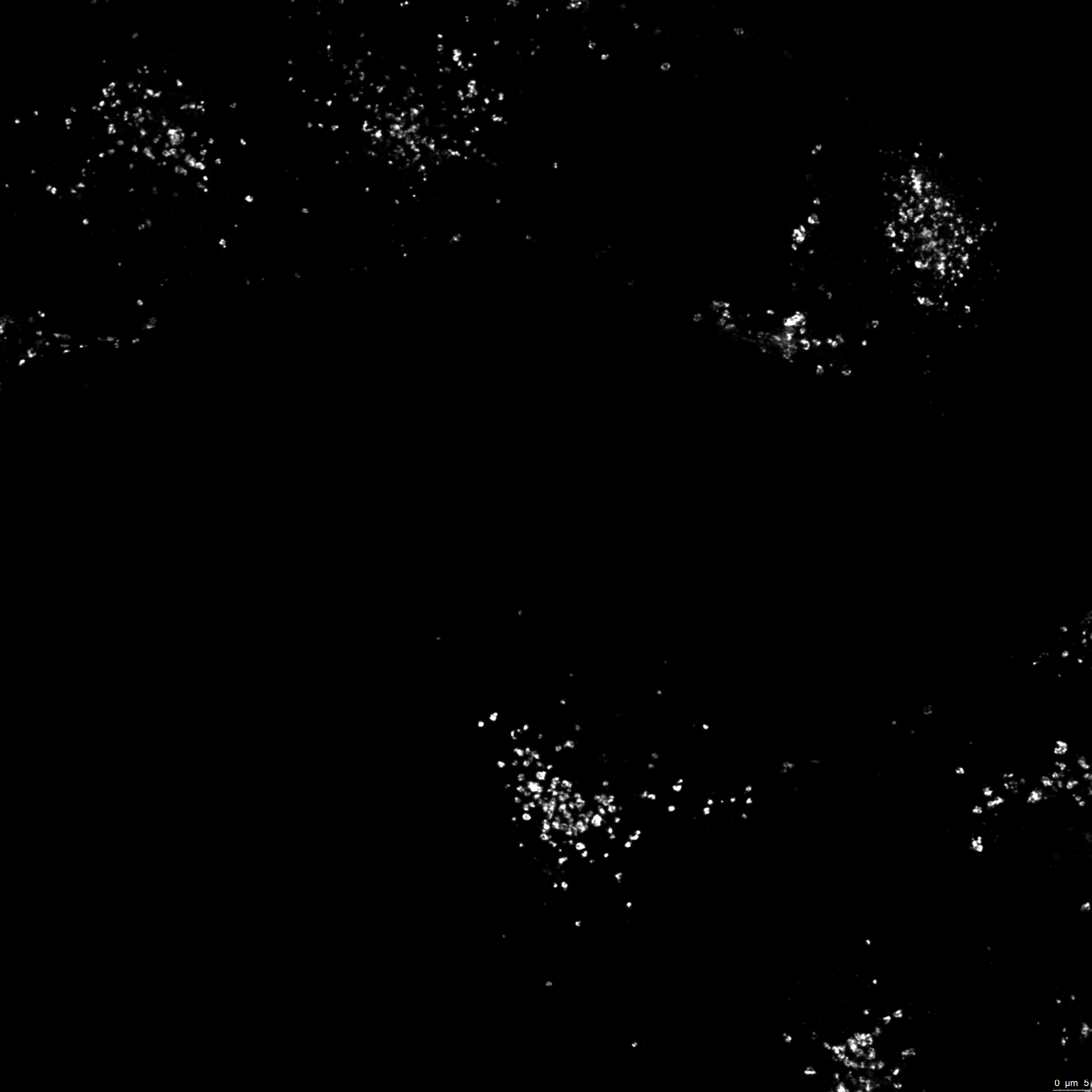

Supplement: Supplementary file 8 — Source data Fig. 1 [file 44318_2025_654_MOESM8_ESM.zip › Figure 1/1K/1K-3-U2OS AREL1 KO-PLA.tif]

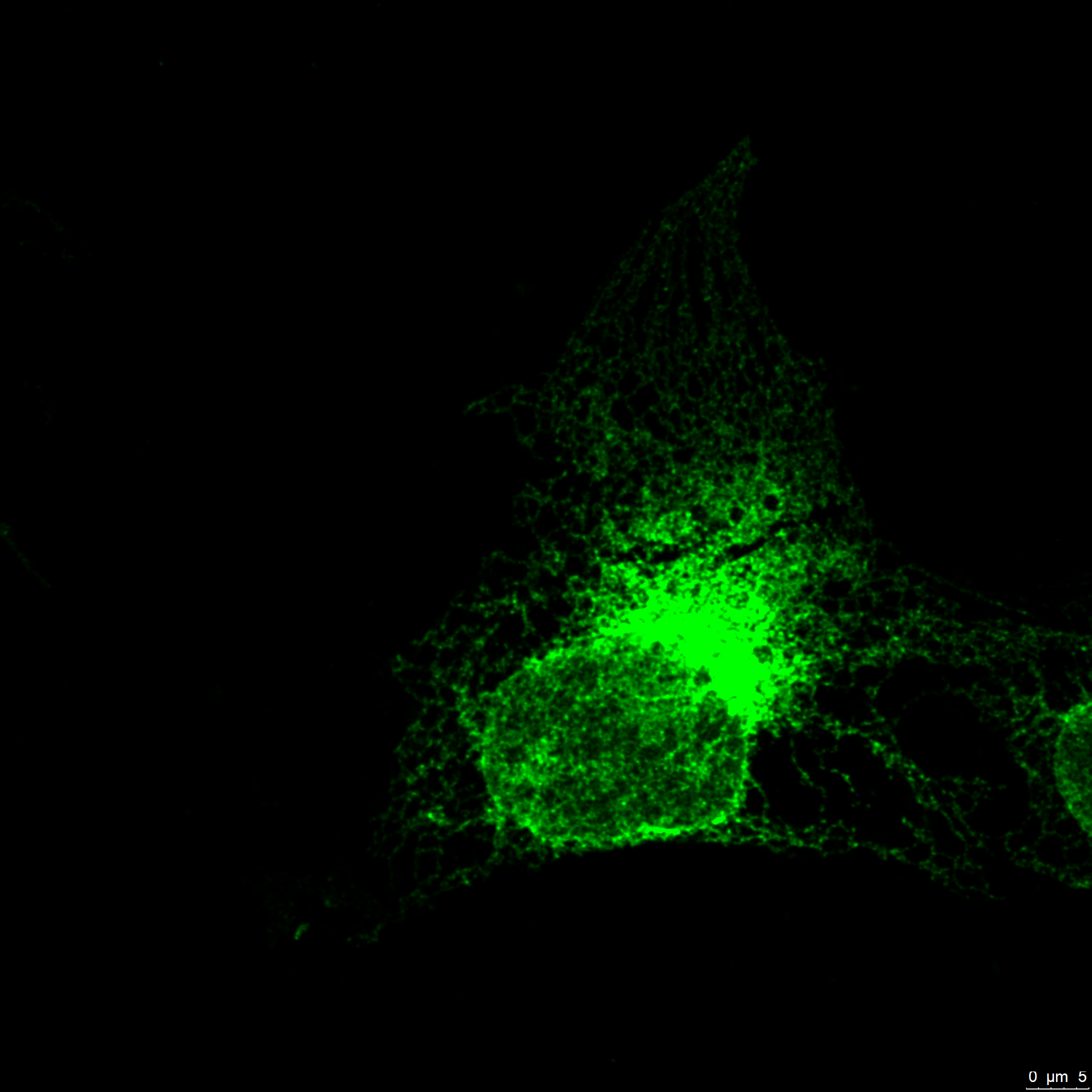

Supplement: Supplementary file 8 — Source data Fig. 1 [file 44318_2025_654_MOESM8_ESM.zip › Figure 1/1K/1K-5-U2OS AREL1 KO-AREL1(Y354A+Y356A)-EGFP.tif]

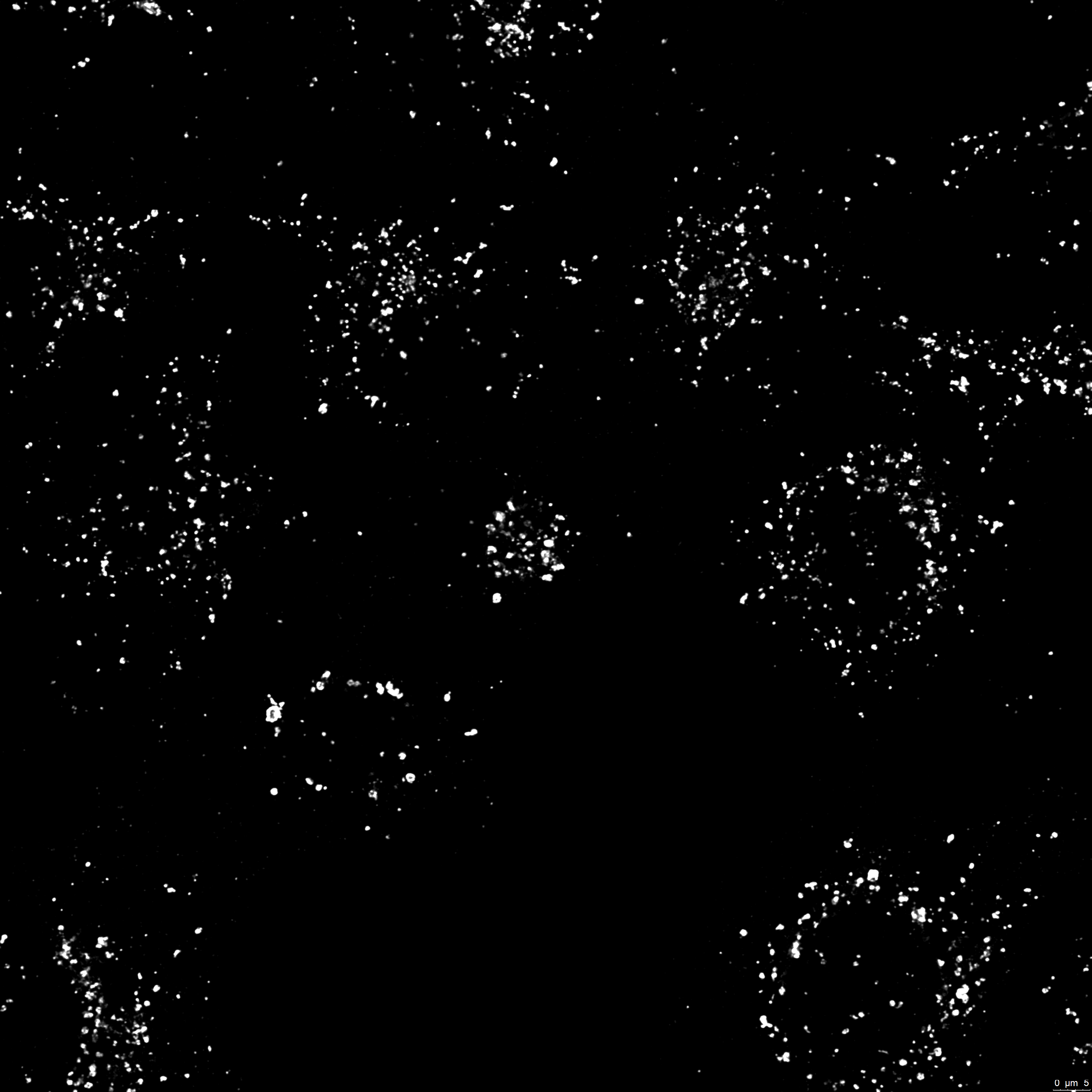

Supplement: Supplementary file 8 — Source data Fig. 1 [file 44318_2025_654_MOESM8_ESM.zip › Figure 1/1K/1K-1-U2OS WT-PLA.tif]

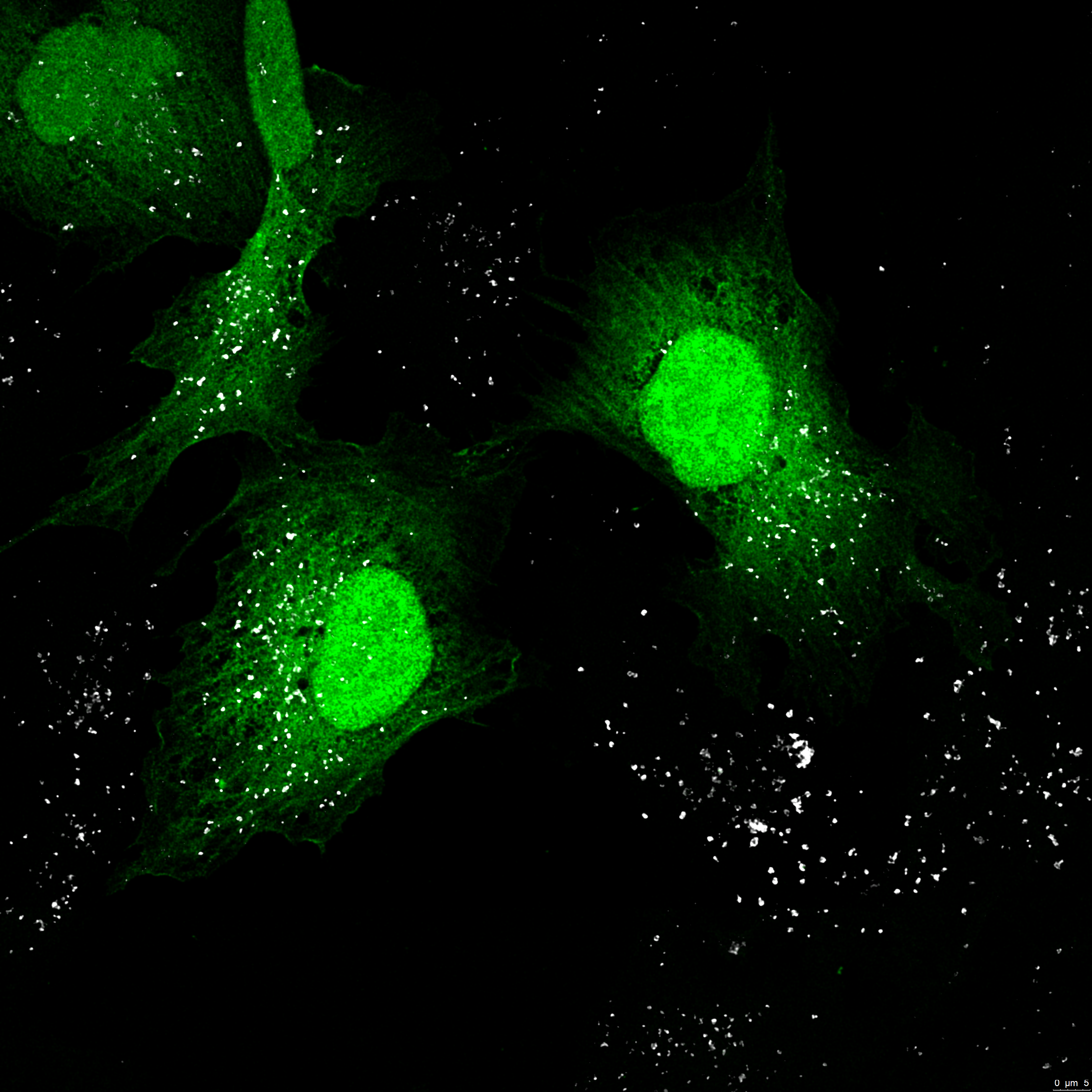

Supplement: Supplementary file 8 — Source data Fig. 1 [file 44318_2025_654_MOESM8_ESM.zip › Figure 1/1K/1K-2-U2OS AREL1 KO-EGFP+PLA merge.tif]

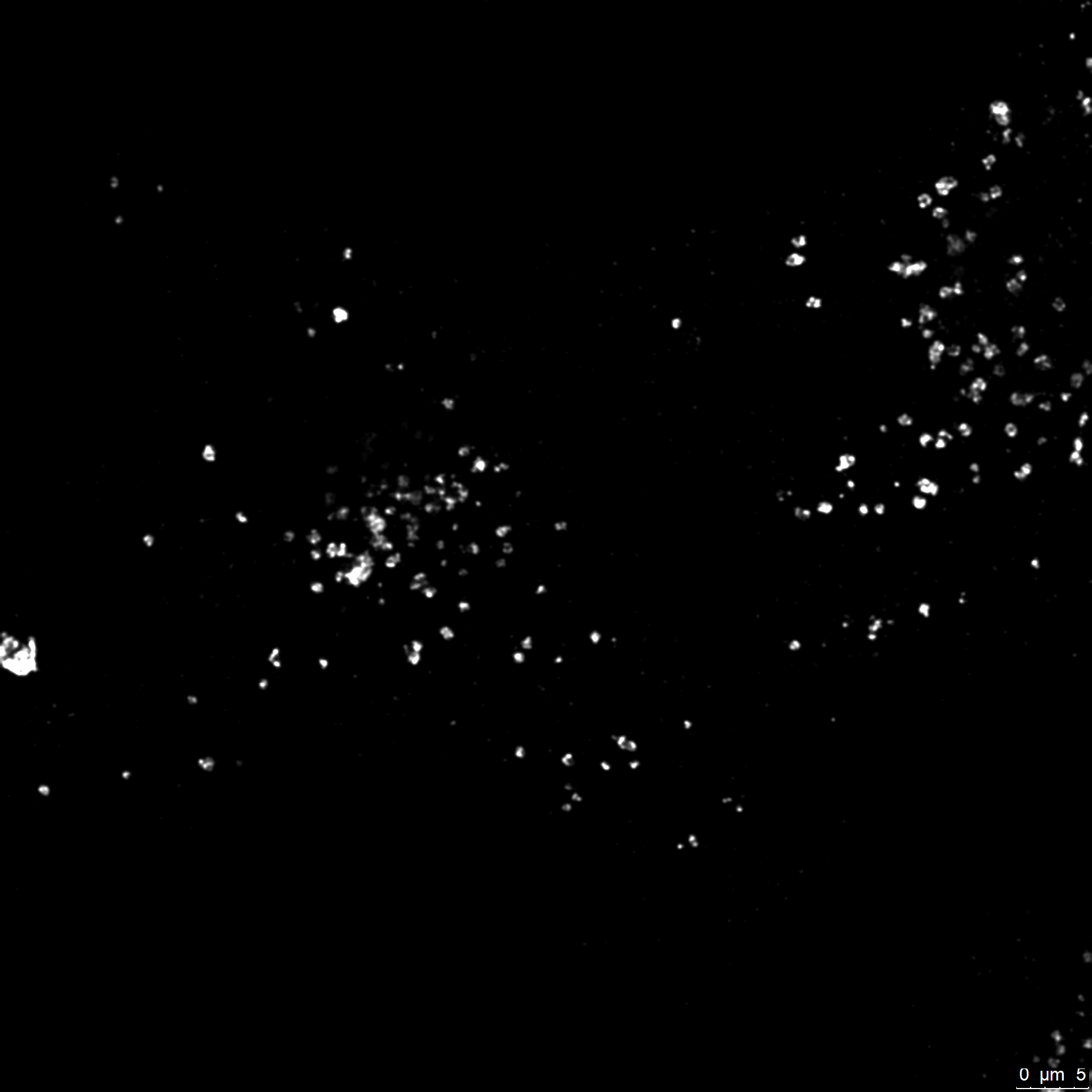

Supplement: Supplementary file 8 — Source data Fig. 1 [file 44318_2025_654_MOESM8_ESM.zip › Figure 1/1K/1K-4-U2OS AREL1 KO-PLA.tif]

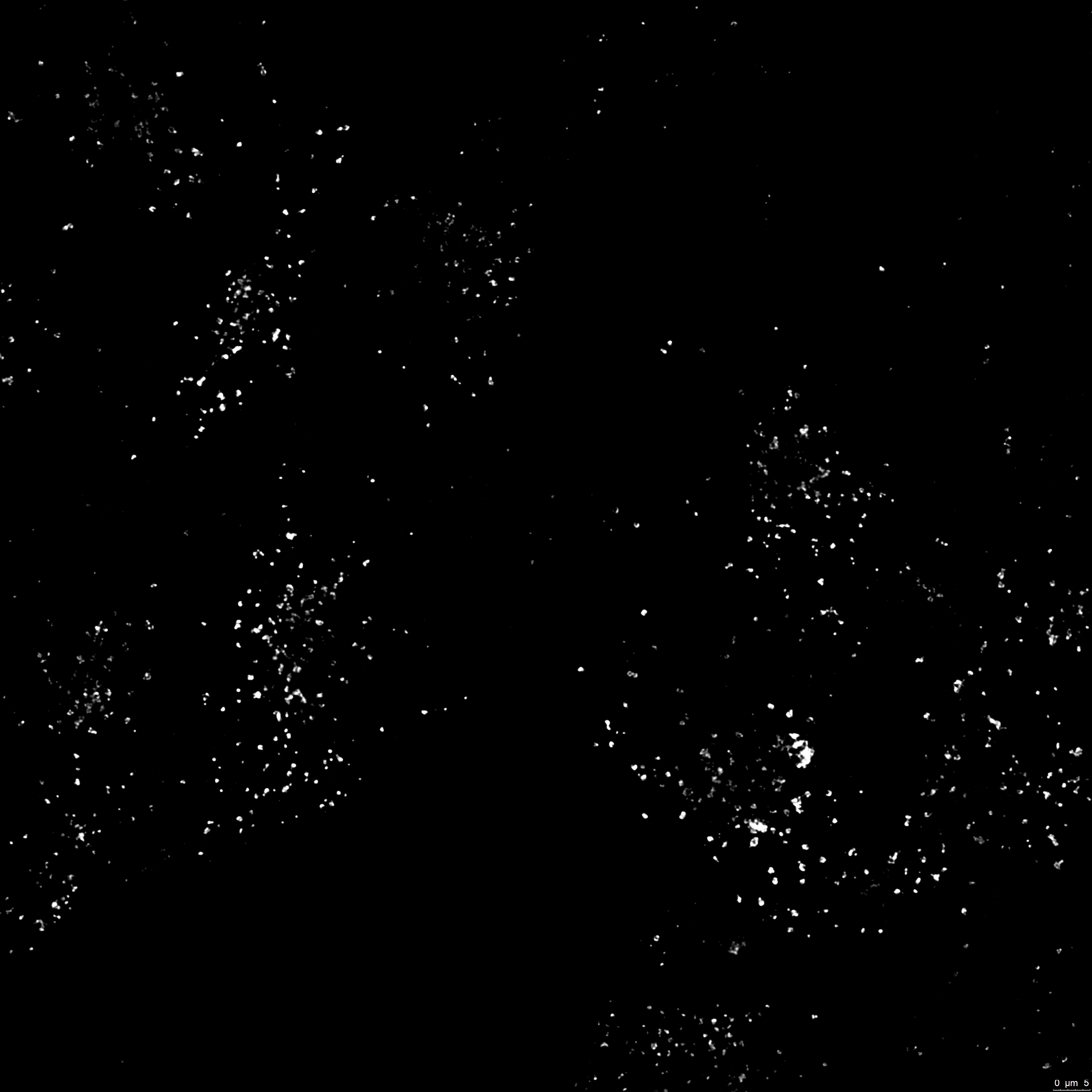

Supplement: Supplementary file 8 — Source data Fig. 1 [file 44318_2025_654_MOESM8_ESM.zip › Figure 1/1K/1K-2-U2OS AREL1 KO-PLA.tif]

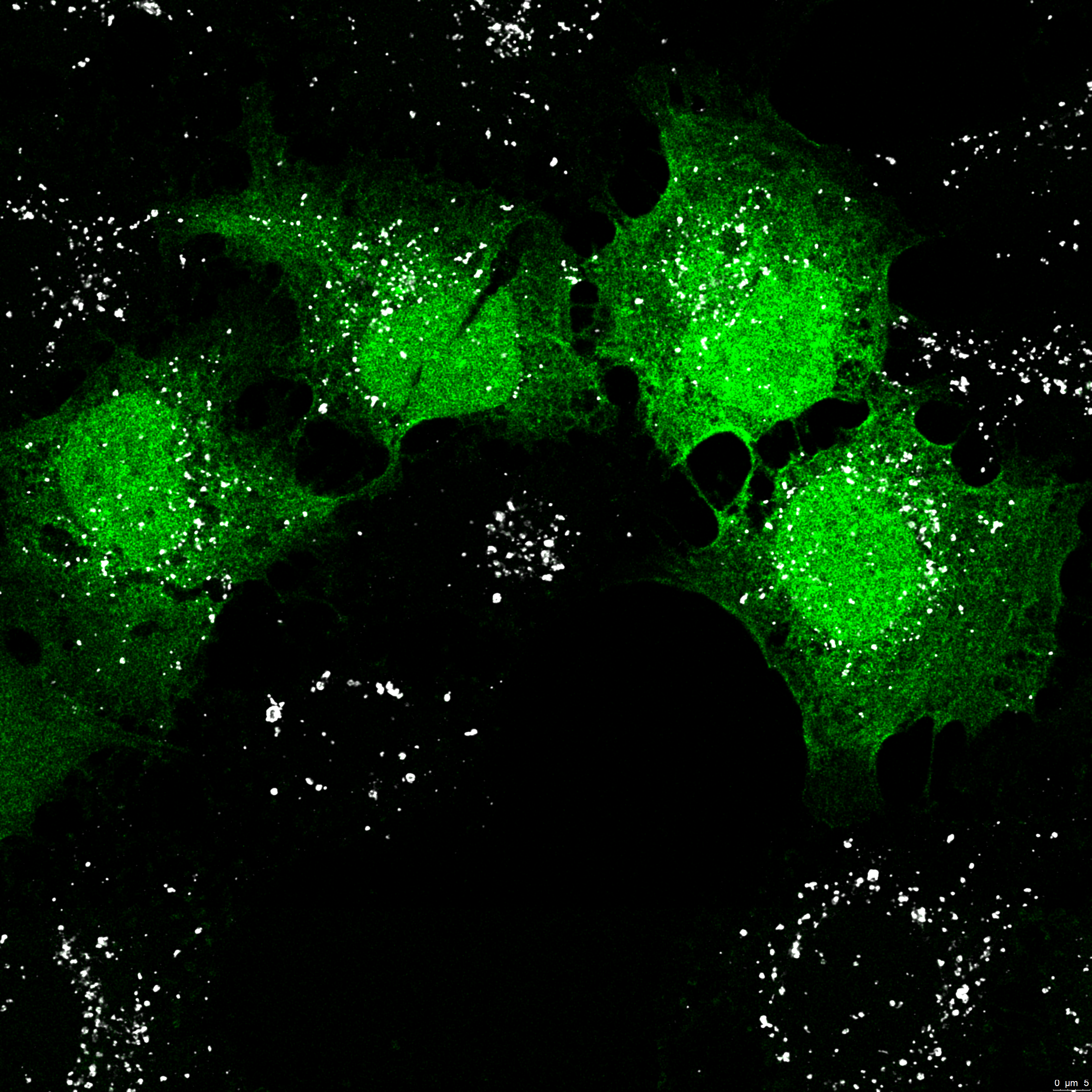

Supplement: Supplementary file 8 — Source data Fig. 1 [file 44318_2025_654_MOESM8_ESM.zip › Figure 1/1K/1K-1-U2OS WT-EGFP+PLA merge.tif]

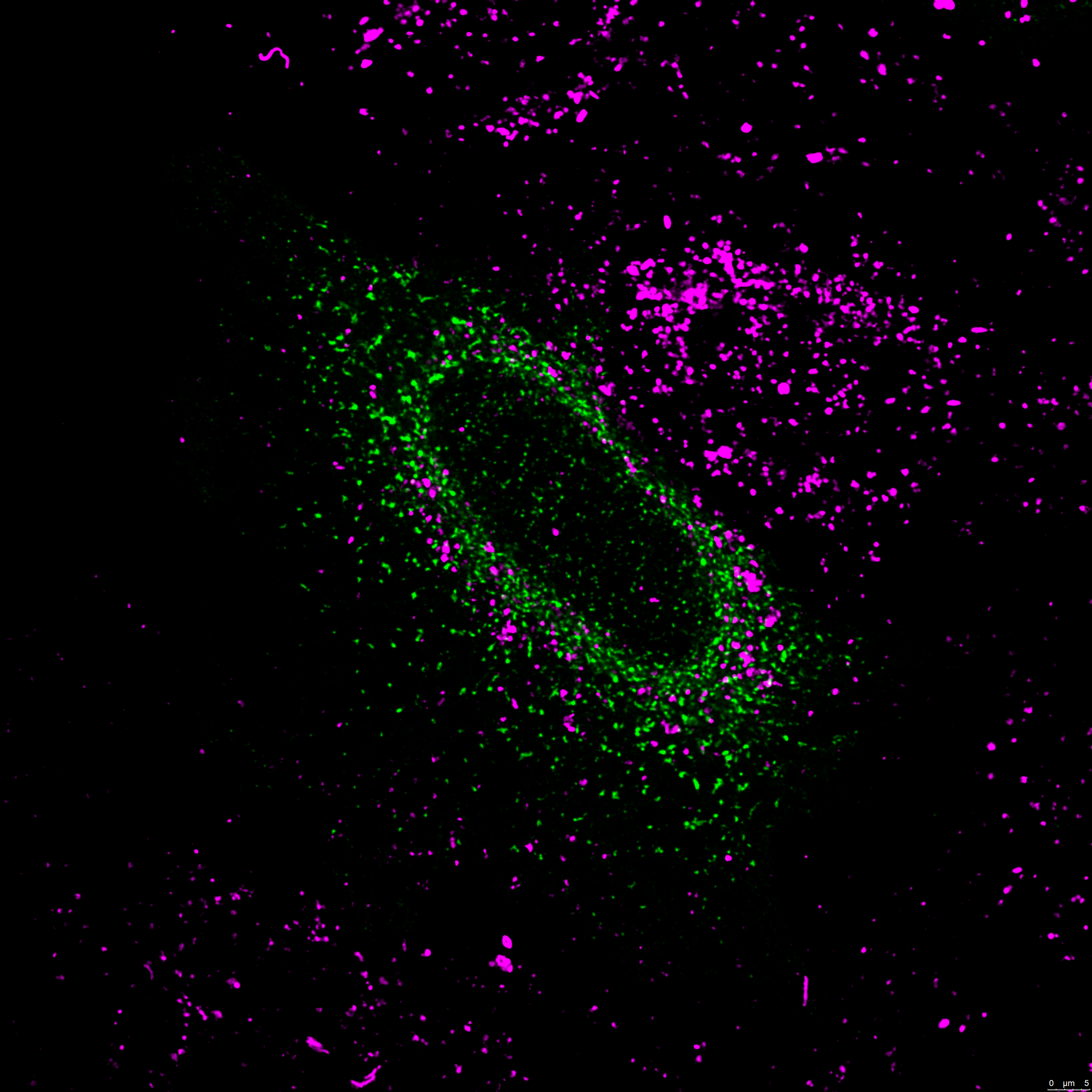

Supplement: Supplementary file 8 — Source data Fig. 1 [file 44318_2025_654_MOESM8_ESM.zip › Figure 1/1E/1E-╬öhinge region.tif]

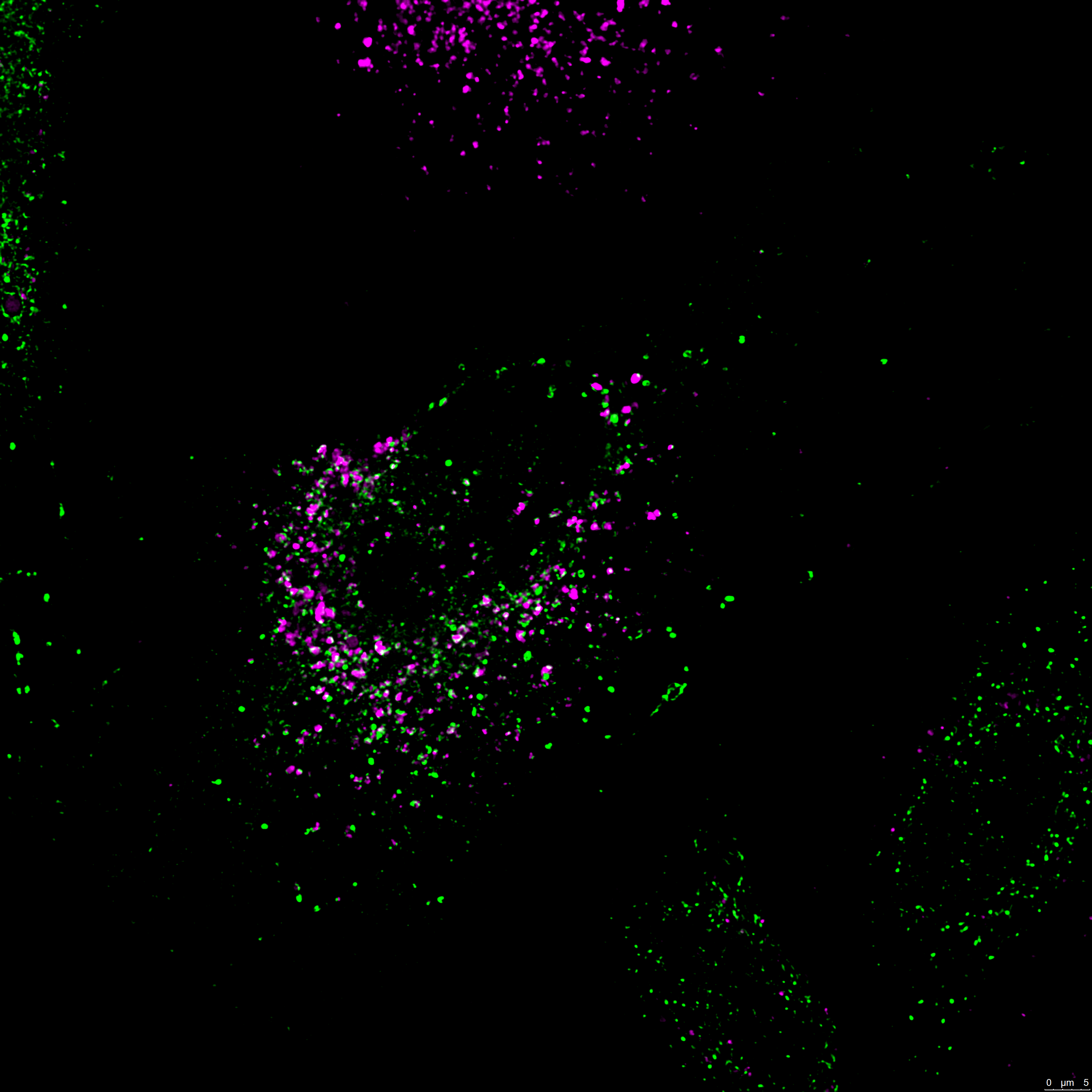

Supplement: Supplementary file 8 — Source data Fig. 1 [file 44318_2025_654_MOESM8_ESM.zip › Figure 1/1E/1E-WT.tif]

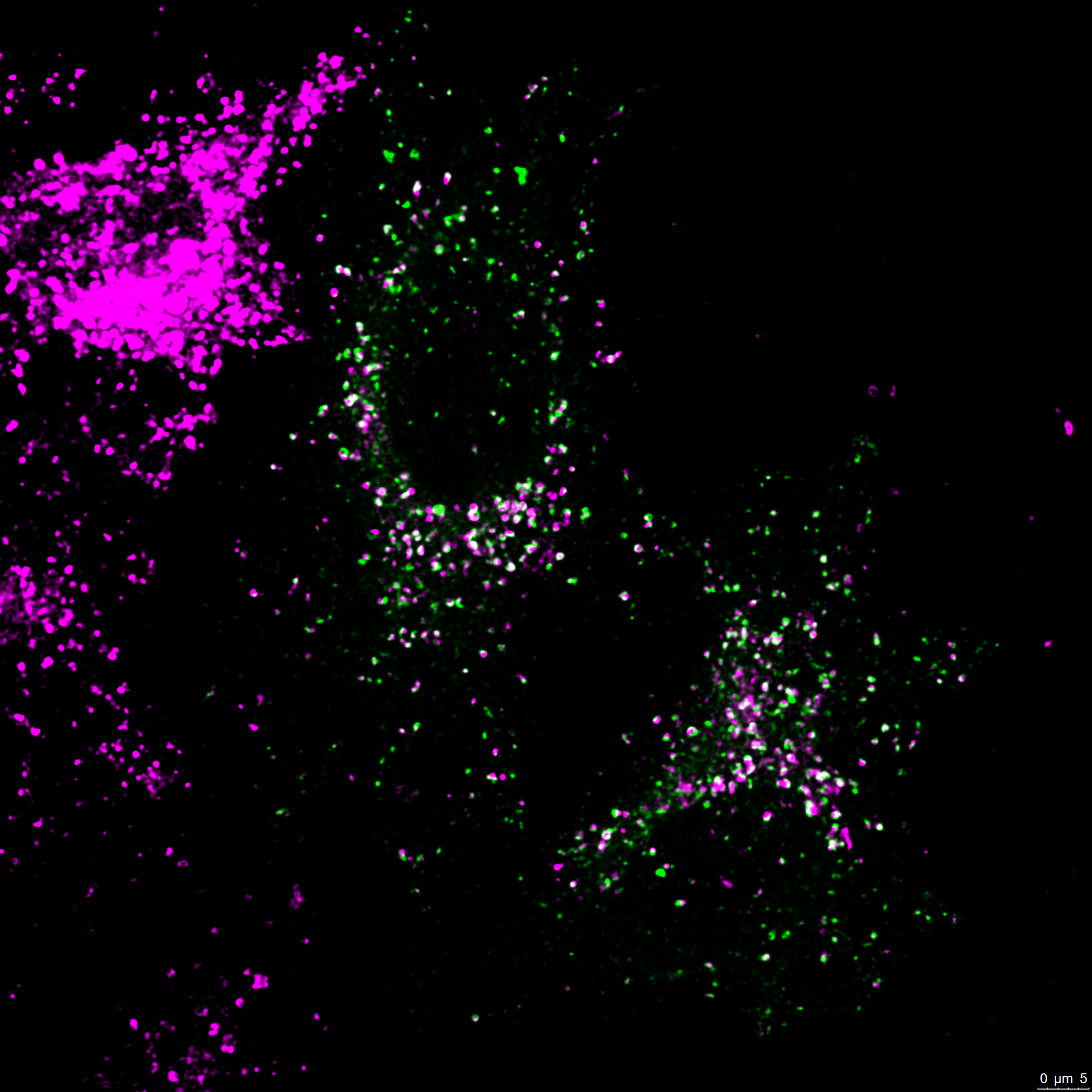

Supplement: Supplementary file 8 — Source data Fig. 1 [file 44318_2025_654_MOESM8_ESM.zip › Figure 1/1E/1E-C790A.tif]

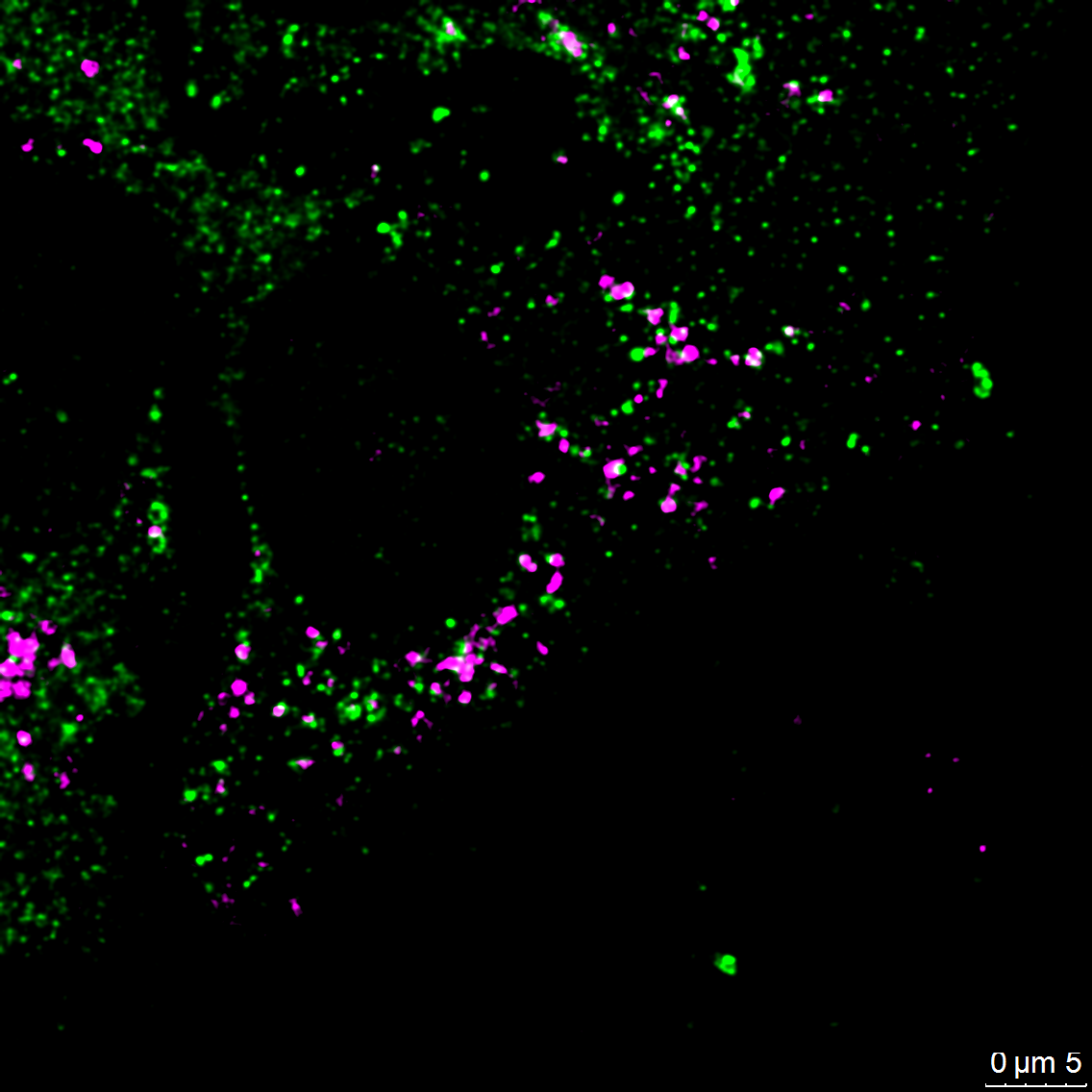

Supplement: Supplementary file 8 — Source data Fig. 1 [file 44318_2025_654_MOESM8_ESM.zip › Figure 1/1D/AREL1+LAMP1 Merge.tif]

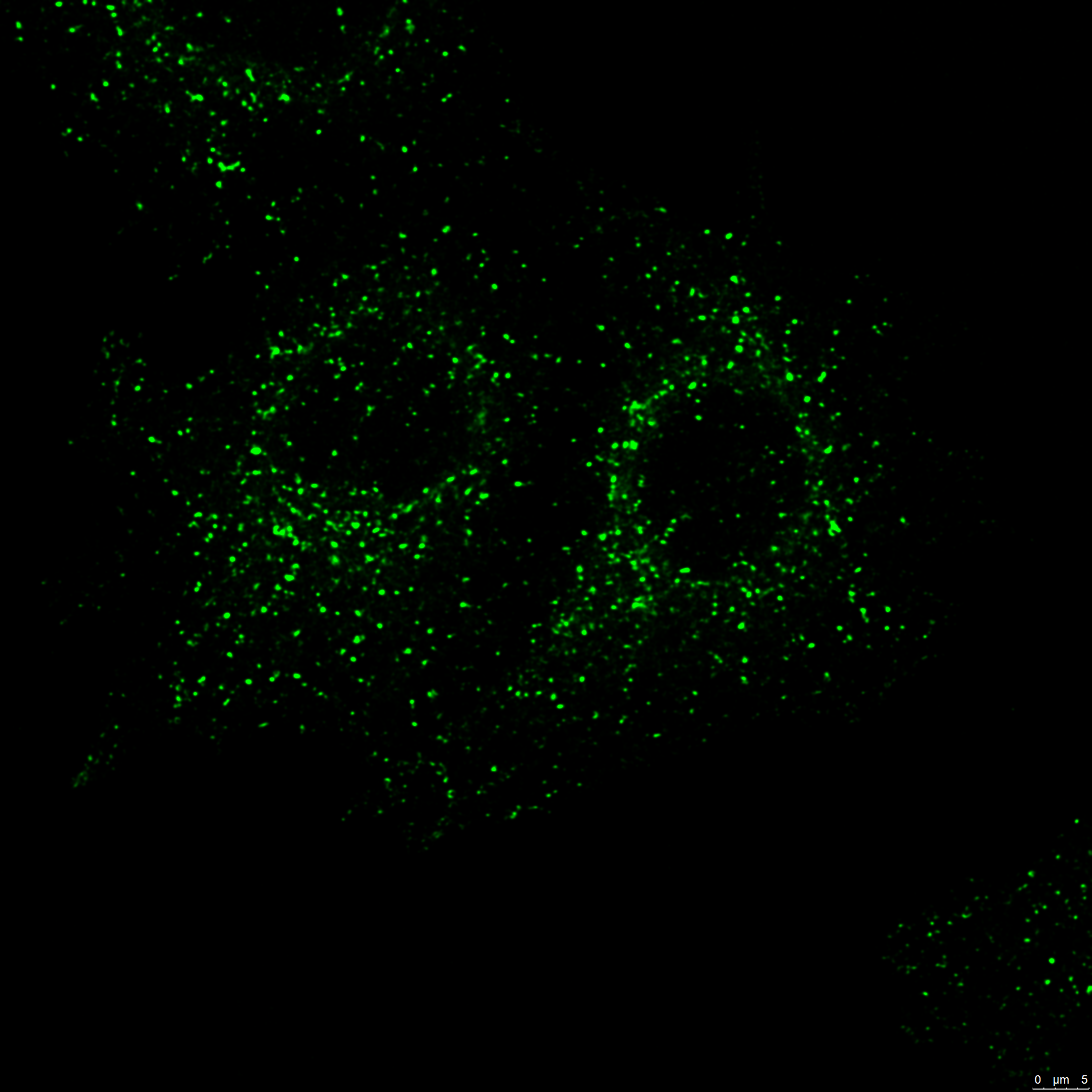

Supplement: Supplementary file 8 — Source data Fig. 1 [file 44318_2025_654_MOESM8_ESM.zip › Figure 1/1C/AREL1-EGFP.tif]

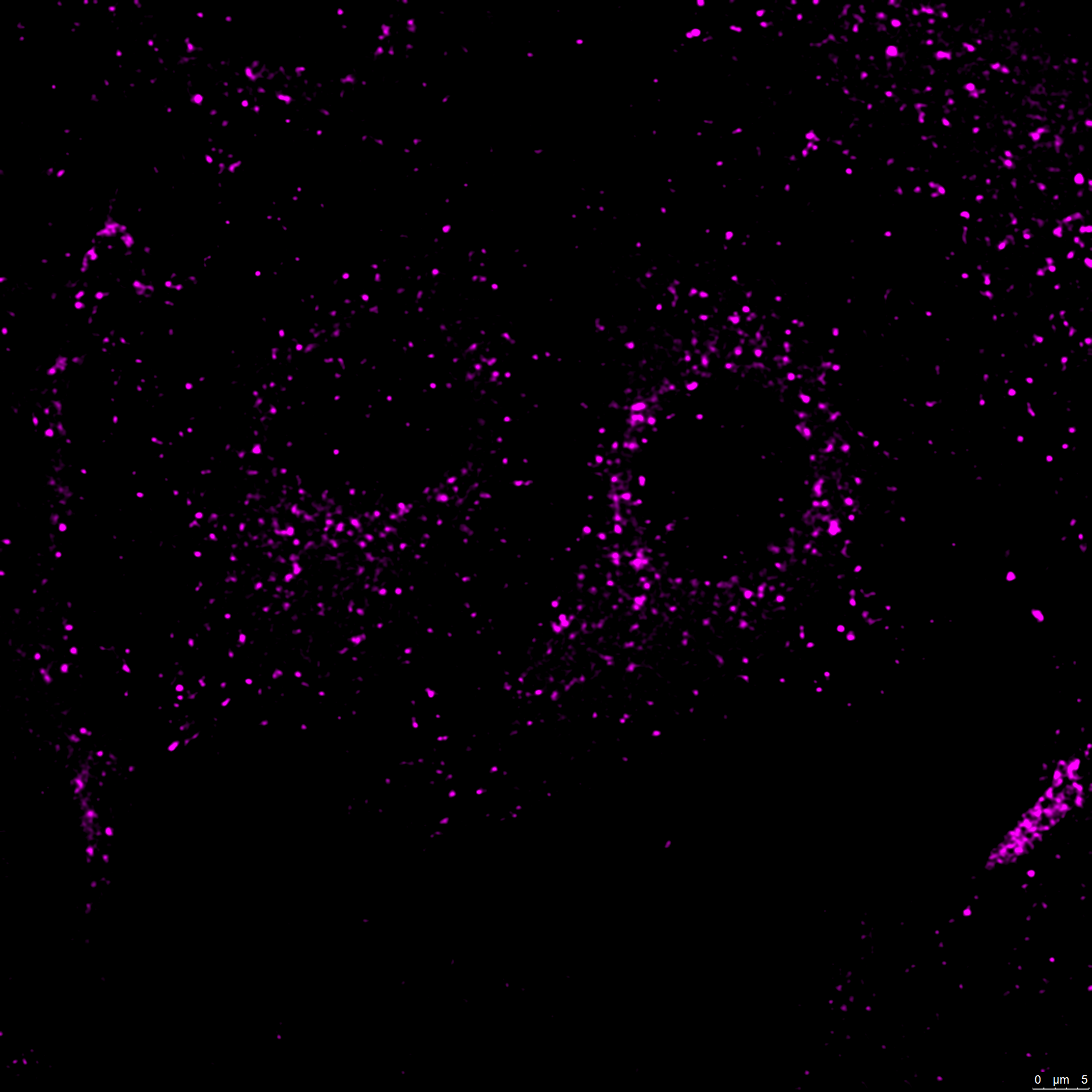

Supplement: Supplementary file 8 — Source data Fig. 1 [file 44318_2025_654_MOESM8_ESM.zip › Figure 1/1C/LAMP1.tif]

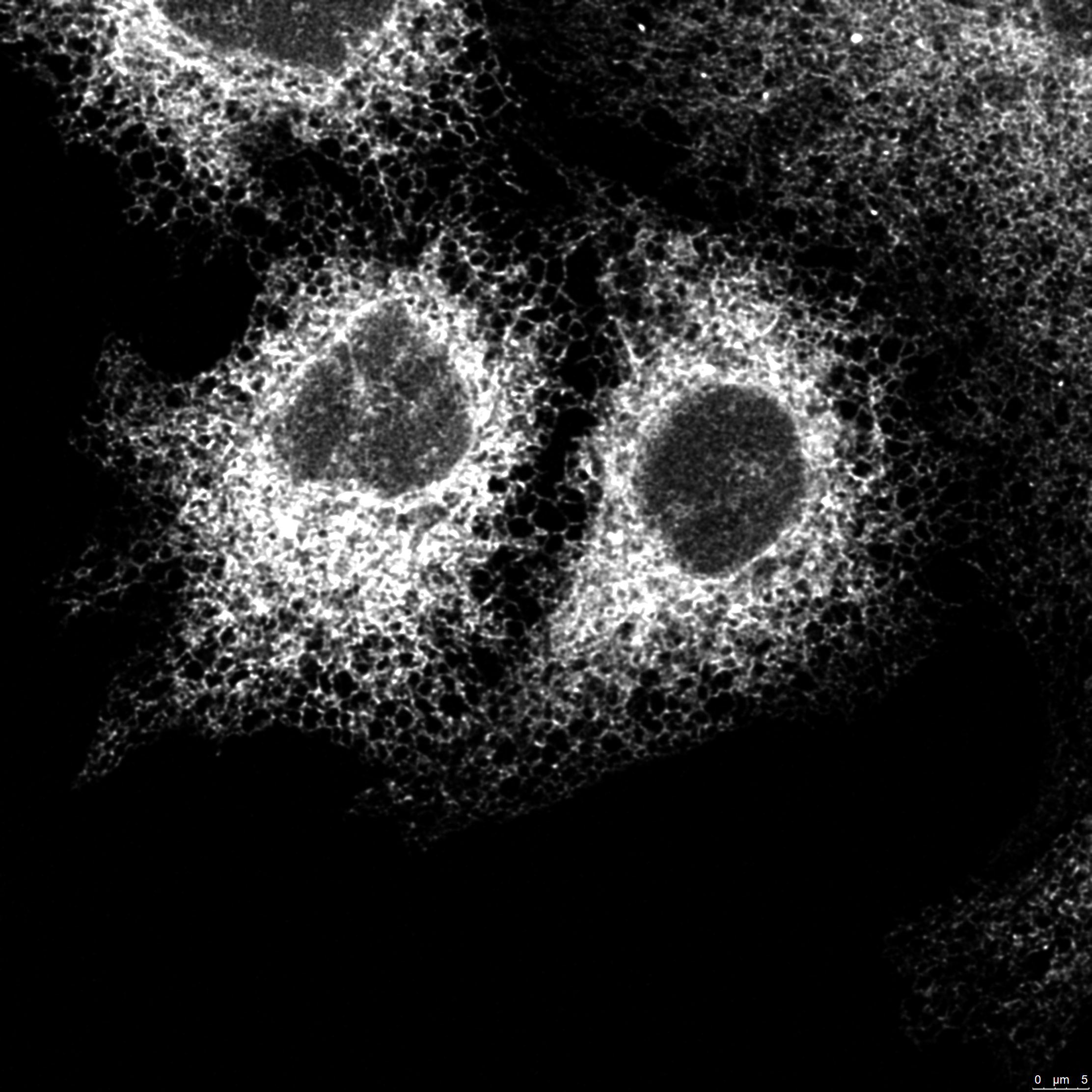

Supplement: Supplementary file 8 — Source data Fig. 1 [file 44318_2025_654_MOESM8_ESM.zip › Figure 1/1C/mCherry-Sec61╬▓.tif]

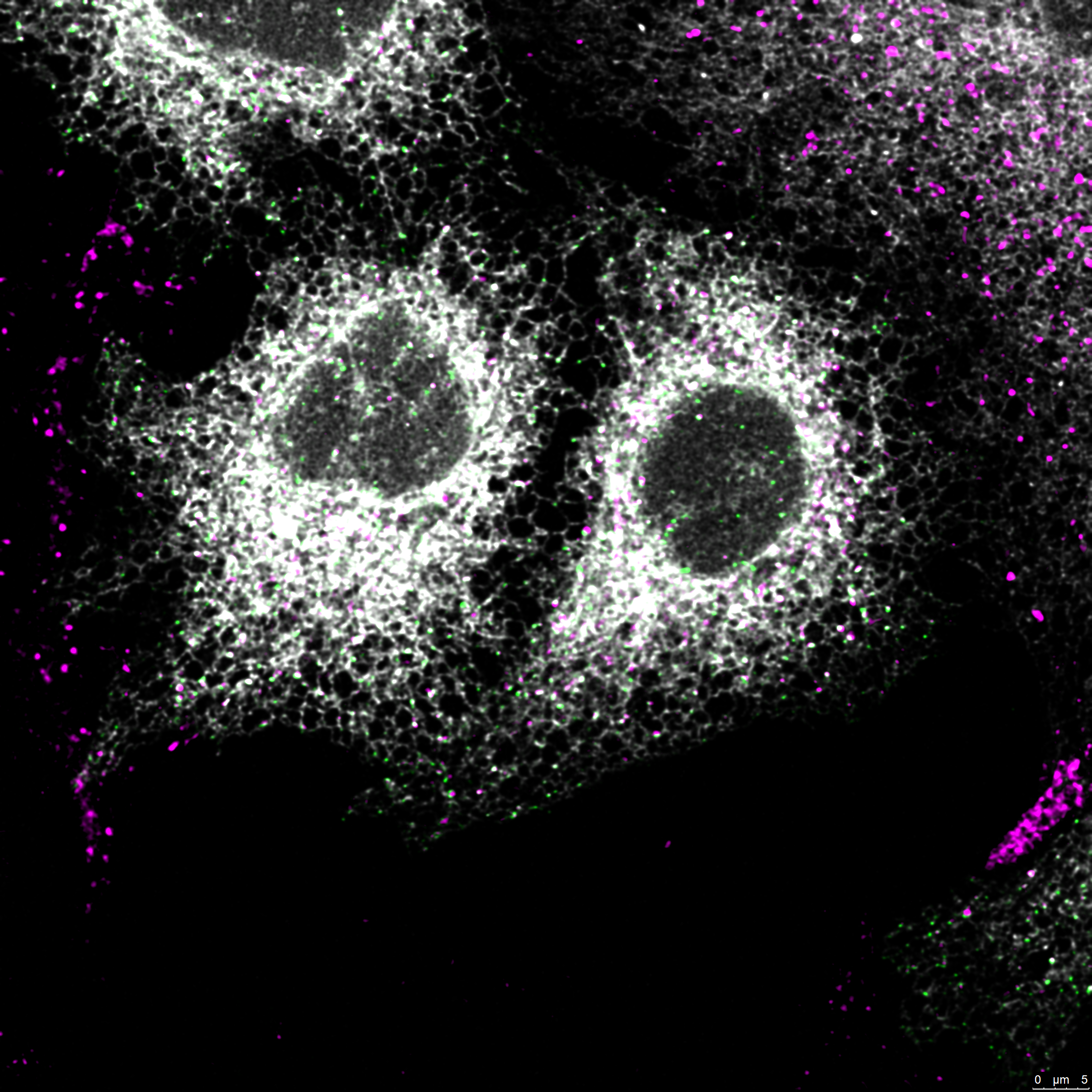

Supplement: Supplementary file 8 — Source data Fig. 1 [file 44318_2025_654_MOESM8_ESM.zip › Figure 1/1C/Merge.tif]

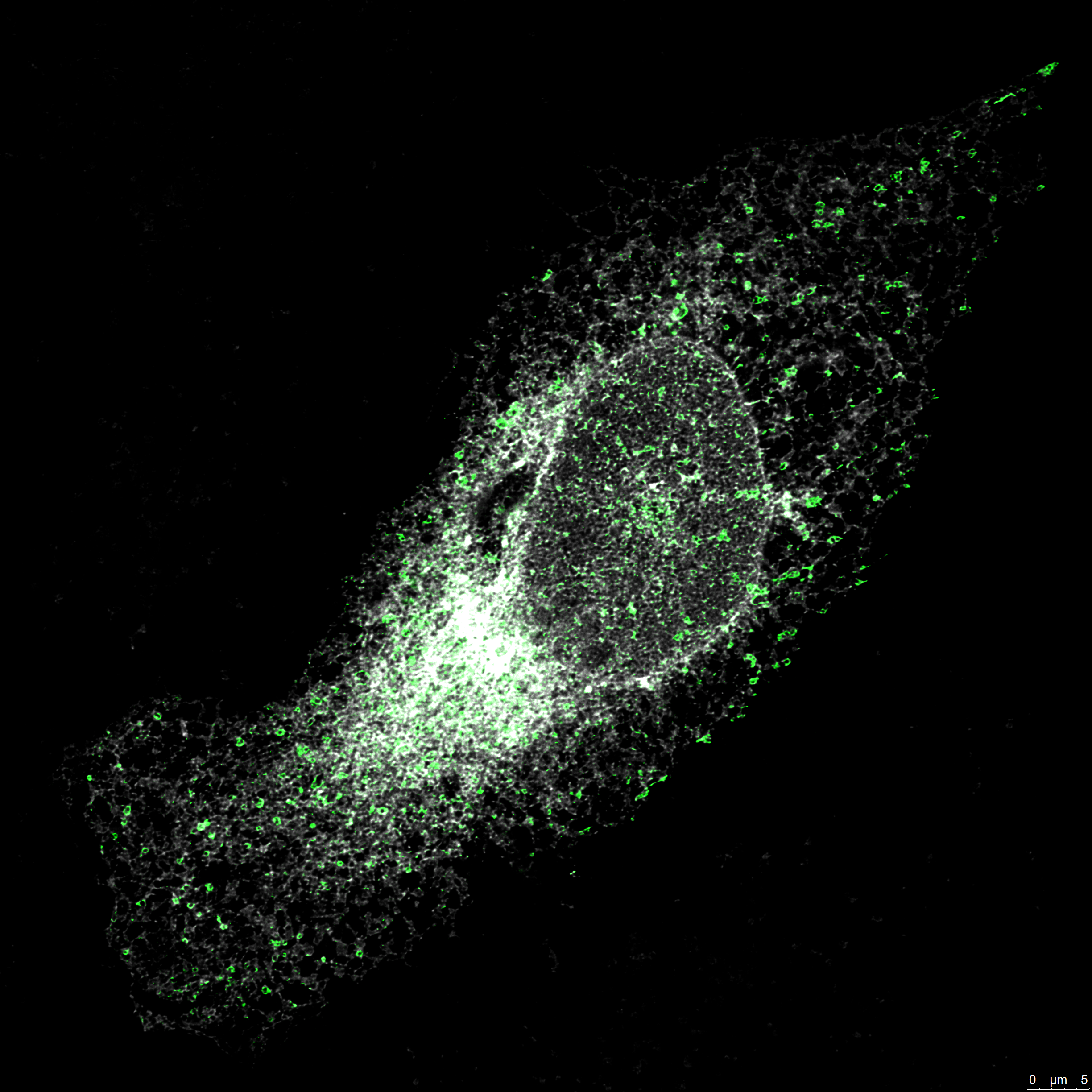

Supplement: Supplementary file 8 — Source data Fig. 1 [file 44318_2025_654_MOESM8_ESM.zip › Figure 1/1J/1J-1-WT-AREL1(WT)-EGFP+mCherry-Sec61╬▓ Merge.tif]

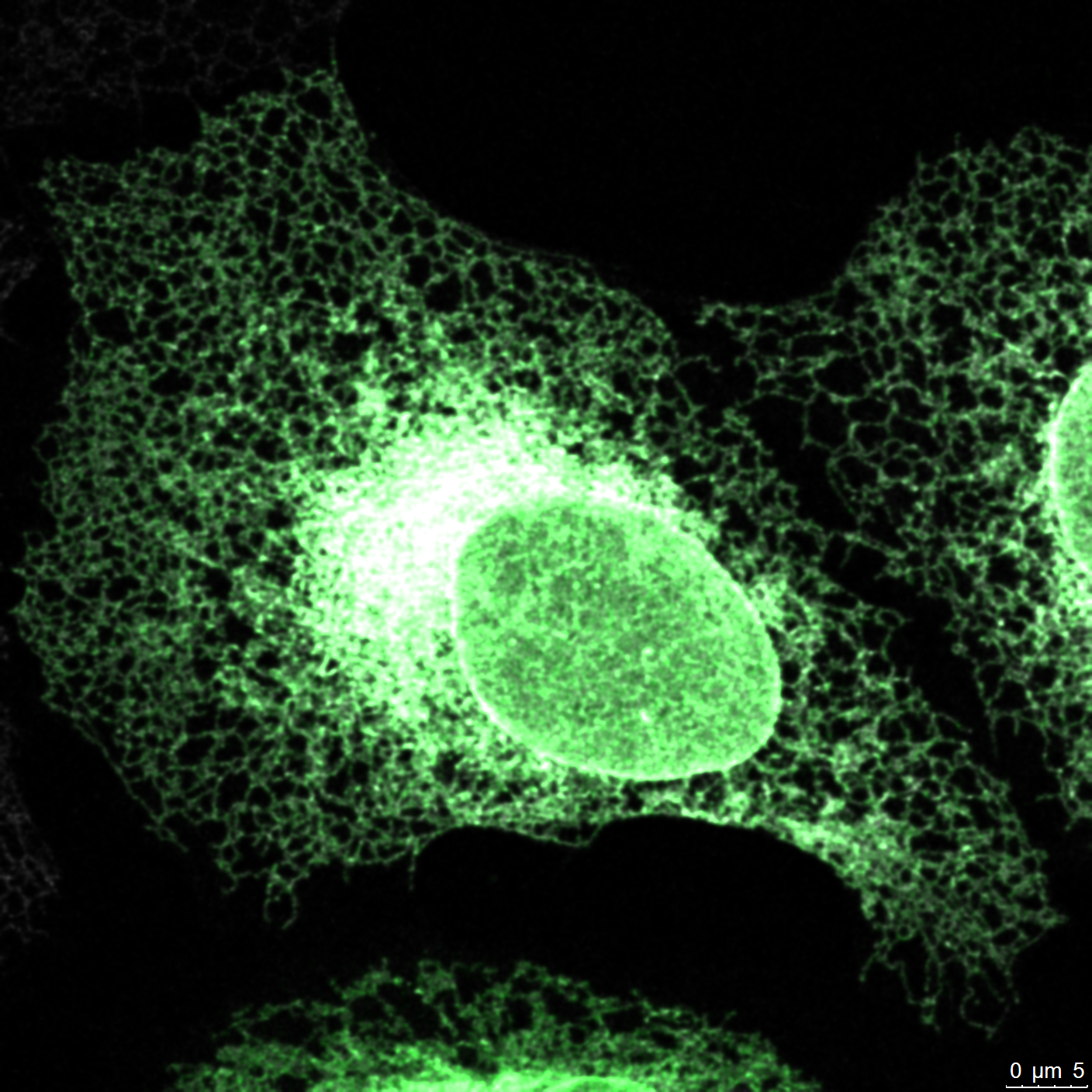

Supplement: Supplementary file 8 — Source data Fig. 1 [file 44318_2025_654_MOESM8_ESM.zip › Figure 1/1J/1J-4-╬ö5K-AREL1(╬ö5K)-EGFP+mCherry-Sec61╬▓ Merge.tif]

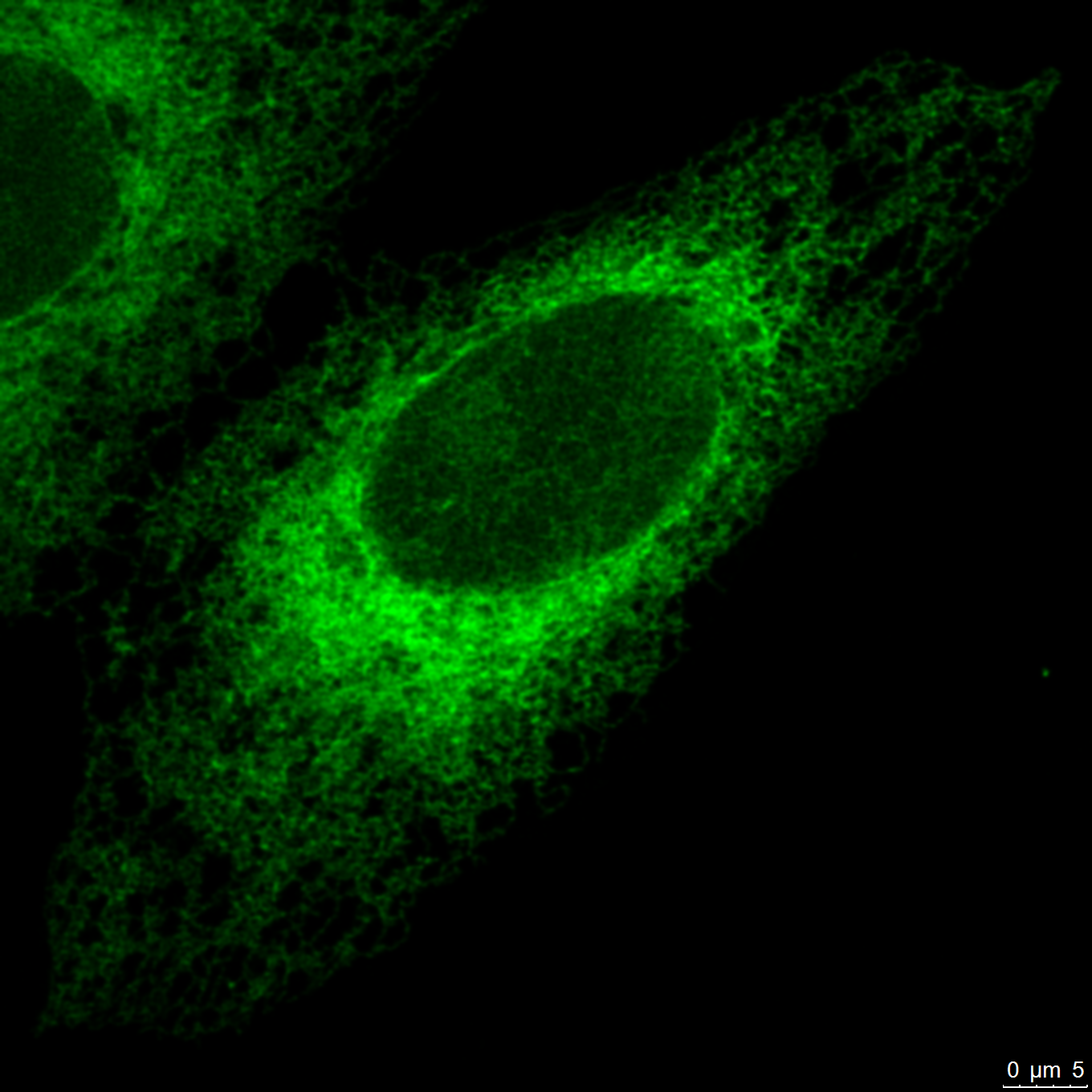

Supplement: Supplementary file 8 — Source data Fig. 1 [file 44318_2025_654_MOESM8_ESM.zip › Figure 1/1J/1J-3-╬öLCR-AREL1(╬öLCR)-EGFP.tif]

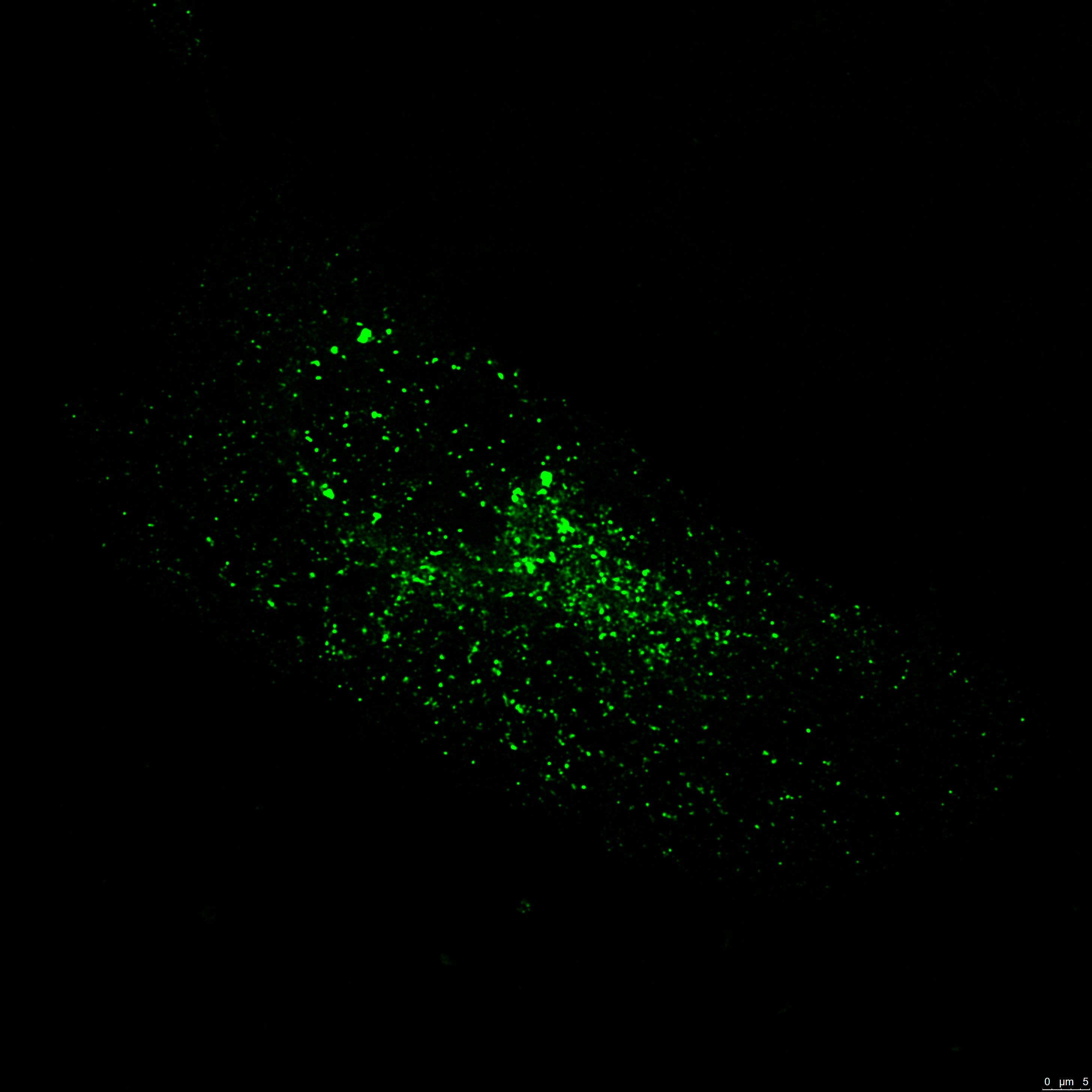

Supplement: Supplementary file 8 — Source data Fig. 1 [file 44318_2025_654_MOESM8_ESM.zip › Figure 1/1J/1J-6-Y354W+Y356W-AREL1(Y354W+Y356W)-EGFP.tif]

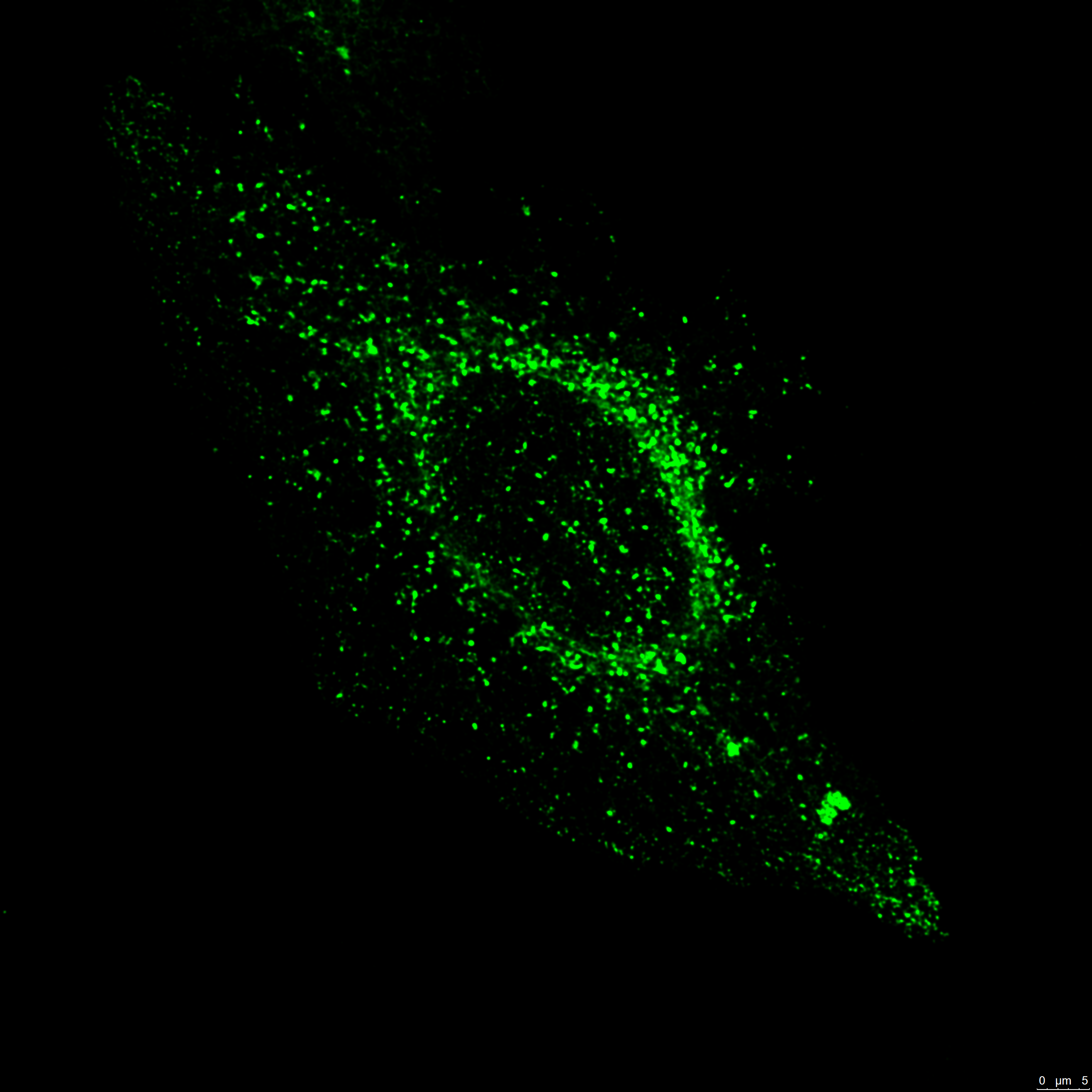

Supplement: Supplementary file 8 — Source data Fig. 1 [file 44318_2025_654_MOESM8_ESM.zip › Figure 1/1J/1J-2-╬öIDR-AREL1(╬öIDR)-EGFP.tif]

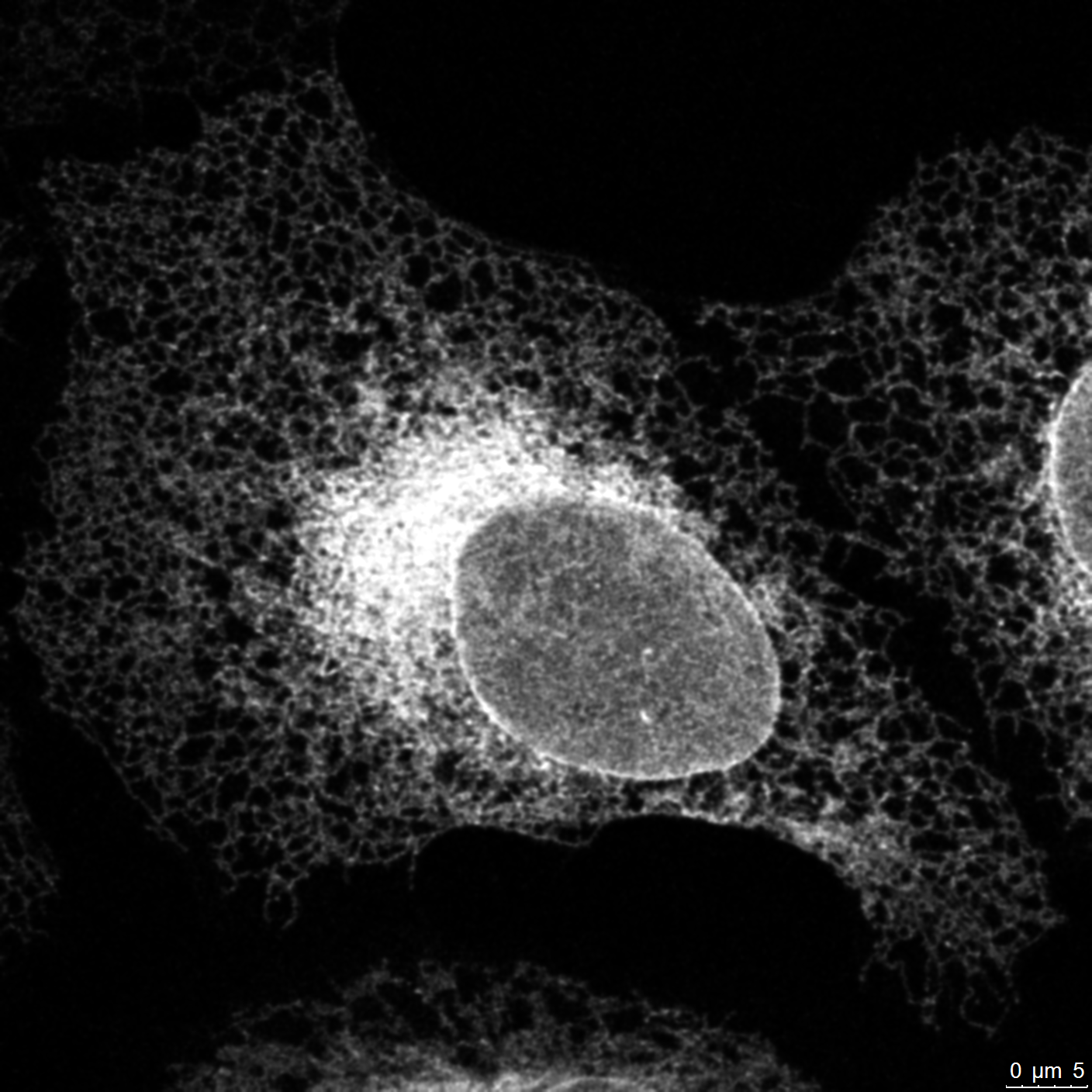

Supplement: Supplementary file 8 — Source data Fig. 1 [file 44318_2025_654_MOESM8_ESM.zip › Figure 1/1J/1J-4-╬ö5K-mCherry-Sec61╬▓.tif]

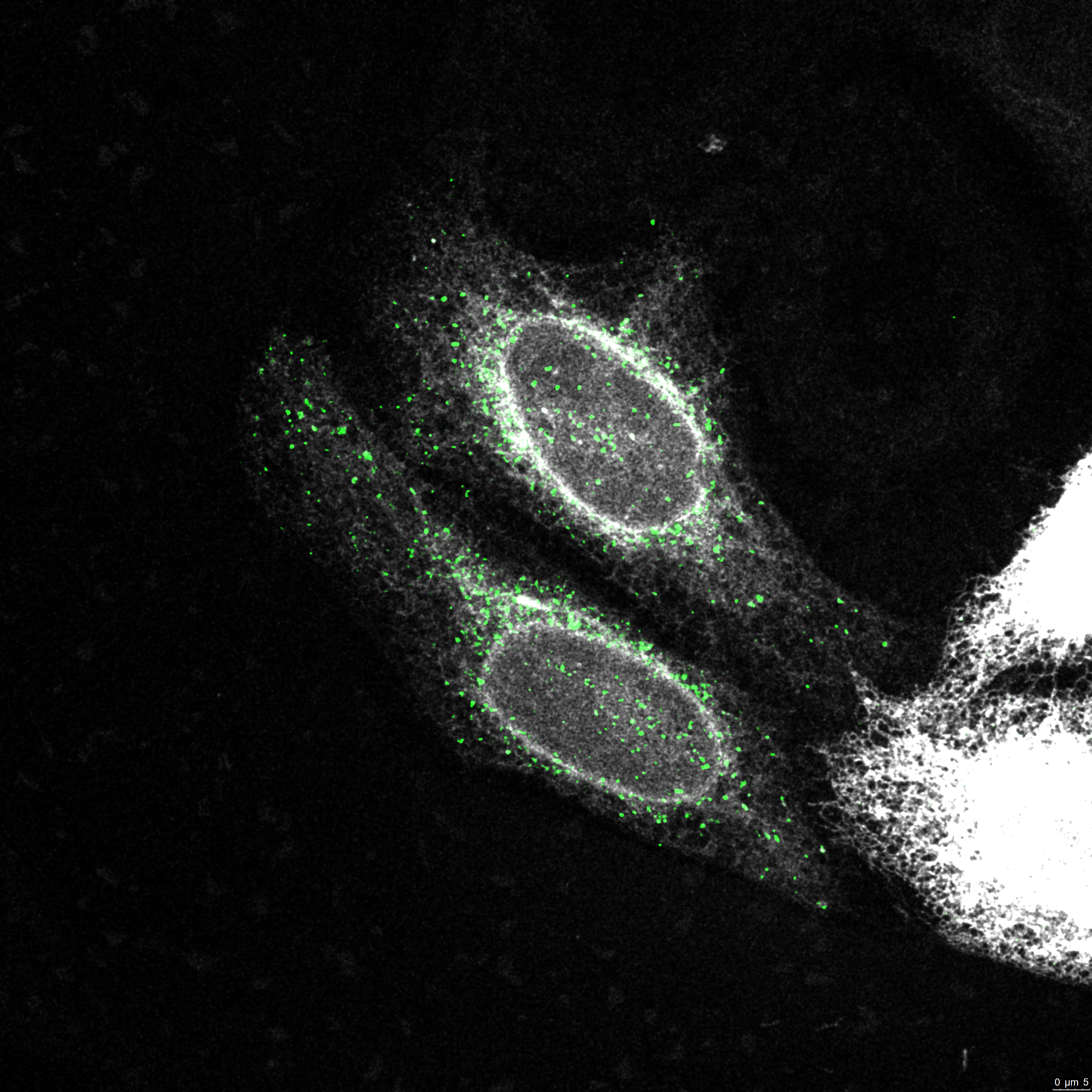

Supplement: Supplementary file 8 — Source data Fig. 1 [file 44318_2025_654_MOESM8_ESM.zip › Figure 1/1J/1J-7-Y354F+Y356F-AREL1(Y354F+Y356F)-EGFP+mCherry-Sec61╬▓ Merge.tif]

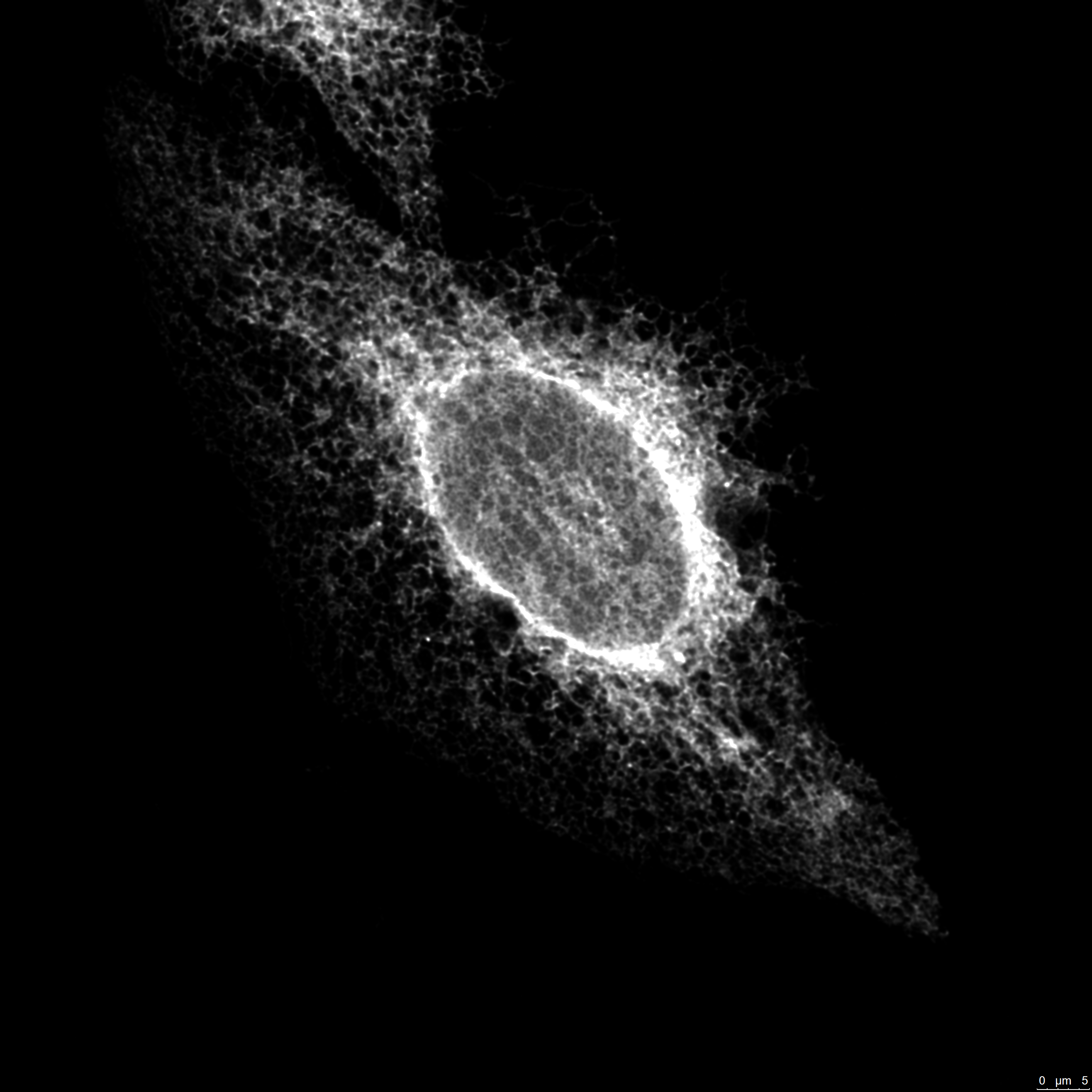

Supplement: Supplementary file 8 — Source data Fig. 1 [file 44318_2025_654_MOESM8_ESM.zip › Figure 1/1J/1J-2-╬öIDR-mCherry-Sec61╬▓.tif]

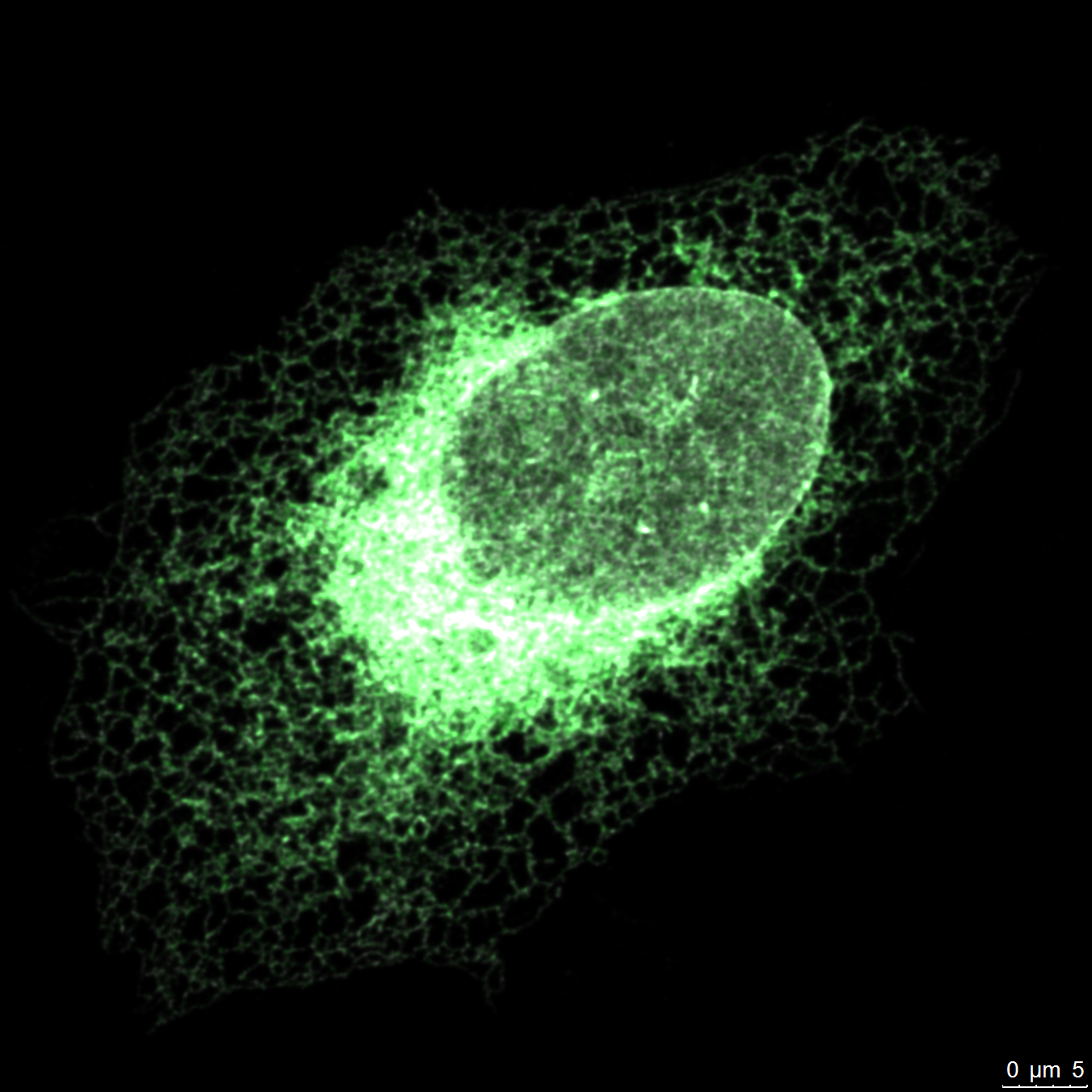

Supplement: Supplementary file 8 — Source data Fig. 1 [file 44318_2025_654_MOESM8_ESM.zip › Figure 1/1J/1J-5-Y354A+Y356A-AREL1(Y354A+Y356A)-EGFP+mCherry-Sec61╬▓ Merge.tif]

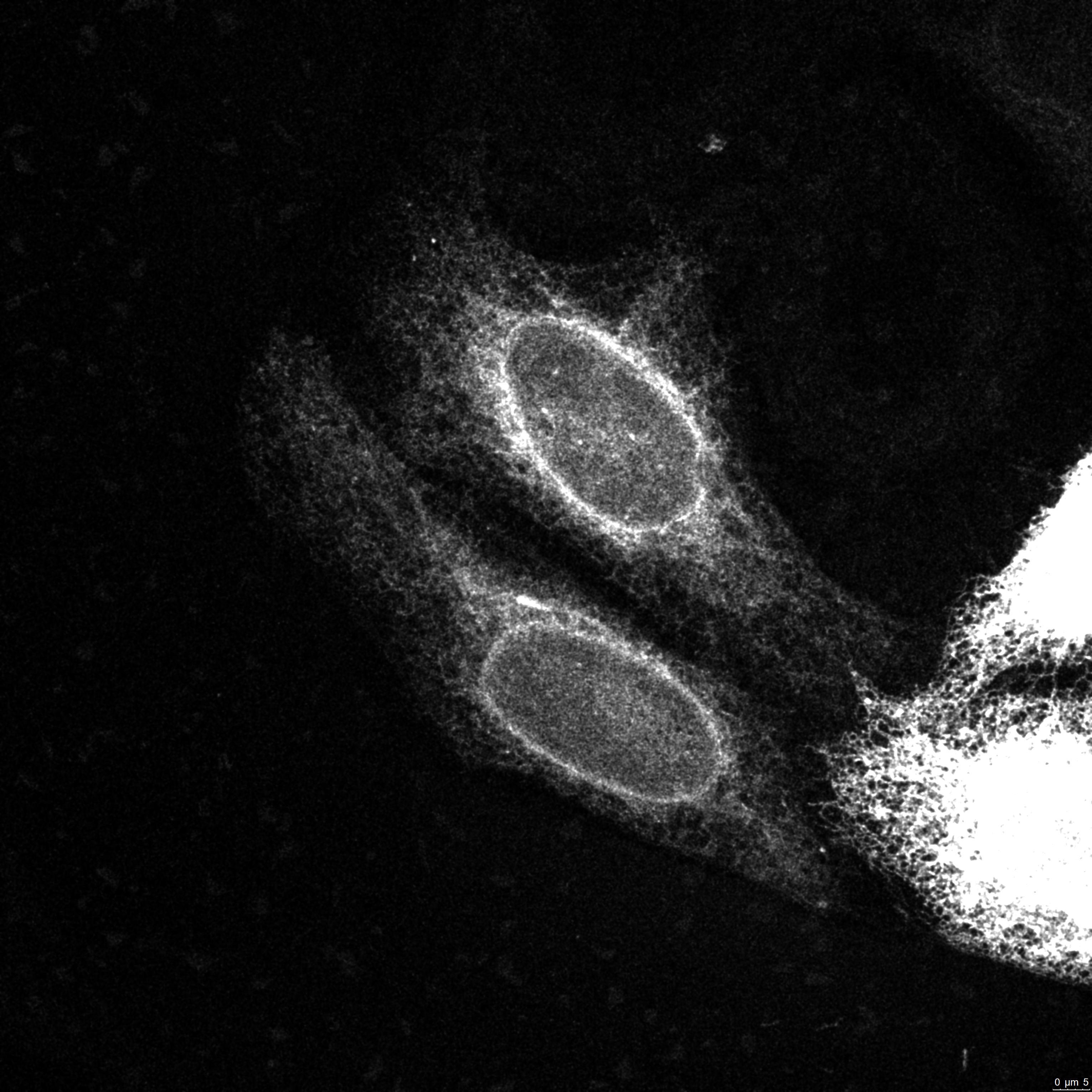

Supplement: Supplementary file 8 — Source data Fig. 1 [file 44318_2025_654_MOESM8_ESM.zip › Figure 1/1J/1J-7-mCherry-Sec61╬▓.tif]

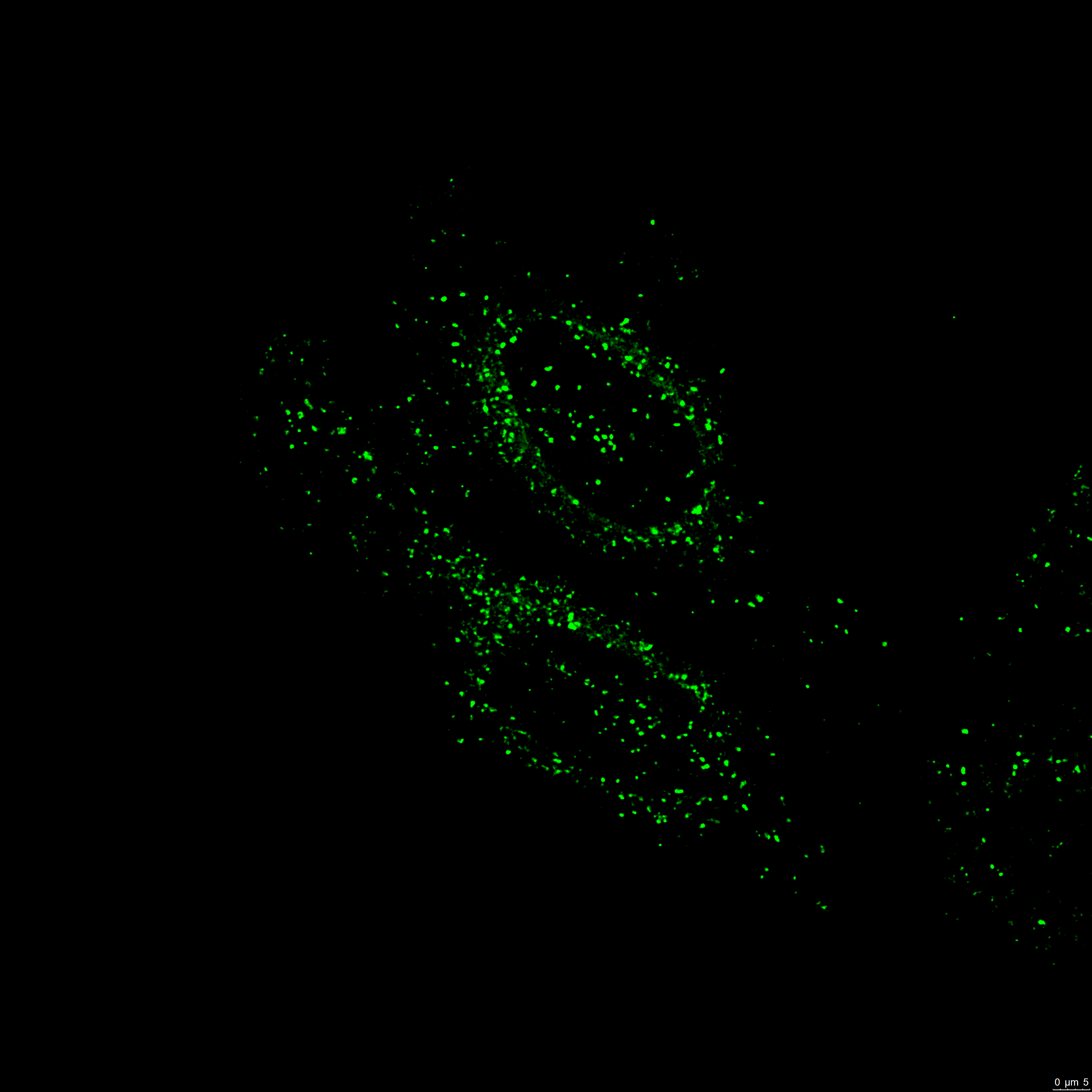

Supplement: Supplementary file 8 — Source data Fig. 1 [file 44318_2025_654_MOESM8_ESM.zip › Figure 1/1J/1J-7-Y354F+Y356F-AREL1(Y354F+Y356F)-EGFP.tif]

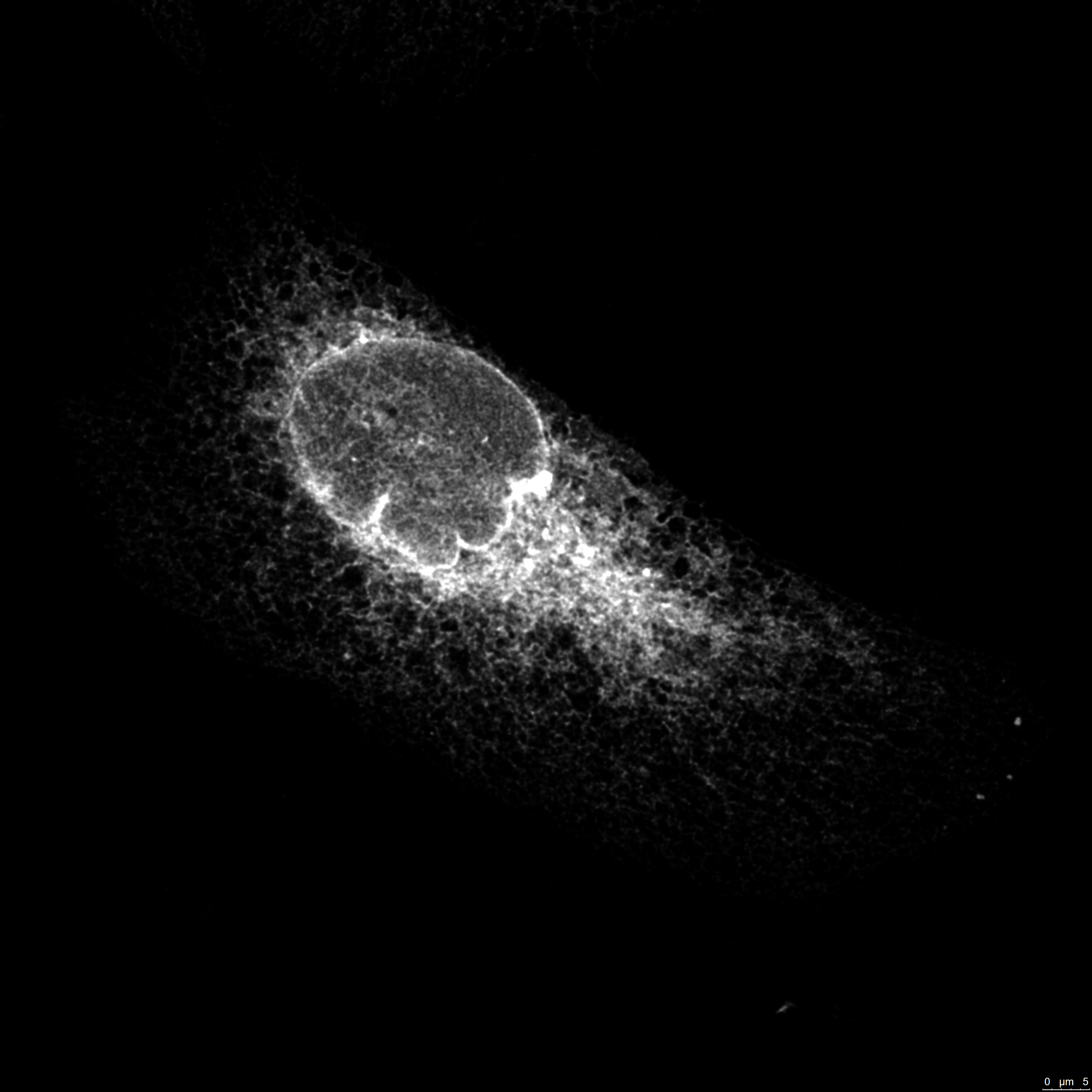

Supplement: Supplementary file 8 — Source data Fig. 1 [file 44318_2025_654_MOESM8_ESM.zip › Figure 1/1J/1J-6-mCherry-Sec61╬▓.tif]

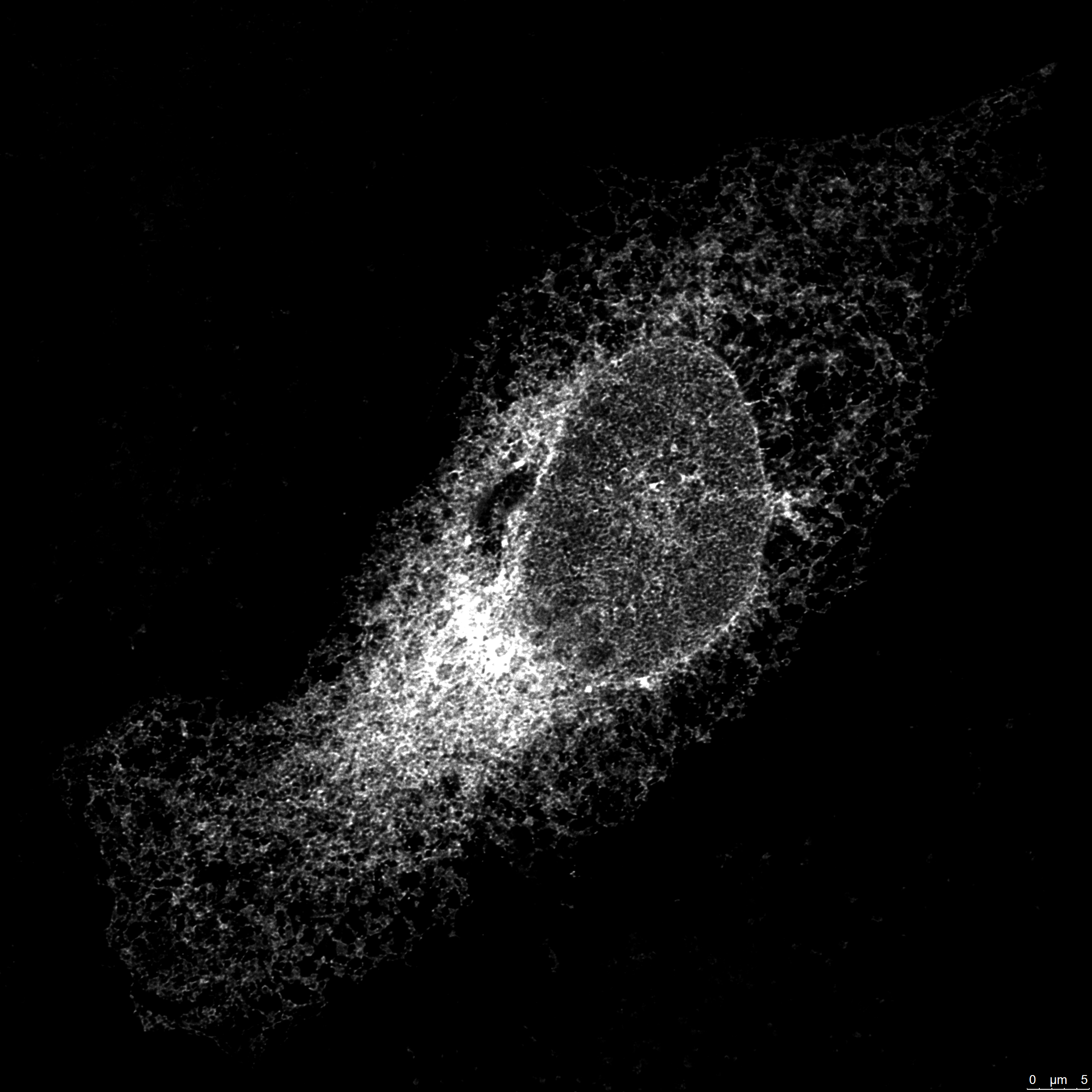

Supplement: Supplementary file 8 — Source data Fig. 1 [file 44318_2025_654_MOESM8_ESM.zip › Figure 1/1J/1J-1-WT-mCherry-Sec61╬▓.tif]

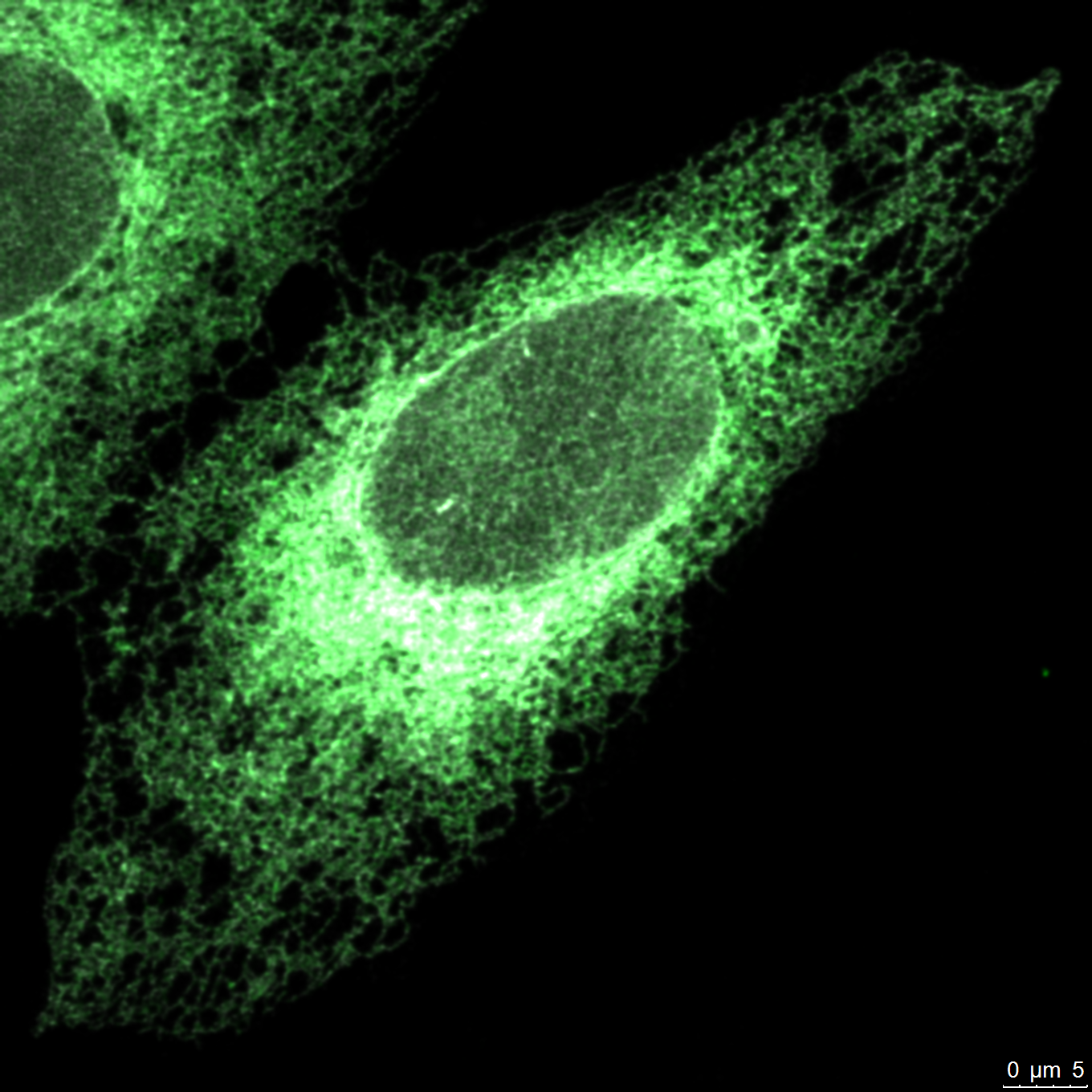

Supplement: Supplementary file 8 — Source data Fig. 1 [file 44318_2025_654_MOESM8_ESM.zip › Figure 1/1J/1J-3-╬öLCR-AREL1(╬öLCR)-EGFP+mCherry-Sec61╬▓ Merge.tif]

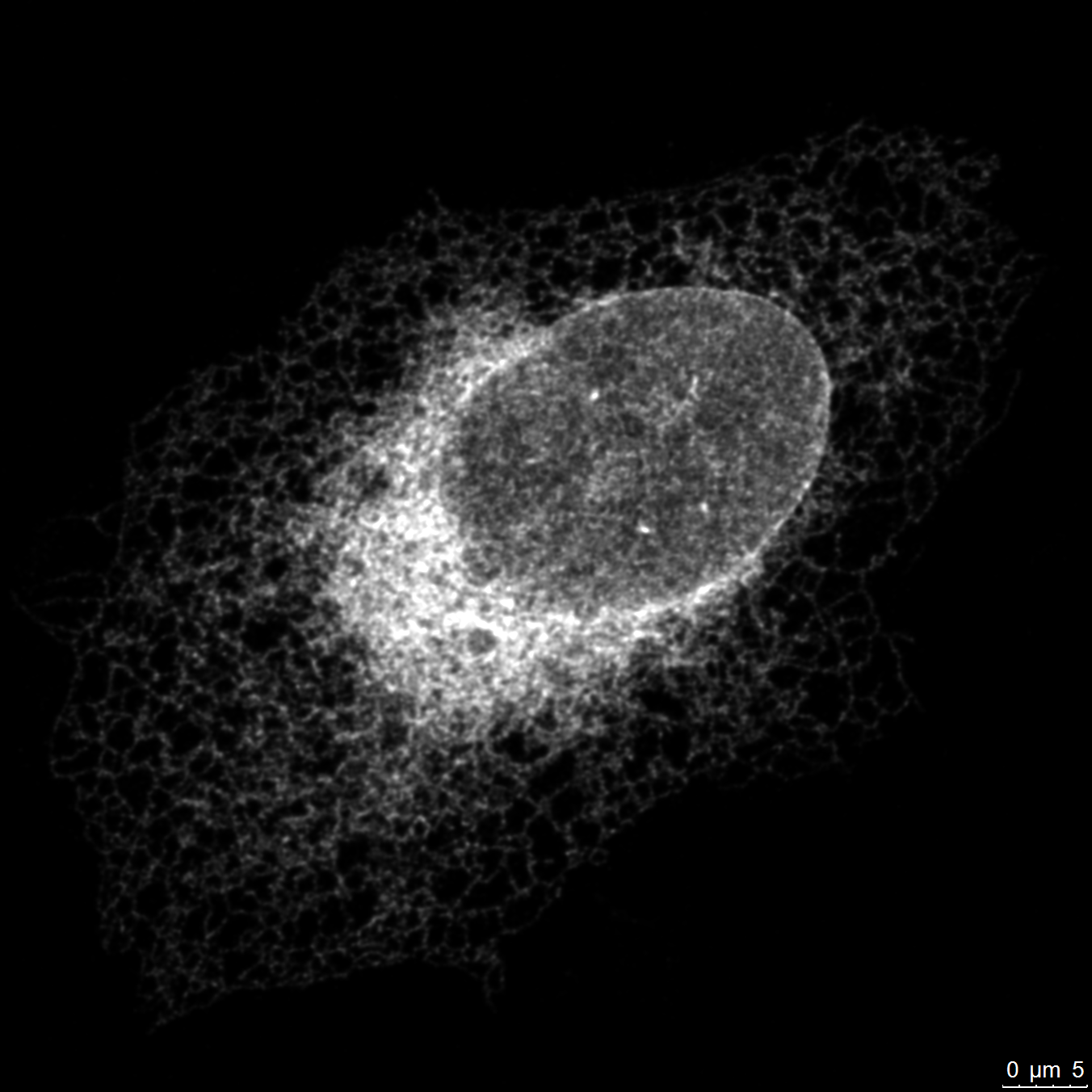

Supplement: Supplementary file 8 — Source data Fig. 1 [file 44318_2025_654_MOESM8_ESM.zip › Figure 1/1J/1J-5-mCherry-Sec61╬▓.tif]

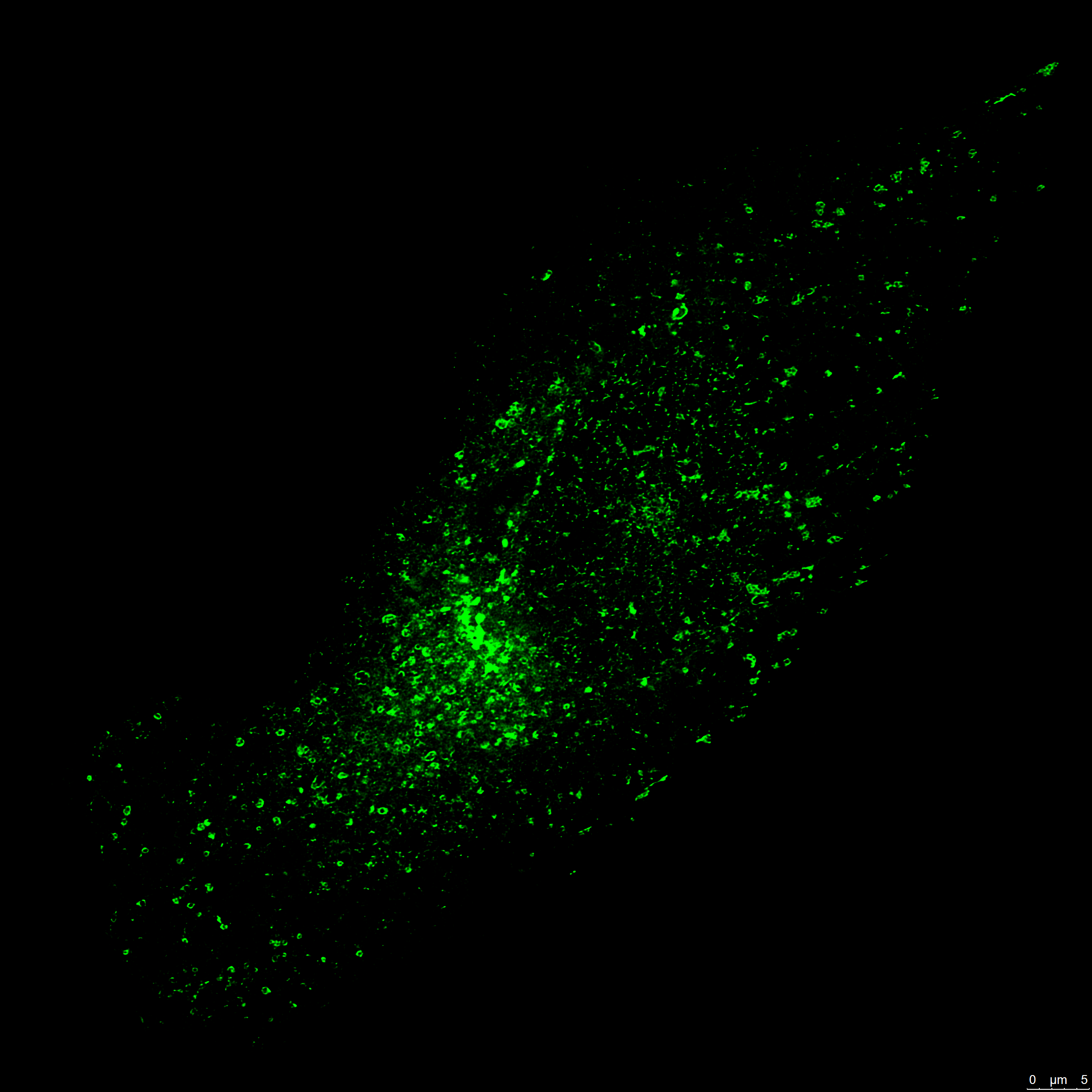

Supplement: Supplementary file 8 — Source data Fig. 1 [file 44318_2025_654_MOESM8_ESM.zip › Figure 1/1J/1J-1-WT-AREL1(WT)-EGFP.tif]

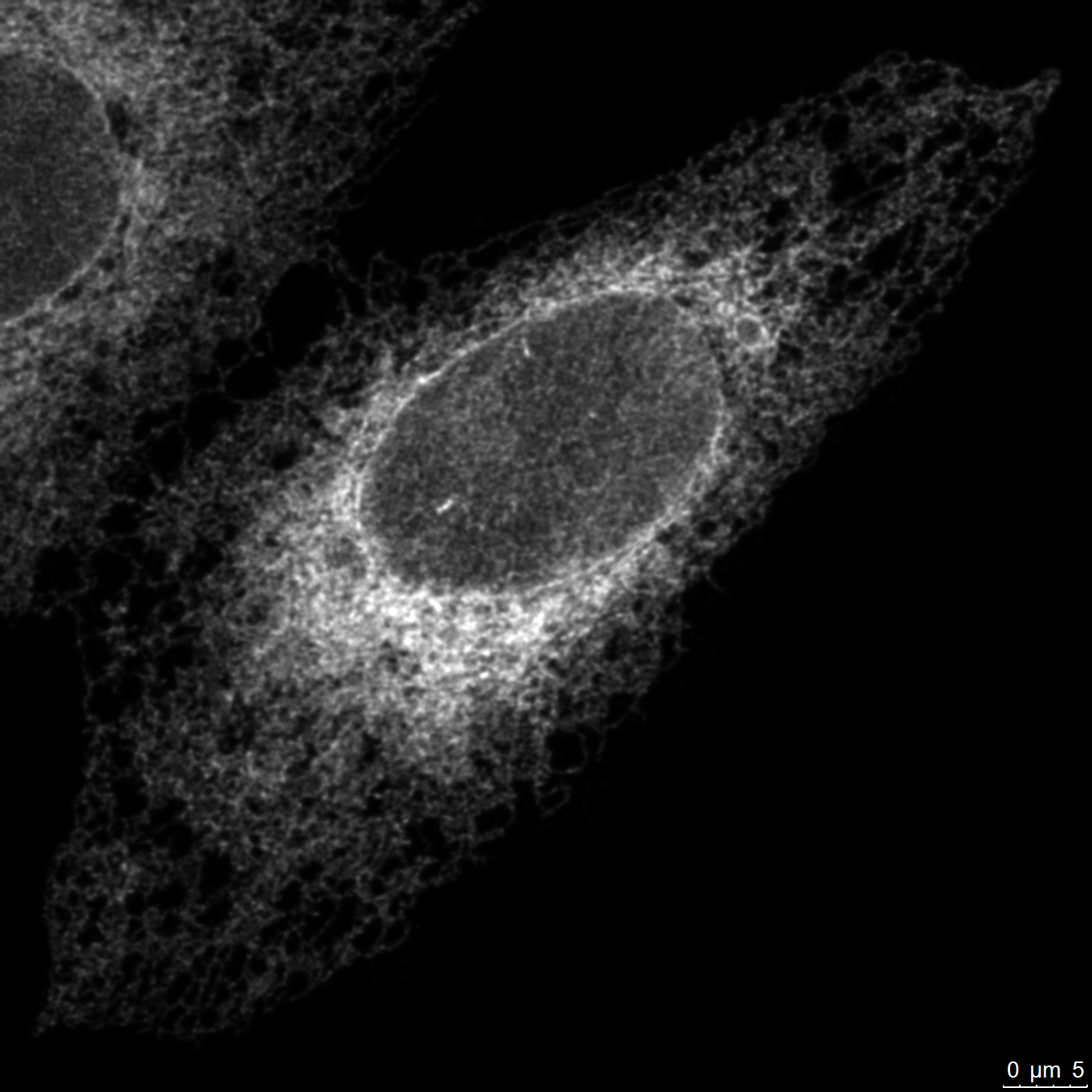

Supplement: Supplementary file 8 — Source data Fig. 1 [file 44318_2025_654_MOESM8_ESM.zip › Figure 1/1J/1J-3-╬öLCR-mCherry-Sec61╬▓.tif]

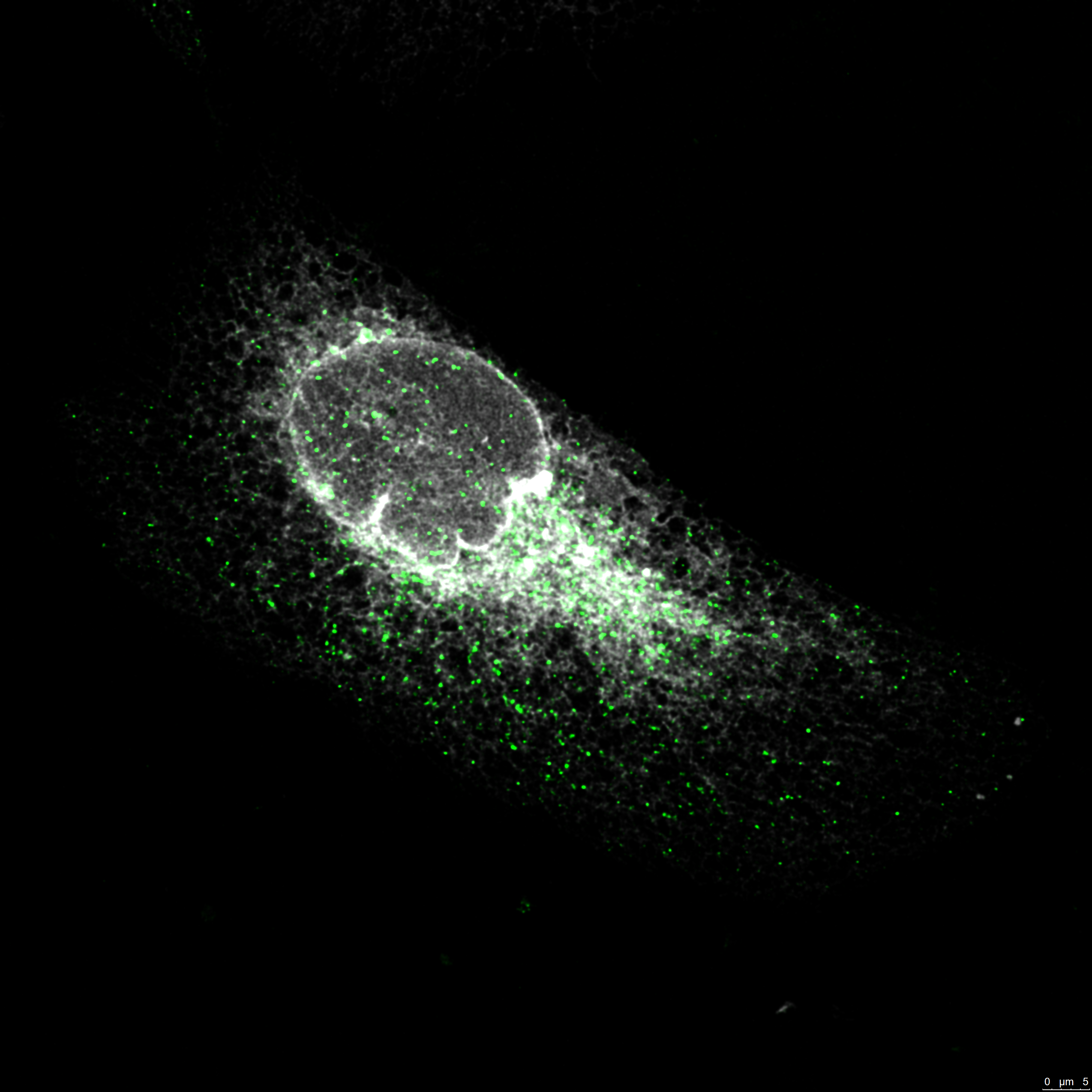

Supplement: Supplementary file 8 — Source data Fig. 1 [file 44318_2025_654_MOESM8_ESM.zip › Figure 1/1J/1J-6-Y354W+Y356W-AREL1(Y354W+Y356W)-EGFP+mCherry-Sec61╬▓ Merge.tif]

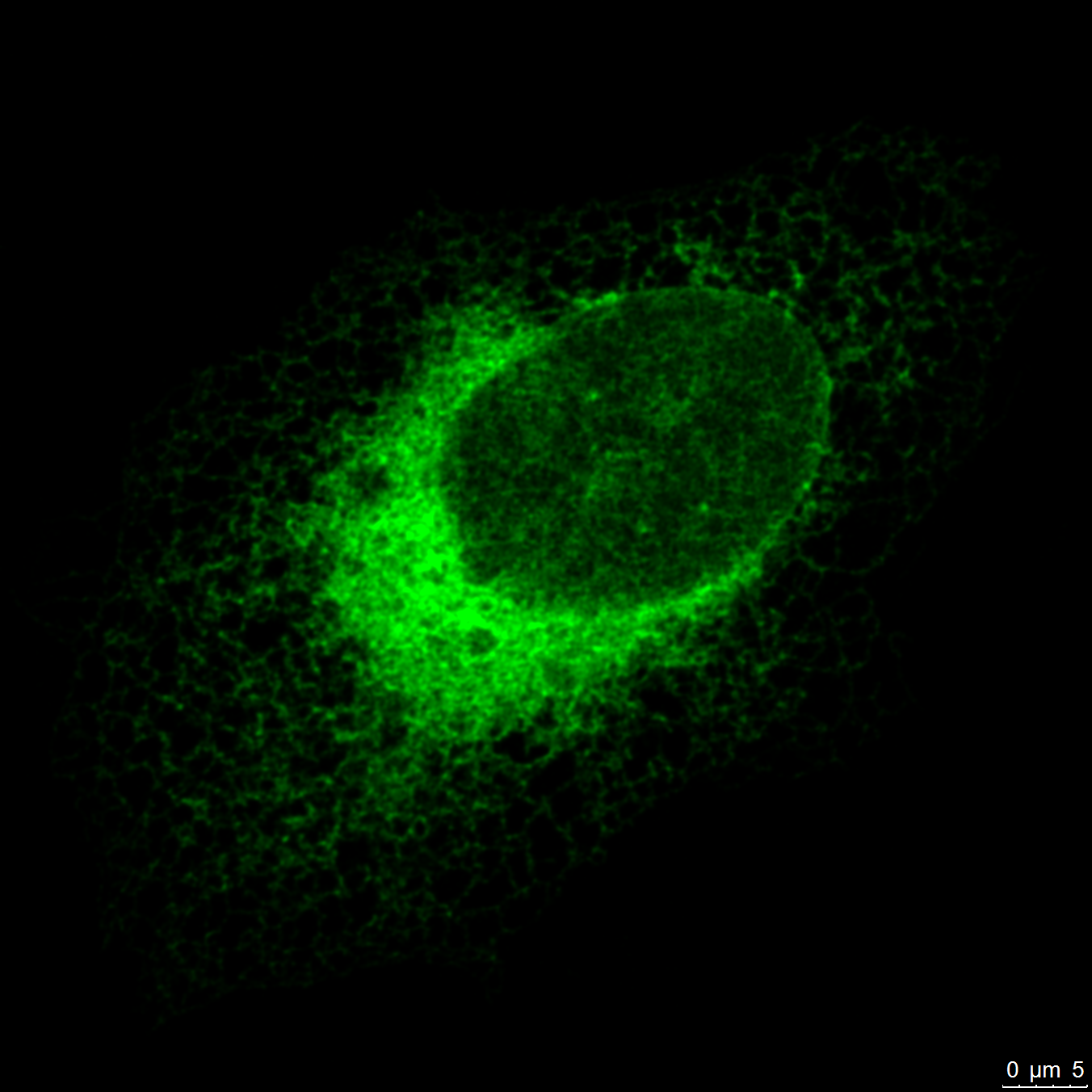

Supplement: Supplementary file 8 — Source data Fig. 1 [file 44318_2025_654_MOESM8_ESM.zip › Figure 1/1J/1J-5-Y354A+Y356A-AREL1(Y354A+Y356A)-EGFP.tif]

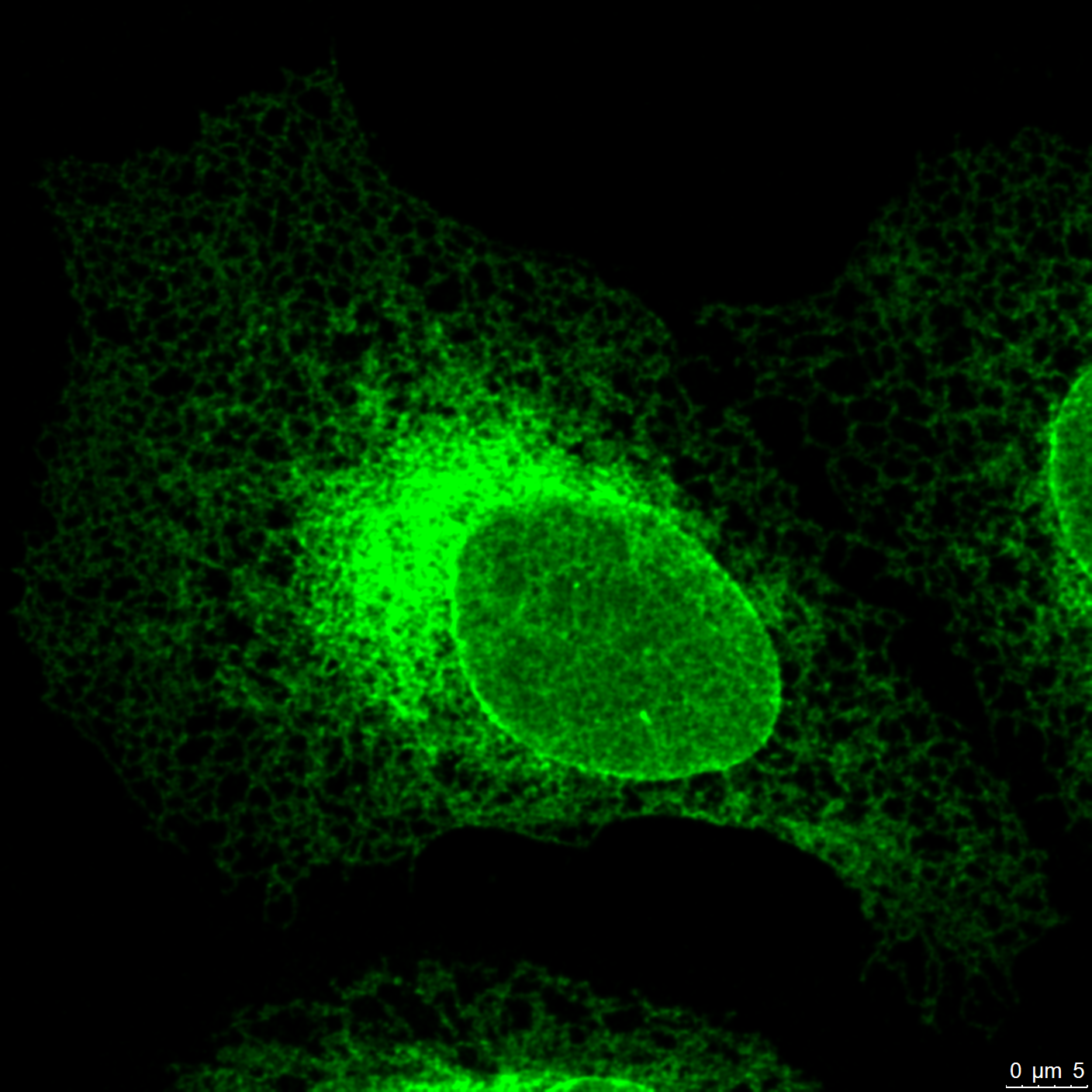

Supplement: Supplementary file 8 — Source data Fig. 1 [file 44318_2025_654_MOESM8_ESM.zip › Figure 1/1J/1J-4-╬ö5K-AREL1(╬ö5K)-EGFP.tif]

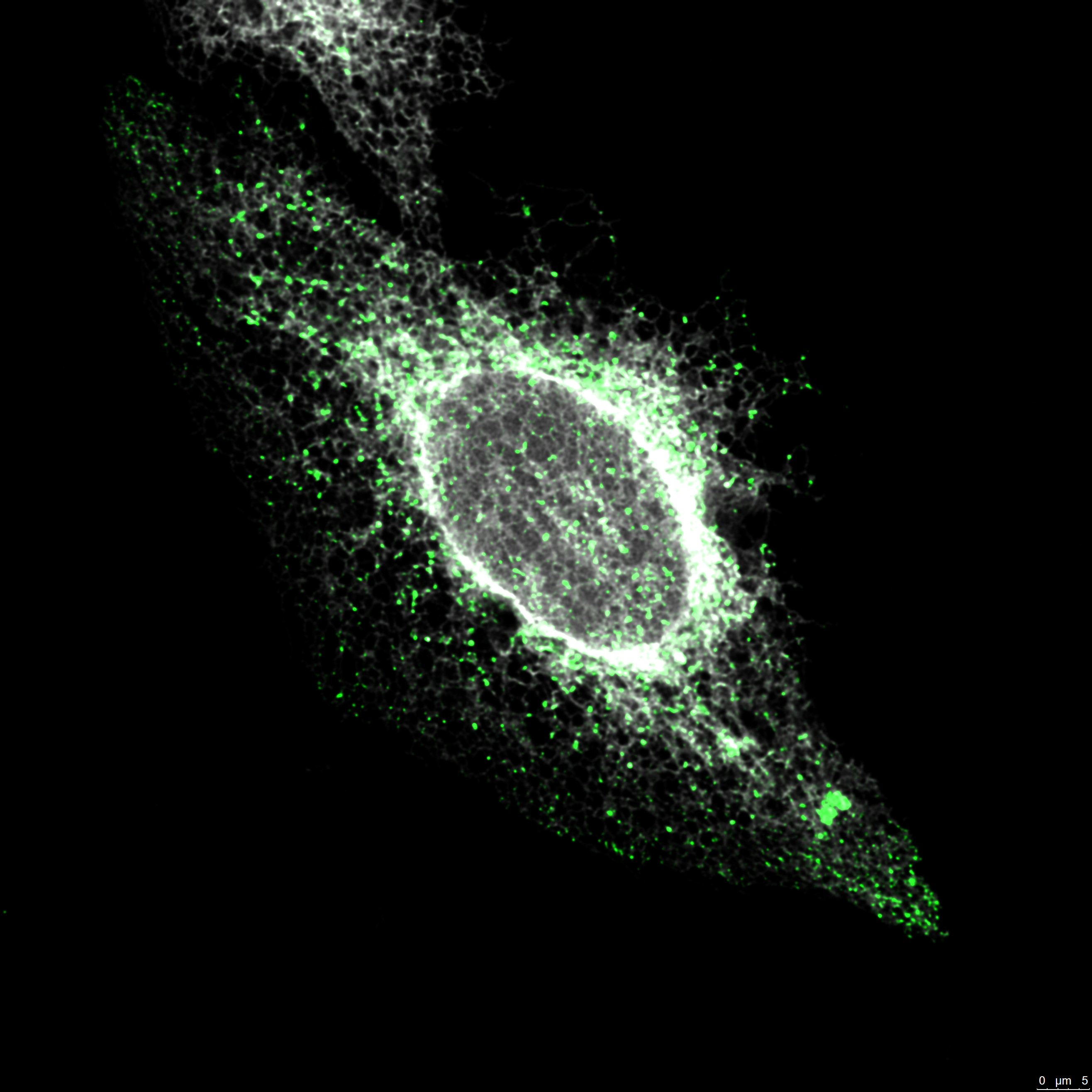

Supplement: Supplementary file 8 — Source data Fig. 1 [file 44318_2025_654_MOESM8_ESM.zip › Figure 1/1J/1J-2-╬öIDR-AREL1(╬öIDR)-EGFP+mCherry-Sec61╬▓ Merge.tif]

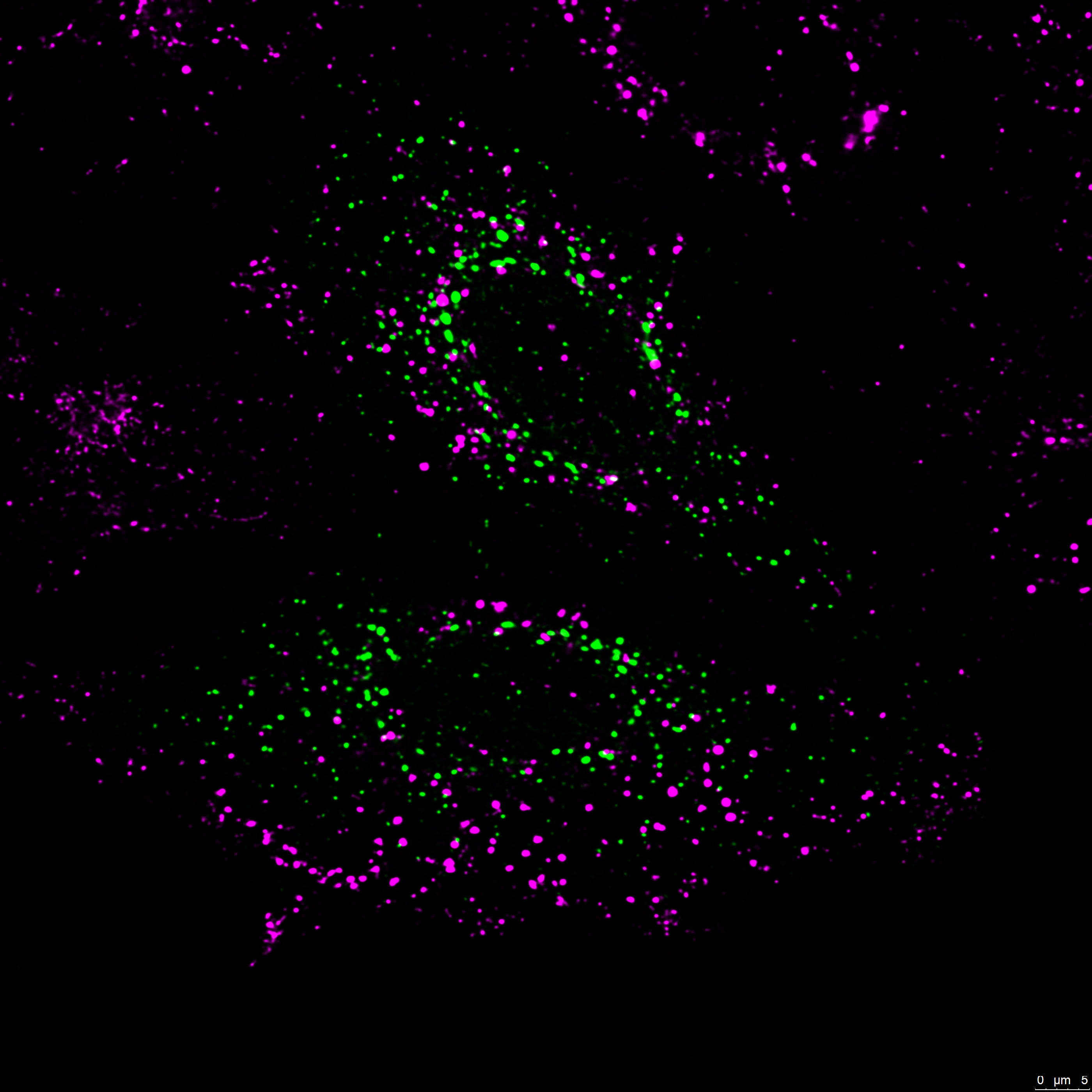

Supplement: Supplementary file 8 — Source data Fig. 1 [file 44318_2025_654_MOESM8_ESM.zip › Figure 1/1M/1M-shATP6V0A3.tif]

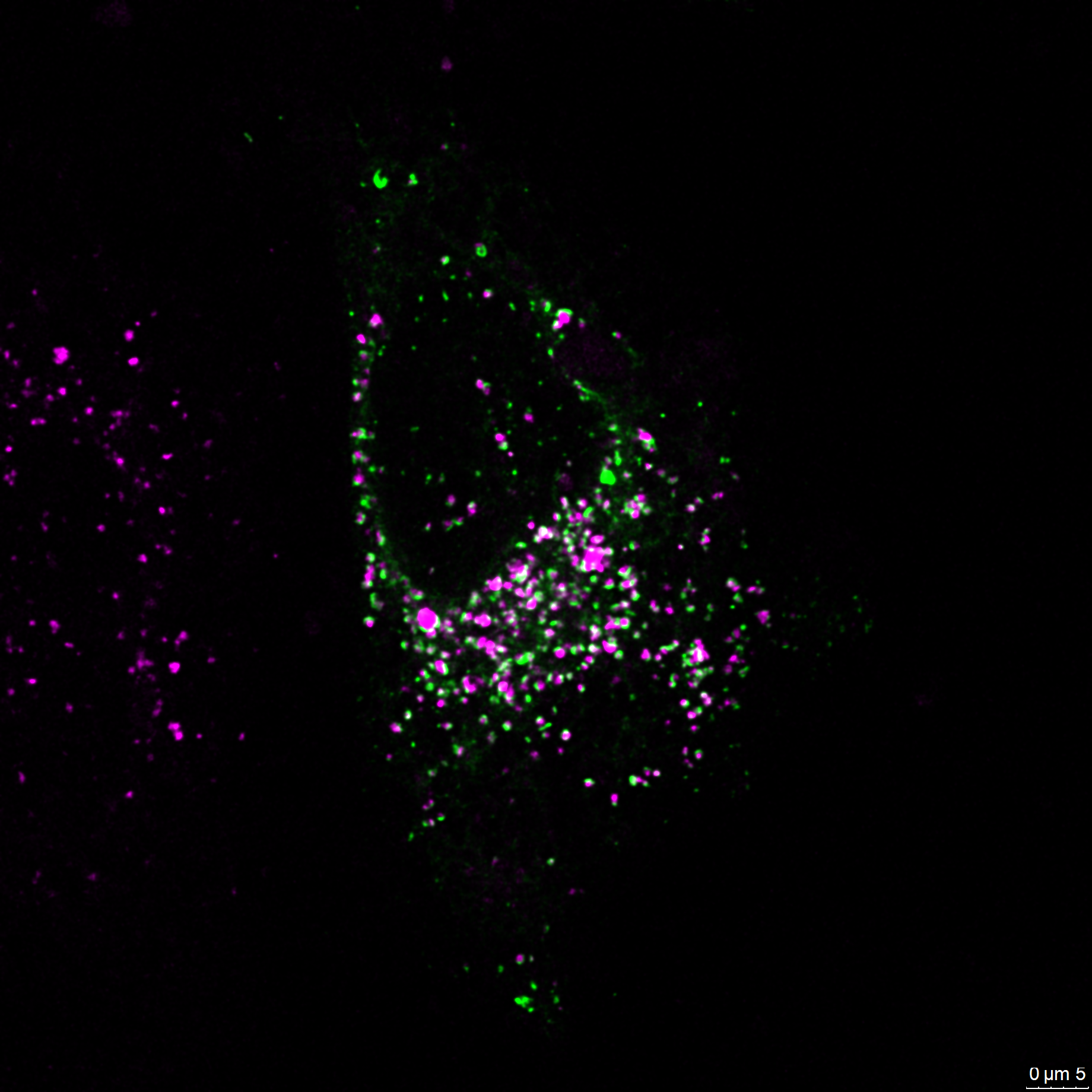

Supplement: Supplementary file 8 — Source data Fig. 1 [file 44318_2025_654_MOESM8_ESM.zip › Figure 1/1M/1M-shNC.tif]

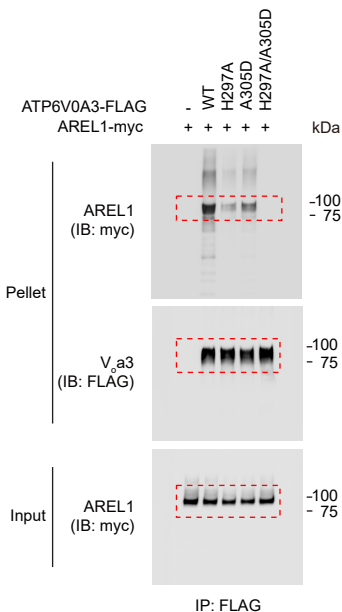

Supplement: Supplementary file 8 — Source data Fig. 1 [file 44318_2025_654_MOESM8_ESM.zip › Figure 1/1Q/1Q.pdf]

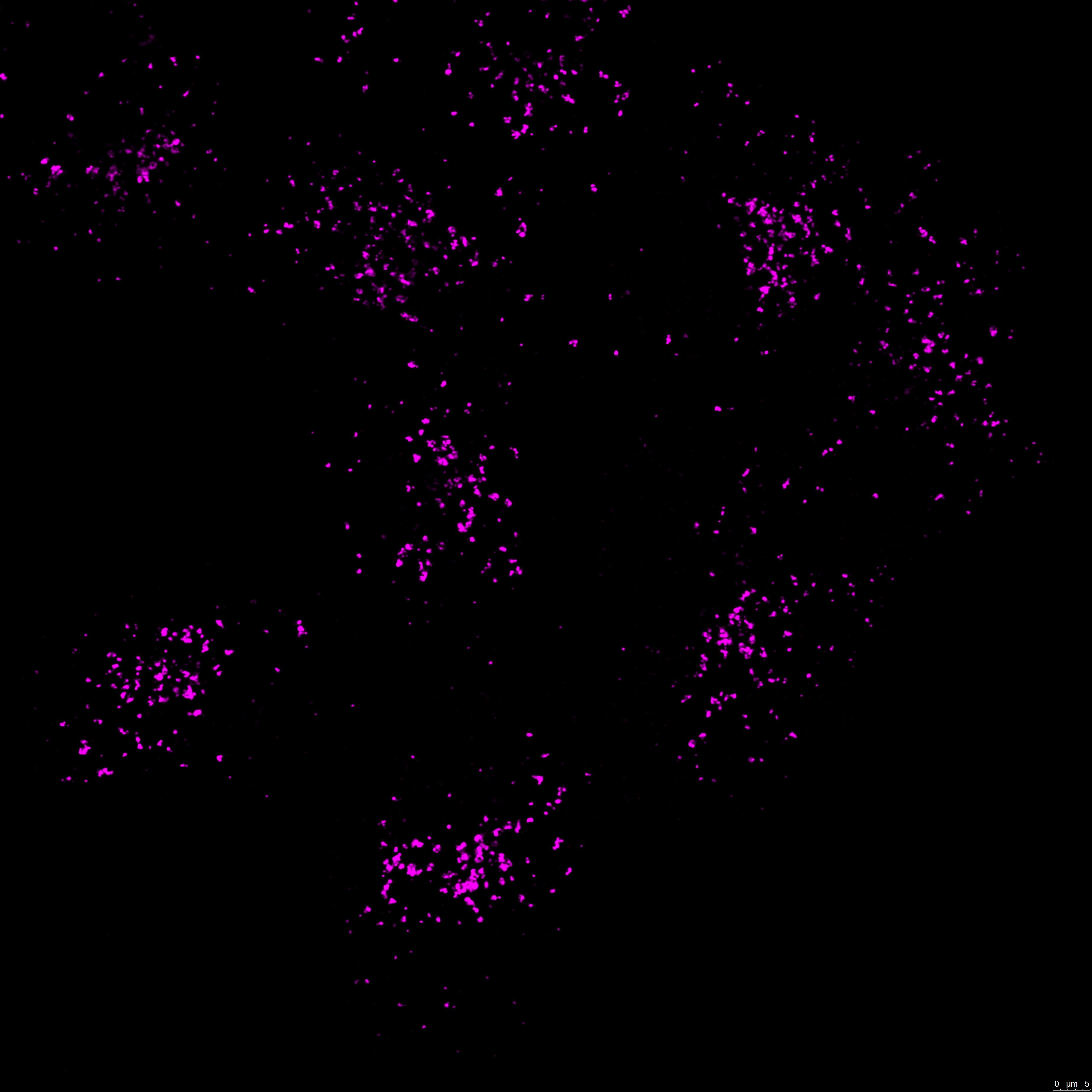

Supplement: Supplementary file 9 — Source data Fig. 2 [file 44318_2025_654_MOESM9_ESM.zip › Figure 2/2A/2A-1 WT-LAMP1.tif]

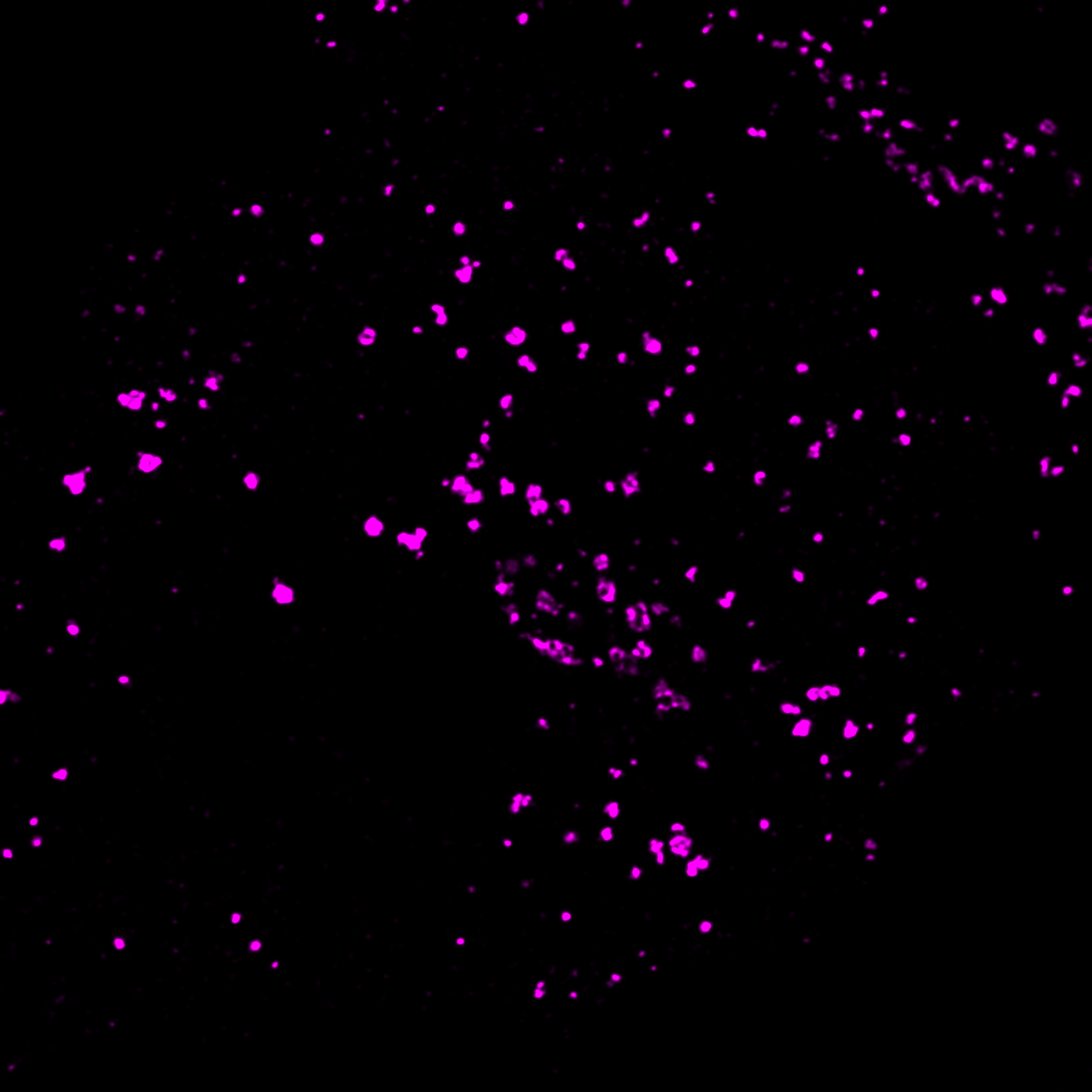

Supplement: Supplementary file 9 — Source data Fig. 2 [file 44318_2025_654_MOESM9_ESM.zip › Figure 2/2A/2A-2 AREL1-KO LAMP1.tif]

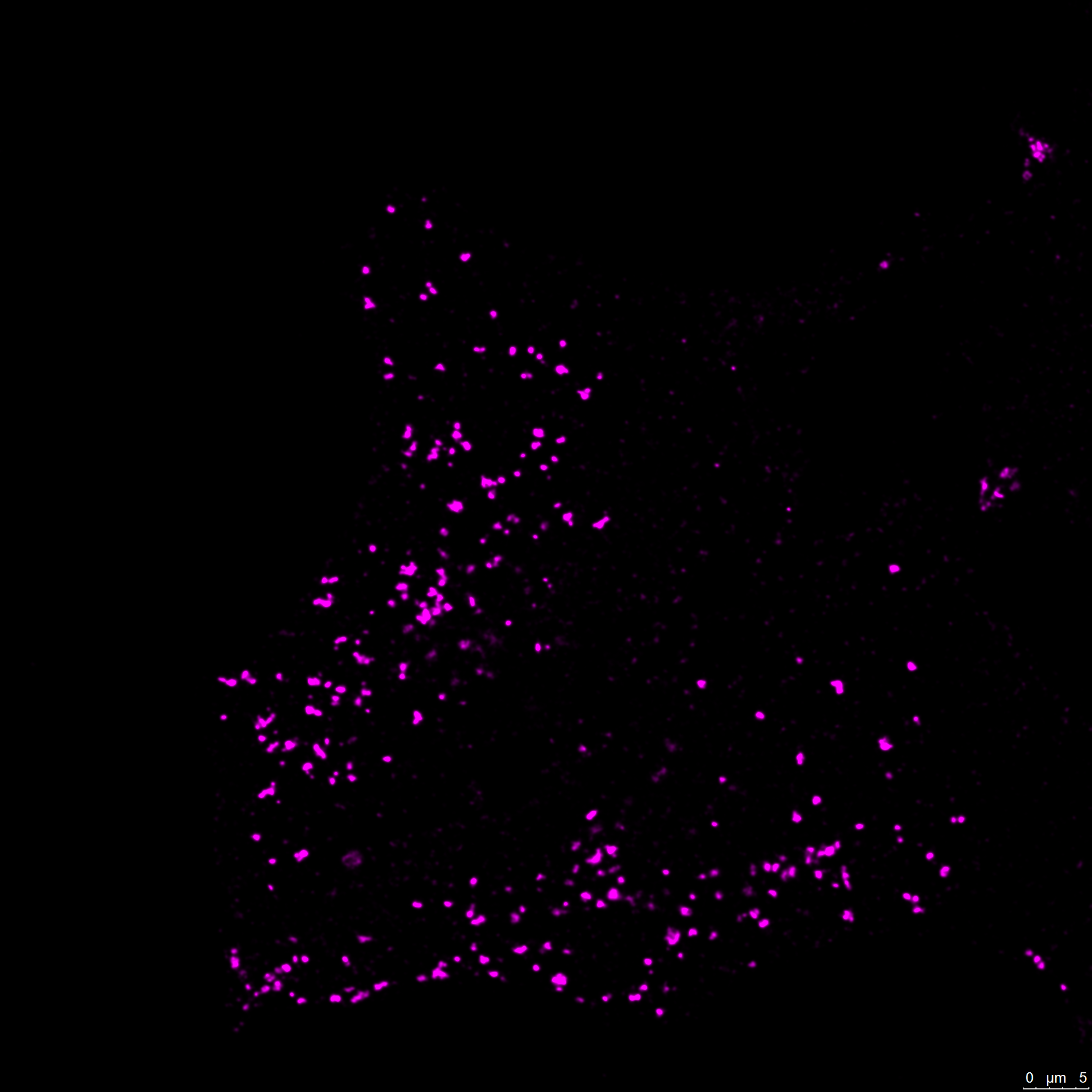

Supplement: Supplementary file 9 — Source data Fig. 2 [file 44318_2025_654_MOESM9_ESM.zip › Figure 2/2F/2F-2 shATP6V0A3 LAMP1.tif]

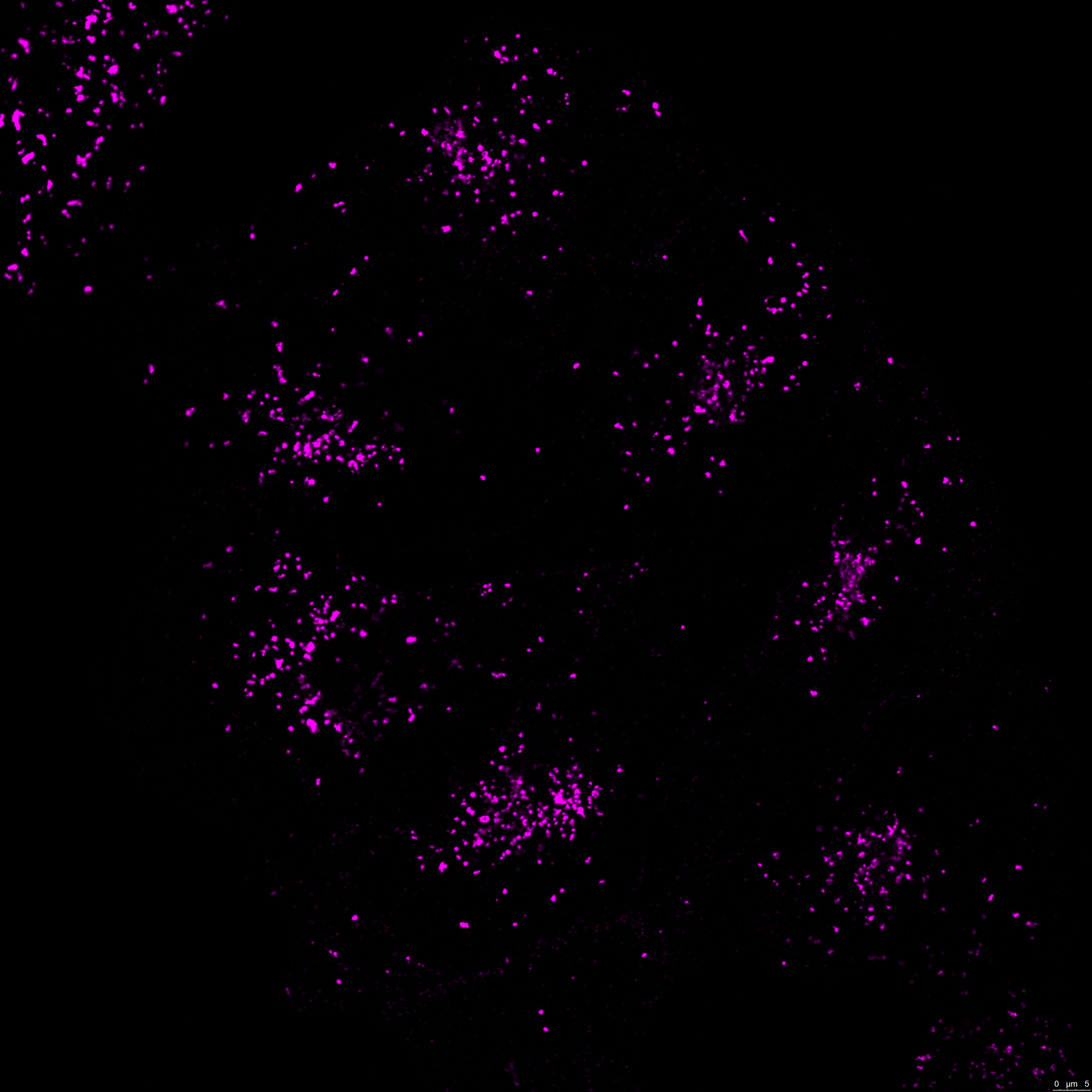

Supplement: Supplementary file 9 — Source data Fig. 2 [file 44318_2025_654_MOESM9_ESM.zip › Figure 2/2F/2F-1 shNC LAMP1.tif]

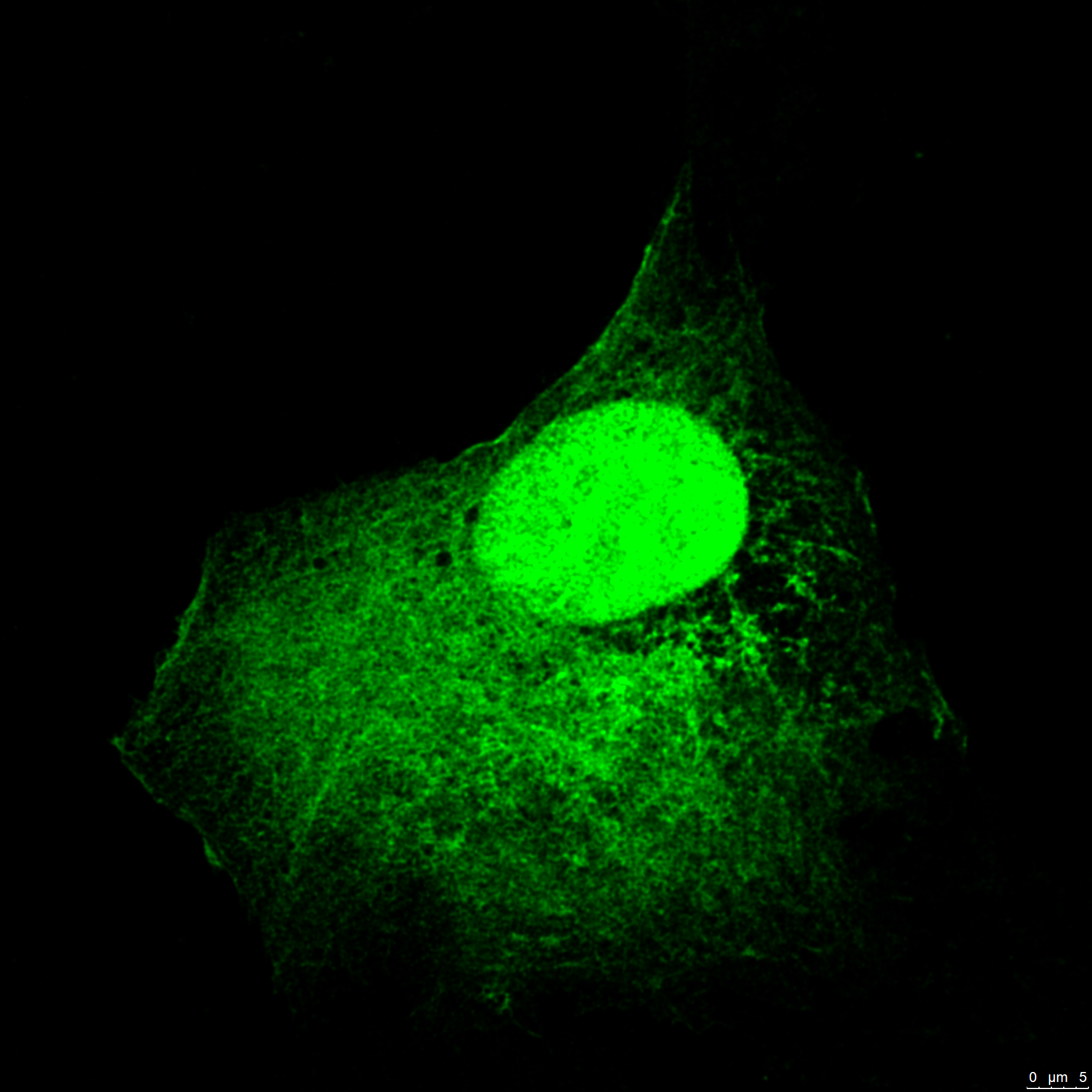

Supplement: Supplementary file 9 — Source data Fig. 2 [file 44318_2025_654_MOESM9_ESM.zip › Figure 2/2D/2D-2-AREL1-KO cell expressing EGFP, EGFP.tif]

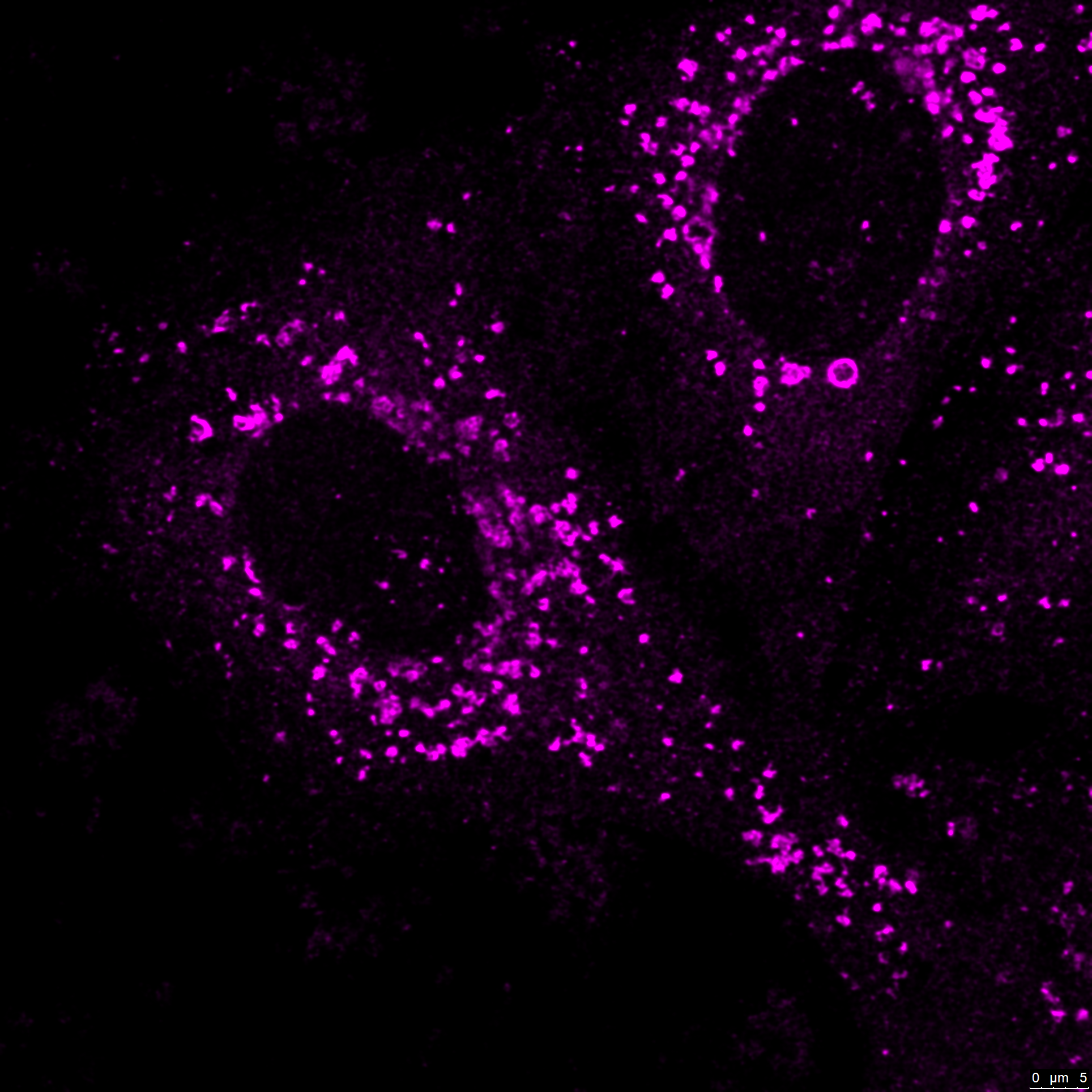

Supplement: Supplementary file 9 — Source data Fig. 2 [file 44318_2025_654_MOESM9_ESM.zip › Figure 2/2D/2D-5-AREL1-KO cell expressing AREL1(╬öhinge region)-EGFP, LAMP1.tif]

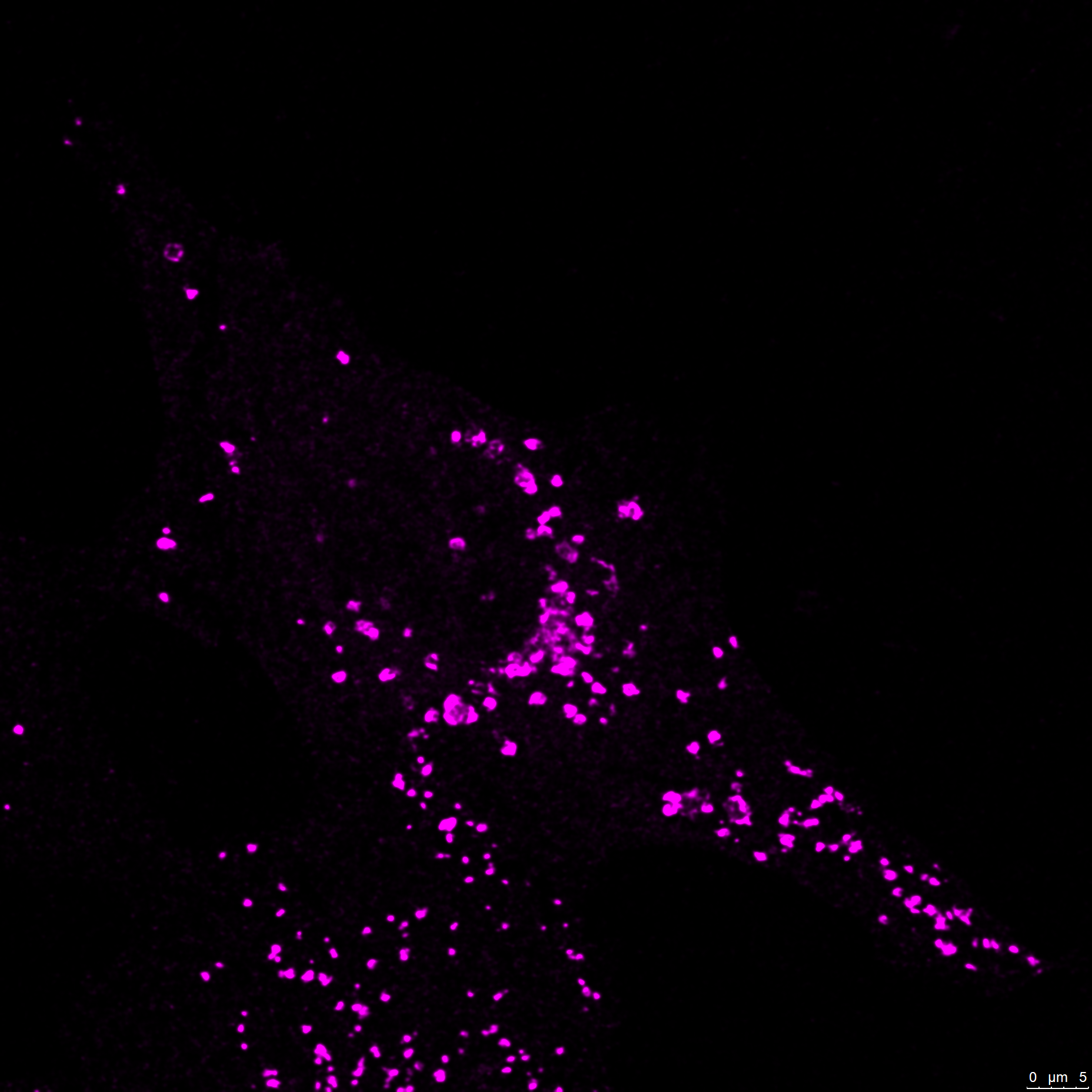

Supplement: Supplementary file 9 — Source data Fig. 2 [file 44318_2025_654_MOESM9_ESM.zip › Figure 2/2D/2D-4-AREL1-KO cell expressing AREL1(C790A)-EGFP, LAMP1.tif]

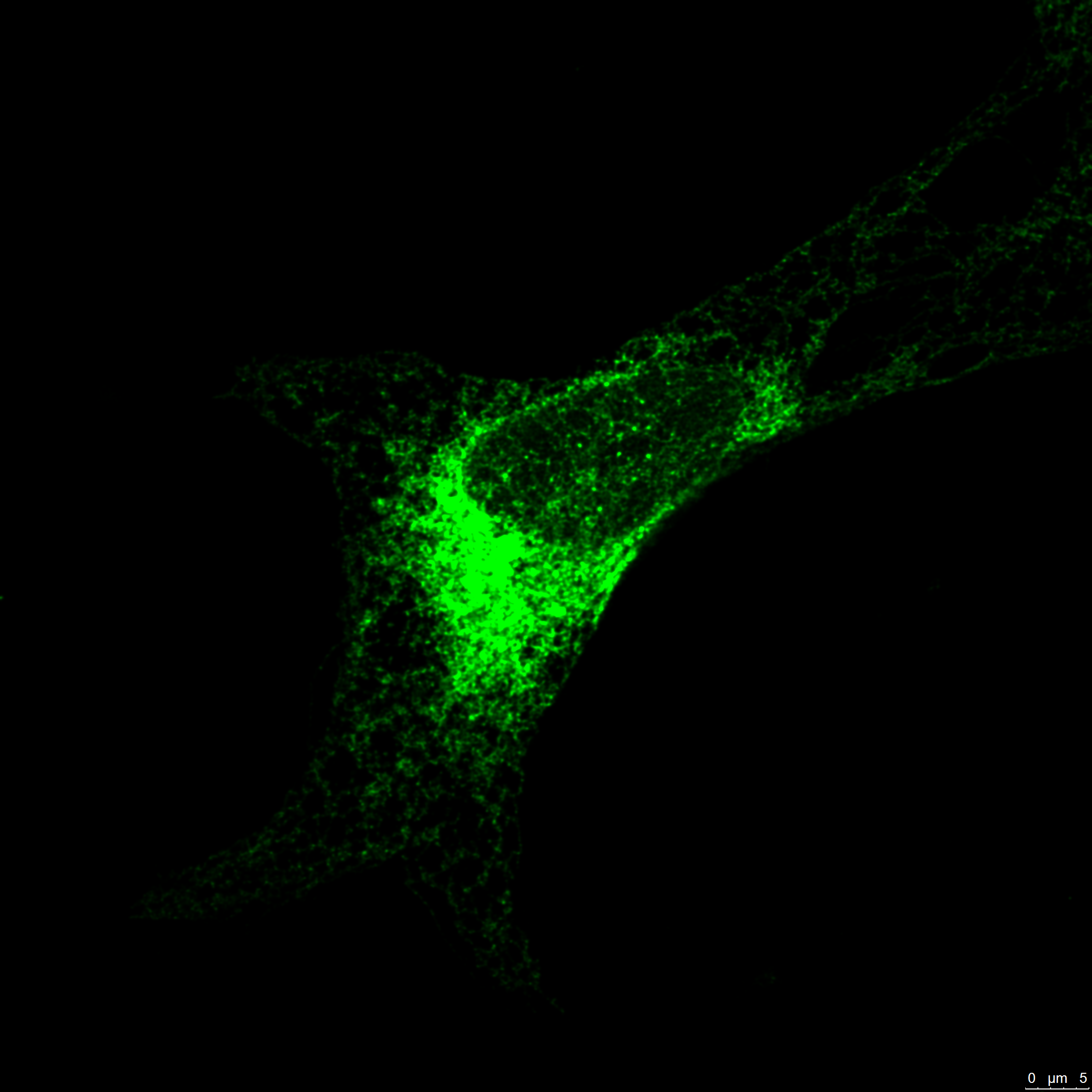

Supplement: Supplementary file 9 — Source data Fig. 2 [file 44318_2025_654_MOESM9_ESM.zip › Figure 2/2D/2D-7-AREL1-KO cell expressing AREL1(Y354A+Y356A)-EGFP, EGFP.tif]

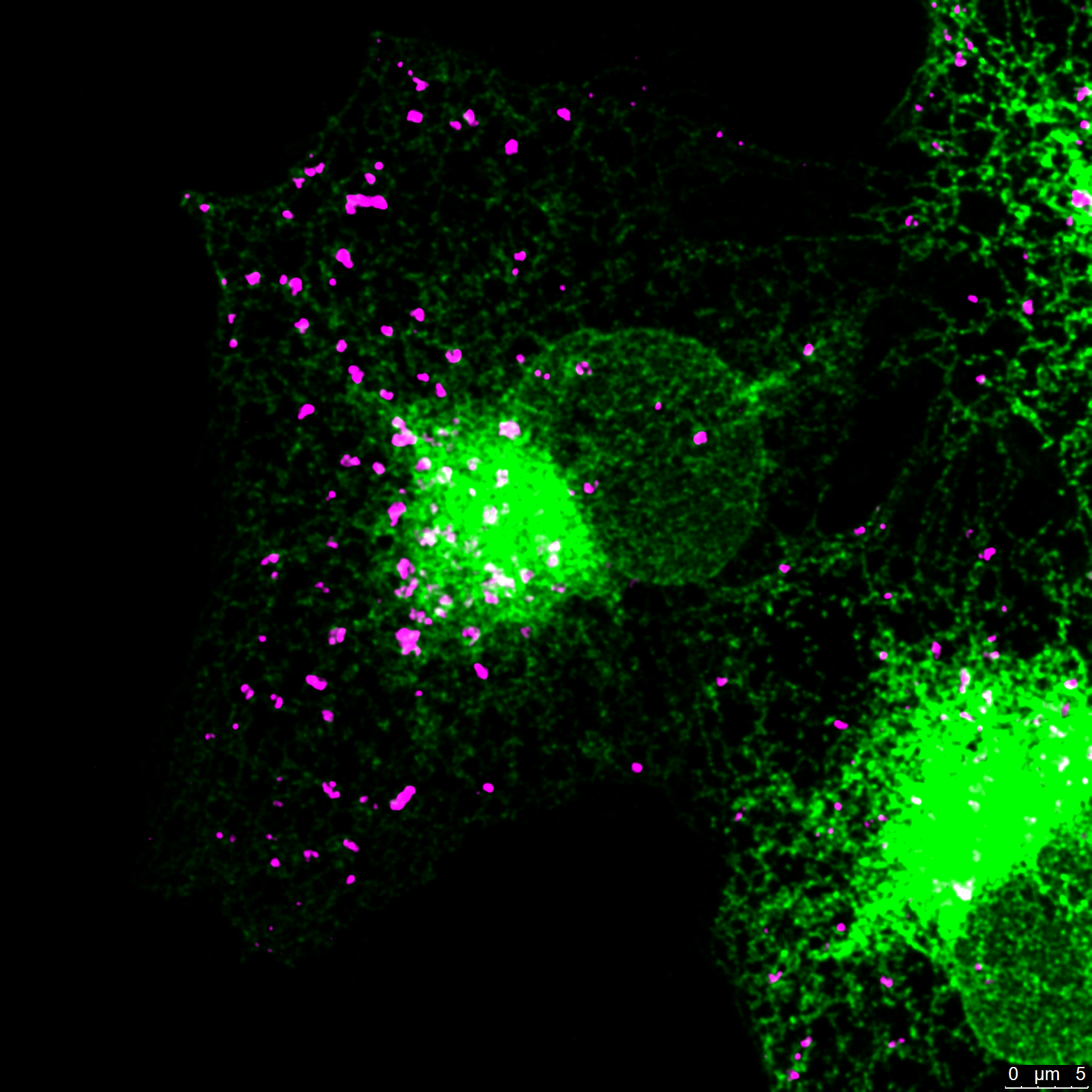

Supplement: Supplementary file 9 — Source data Fig. 2 [file 44318_2025_654_MOESM9_ESM.zip › Figure 2/2D/2D-6-AREL1-KO cell expressing AREL1(╬öLCR)-EGFP, merge.tif]

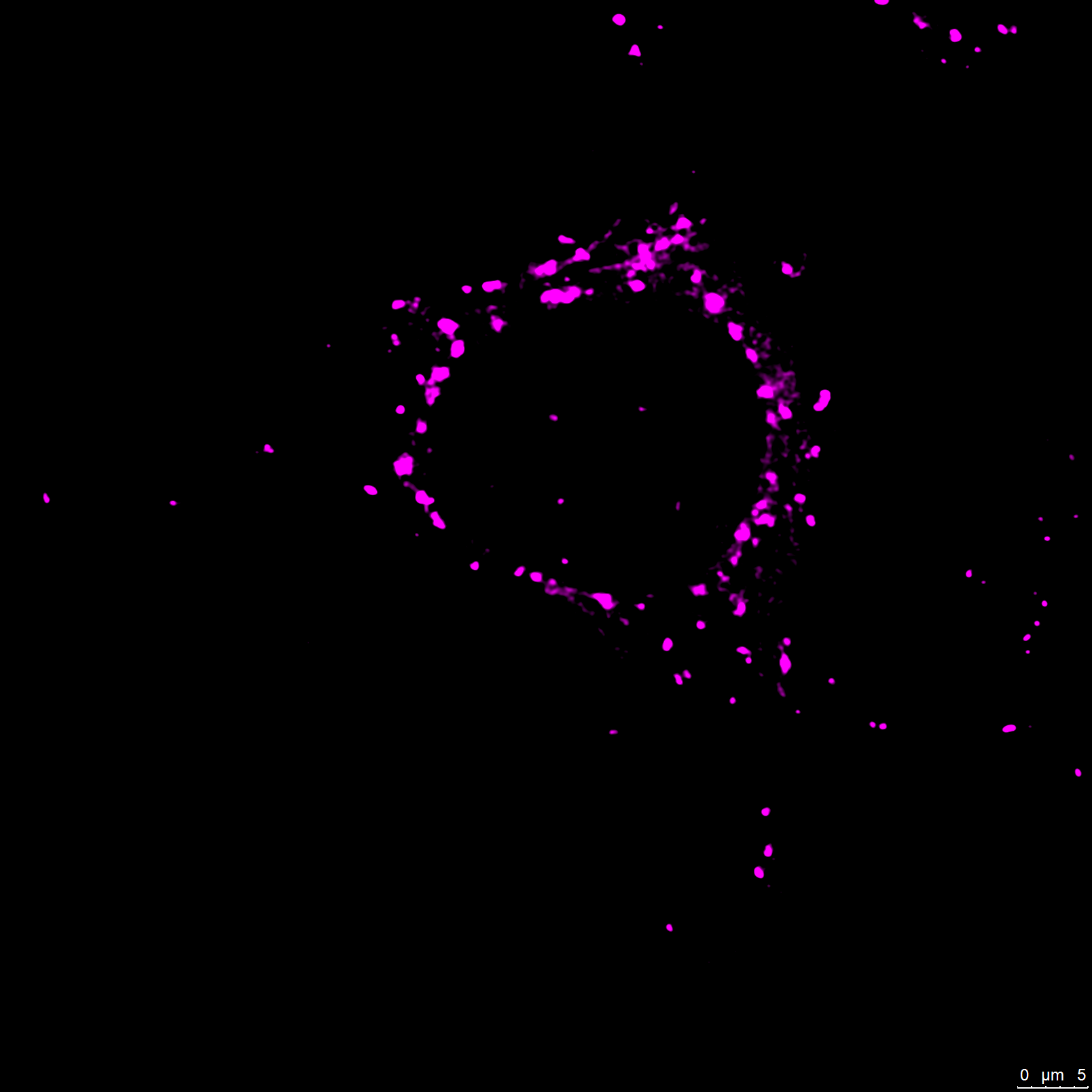

Supplement: Supplementary file 9 — Source data Fig. 2 [file 44318_2025_654_MOESM9_ESM.zip › Figure 2/2D/2D-1-WT cell expressing EGFP, LAMP1.tif]

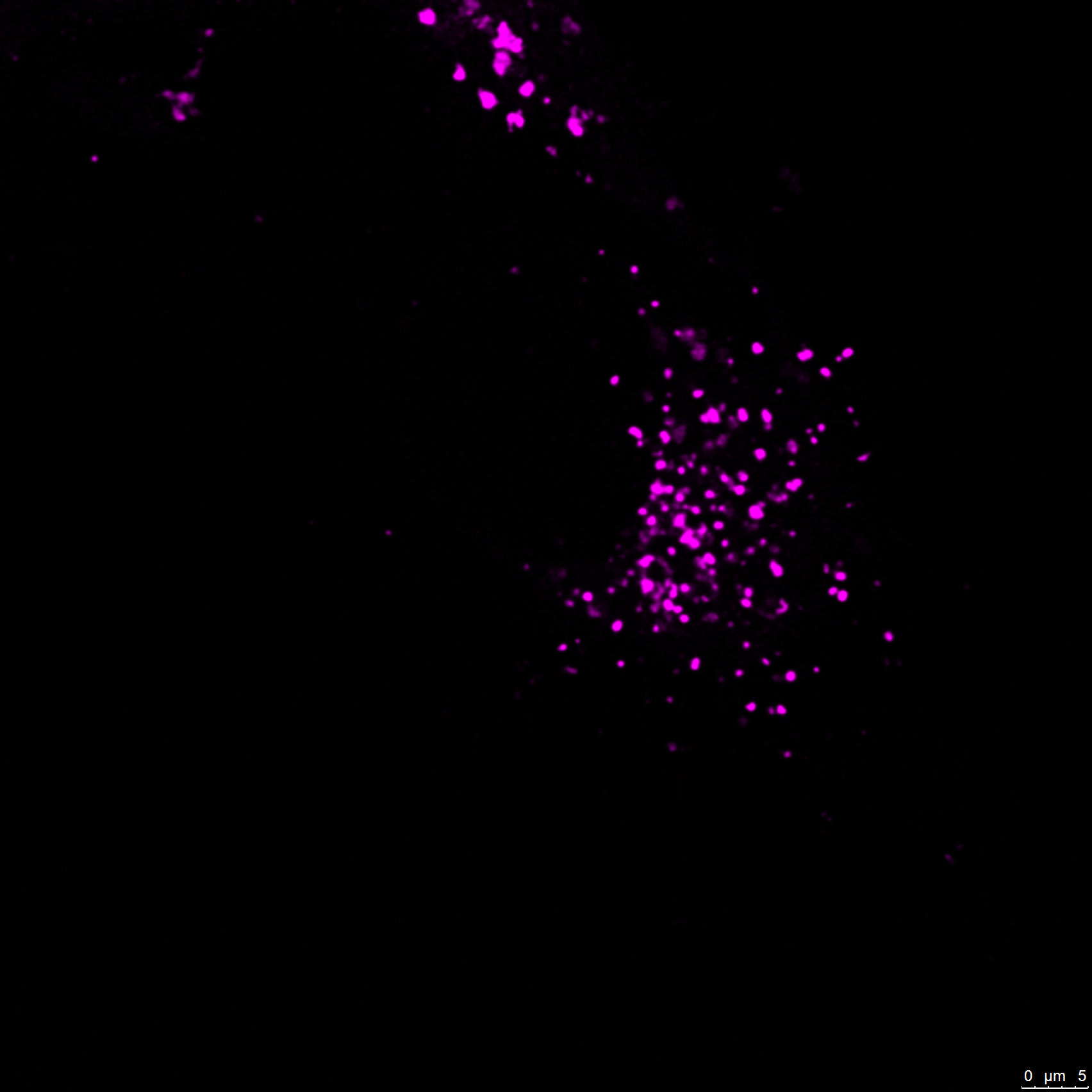

Supplement: Supplementary file 9 — Source data Fig. 2 [file 44318_2025_654_MOESM9_ESM.zip › Figure 2/2D/2D-3-AREL1-KO cell expressing AREL1(WT)-EGFP, LAMP1.tif]

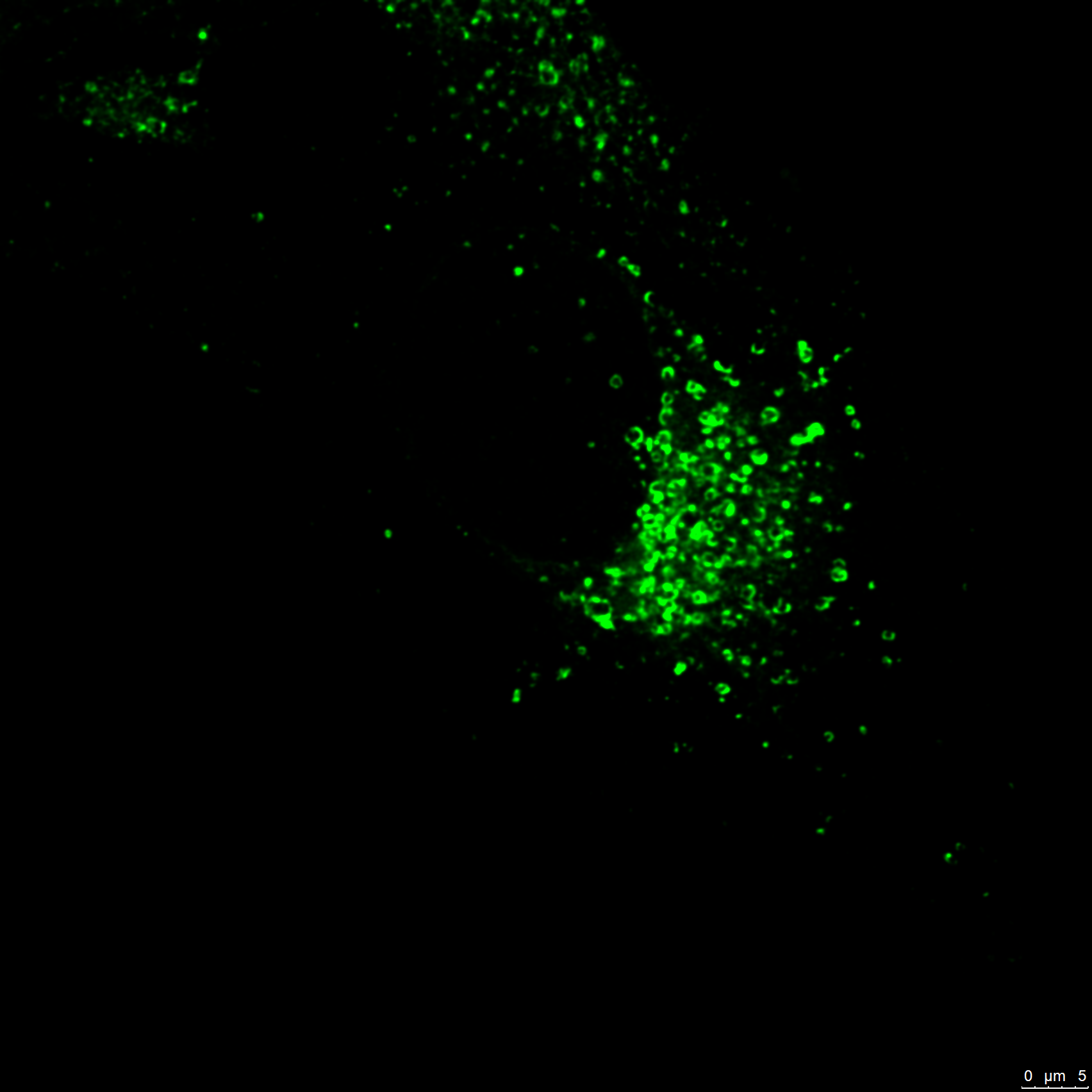

Supplement: Supplementary file 9 — Source data Fig. 2 [file 44318_2025_654_MOESM9_ESM.zip › Figure 2/2D/2D-3-AREL1-KO cell expressing AREL1(WT)-EGFP, EGFP.tif]

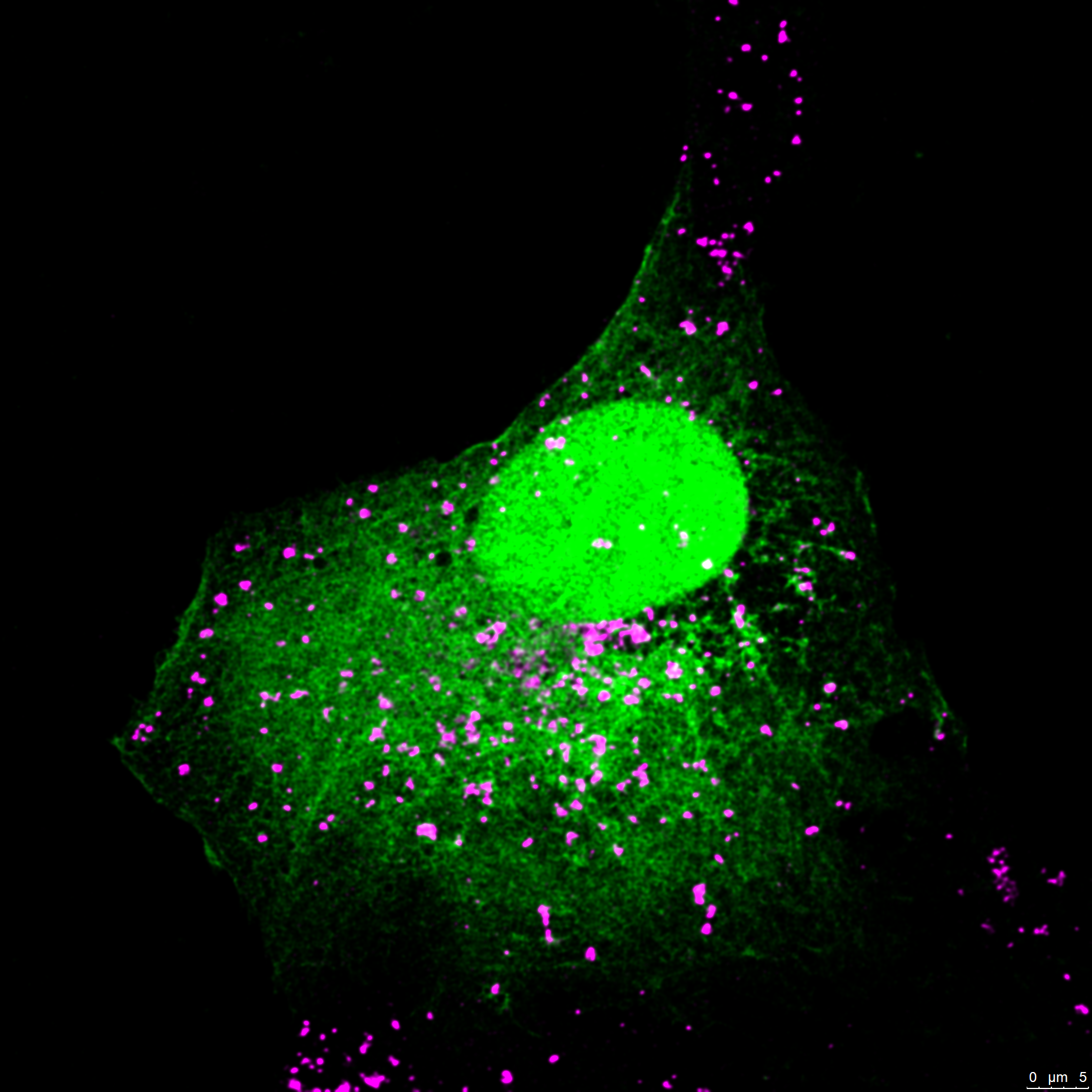

Supplement: Supplementary file 9 — Source data Fig. 2 [file 44318_2025_654_MOESM9_ESM.zip › Figure 2/2D/2D-2-AREL1-KO cell expressing EGFP, merge.tif]

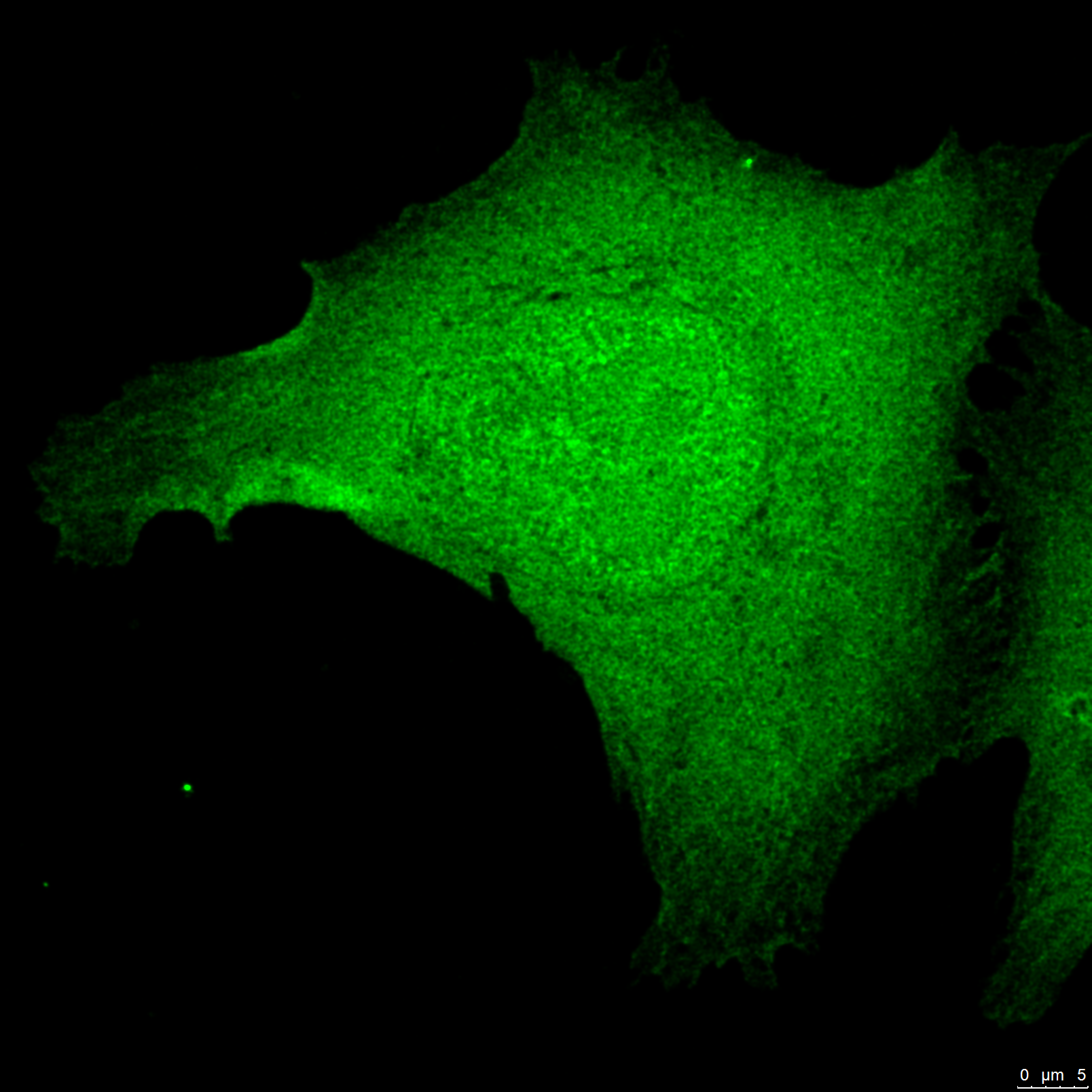

Supplement: Supplementary file 9 — Source data Fig. 2 [file 44318_2025_654_MOESM9_ESM.zip › Figure 2/2D/2D-1-WT cell expressing EGFP, EGFP.tif]

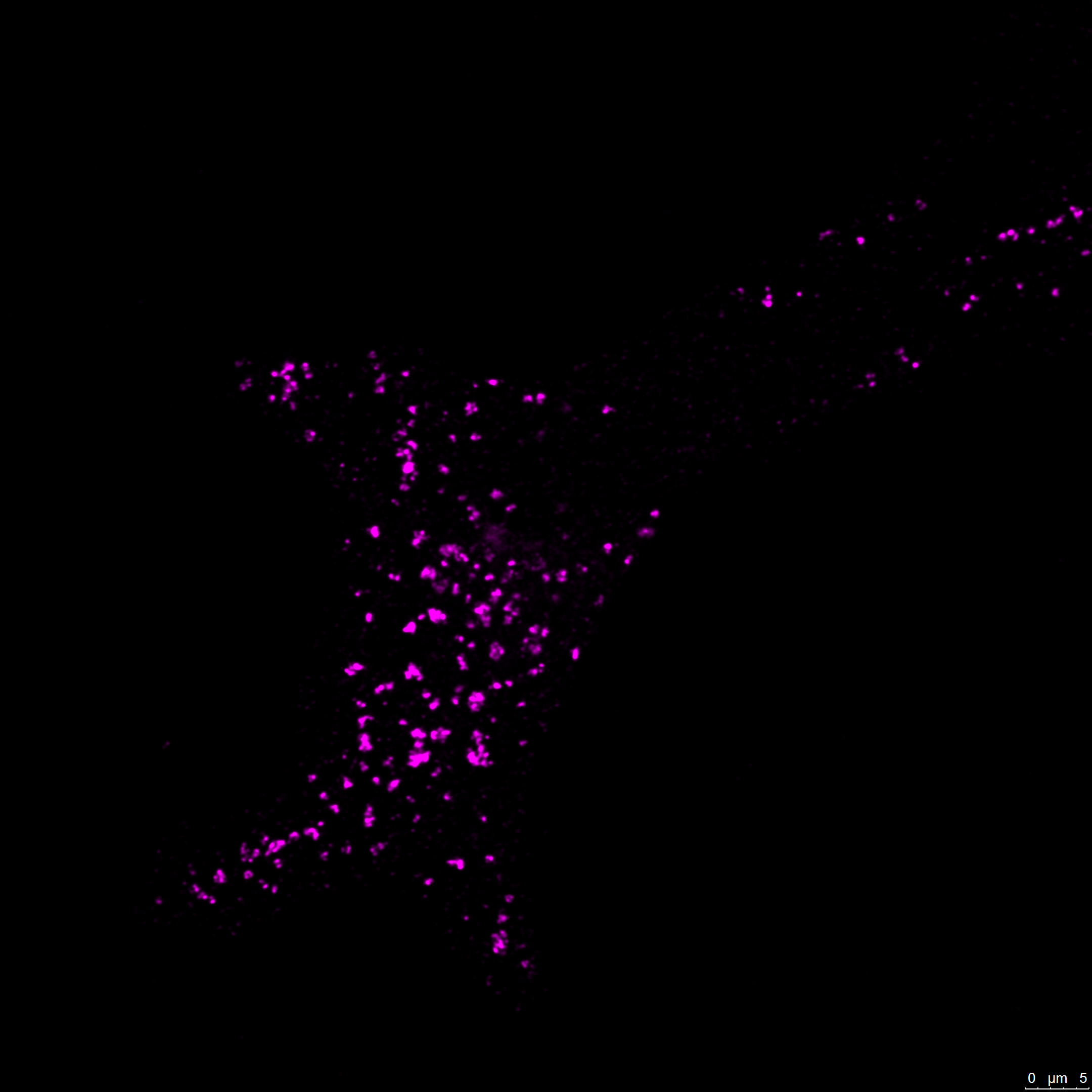

Supplement: Supplementary file 9 — Source data Fig. 2 [file 44318_2025_654_MOESM9_ESM.zip › Figure 2/2D/2D-7-AREL1-KO cell expressing AREL1(Y354A+Y356A)-EGFP, LAMP1.tif]

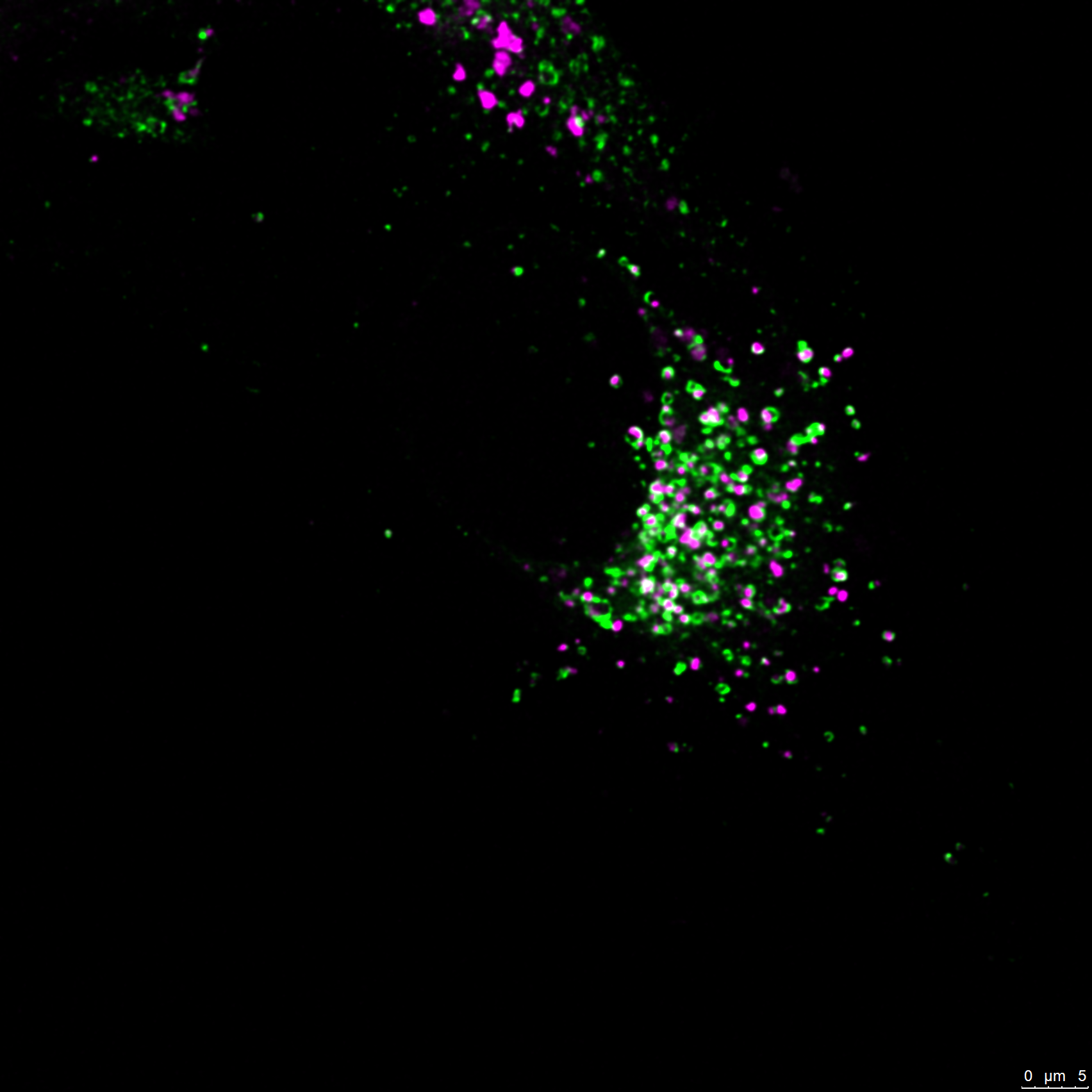

Supplement: Supplementary file 9 — Source data Fig. 2 [file 44318_2025_654_MOESM9_ESM.zip › Figure 2/2D/2D-3-AREL1-KO cell expressing AREL1(WT)-EGFP, merge.tif]

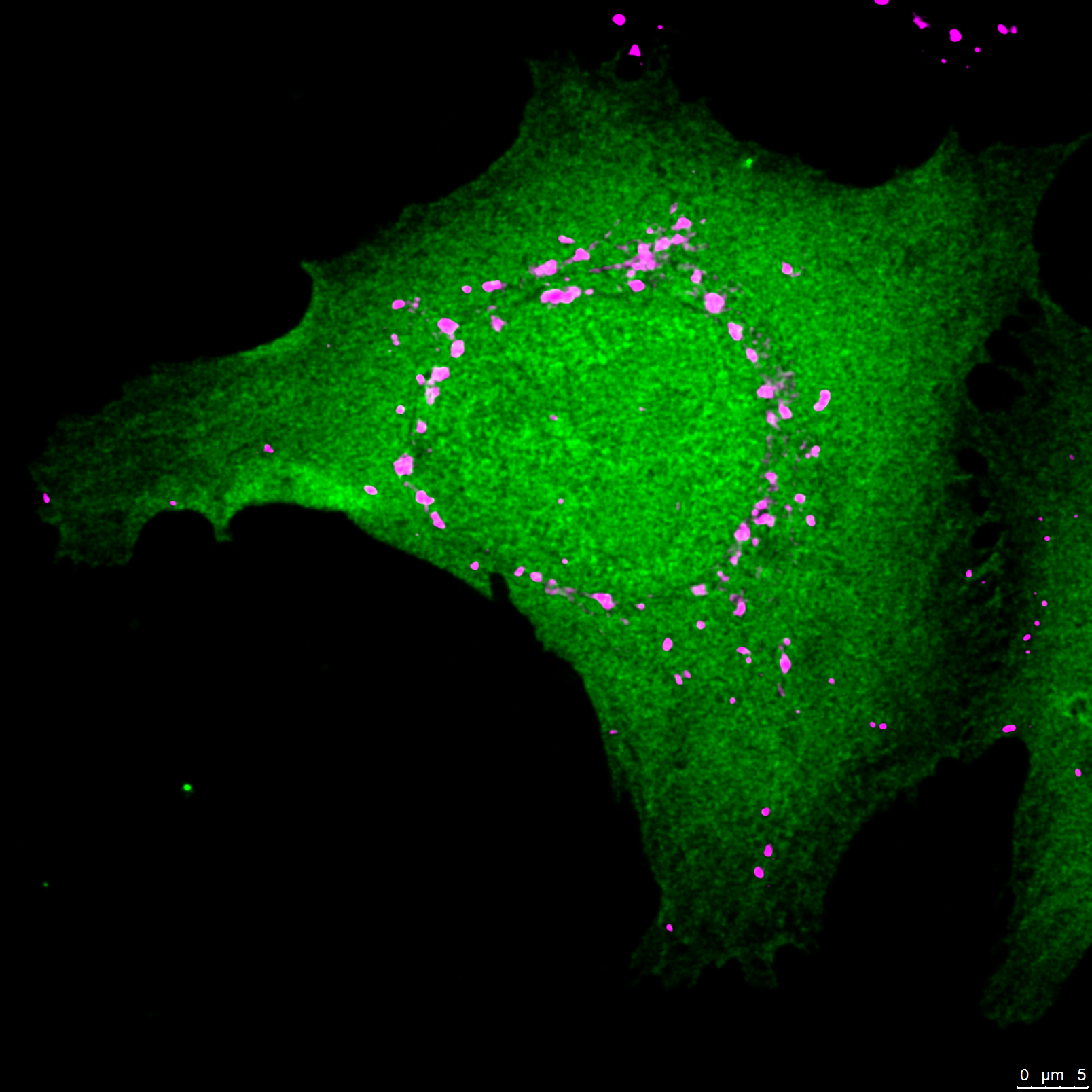

Supplement: Supplementary file 9 — Source data Fig. 2 [file 44318_2025_654_MOESM9_ESM.zip › Figure 2/2D/2D-1-WT cell expressing EGFP, merge.tif]

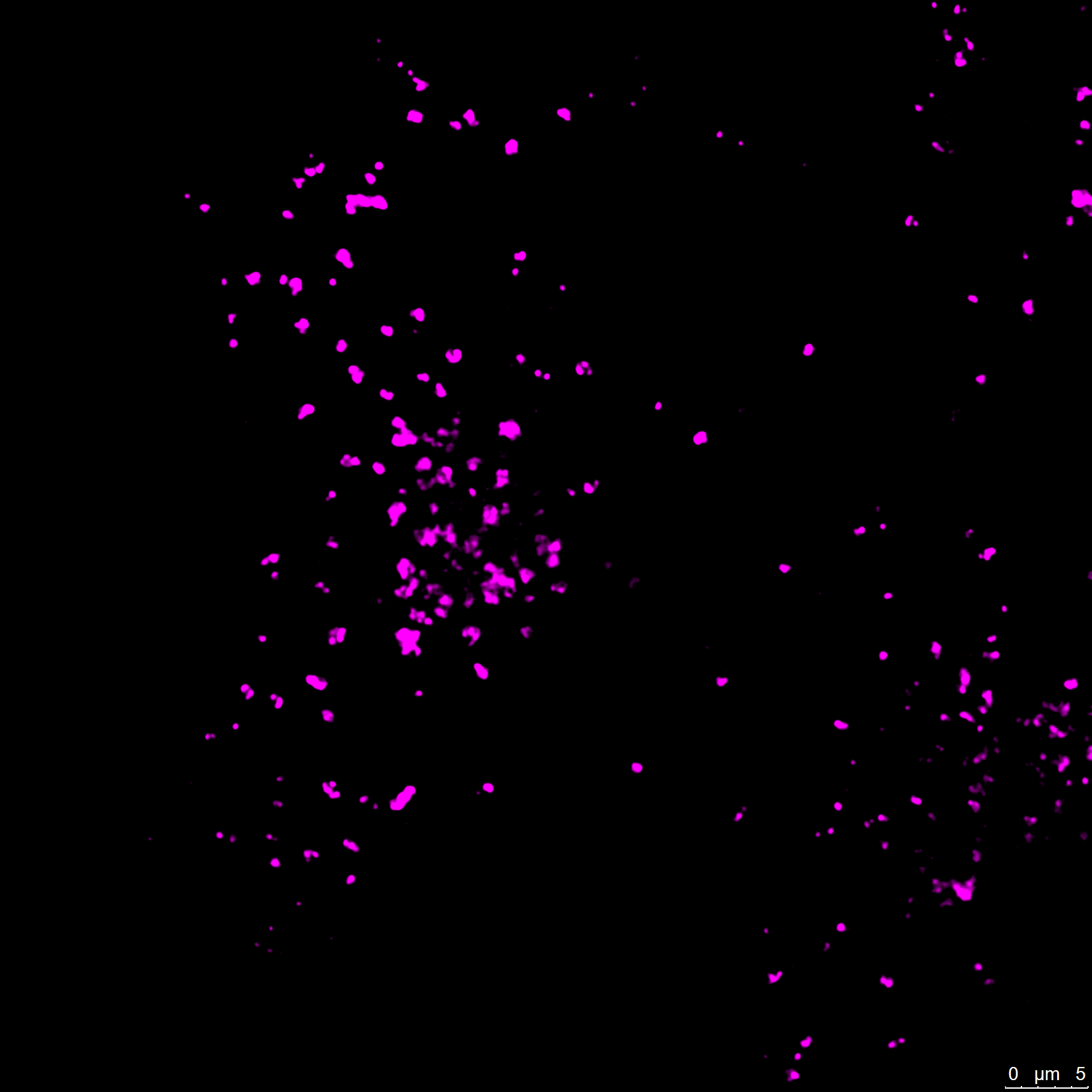

Supplement: Supplementary file 9 — Source data Fig. 2 [file 44318_2025_654_MOESM9_ESM.zip › Figure 2/2D/2D-6-AREL1-KO cell expressing AREL1(╬öLCR)-EGFP, LAMP1.tif]

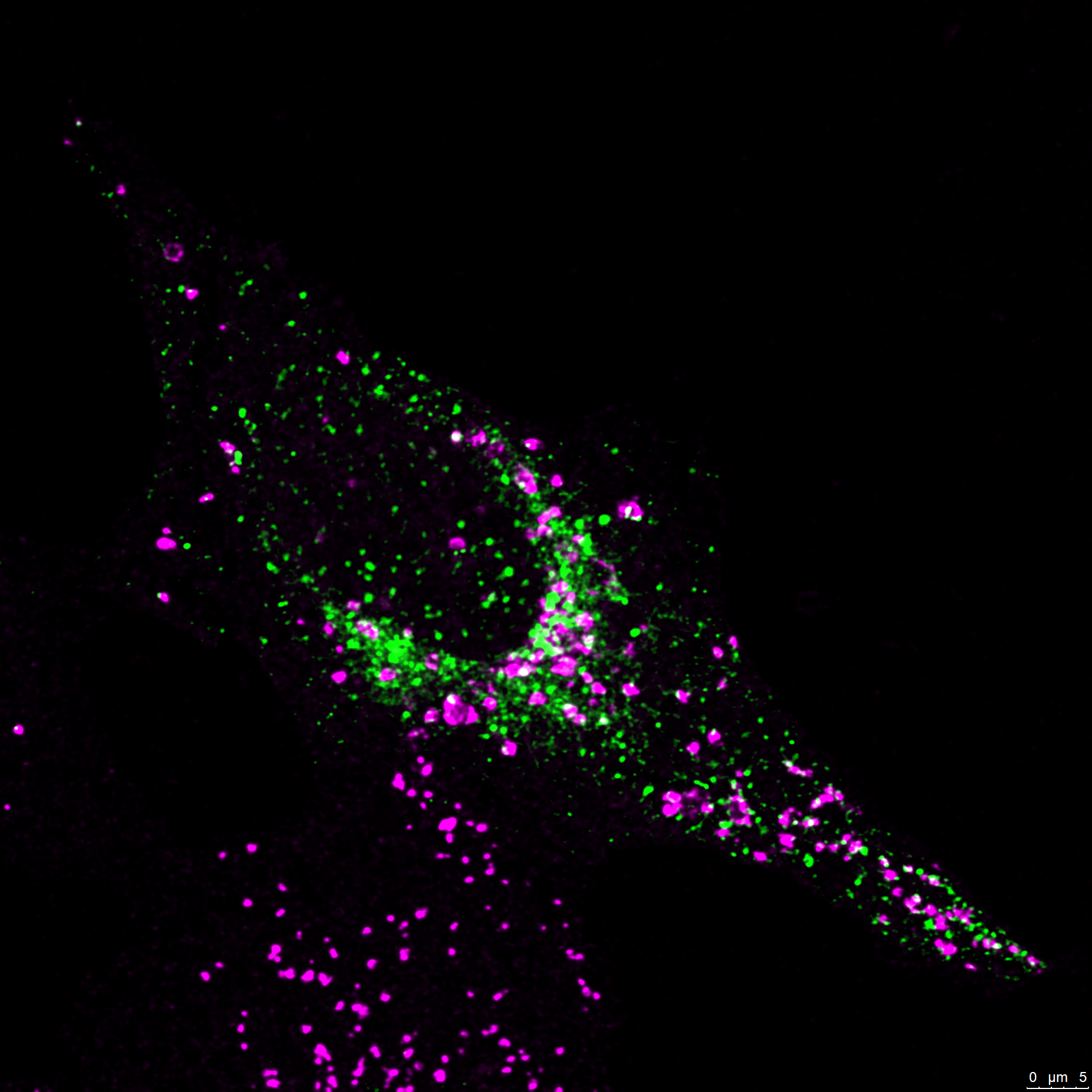

Supplement: Supplementary file 9 — Source data Fig. 2 [file 44318_2025_654_MOESM9_ESM.zip › Figure 2/2D/2D-4-AREL1-KO cell expressing AREL1(C790A)-EGFP, merge.tif]

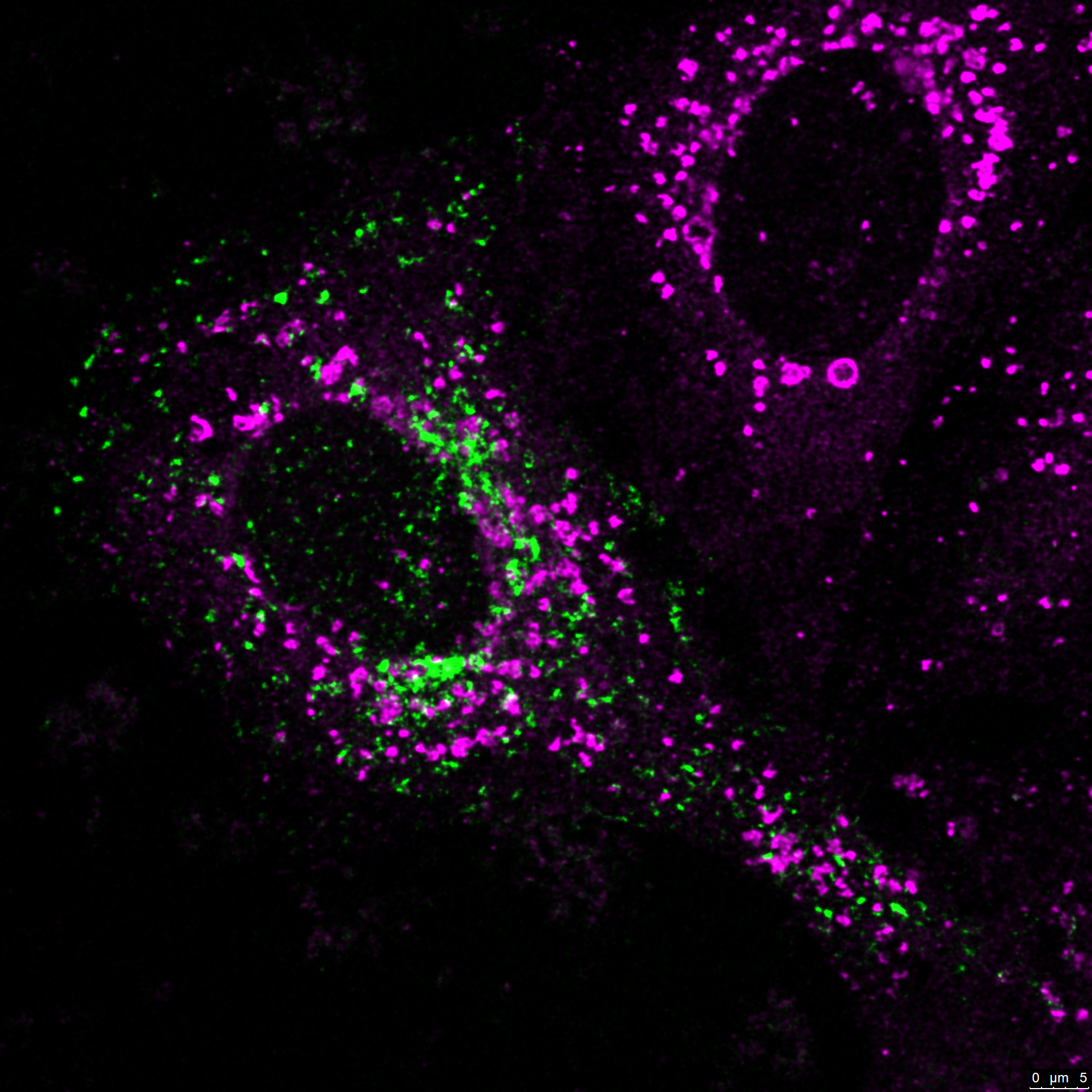

Supplement: Supplementary file 9 — Source data Fig. 2 [file 44318_2025_654_MOESM9_ESM.zip › Figure 2/2D/2D-5-AREL1-KO cell expressing AREL1(╬öhinge region)-EGFP, merge.tif]

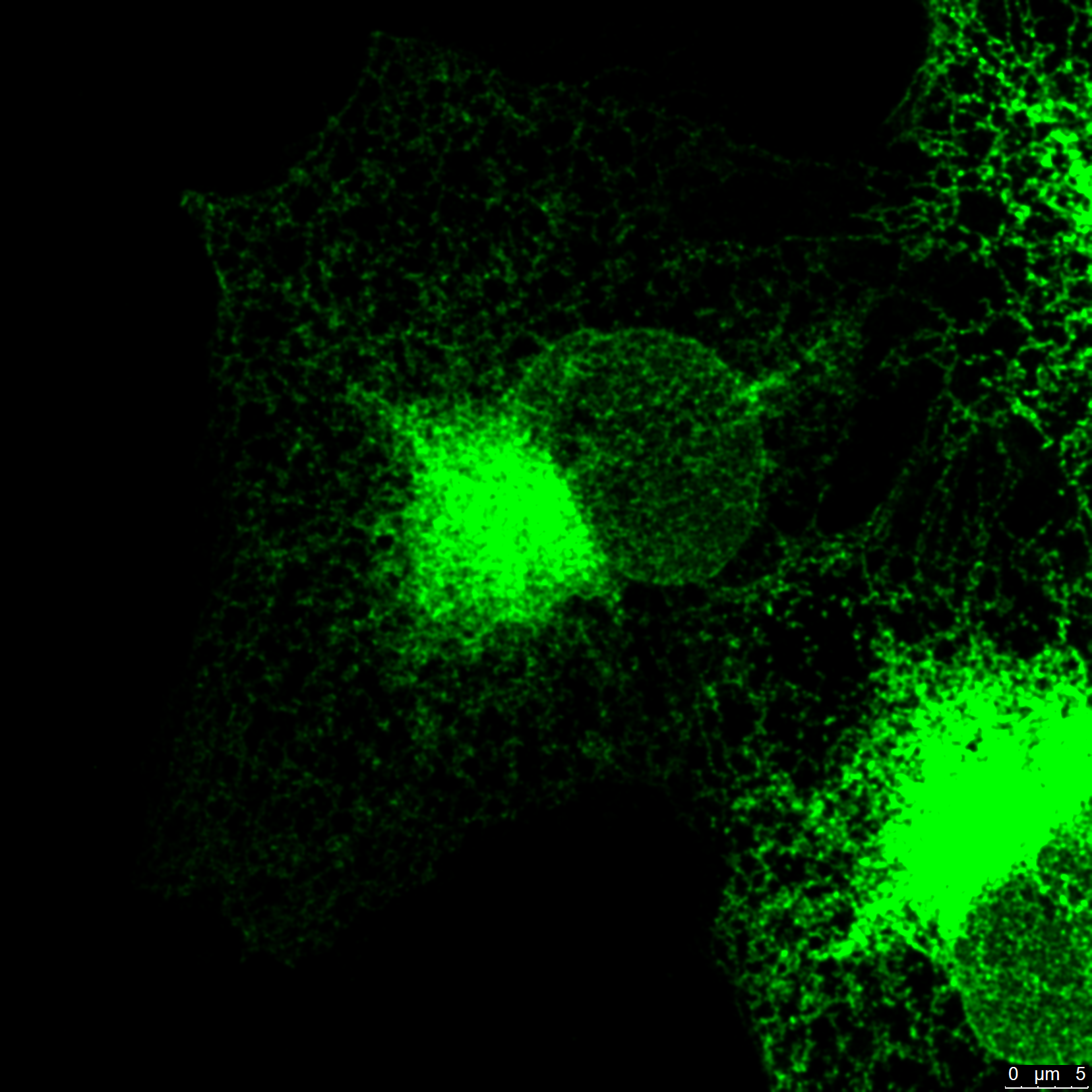

Supplement: Supplementary file 9 — Source data Fig. 2 [file 44318_2025_654_MOESM9_ESM.zip › Figure 2/2D/2D-6-AREL1-KO cell expressing AREL1(╬öLCR)-EGFP, EGFP.tif]

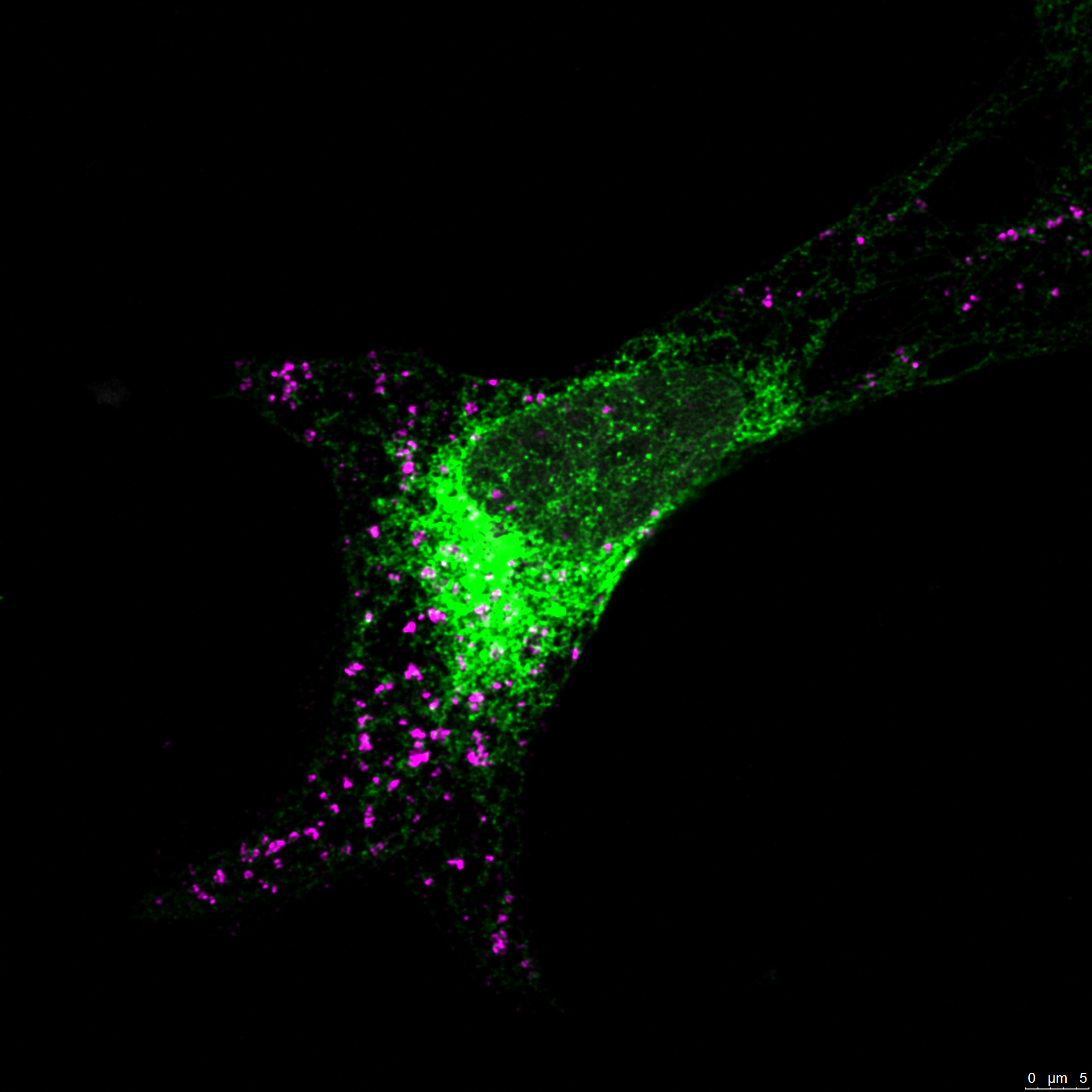

Supplement: Supplementary file 9 — Source data Fig. 2 [file 44318_2025_654_MOESM9_ESM.zip › Figure 2/2D/2D-7-AREL1-KO cell expressing AREL1(Y354A+Y356A)-EGFP, merge.tif]

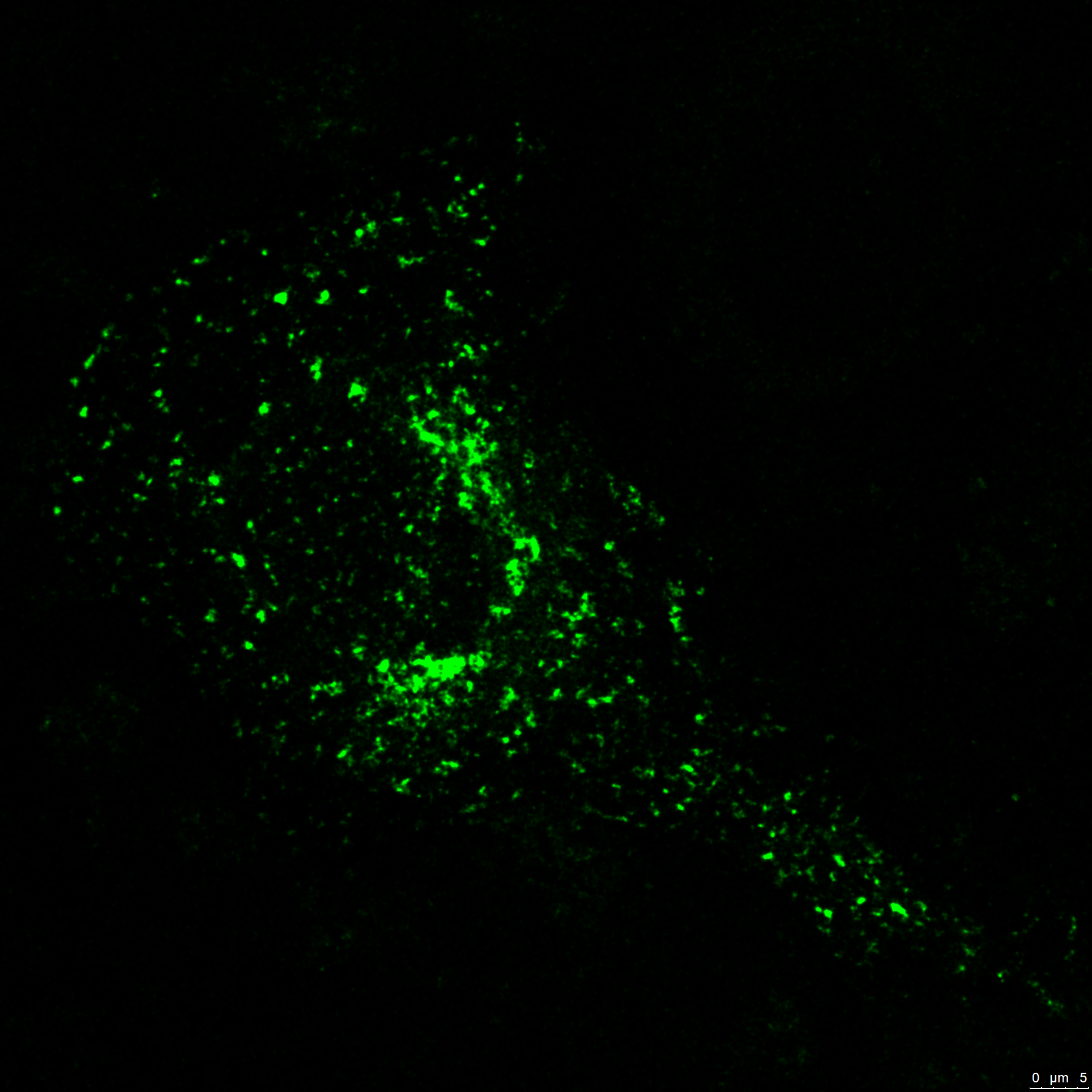

Supplement: Supplementary file 9 — Source data Fig. 2 [file 44318_2025_654_MOESM9_ESM.zip › Figure 2/2D/2D-5-AREL1-KO cell expressing AREL1(╬öhinge region)-EGFP, EGFP.tif]

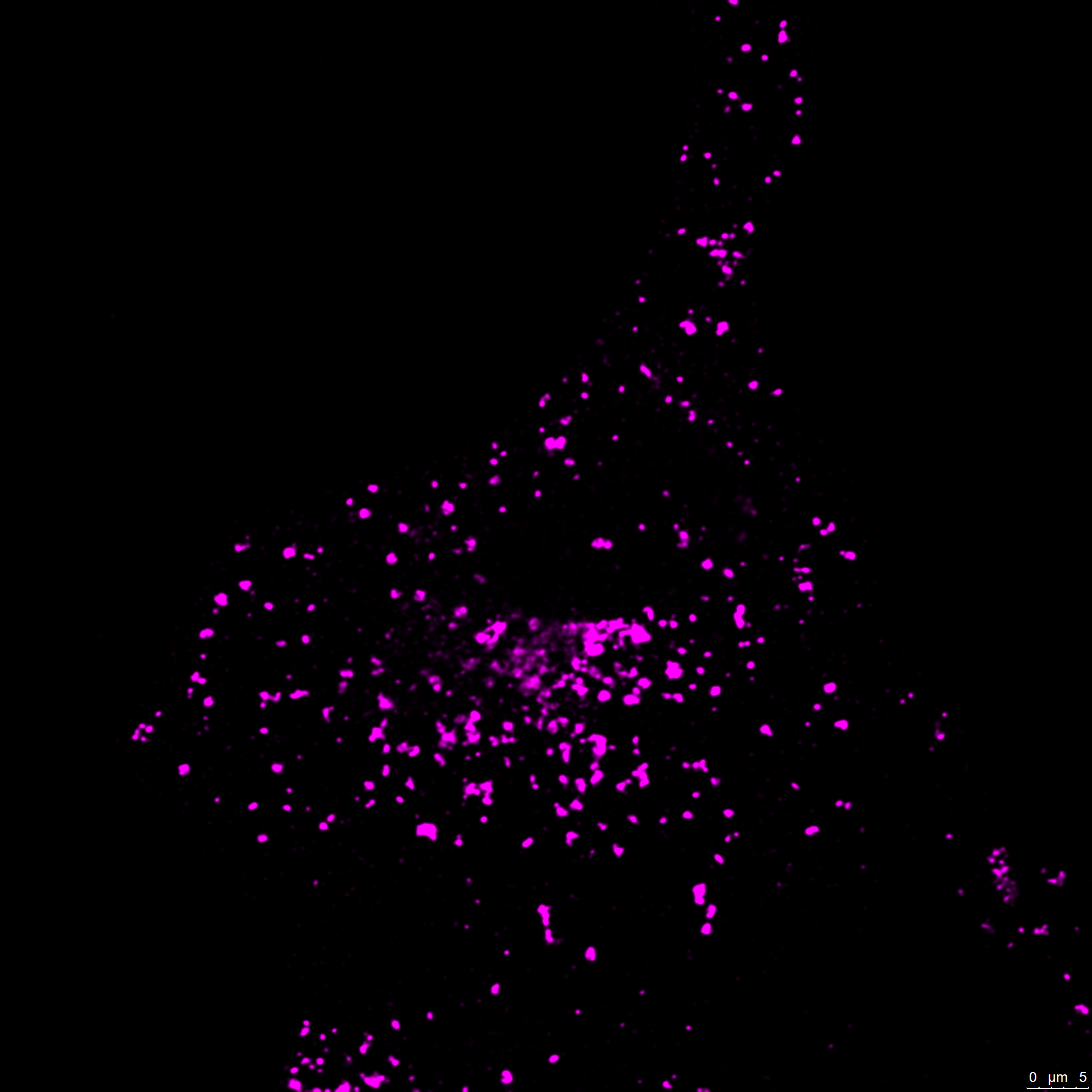

Supplement: Supplementary file 9 — Source data Fig. 2 [file 44318_2025_654_MOESM9_ESM.zip › Figure 2/2D/2D-2-AREL1-KO cell expressing EGFP, LAMP1.tif]

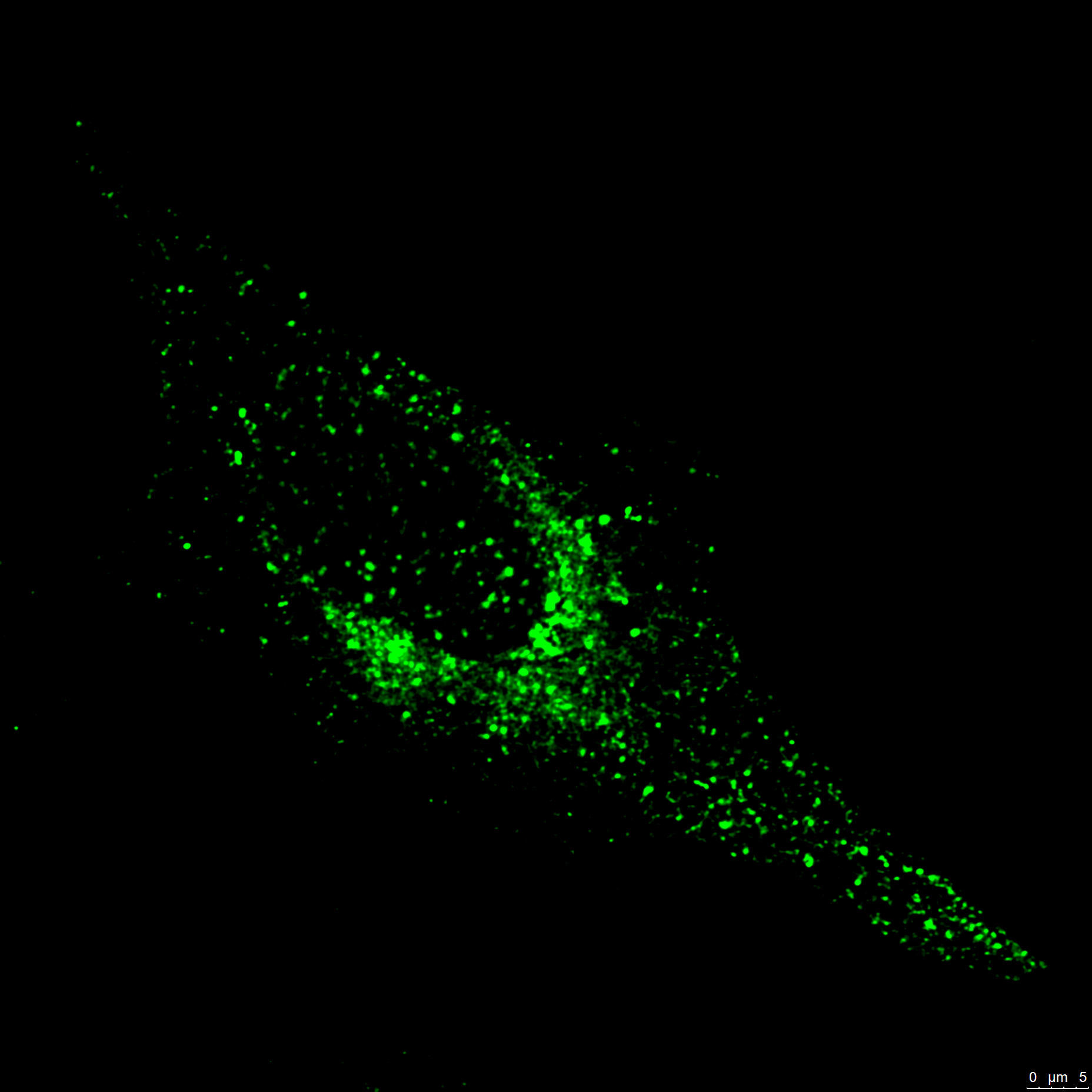

Supplement: Supplementary file 9 — Source data Fig. 2 [file 44318_2025_654_MOESM9_ESM.zip › Figure 2/2D/2D-4-AREL1-KO cell expressing AREL1(C790A)-EGFP, EGFP.tif]

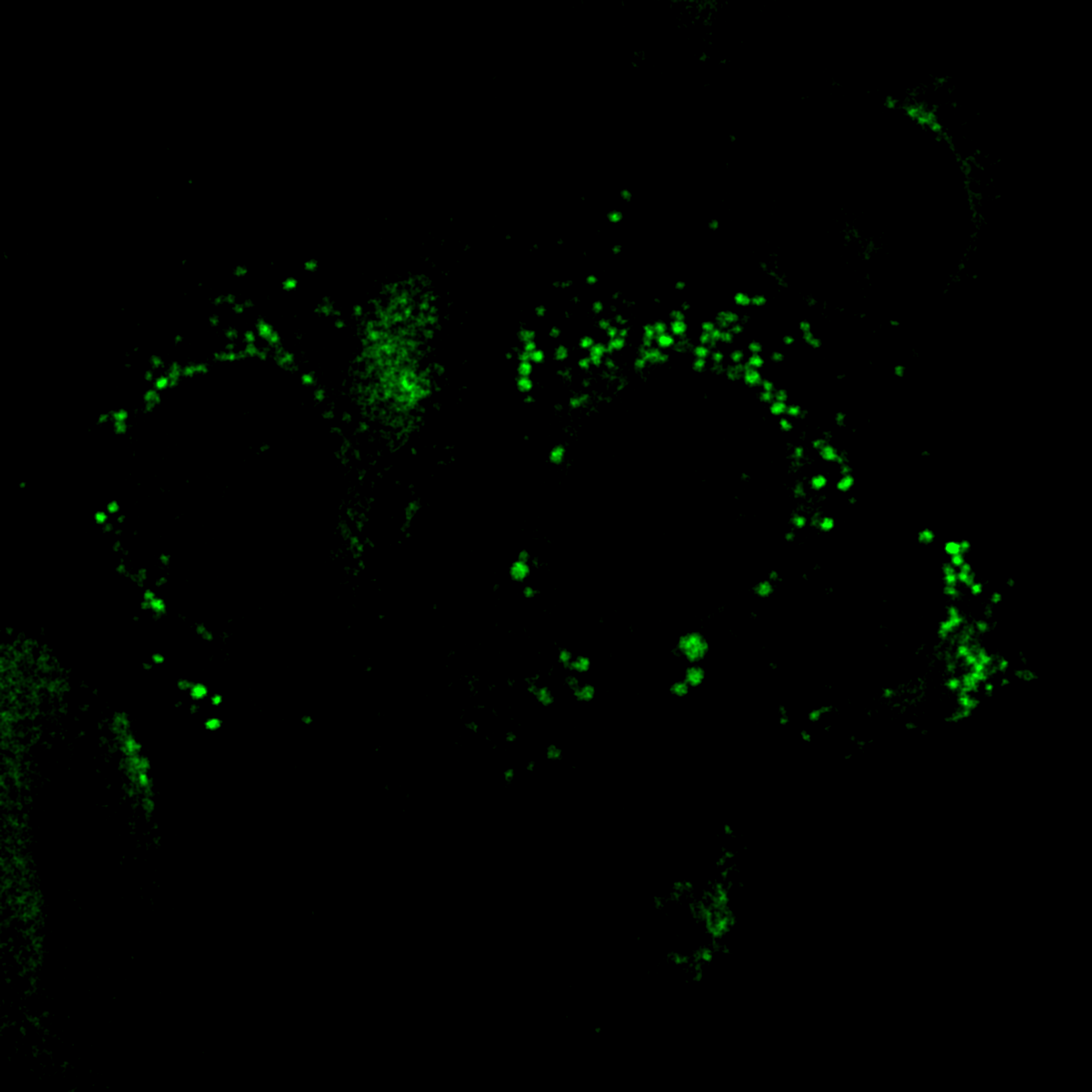

Supplement: Supplementary file 10 — Source data Fig. 3 [file 44318_2025_654_MOESM10_ESM.zip › Figure 3 /3B/3B-1 WT-Lysosensor Green.tif]

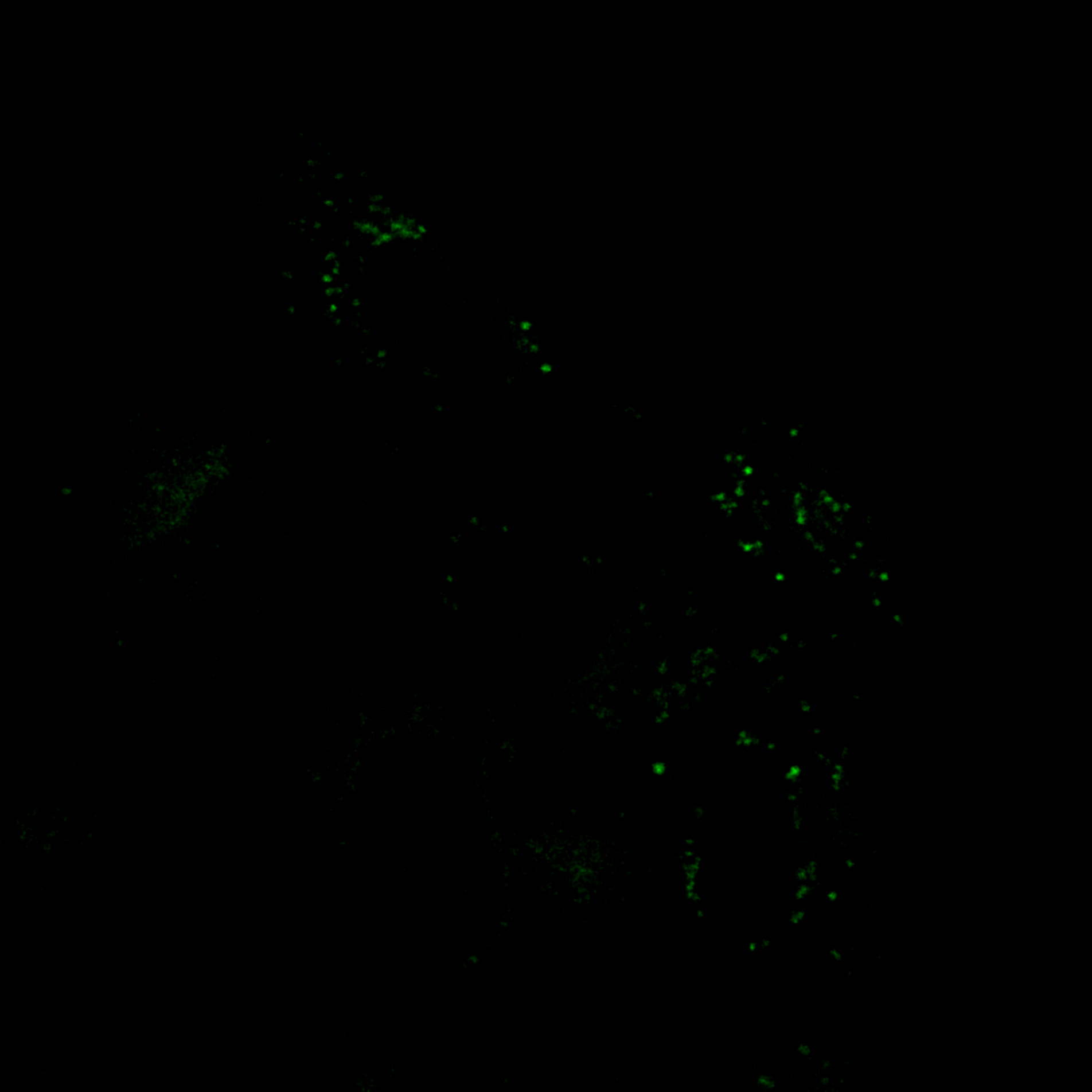

Supplement: Supplementary file 10 — Source data Fig. 3 [file 44318_2025_654_MOESM10_ESM.zip › Figure 3 /3B/3B-2 AREL1 KO-Lysosensor Green.tif]

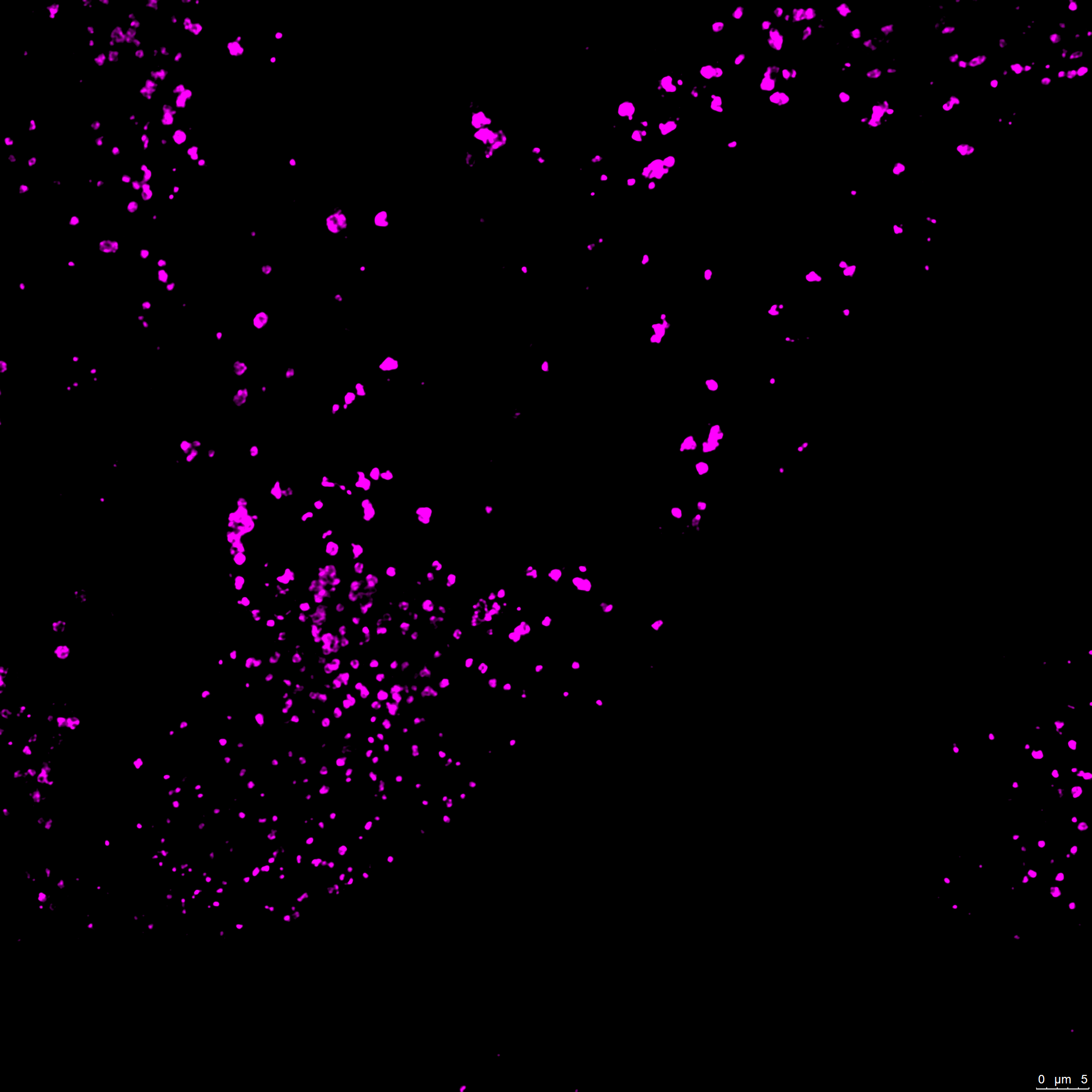

Supplement: Supplementary file 10 — Source data Fig. 3 [file 44318_2025_654_MOESM10_ESM.zip › Figure 3 /3D/3D-2 AREL1-KO LAMP1.tif]

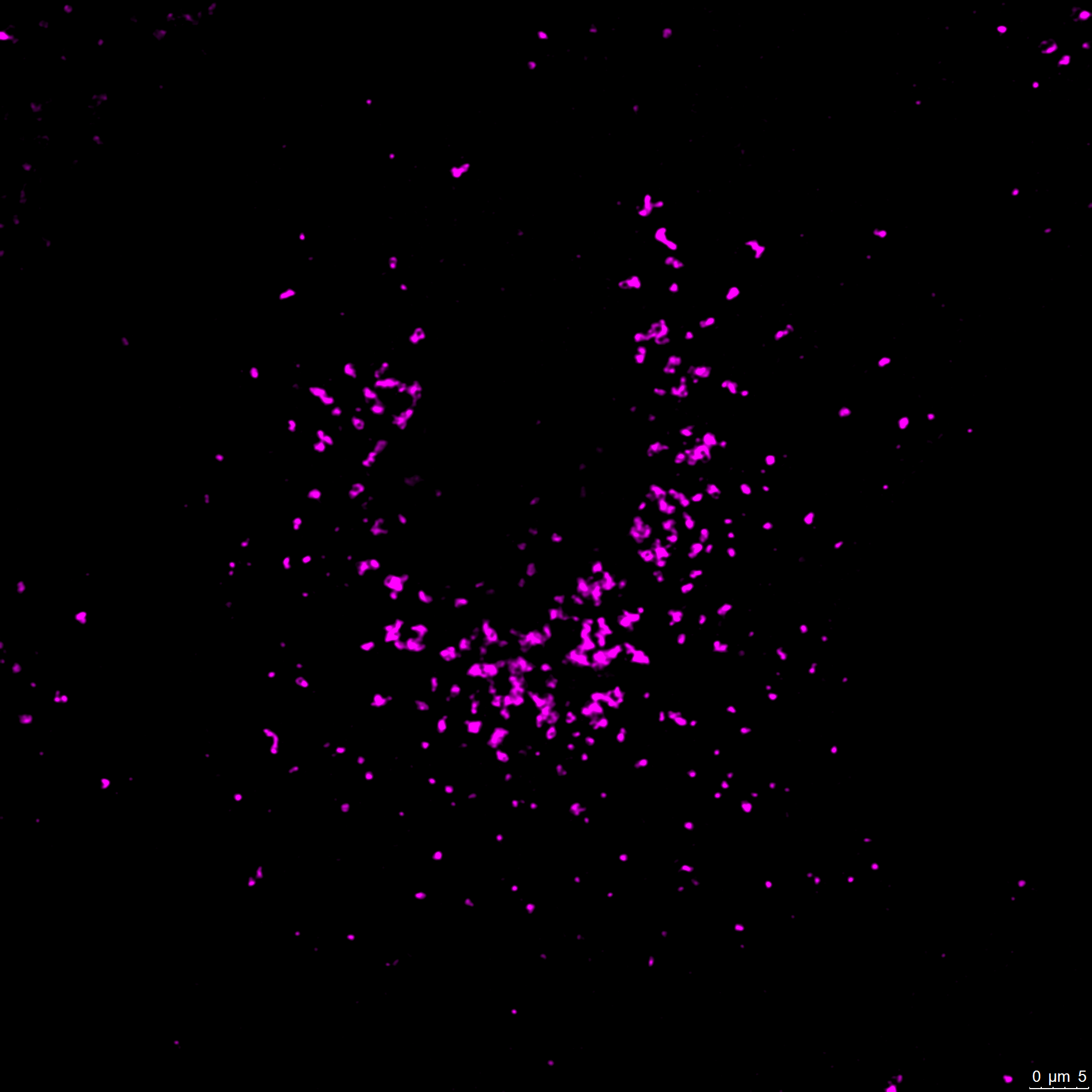

Supplement: Supplementary file 10 — Source data Fig. 3 [file 44318_2025_654_MOESM10_ESM.zip › Figure 3 /3D/3D-1 WT LAMP1.tif]

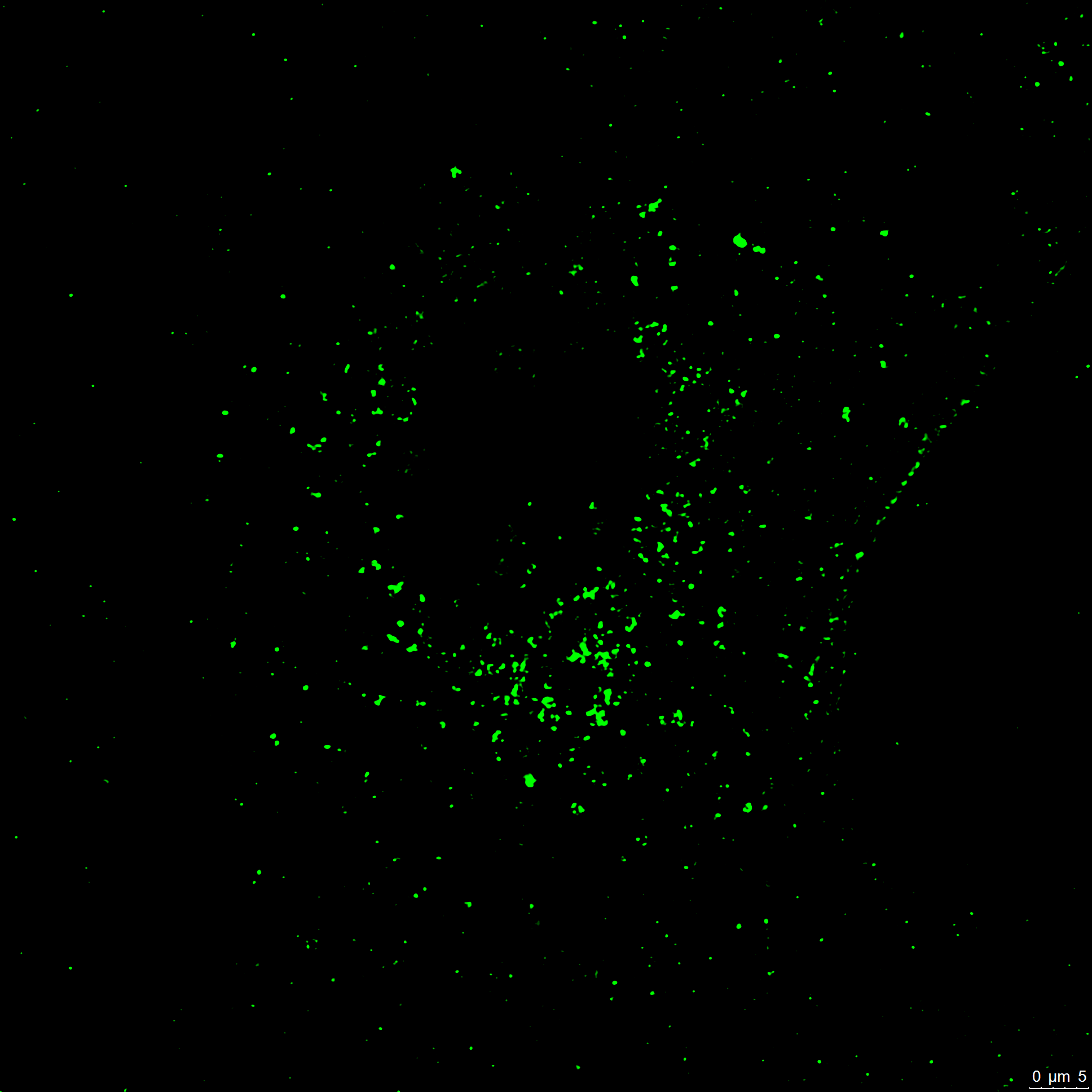

Supplement: Supplementary file 10 — Source data Fig. 3 [file 44318_2025_654_MOESM10_ESM.zip › Figure 3 /3D/3D-1 WT ATP6V1D.tif]

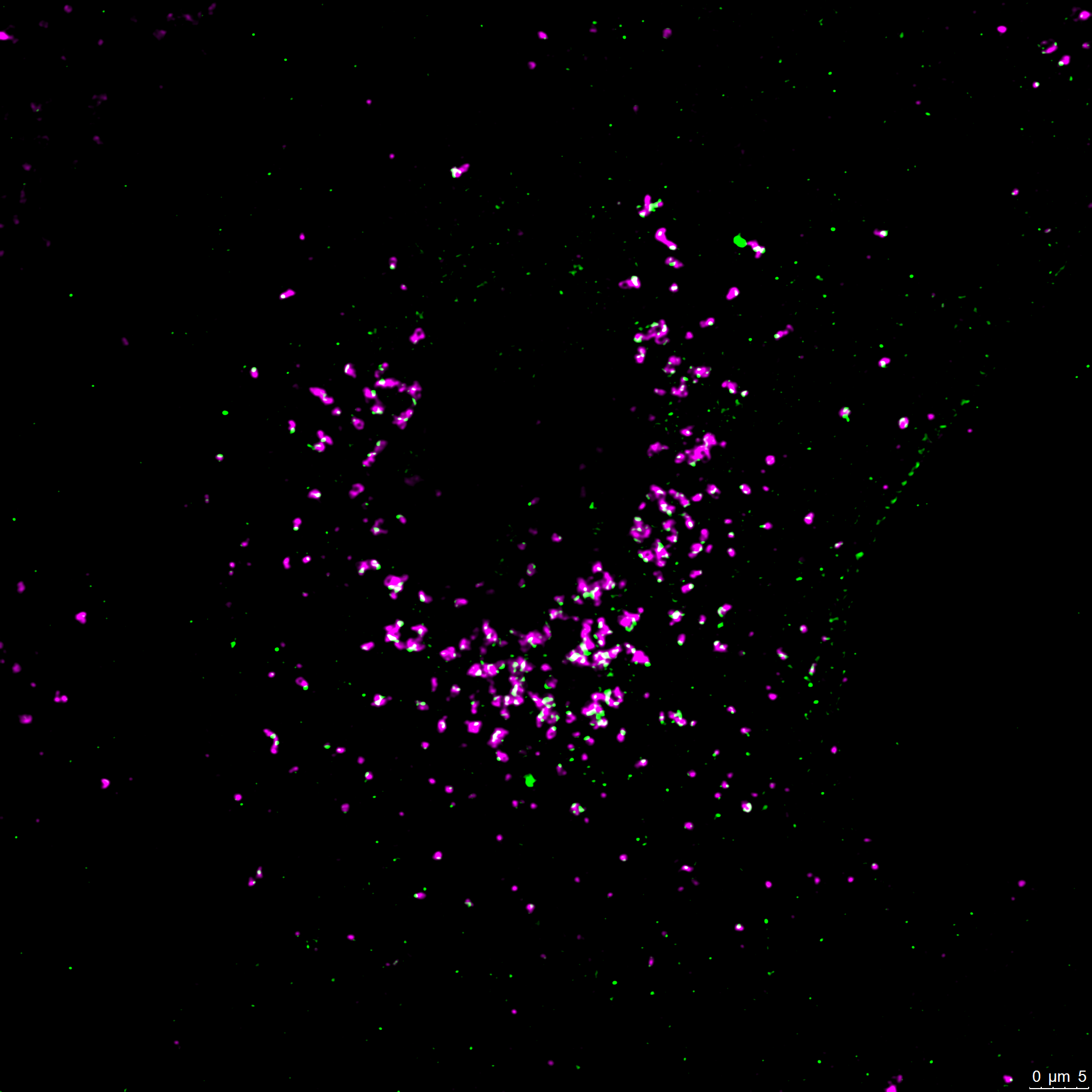

Supplement: Supplementary file 10 — Source data Fig. 3 [file 44318_2025_654_MOESM10_ESM.zip › Figure 3 /3D/3D-1 WT ATP6V1D+LAMP1 merge.tif]

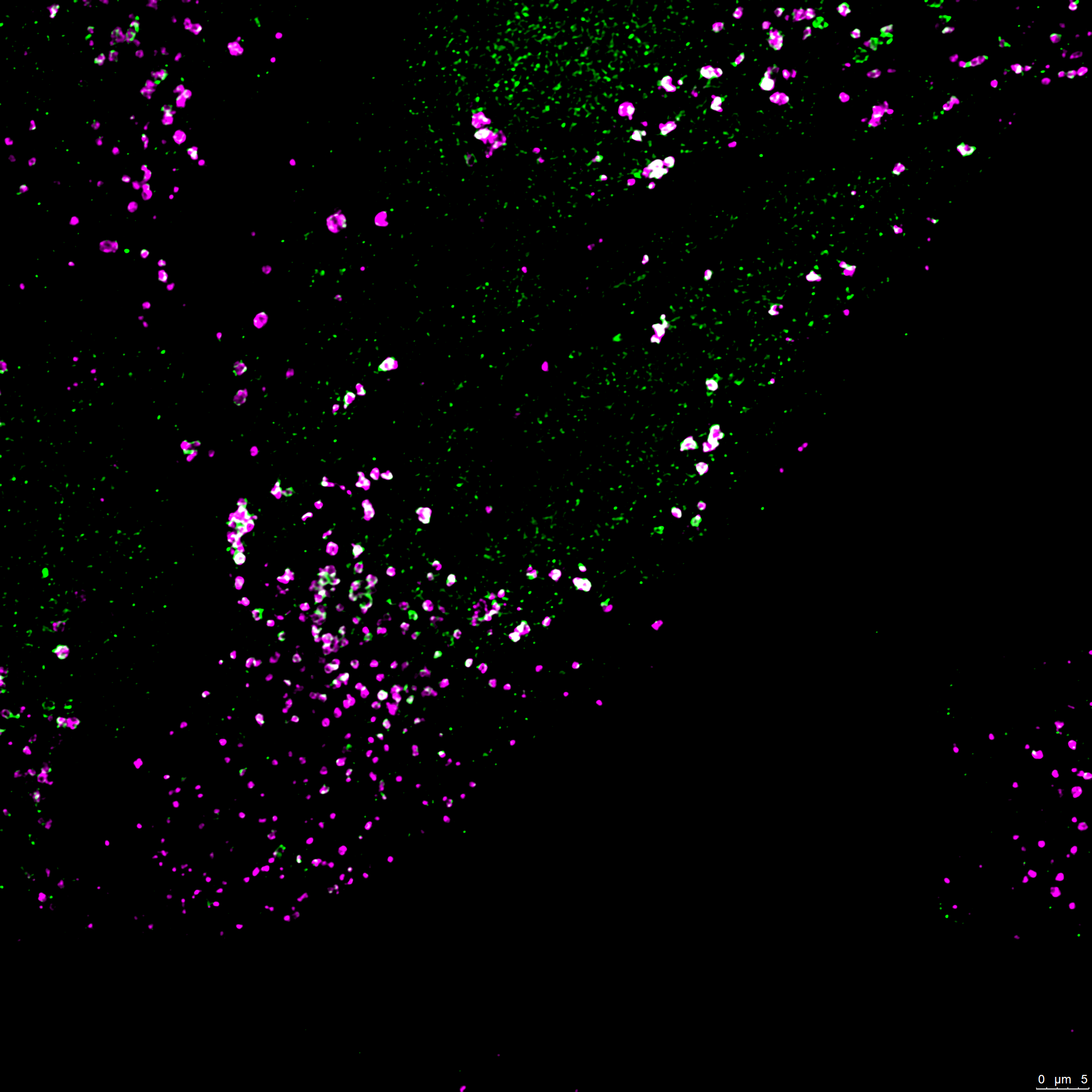

Supplement: Supplementary file 10 — Source data Fig. 3 [file 44318_2025_654_MOESM10_ESM.zip › Figure 3 /3D/3D-2 AREL1-KO ATP6V1D+LAMP1 merge.tif]

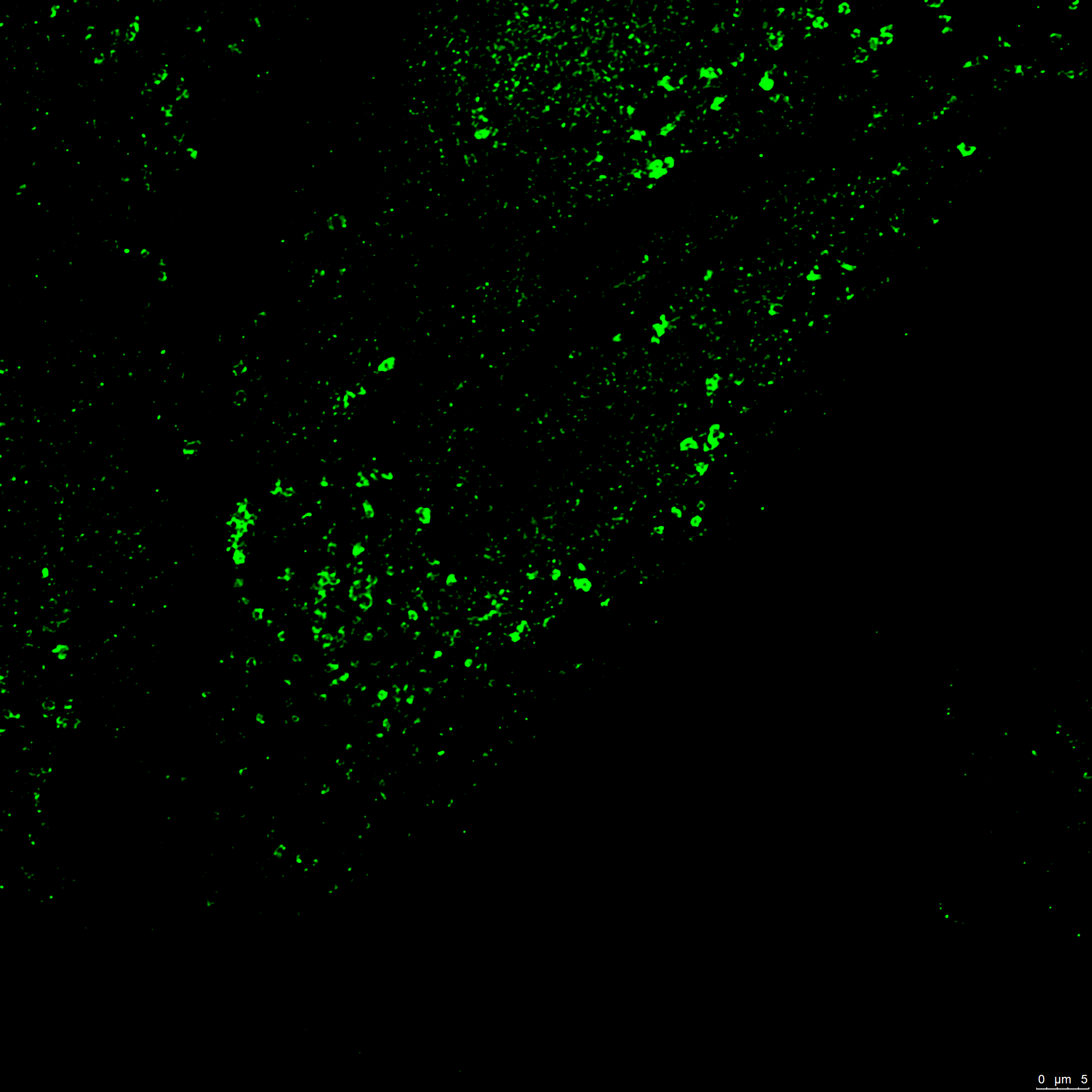

Supplement: Supplementary file 10 — Source data Fig. 3 [file 44318_2025_654_MOESM10_ESM.zip › Figure 3 /3D/3D-2 AREL1-KO ATP6V1D.tif]

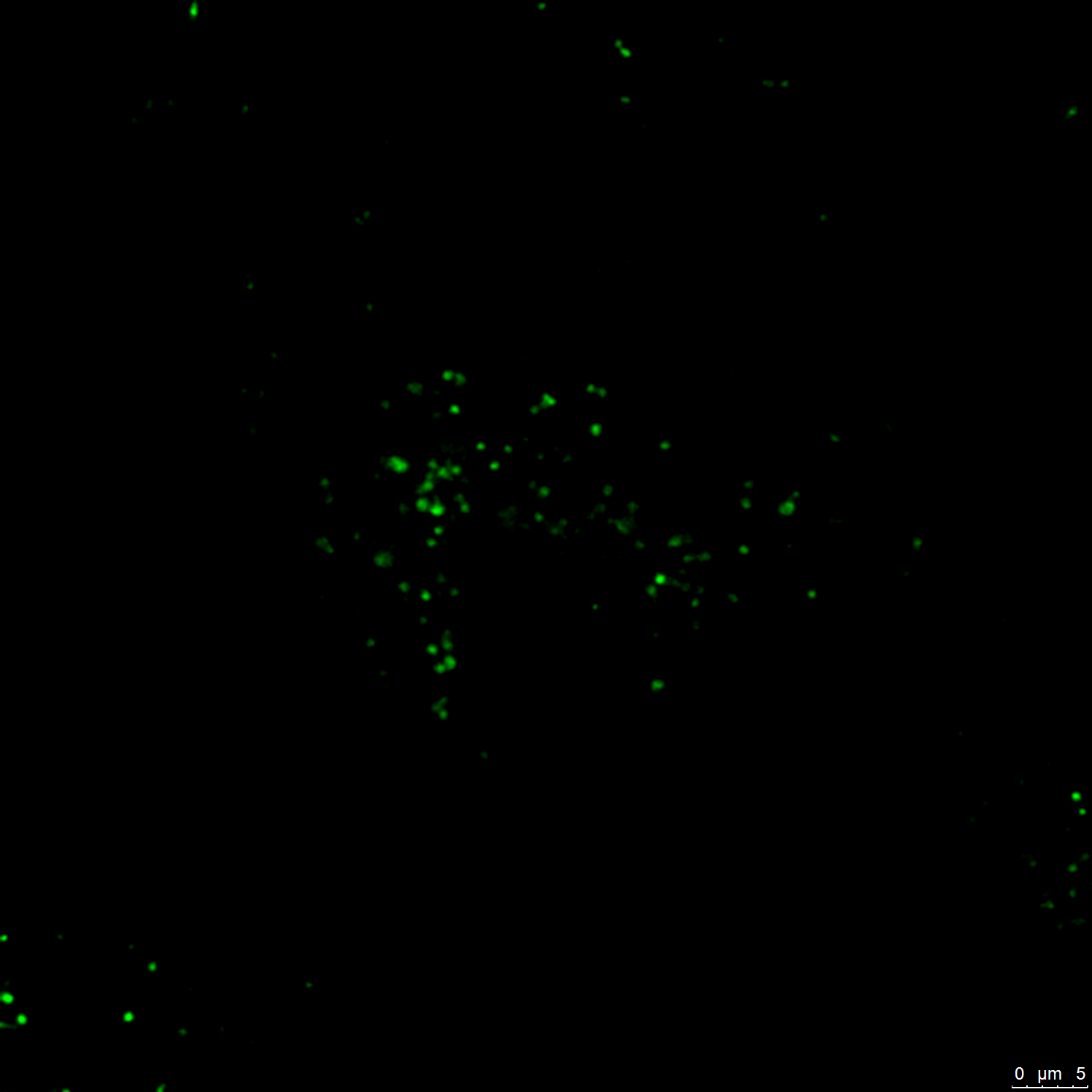

Supplement: Supplementary file 10 — Source data Fig. 3 [file 44318_2025_654_MOESM10_ESM.zip › Figure 3 /3J/3J-2 AREL1-KO SiR-lysosome.tif]

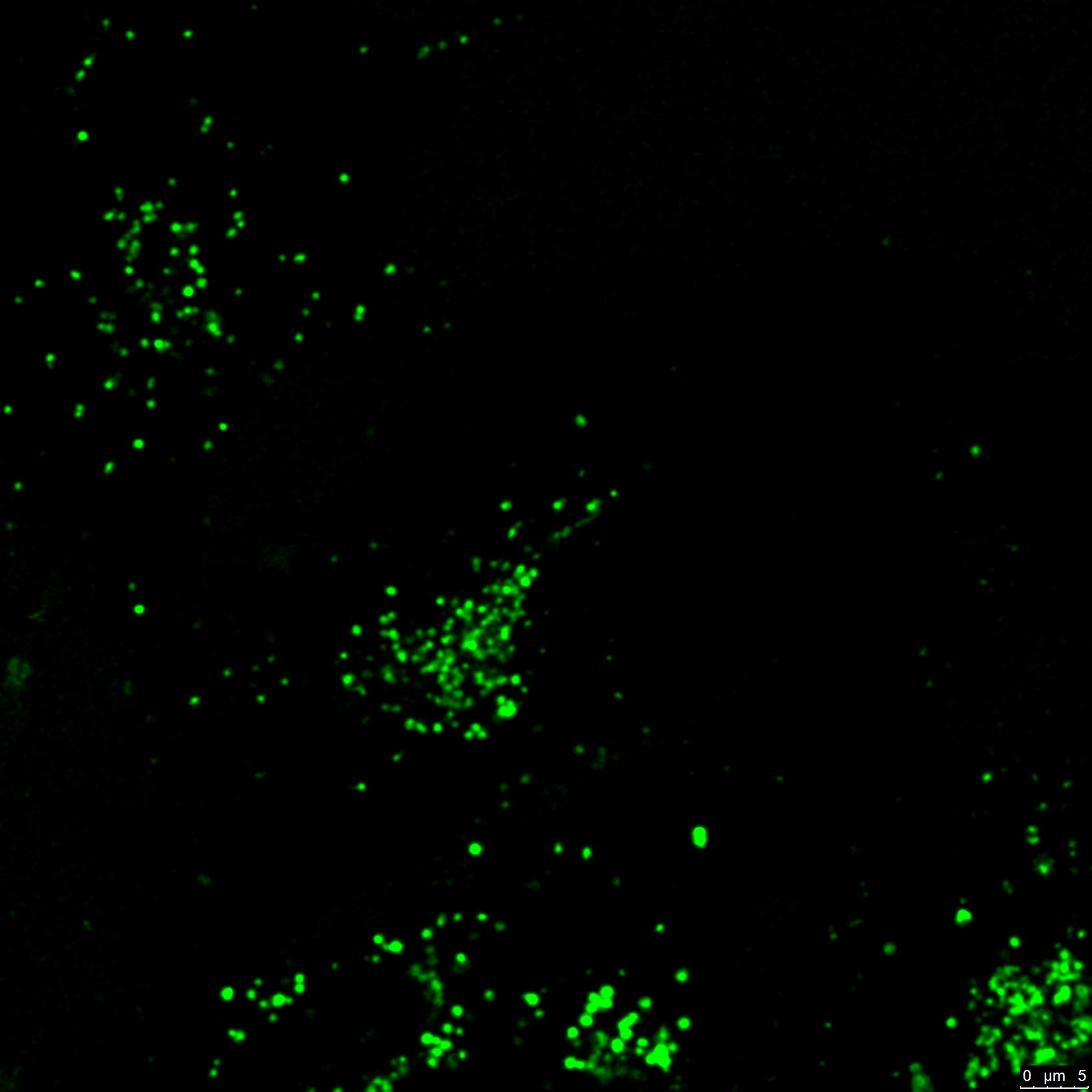

Supplement: Supplementary file 10 — Source data Fig. 3 [file 44318_2025_654_MOESM10_ESM.zip › Figure 3 /3J/3J-1 WT SiR-lysosome.tif]

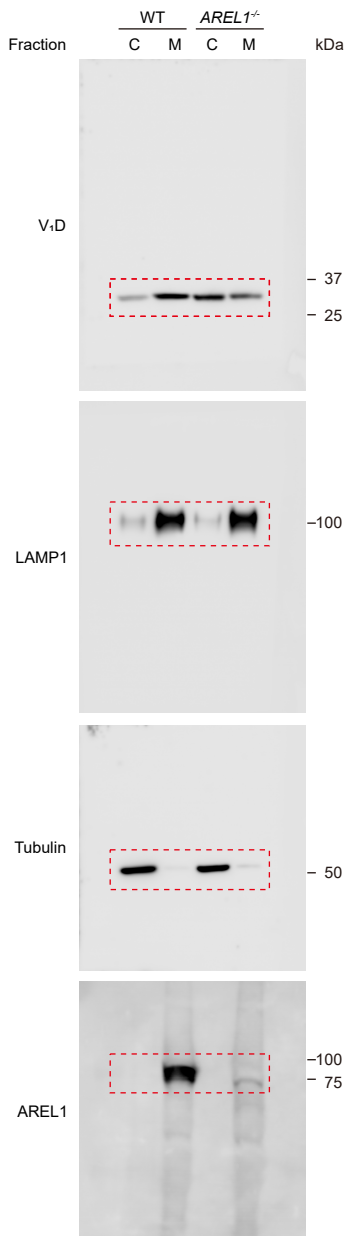

Supplement: Supplementary file 10 — Source data Fig. 3 [file 44318_2025_654_MOESM10_ESM.zip › Figure 3 /3G/3G.pdf]

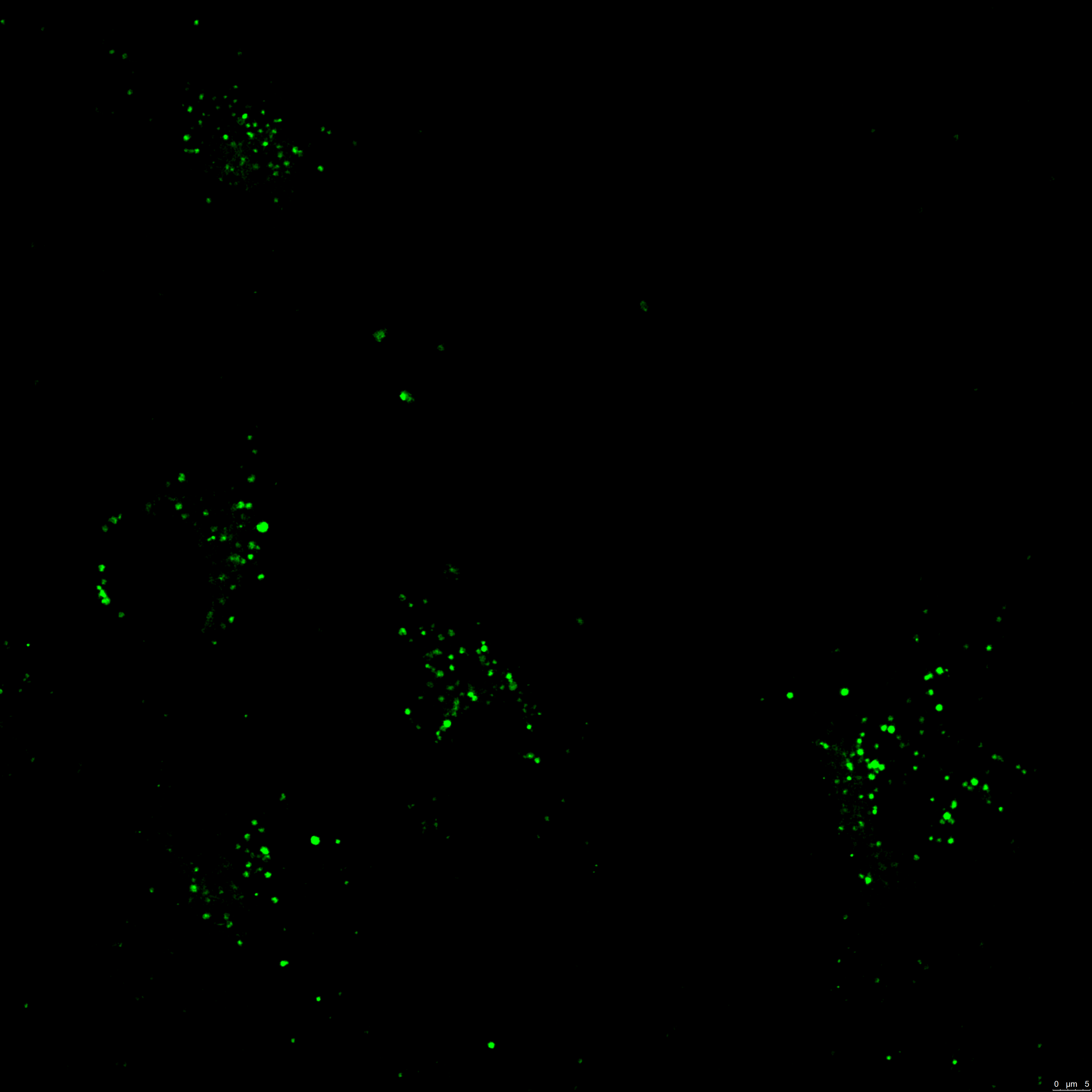

Supplement: Supplementary file 10 — Source data Fig. 3 [file 44318_2025_654_MOESM10_ESM.zip › Figure 3 /3I/3I-2 AREL1-KO DQ-OVA.tif]

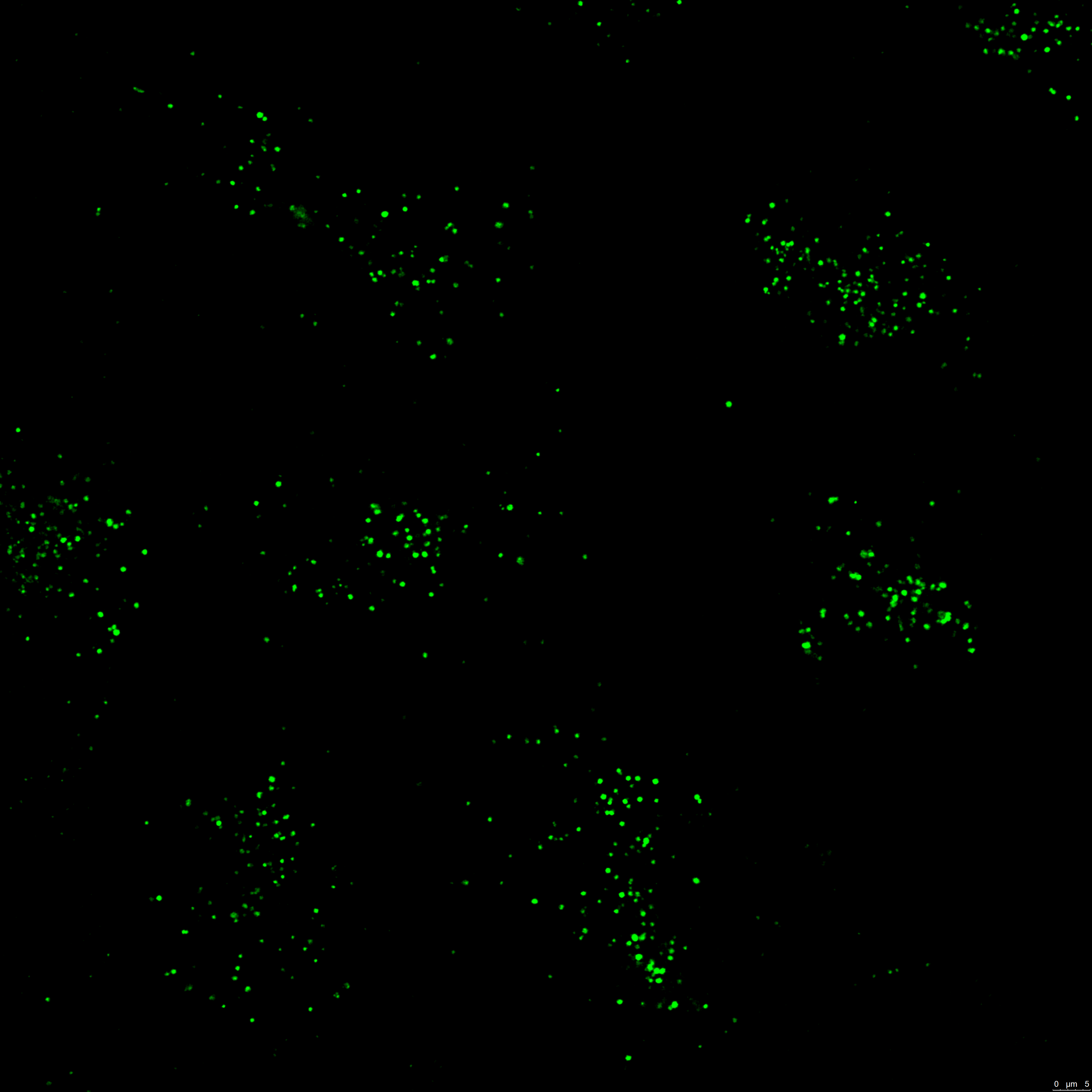

Supplement: Supplementary file 10 — Source data Fig. 3 [file 44318_2025_654_MOESM10_ESM.zip › Figure 3 /3I/3I-1 WT DQ-OVA.tif]

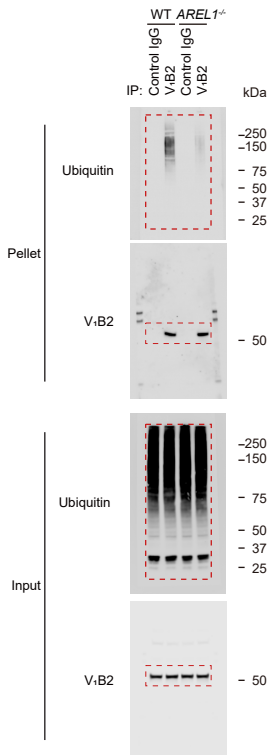

Supplement: Supplementary file 11 — Source data Fig. 4 [file 44318_2025_654_MOESM11_ESM.zip › Figure 4 /4B/4B.pdf]

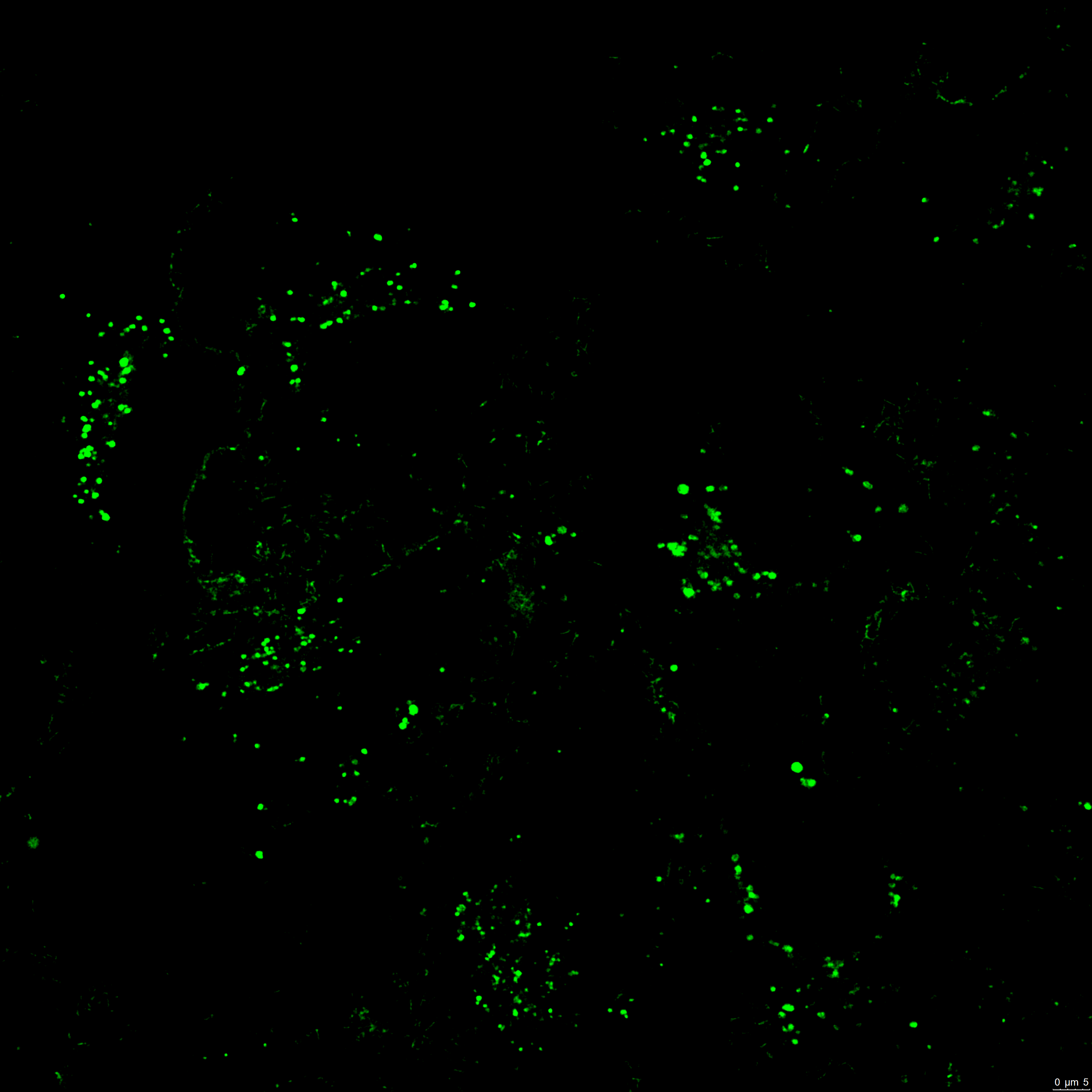

Supplement: Supplementary file 11 — Source data Fig. 4 [file 44318_2025_654_MOESM11_ESM.zip › Figure 4 /4L/4L-2-shZRANB1-lysosensor green.tif]

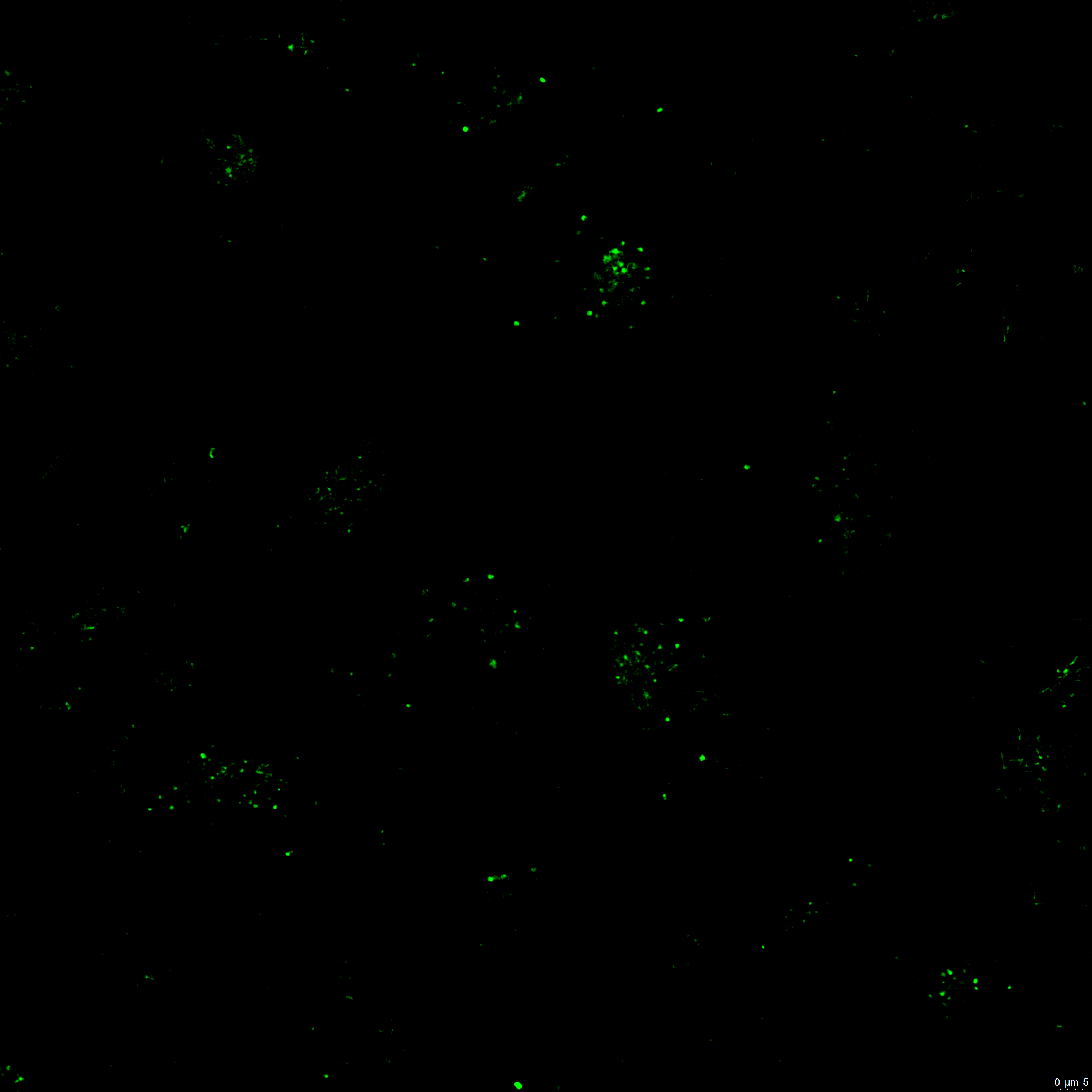

Supplement: Supplementary file 11 — Source data Fig. 4 [file 44318_2025_654_MOESM11_ESM.zip › Figure 4 /4L/4L-1-shNC-lysosensor green.tif]

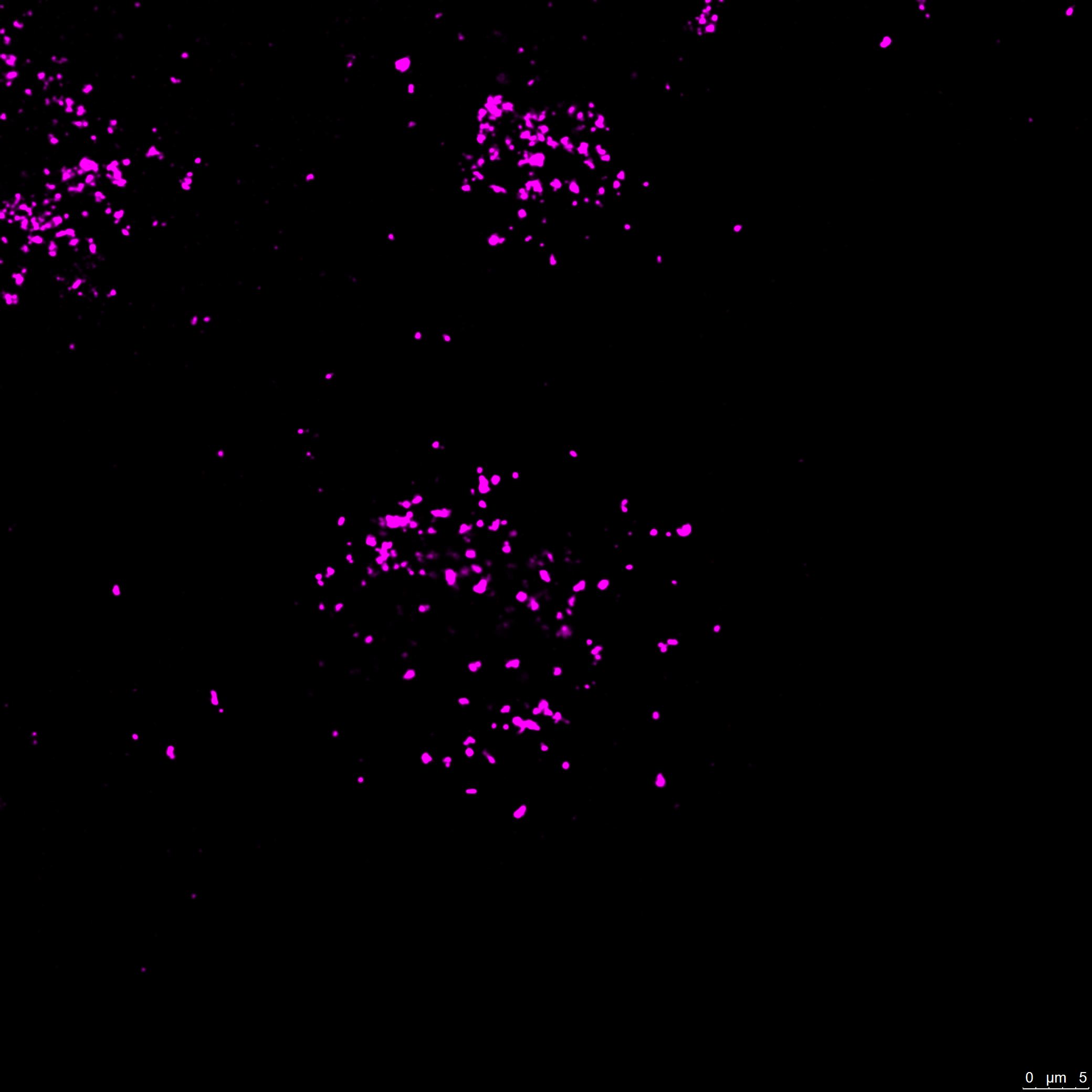

Supplement: Supplementary file 11 — Source data Fig. 4 [file 44318_2025_654_MOESM11_ESM.zip › Figure 4 /4J/4J-1-shNC-LAMP1.tif]
