# Supplementary figures and images for: E3 ligase AREL1 controls perinuclear localization of lysosomes and supports Purkinje cell survival (part 2 of 4)
Source: EMBO J. 2025 Dec 2;45(3):655–91. doi: 10.1038/s44318-025-00654-3 (PMC12864862; doi:10.1038/s44318-025-00654-3)

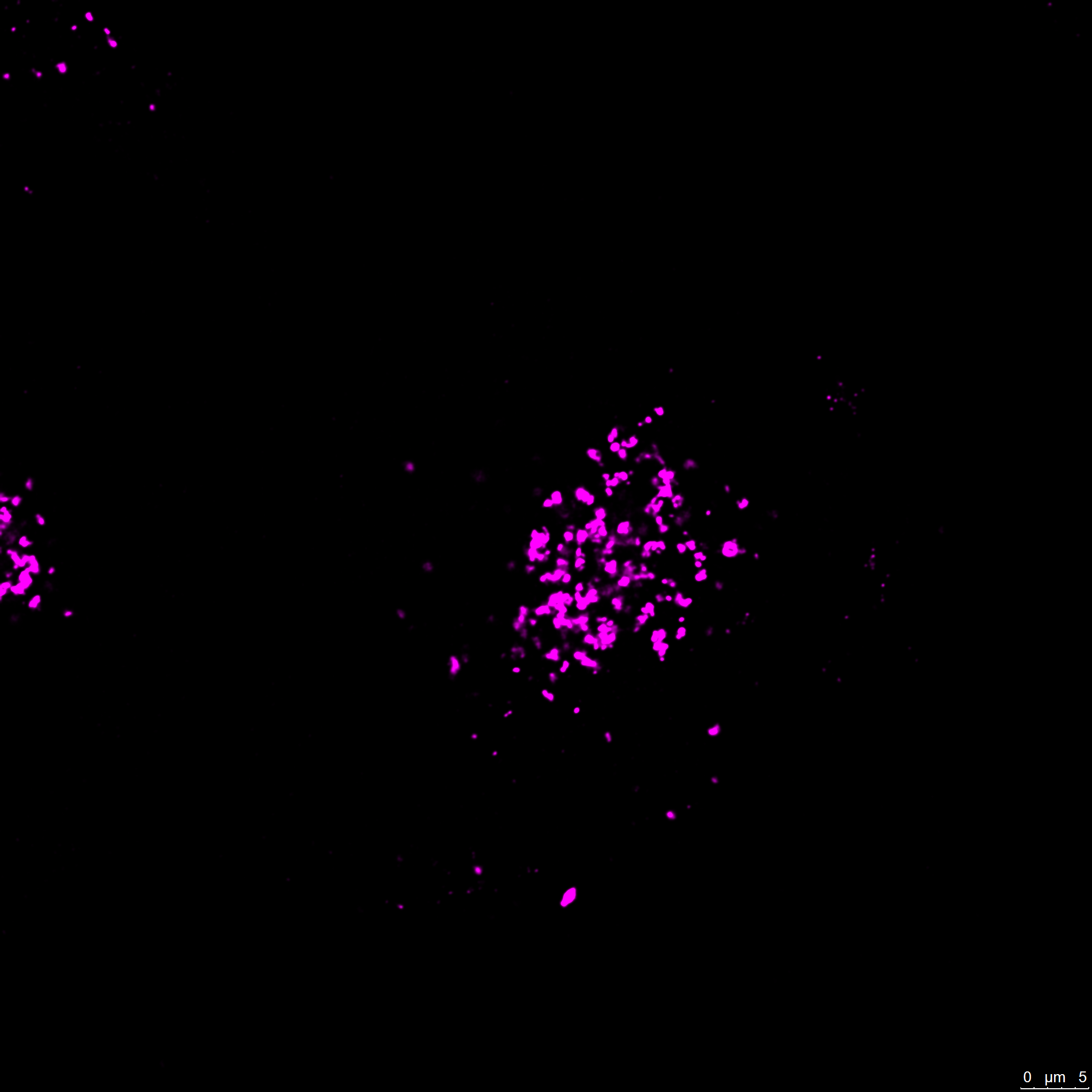

Supplement: Supplementary file 11 — Source data Fig. 4 [file 44318_2025_654_MOESM11_ESM.zip › Figure 4 /4J/4J-2-shZRANB1-LAMP1.tif]

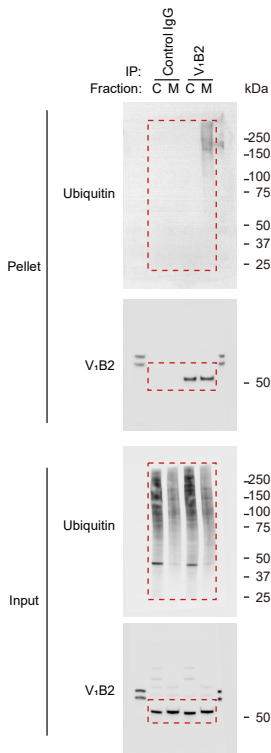

Supplement: Supplementary file 11 — Source data Fig. 4 [file 44318_2025_654_MOESM11_ESM.zip › Figure 4 /4C/4C.pdf]

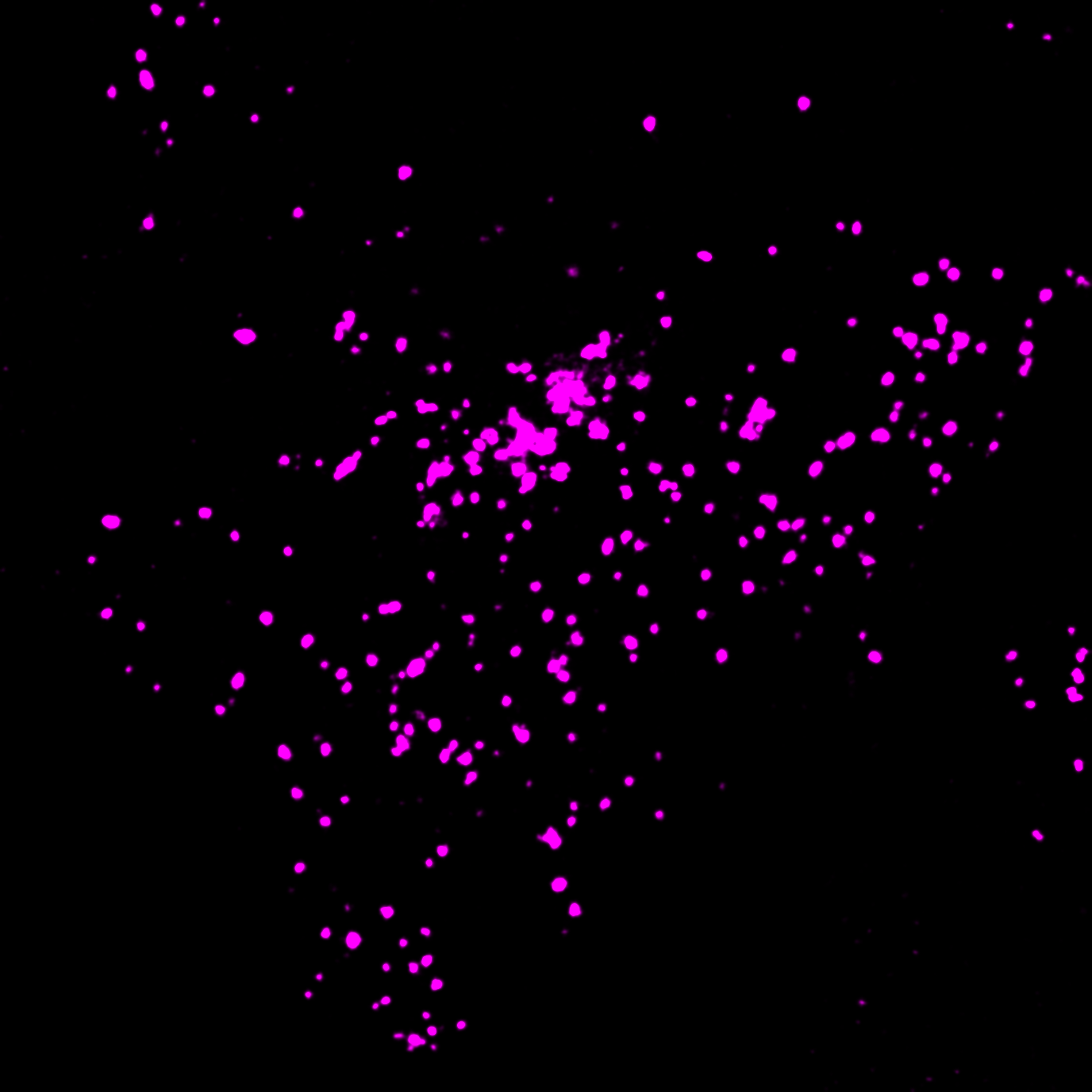

Supplement: Supplementary file 11 — Source data Fig. 4 [file 44318_2025_654_MOESM11_ESM.zip › Figure 4 /4D/4D-2 shATP6V1B2-LAMP1.tif]

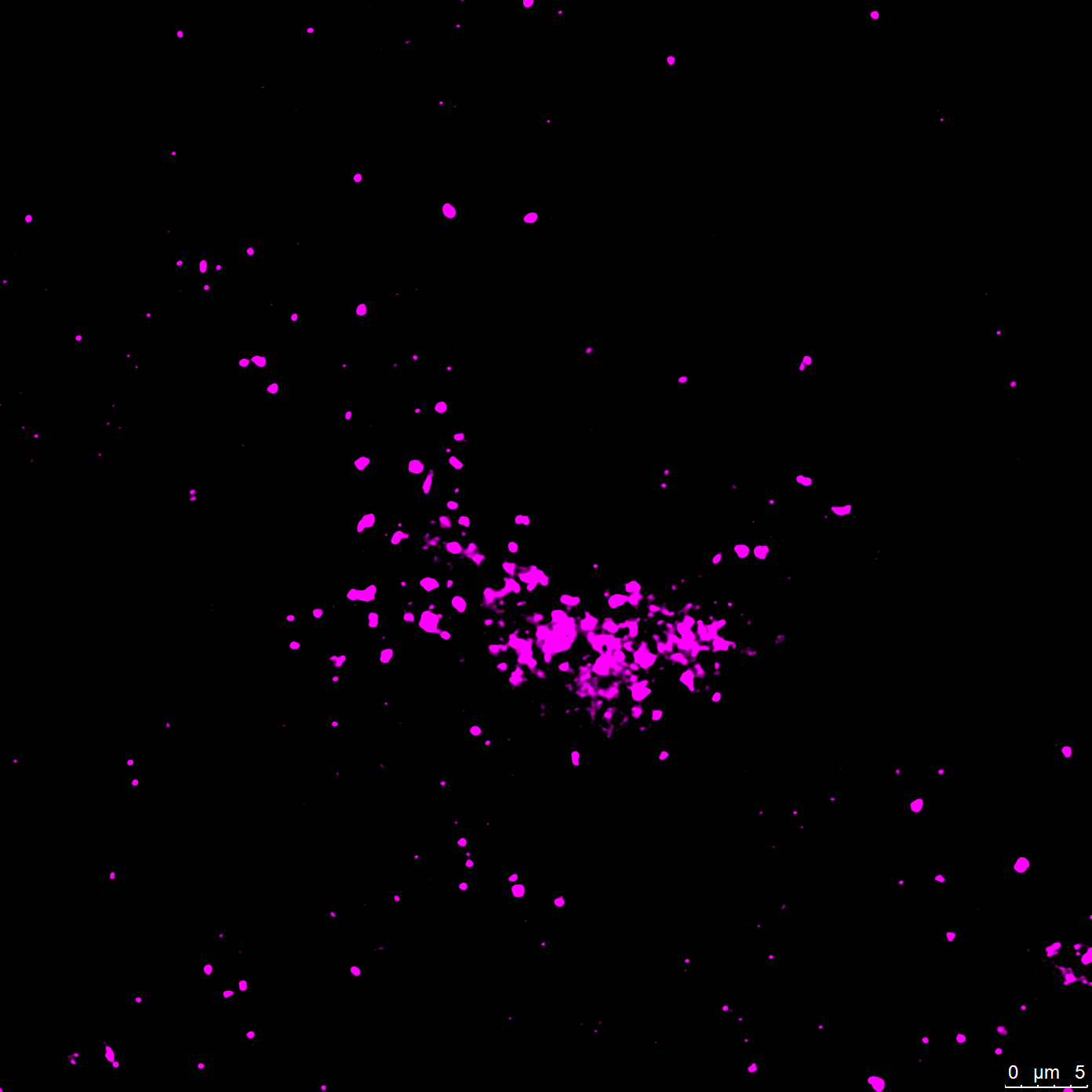

Supplement: Supplementary file 11 — Source data Fig. 4 [file 44318_2025_654_MOESM11_ESM.zip › Figure 4 /4D/4D-1 shNC-LAMP1.tif]

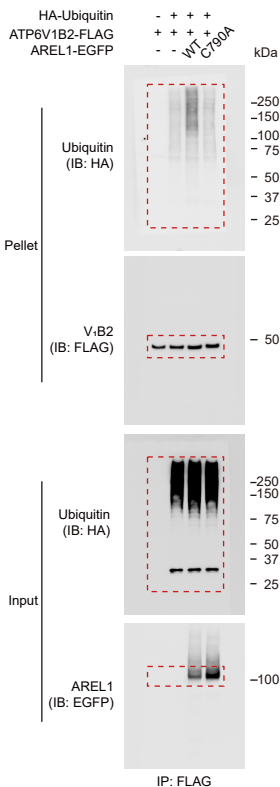

Supplement: Supplementary file 11 — Source data Fig. 4 [file 44318_2025_654_MOESM11_ESM.zip › Figure 4 /4A/4A.pdf]

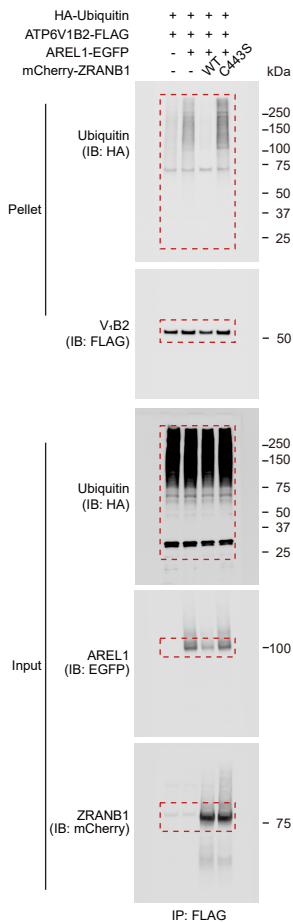

Supplement: Supplementary file 11 — Source data Fig. 4 [file 44318_2025_654_MOESM11_ESM.zip › Figure 4 /4F/4F.pdf]

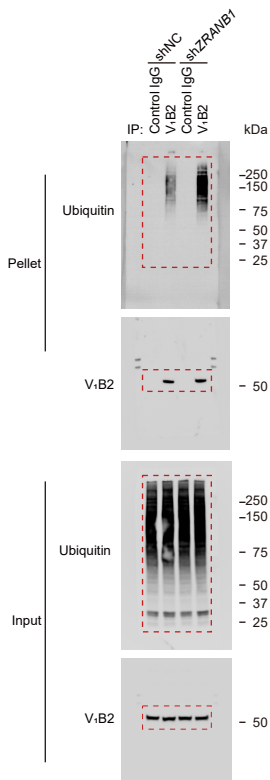

Supplement: Supplementary file 11 — Source data Fig. 4 [file 44318_2025_654_MOESM11_ESM.zip › Figure 4 /4I/4I.pdf]

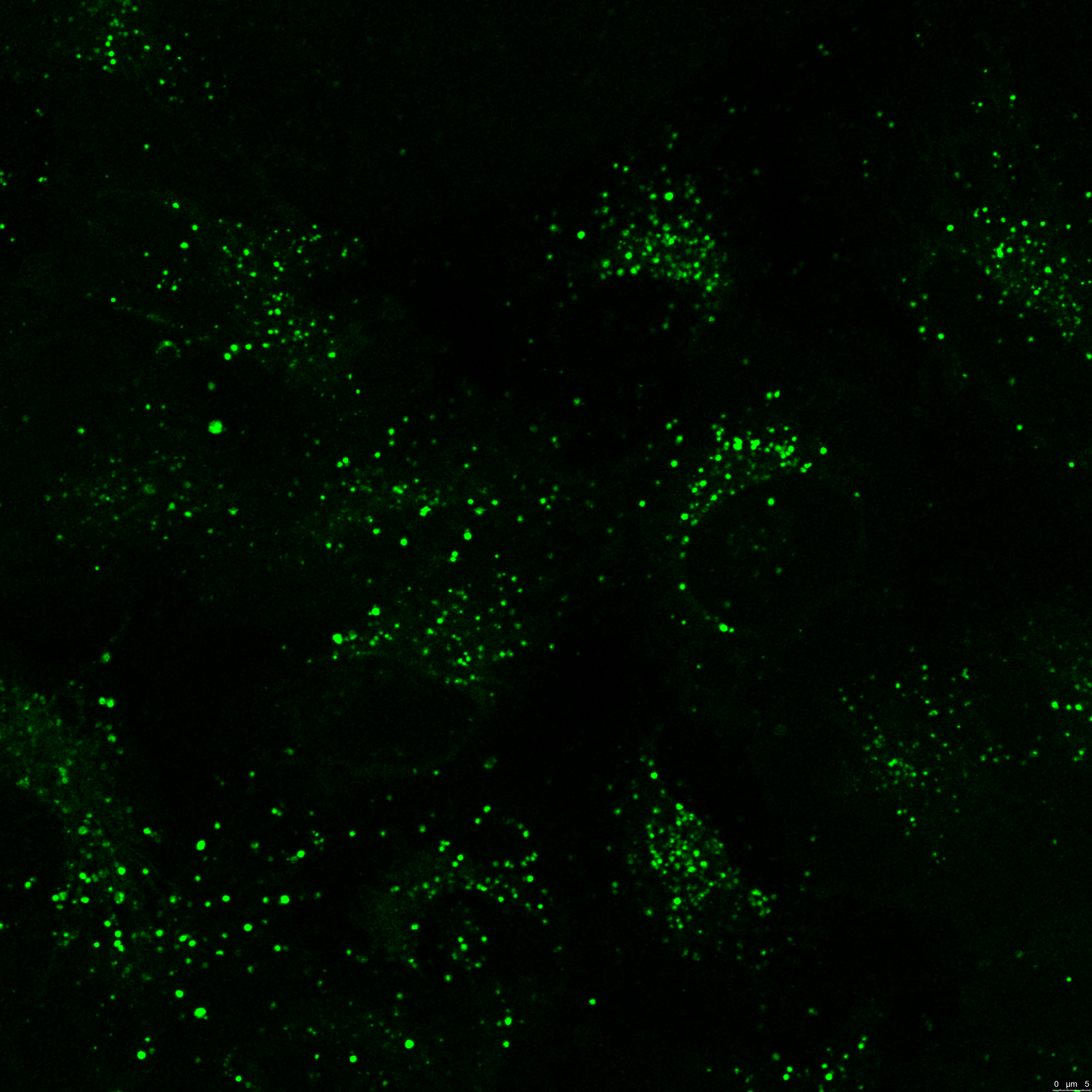

Supplement: Supplementary file 11 — Source data Fig. 4 [file 44318_2025_654_MOESM11_ESM.zip › Figure 4 /4N/4N-2-shZRANB1_DQ-OVA.tif]

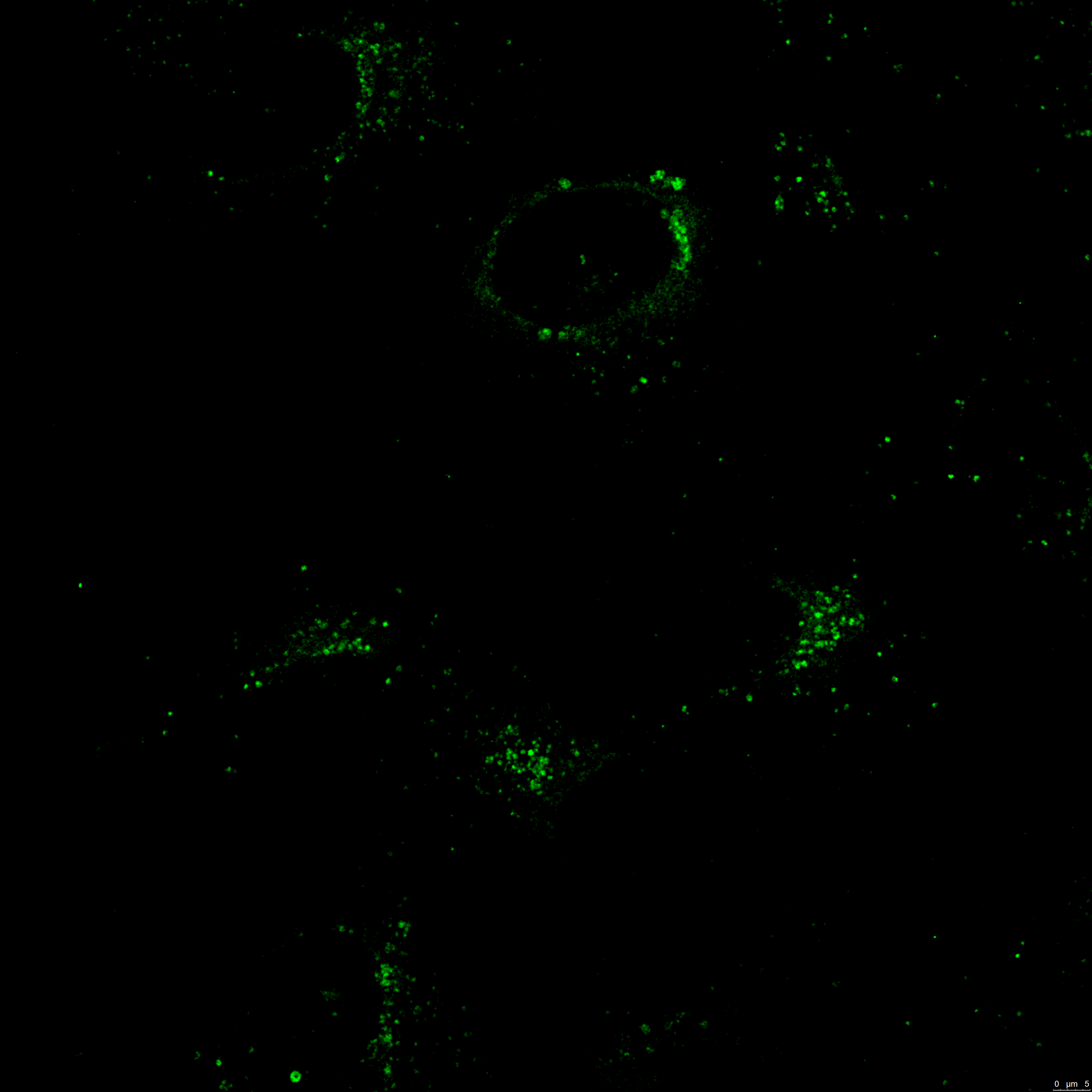

Supplement: Supplementary file 11 — Source data Fig. 4 [file 44318_2025_654_MOESM11_ESM.zip › Figure 4 /4N/4N-1-shNC_DQ-OVA.tif]

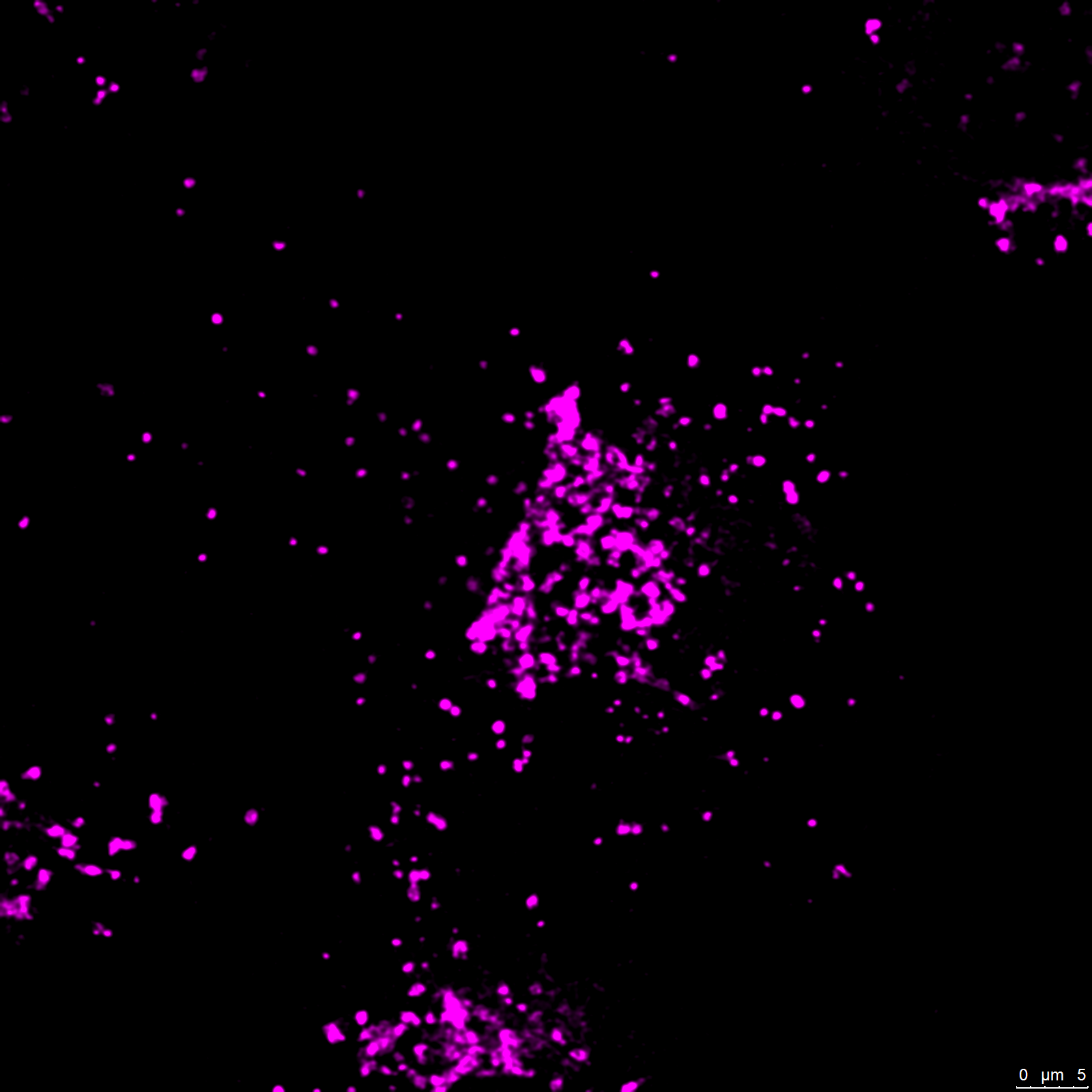

Supplement: Supplementary file 11 — Source data Fig. 4 [file 44318_2025_654_MOESM11_ESM.zip › Figure 4 /4G/4G-3-LAMP1.tif]

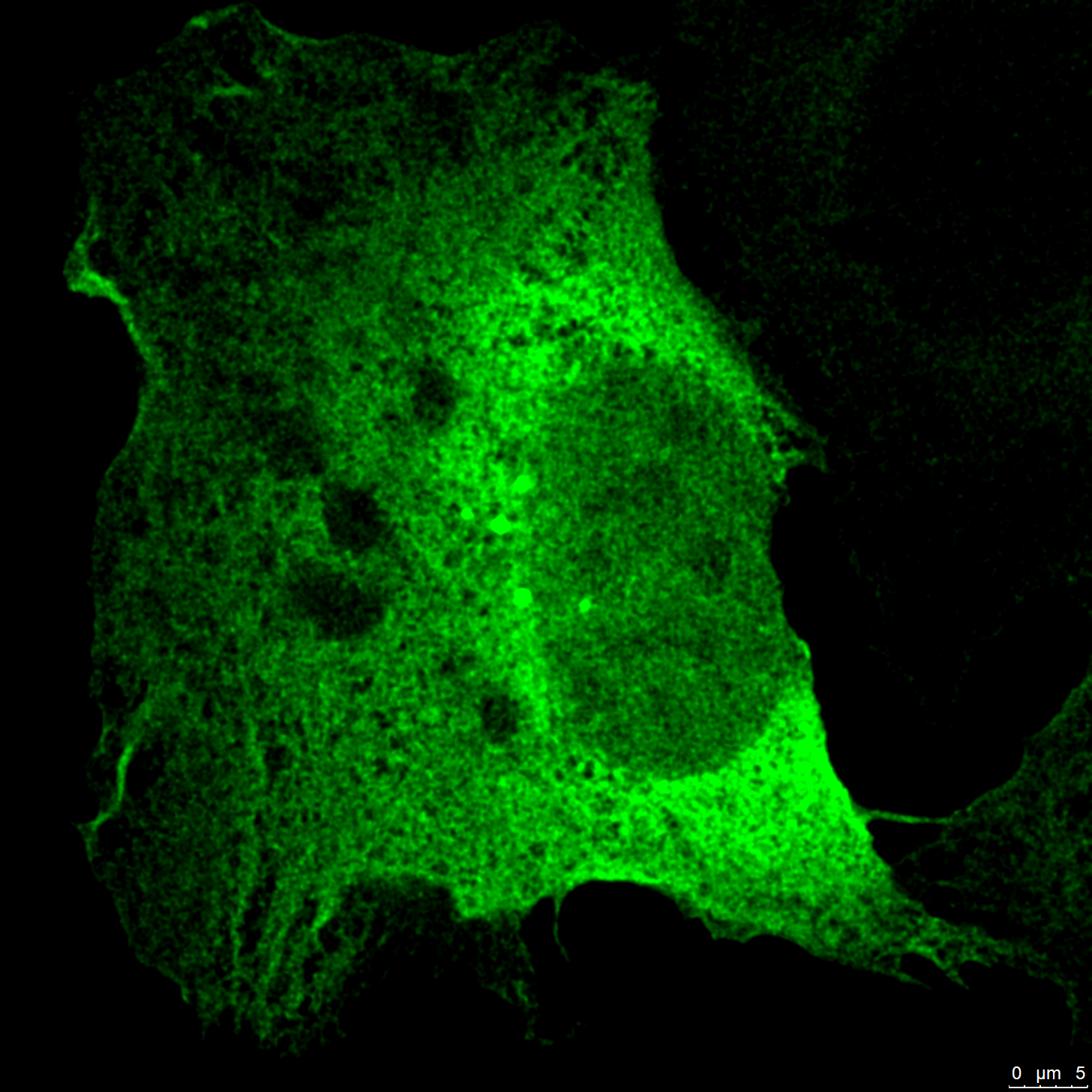

Supplement: Supplementary file 11 — Source data Fig. 4 [file 44318_2025_654_MOESM11_ESM.zip › Figure 4 /4G/4G-2-mCherry-ZRANB1(WT).tif]

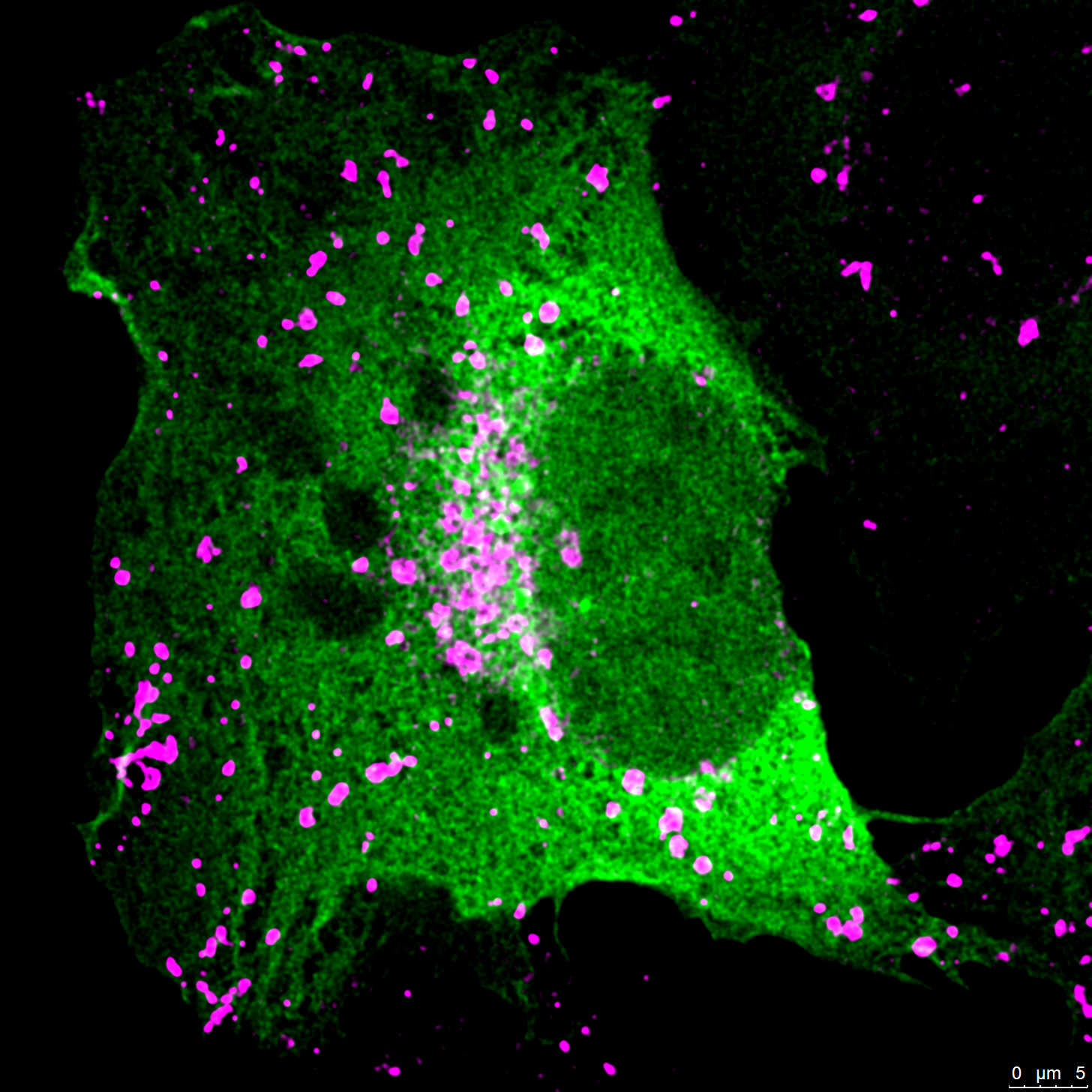

Supplement: Supplementary file 11 — Source data Fig. 4 [file 44318_2025_654_MOESM11_ESM.zip › Figure 4 /4G/4G-2-mCherry-ZRANB1(WT)-merge.tif]

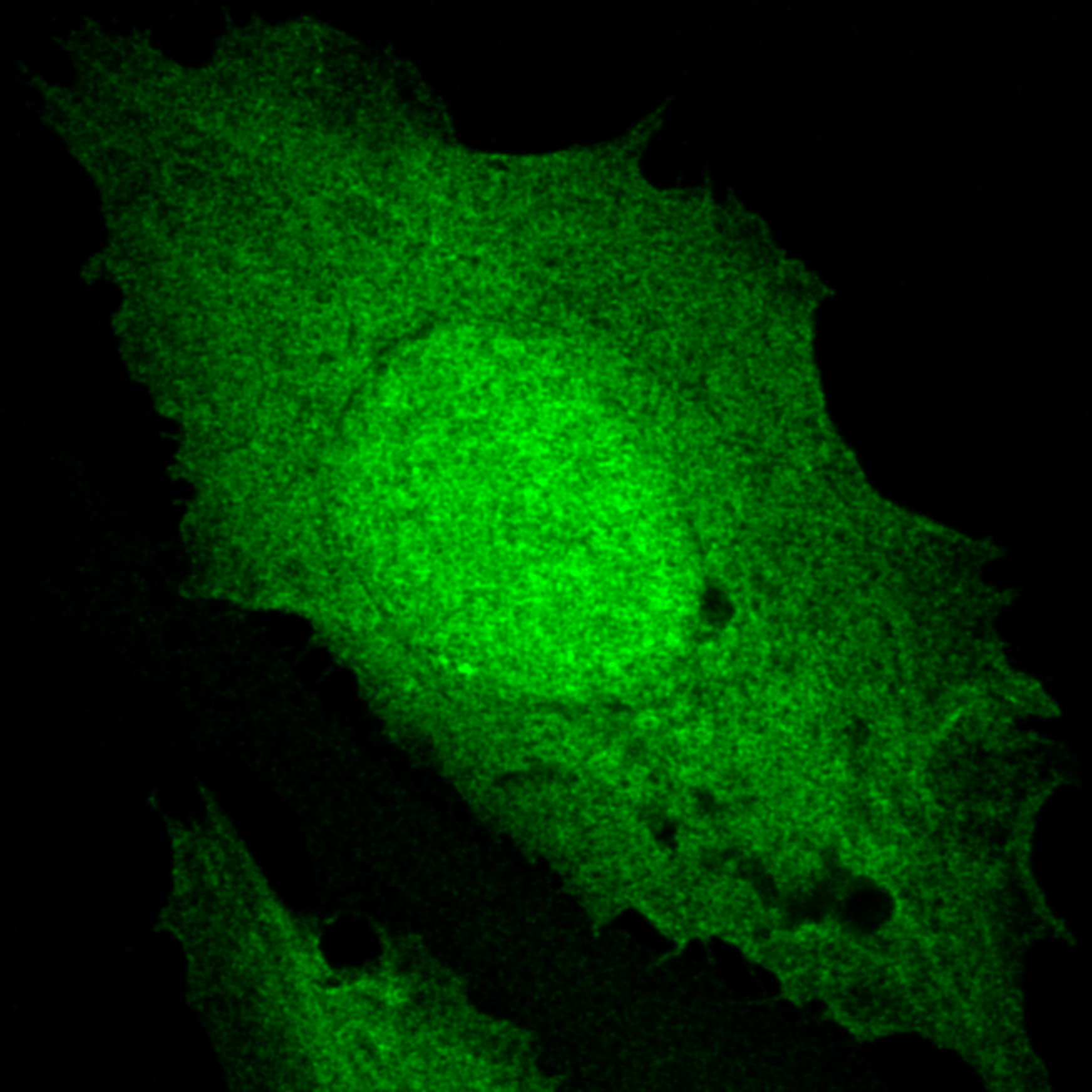

Supplement: Supplementary file 11 — Source data Fig. 4 [file 44318_2025_654_MOESM11_ESM.zip › Figure 4 /4G/4G-1-mCherry.tif]

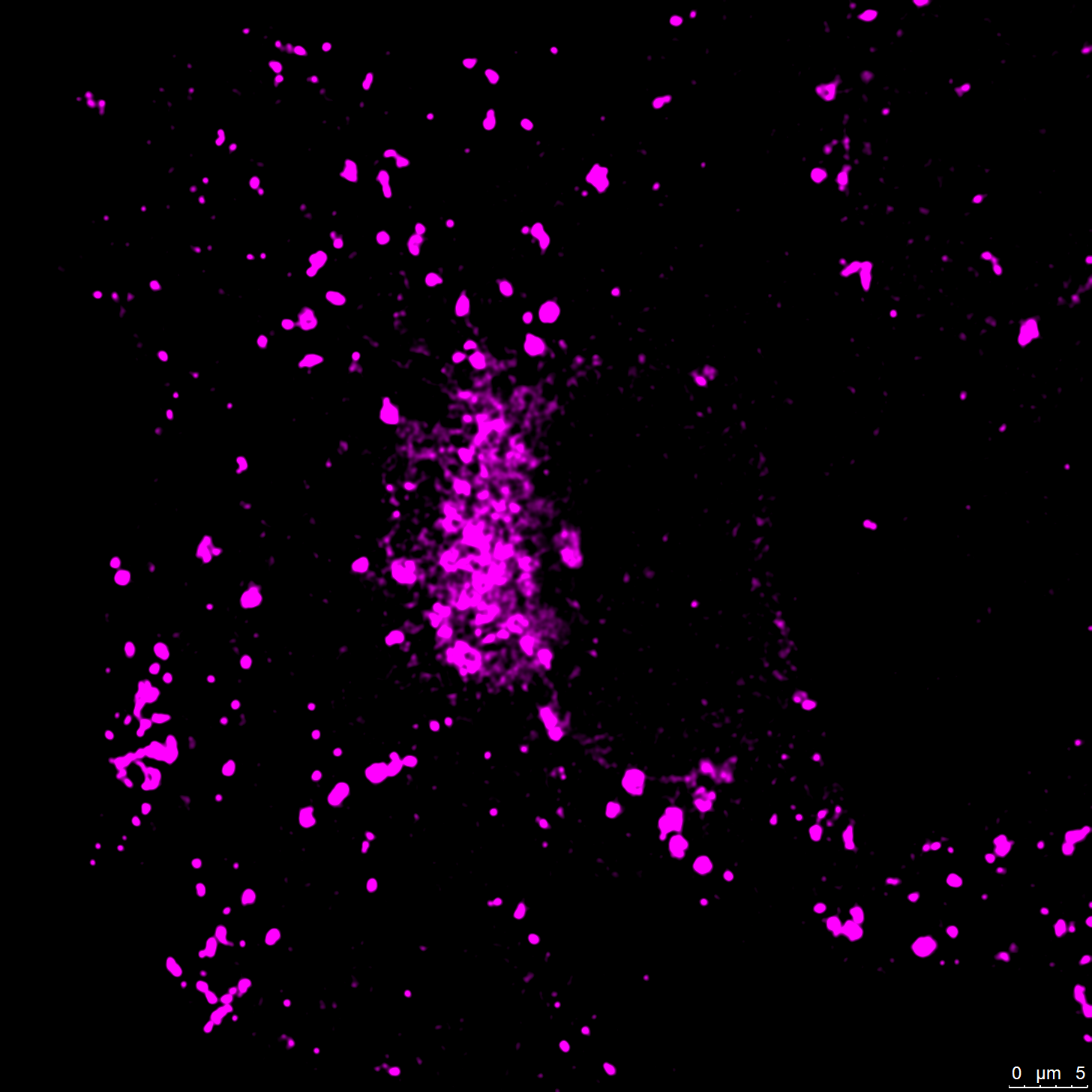

Supplement: Supplementary file 11 — Source data Fig. 4 [file 44318_2025_654_MOESM11_ESM.zip › Figure 4 /4G/4G-2-LAMP1.tif]

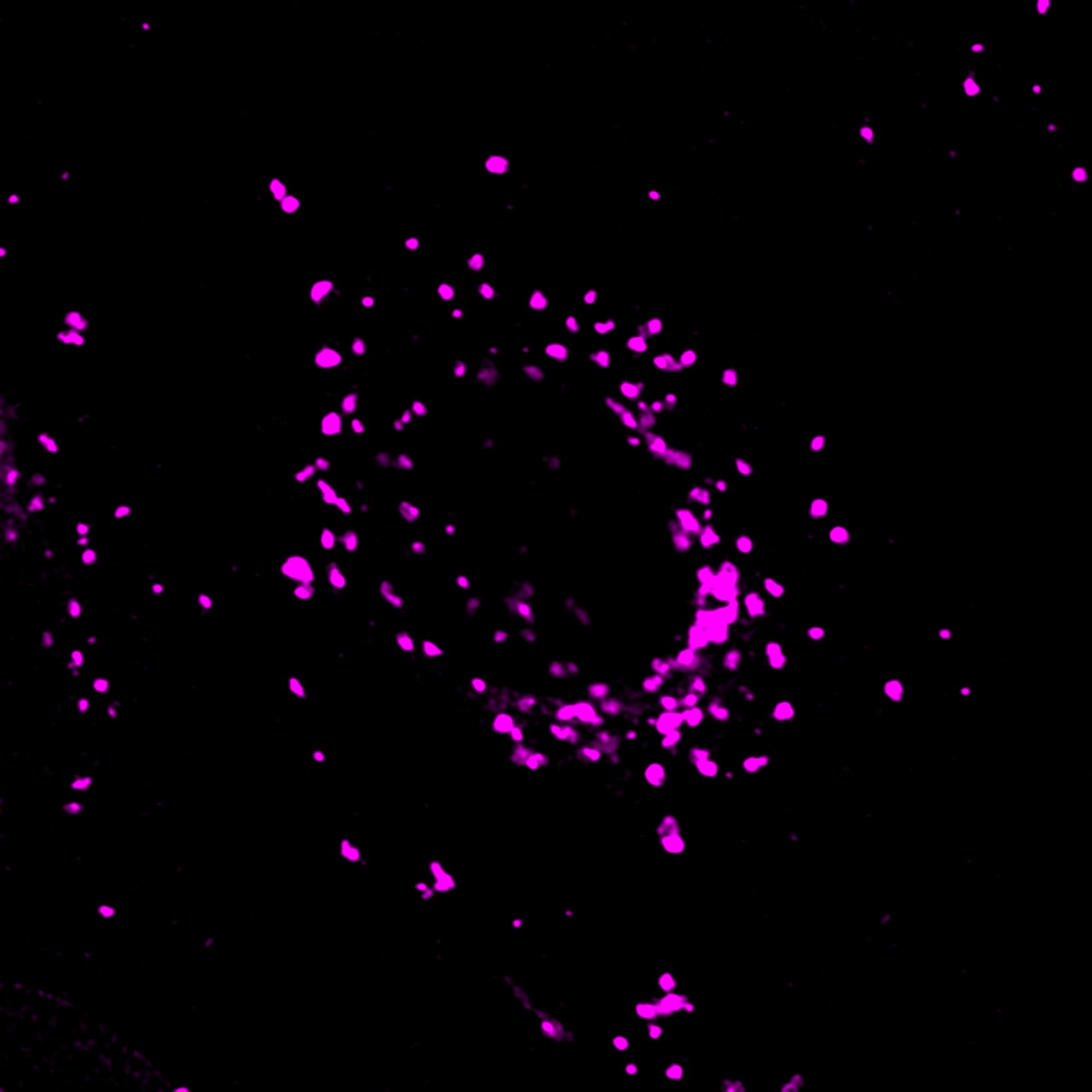

Supplement: Supplementary file 11 — Source data Fig. 4 [file 44318_2025_654_MOESM11_ESM.zip › Figure 4 /4G/4G-1-LAMP1.tif]

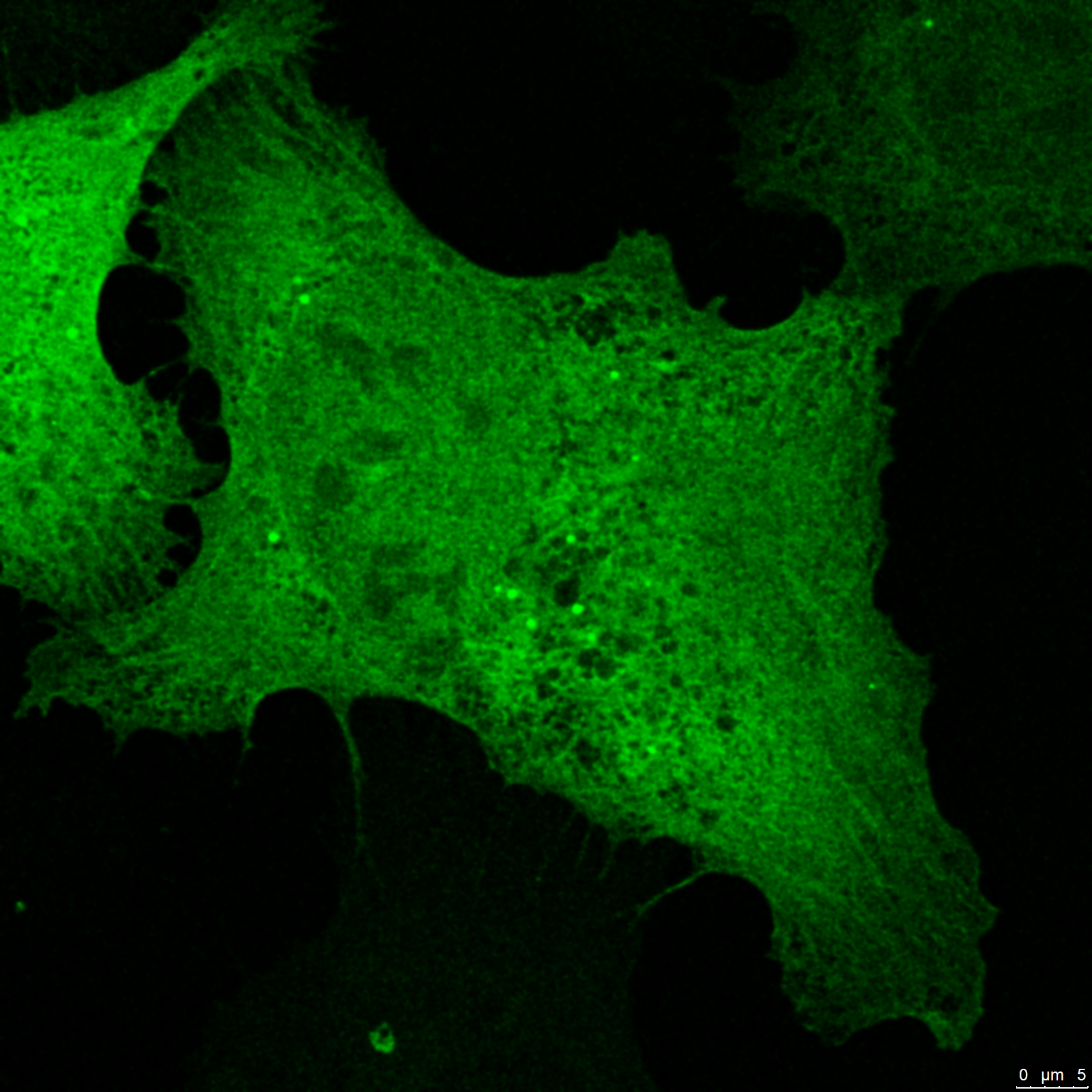

Supplement: Supplementary file 11 — Source data Fig. 4 [file 44318_2025_654_MOESM11_ESM.zip › Figure 4 /4G/4G-3-mCherry-ZRANB1(C443S).tif]

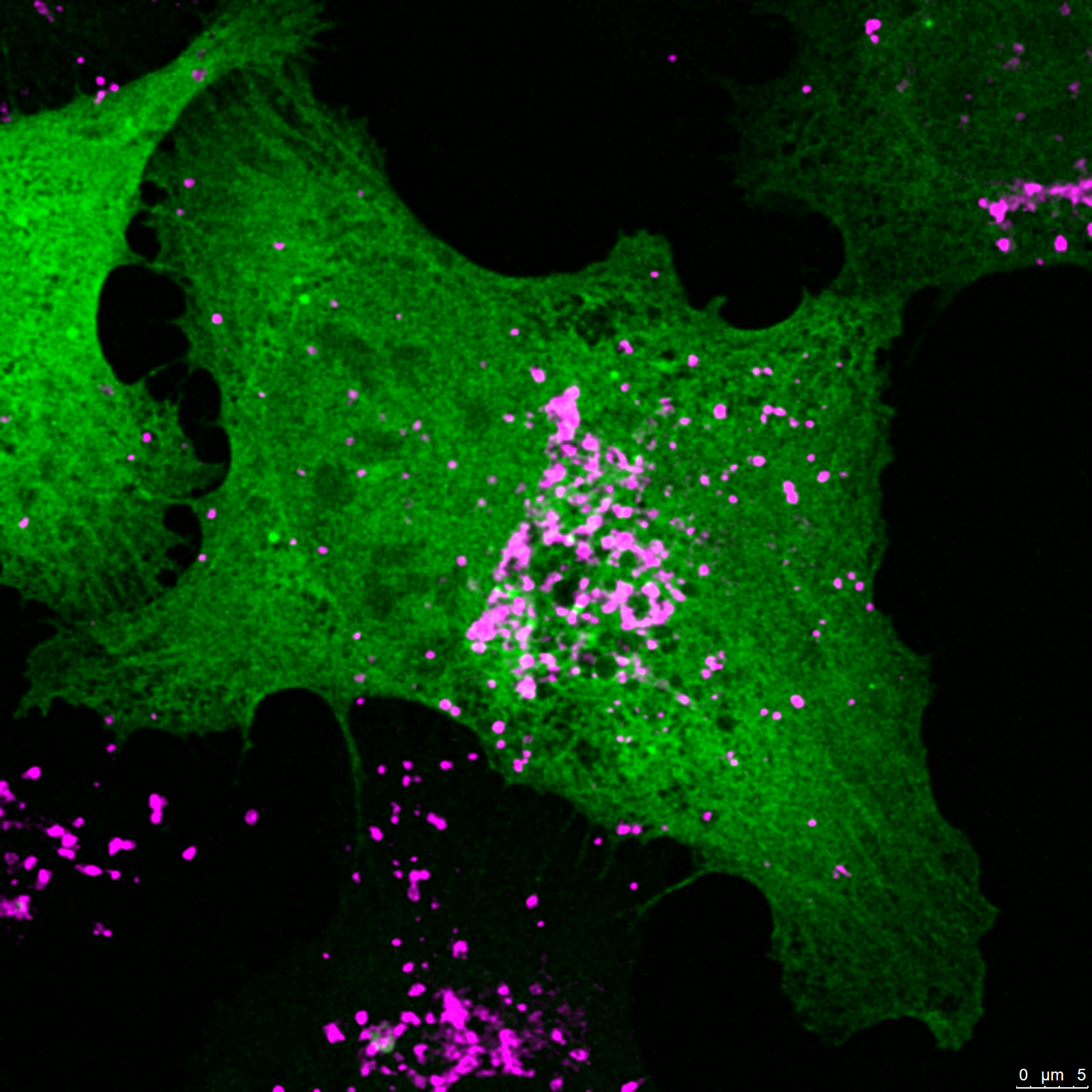

Supplement: Supplementary file 11 — Source data Fig. 4 [file 44318_2025_654_MOESM11_ESM.zip › Figure 4 /4G/4G-3-mCherry-ZRANB1(C443S)-merge.tif]

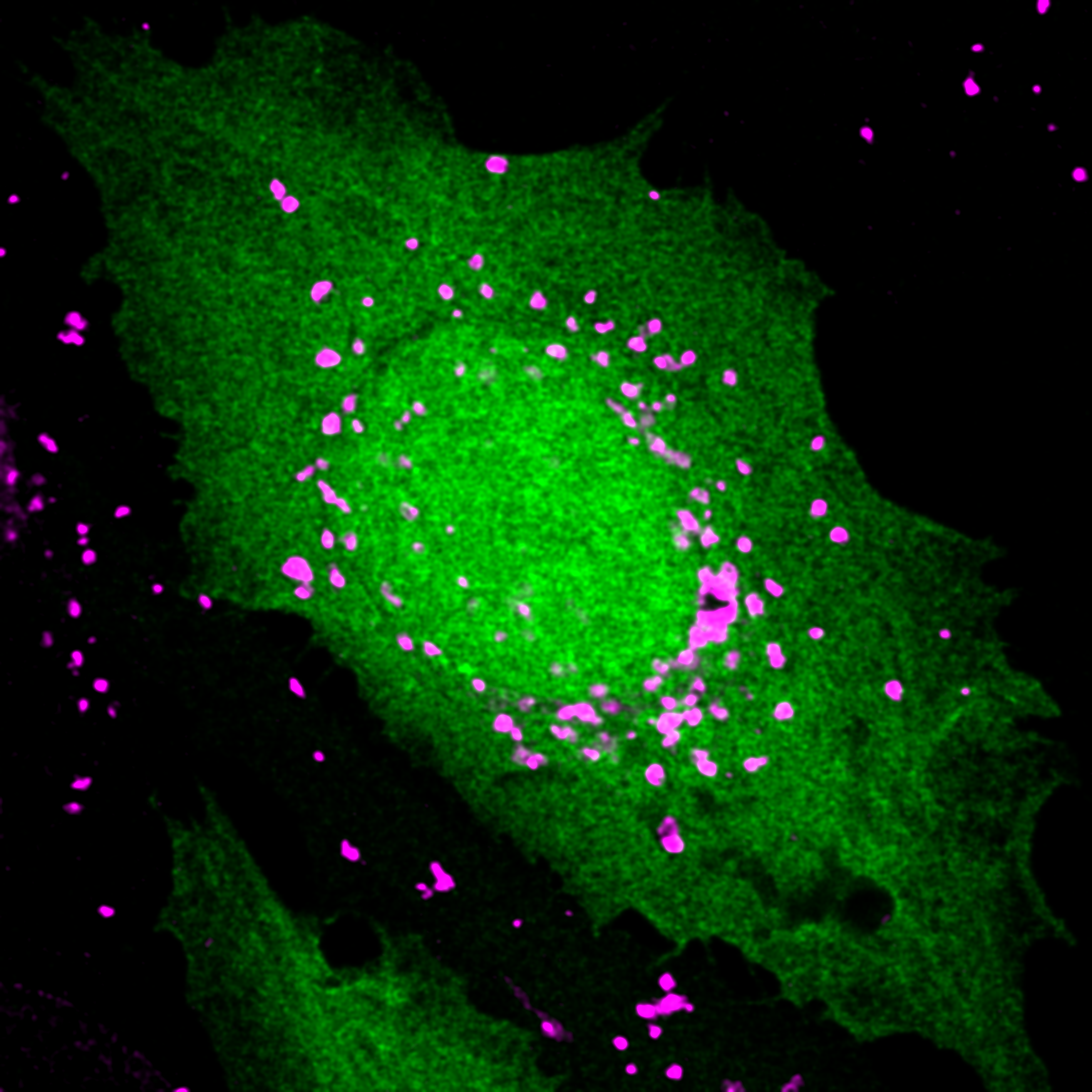

Supplement: Supplementary file 11 — Source data Fig. 4 [file 44318_2025_654_MOESM11_ESM.zip › Figure 4 /4G/4G-1-mCherry-merge.tif]

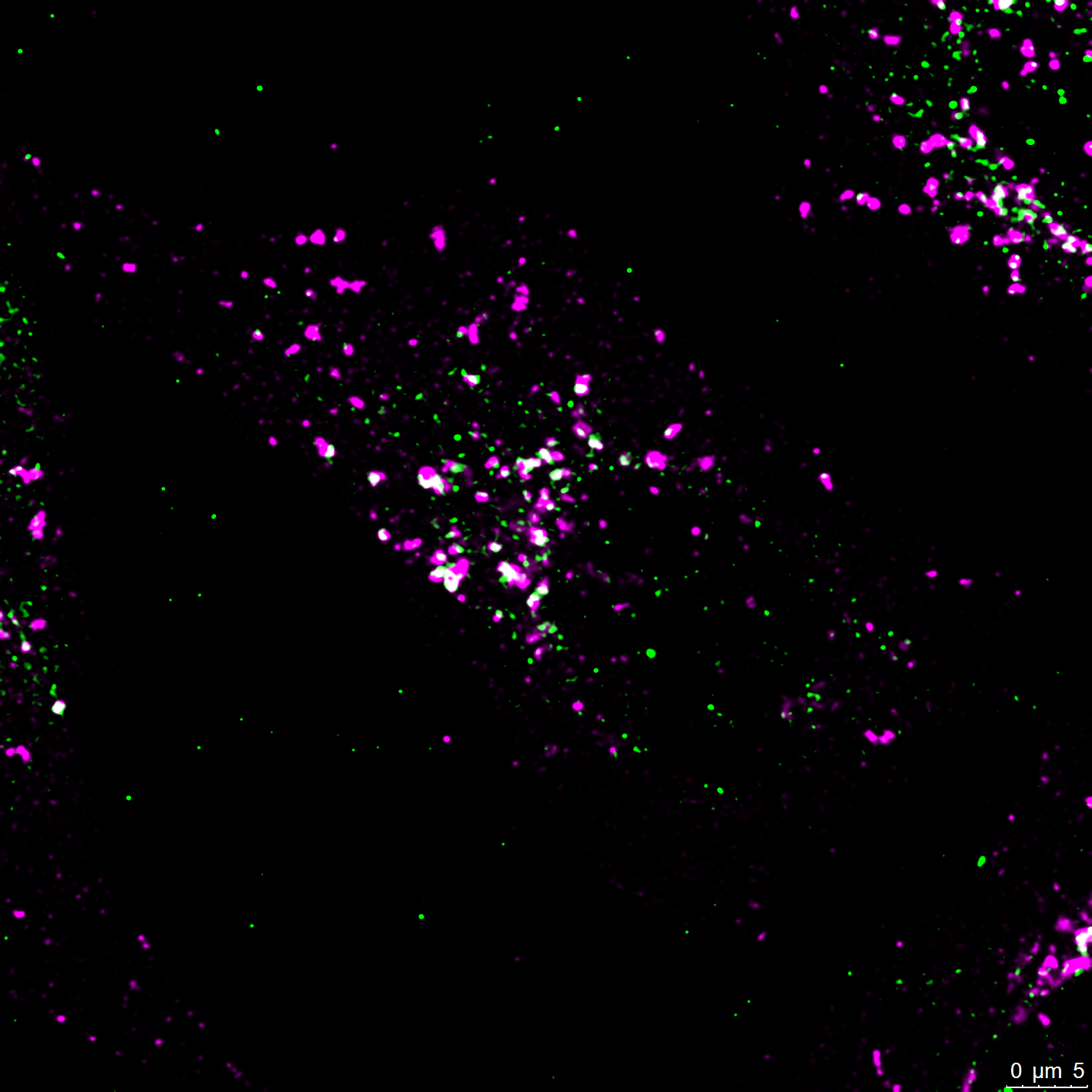

Supplement: Supplementary file 12 — Source data Fig. 5 [file 44318_2025_654_MOESM12_ESM.zip › Figure 5/5I/5I-2-shUBAC2_merge.tif]

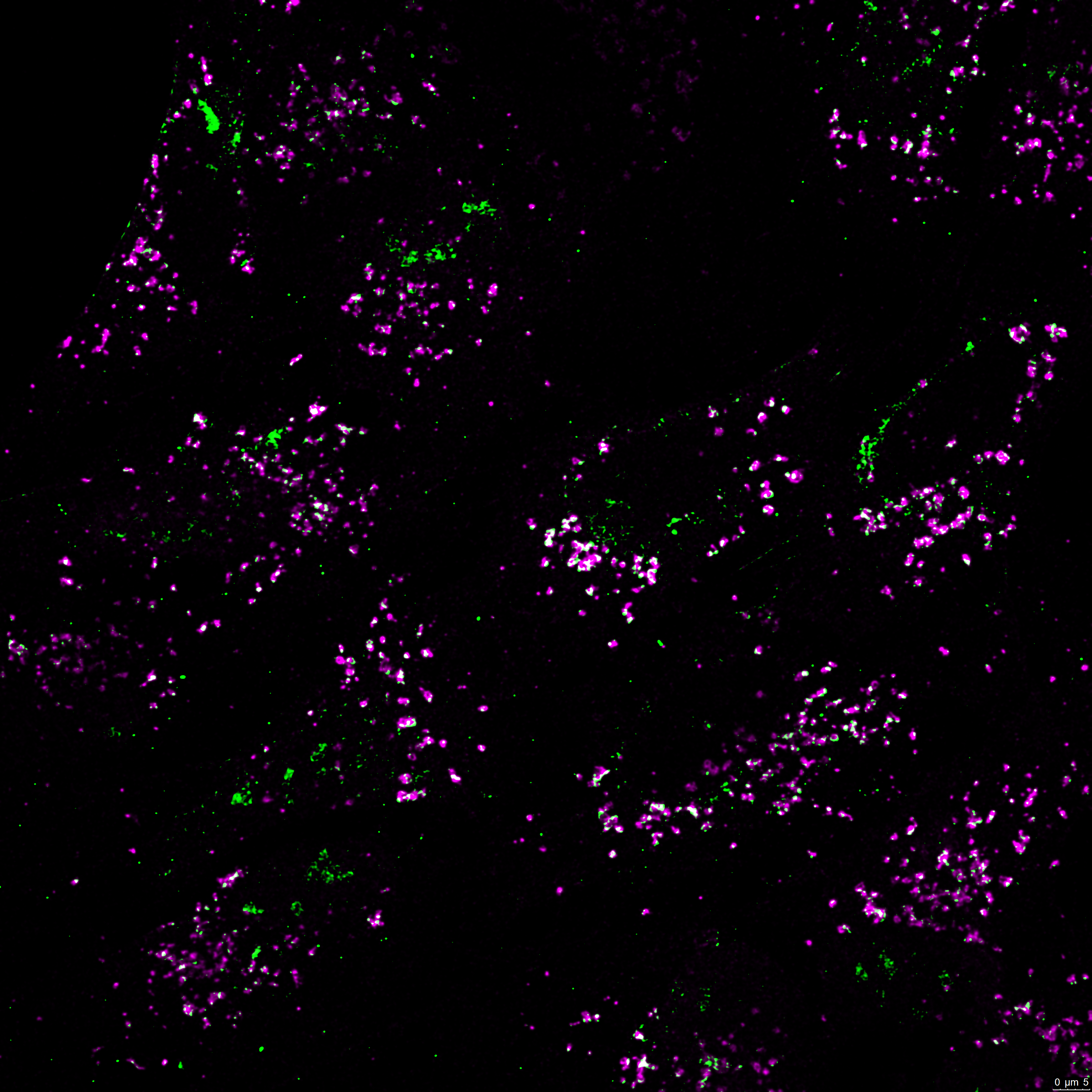

Supplement: Supplementary file 12 — Source data Fig. 5 [file 44318_2025_654_MOESM12_ESM.zip › Figure 5/5I/5I-1-shNC_merge.tif]

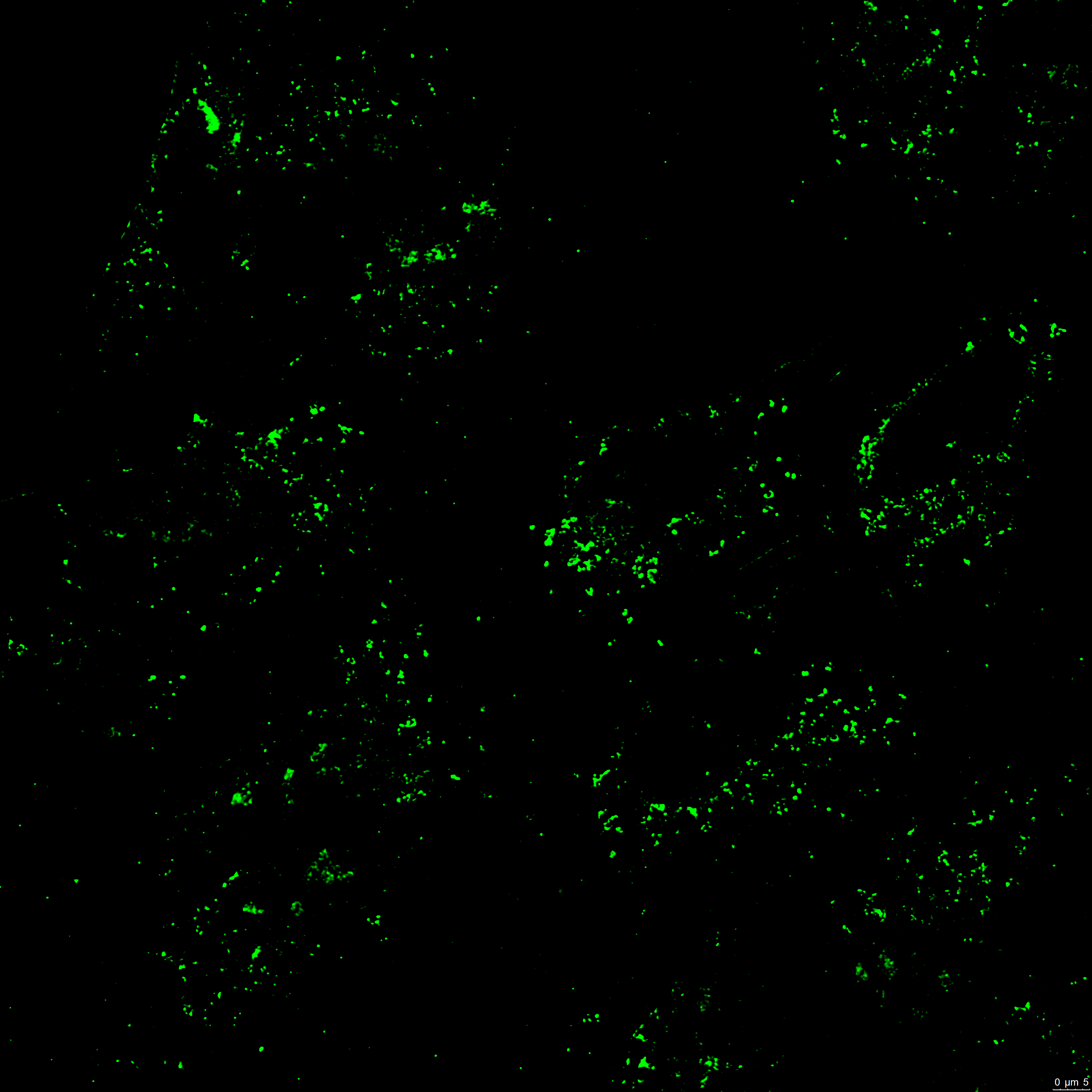

Supplement: Supplementary file 12 — Source data Fig. 5 [file 44318_2025_654_MOESM12_ESM.zip › Figure 5/5I/5I-1-shNC_ATP6V1D.tif]

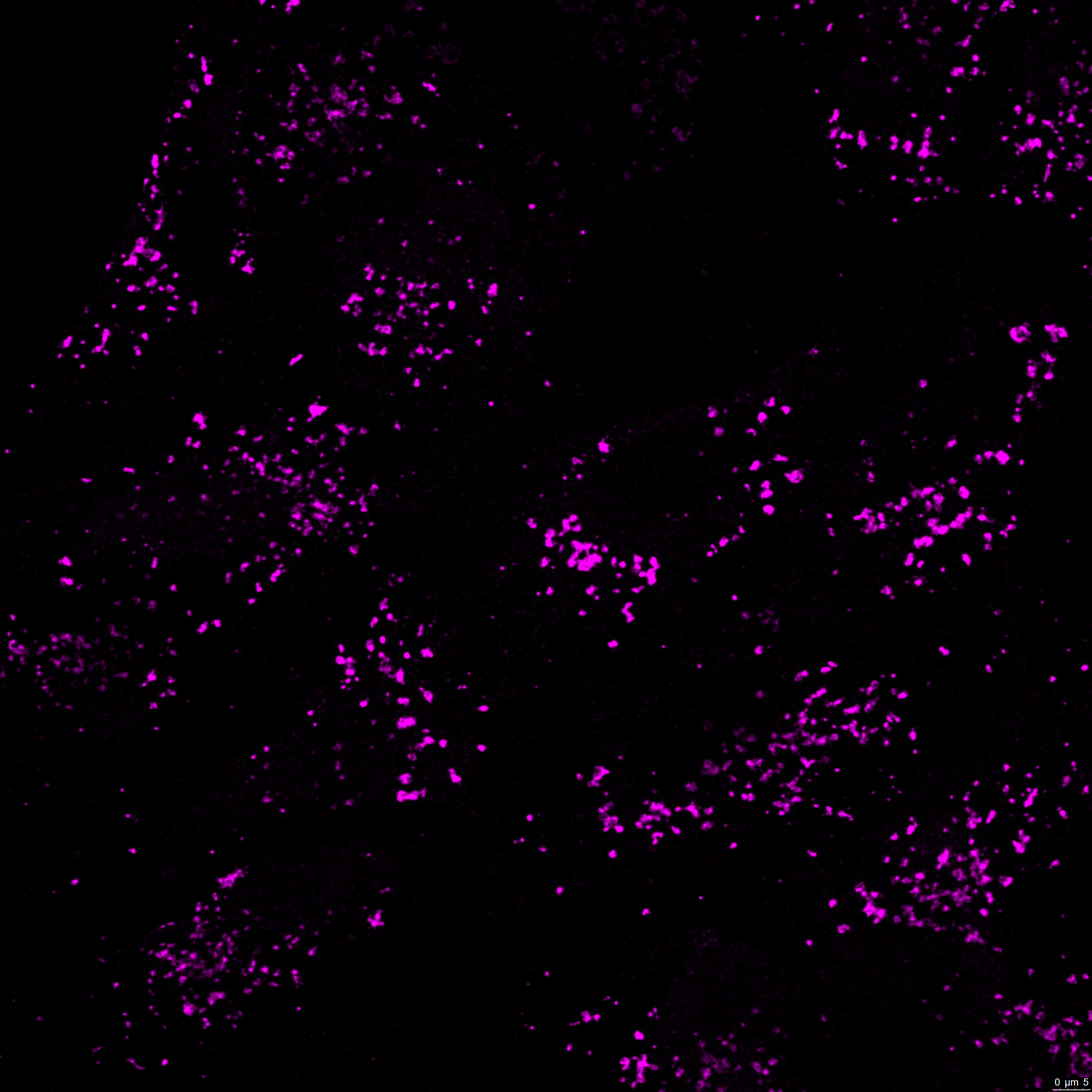

Supplement: Supplementary file 12 — Source data Fig. 5 [file 44318_2025_654_MOESM12_ESM.zip › Figure 5/5I/5I-1-shNC_LAMP1.tif]

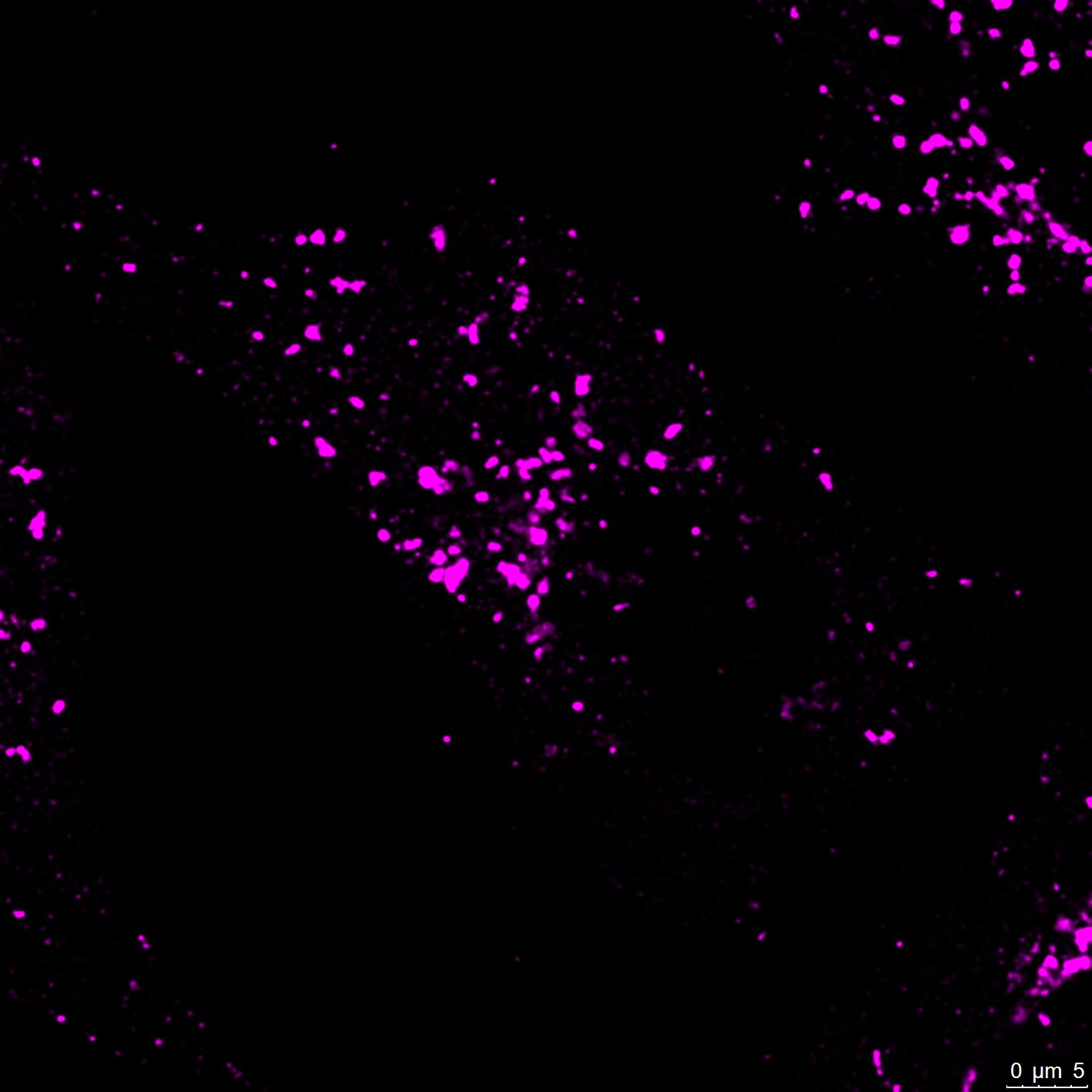

Supplement: Supplementary file 12 — Source data Fig. 5 [file 44318_2025_654_MOESM12_ESM.zip › Figure 5/5I/5I-2-shUBAC2_LAMP1.tif]

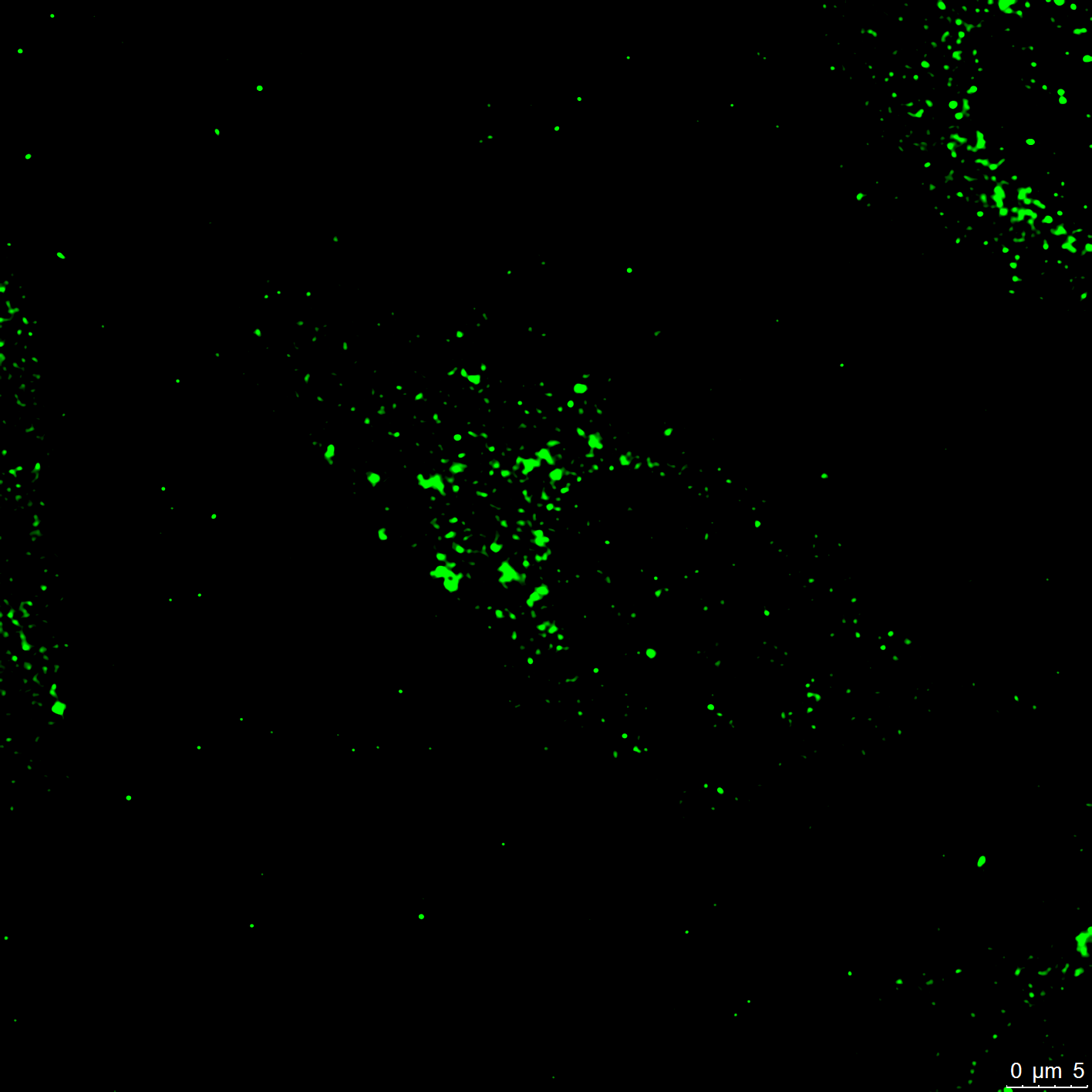

Supplement: Supplementary file 12 — Source data Fig. 5 [file 44318_2025_654_MOESM12_ESM.zip › Figure 5/5I/5I-2-shUBAC2_ATP6V1D.tif]

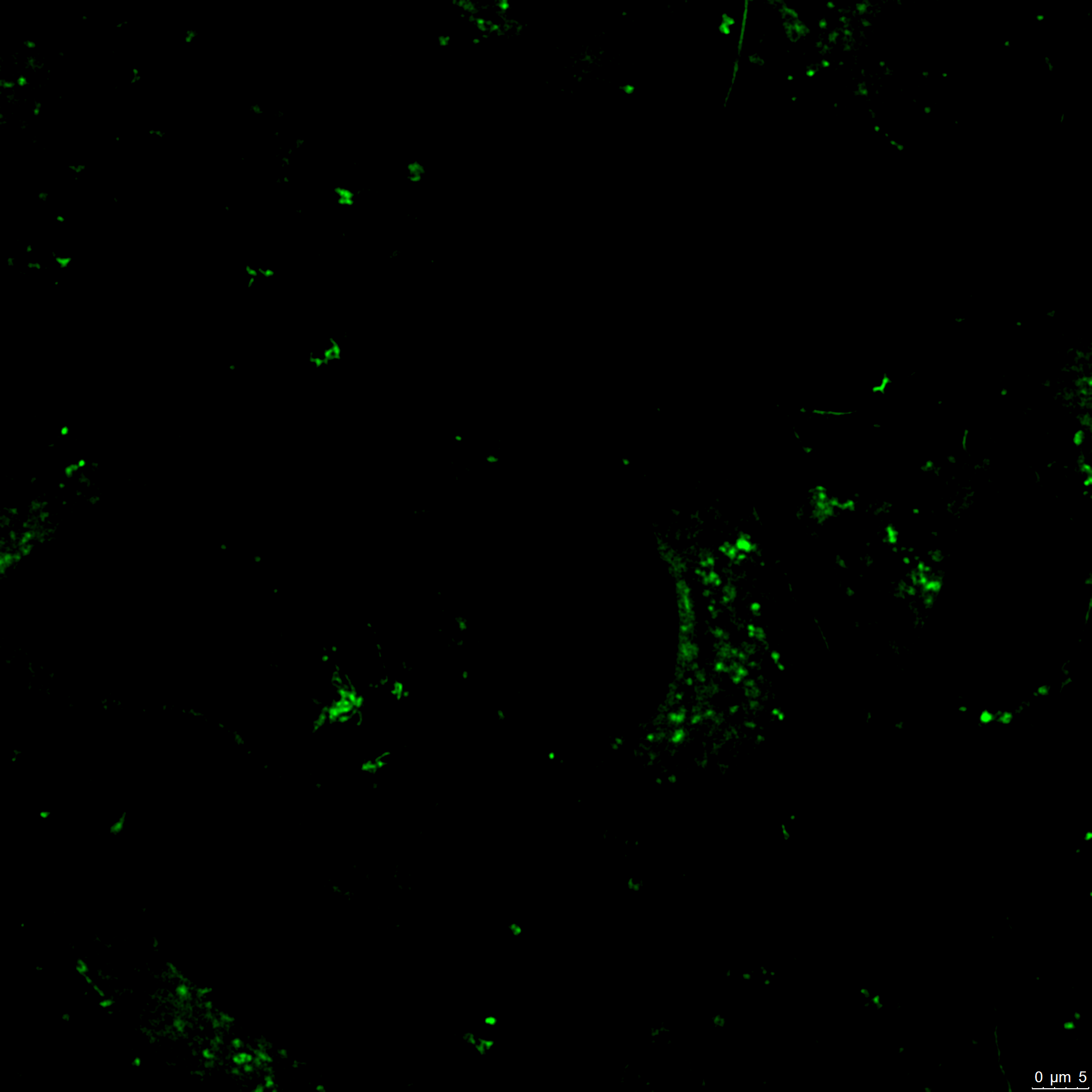

Supplement: Supplementary file 12 — Source data Fig. 5 [file 44318_2025_654_MOESM12_ESM.zip › Figure 5/5G/5G-1-shNC-lysosensor green.tif]

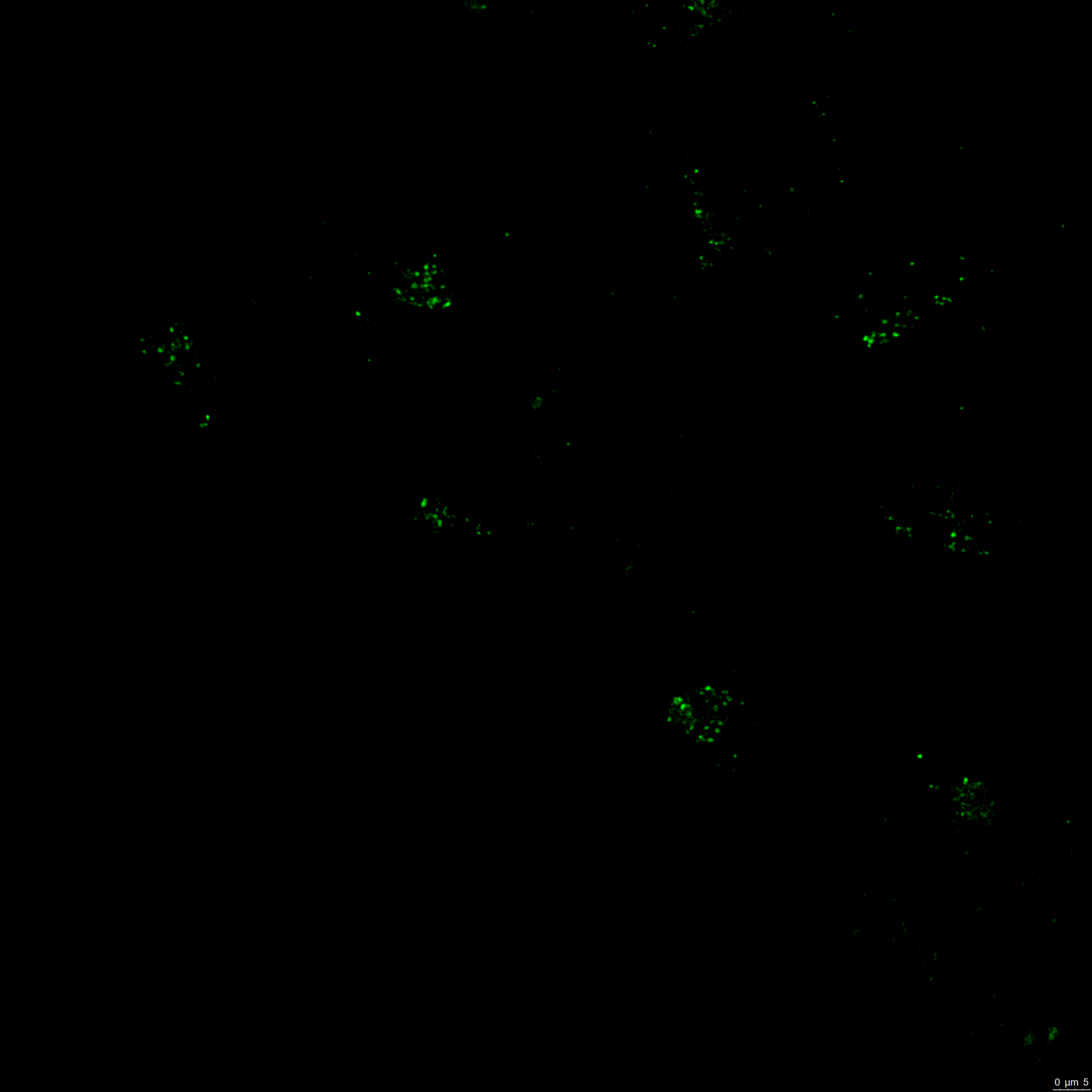

Supplement: Supplementary file 12 — Source data Fig. 5 [file 44318_2025_654_MOESM12_ESM.zip › Figure 5/5G/5G-2-shUBAC2-lysosensor green.tif]

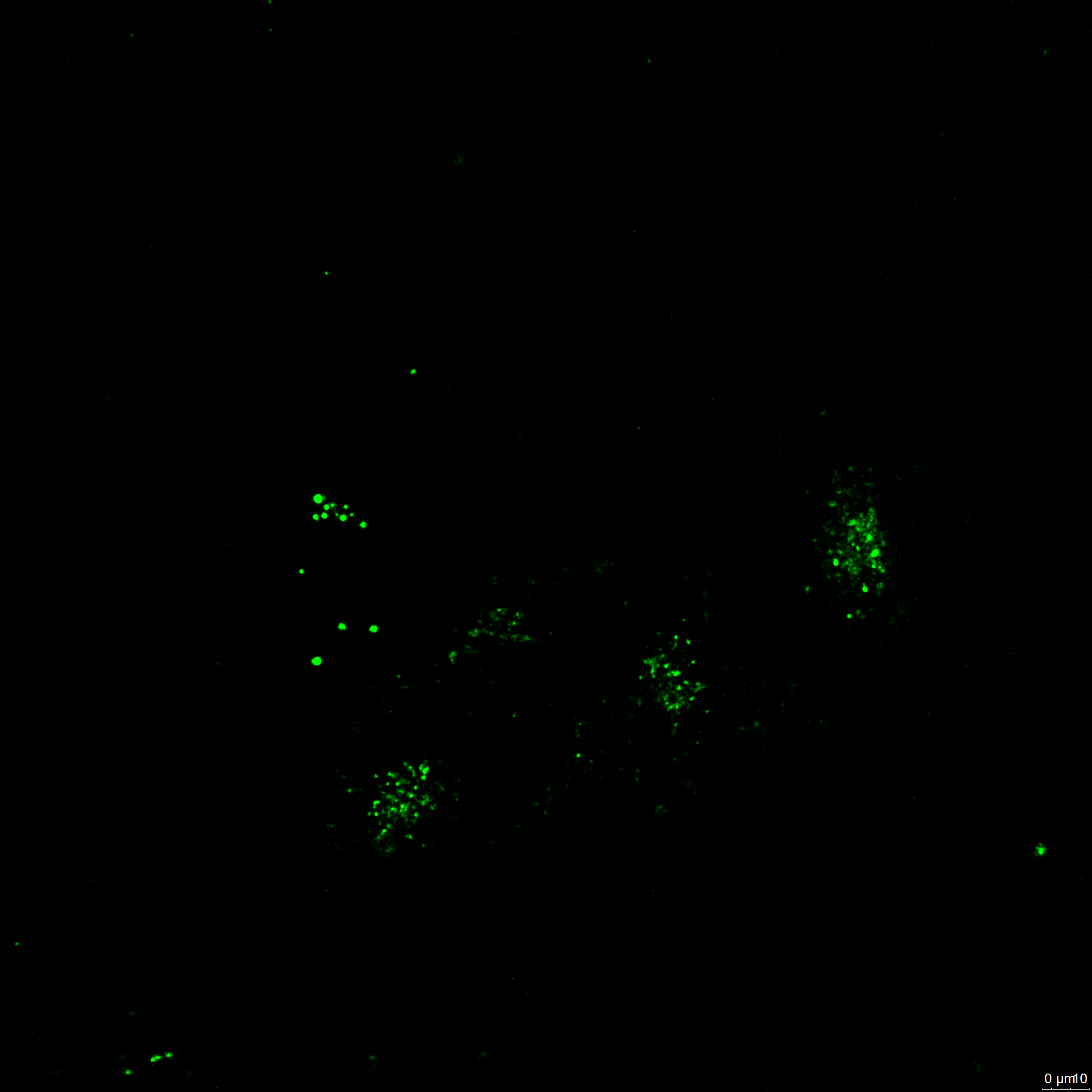

Supplement: Supplementary file 12 — Source data Fig. 5 [file 44318_2025_654_MOESM12_ESM.zip › Figure 5/5M/5M-2_shUBAC2_SiR-lysosome.tif]

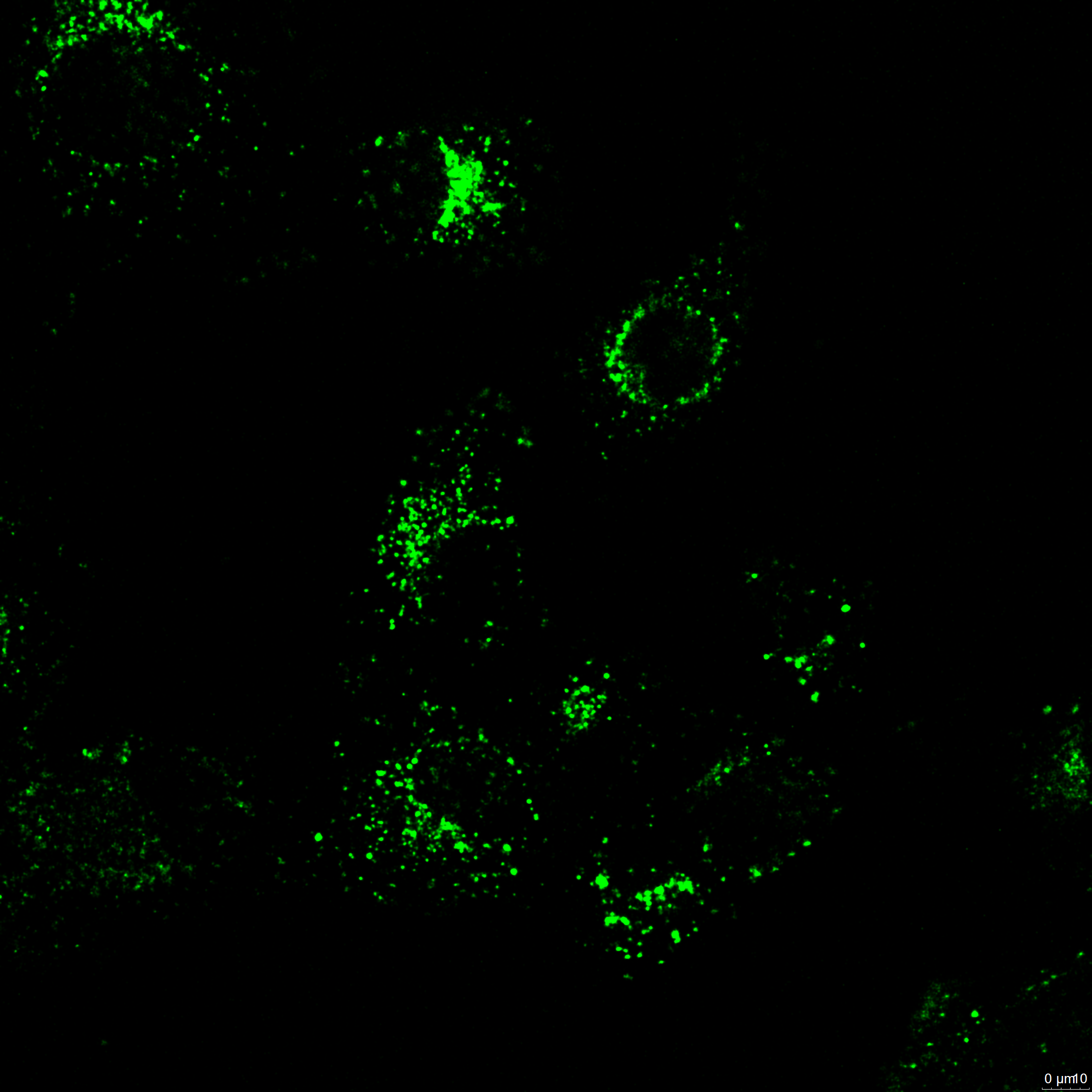

Supplement: Supplementary file 12 — Source data Fig. 5 [file 44318_2025_654_MOESM12_ESM.zip › Figure 5/5M/5M-1_shNC_SiR-lysosome.tif]

|               |    |   |                      |   |    |       |
|---------------|----|---|----------------------|---|----|-------|
| UBAC2-myc     | WT |   | AREL1 <sup>-/-</sup> |   |    |       |
| ATP6V1B2-FLAG | +  | + | +                    | + | +  | +     |
| AREL1-EGFP    | -  | + | -                    | + | +  | +     |
|               | -  | - | -                    | - | WT | C790A |

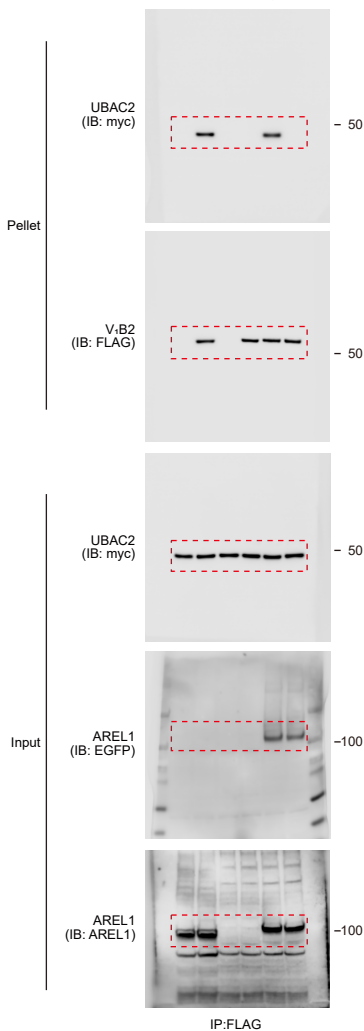

Supplement: Supplementary file 12 — Source data Fig. 5 [file 44318_2025_654_MOESM12_ESM.zip › Figure 5/5C/5C.pdf]

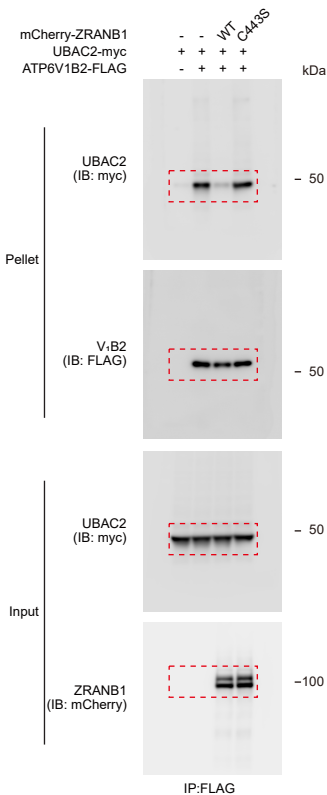

Supplement: Supplementary file 12 — Source data Fig. 5 [file 44318_2025_654_MOESM12_ESM.zip › Figure 5/5D/5D.pdf]

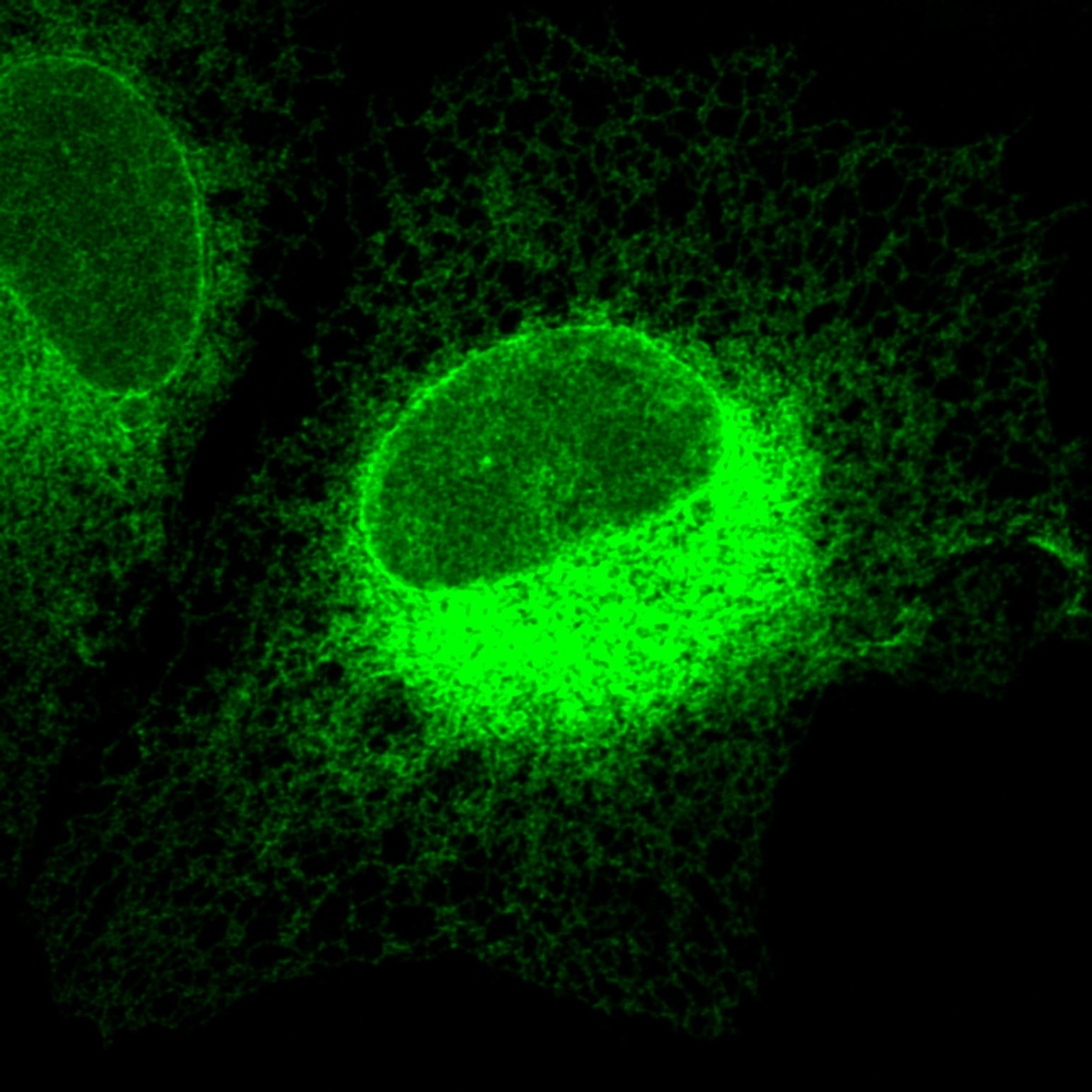

Supplement: Supplementary file 12 — Source data Fig. 5 [file 44318_2025_654_MOESM12_ESM.zip › Figure 5/5E/5E-3-shUBAC2 expressing UBAC2(WT)-EGFP.tif]

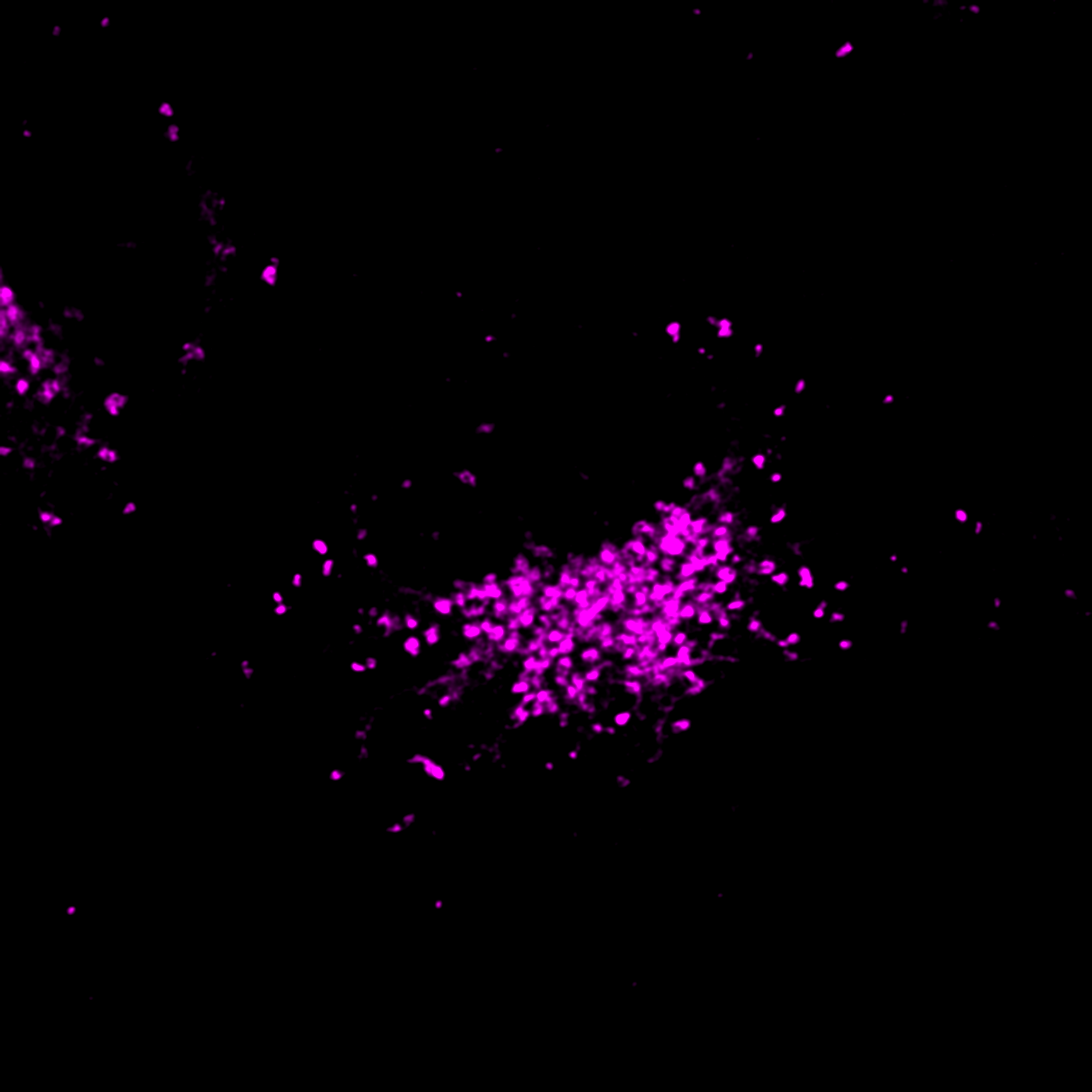

Supplement: Supplementary file 12 — Source data Fig. 5 [file 44318_2025_654_MOESM12_ESM.zip › Figure 5/5E/5E-3-shUBAC2 expressing UBAC2(WT)-EGFP, LAMP1.tif]

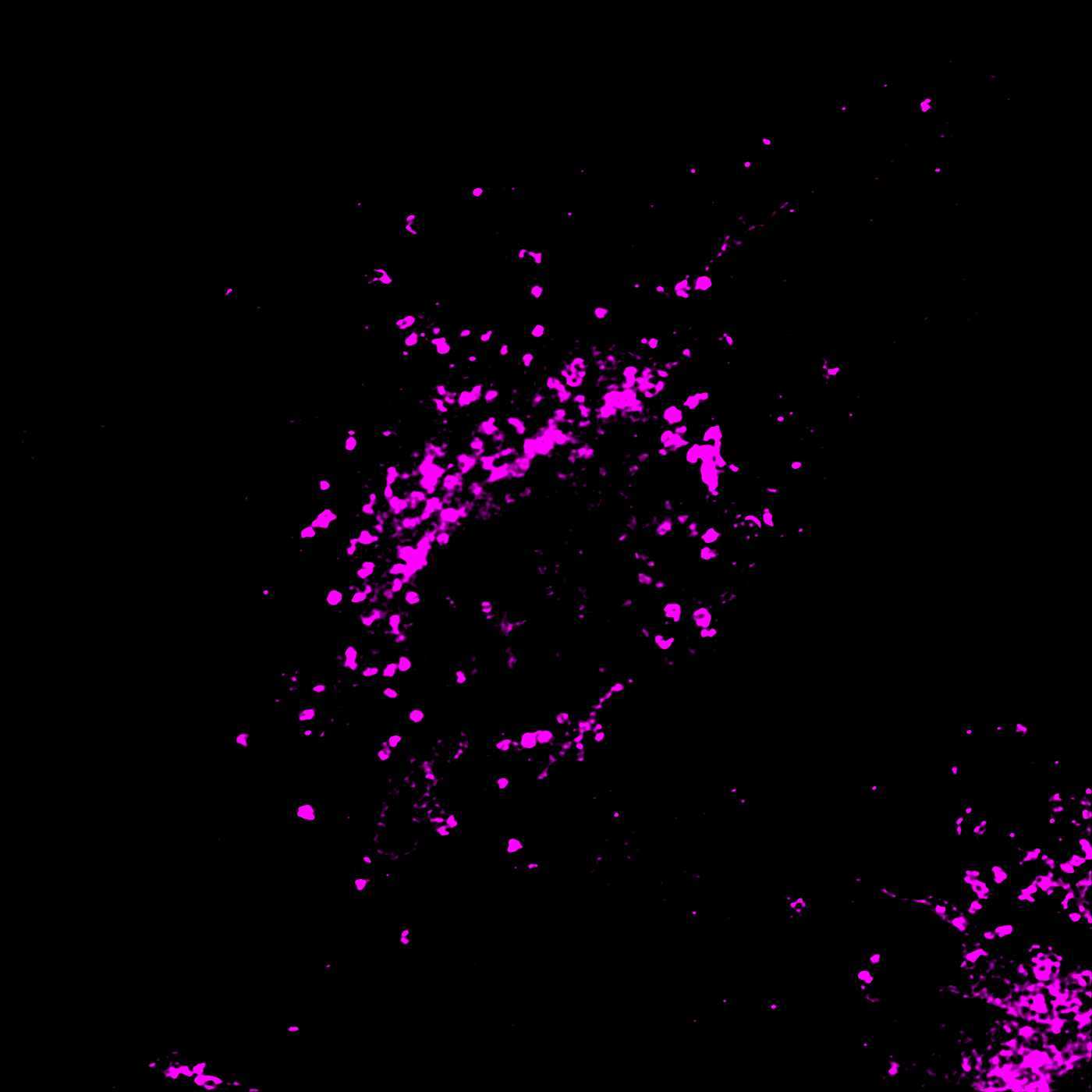

Supplement: Supplementary file 12 — Source data Fig. 5 [file 44318_2025_654_MOESM12_ESM.zip › Figure 5/5E/5E-1-shNC expressing EGFP,LAMP1.tif]

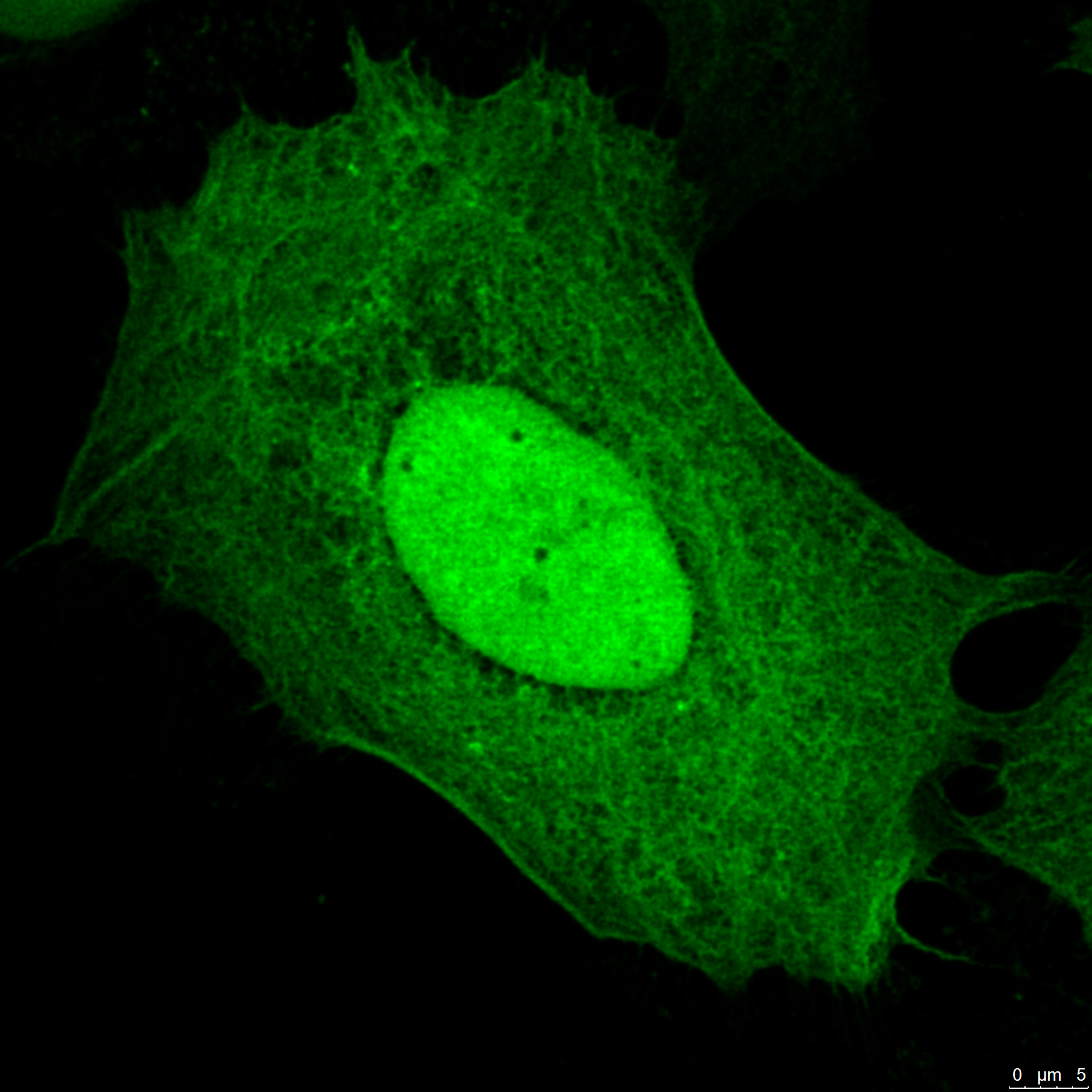

Supplement: Supplementary file 12 — Source data Fig. 5 [file 44318_2025_654_MOESM12_ESM.zip › Figure 5/5E/5E-2-shUBAC2 expressing EGFP.tif]

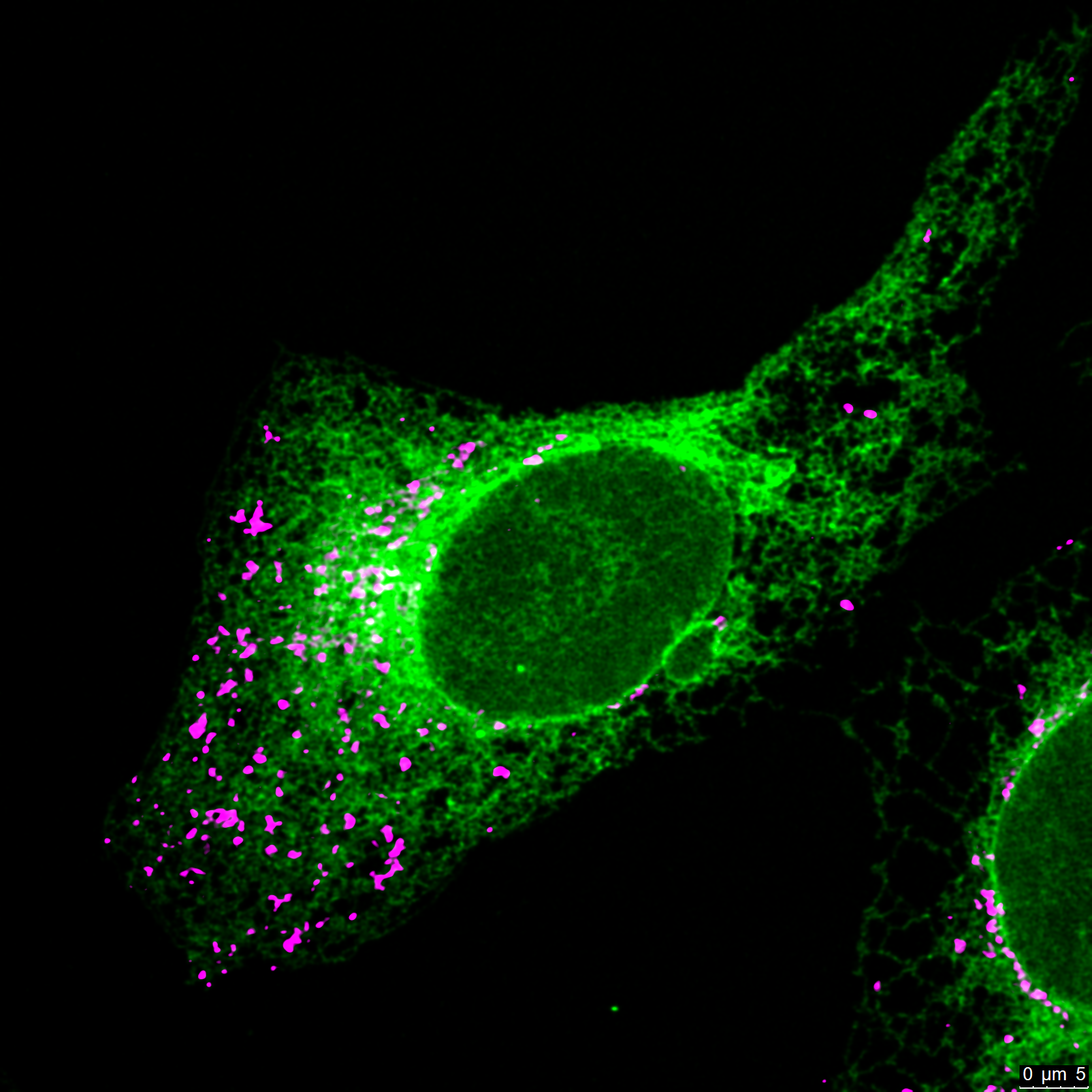

Supplement: Supplementary file 12 — Source data Fig. 5 [file 44318_2025_654_MOESM12_ESM.zip › Figure 5/5E/5E-4-shUBAC2 expressing UBAC2(╬öUBA)-EGFP, merge.tif]

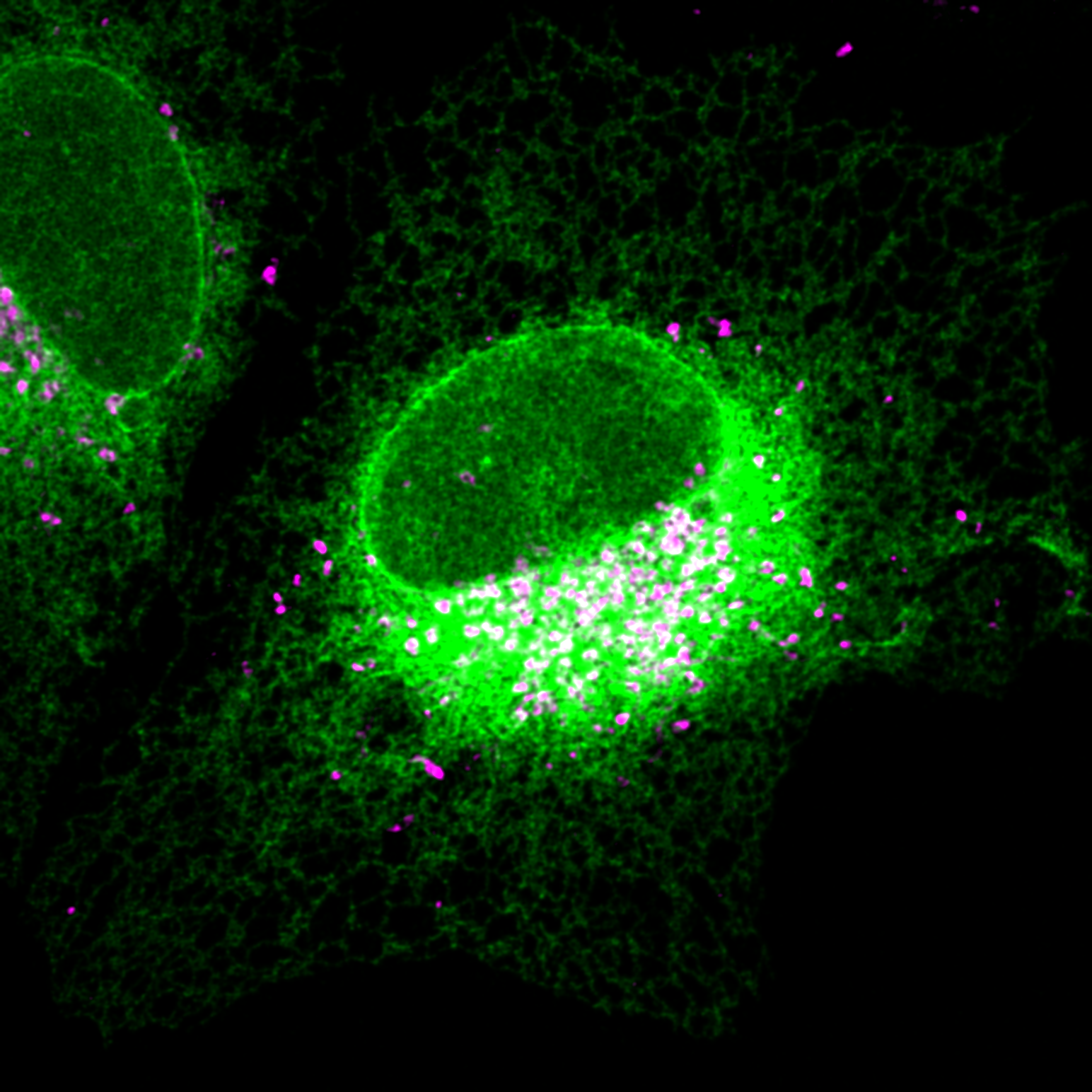

Supplement: Supplementary file 12 — Source data Fig. 5 [file 44318_2025_654_MOESM12_ESM.zip › Figure 5/5E/5E-3-shUBAC2 expressing UBAC2(WT)-EGFP, merge.tif]

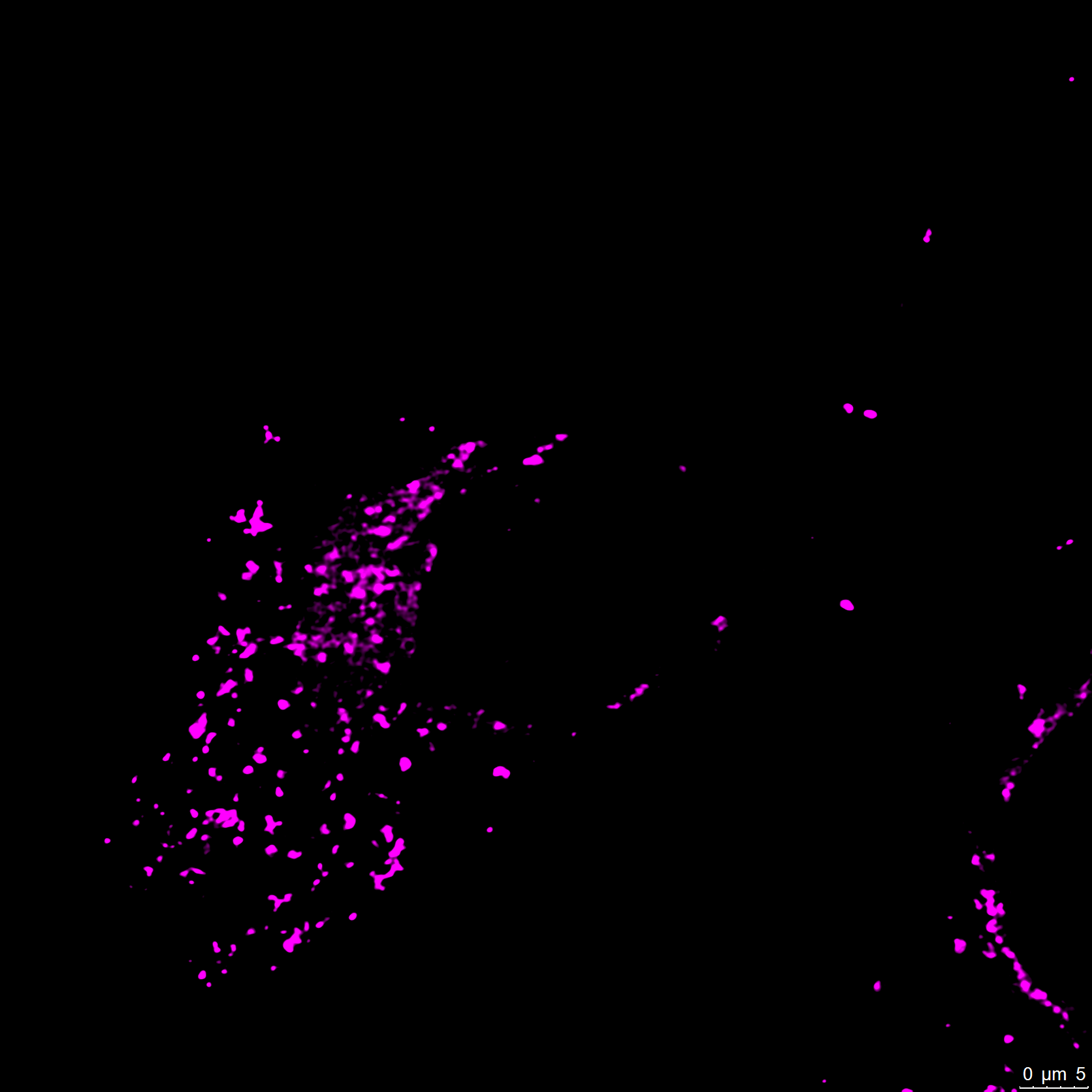

Supplement: Supplementary file 12 — Source data Fig. 5 [file 44318_2025_654_MOESM12_ESM.zip › Figure 5/5E/5E-4-shUBAC2 expressing UBAC2(╬öUBA)-EGFP,LAMP1.tif]

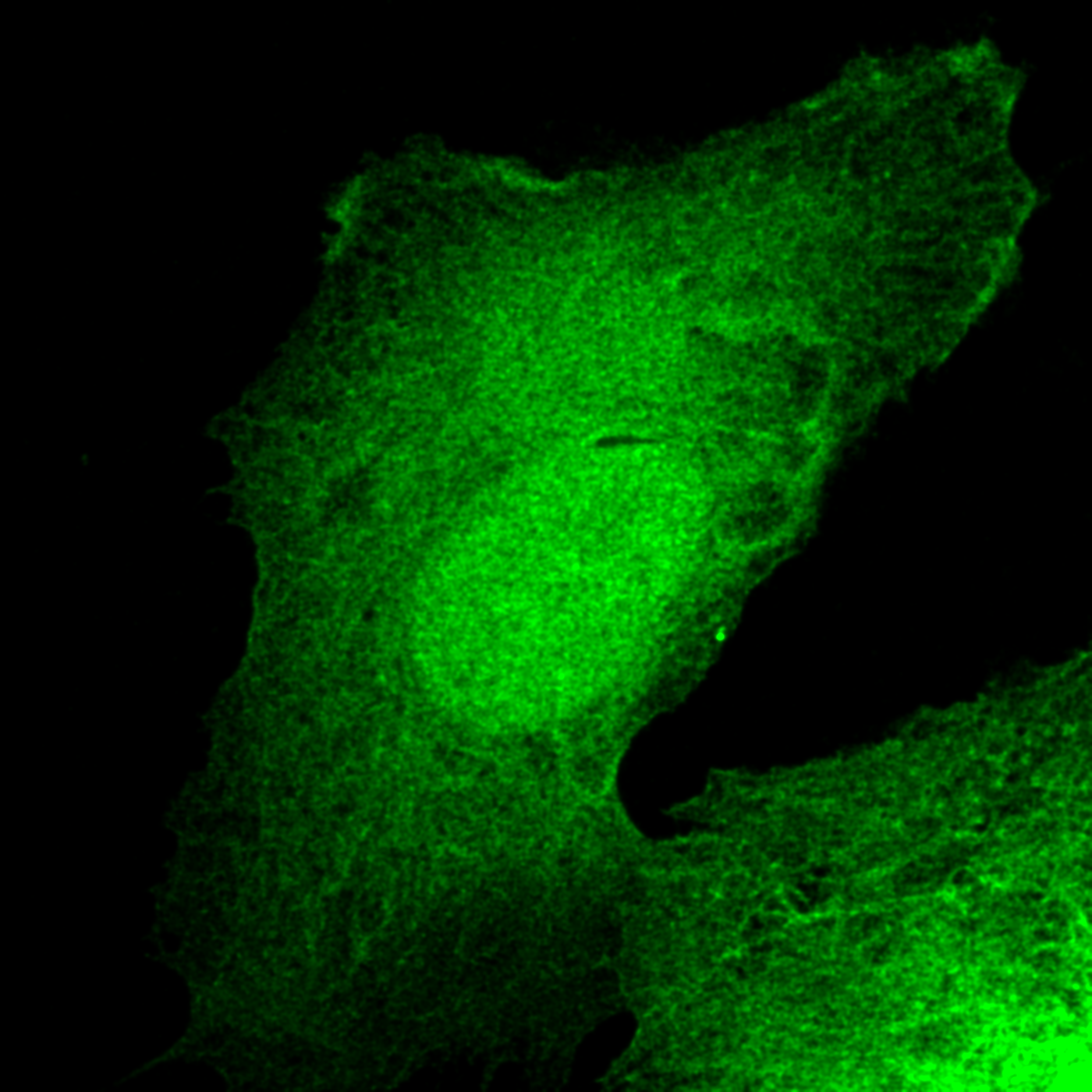

Supplement: Supplementary file 12 — Source data Fig. 5 [file 44318_2025_654_MOESM12_ESM.zip › Figure 5/5E/5E-1-shNC expressing EGFP.tif]

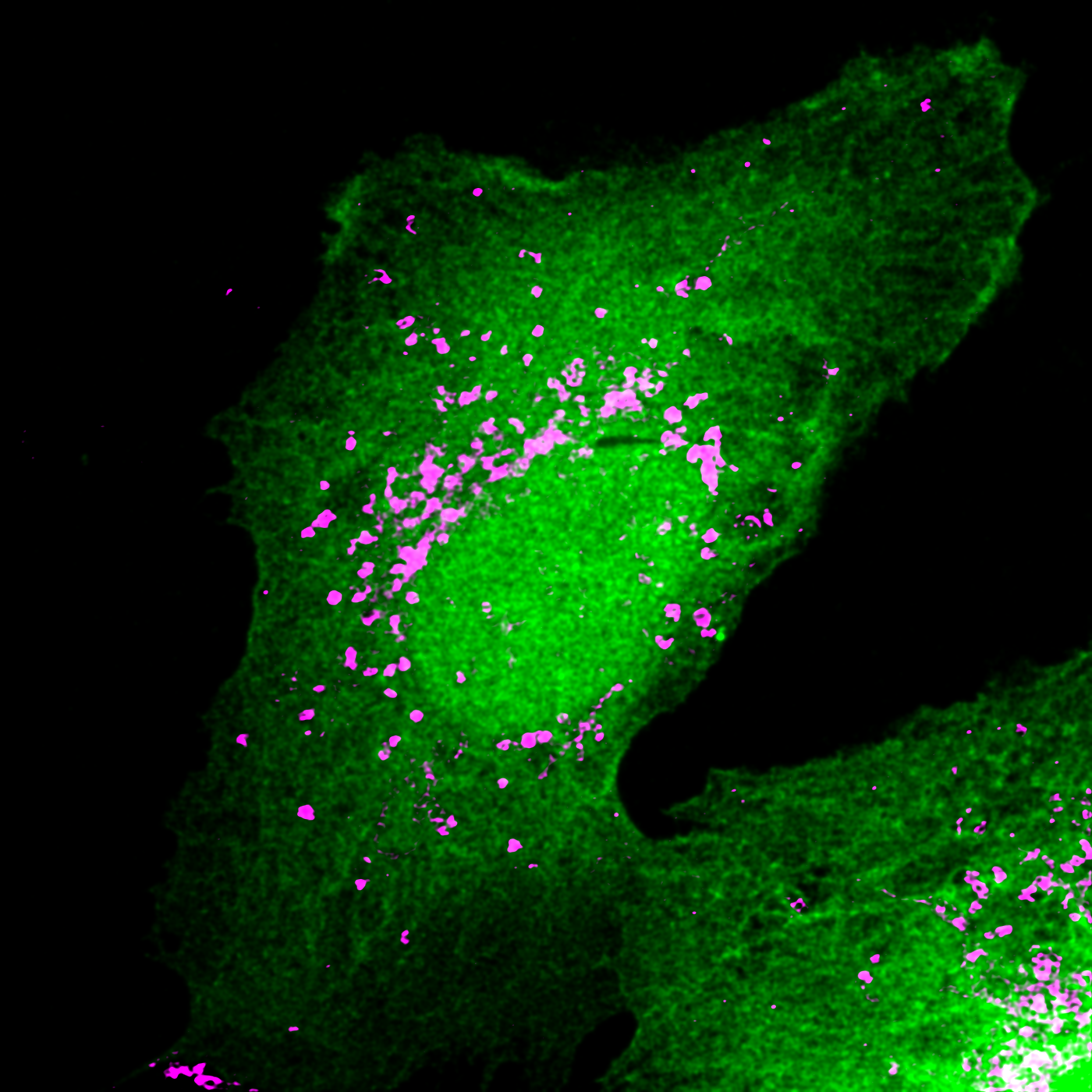

Supplement: Supplementary file 12 — Source data Fig. 5 [file 44318_2025_654_MOESM12_ESM.zip › Figure 5/5E/5E-1-shNC expressing EGFP, merge.tif]

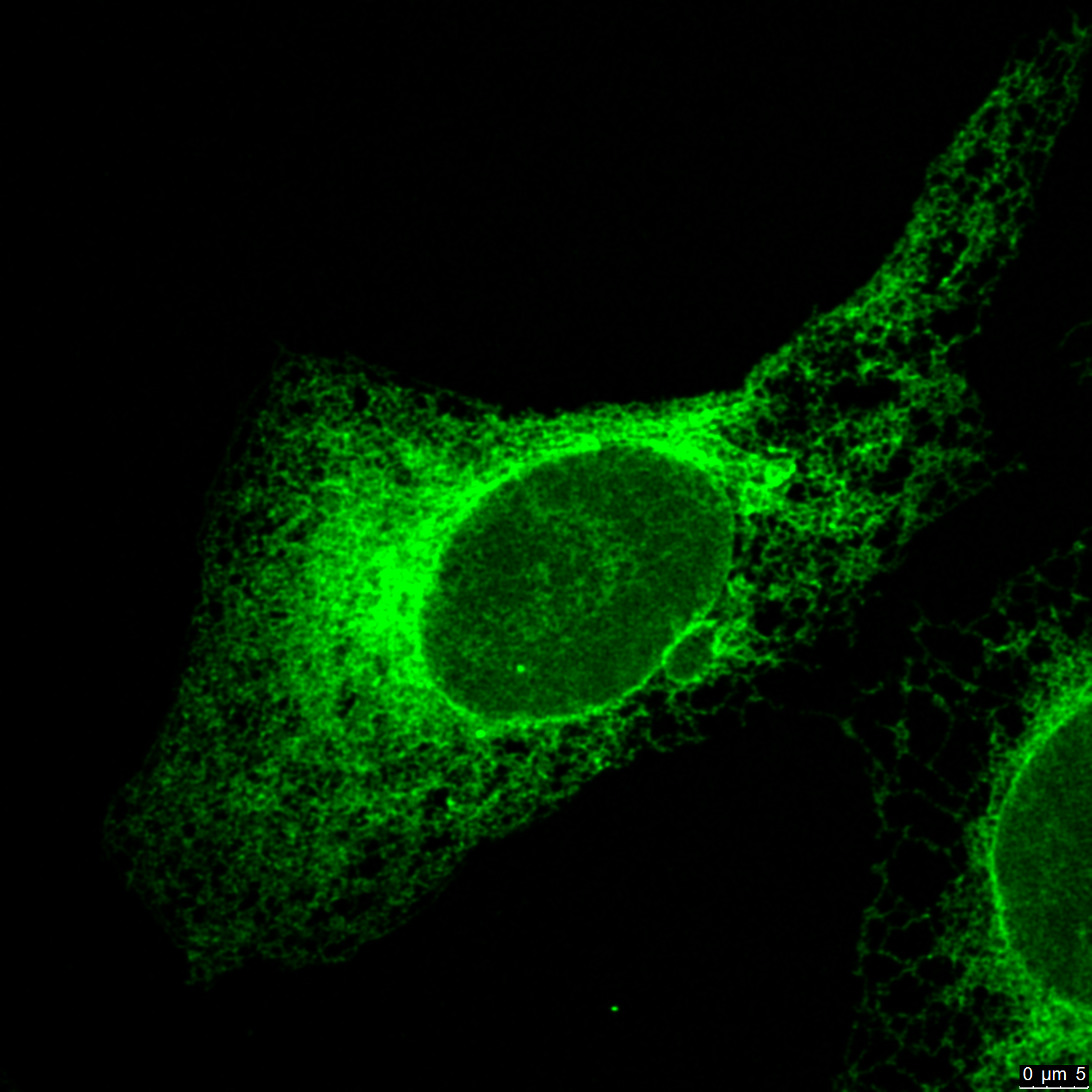

Supplement: Supplementary file 12 — Source data Fig. 5 [file 44318_2025_654_MOESM12_ESM.zip › Figure 5/5E/5E-4-shUBAC2 expressing UBAC2(╬öUBA)-EGFP.tif]

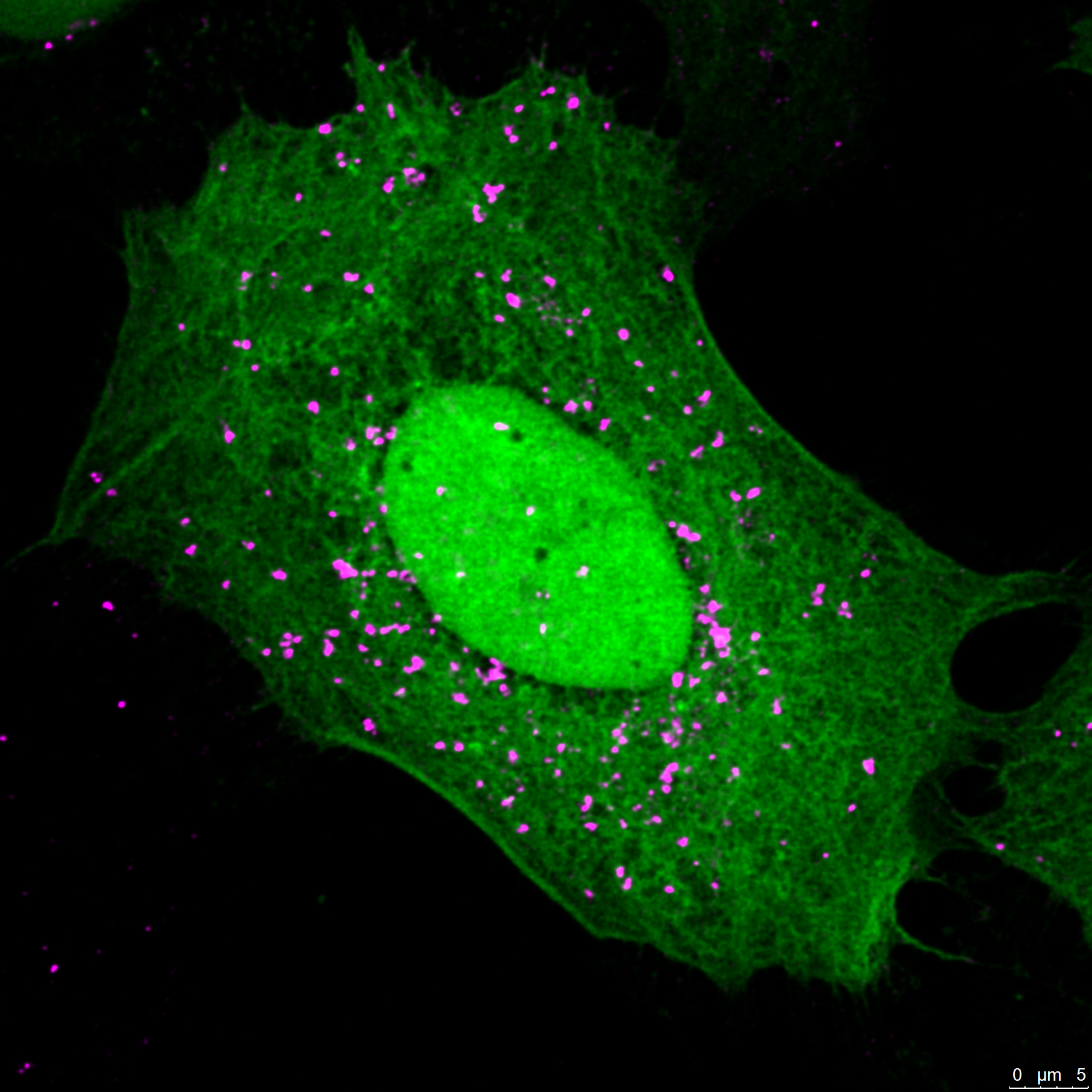

Supplement: Supplementary file 12 — Source data Fig. 5 [file 44318_2025_654_MOESM12_ESM.zip › Figure 5/5E/5E-2-shUBAC2 expressing EGFP, merge.tif]

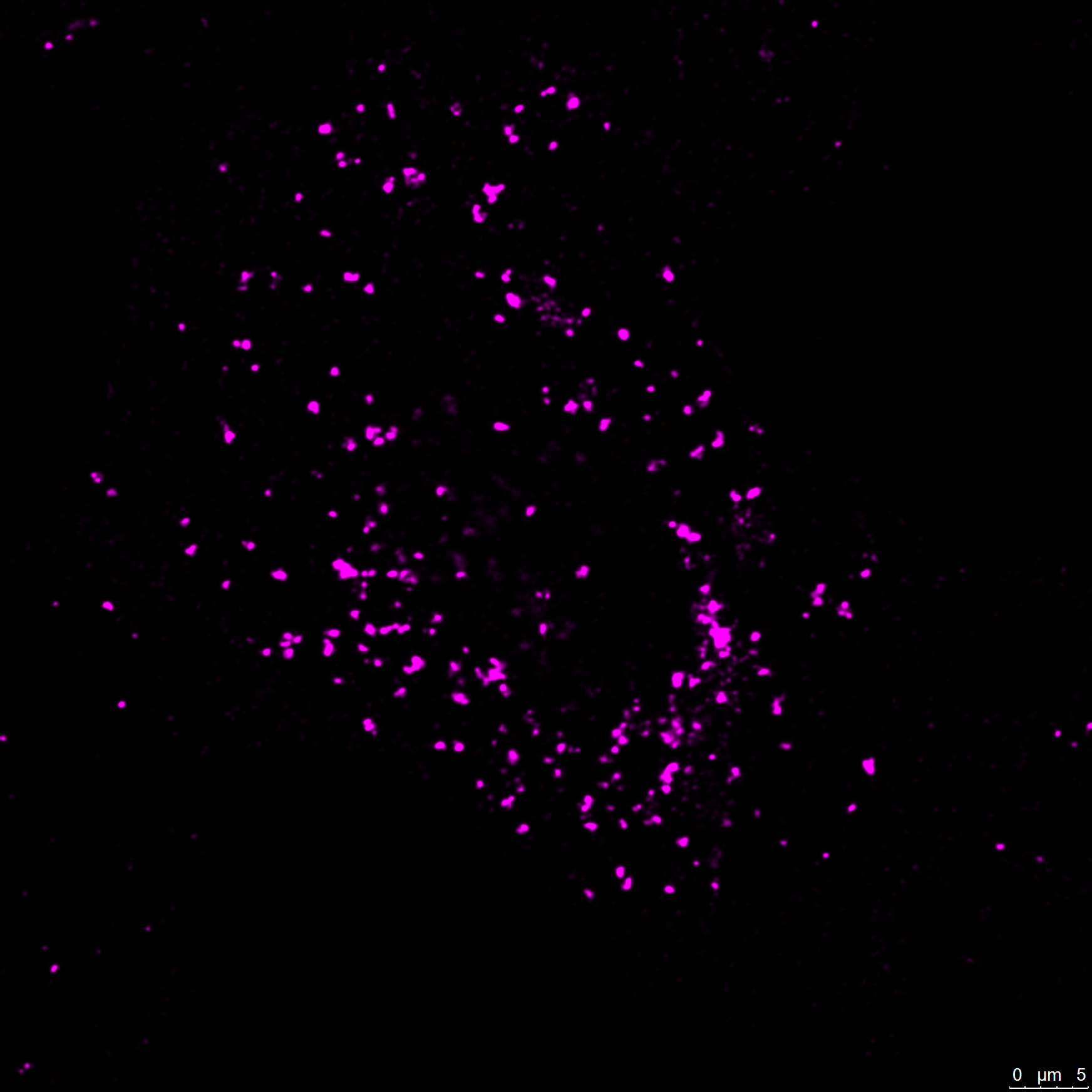

Supplement: Supplementary file 12 — Source data Fig. 5 [file 44318_2025_654_MOESM12_ESM.zip › Figure 5/5E/5E-2-shUBAC2 expressing EGFP,LAMP1.tif]

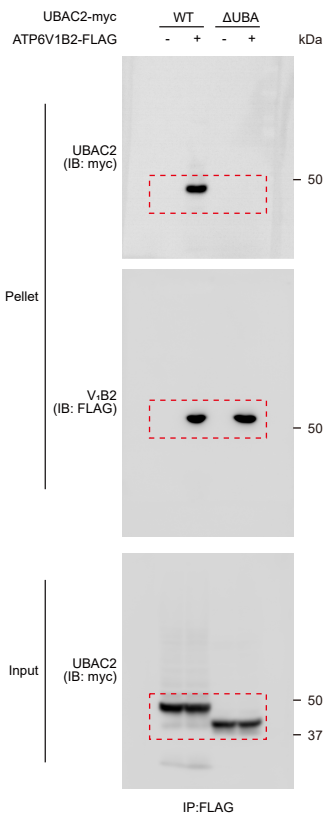

Supplement: Supplementary file 12 — Source data Fig. 5 [file 44318_2025_654_MOESM12_ESM.zip › Figure 5/5B/5B.pdf]

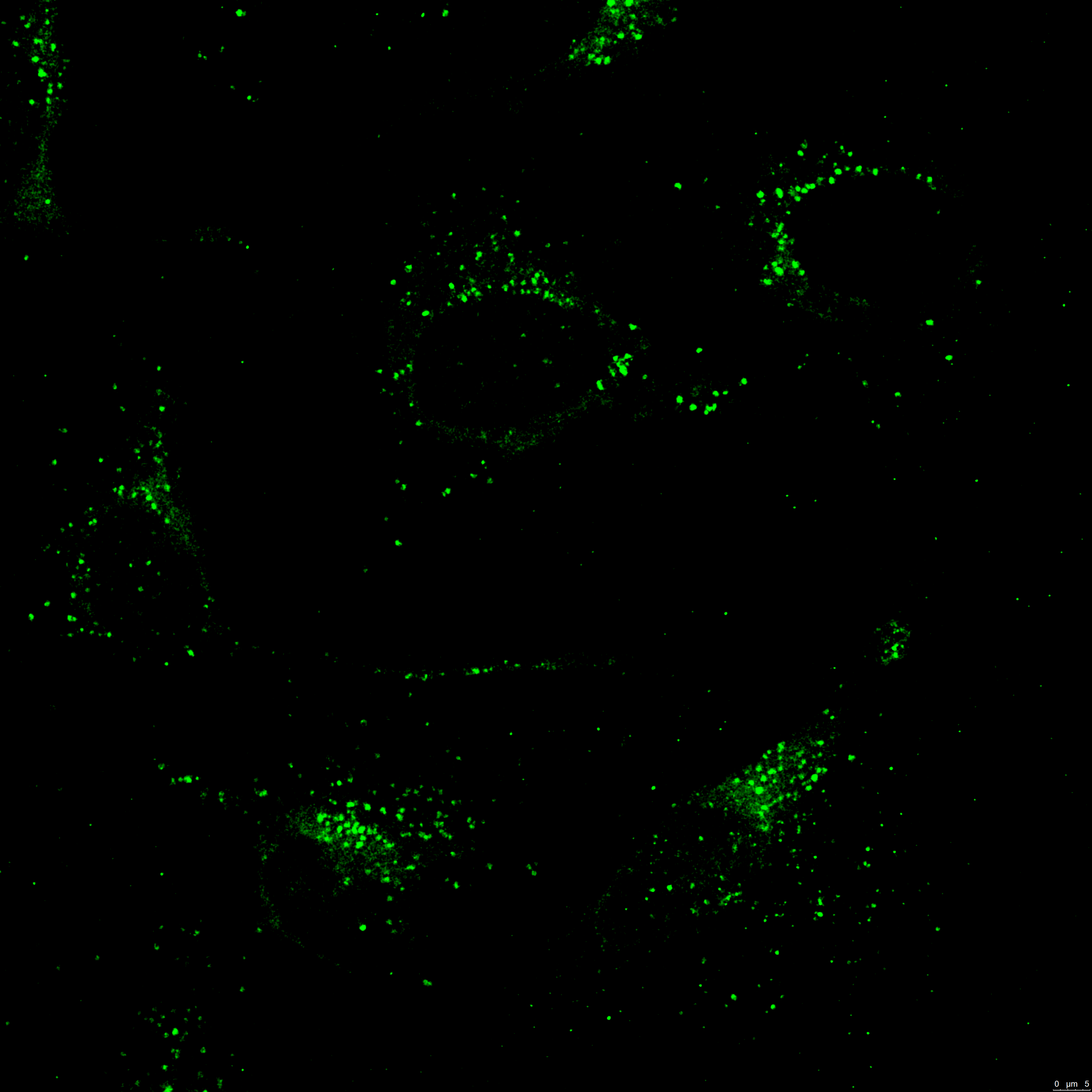

Supplement: Supplementary file 12 — Source data Fig. 5 [file 44318_2025_654_MOESM12_ESM.zip › Figure 5/5L/5L-1-shNC_DQ-OVA.tif]

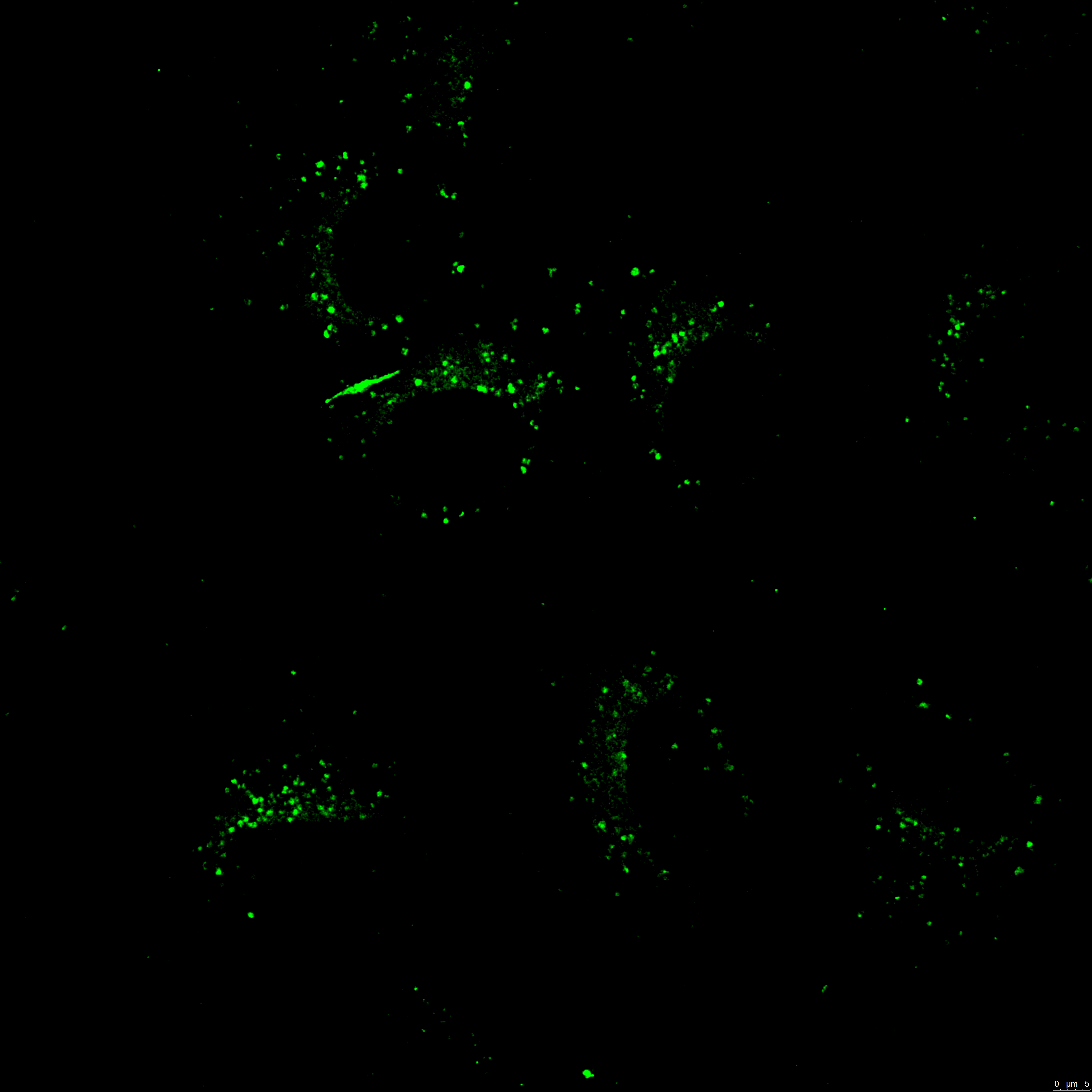

Supplement: Supplementary file 12 — Source data Fig. 5 [file 44318_2025_654_MOESM12_ESM.zip › Figure 5/5L/5L-2-shUBAC2_DQ-OVA.tif]

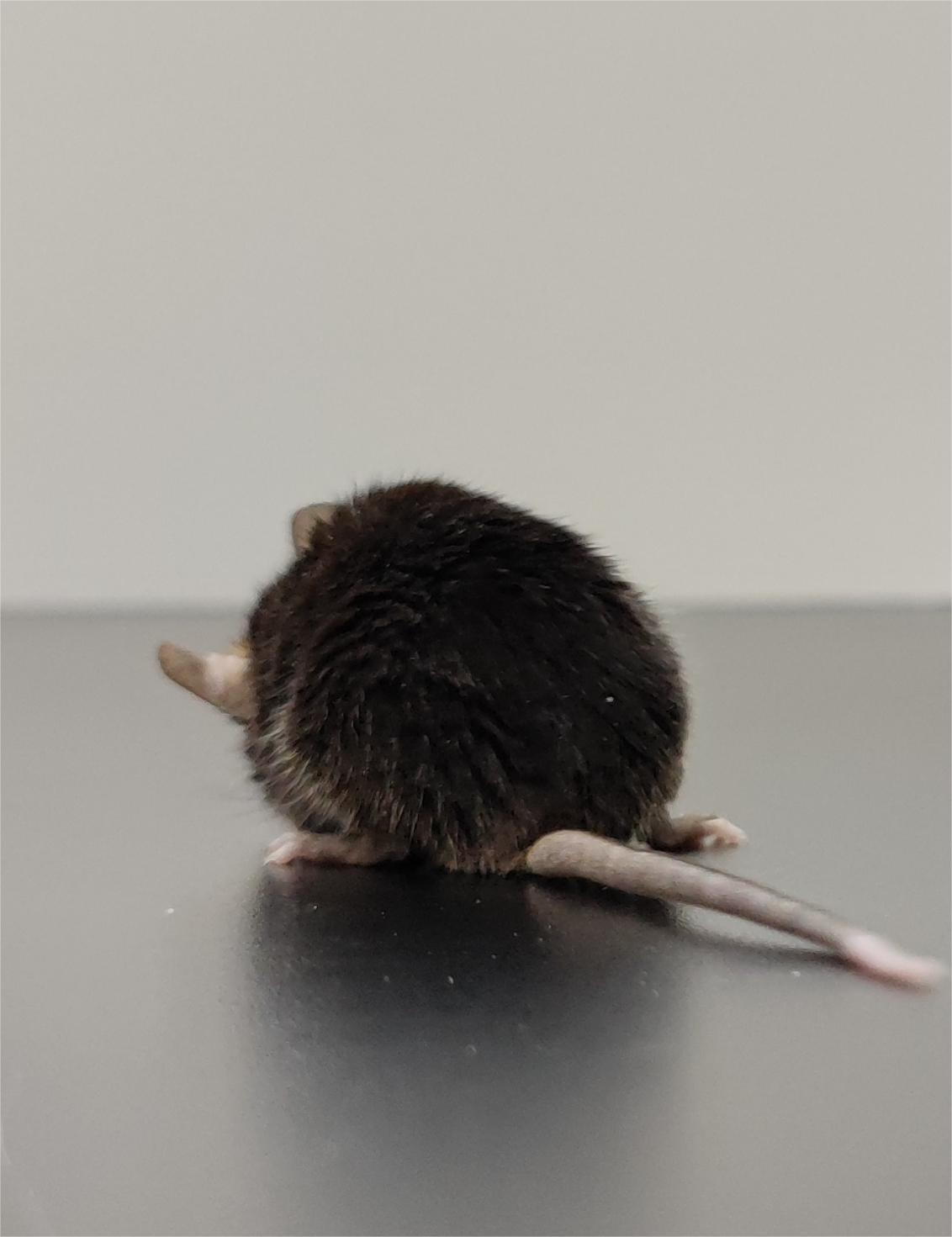

Supplement: Supplementary file 13 — Source data Fig. 6 [file 44318_2025_654_MOESM13_ESM.zip › Figure 6/6A/6A-2-KO-2.jpg]

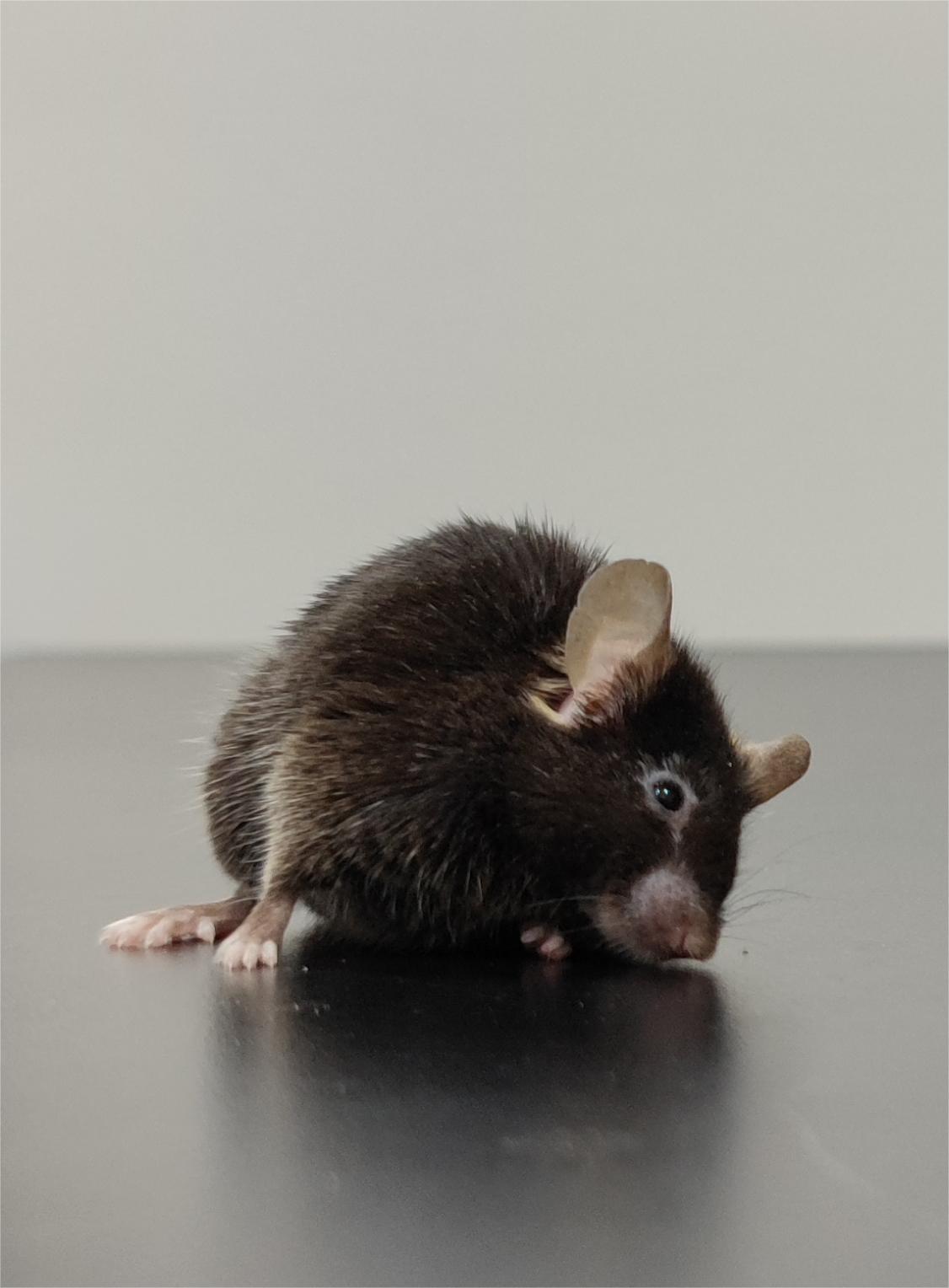

Supplement: Supplementary file 13 — Source data Fig. 6 [file 44318_2025_654_MOESM13_ESM.zip › Figure 6/6A/6A-2-KO-1.jpg]

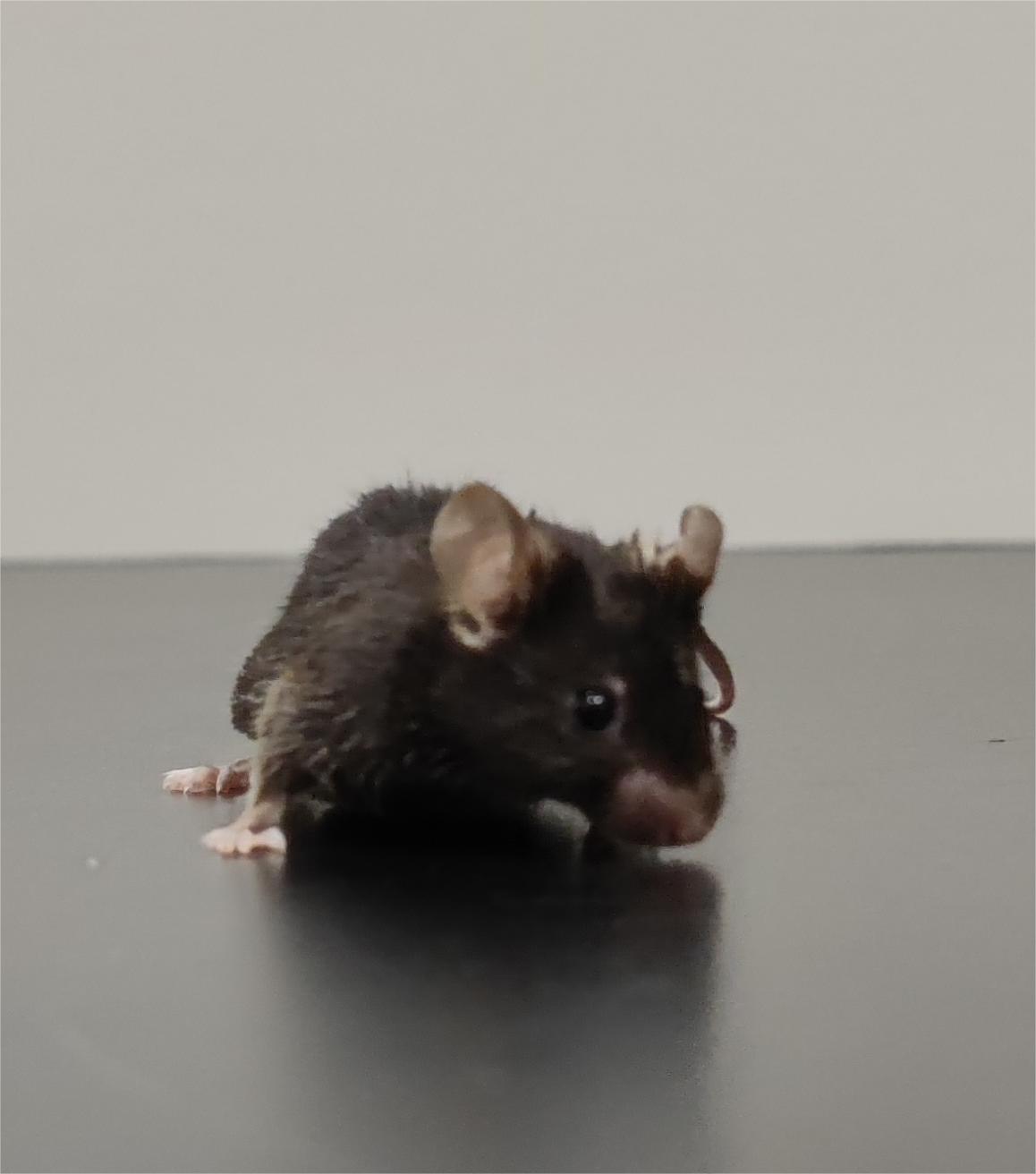

Supplement: Supplementary file 13 — Source data Fig. 6 [file 44318_2025_654_MOESM13_ESM.zip › Figure 6/6A/6A-1-WT-1.jpg]

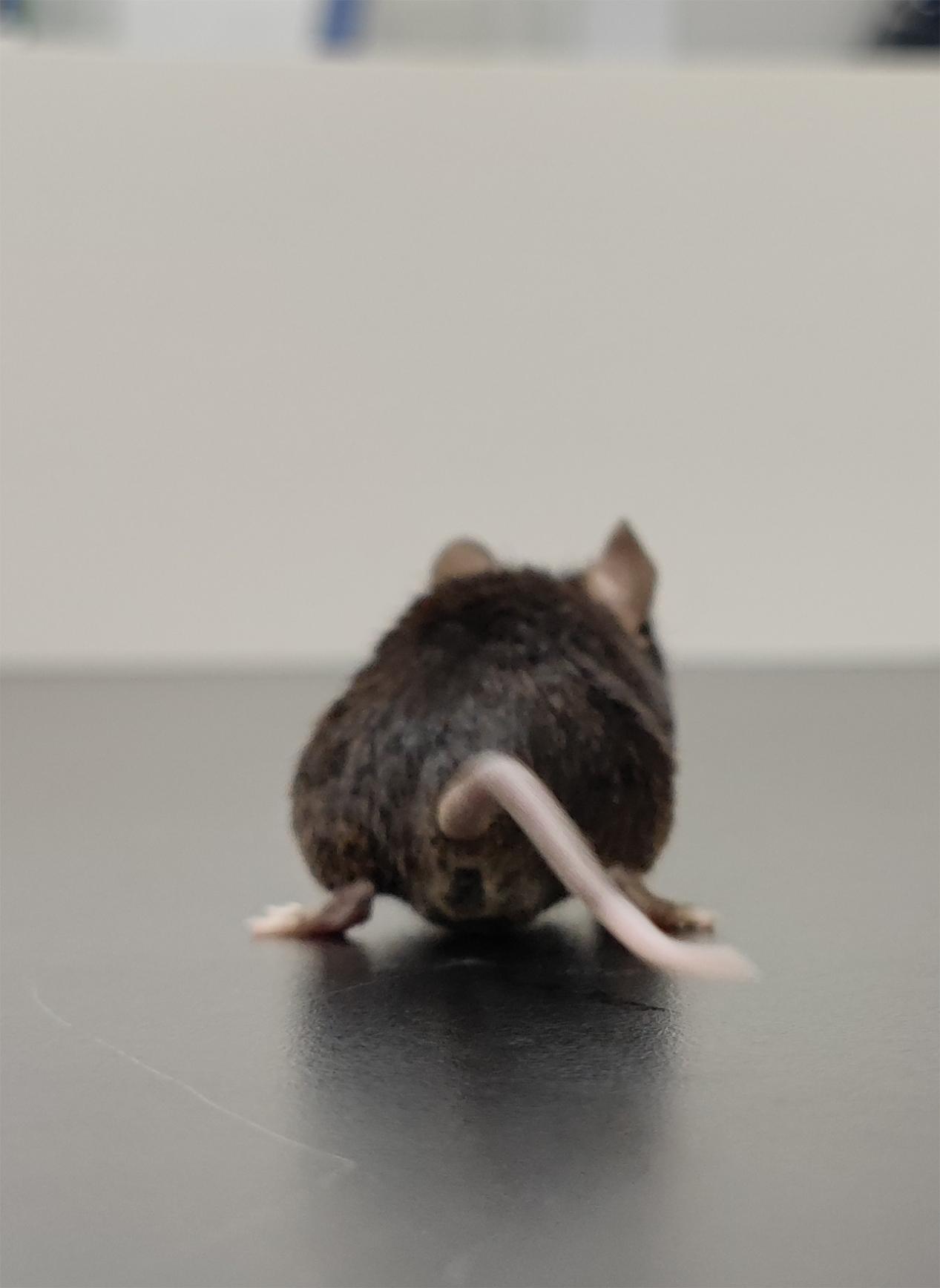

Supplement: Supplementary file 13 — Source data Fig. 6 [file 44318_2025_654_MOESM13_ESM.zip › Figure 6/6A/6A-1-WT-2.jpg]

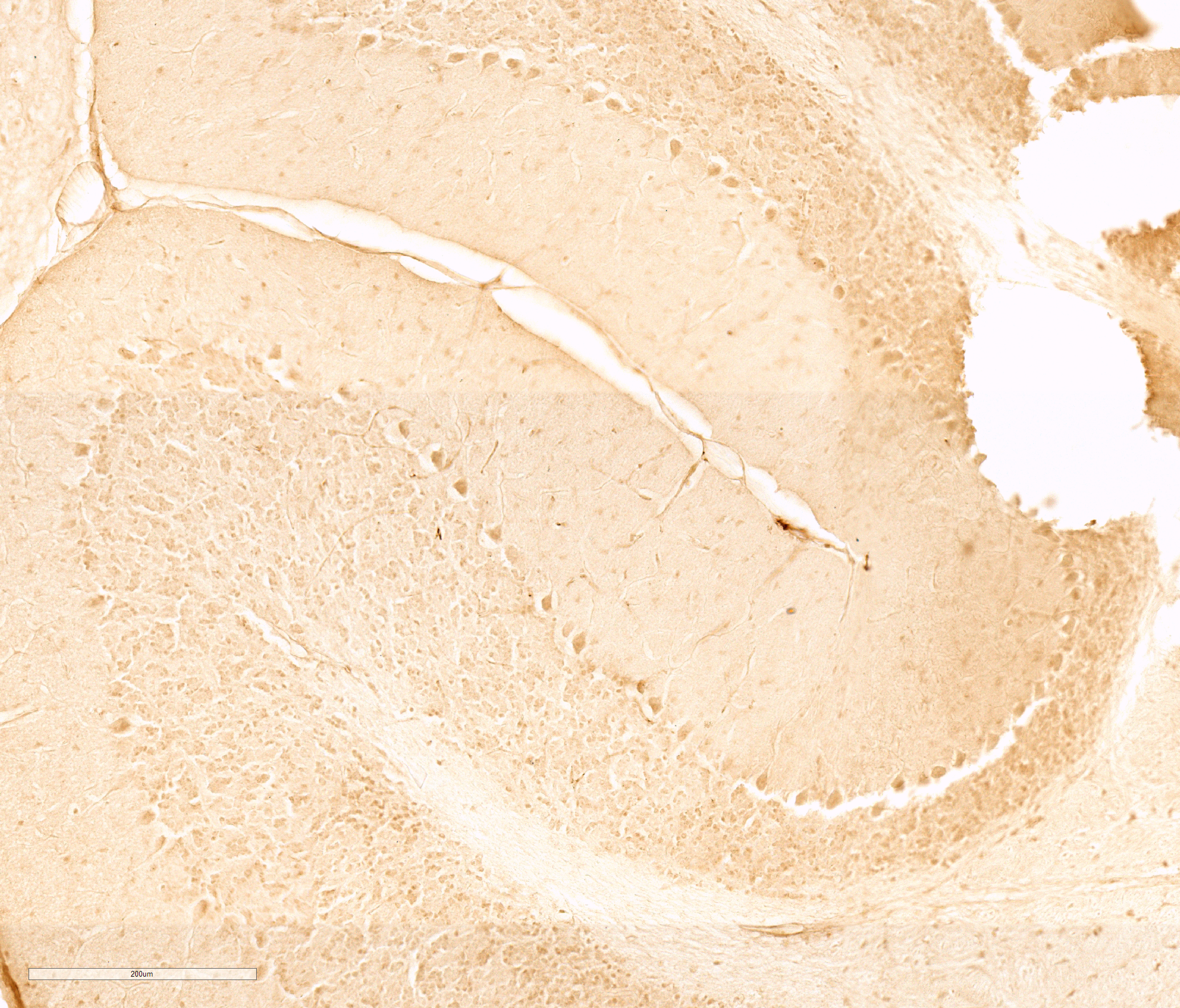

Supplement: Supplementary file 13 — Source data Fig. 6 [file 44318_2025_654_MOESM13_ESM.zip › Figure 6/6H/6H-1-WT.tif]

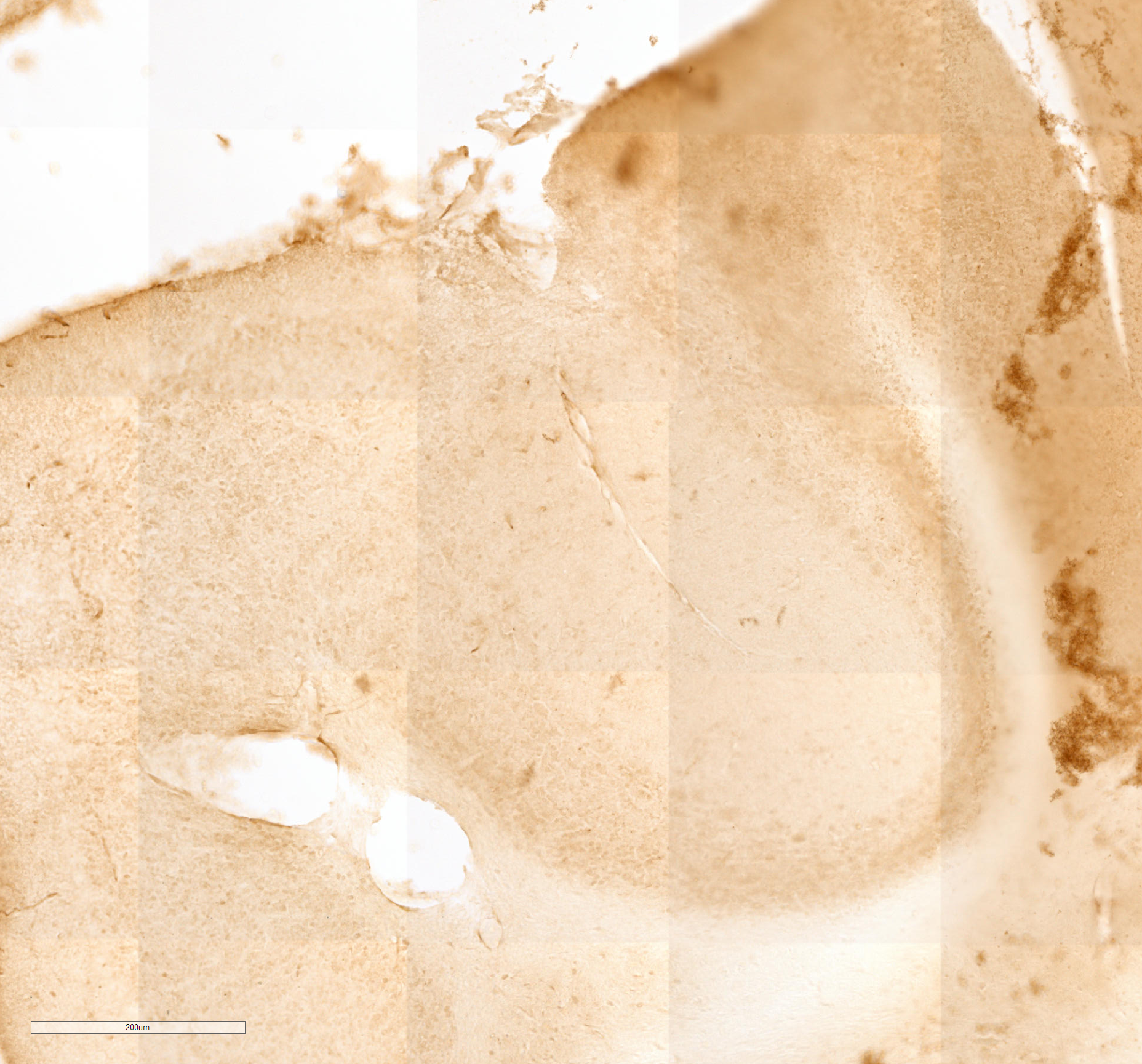

Supplement: Supplementary file 13 — Source data Fig. 6 [file 44318_2025_654_MOESM13_ESM.zip › Figure 6/6H/6H-2-KO.tif]

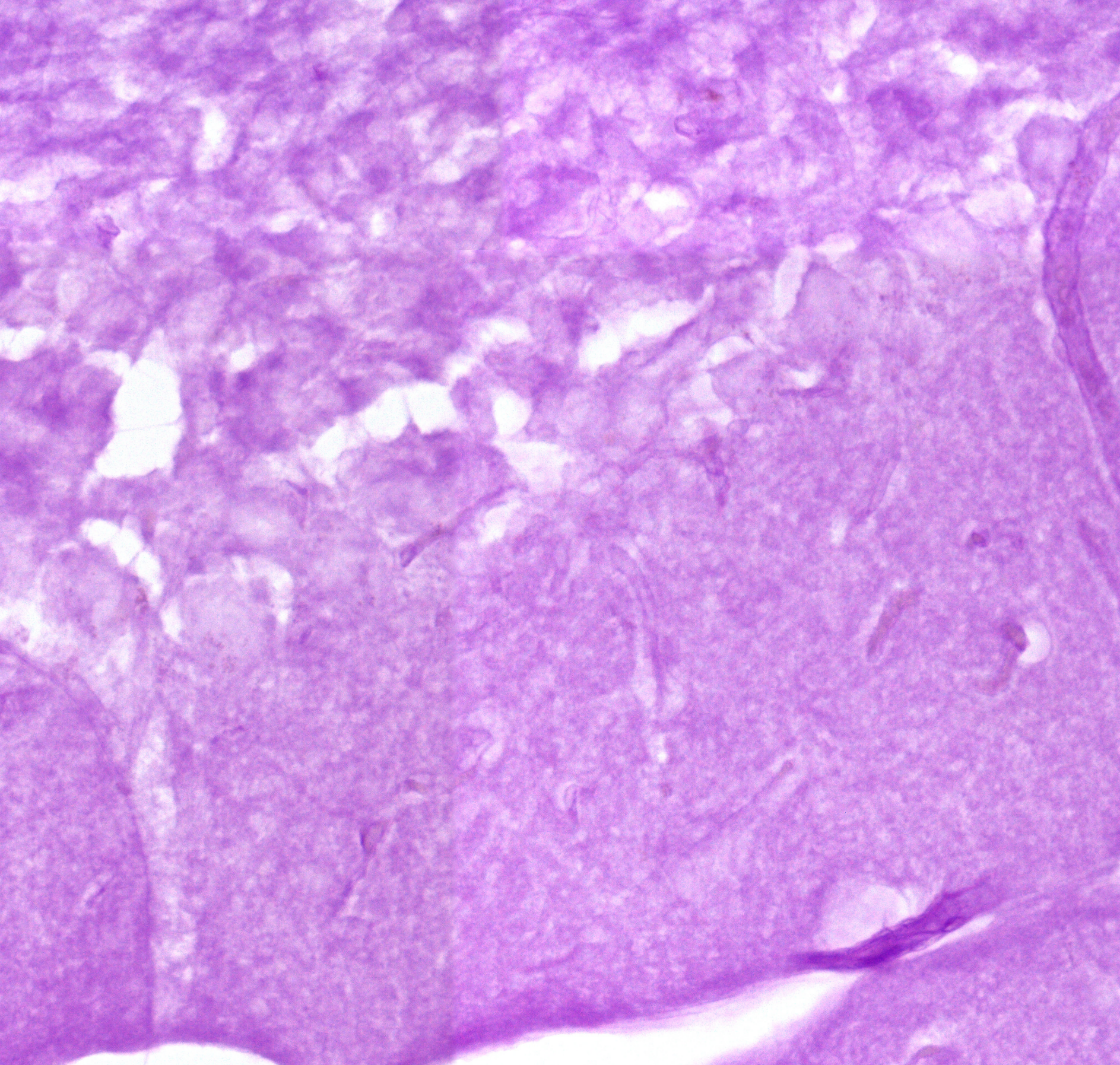

Supplement: Supplementary file 13 — Source data Fig. 6 [file 44318_2025_654_MOESM13_ESM.zip › Figure 6/6R/6R-1-WT zoom in-1.tif]

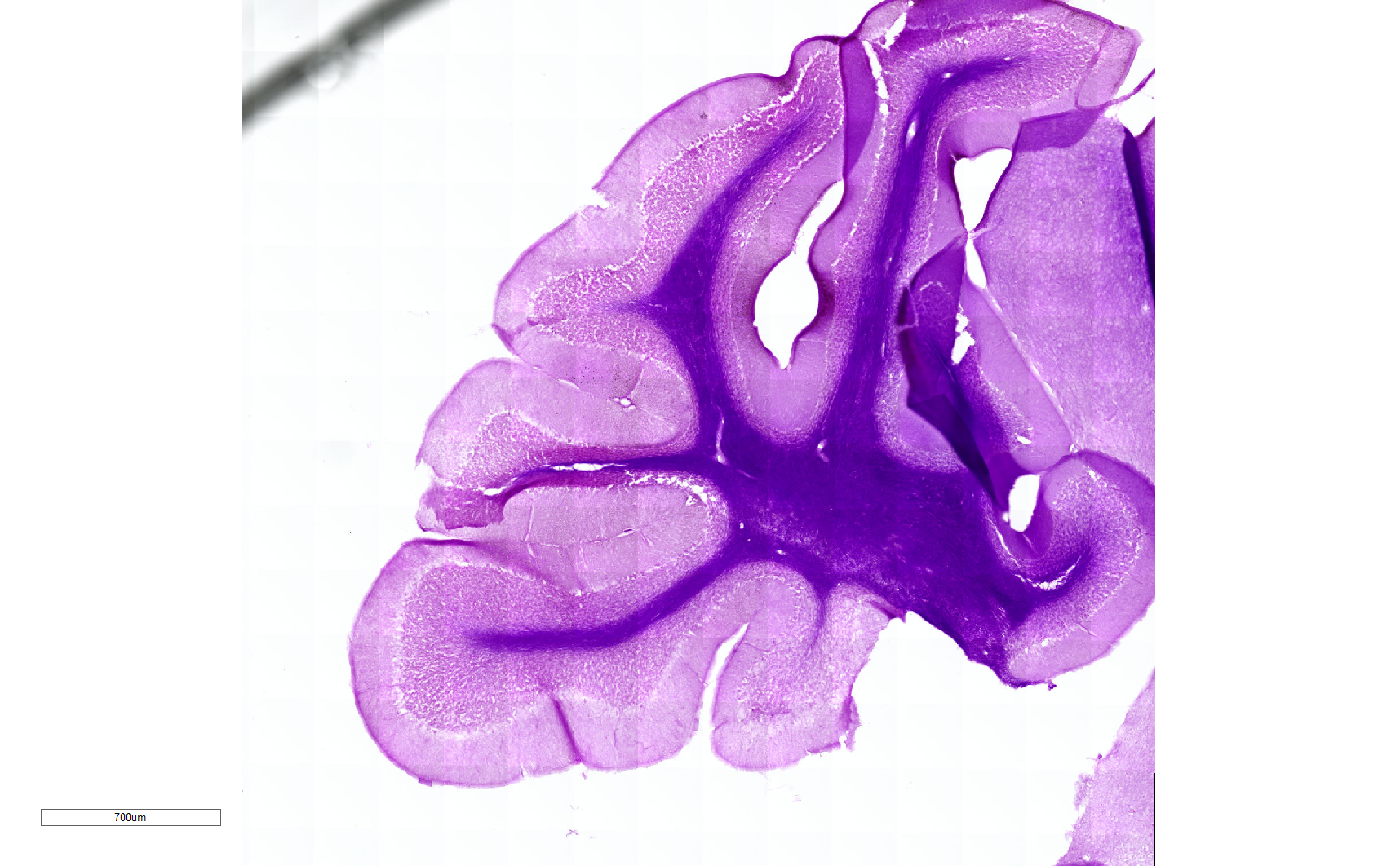

Supplement: Supplementary file 13 — Source data Fig. 6 [file 44318_2025_654_MOESM13_ESM.zip › Figure 6/6R/6R-2-KO.tif]

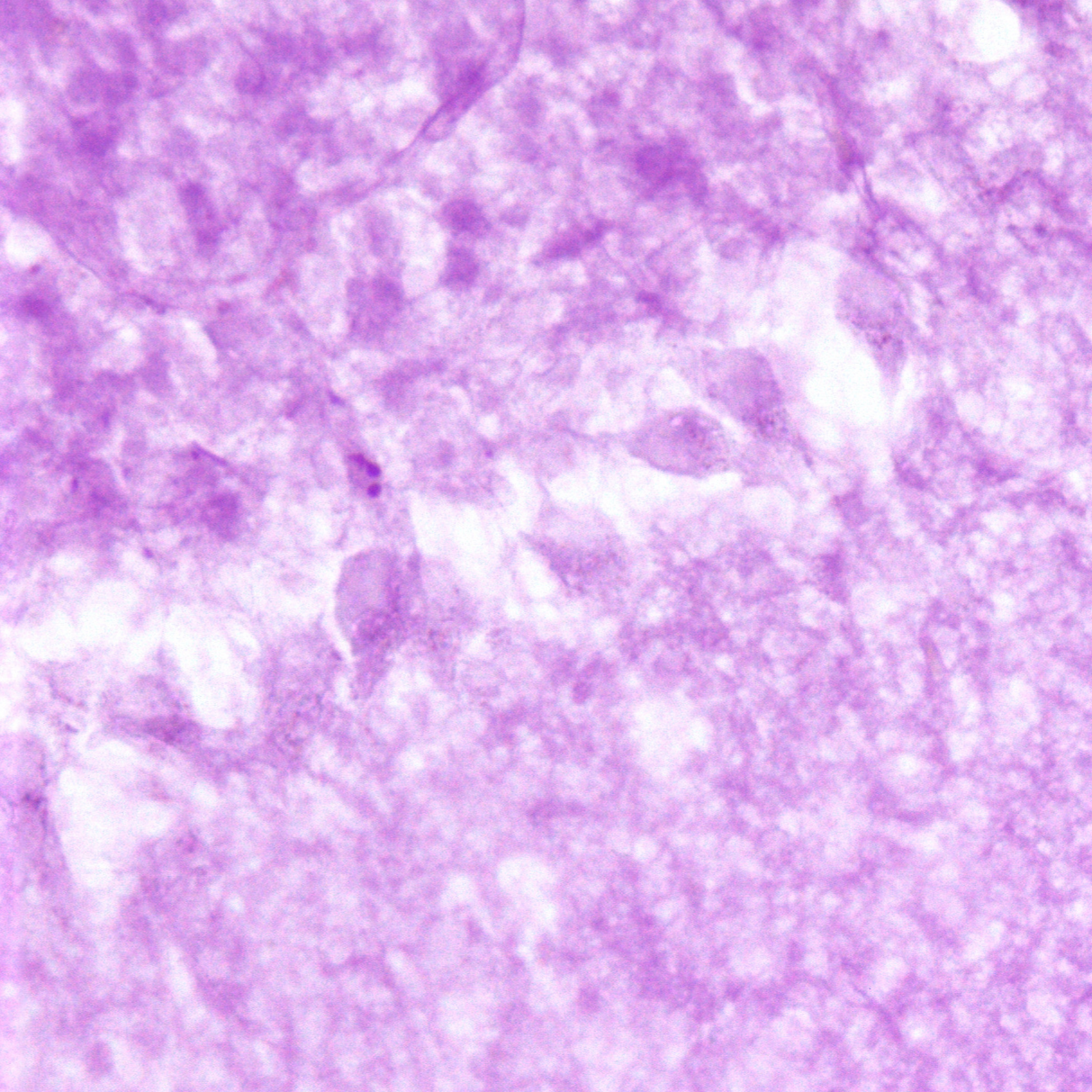

Supplement: Supplementary file 13 — Source data Fig. 6 [file 44318_2025_654_MOESM13_ESM.zip › Figure 6/6R/6R-1-WT zoom in-3.tif]

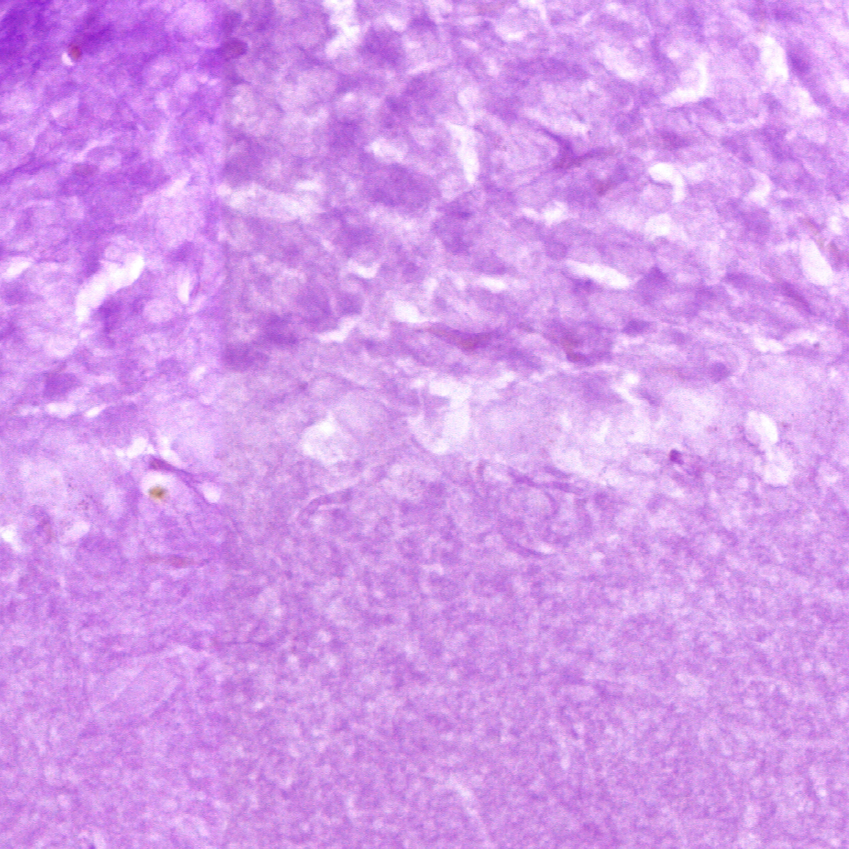

Supplement: Supplementary file 13 — Source data Fig. 6 [file 44318_2025_654_MOESM13_ESM.zip › Figure 6/6R/6R-1-WT zoom in-2.tif]

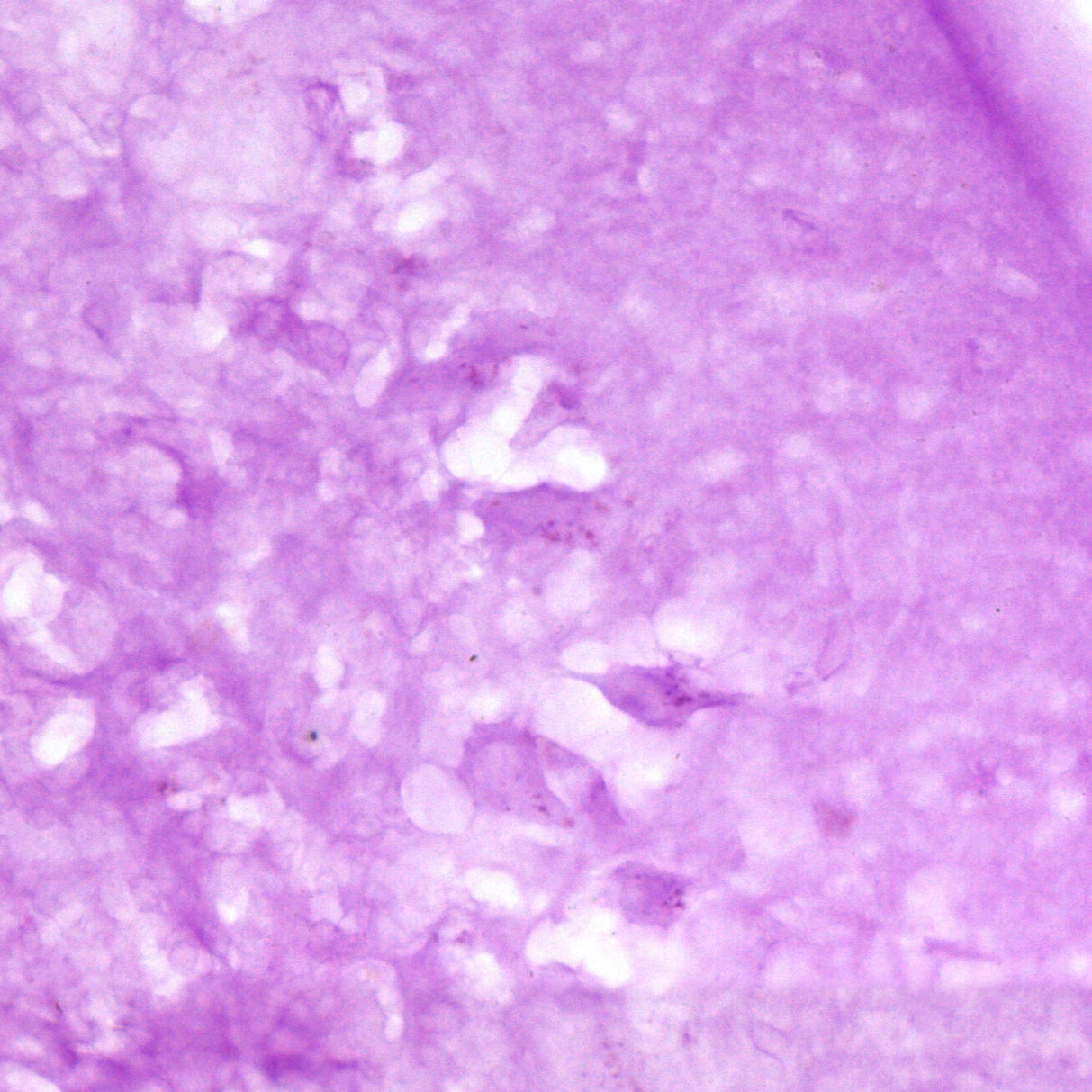

Supplement: Supplementary file 13 — Source data Fig. 6 [file 44318_2025_654_MOESM13_ESM.zip › Figure 6/6R/6R-2-KO zoom in-1.tif]

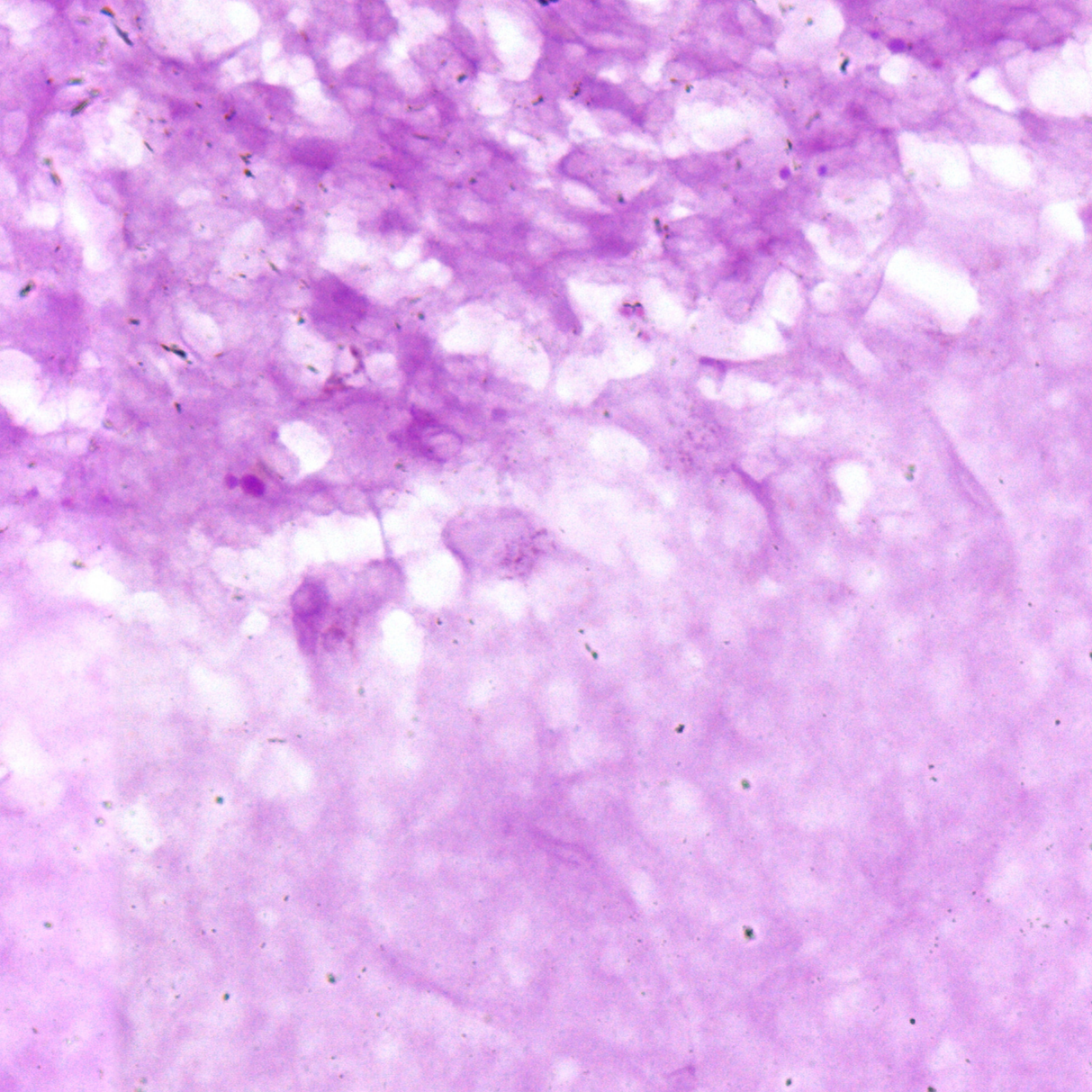

Supplement: Supplementary file 13 — Source data Fig. 6 [file 44318_2025_654_MOESM13_ESM.zip › Figure 6/6R/6R-2-KO zoom in-3.tif]

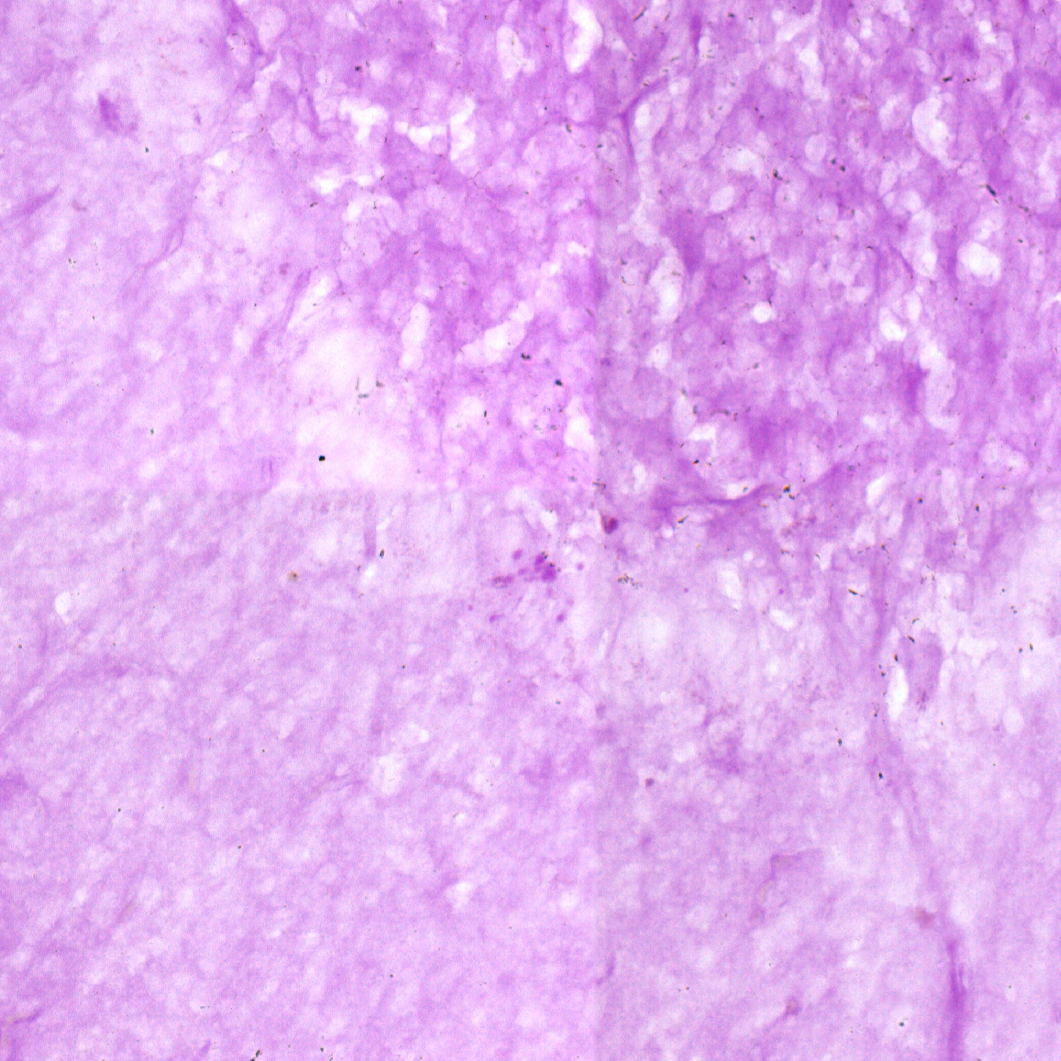

Supplement: Supplementary file 13 — Source data Fig. 6 [file 44318_2025_654_MOESM13_ESM.zip › Figure 6/6R/6R-2-KO zoom in-2.tif]

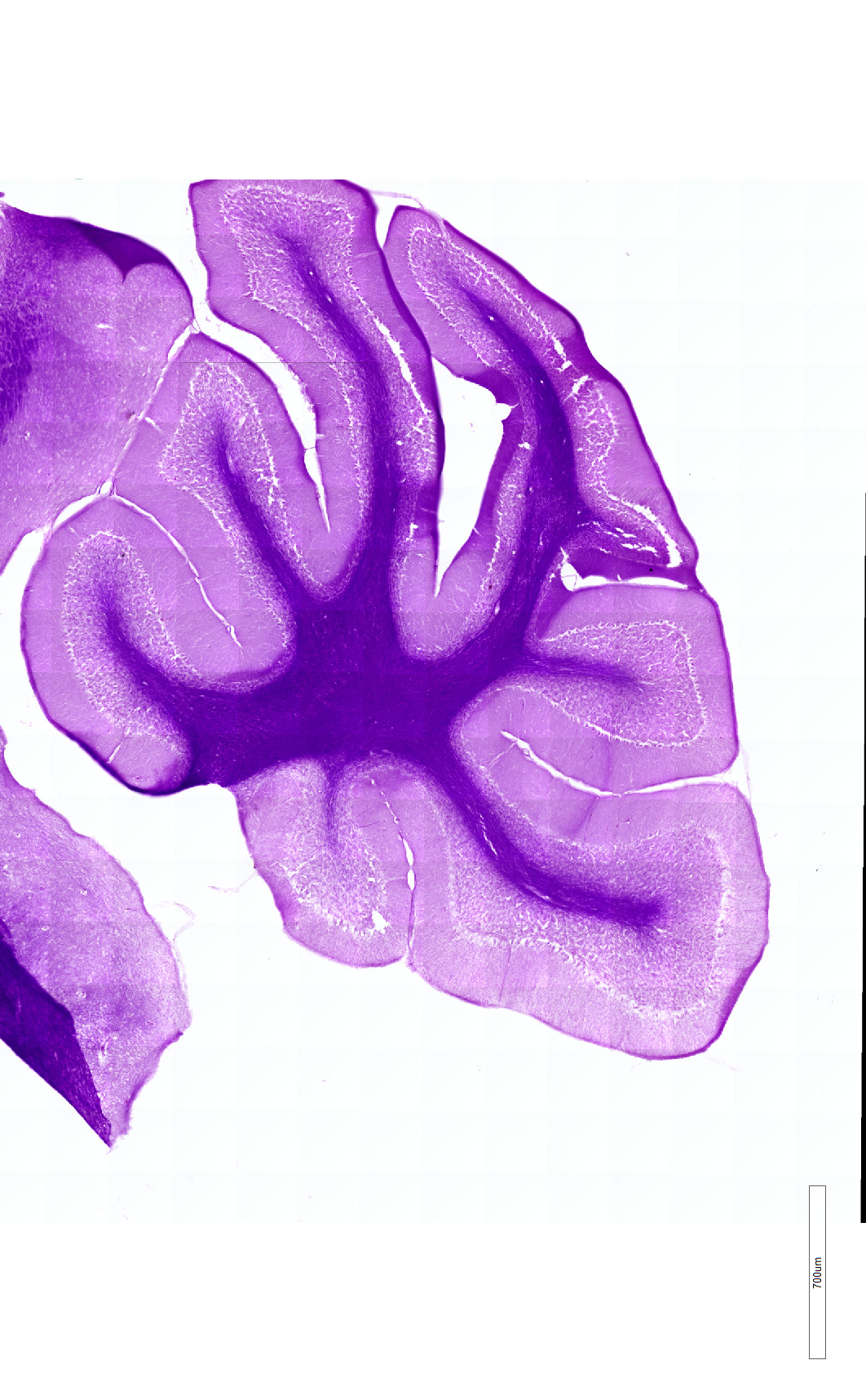

Supplement: Supplementary file 13 — Source data Fig. 6 [file 44318_2025_654_MOESM13_ESM.zip › Figure 6/6R/6R-1-WT.tif]

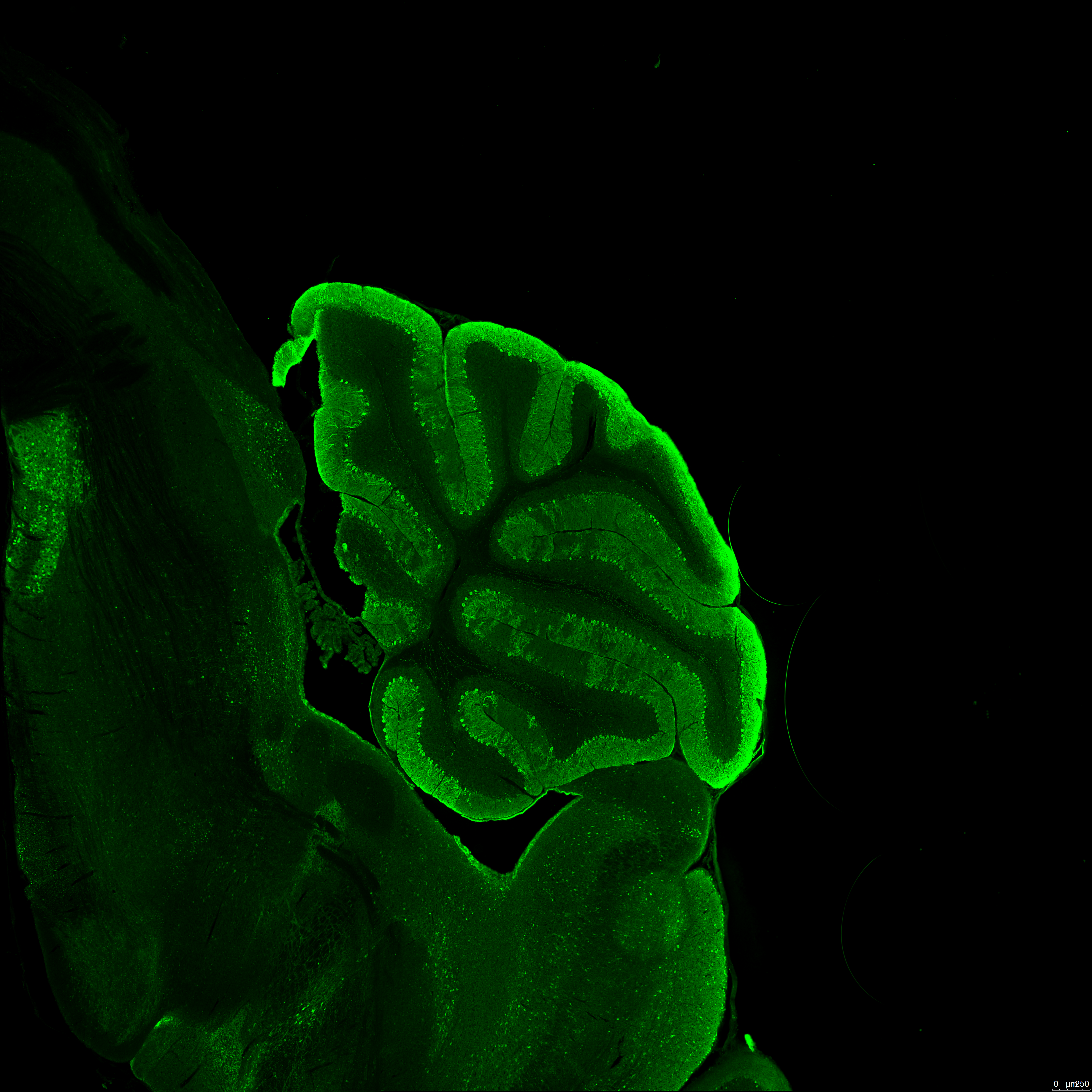

Supplement: Supplementary file 13 — Source data Fig. 6 [file 44318_2025_654_MOESM13_ESM.zip › Figure 6/6I/6I-2-12 month old male calbindin-KO.tif]

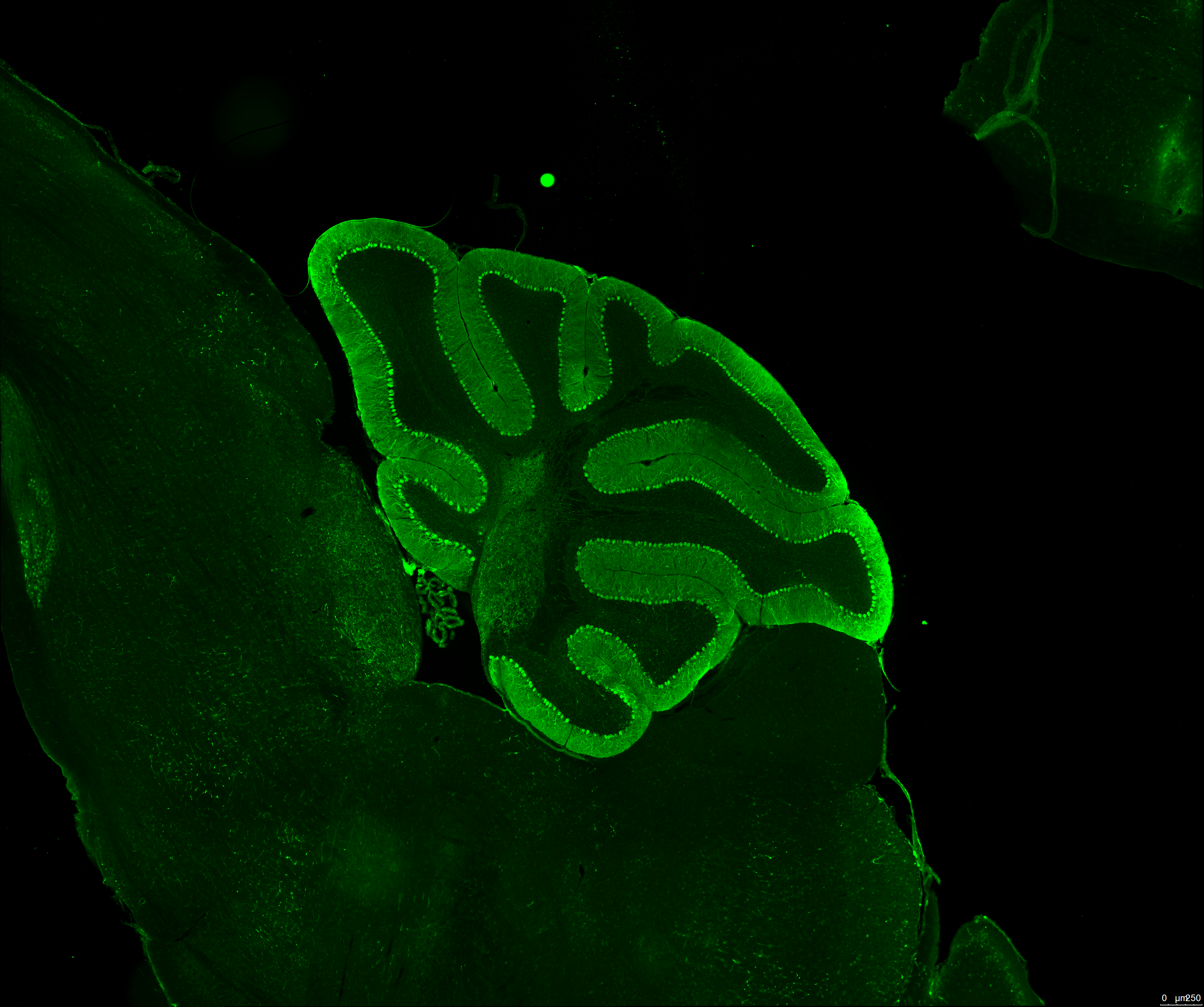

Supplement: Supplementary file 13 — Source data Fig. 6 [file 44318_2025_654_MOESM13_ESM.zip › Figure 6/6I/6I-1-12 month old male calbindin-WT.tif]

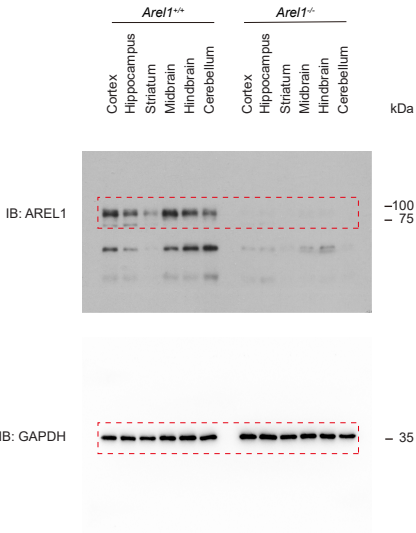

Supplement: Supplementary file 13 — Source data Fig. 6 [file 44318_2025_654_MOESM13_ESM.zip › Figure 6/6G/6G.pdf]

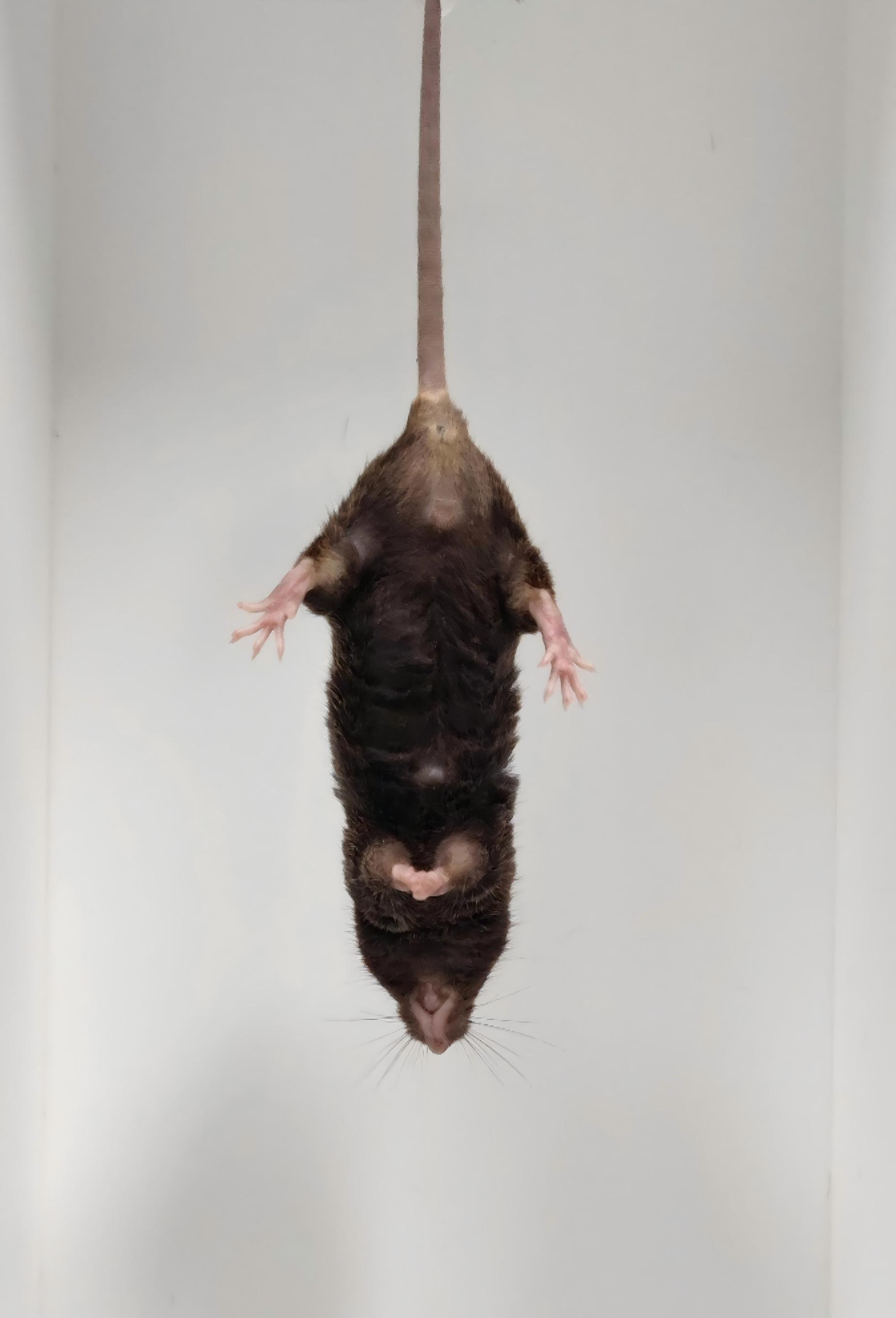

Supplement: Supplementary file 13 — Source data Fig. 6 [file 44318_2025_654_MOESM13_ESM.zip › Figure 6/6B/6B-1-WT.jpg]

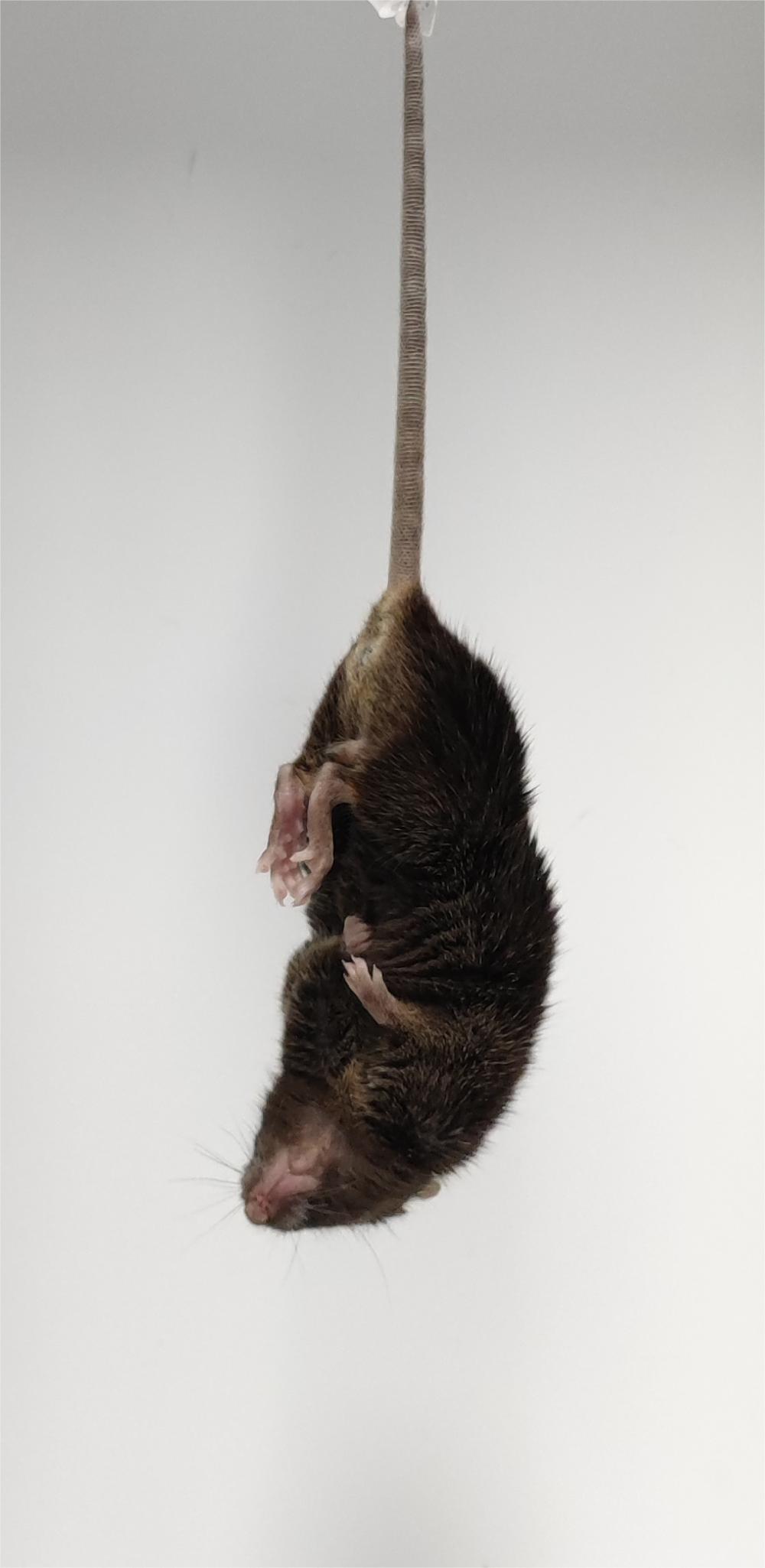

Supplement: Supplementary file 13 — Source data Fig. 6 [file 44318_2025_654_MOESM13_ESM.zip › Figure 6/6B/6B-2-KO.jpg]

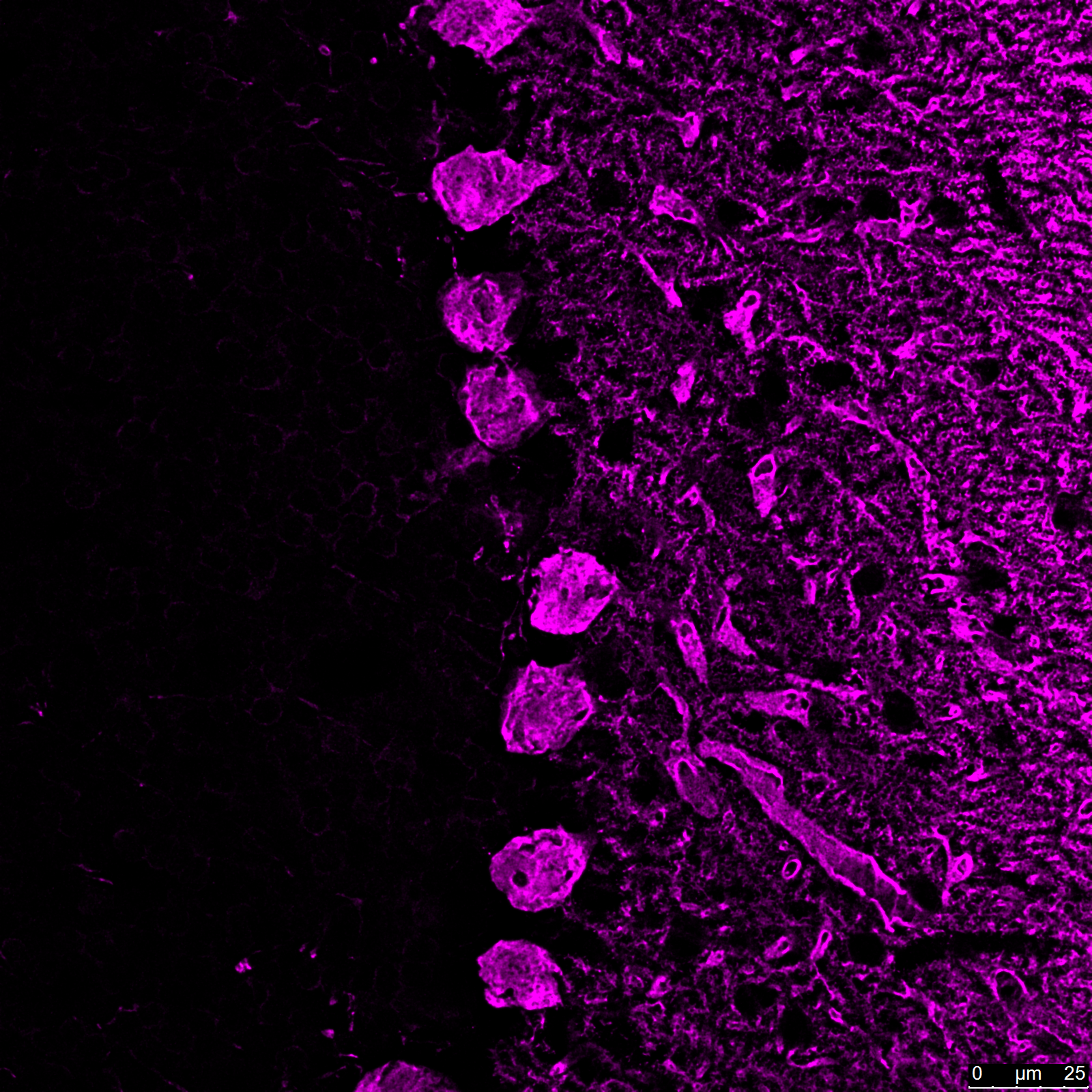

Supplement: Supplementary file 13 — Source data Fig. 6 [file 44318_2025_654_MOESM13_ESM.zip › Figure 6/6L/6L-2-12 month old female-KO calbindin.tif]

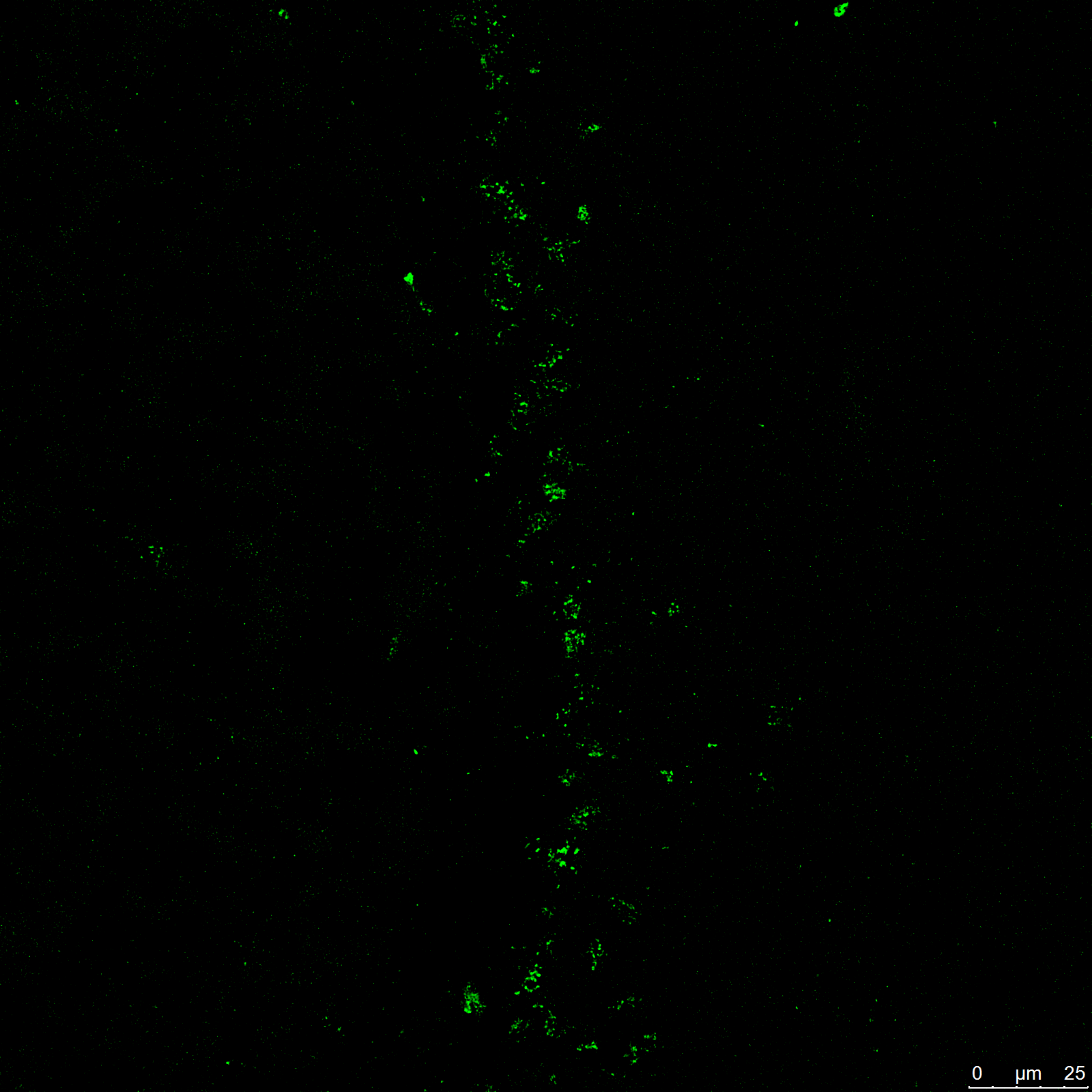

Supplement: Supplementary file 13 — Source data Fig. 6 [file 44318_2025_654_MOESM13_ESM.zip › Figure 6/6L/6L-2-12 month old female-KO lipofuscin.tif]

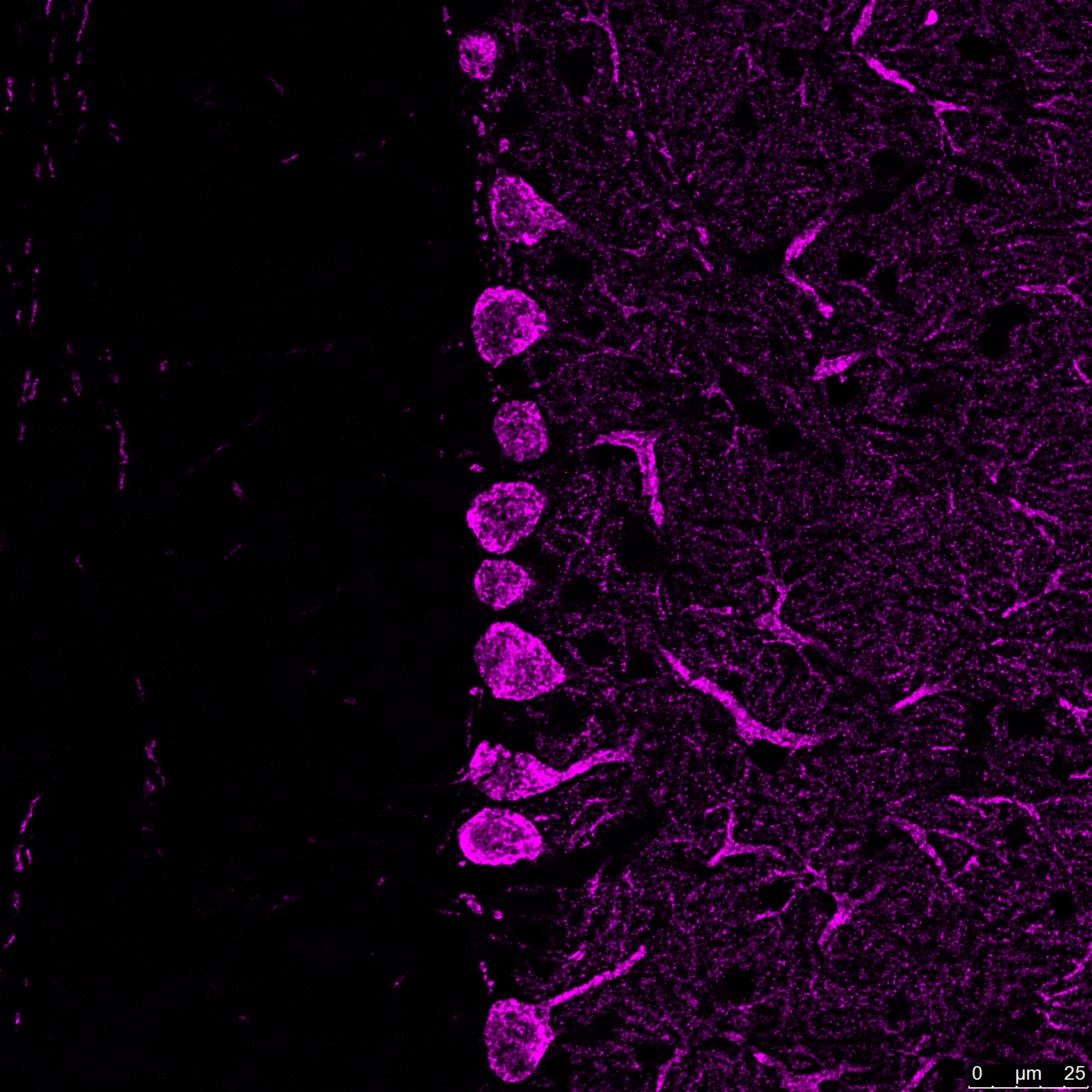

Supplement: Supplementary file 13 — Source data Fig. 6 [file 44318_2025_654_MOESM13_ESM.zip › Figure 6/6L/6L-1-12 month old female-WT calbindin.tif]

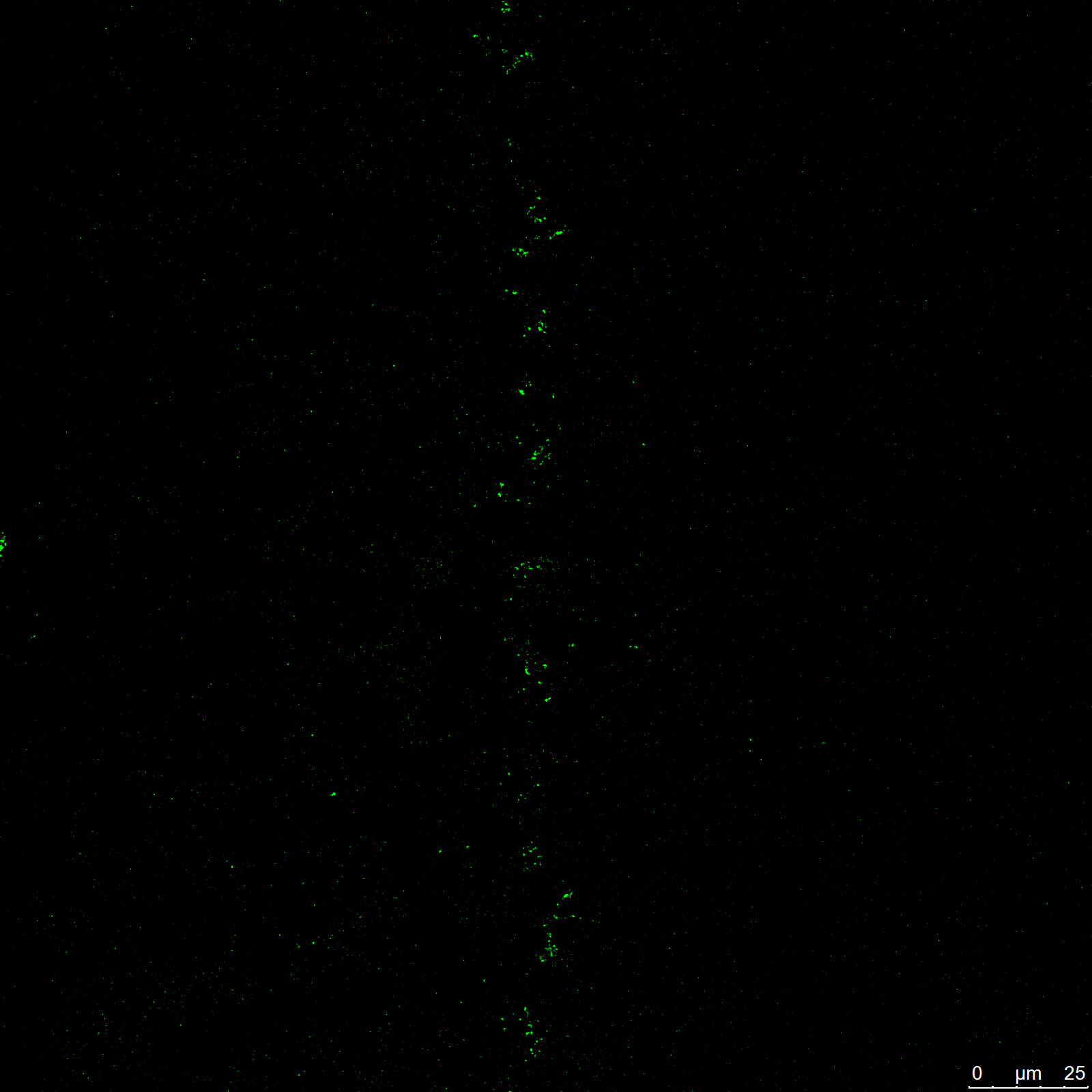

Supplement: Supplementary file 13 — Source data Fig. 6 [file 44318_2025_654_MOESM13_ESM.zip › Figure 6/6L/6L-1-12 month old female-WT lipofuscin.tif]

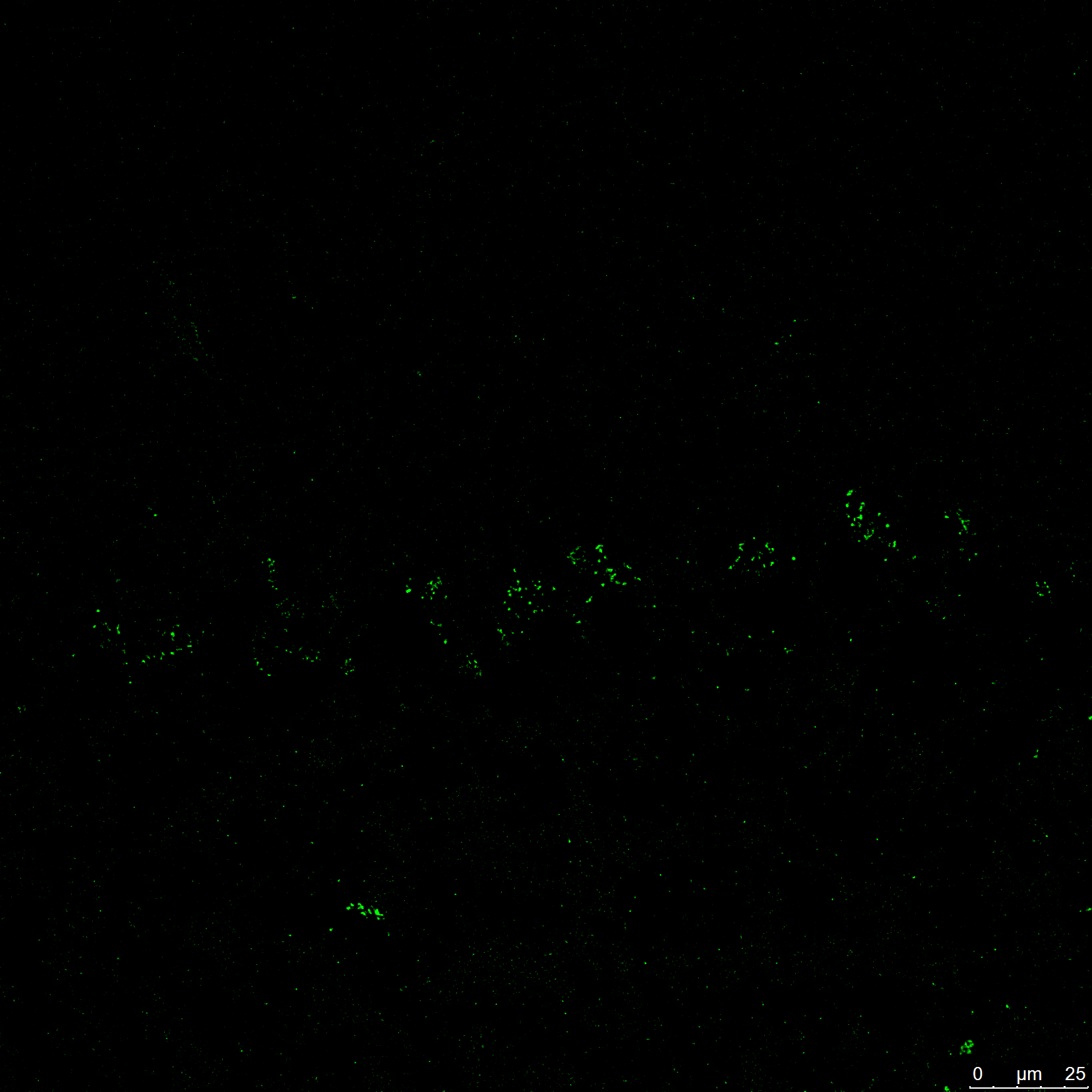

Supplement: Supplementary file 13 — Source data Fig. 6 [file 44318_2025_654_MOESM13_ESM.zip › Figure 6/6K/6K-1-12 month old male-WT lipofuscin.tif]

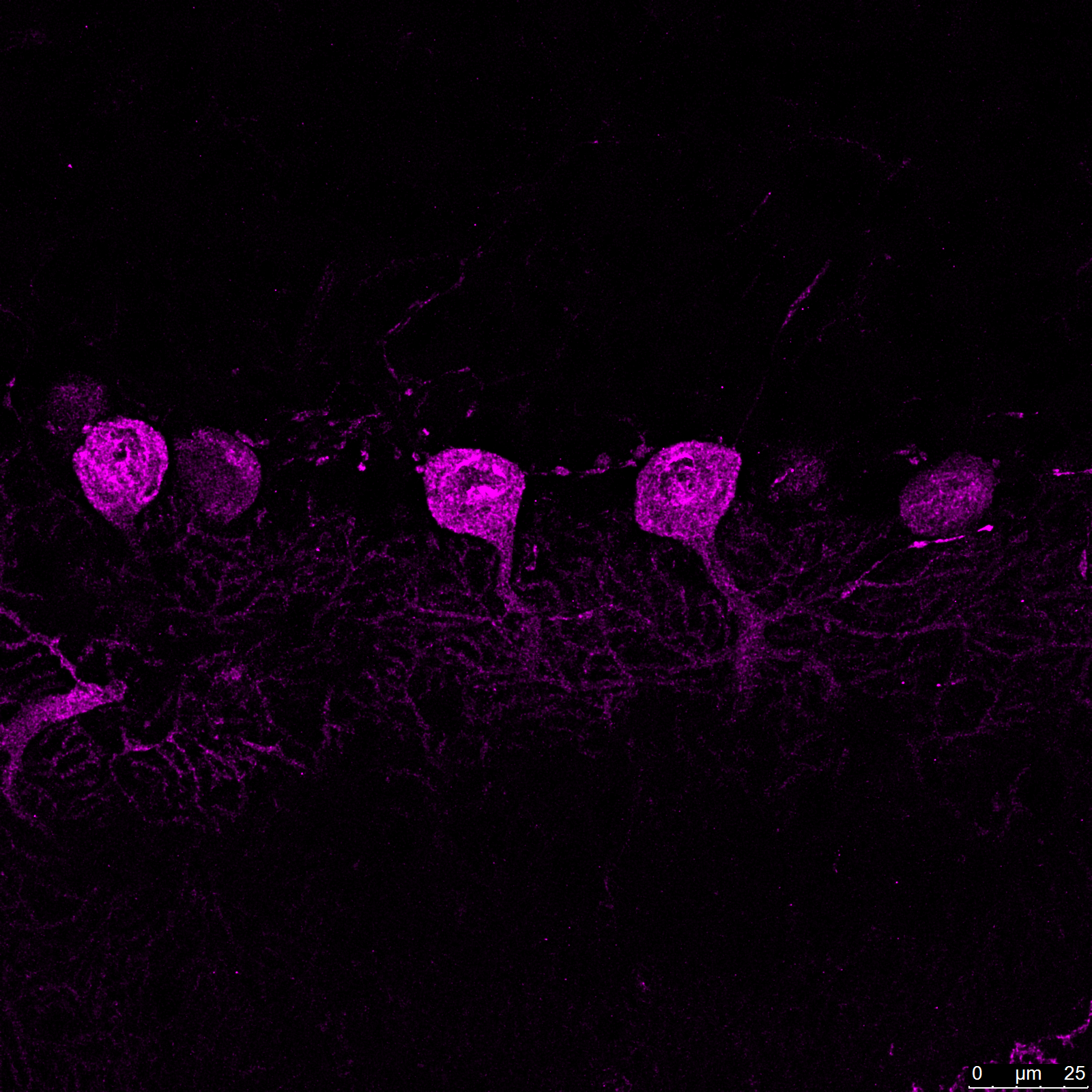

Supplement: Supplementary file 13 — Source data Fig. 6 [file 44318_2025_654_MOESM13_ESM.zip › Figure 6/6K/6K-2-12 month old male-KO calbindin.tif]

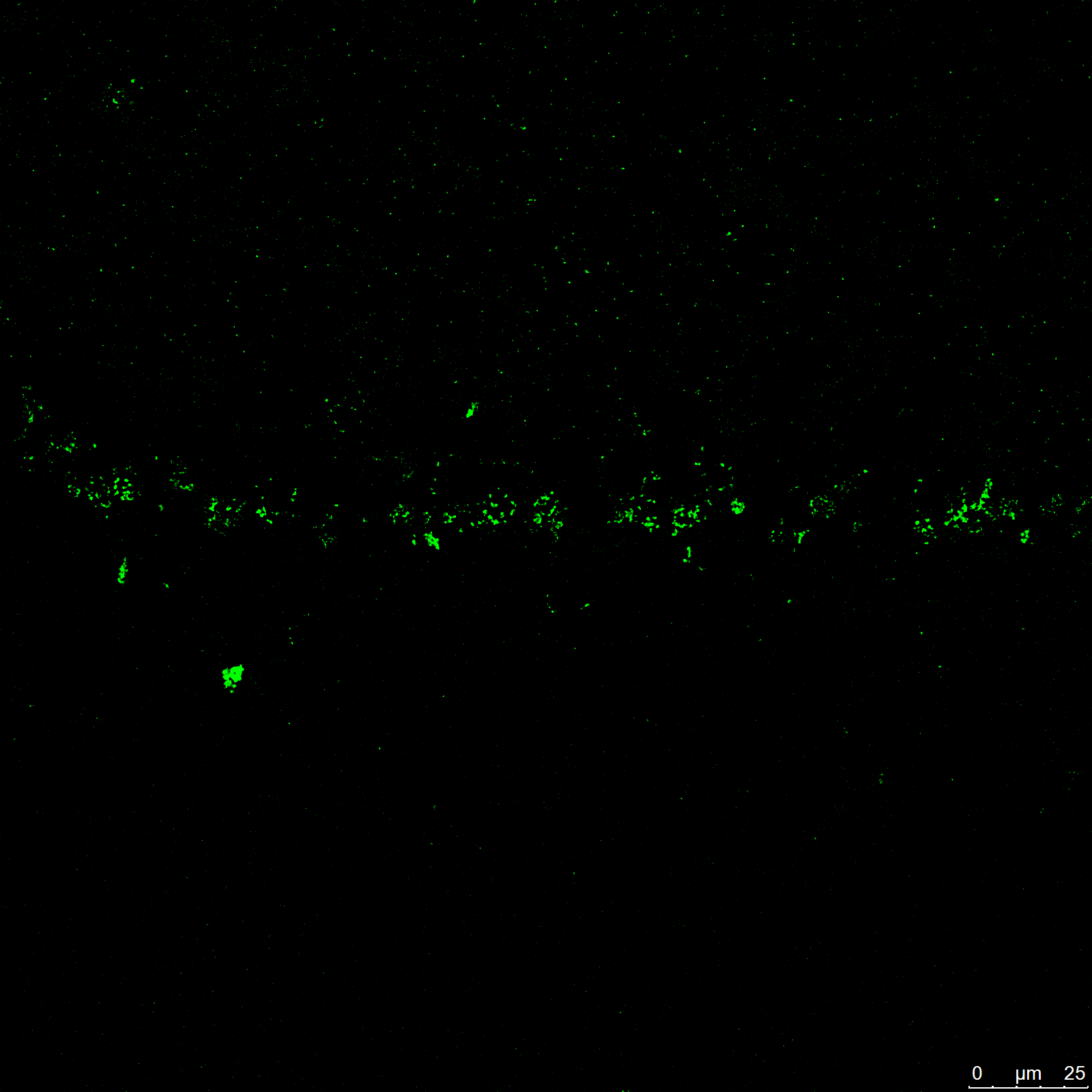

Supplement: Supplementary file 13 — Source data Fig. 6 [file 44318_2025_654_MOESM13_ESM.zip › Figure 6/6K/6K-2-12 month old male-KO lipofuscin.tif]

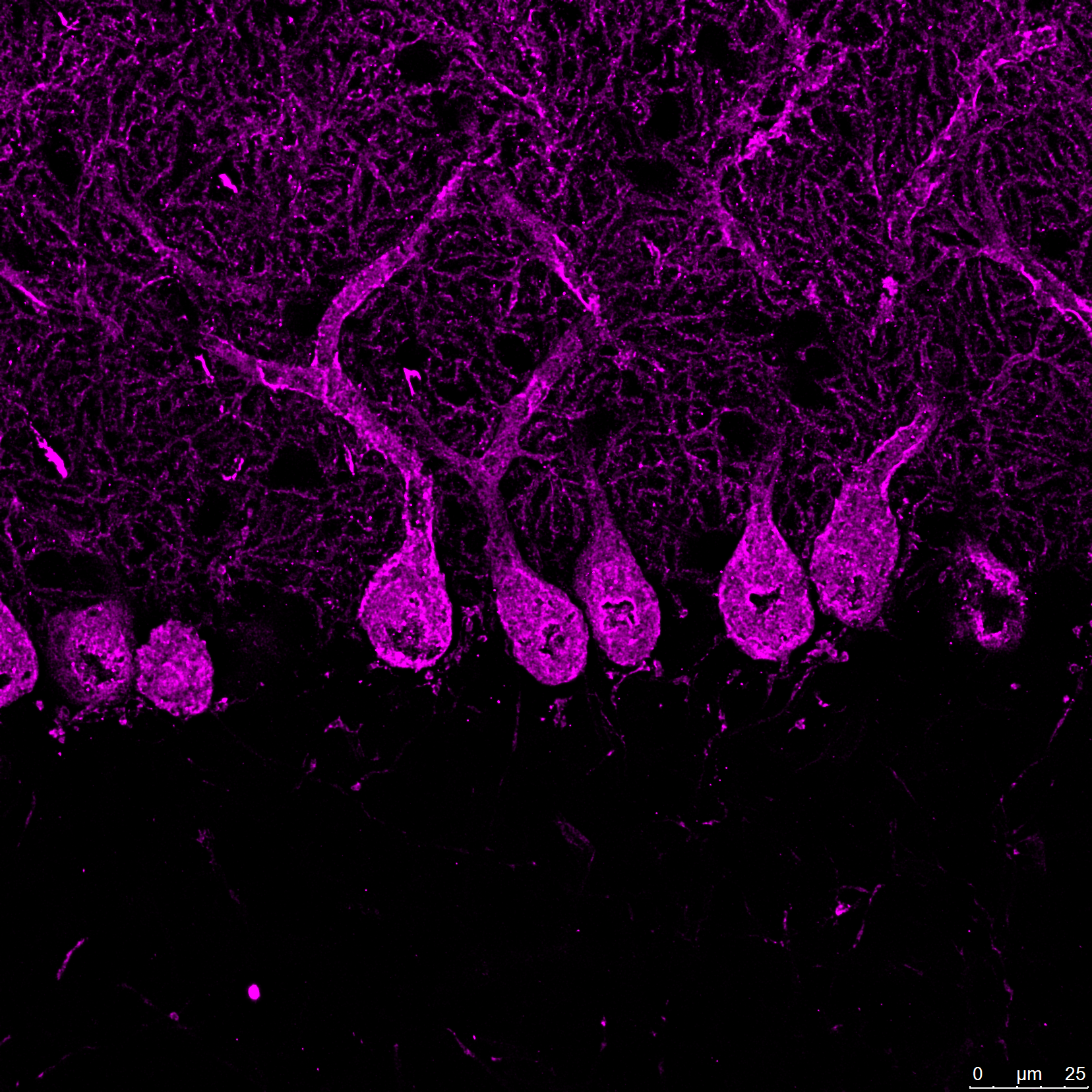

Supplement: Supplementary file 13 — Source data Fig. 6 [file 44318_2025_654_MOESM13_ESM.zip › Figure 6/6K/6K-1-12 month old male-WT calbindin.tif]

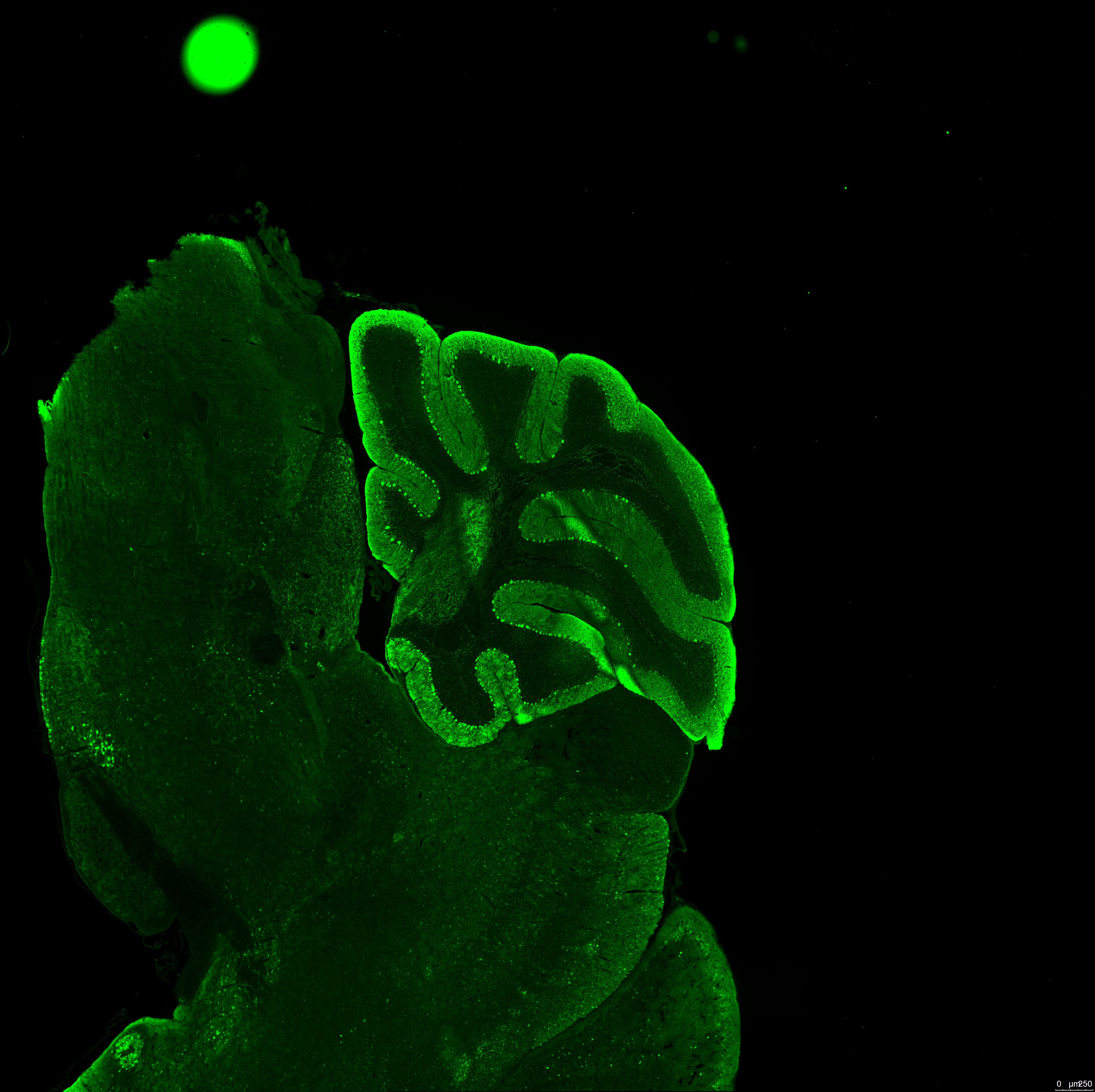

Supplement: Supplementary file 13 — Source data Fig. 6 [file 44318_2025_654_MOESM13_ESM.zip › Figure 6/6J/6J-2-12 month old female calbindin-KO.tif]

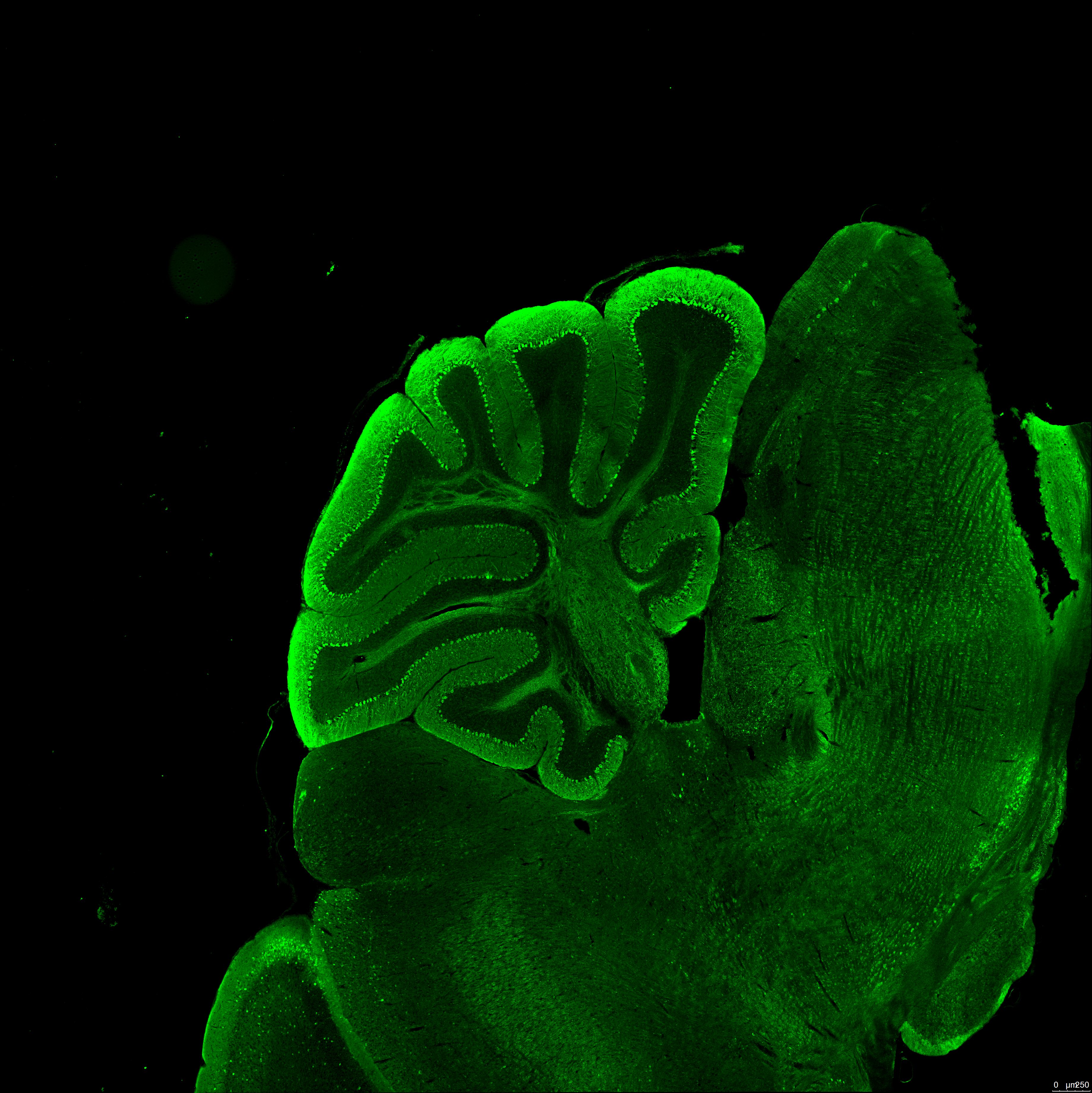

Supplement: Supplementary file 13 — Source data Fig. 6 [file 44318_2025_654_MOESM13_ESM.zip › Figure 6/6J/6J-1-12 month old female calbindin-WT.tif]

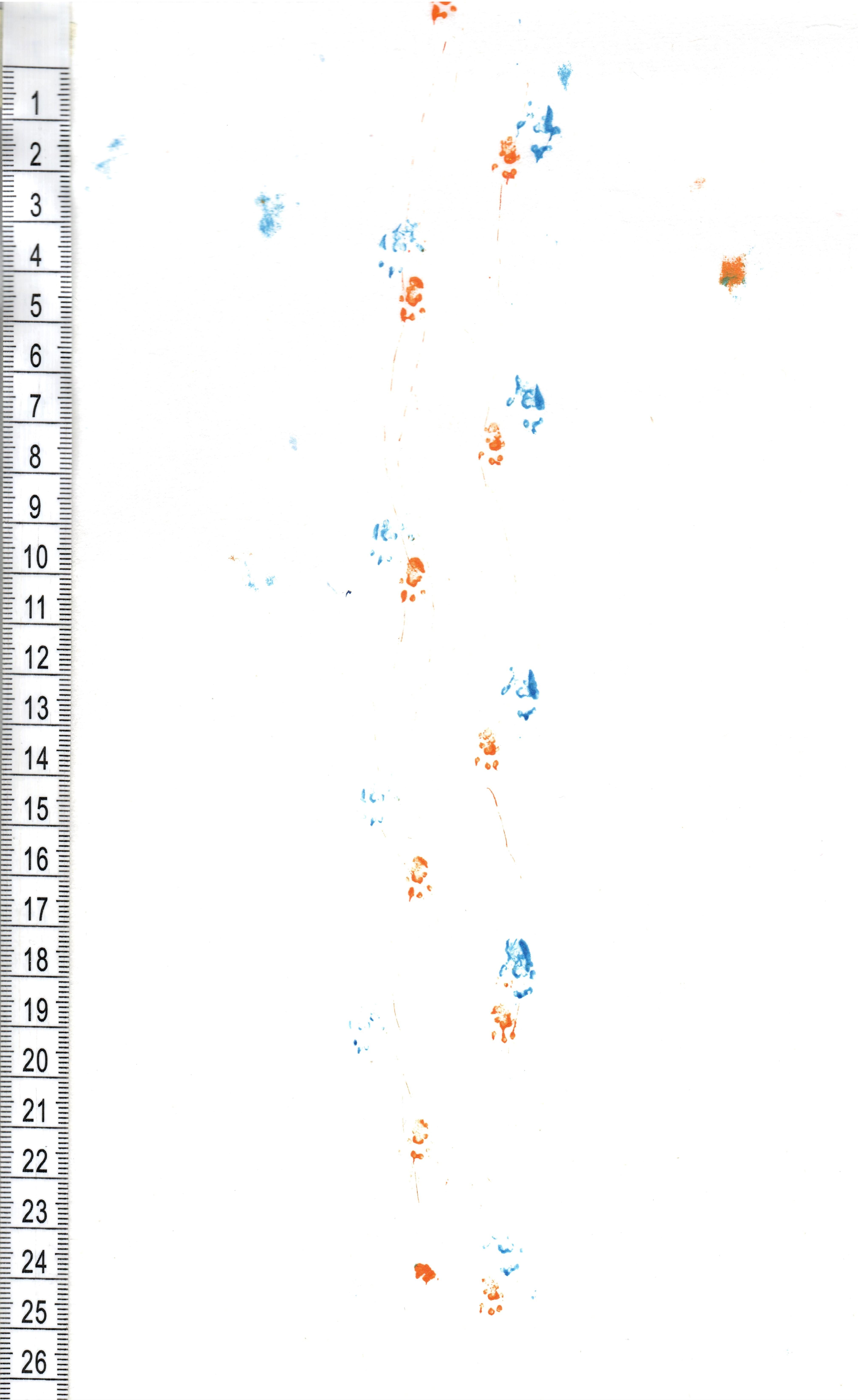

Supplement: Supplementary file 13 — Source data Fig. 6 [file 44318_2025_654_MOESM13_ESM.zip › Figure 6/6D/6D-2-male-KO.jpg]

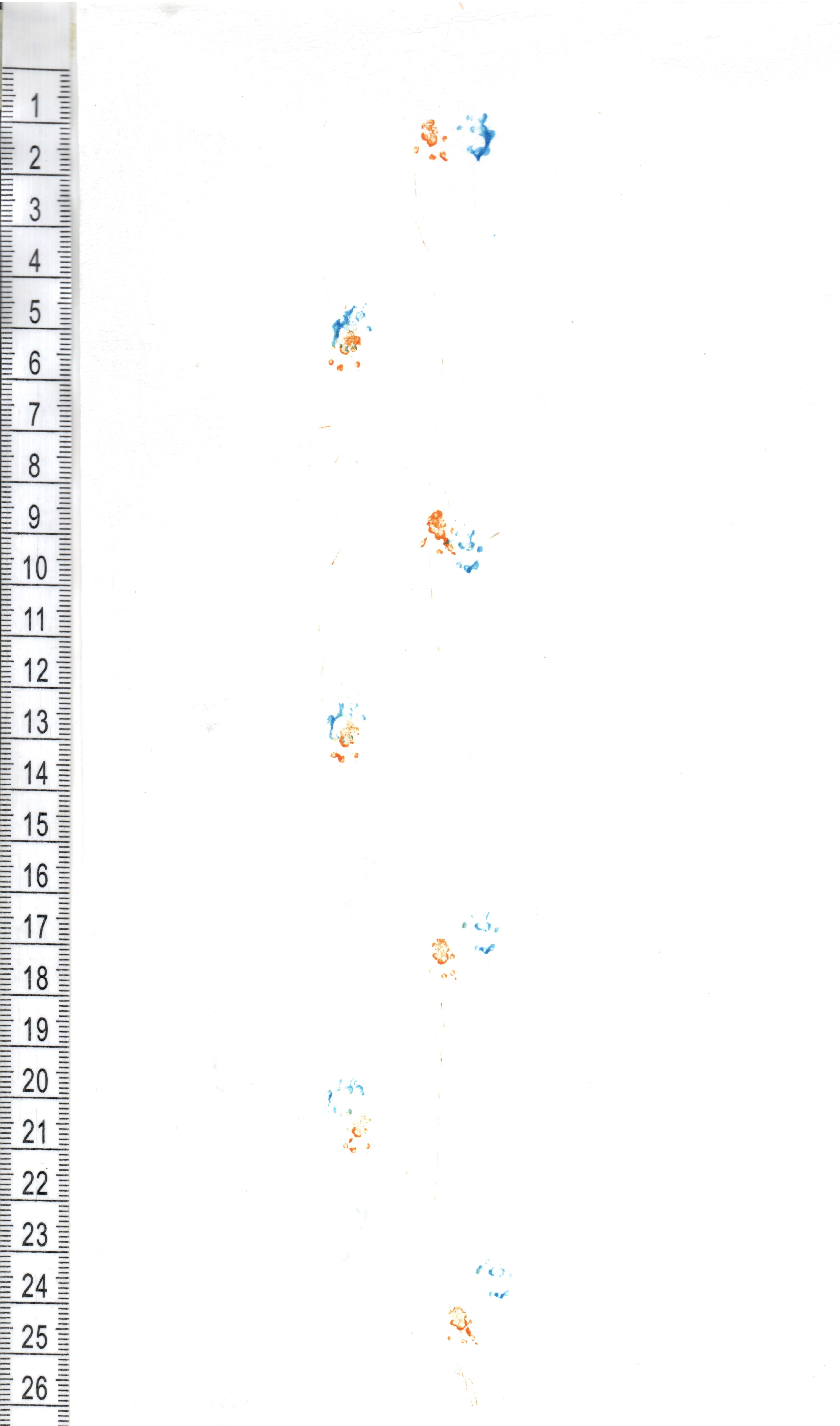

Supplement: Supplementary file 13 — Source data Fig. 6 [file 44318_2025_654_MOESM13_ESM.zip › Figure 6/6D/6D-1-male-WT.jpg]

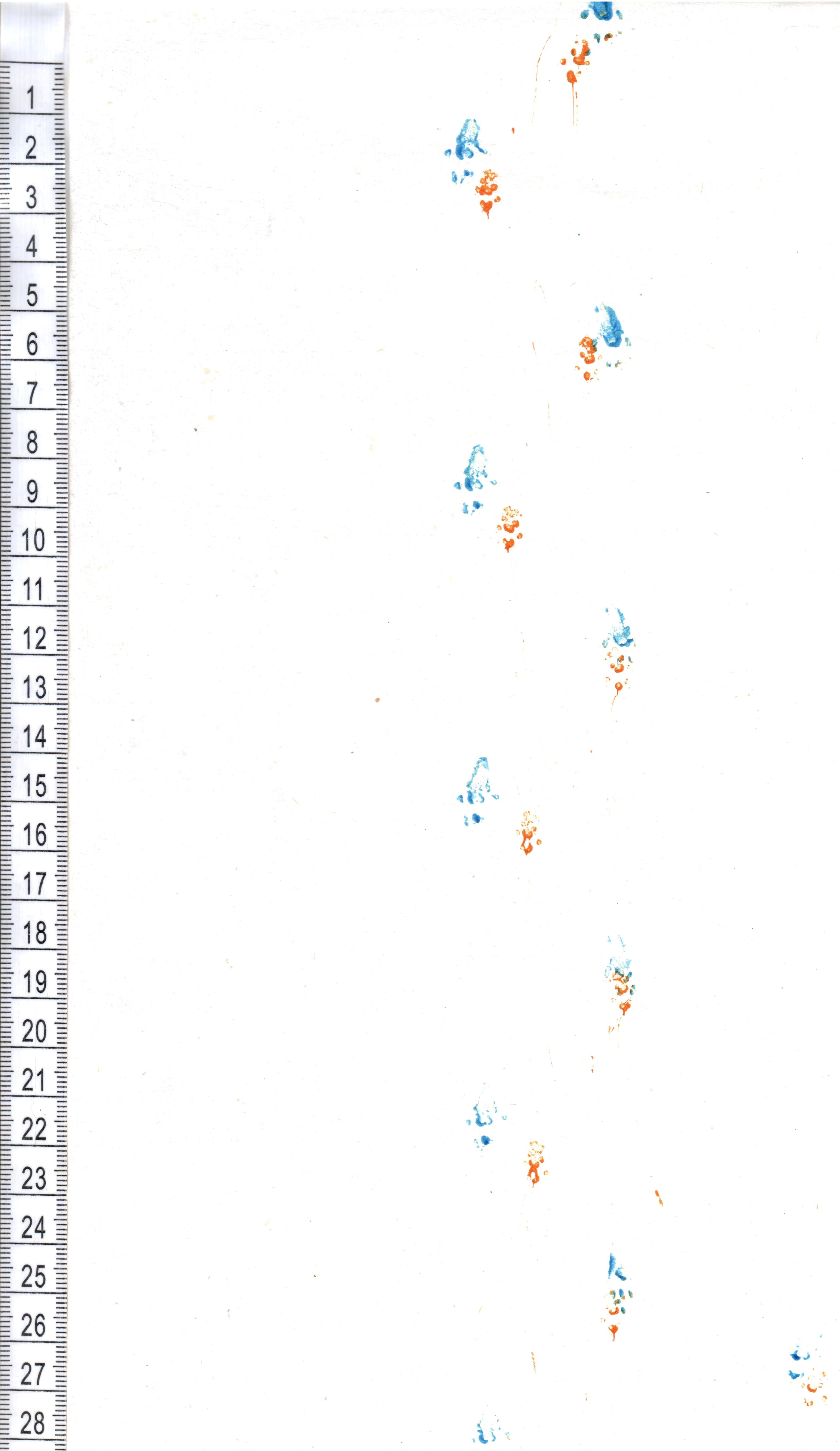

Supplement: Supplementary file 13 — Source data Fig. 6 [file 44318_2025_654_MOESM13_ESM.zip › Figure 6/6D/6D-3-female-WT.jpg]

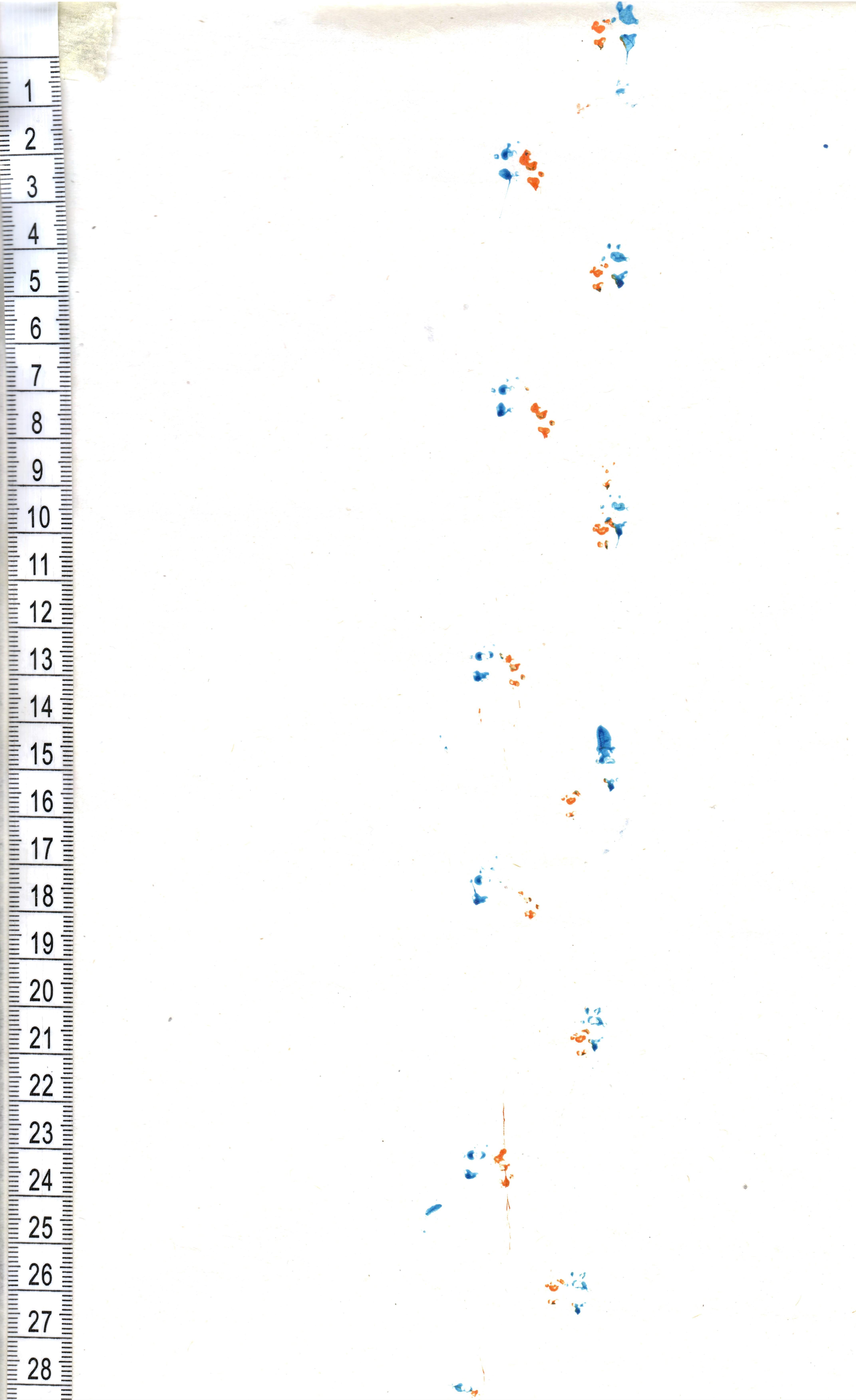

Supplement: Supplementary file 13 — Source data Fig. 6 [file 44318_2025_654_MOESM13_ESM.zip › Figure 6/6D/6D-4-female-KO.jpg]

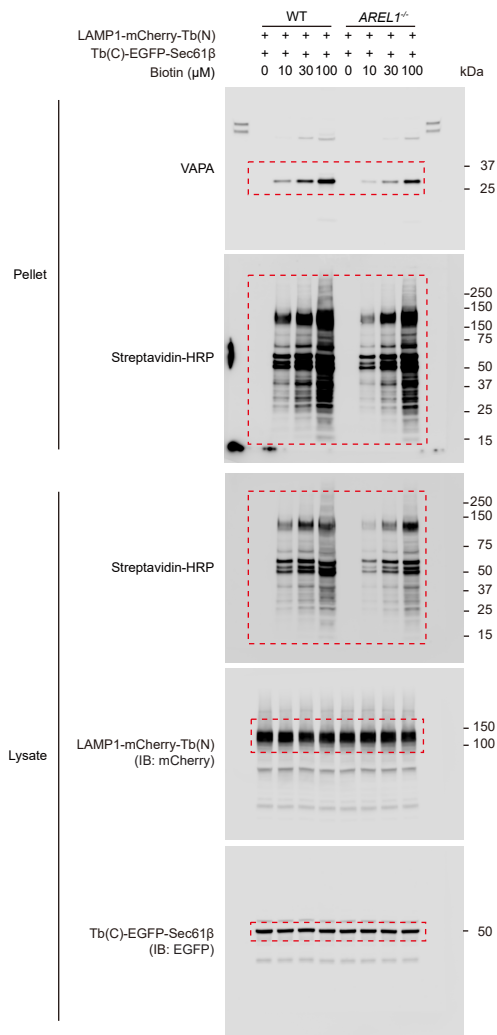

Supplement: Supplementary file 14 — Figure EV1 Source Data [file 44318_2025_654_MOESM14_ESM.zip › EV Figure 1/EV1J/EV1J.pdf]

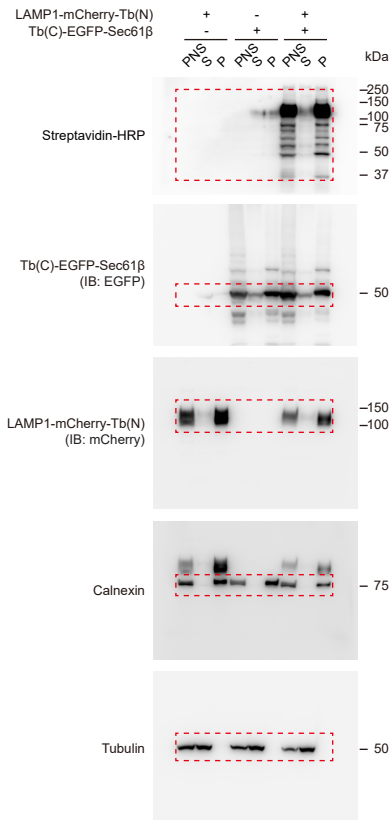

Supplement: Supplementary file 14 — Figure EV1 Source Data [file 44318_2025_654_MOESM14_ESM.zip › EV Figure 1/EV1C/EV1C.pdf]

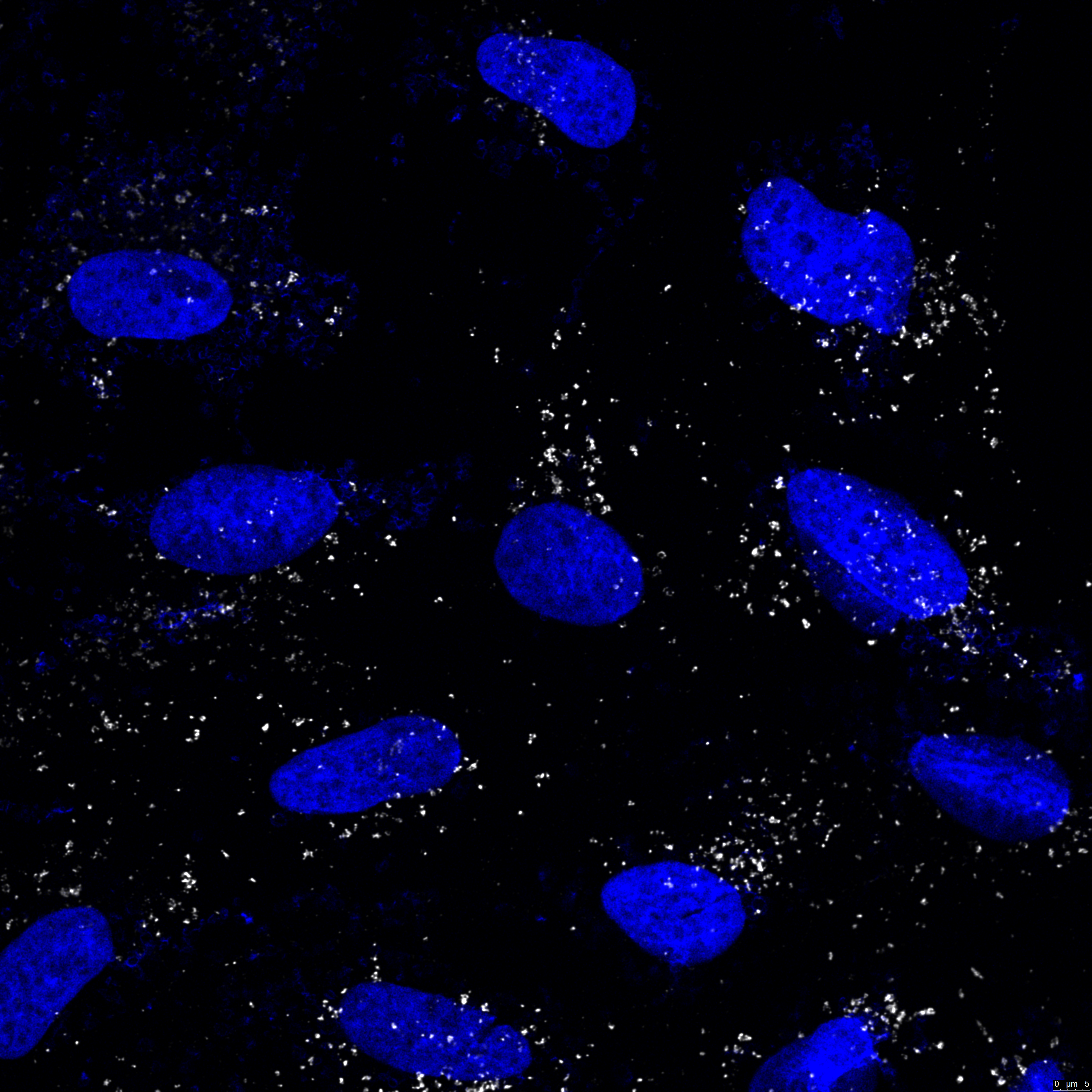

Supplement: Supplementary file 14 — Figure EV1 Source Data [file 44318_2025_654_MOESM14_ESM.zip › EV Figure 1/EV1K/EV1K-2-KO PLA.tif]

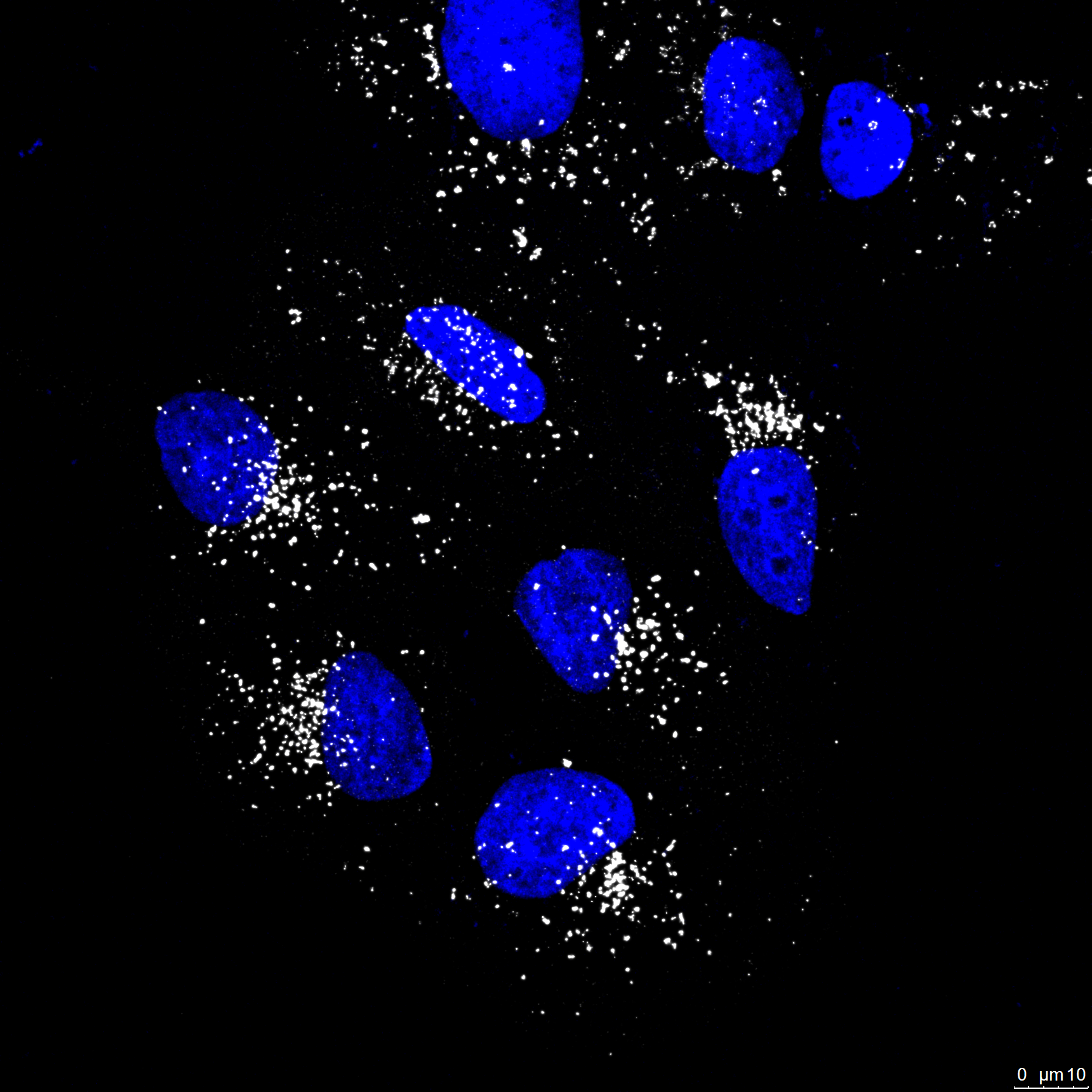

Supplement: Supplementary file 14 — Figure EV1 Source Data [file 44318_2025_654_MOESM14_ESM.zip › EV Figure 1/EV1K/EV1K-1-WT PLA.tif]

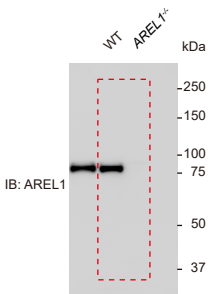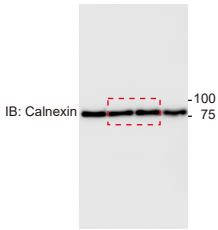

Supplement: Supplementary file 14 — Figure EV1 Source Data [file 44318_2025_654_MOESM14_ESM.zip › EV Figure 1/EV1I/EV1I.pdf]

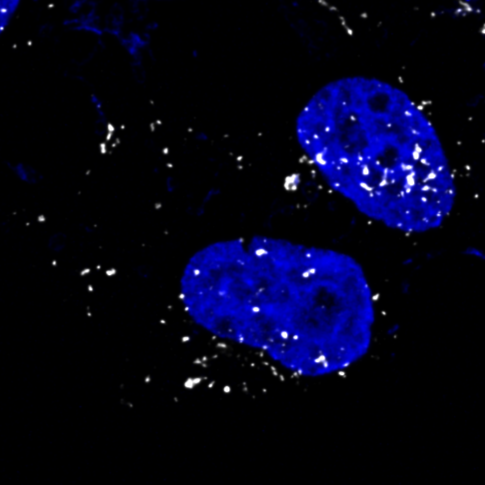

Supplement: Supplementary file 14 — Figure EV1 Source Data [file 44318_2025_654_MOESM14_ESM.zip › EV Figure 1/EV1N/EV1N-2-293 KO PLA.tif]

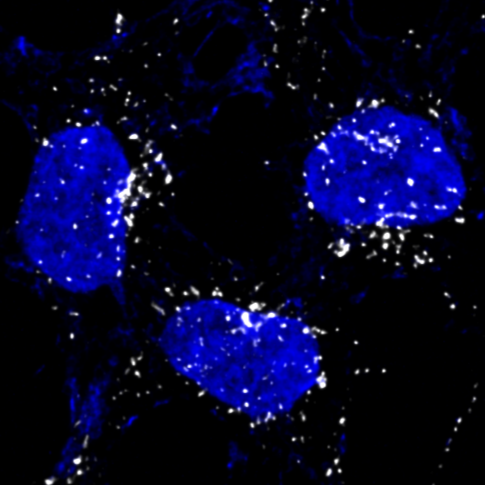

Supplement: Supplementary file 14 — Figure EV1 Source Data [file 44318_2025_654_MOESM14_ESM.zip › EV Figure 1/EV1N/EV1N-1-293 WT PLA.tif]

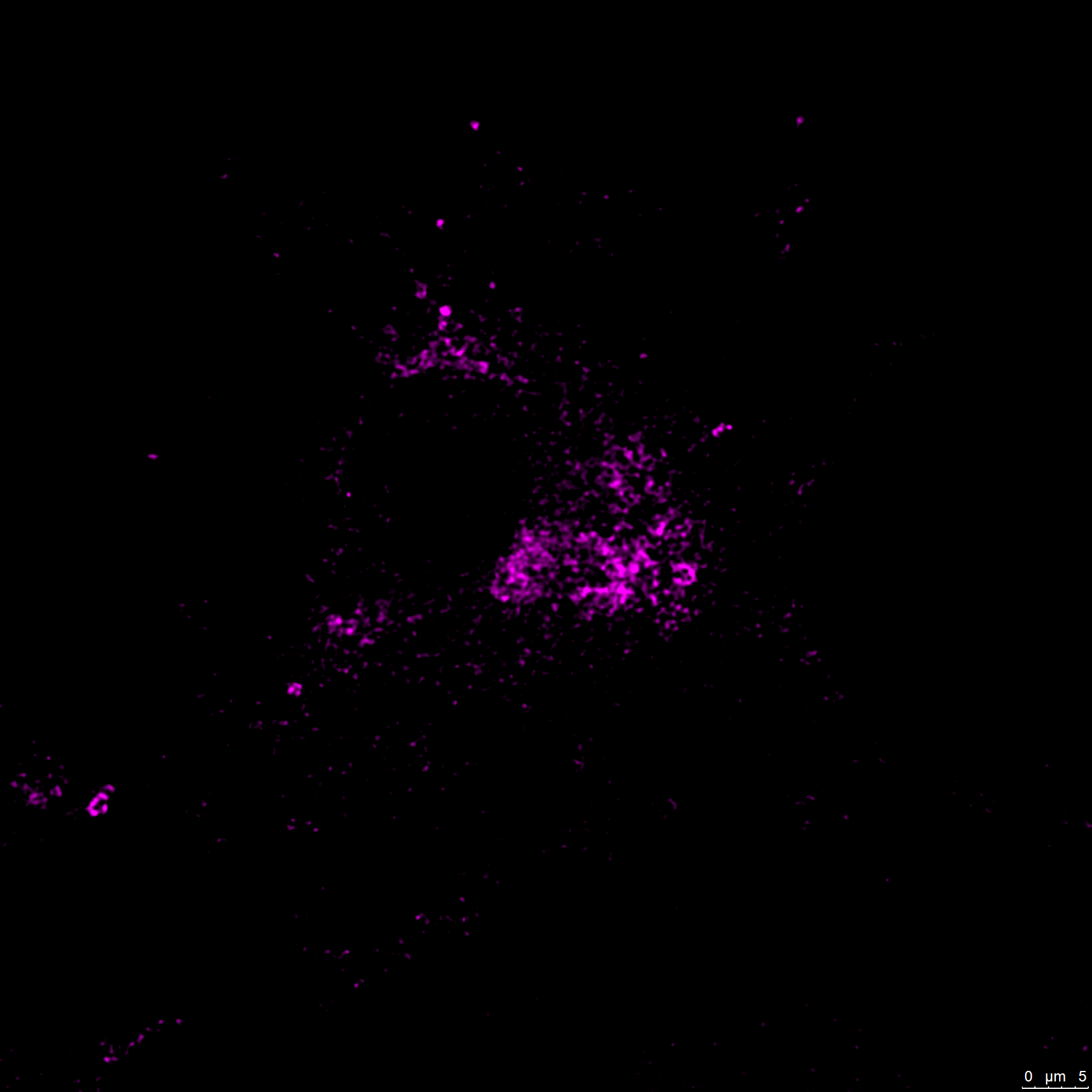

Supplement: Supplementary file 14 — Figure EV1 Source Data [file 44318_2025_654_MOESM14_ESM.zip › EV Figure 1/EV1A/EV1A-Streptavidin-647.tif]

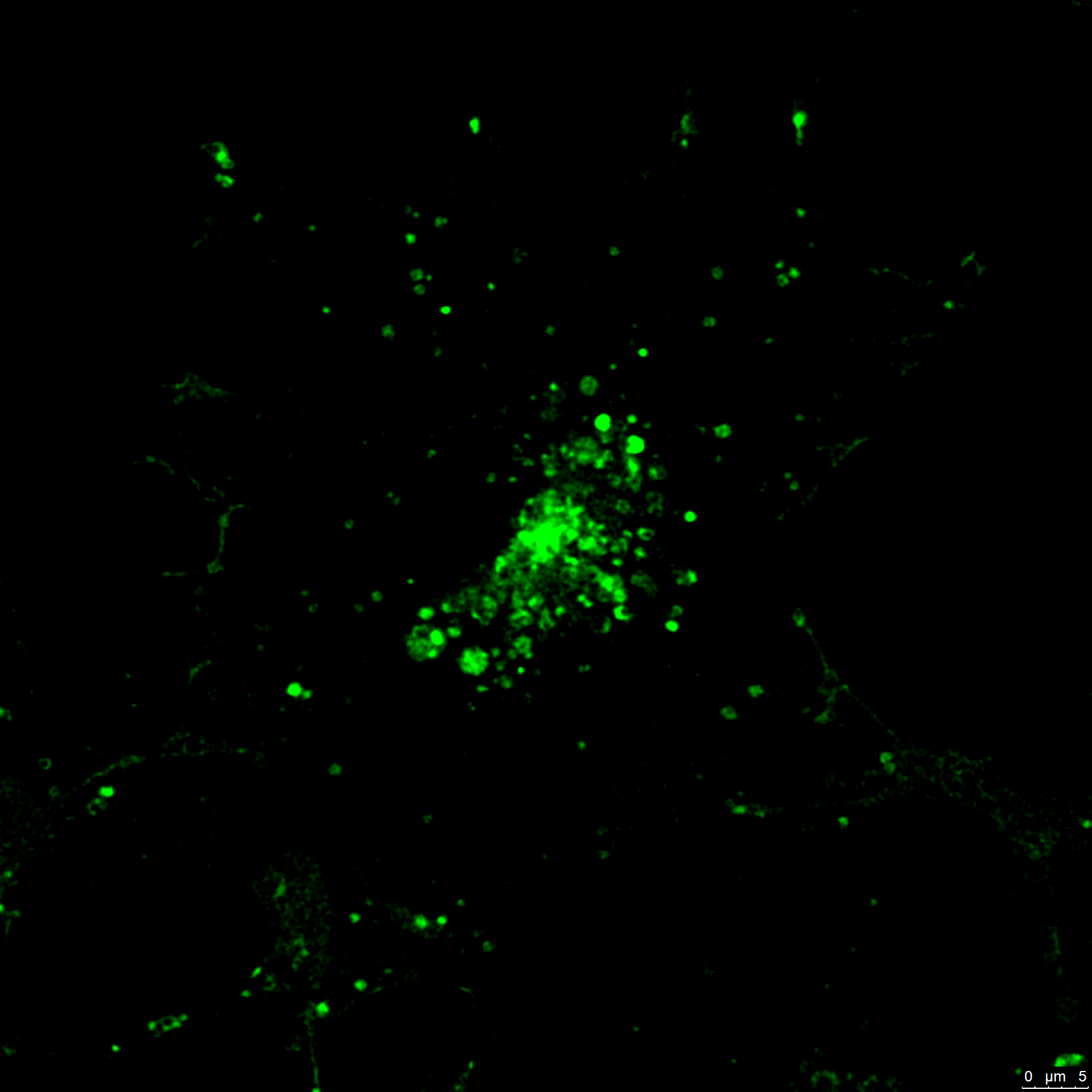

Supplement: Supplementary file 14 — Figure EV1 Source Data [file 44318_2025_654_MOESM14_ESM.zip › EV Figure 1/EV1A/EV1A-LAMP1-mCherry-TB(N).tif]

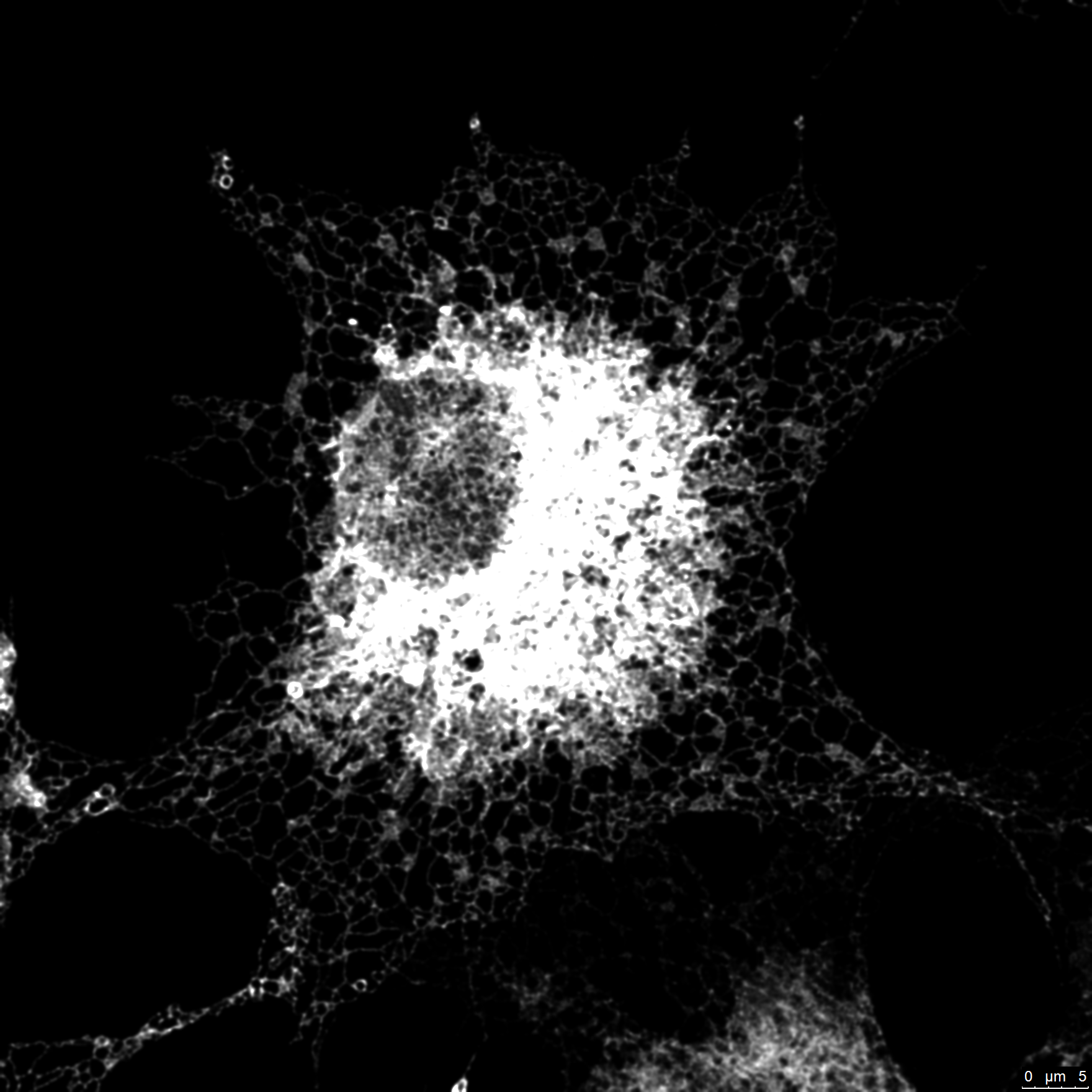

Supplement: Supplementary file 14 — Figure EV1 Source Data [file 44318_2025_654_MOESM14_ESM.zip › EV Figure 1/EV1A/EV1A-Tb(C)-EGFP-Sec61╬▓.tif]

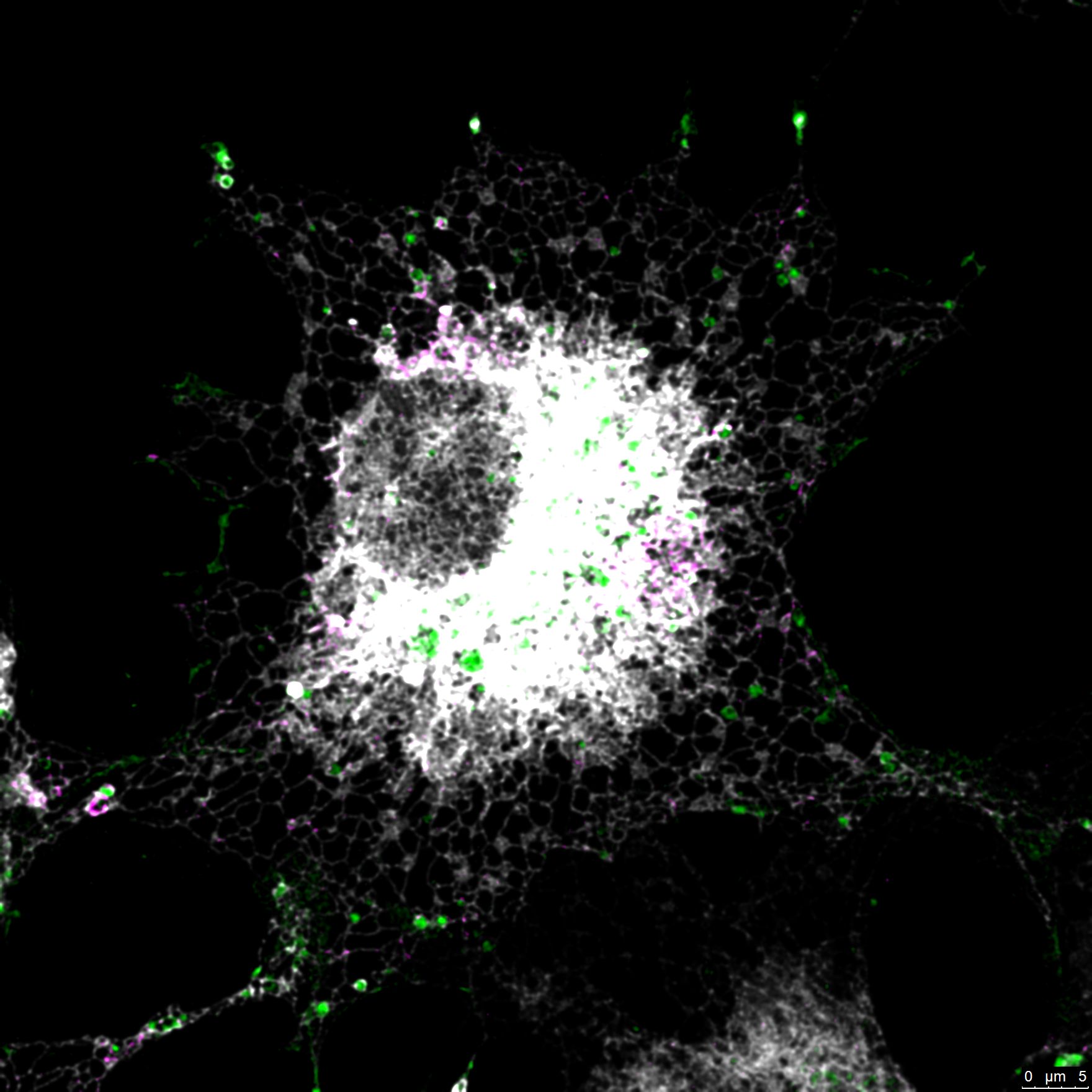

Supplement: Supplementary file 14 — Figure EV1 Source Data [file 44318_2025_654_MOESM14_ESM.zip › EV Figure 1/EV1A/EV1A-merge.tif]

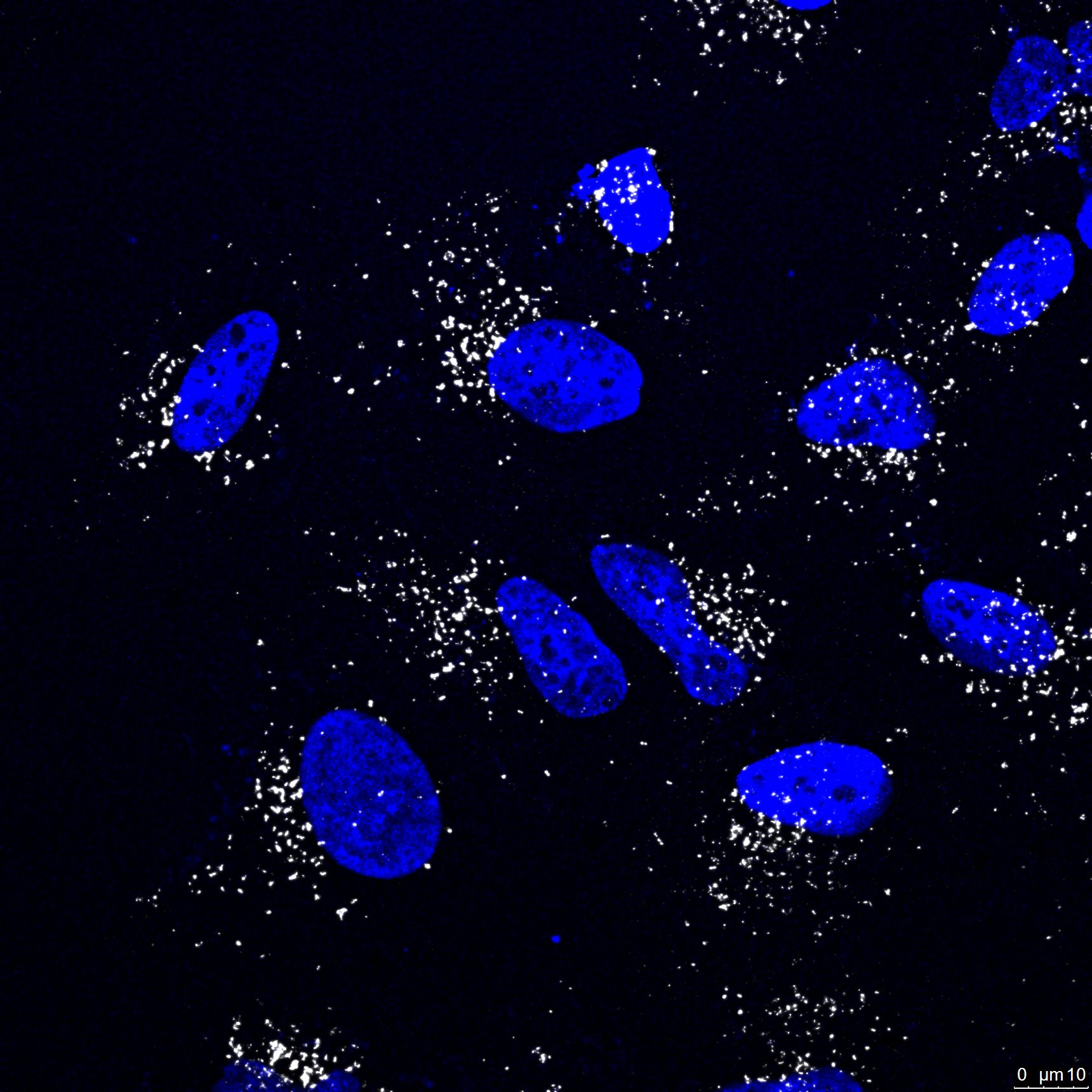

Supplement: Supplementary file 14 — Figure EV1 Source Data [file 44318_2025_654_MOESM14_ESM.zip › EV Figure 1/EV1F/EV1F-8-shESYT1.tif]

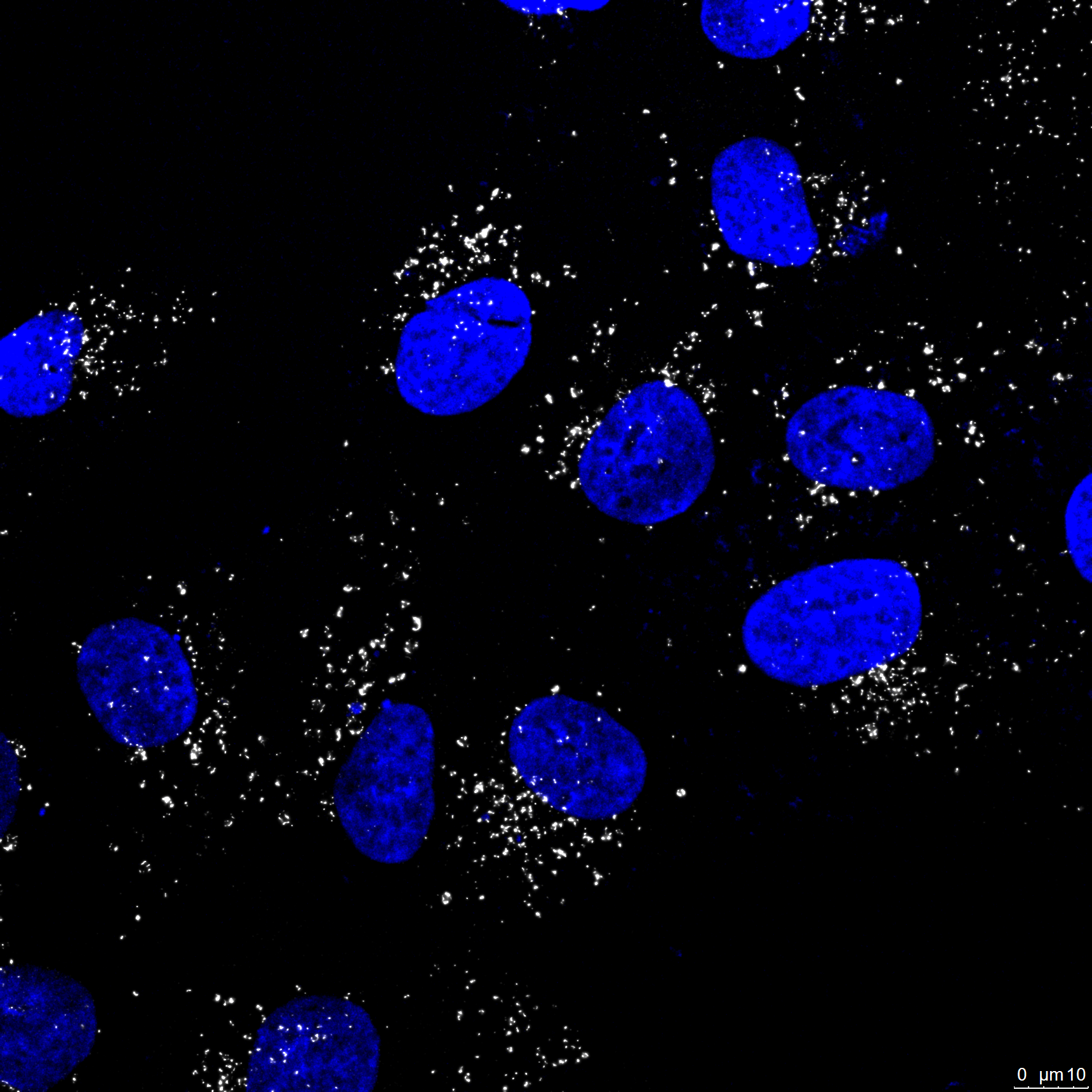

Supplement: Supplementary file 14 — Figure EV1 Source Data [file 44318_2025_654_MOESM14_ESM.zip › EV Figure 1/EV1F/EV1F-19-shTMEM9.tif]

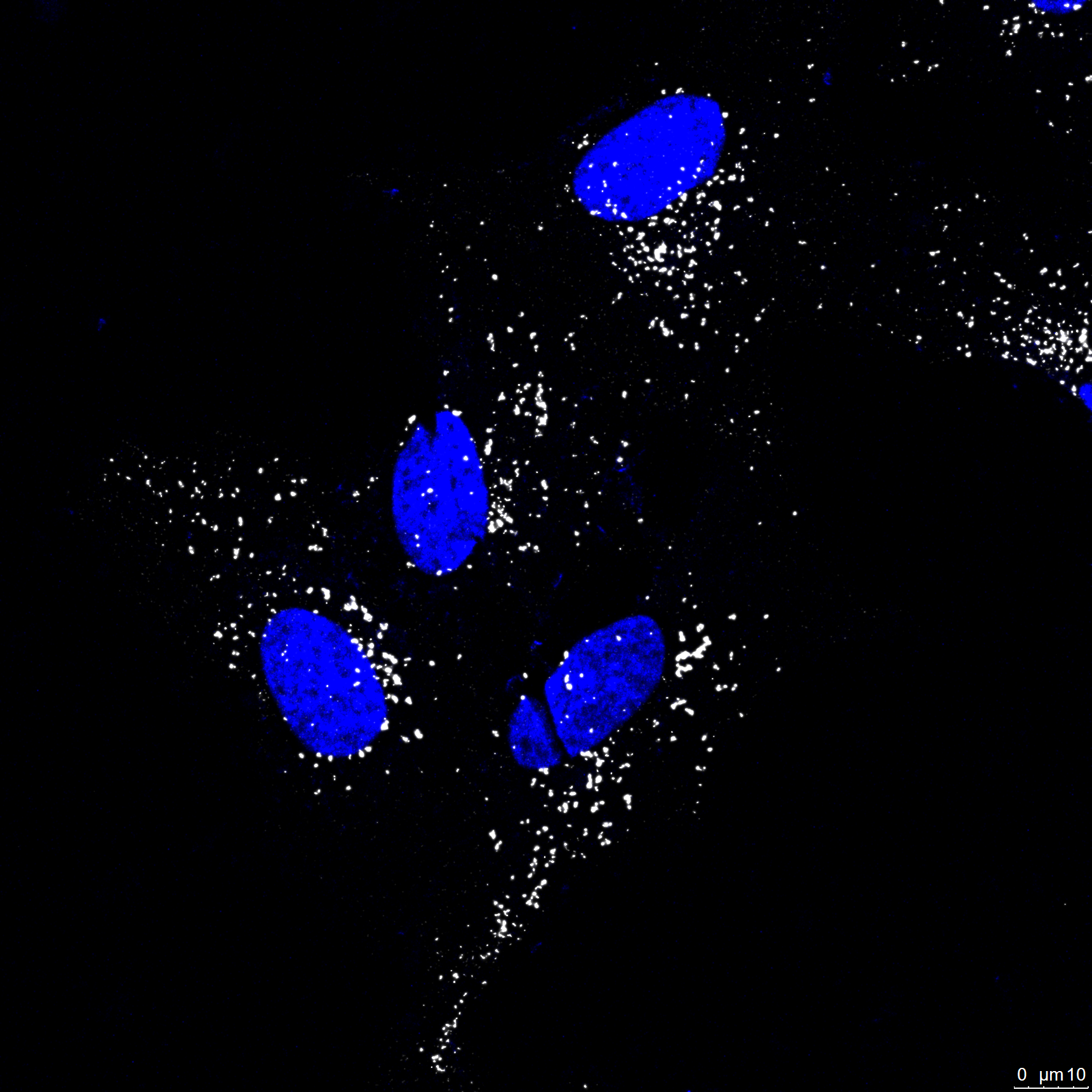

Supplement: Supplementary file 14 — Figure EV1 Source Data [file 44318_2025_654_MOESM14_ESM.zip › EV Figure 1/EV1F/EV1F-15-shTMX1.tif]

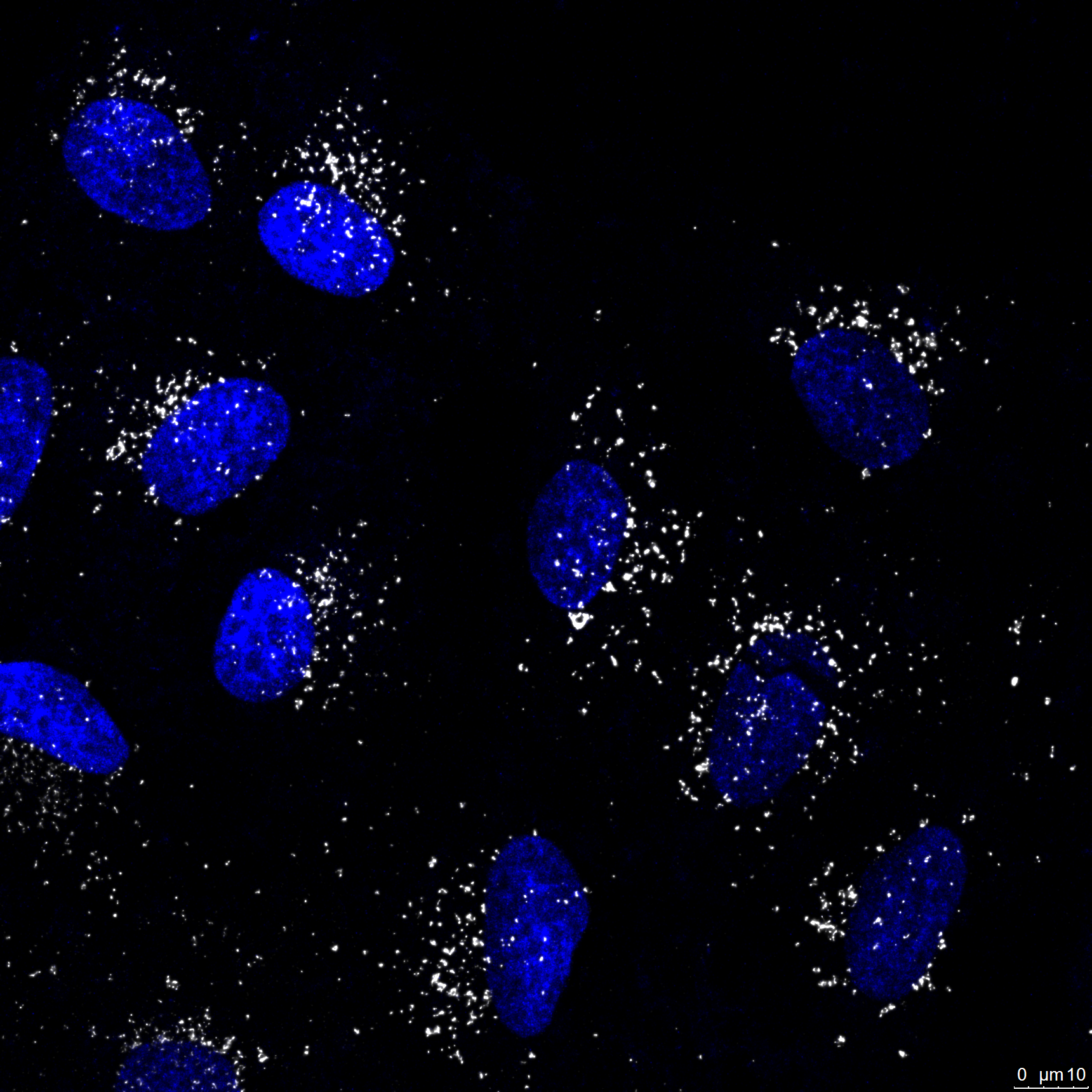

Supplement: Supplementary file 14 — Figure EV1 Source Data [file 44318_2025_654_MOESM14_ESM.zip › EV Figure 1/EV1F/EV1F-17-shZFYVE27.tif]

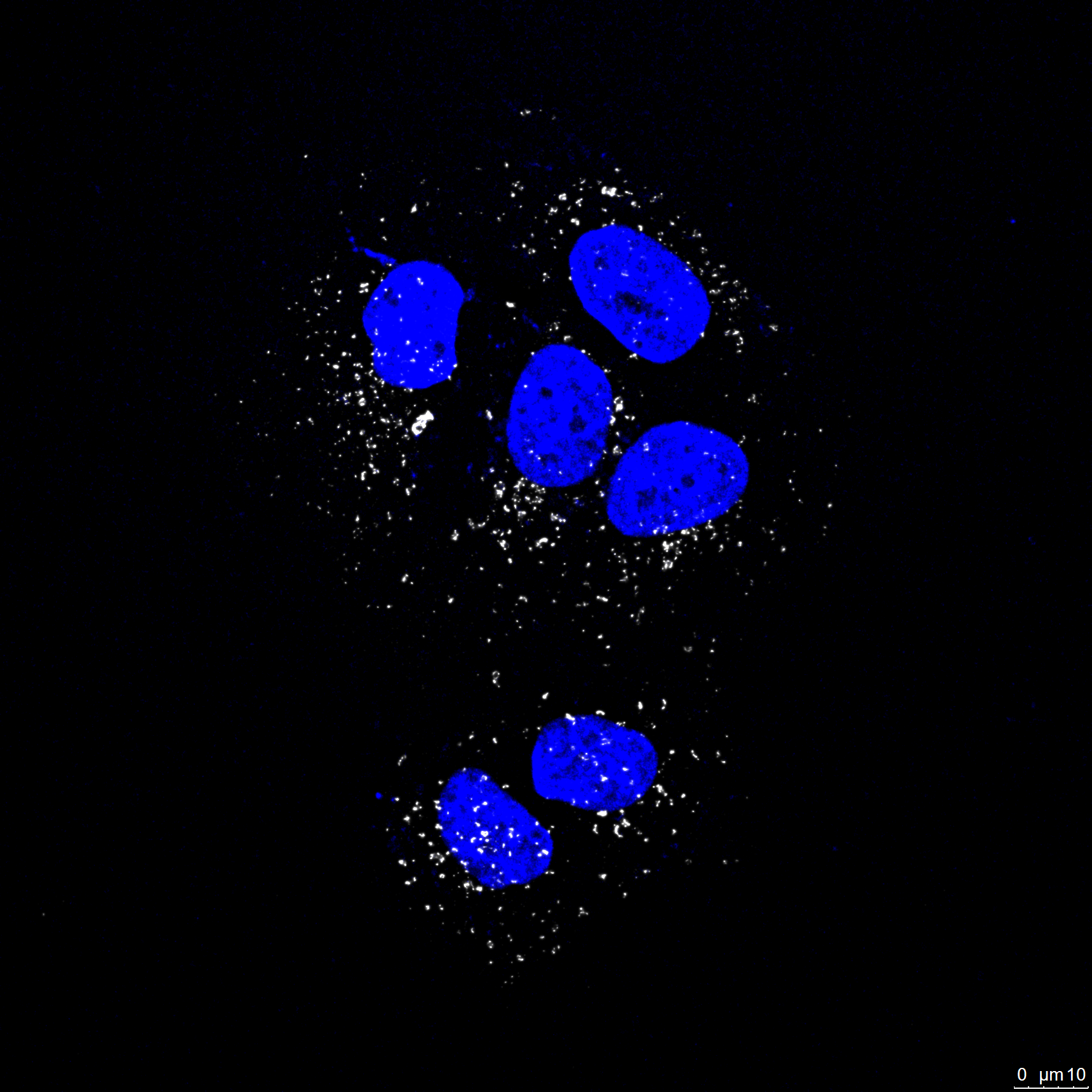

Supplement: Supplementary file 14 — Figure EV1 Source Data [file 44318_2025_654_MOESM14_ESM.zip › EV Figure 1/EV1F/EV1F-16-shMOSPD2.tif]

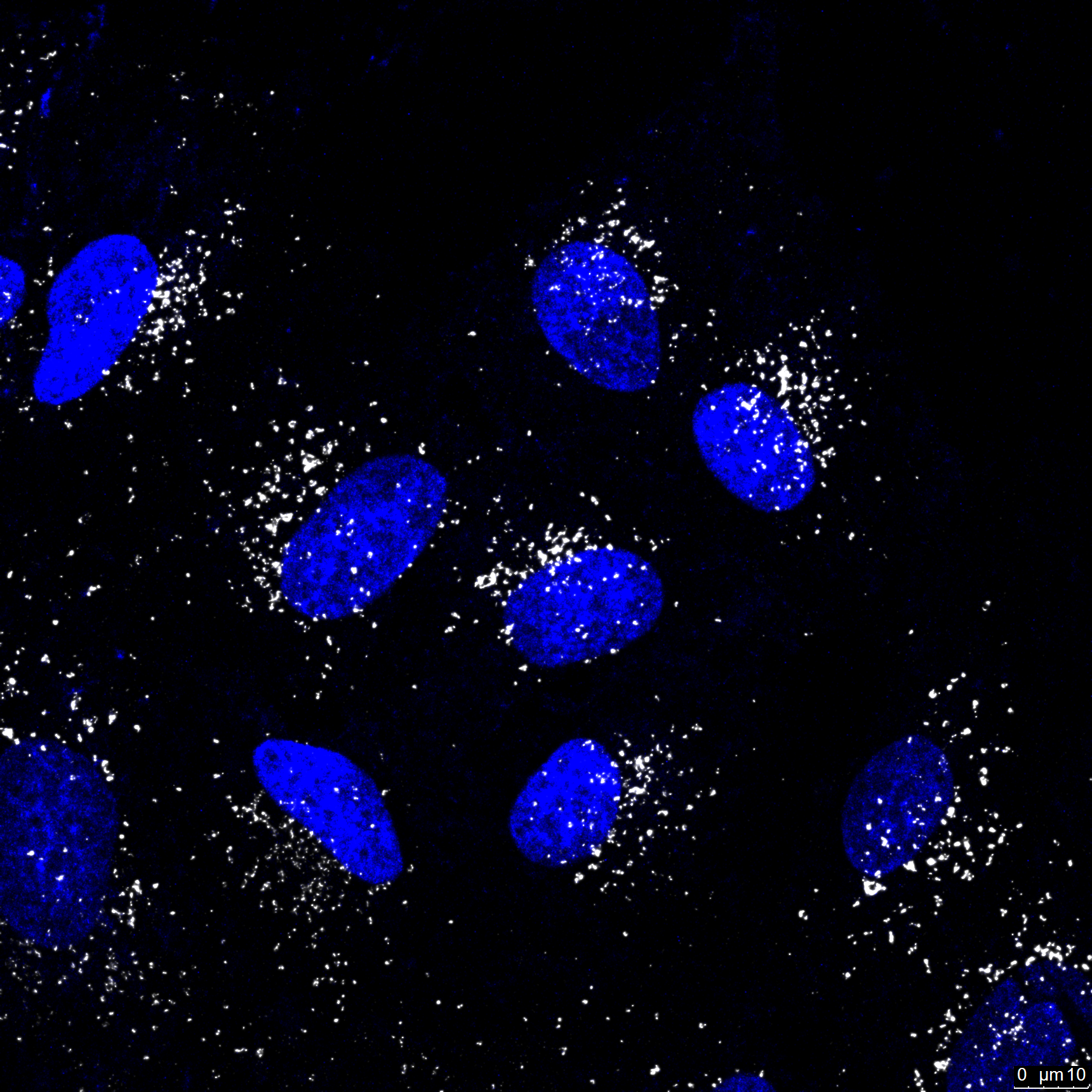

Supplement: Supplementary file 14 — Figure EV1 Source Data [file 44318_2025_654_MOESM14_ESM.zip › EV Figure 1/EV1F/EV1F-12-shUSE1.tif]

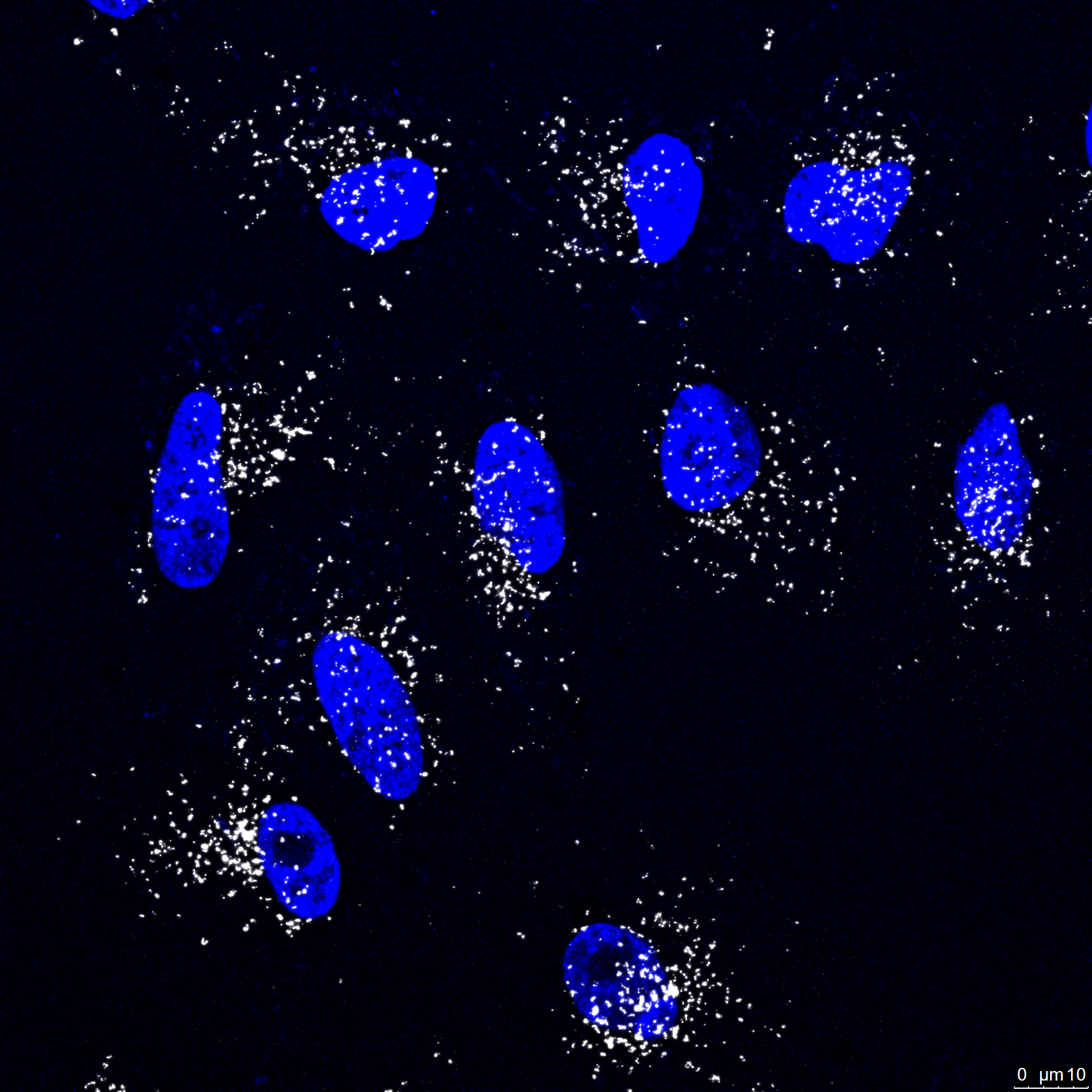

Supplement: Supplementary file 14 — Figure EV1 Source Data [file 44318_2025_654_MOESM14_ESM.zip › EV Figure 1/EV1F/EV1F-9-shTMEM214.tif]

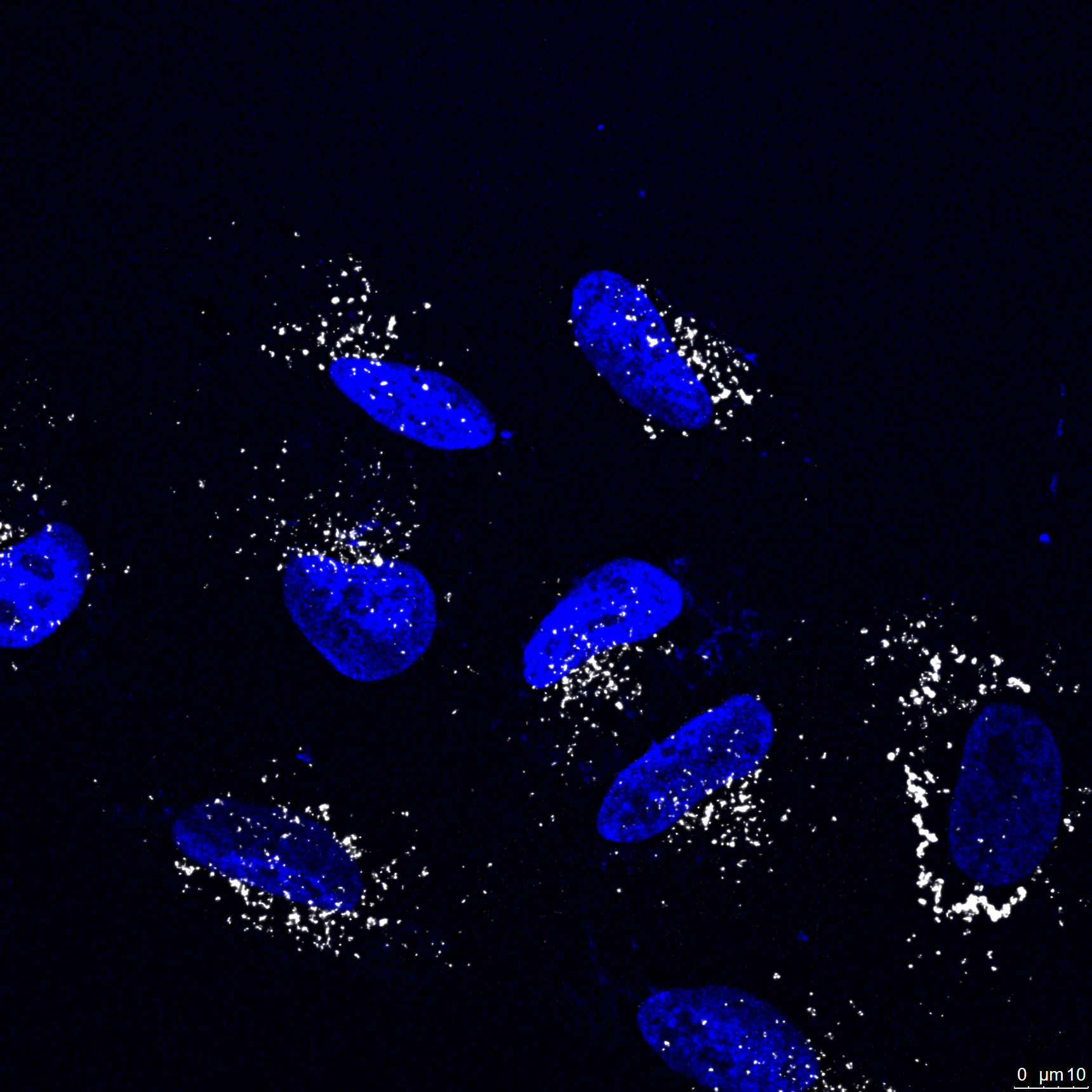

Supplement: Supplementary file 14 — Figure EV1 Source Data [file 44318_2025_654_MOESM14_ESM.zip › EV Figure 1/EV1F/EV1F-2-shATP6AP1.tif]

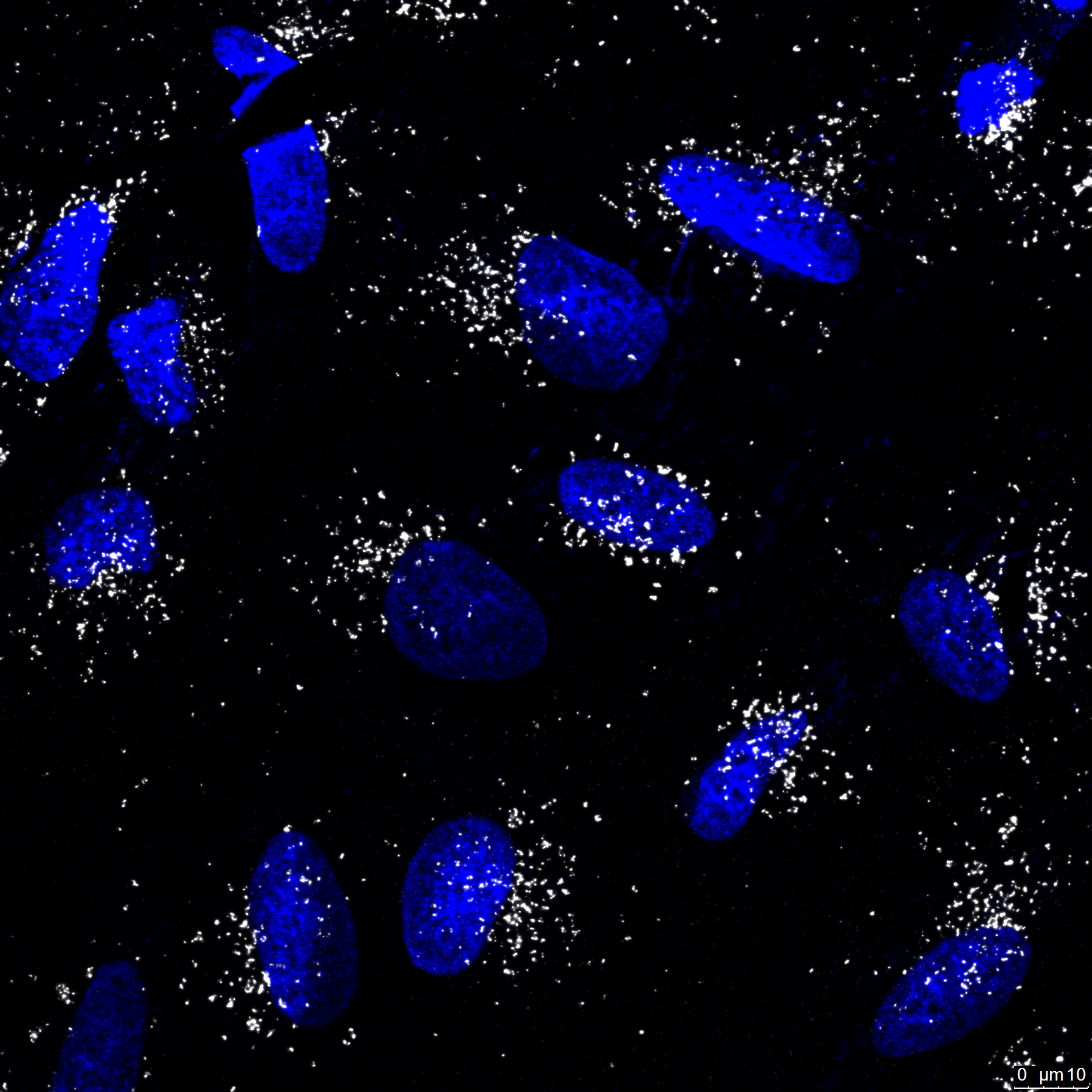

Supplement: Supplementary file 14 — Figure EV1 Source Data [file 44318_2025_654_MOESM14_ESM.zip › EV Figure 1/EV1F/EV1F-1-shNC.tif]

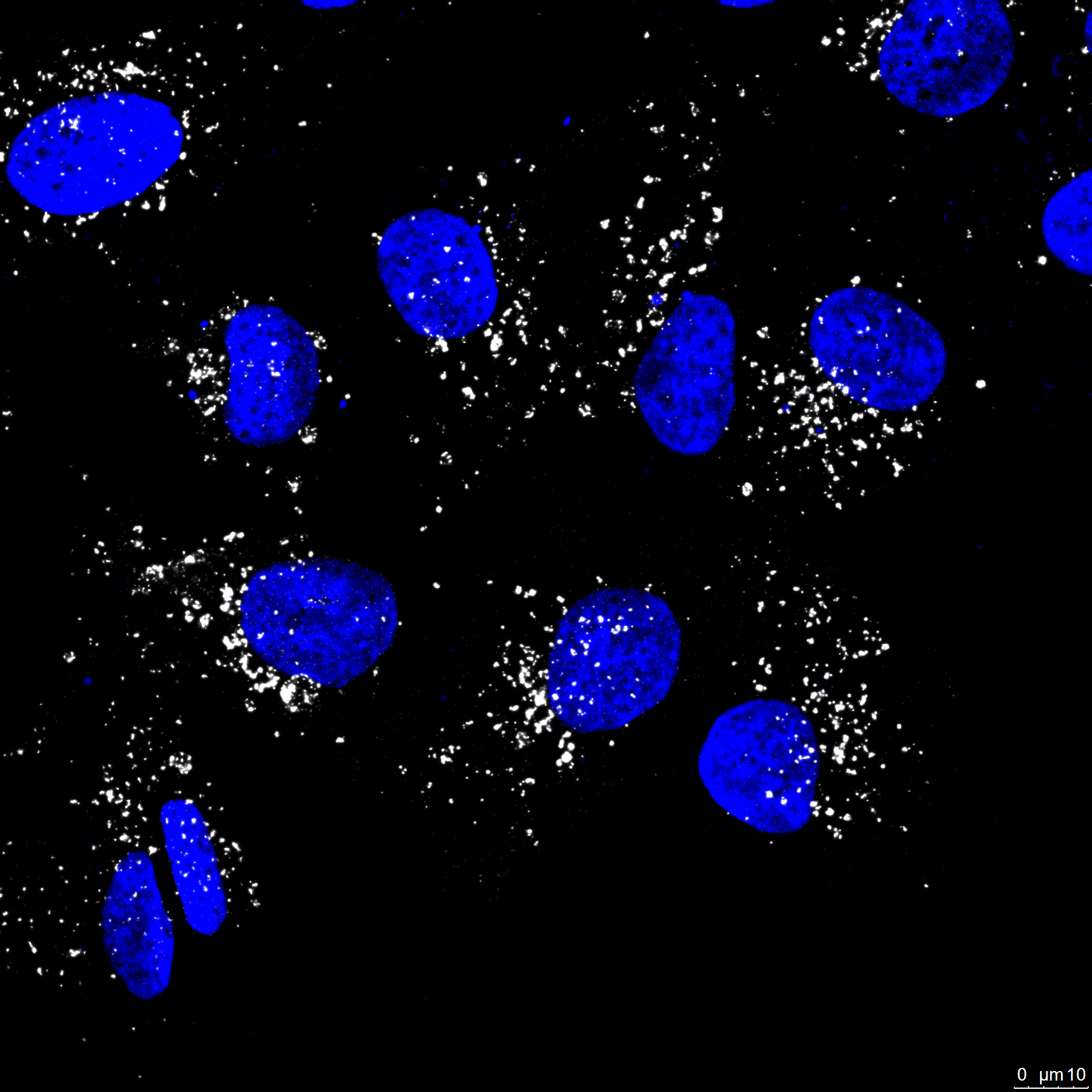

Supplement: Supplementary file 14 — Figure EV1 Source Data [file 44318_2025_654_MOESM14_ESM.zip › EV Figure 1/EV1F/EV1F-10-shSTX18.tif]

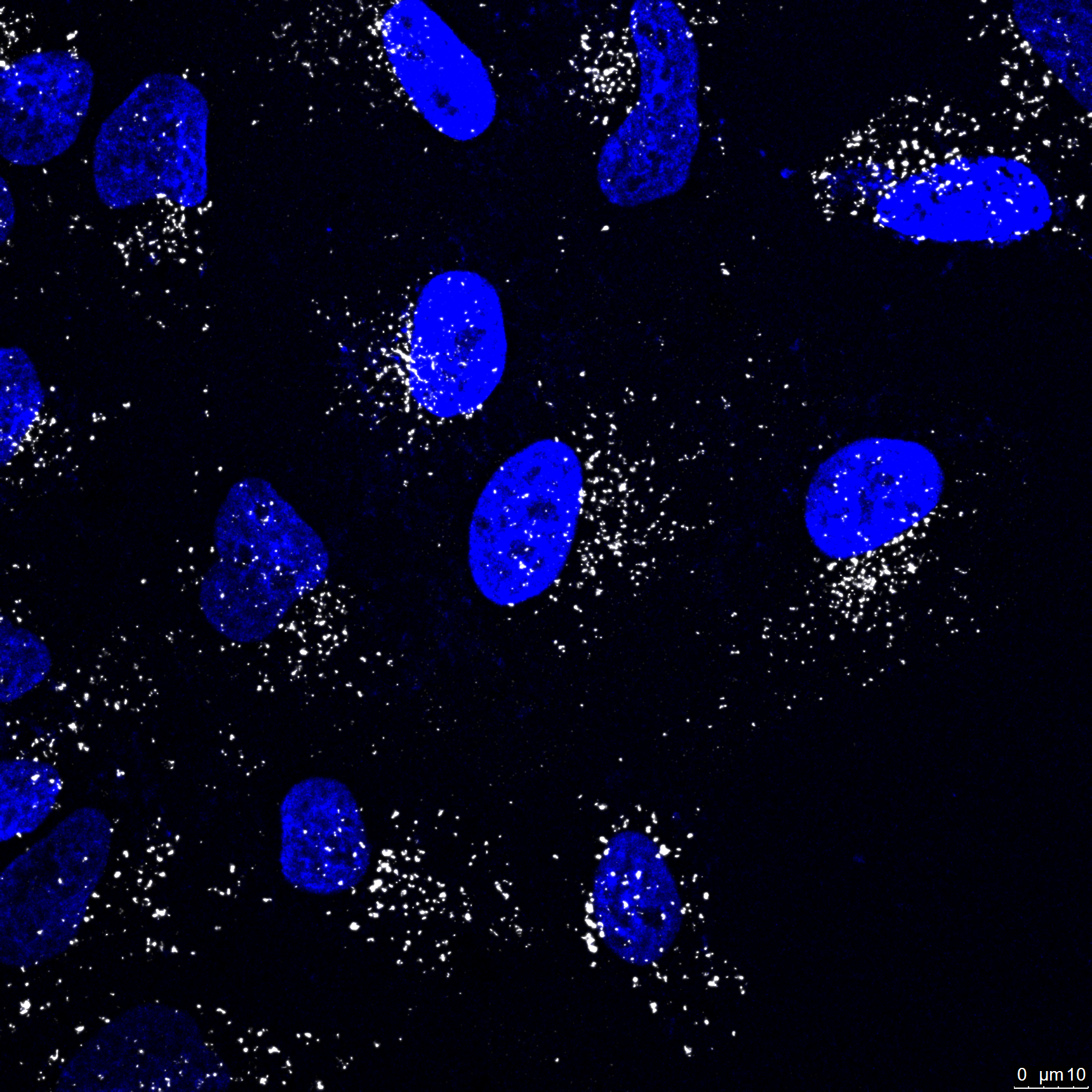

Supplement: Supplementary file 14 — Figure EV1 Source Data [file 44318_2025_654_MOESM14_ESM.zip › EV Figure 1/EV1F/EV1F-7-shANKLE2.tif]
