# Supplementary figures and images for: E3 ligase AREL1 controls perinuclear localization of lysosomes and supports Purkinje cell survival (part 3 of 4)
Source: EMBO J. 2025 Dec 2;45(3):655–91. doi: 10.1038/s44318-025-00654-3 (PMC12864862; doi:10.1038/s44318-025-00654-3)

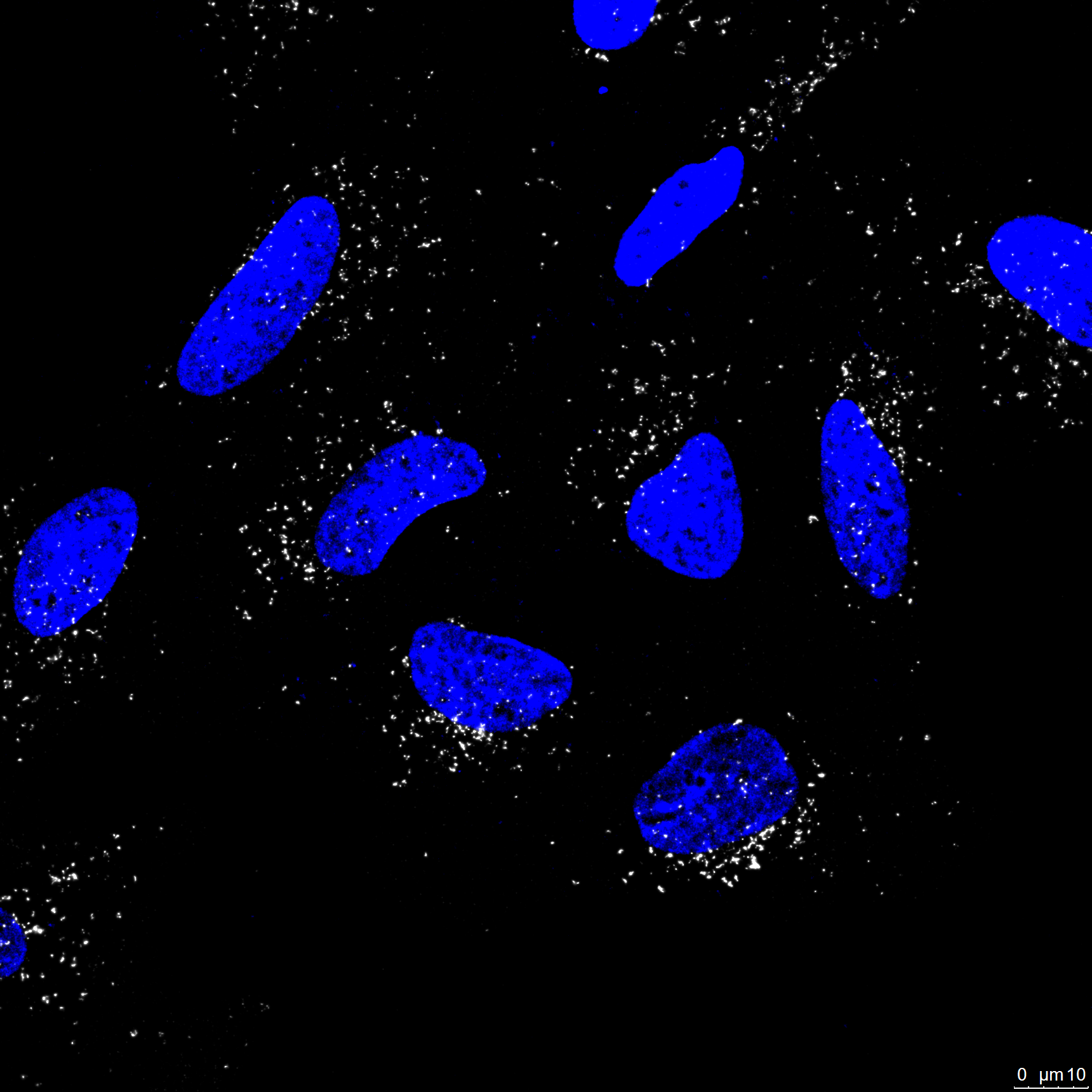

Supplement: Supplementary file 14 — Figure EV1 Source Data [file 44318_2025_654_MOESM14_ESM.zip › EV Figure 1/EV1F/EV1F-13-shUBE2J1.tif]

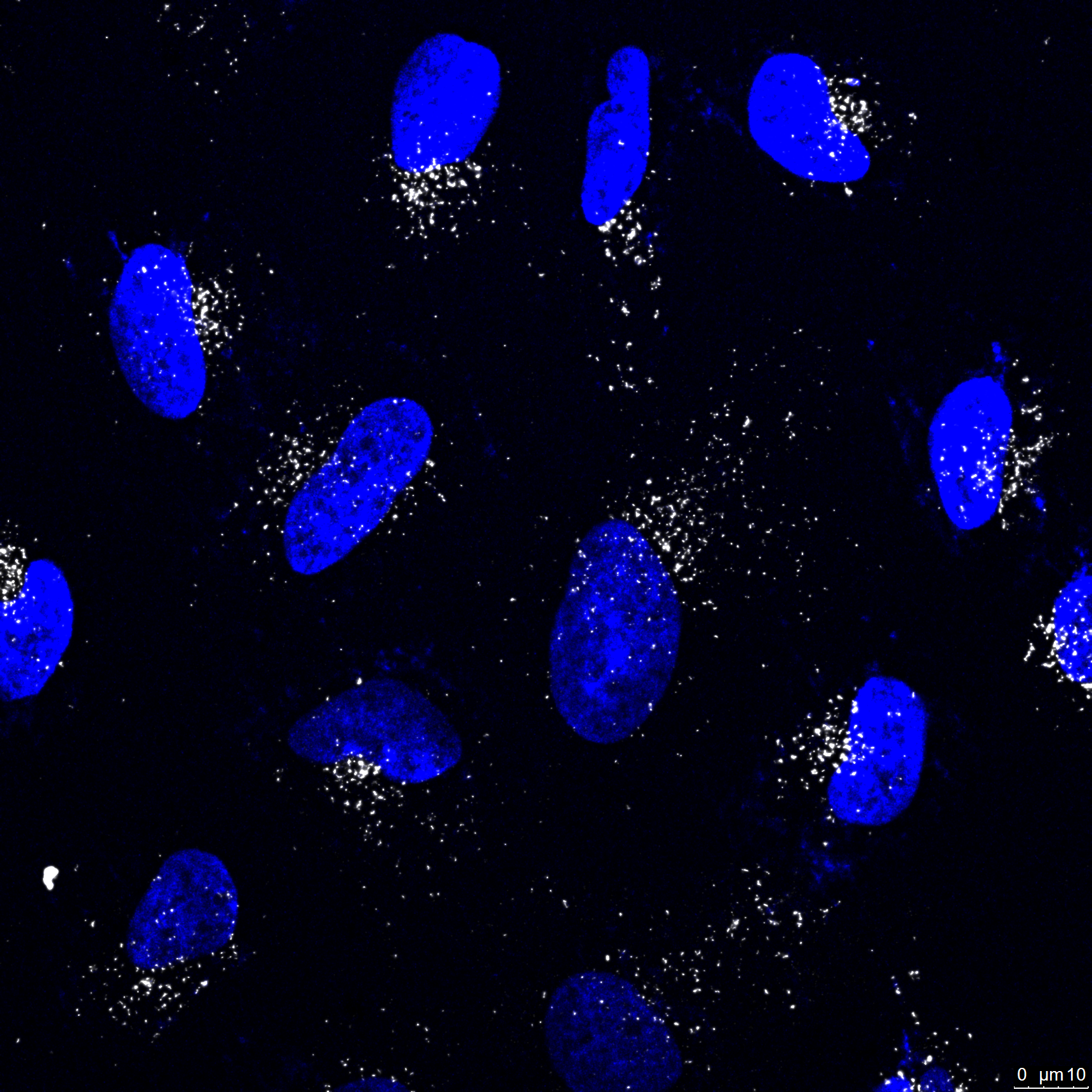

Supplement: Supplementary file 14 — Figure EV1 Source Data [file 44318_2025_654_MOESM14_ESM.zip › EV Figure 1/EV1F/EV1F-14-shAREL1.tif]

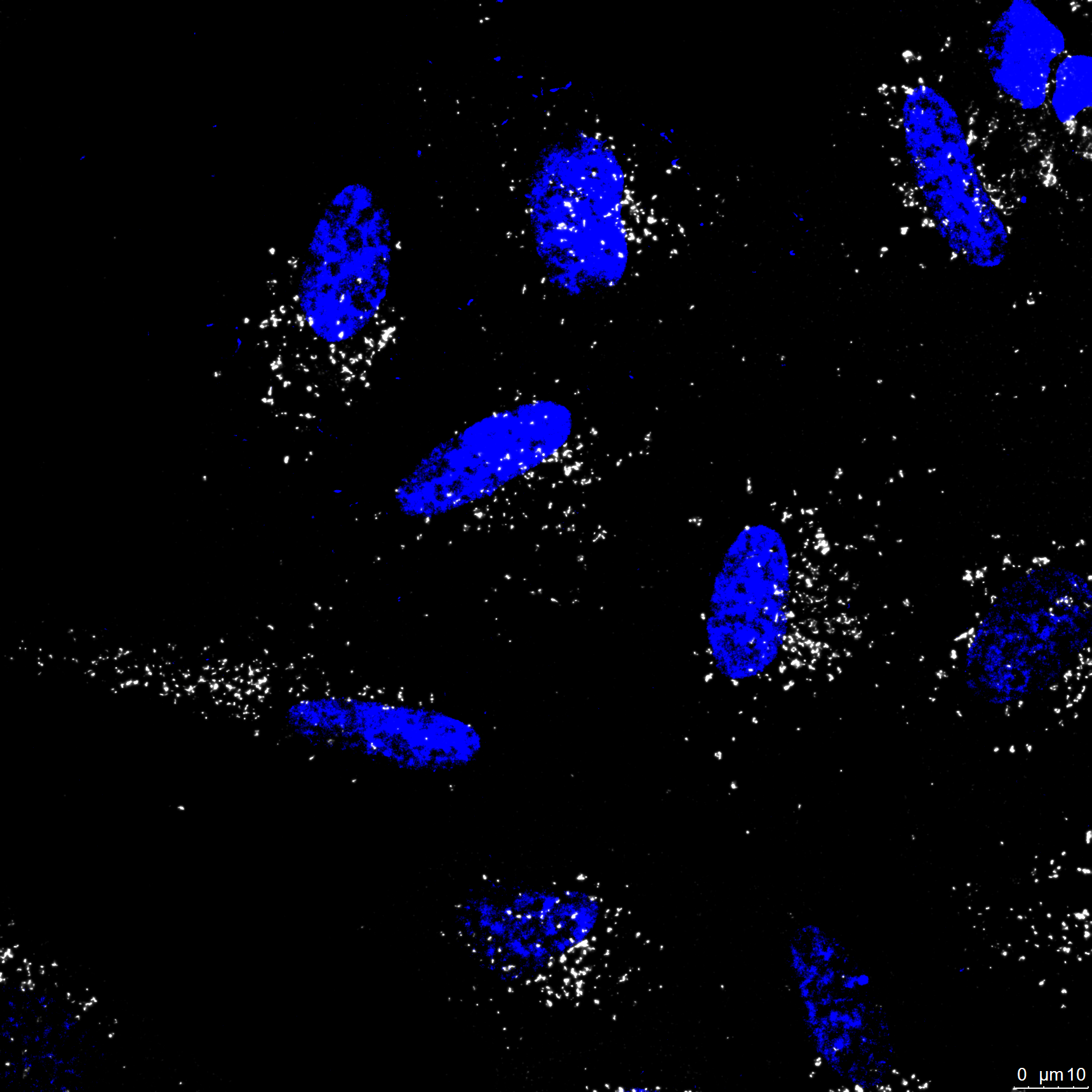

Supplement: Supplementary file 14 — Figure EV1 Source Data [file 44318_2025_654_MOESM14_ESM.zip › EV Figure 1/EV1F/EV1F-4-shATP6AP2.tif]

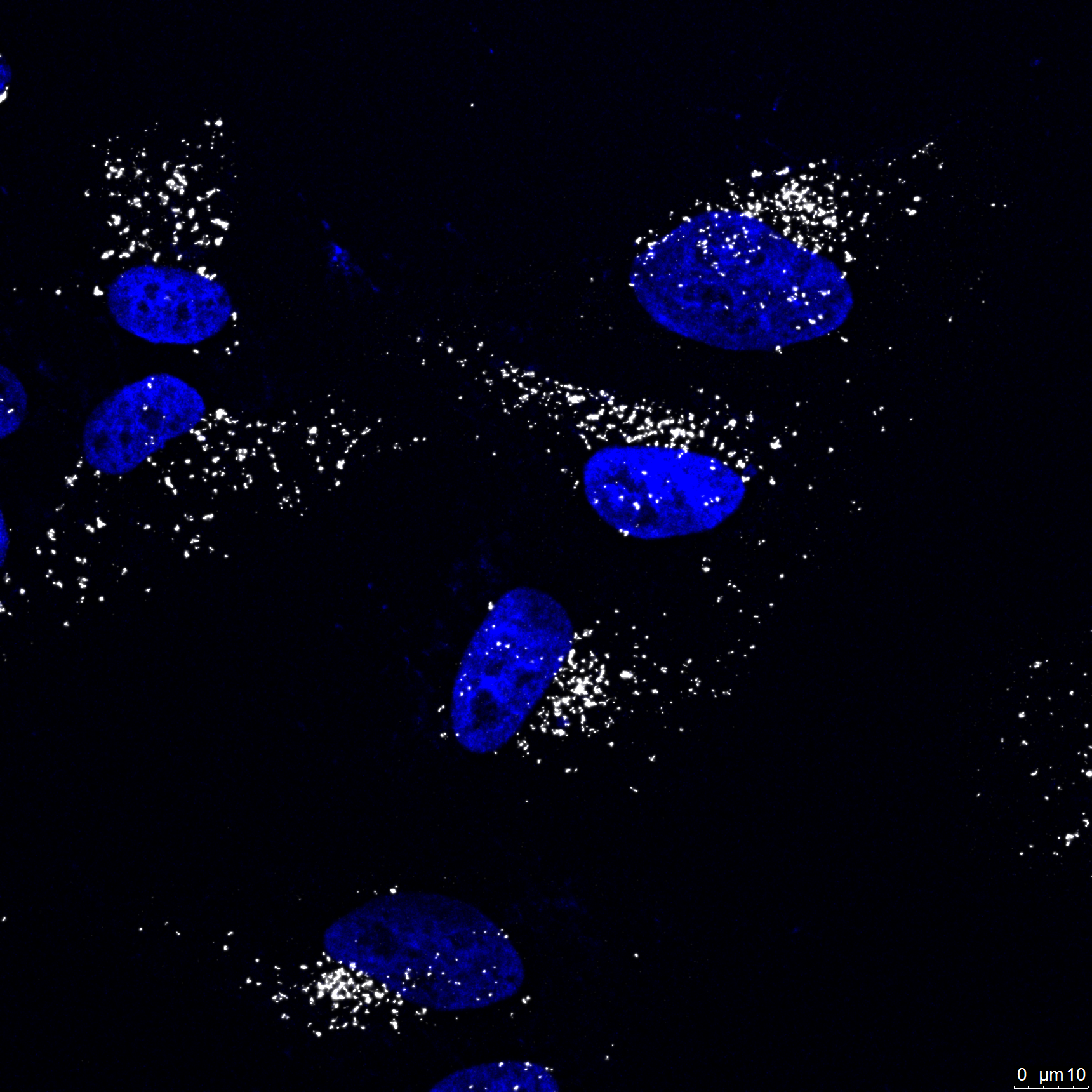

Supplement: Supplementary file 14 — Figure EV1 Source Data [file 44318_2025_654_MOESM14_ESM.zip › EV Figure 1/EV1F/EV1F-18-shESYT2.tif]

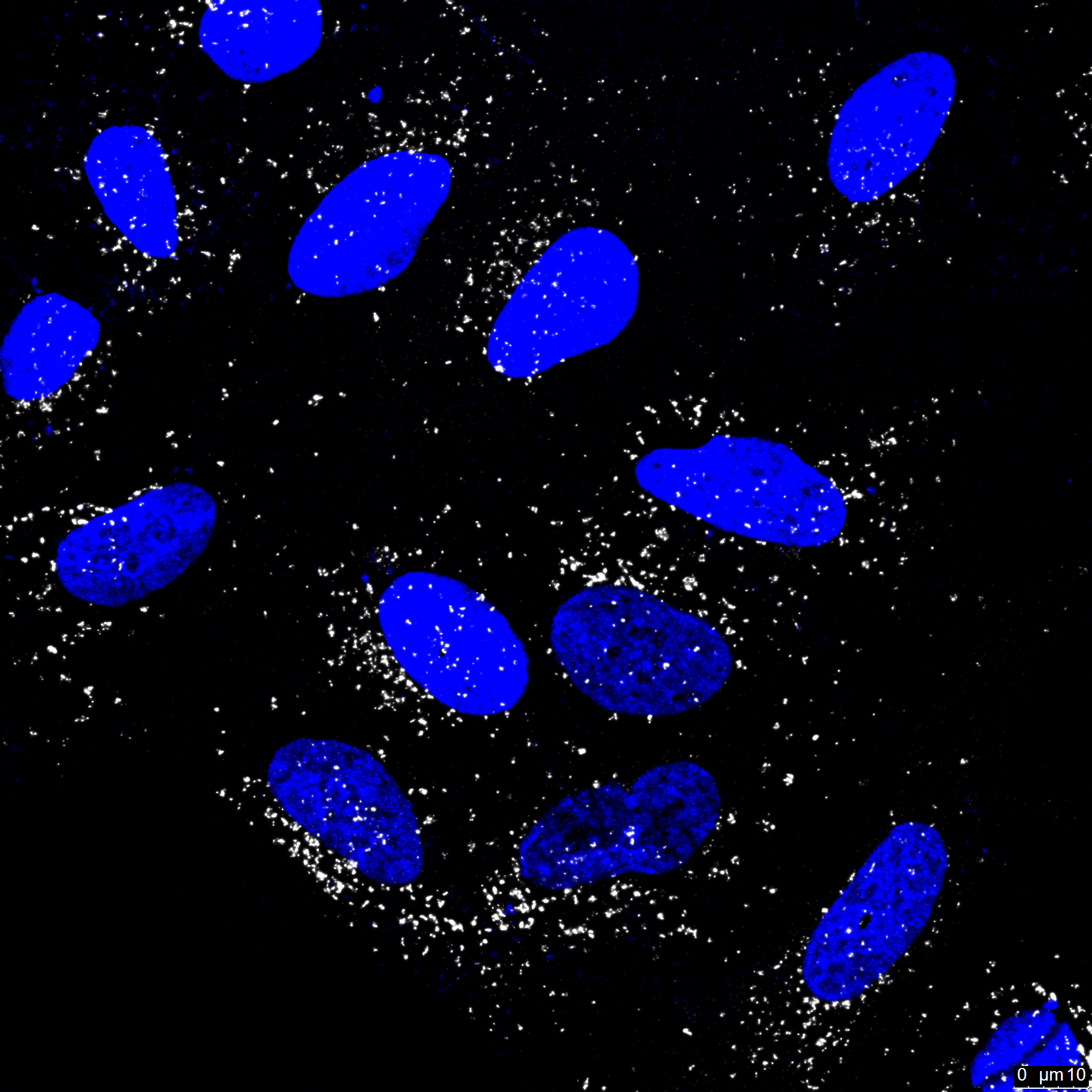

Supplement: Supplementary file 14 — Figure EV1 Source Data [file 44318_2025_654_MOESM14_ESM.zip › EV Figure 1/EV1F/EV1F-3-shMXRA7.tif]

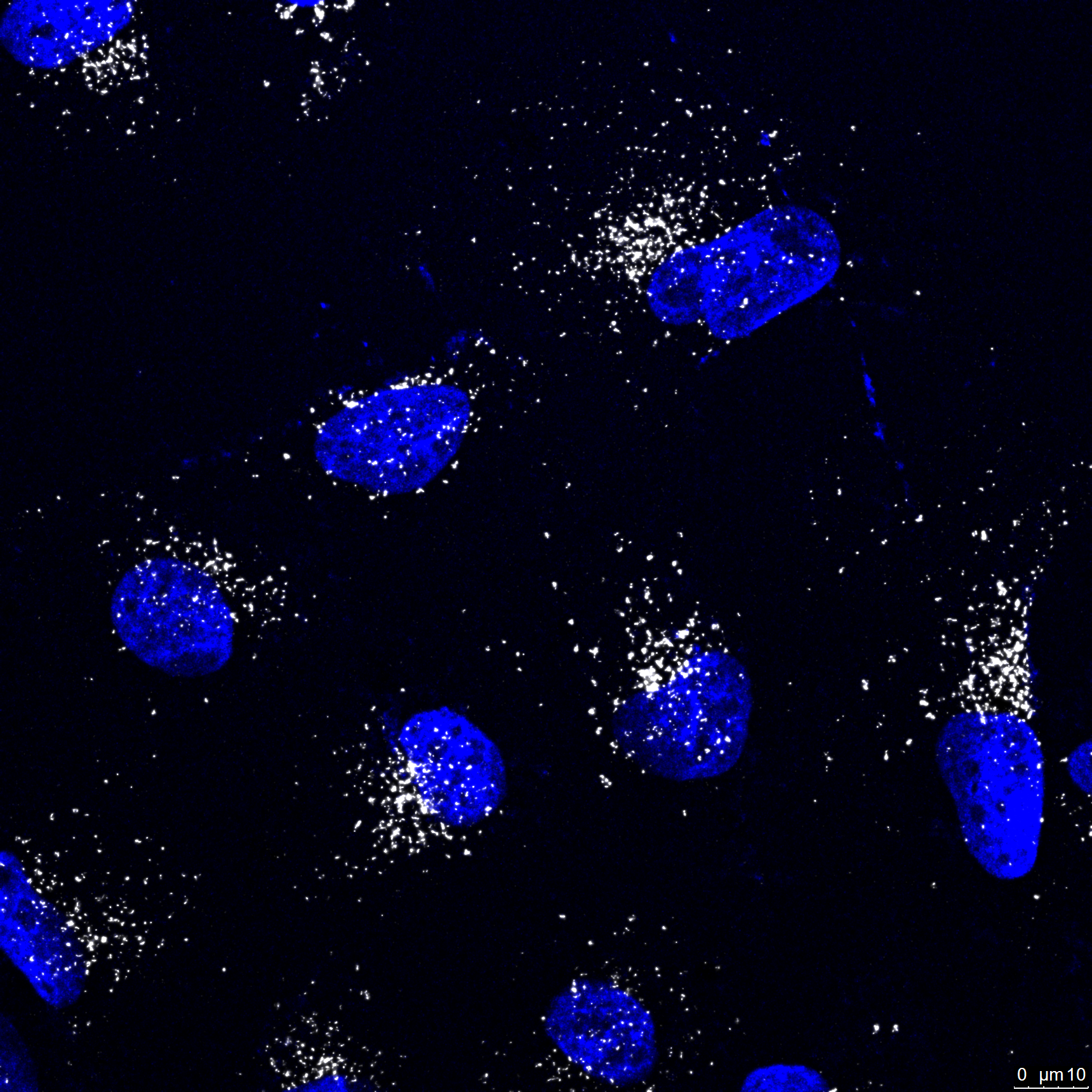

Supplement: Supplementary file 14 — Figure EV1 Source Data [file 44318_2025_654_MOESM14_ESM.zip › EV Figure 1/EV1F/EV1F-11-shTMEM106B.tif]

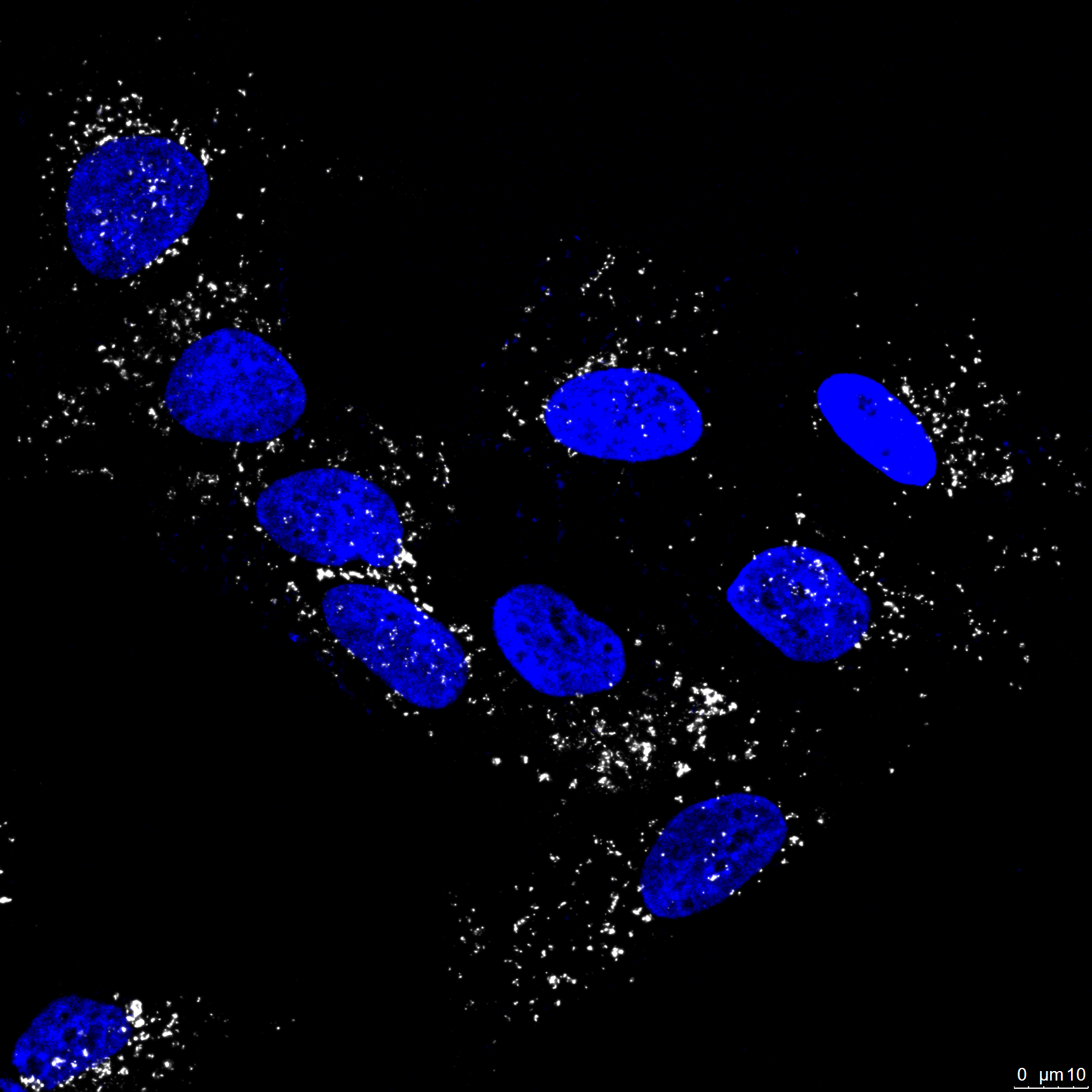

Supplement: Supplementary file 14 — Figure EV1 Source Data [file 44318_2025_654_MOESM14_ESM.zip › EV Figure 1/EV1F/EV1F-20-shRNF26.tif]

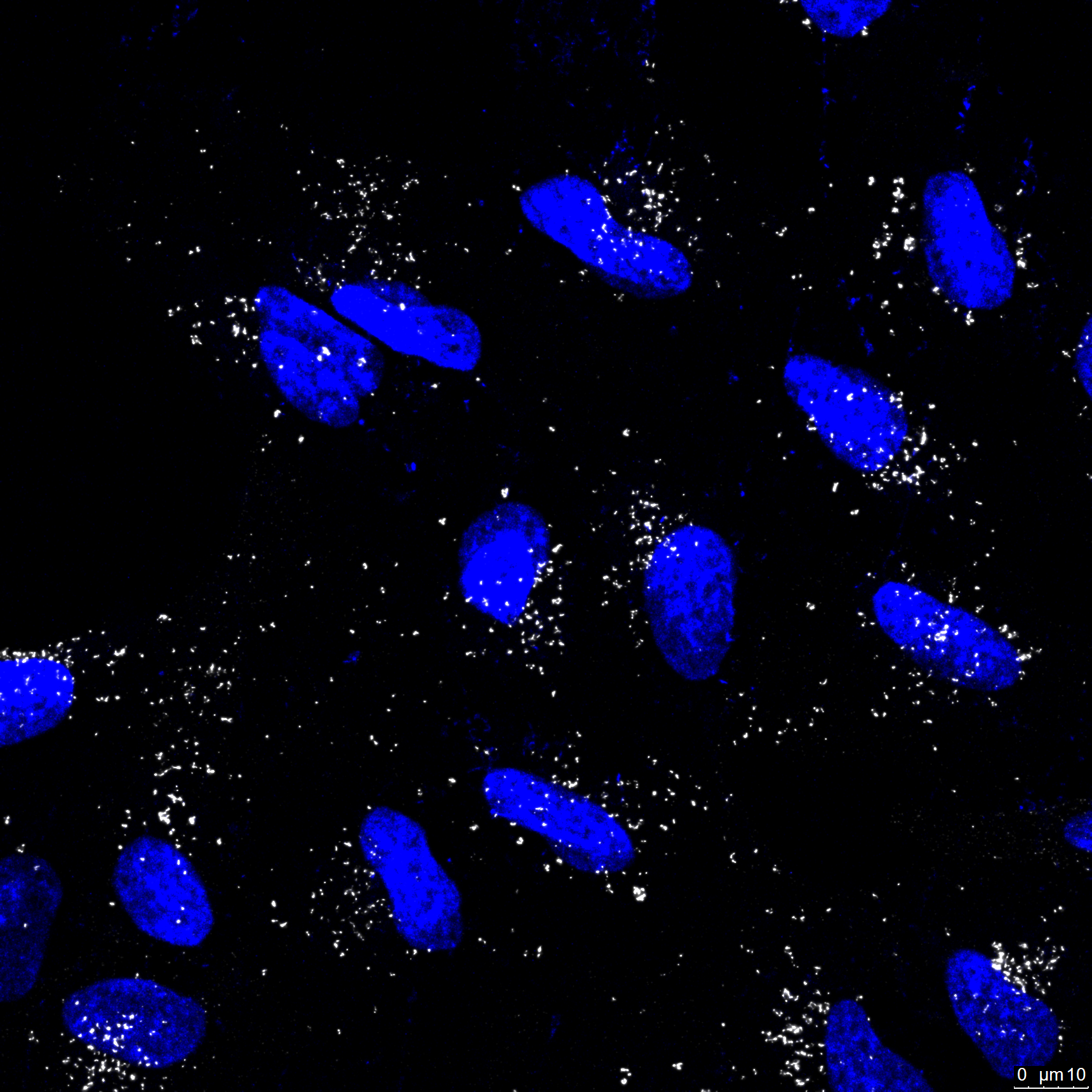

Supplement: Supplementary file 14 — Figure EV1 Source Data [file 44318_2025_654_MOESM14_ESM.zip › EV Figure 1/EV1F/EV1F-5-shVAPA.tif]

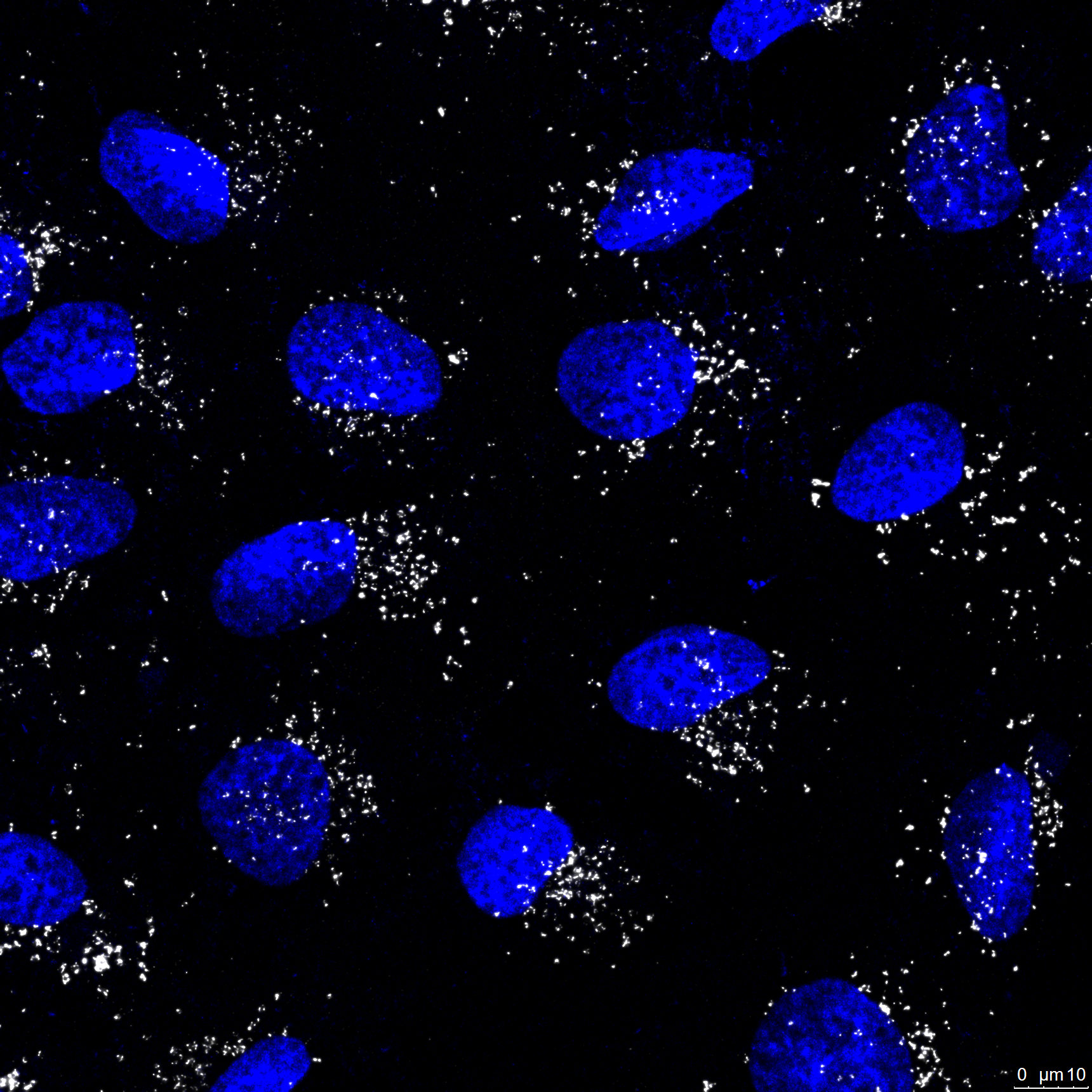

Supplement: Supplementary file 14 — Figure EV1 Source Data [file 44318_2025_654_MOESM14_ESM.zip › EV Figure 1/EV1F/EV1F-6-shVMA21.tif]

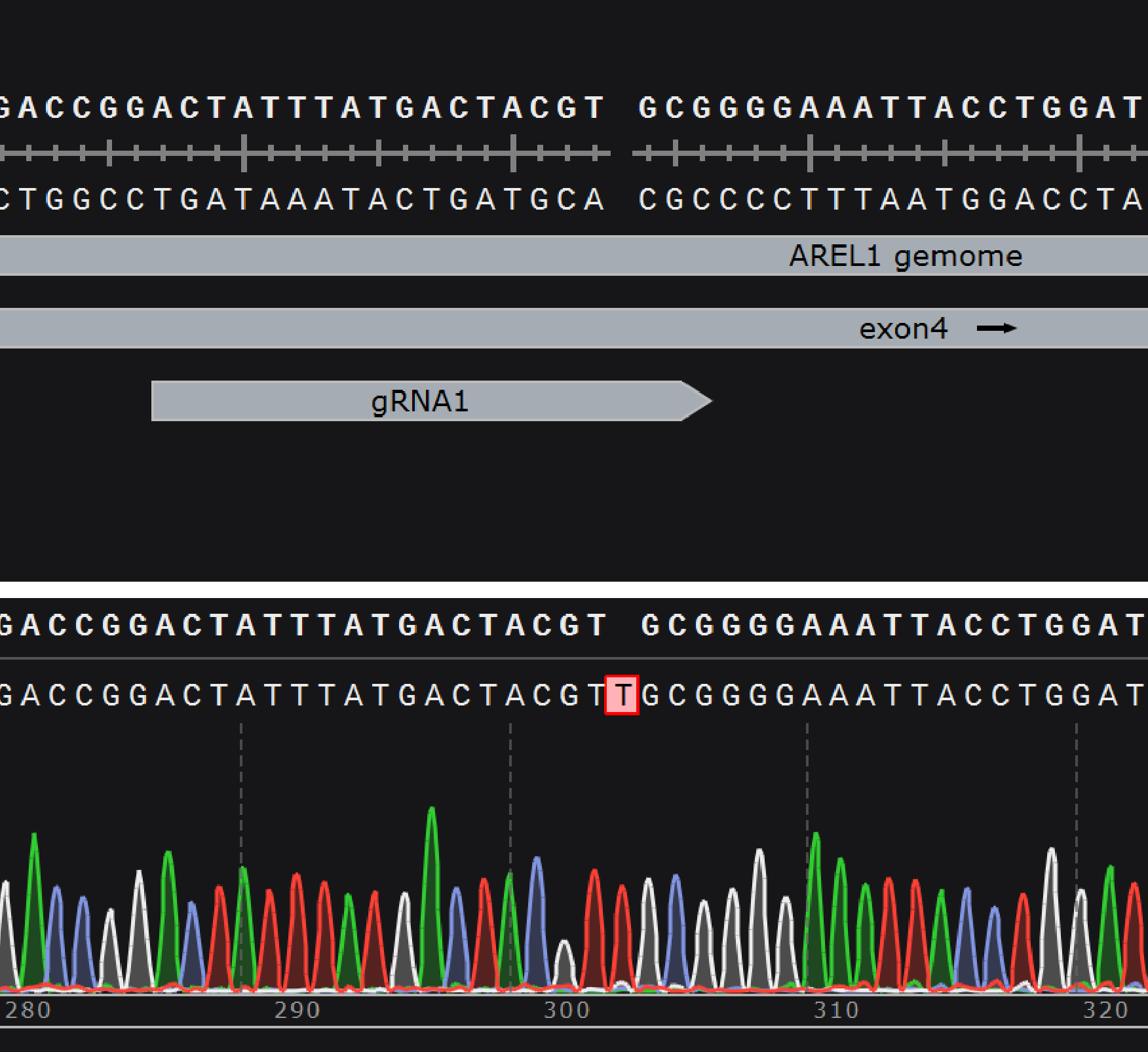

Supplement: Supplementary file 14 — Figure EV1 Source Data [file 44318_2025_654_MOESM14_ESM.zip › EV Figure 1/EV1H/EV1H.png]

|              |   |   |   |   |     |
|--------------|---|---|---|---|-----|
| Trypsin      | - | + | - | + |     |
| Triton X-100 | - | - | + | + | kDa |

AREL1

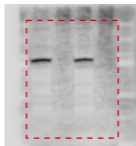

-250  
-150  
- 75  
- 50  
- 37

Calnexin

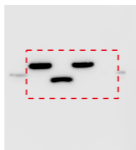

- 75  
- 50

Supplement: Supplementary file 15 — Figure EV2 Source Data [file 44318_2025_654_MOESM15_ESM.zip › EV Figure 2/EV2B/EV2B.pdf]

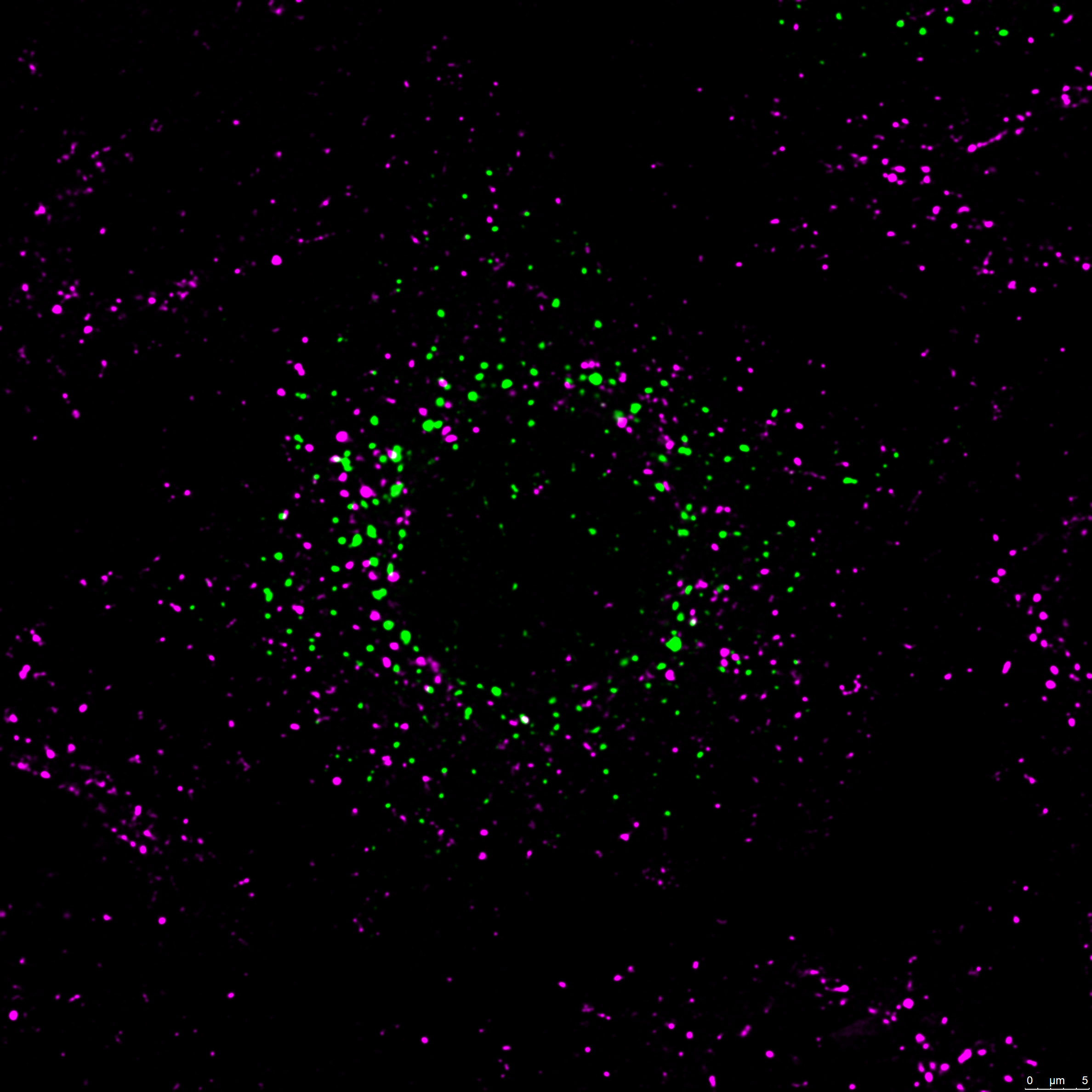

Supplement: Supplementary file 15 — Figure EV2 Source Data [file 44318_2025_654_MOESM15_ESM.zip › EV Figure 2/EV2D/EV2D-3-AREL1(1-705)-EGFP.tif]

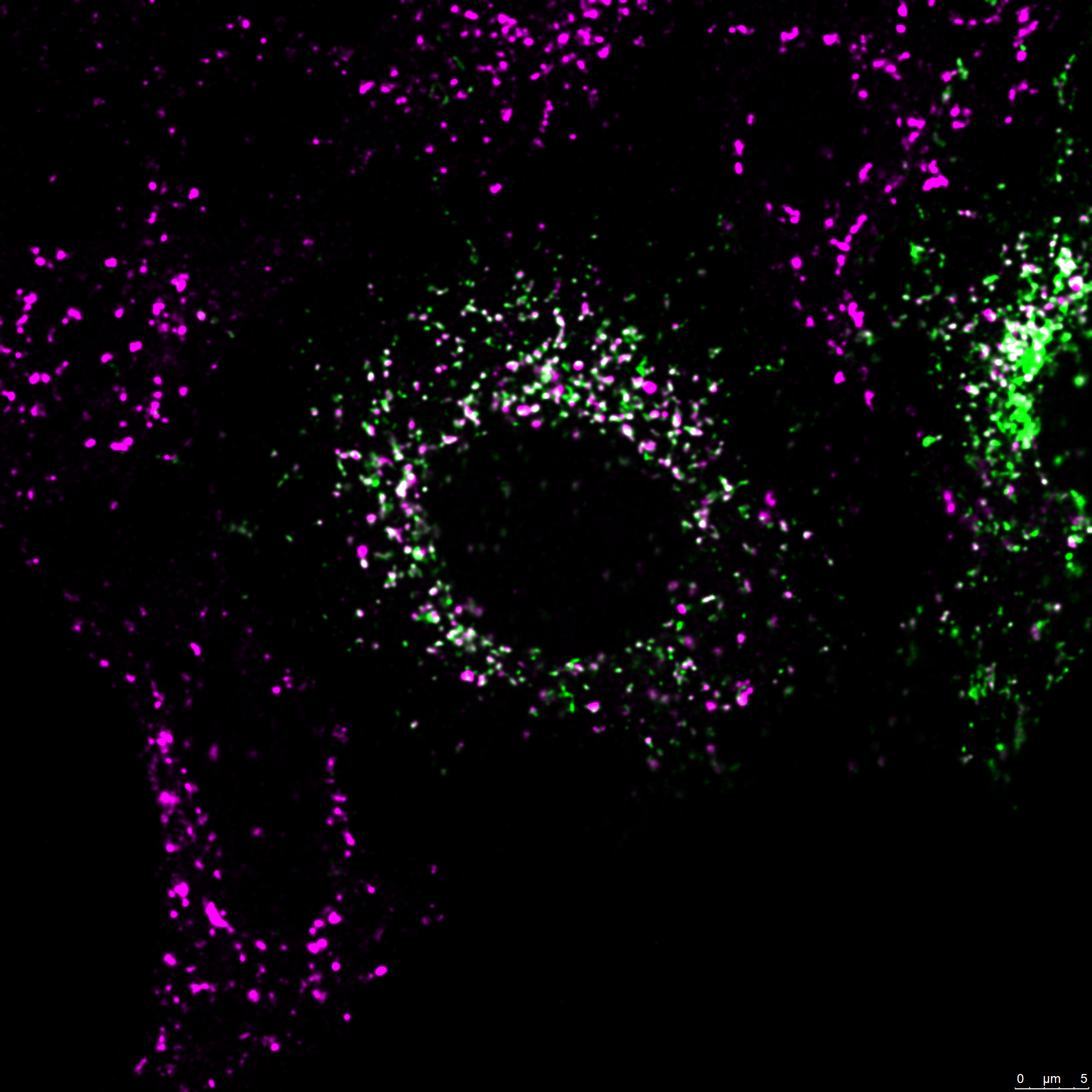

Supplement: Supplementary file 15 — Figure EV2 Source Data [file 44318_2025_654_MOESM15_ESM.zip › EV Figure 2/EV2D/EV2D-2-AREL1(1-712)-EGFP.tif]

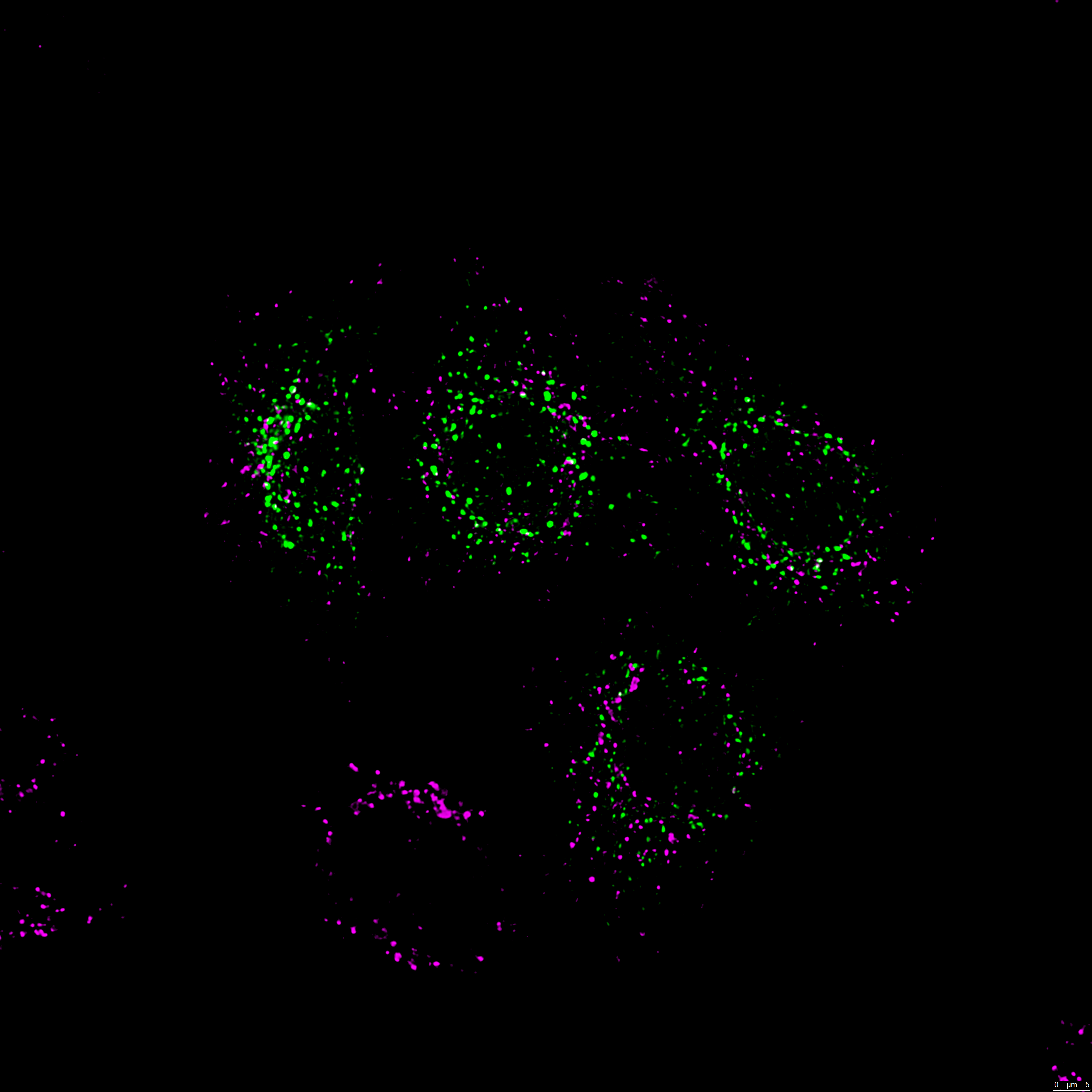

Supplement: Supplementary file 15 — Figure EV2 Source Data [file 44318_2025_654_MOESM15_ESM.zip › EV Figure 2/EV2D/EV2D-4-AREL1(1-668)-EGFP.tif]

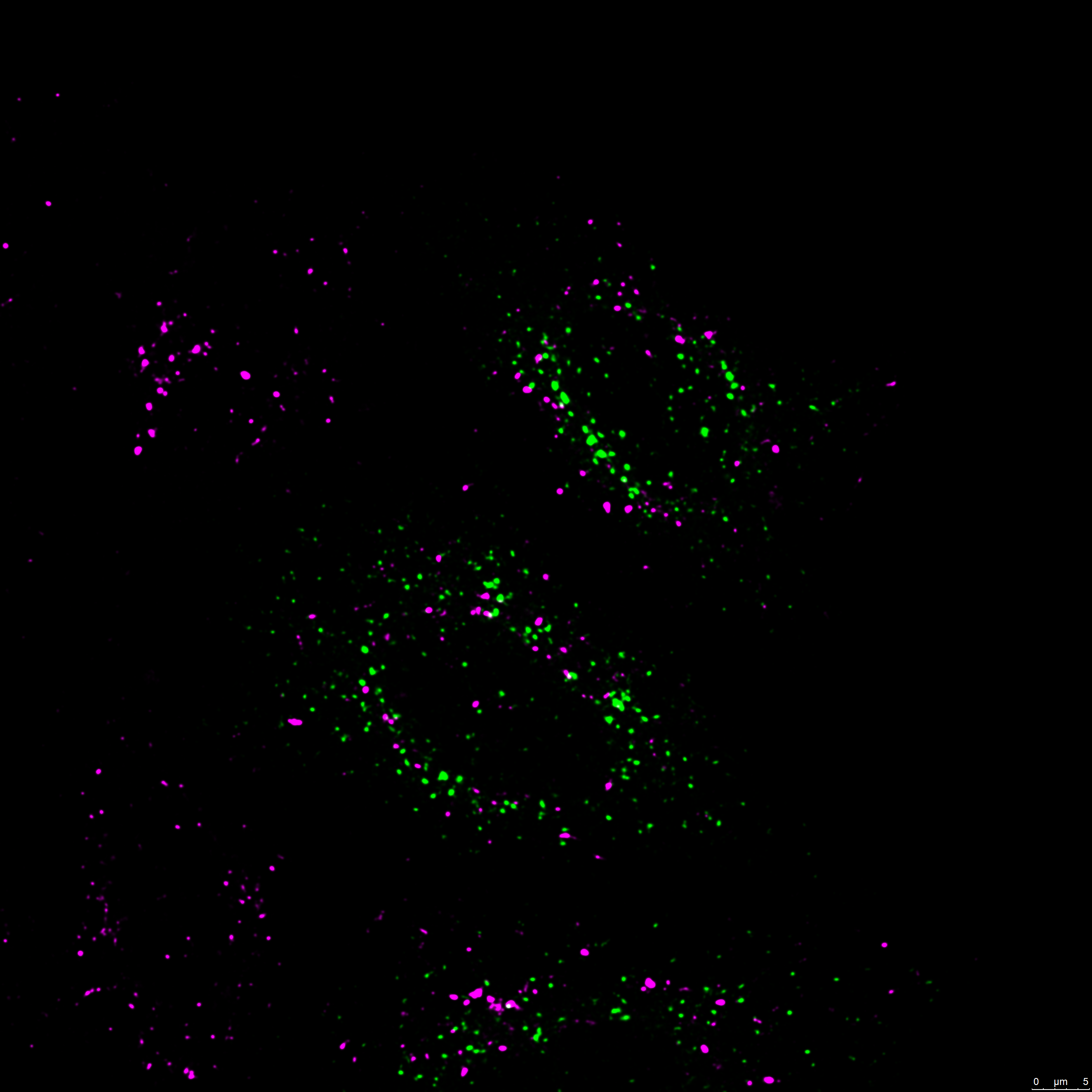

Supplement: Supplementary file 15 — Figure EV2 Source Data [file 44318_2025_654_MOESM15_ESM.zip › EV Figure 2/EV2D/EV2D-6-AREL1(1-482)-EGFP.tif]

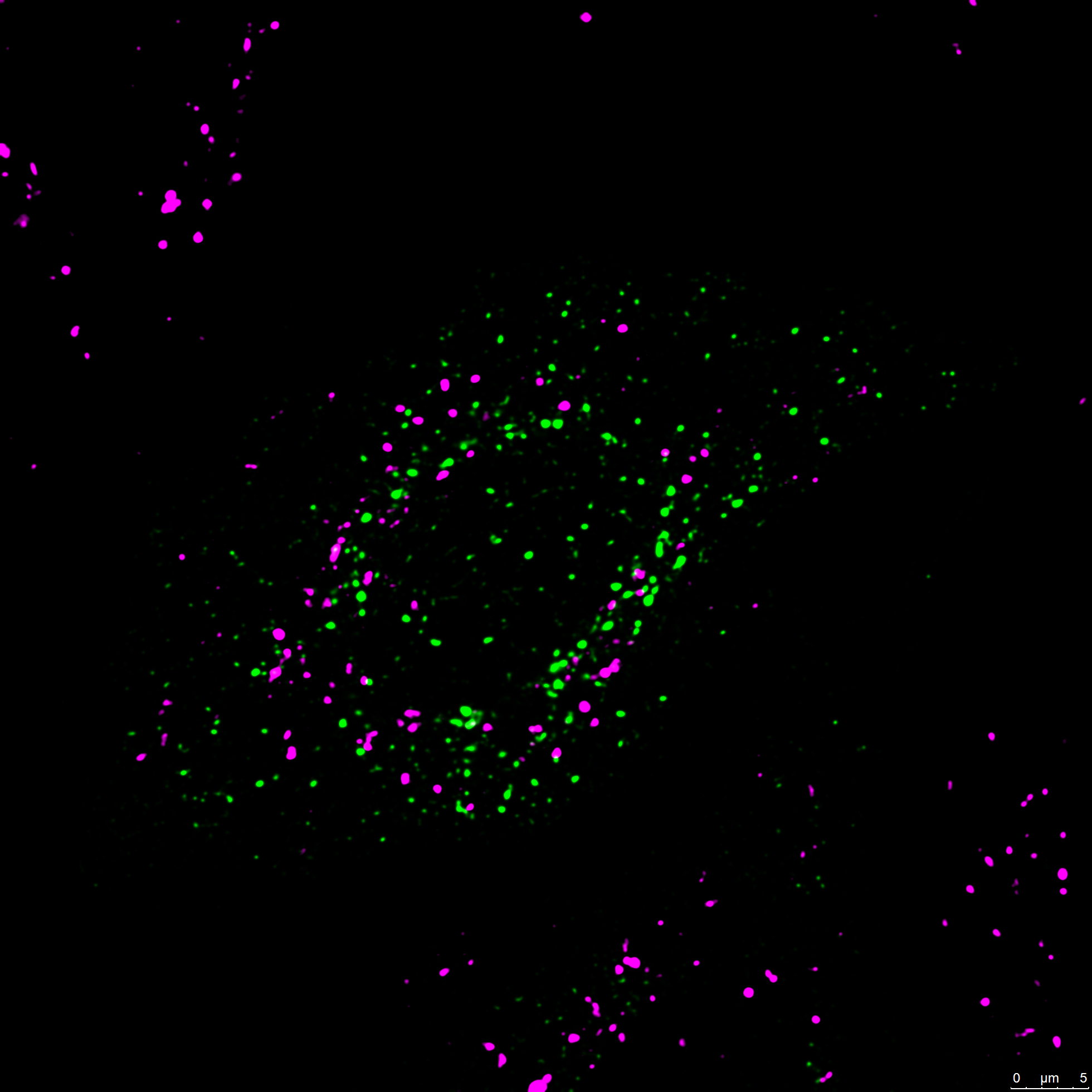

Supplement: Supplementary file 15 — Figure EV2 Source Data [file 44318_2025_654_MOESM15_ESM.zip › EV Figure 2/EV2D/EV2D-5-AREL1(1-589)-EGFP.tif]

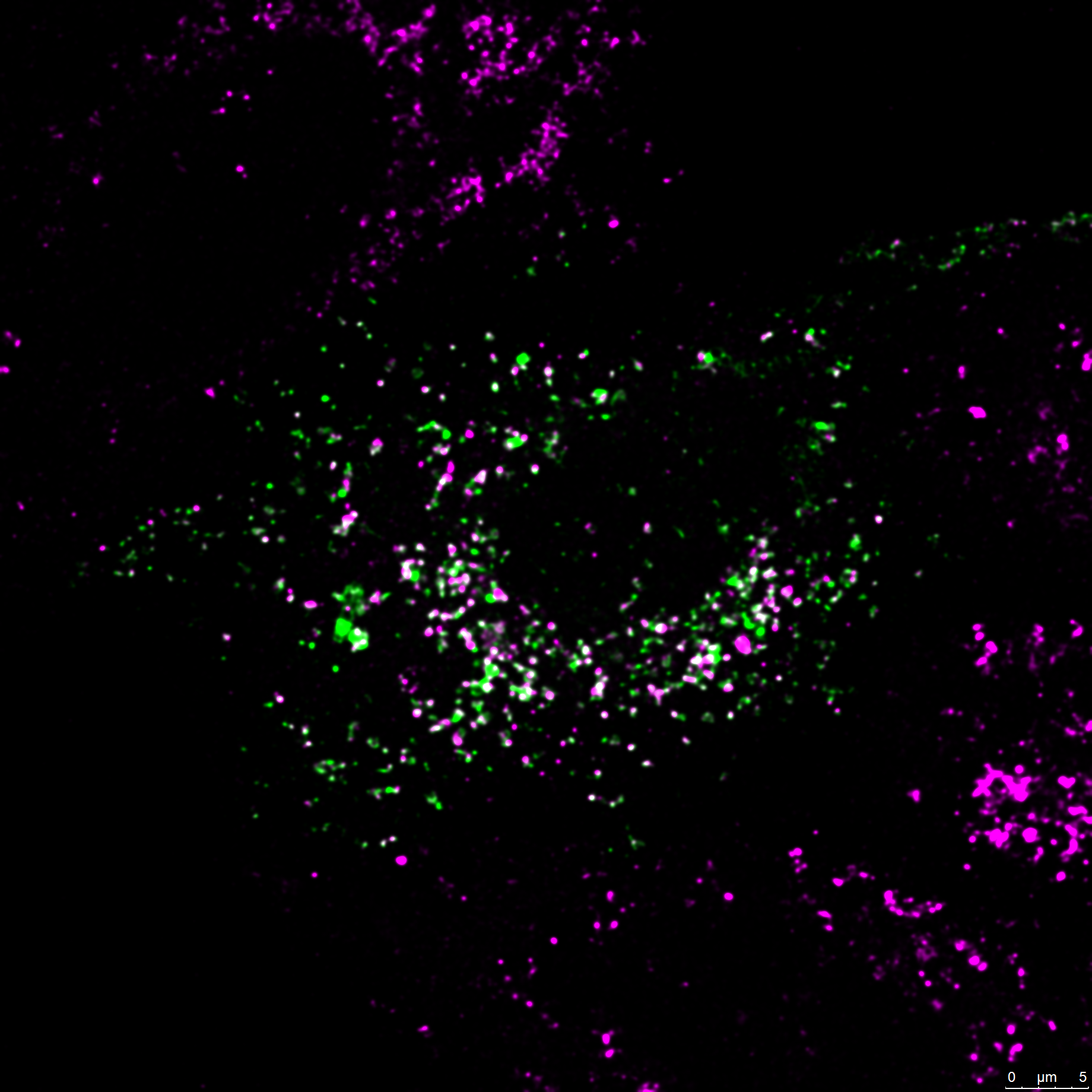

Supplement: Supplementary file 15 — Figure EV2 Source Data [file 44318_2025_654_MOESM15_ESM.zip › EV Figure 2/EV2D/EV2D-1-AREL1(WT)-EGFP.tif]

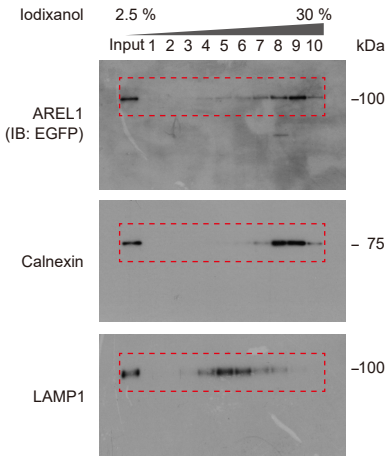

Supplement: Supplementary file 15 — Figure EV2 Source Data [file 44318_2025_654_MOESM15_ESM.zip › EV Figure 2/EV2A/EV2A.pdf]

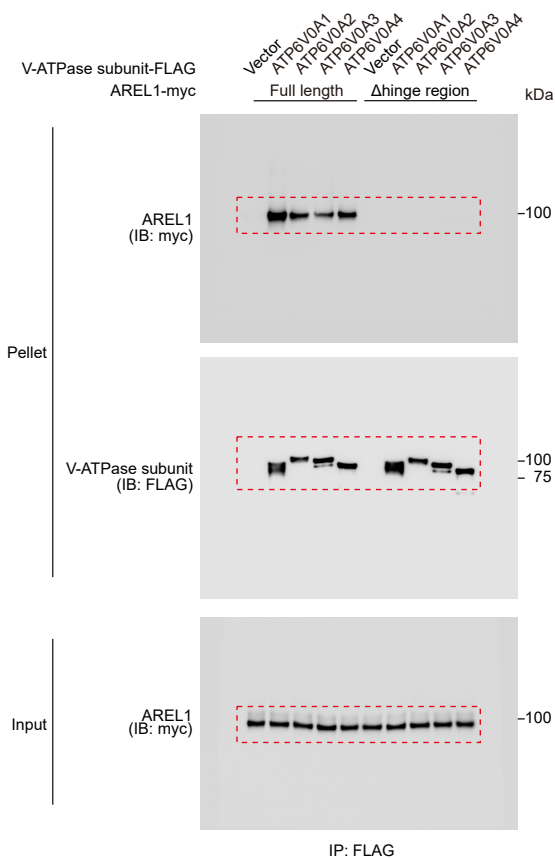

Supplement: Supplementary file 16 — Figure EV3 Source Data [file 44318_2025_654_MOESM16_ESM.zip › EV Figure 3/EV3G/EV3G.pdf]

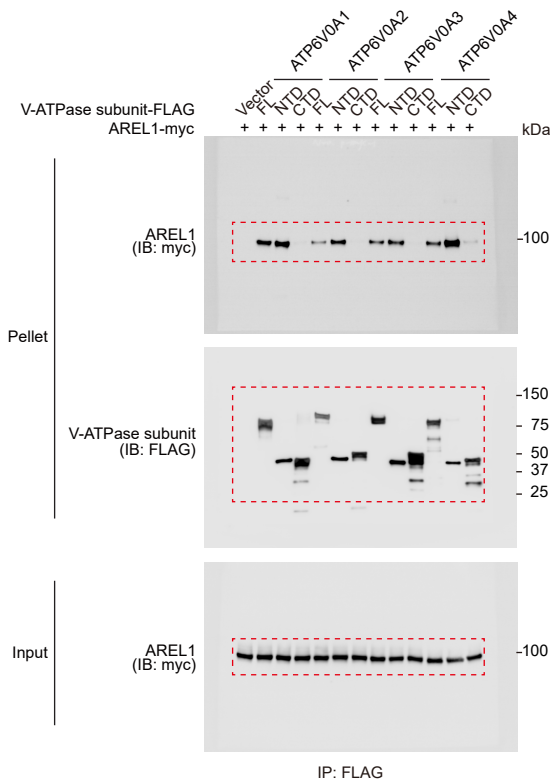

Supplement: Supplementary file 16 — Figure EV3 Source Data [file 44318_2025_654_MOESM16_ESM.zip › EV Figure 3/EV3F/EV3F.pdf]

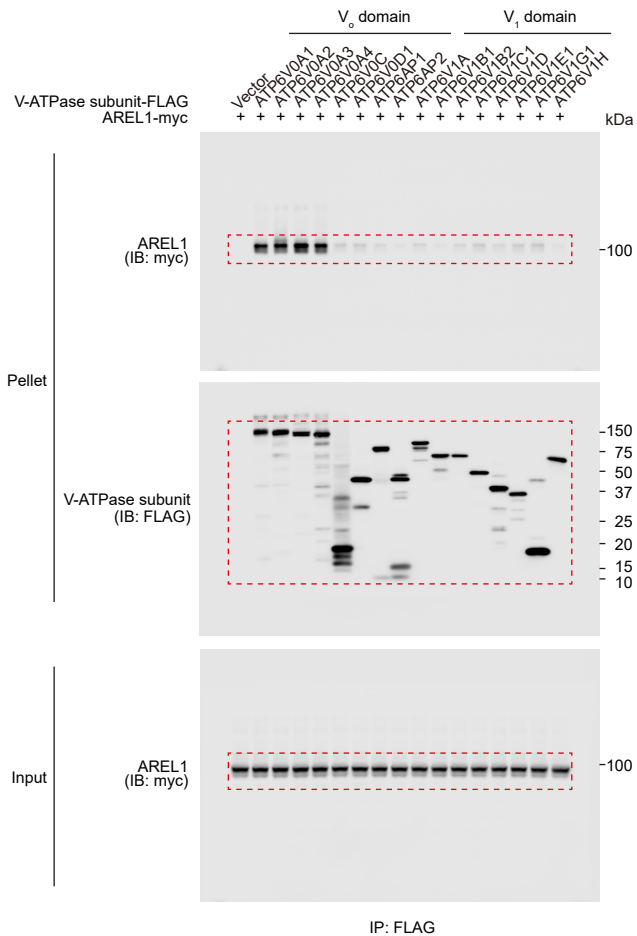

Supplement: Supplementary file 16 — Figure EV3 Source Data [file 44318_2025_654_MOESM16_ESM.zip › EV Figure 3/EV3D/EV3D.pdf]

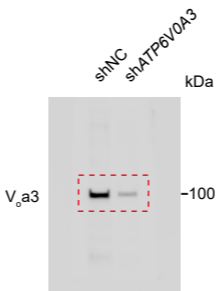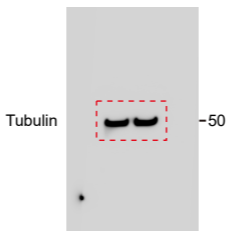

Supplement: Supplementary file 16 — Figure EV3 Source Data [file 44318_2025_654_MOESM16_ESM.zip › EV Figure 3/EV3K/EV3K.pdf]

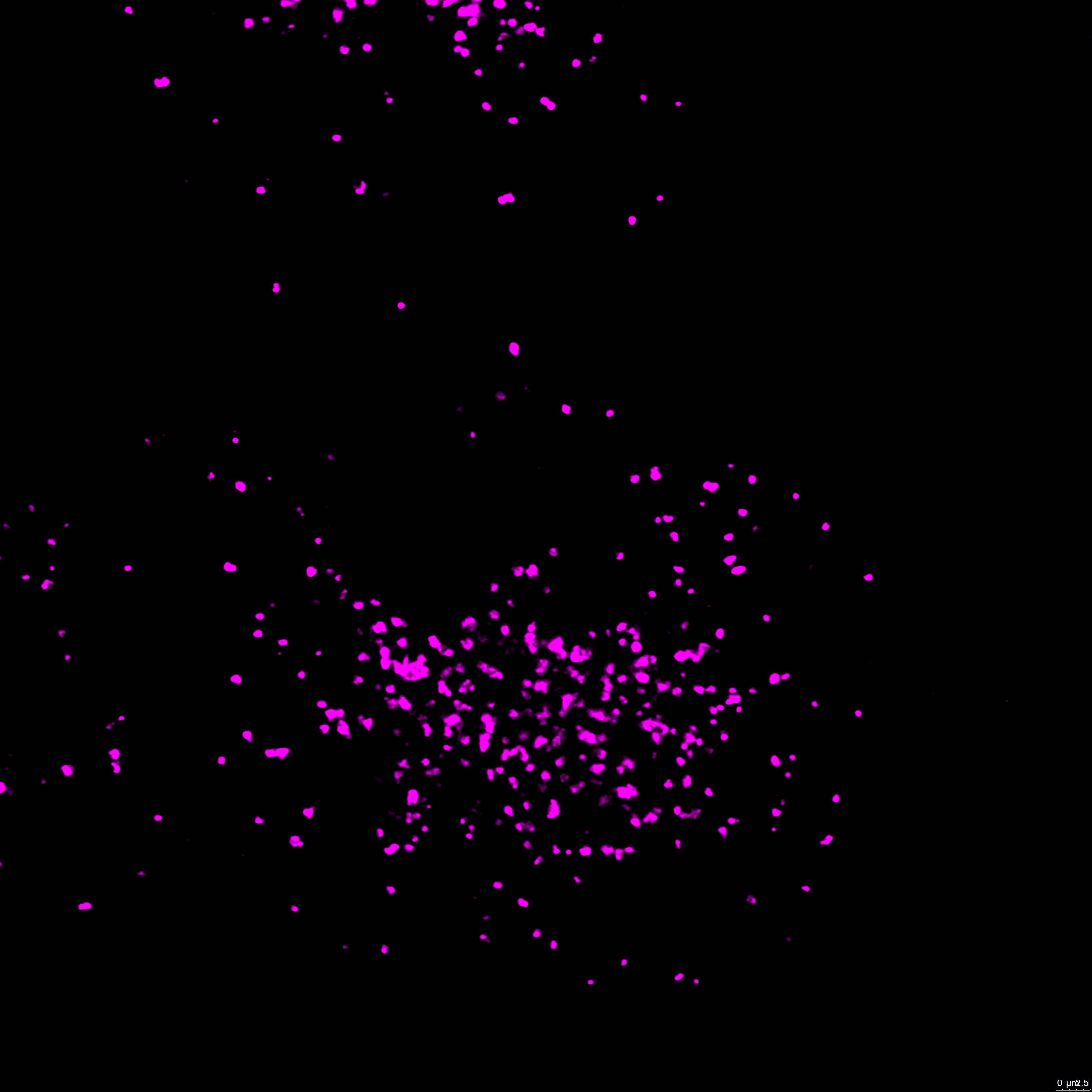

Supplement: Supplementary file 17 — Figure EV4 Source Data [file 44318_2025_654_MOESM17_ESM.zip › EV Figure 4/EV4G/EV4G-1-Hela WT-LAMP1.tif]

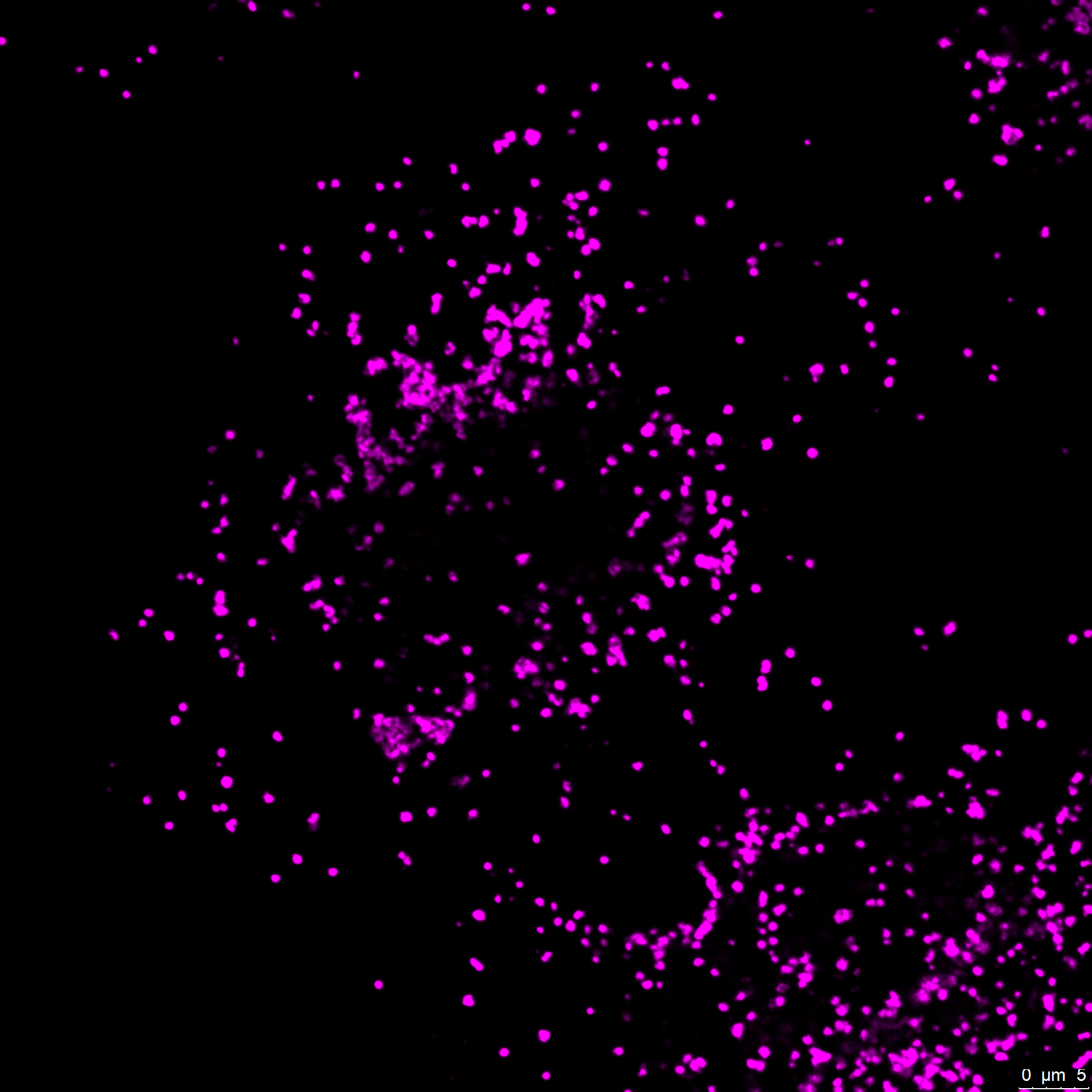

Supplement: Supplementary file 17 — Figure EV4 Source Data [file 44318_2025_654_MOESM17_ESM.zip › EV Figure 4/EV4G/EV4G-2-Hela KO-LAMP1.tif]

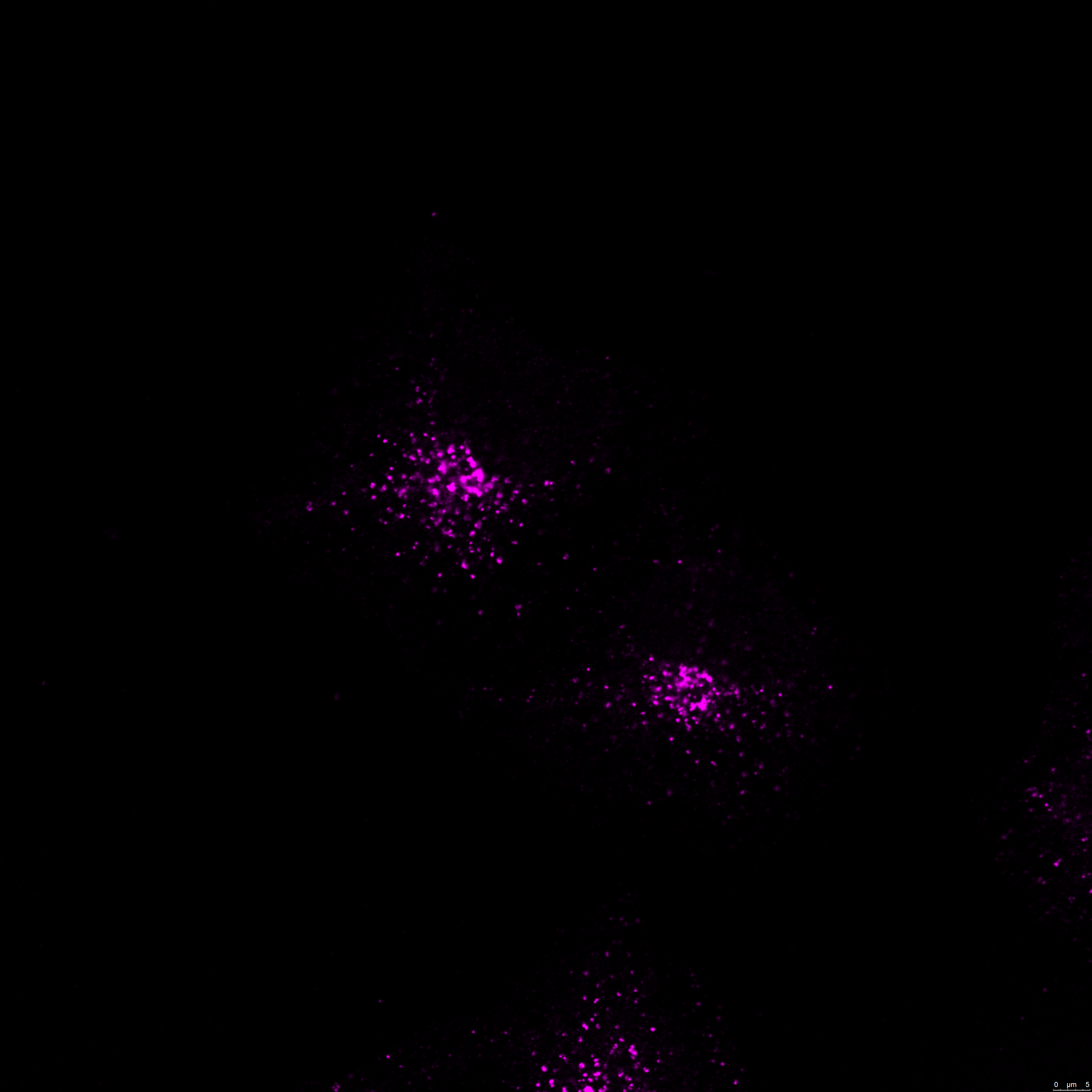

Supplement: Supplementary file 17 — Figure EV4 Source Data [file 44318_2025_654_MOESM17_ESM.zip › EV Figure 4/EV4I/EV4I-2-Hela KO-EEA1.tif]

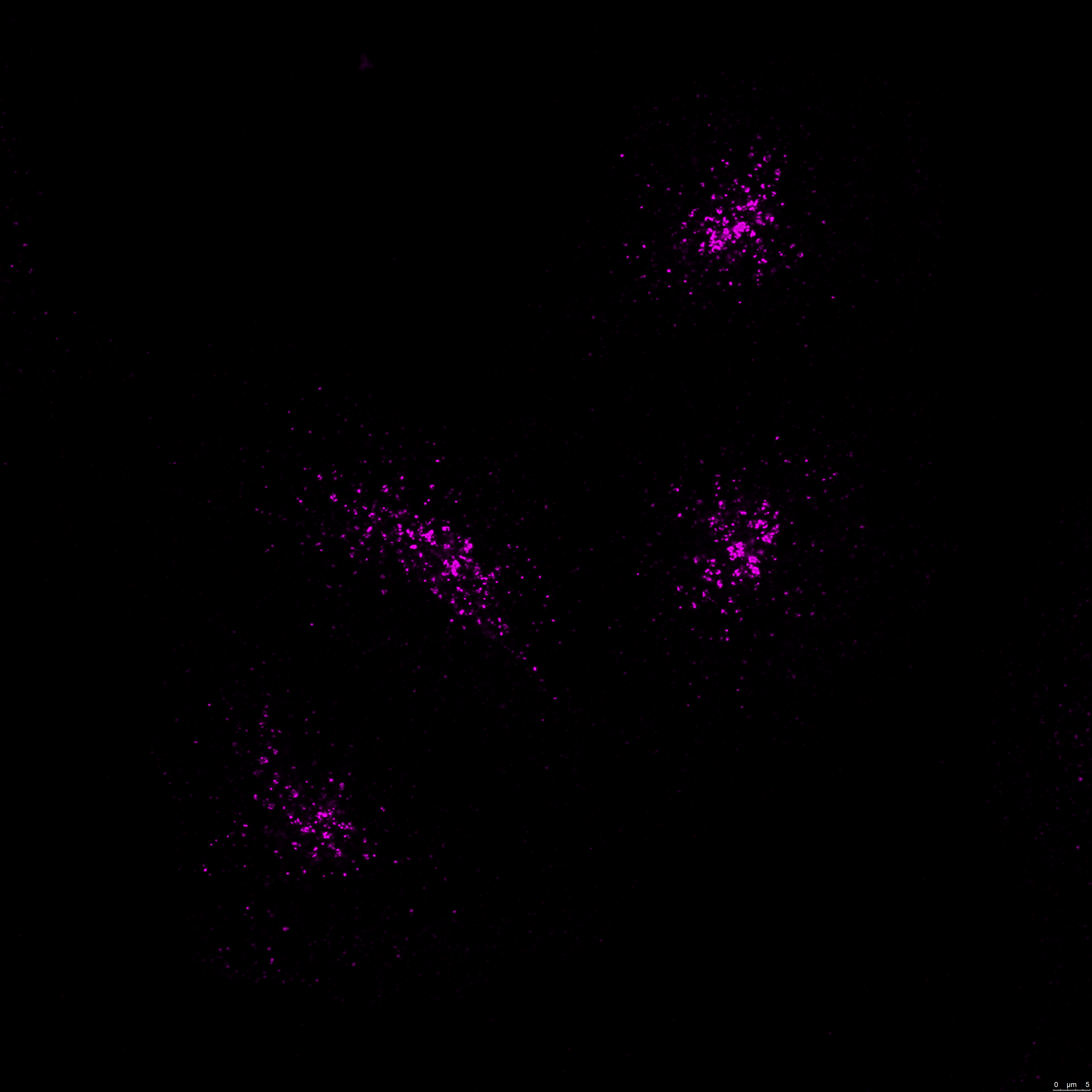

Supplement: Supplementary file 17 — Figure EV4 Source Data [file 44318_2025_654_MOESM17_ESM.zip › EV Figure 4/EV4I/EV4I-1-Hela WT-EEA1.tif]

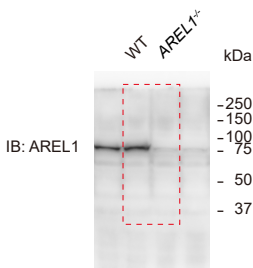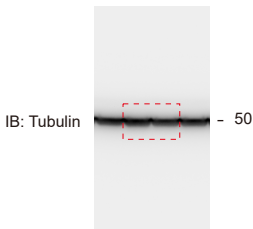

Supplement: Supplementary file 17 — Figure EV4 Source Data [file 44318_2025_654_MOESM17_ESM.zip › EV Figure 4/EV4F/EV4F.pdf]

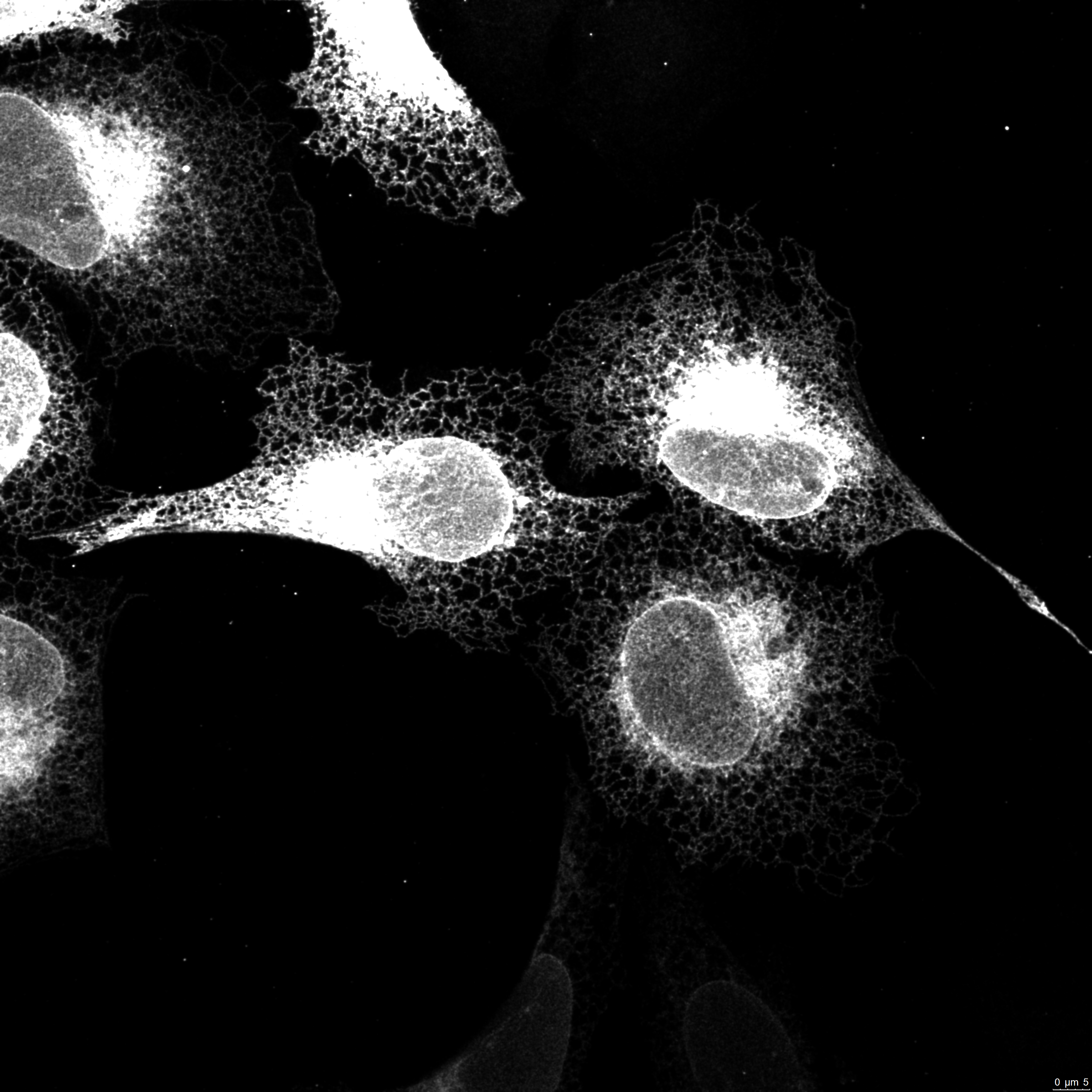

Supplement: Supplementary file 17 — Figure EV4 Source Data [file 44318_2025_654_MOESM17_ESM.zip › EV Figure 4/EV4A/EV4A-2-KO_EGFP-Sec61╬▓.tif]

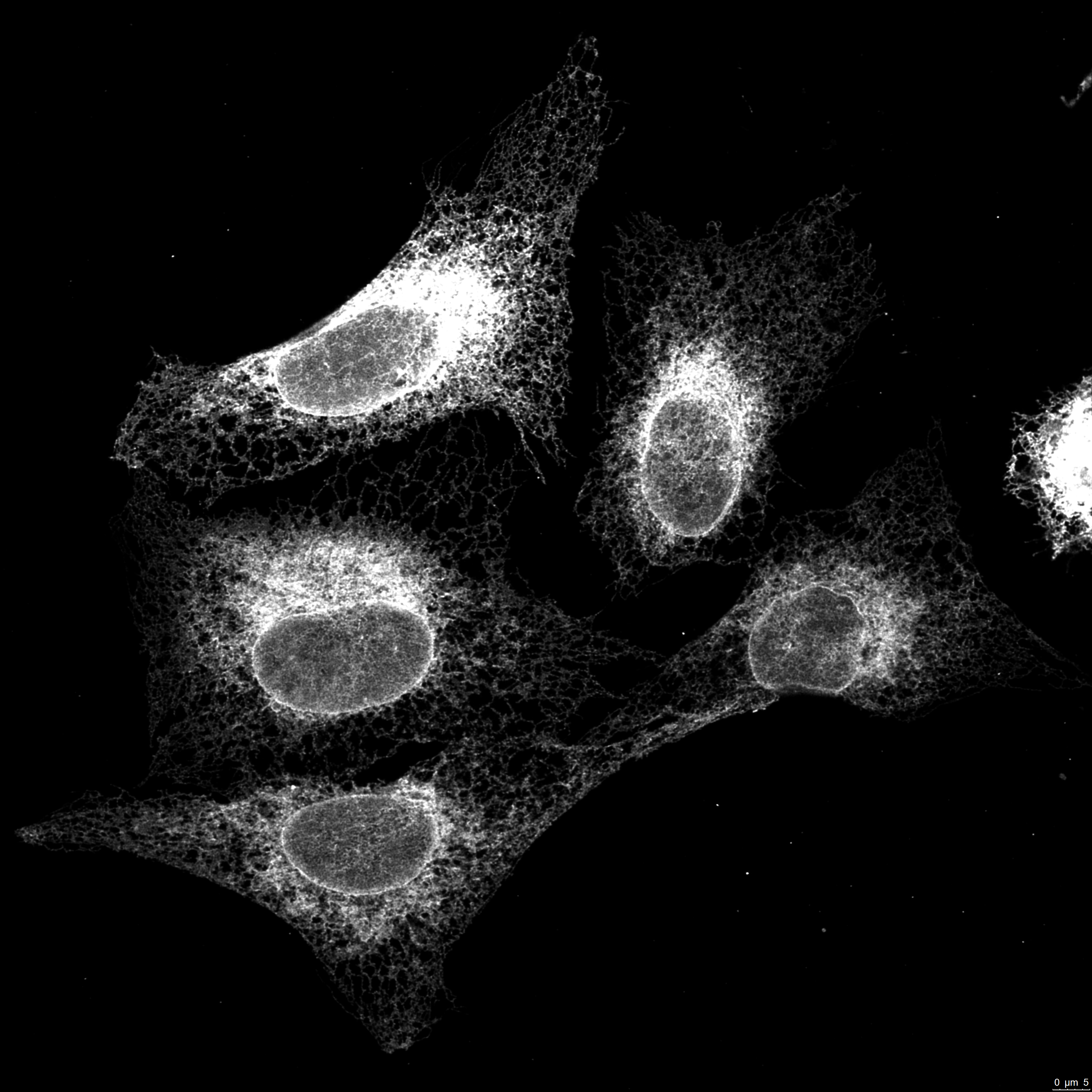

Supplement: Supplementary file 17 — Figure EV4 Source Data [file 44318_2025_654_MOESM17_ESM.zip › EV Figure 4/EV4A/EV4A-1-WT_EGFP-Sec61╬▓.tif]

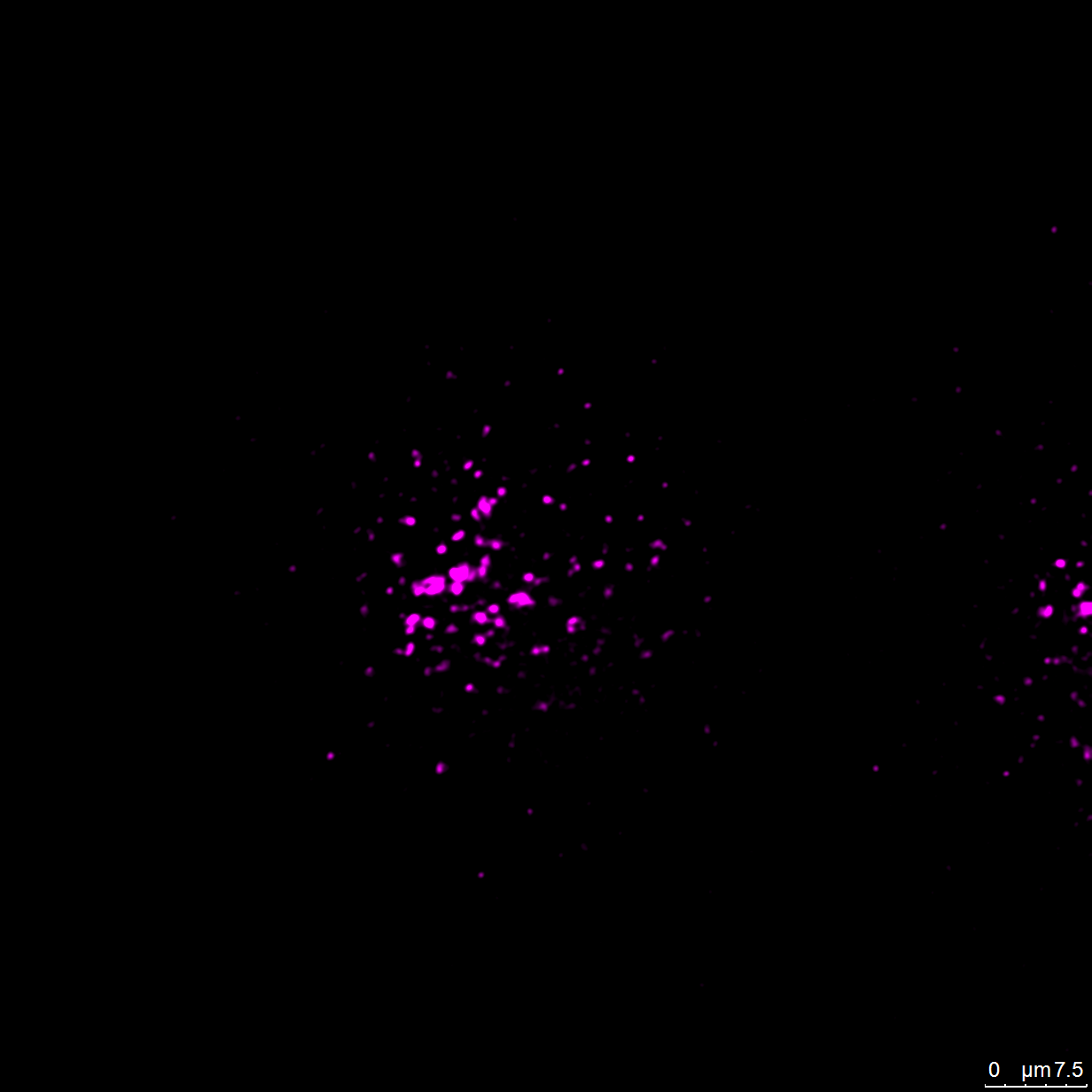

Supplement: Supplementary file 17 — Figure EV4 Source Data [file 44318_2025_654_MOESM17_ESM.zip › EV Figure 4/EV4C/EV4C-1-WT EEA1.tif]

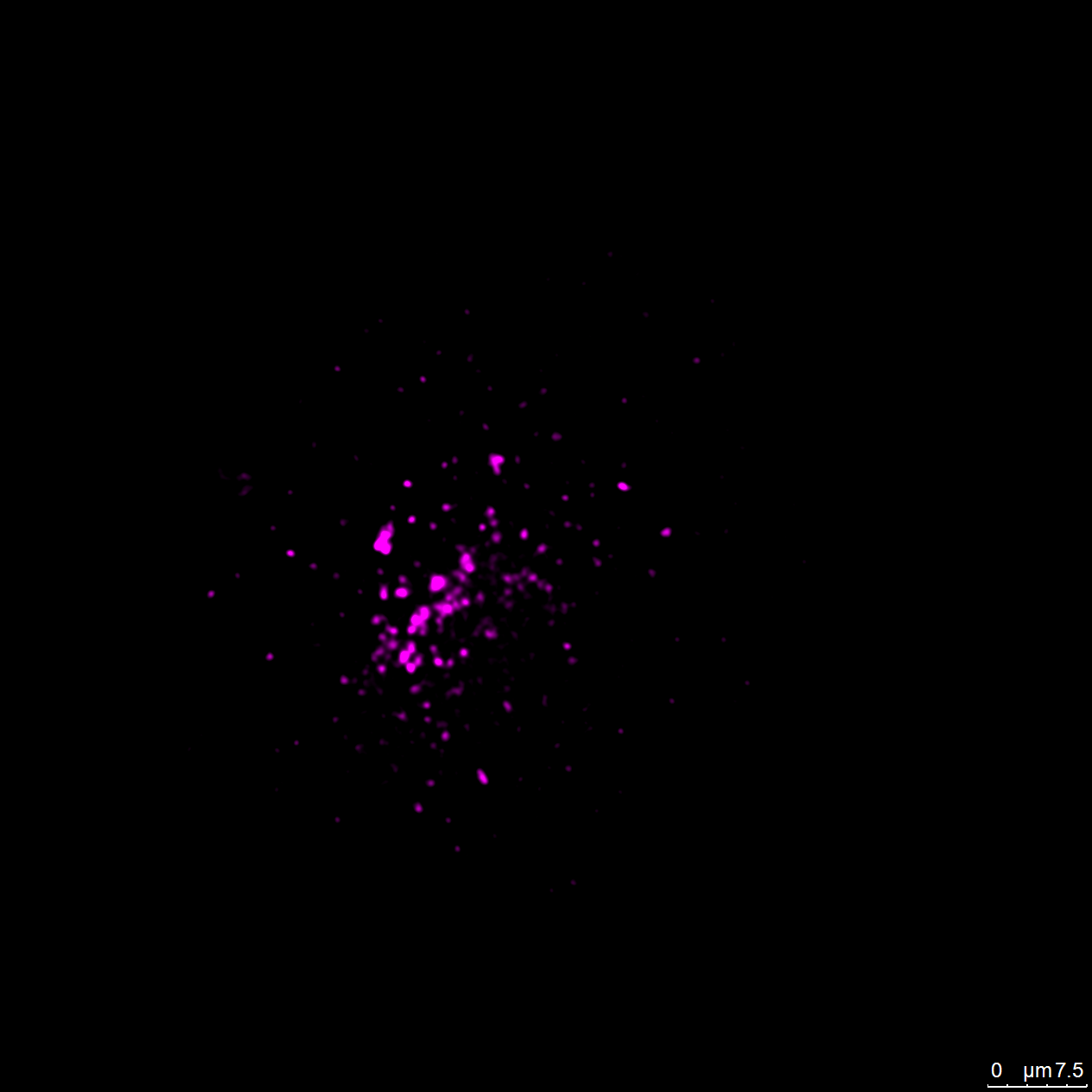

Supplement: Supplementary file 17 — Figure EV4 Source Data [file 44318_2025_654_MOESM17_ESM.zip › EV Figure 4/EV4C/EV4C-2-KO EEA1.tif]

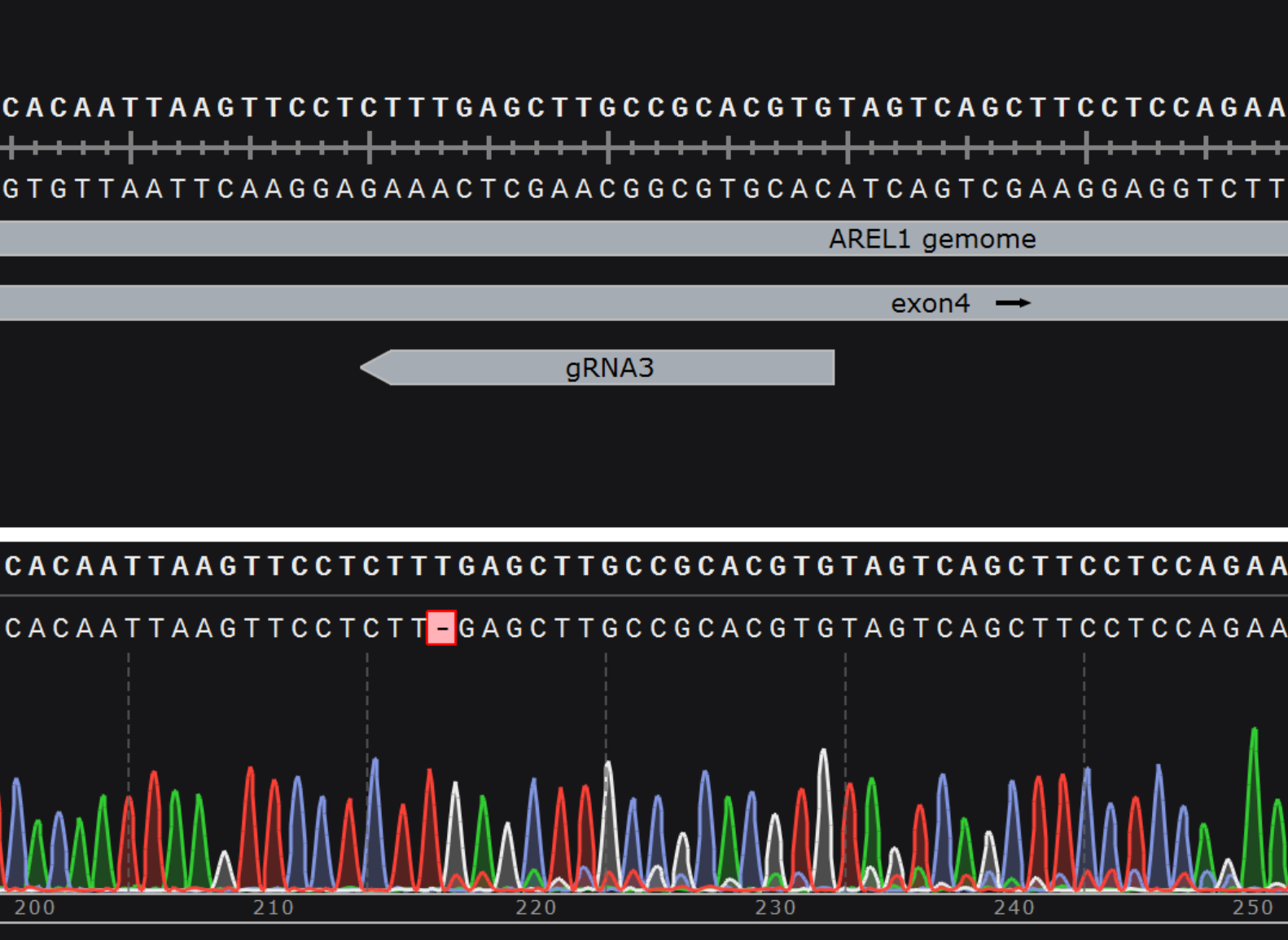

Supplement: Supplementary file 17 — Figure EV4 Source Data [file 44318_2025_654_MOESM17_ESM.zip › EV Figure 4/EV4E/EV4E.png]

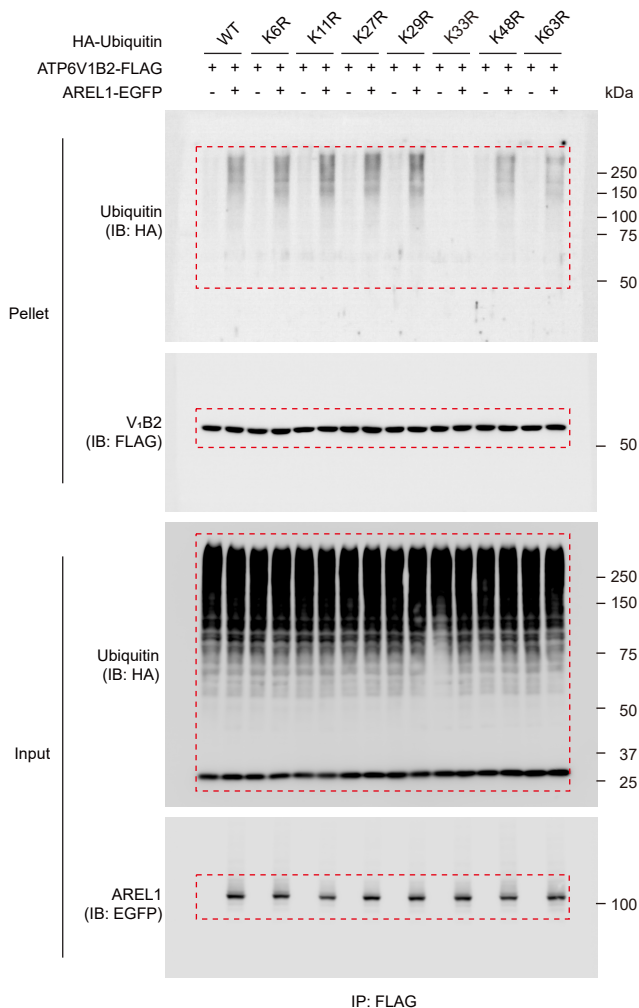

Supplement: Supplementary file 18 — Figure EV5 Source Data [file 44318_2025_654_MOESM18_ESM.zip › EV Figure 5/EV5B/EV5B.pdf]

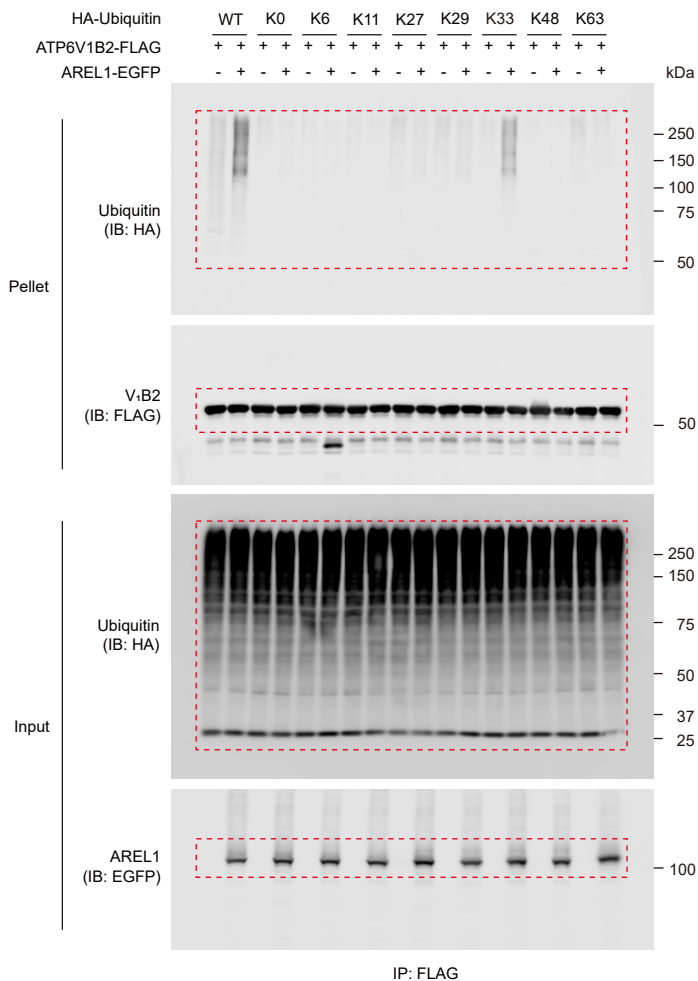

Supplement: Supplementary file 18 — Figure EV5 Source Data [file 44318_2025_654_MOESM18_ESM.zip › EV Figure 5/EV5C/EV5C.pdf]

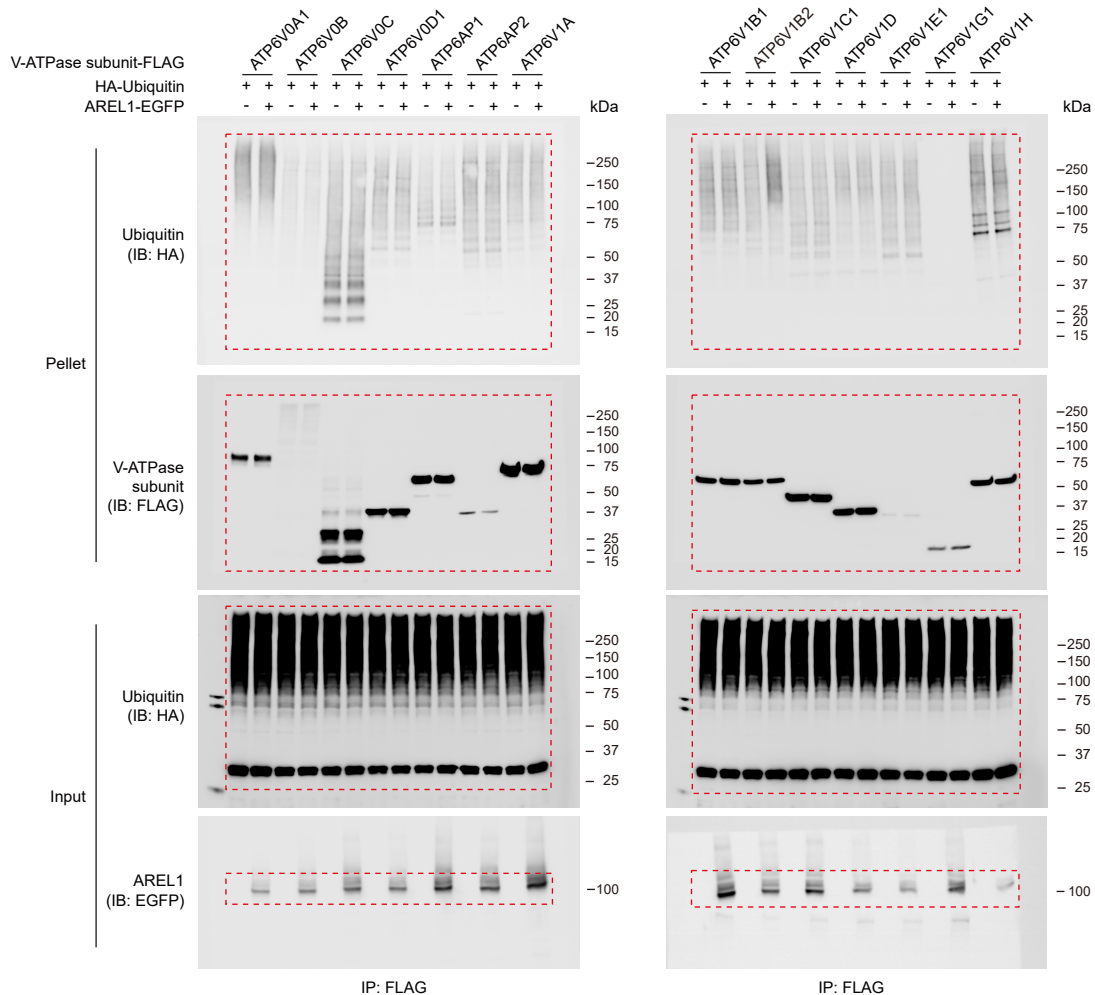

Supplement: Supplementary file 18 — Figure EV5 Source Data [file 44318_2025_654_MOESM18_ESM.zip › EV Figure 5/EV5A/EV5A.pdf]

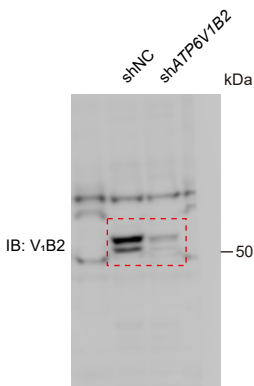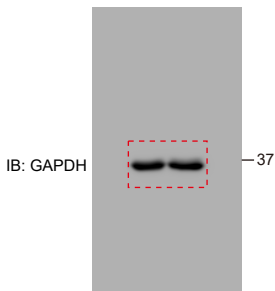

Supplement: Supplementary file 19 — Figure EV6 Source Data [file 44318_2025_654_MOESM19_ESM.zip › EV Figure 6/EV6C/EV6C.pdf]

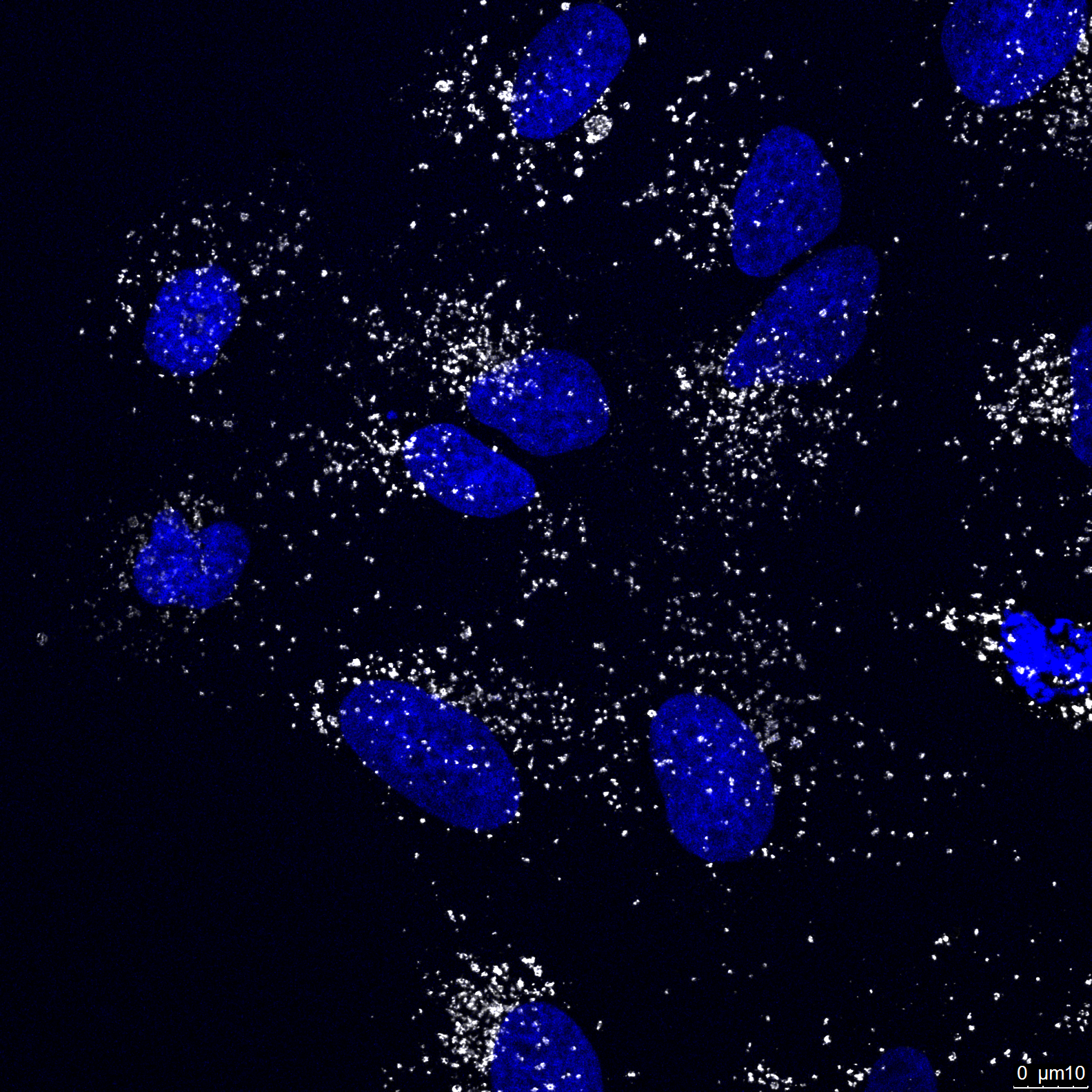

Supplement: Supplementary file 19 — Figure EV6 Source Data [file 44318_2025_654_MOESM19_ESM.zip › EV Figure 6/EV6D/EV6D-1-shNC-PLA.tif]

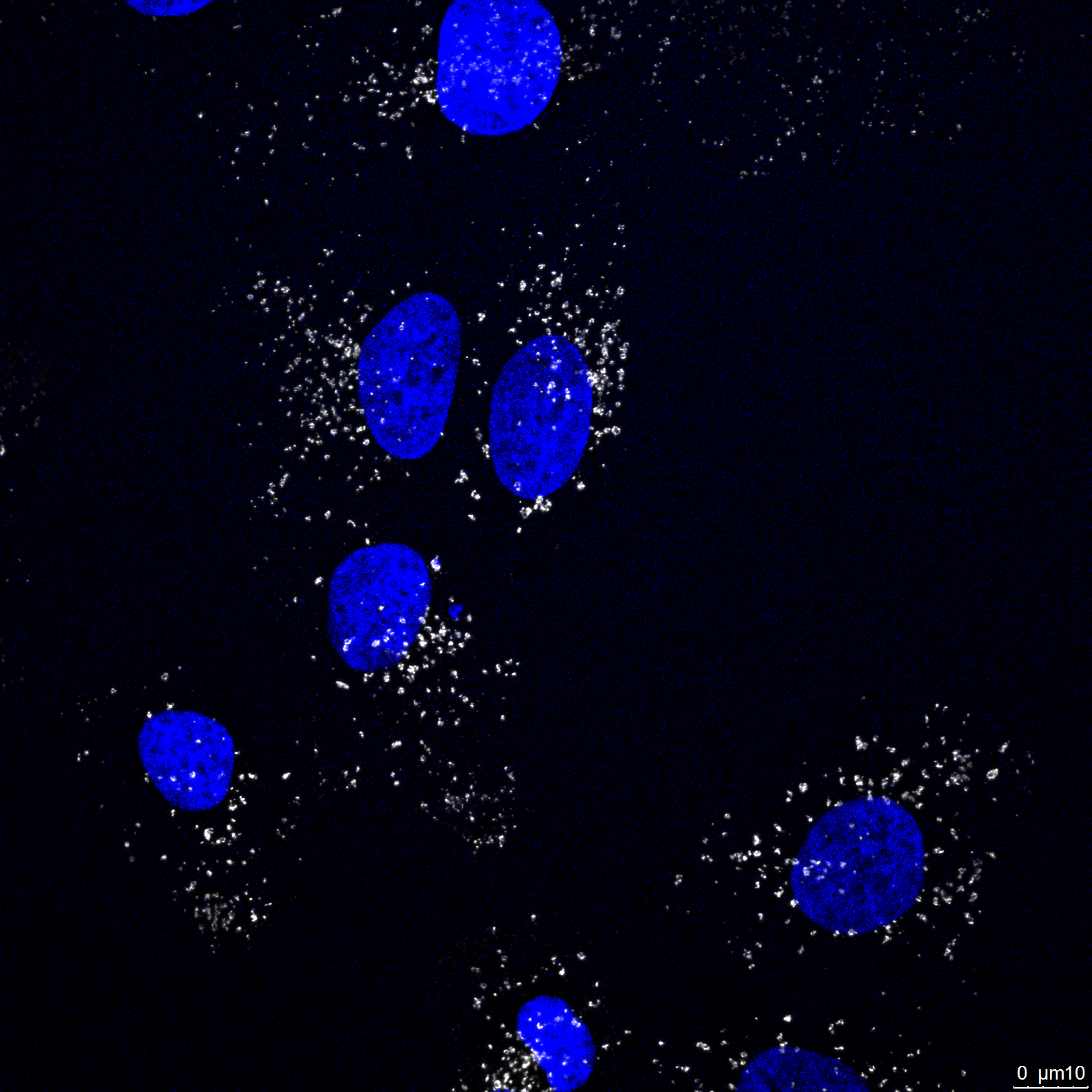

Supplement: Supplementary file 19 — Figure EV6 Source Data [file 44318_2025_654_MOESM19_ESM.zip › EV Figure 6/EV6D/EV6D-2-shATP6V1B2-PLA.tif]

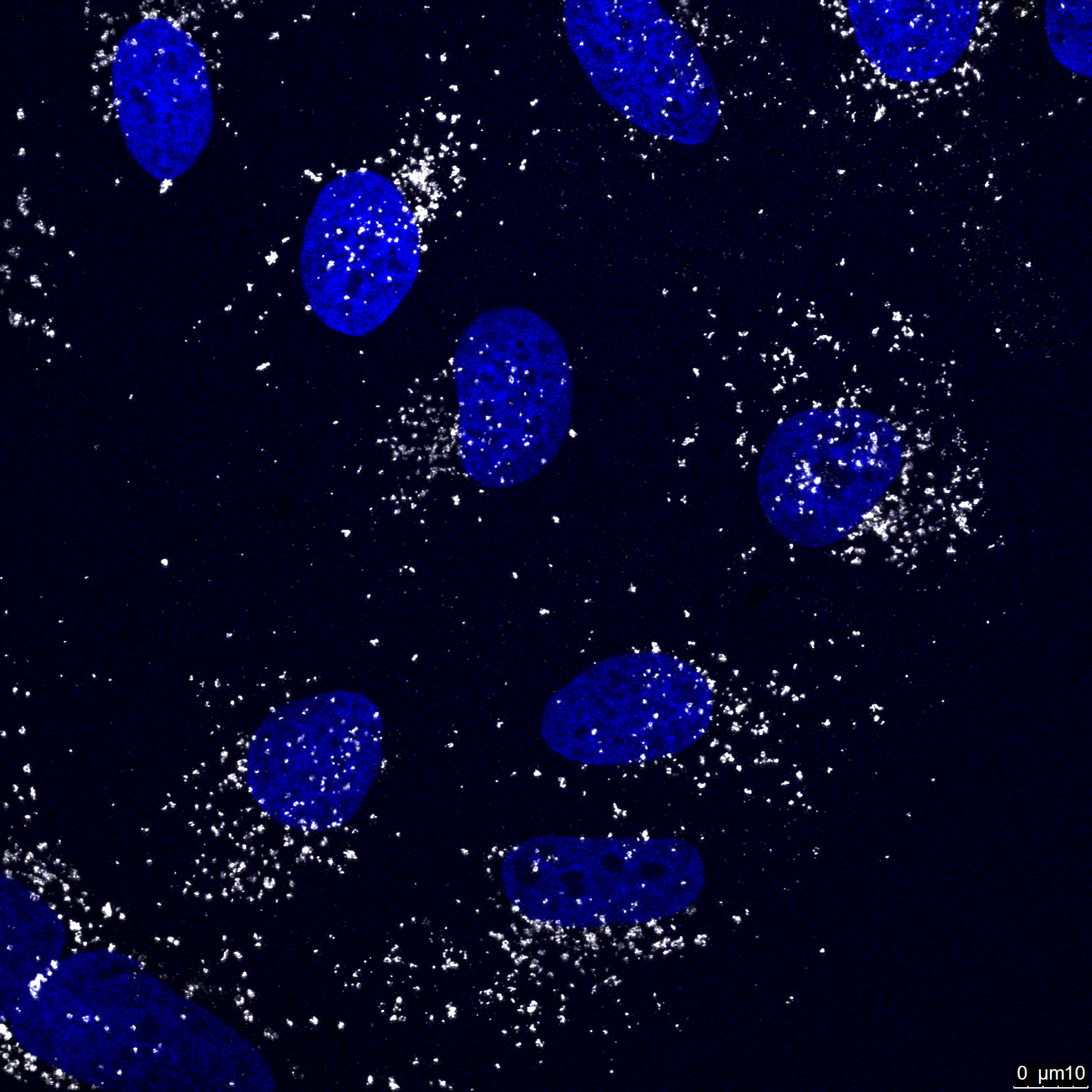

Supplement: Supplementary file 19 — Figure EV6 Source Data [file 44318_2025_654_MOESM19_ESM.zip › EV Figure 6/EV6I/EV6I-1-shNC-PLA.tif]

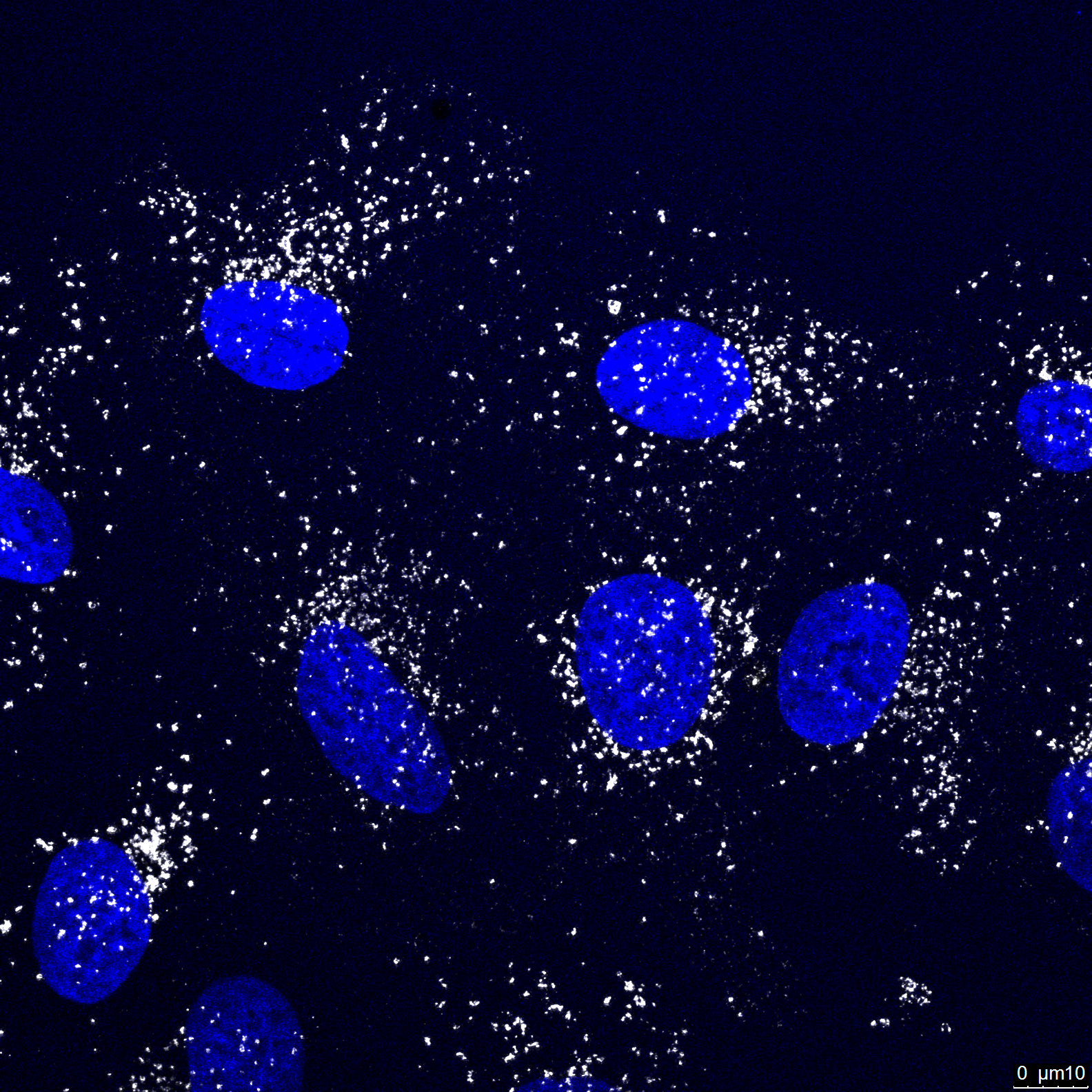

Supplement: Supplementary file 19 — Figure EV6 Source Data [file 44318_2025_654_MOESM19_ESM.zip › EV Figure 6/EV6I/EV6I-2-shZRANB1-PLA.tif]

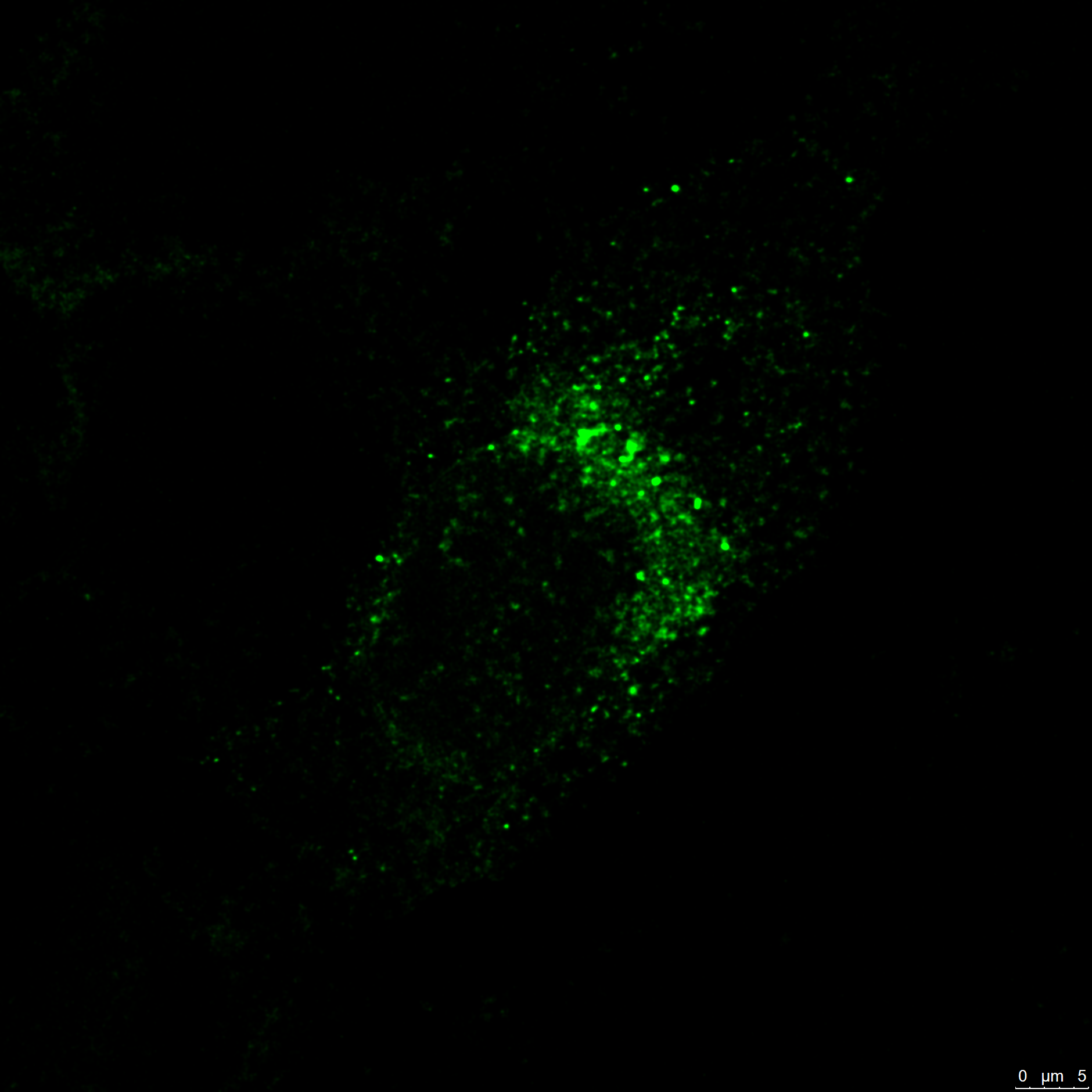

Supplement: Supplementary file 19 — Figure EV6 Source Data [file 44318_2025_654_MOESM19_ESM.zip › EV Figure 6/EV6F/EV6F-3-BFP-ZRANB1(C443S)-AREL1-EGFP.tif]

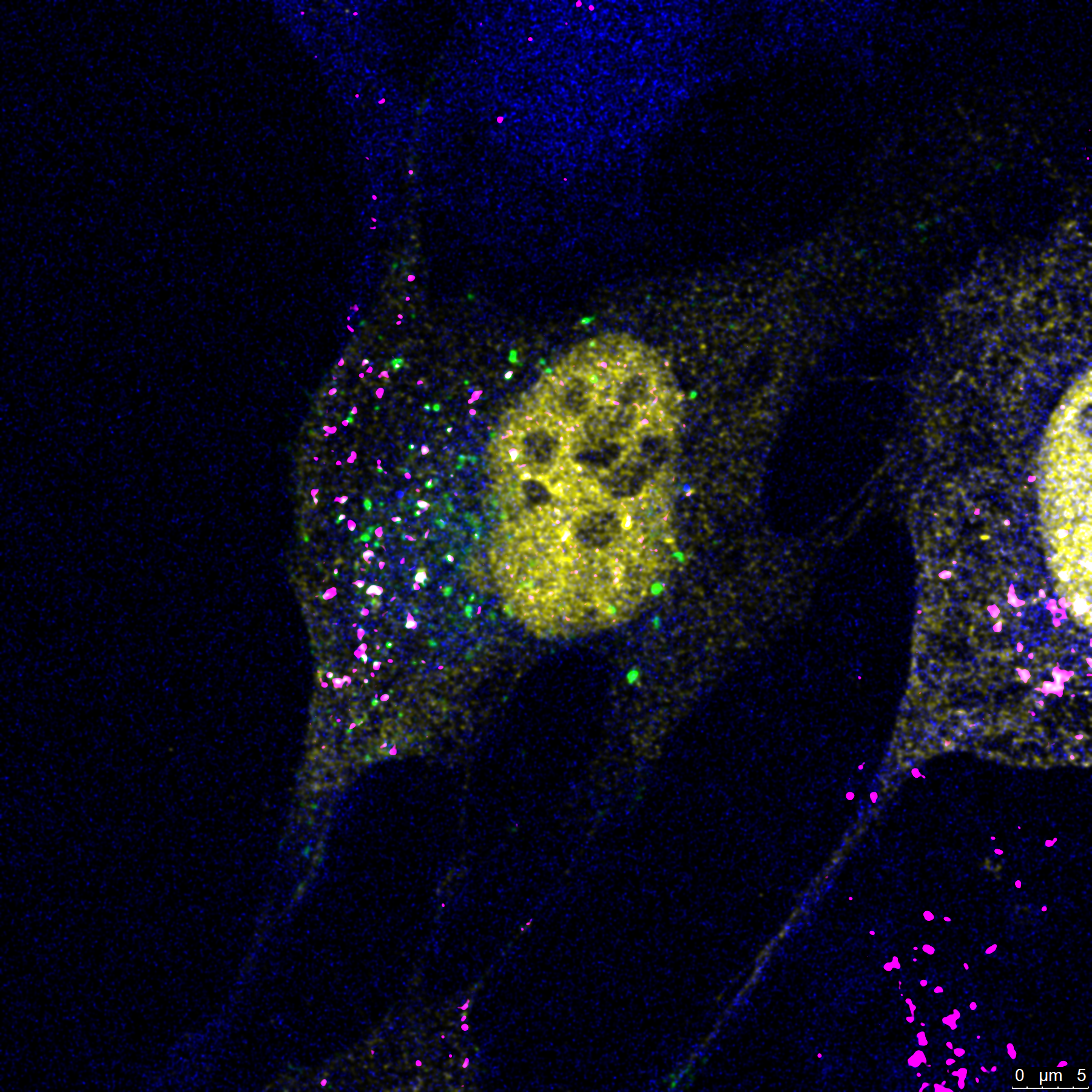

Supplement: Supplementary file 19 — Figure EV6 Source Data [file 44318_2025_654_MOESM19_ESM.zip › EV Figure 6/EV6F/EV6F-2-BFP-ZRANB1(WT)-merge.tif]

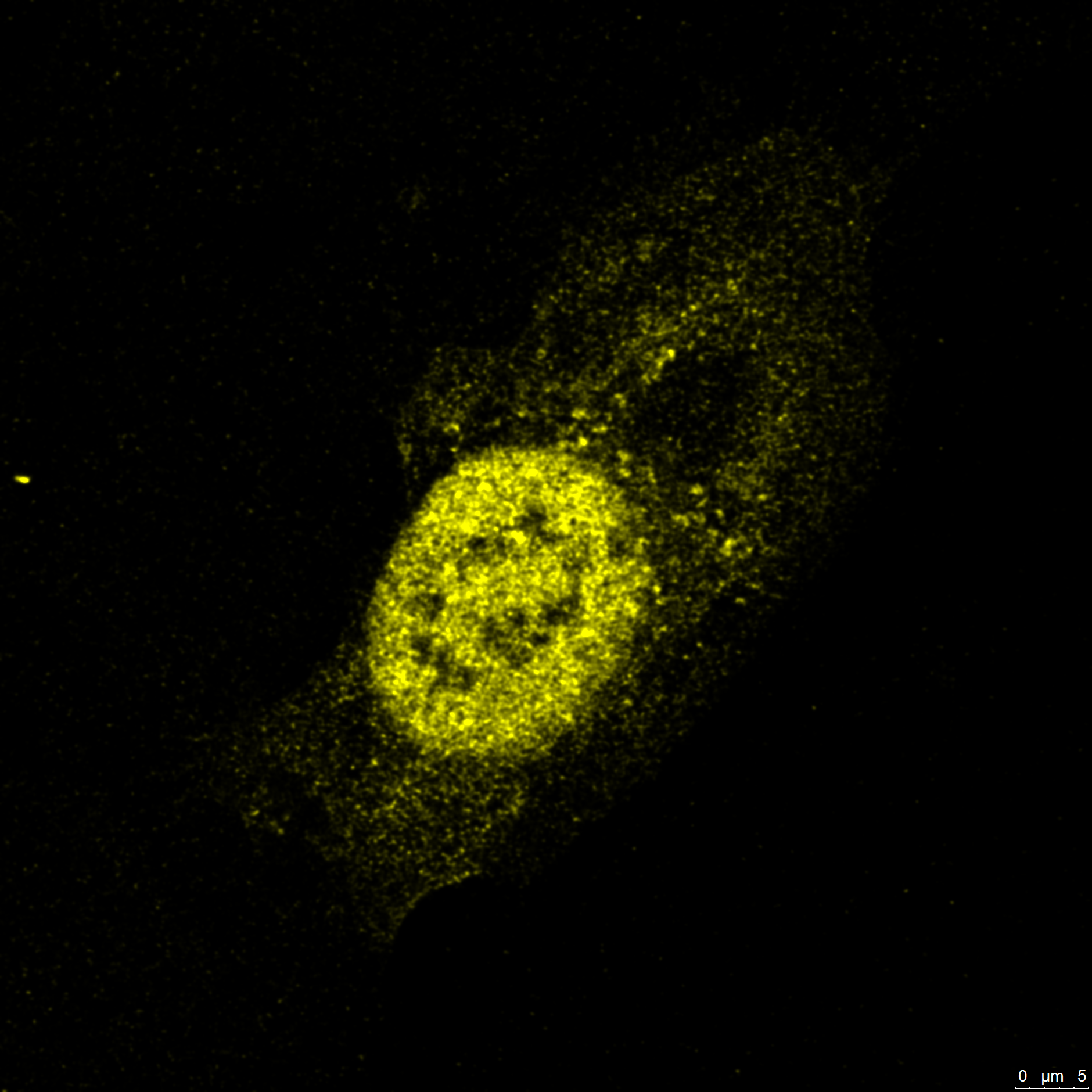

Supplement: Supplementary file 19 — Figure EV6 Source Data [file 44318_2025_654_MOESM19_ESM.zip › EV Figure 6/EV6F/EV6F-3-BFP-ZRANB1(C443S)-HA-ub.tif]

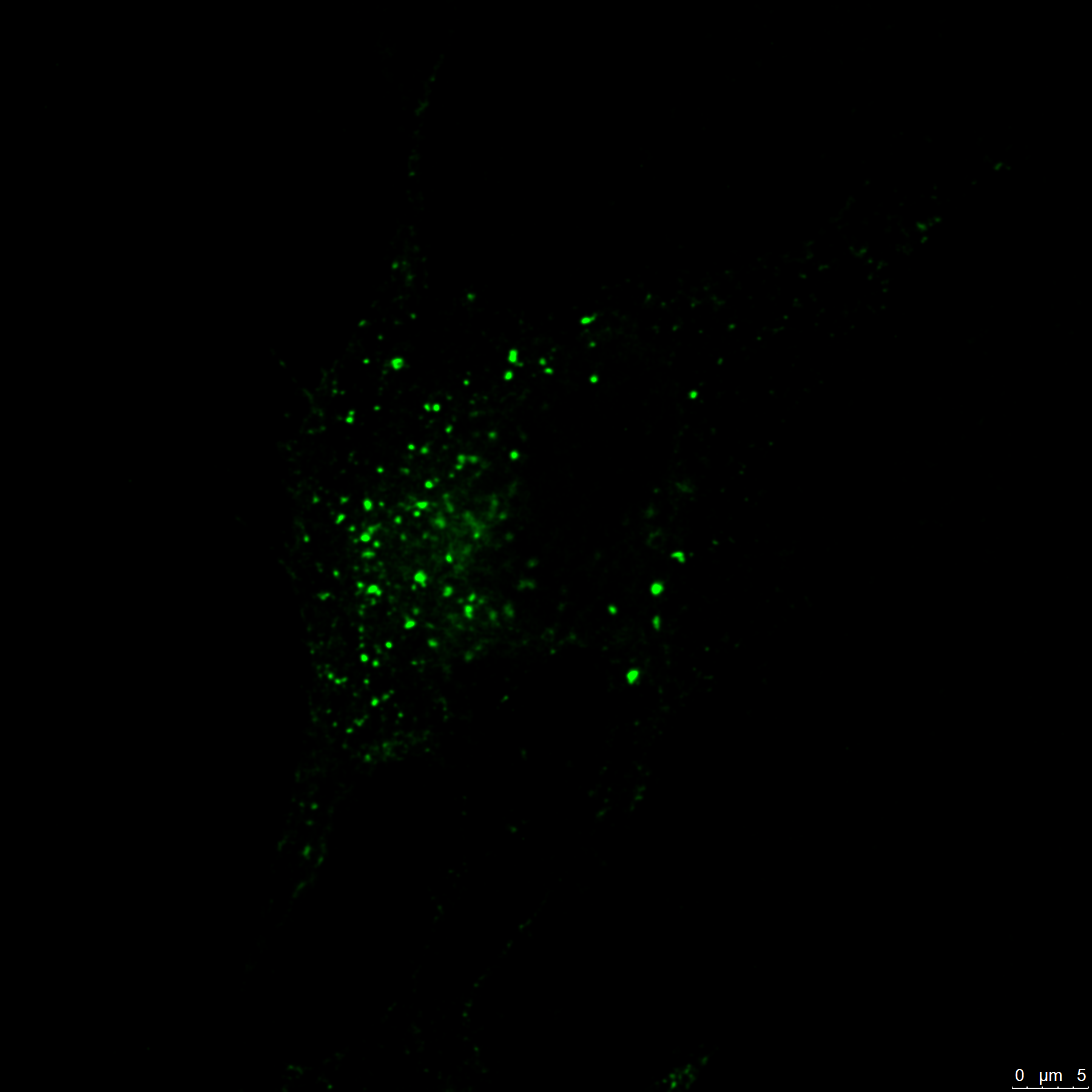

Supplement: Supplementary file 19 — Figure EV6 Source Data [file 44318_2025_654_MOESM19_ESM.zip › EV Figure 6/EV6F/EV6F-2-BFP-ZRANB1(WT)-AREL1-EGFP.tif]

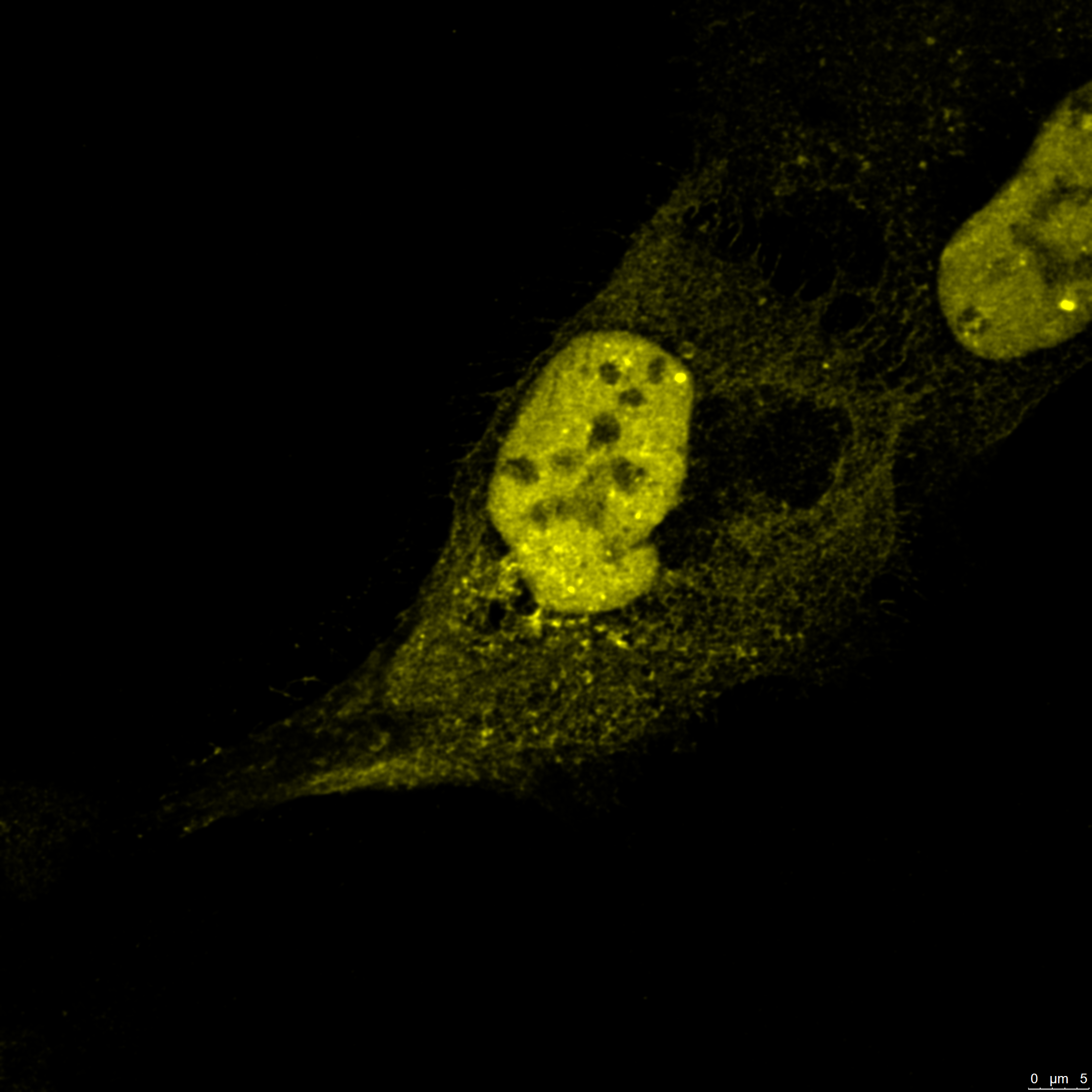

Supplement: Supplementary file 19 — Figure EV6 Source Data [file 44318_2025_654_MOESM19_ESM.zip › EV Figure 6/EV6F/EV6F-1-BFP-HA-ub.tif]

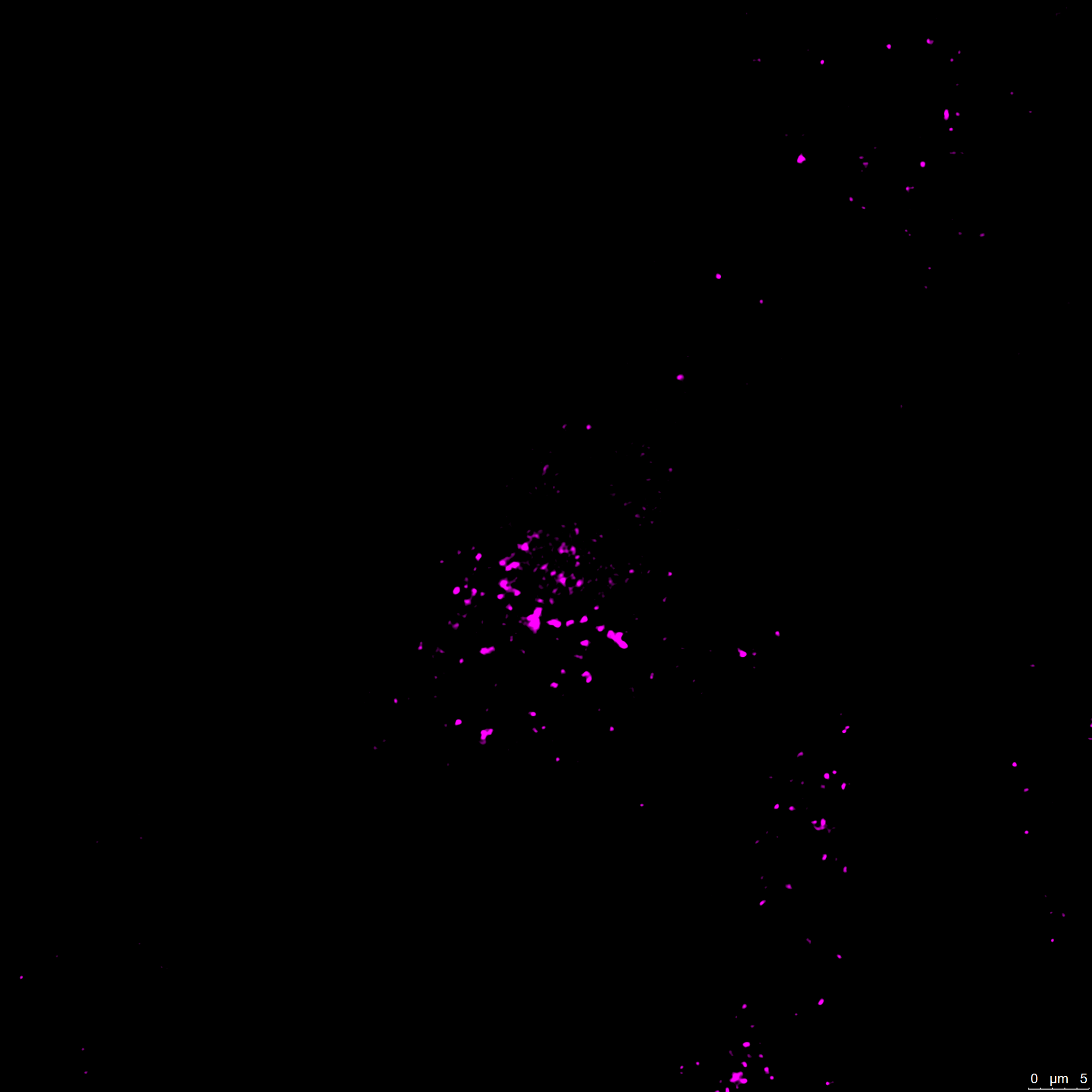

Supplement: Supplementary file 19 — Figure EV6 Source Data [file 44318_2025_654_MOESM19_ESM.zip › EV Figure 6/EV6F/EV6F-1-BFP-LAMP1.tif]

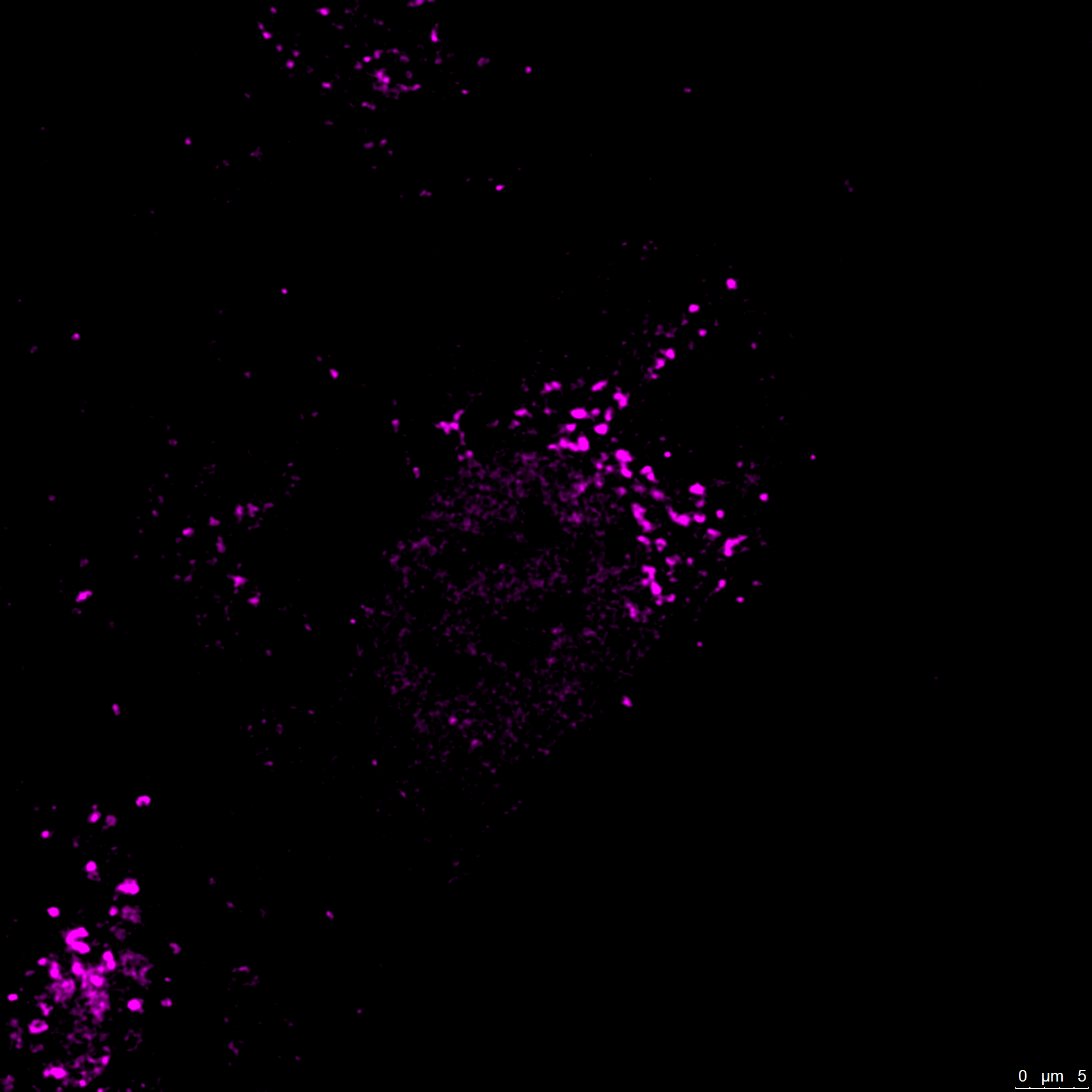

Supplement: Supplementary file 19 — Figure EV6 Source Data [file 44318_2025_654_MOESM19_ESM.zip › EV Figure 6/EV6F/EV6F-3-BFP-ZRANB1(C443S)-LAMP1.tif]

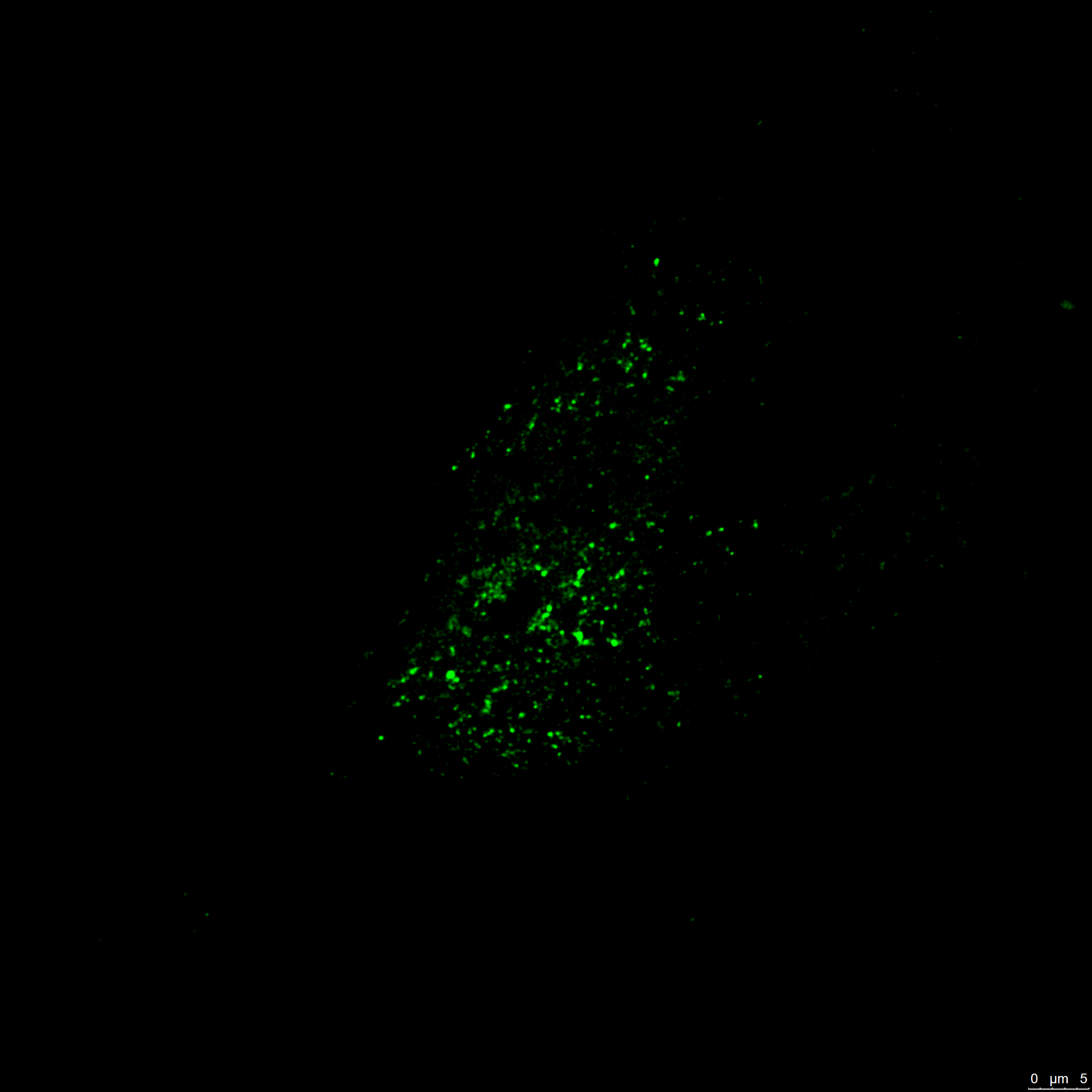

Supplement: Supplementary file 19 — Figure EV6 Source Data [file 44318_2025_654_MOESM19_ESM.zip › EV Figure 6/EV6F/EV6F-1-BFP-AREL1-EGFP.tif]

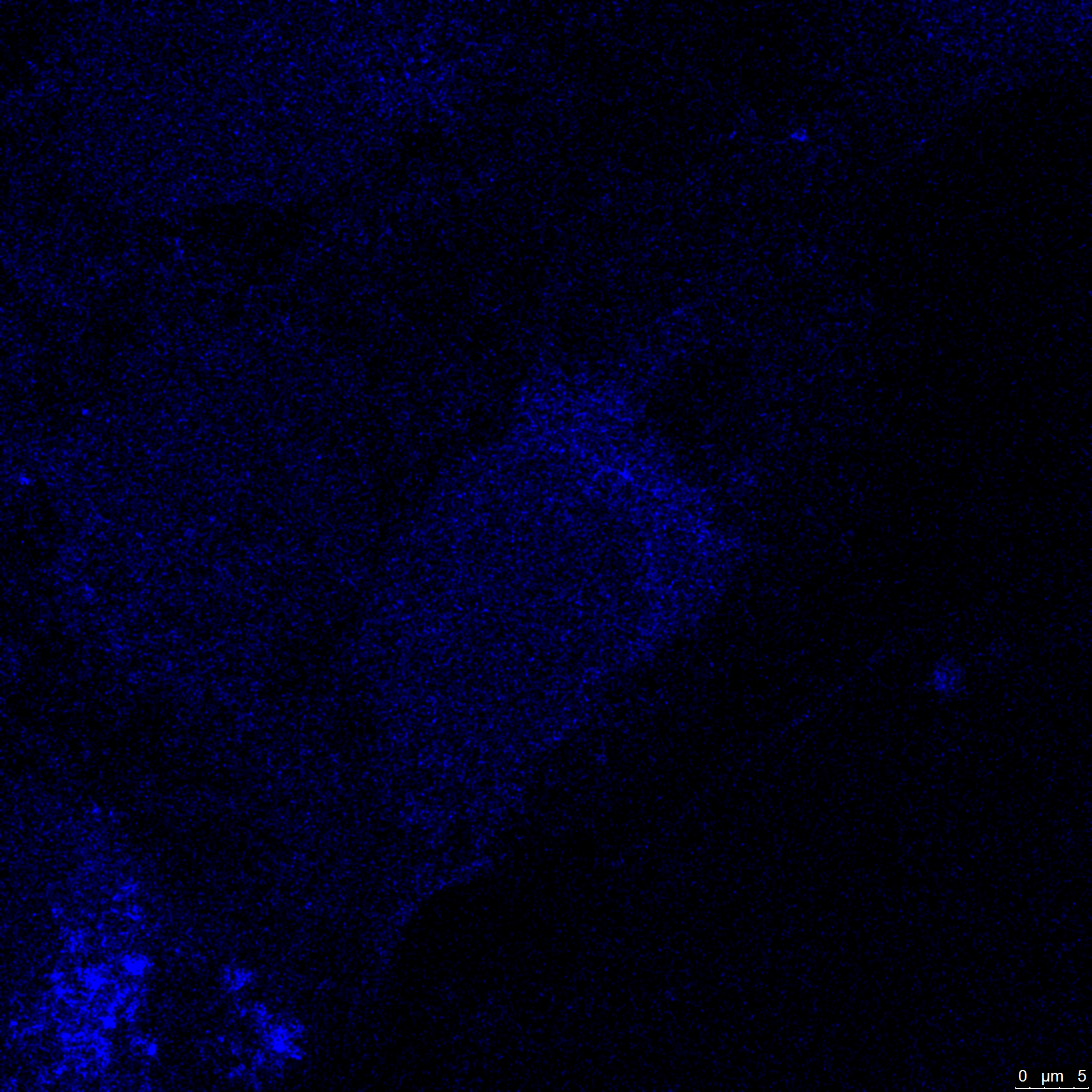

Supplement: Supplementary file 19 — Figure EV6 Source Data [file 44318_2025_654_MOESM19_ESM.zip › EV Figure 6/EV6F/EV6F-3-BFP-ZRANB1(C443S).tif]

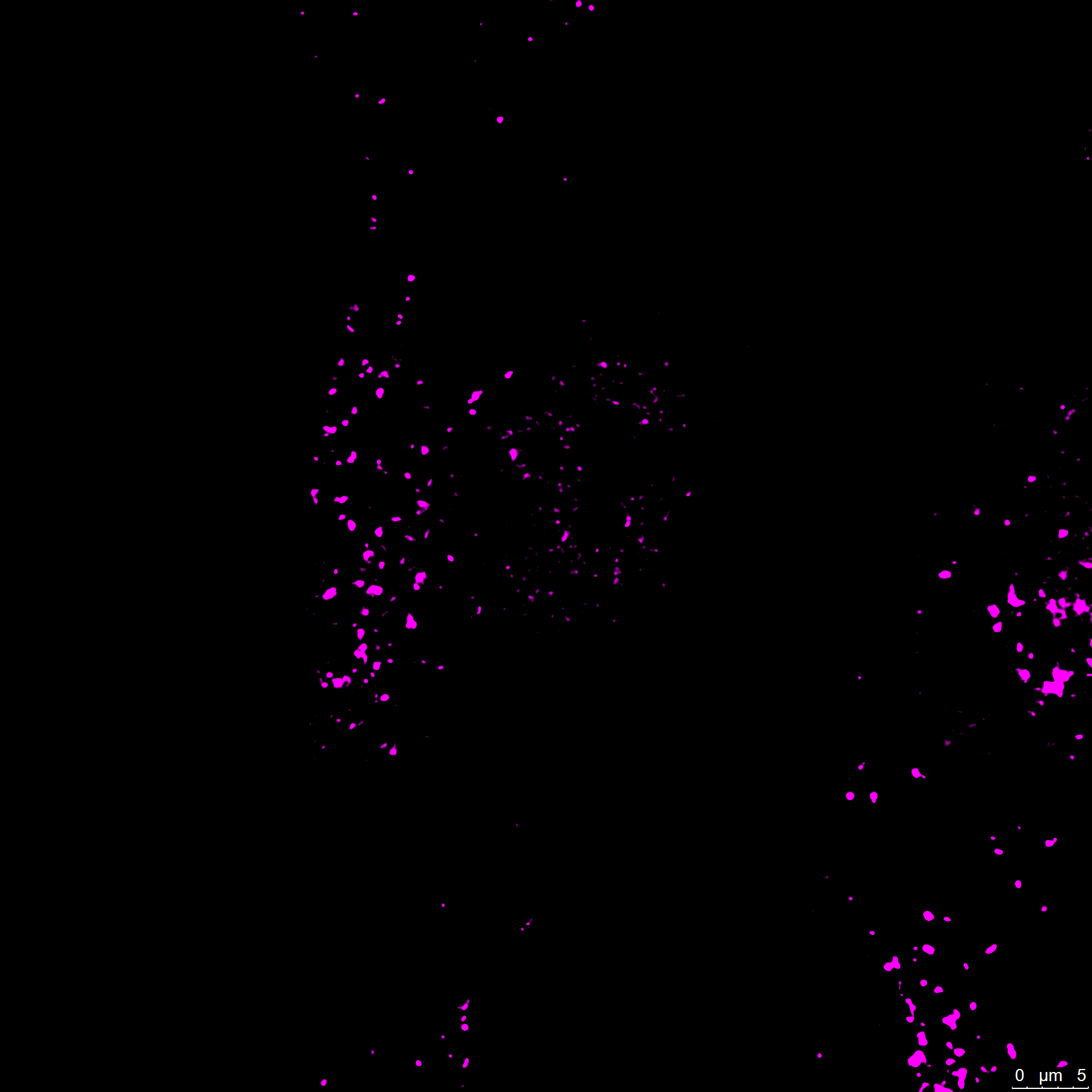

Supplement: Supplementary file 19 — Figure EV6 Source Data [file 44318_2025_654_MOESM19_ESM.zip › EV Figure 6/EV6F/EV6F-2-BFP-ZRANB1(WT)-LAMP1.tif]

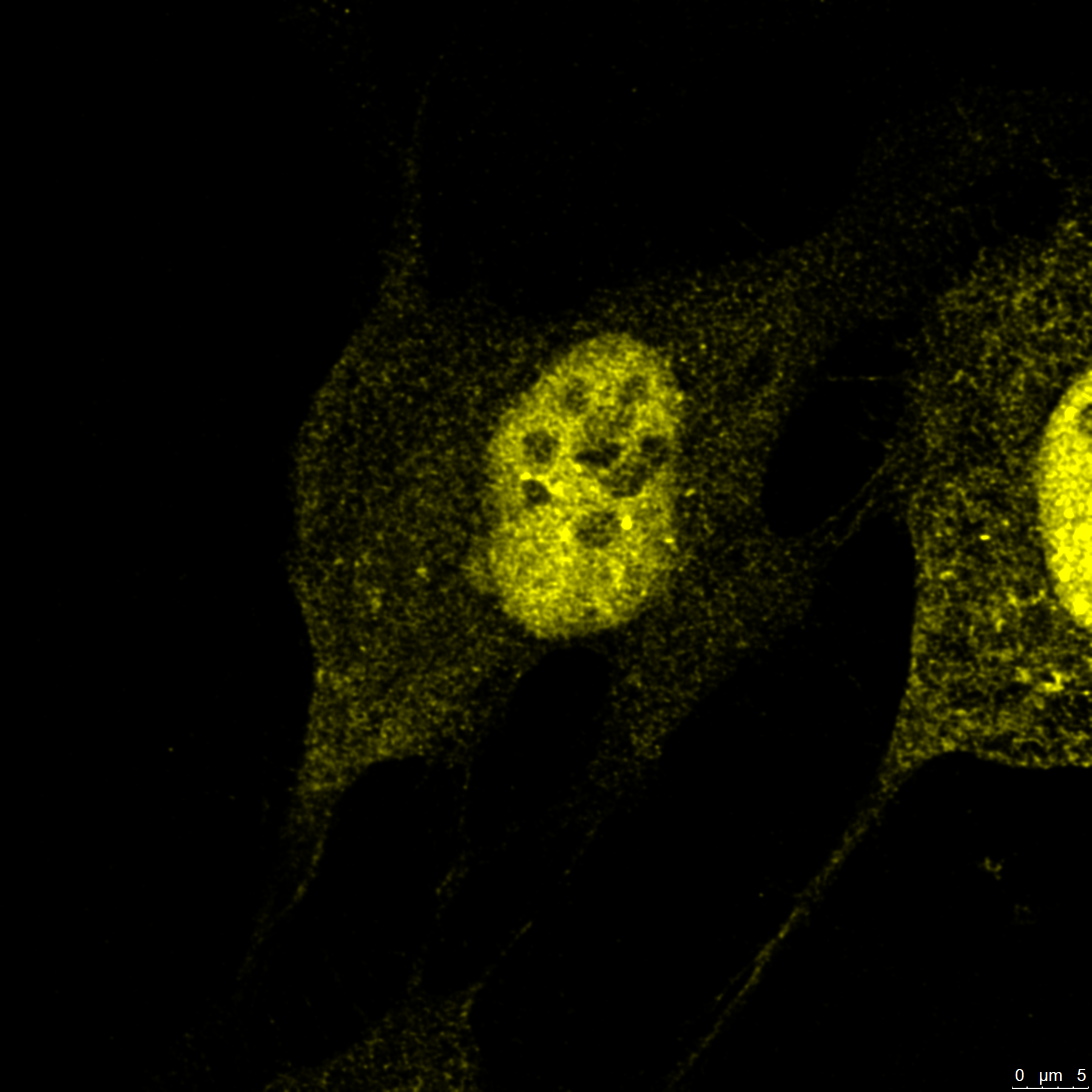

Supplement: Supplementary file 19 — Figure EV6 Source Data [file 44318_2025_654_MOESM19_ESM.zip › EV Figure 6/EV6F/EV6F-2-BFP-ZRANB1(WT)-HA-ub.tif]

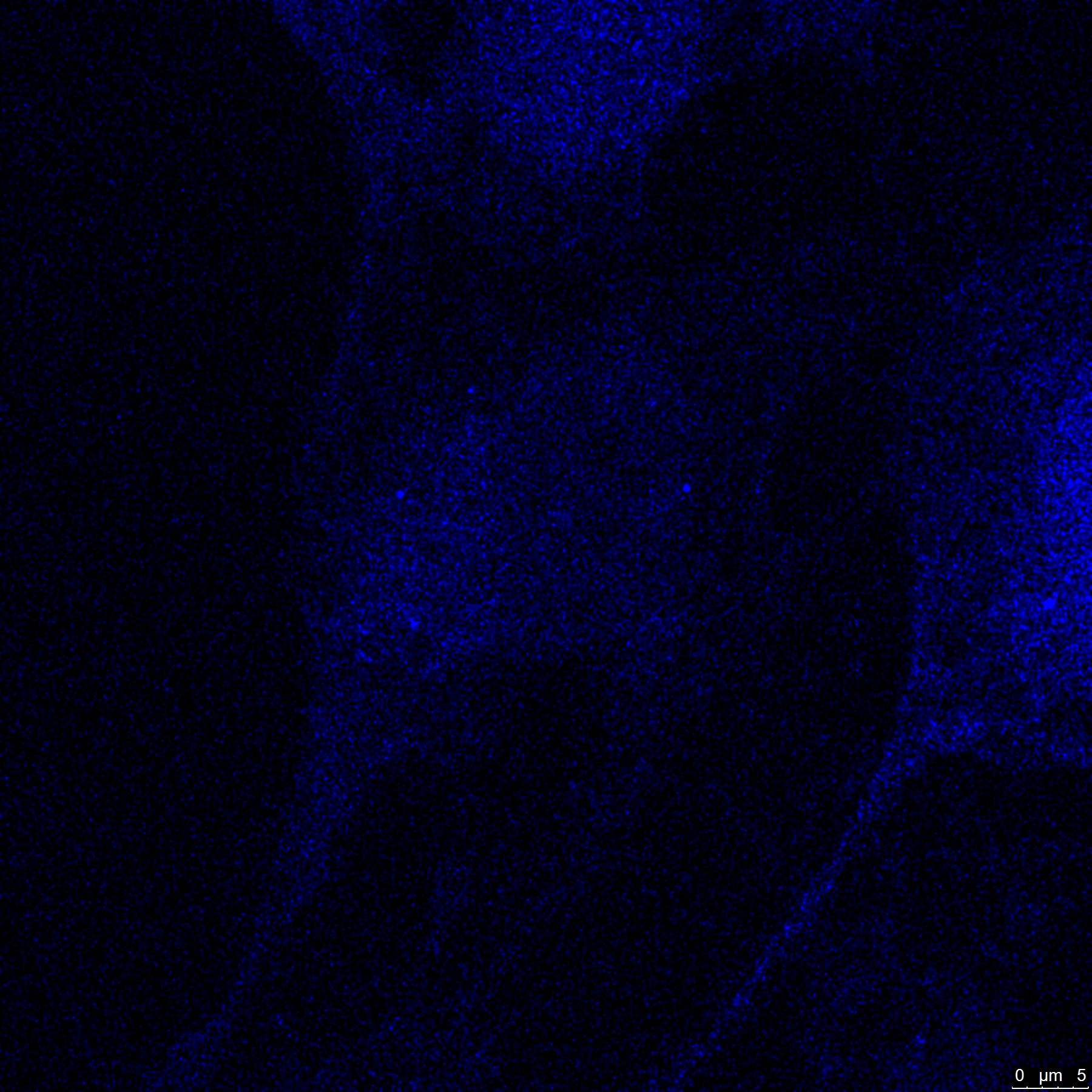

Supplement: Supplementary file 19 — Figure EV6 Source Data [file 44318_2025_654_MOESM19_ESM.zip › EV Figure 6/EV6F/EV6F-2-BFP-ZRANB1(WT).tif]

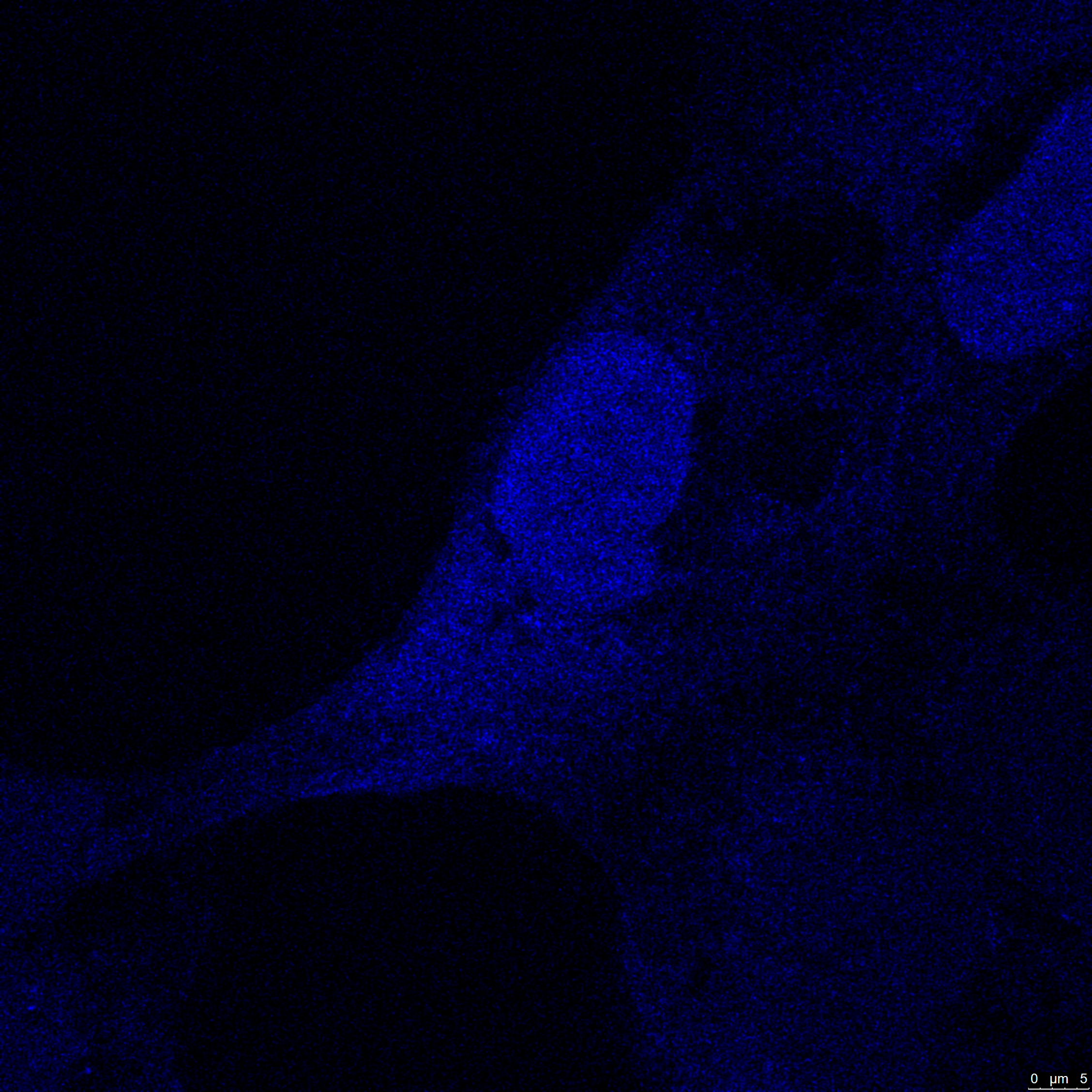

Supplement: Supplementary file 19 — Figure EV6 Source Data [file 44318_2025_654_MOESM19_ESM.zip › EV Figure 6/EV6F/EV6F-1-BFP.tif]

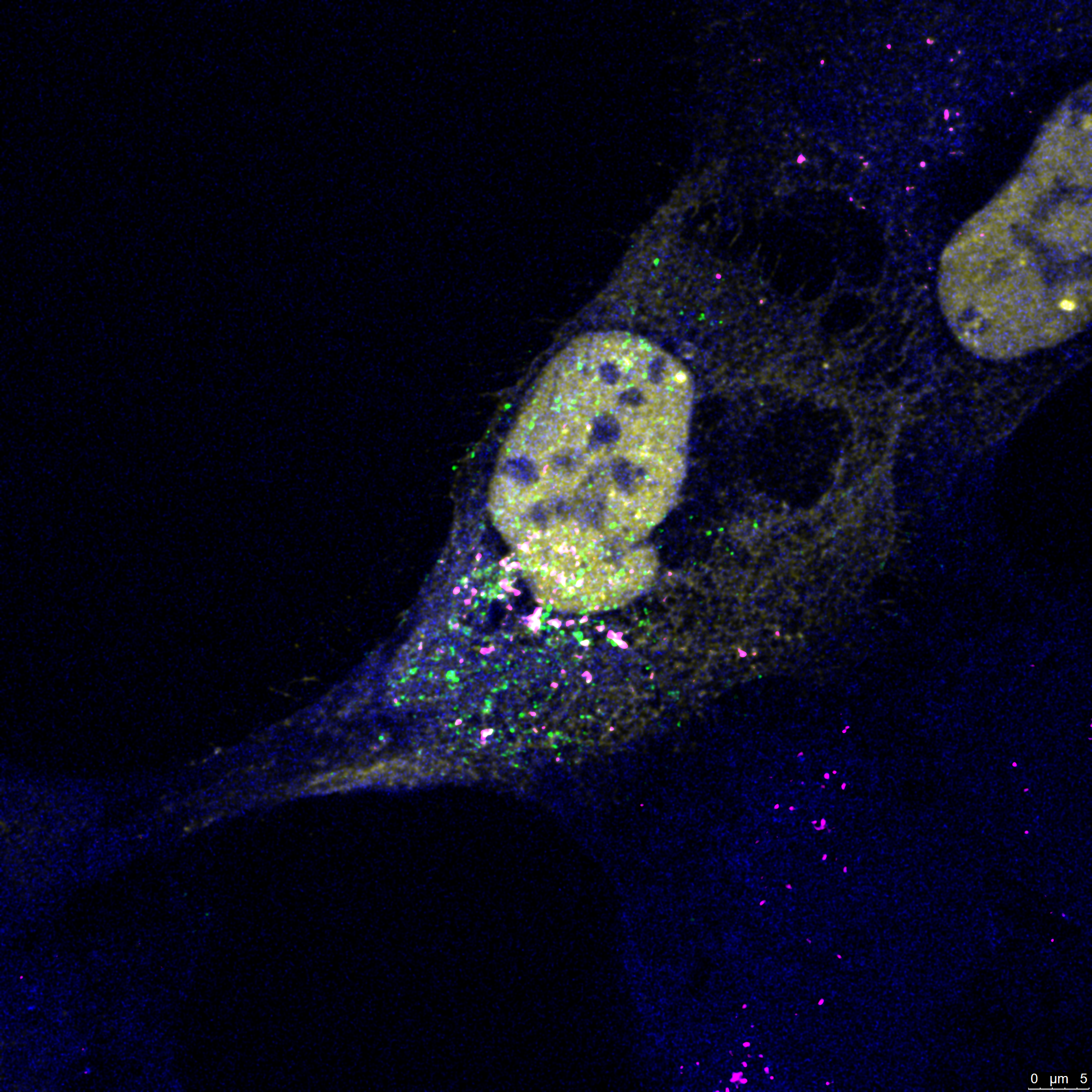

Supplement: Supplementary file 19 — Figure EV6 Source Data [file 44318_2025_654_MOESM19_ESM.zip › EV Figure 6/EV6F/EV6F-1-BFP-merge.tif]

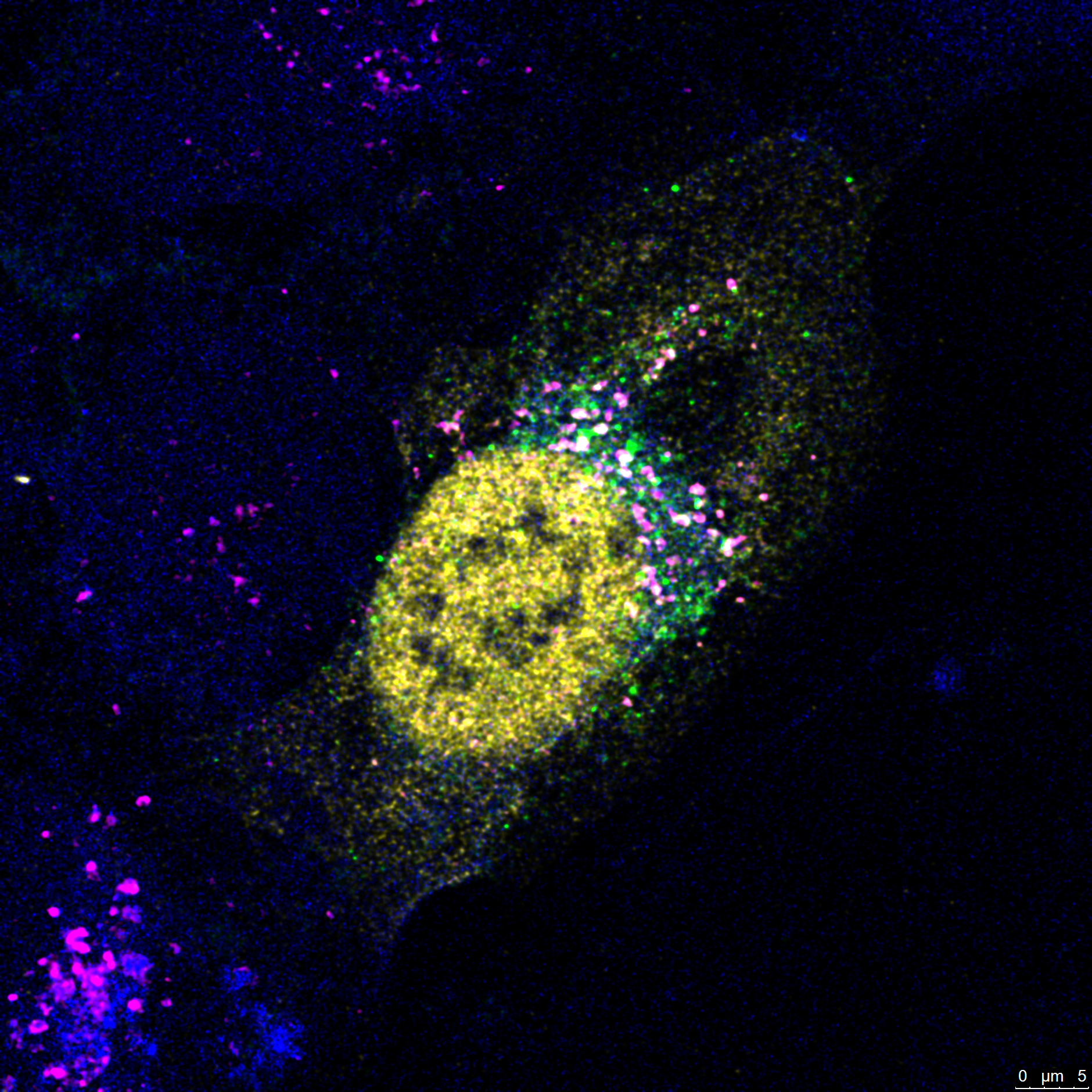

Supplement: Supplementary file 19 — Figure EV6 Source Data [file 44318_2025_654_MOESM19_ESM.zip › EV Figure 6/EV6F/EV6F-3-BFP-ZRANB1(C443S)-merge.tif]

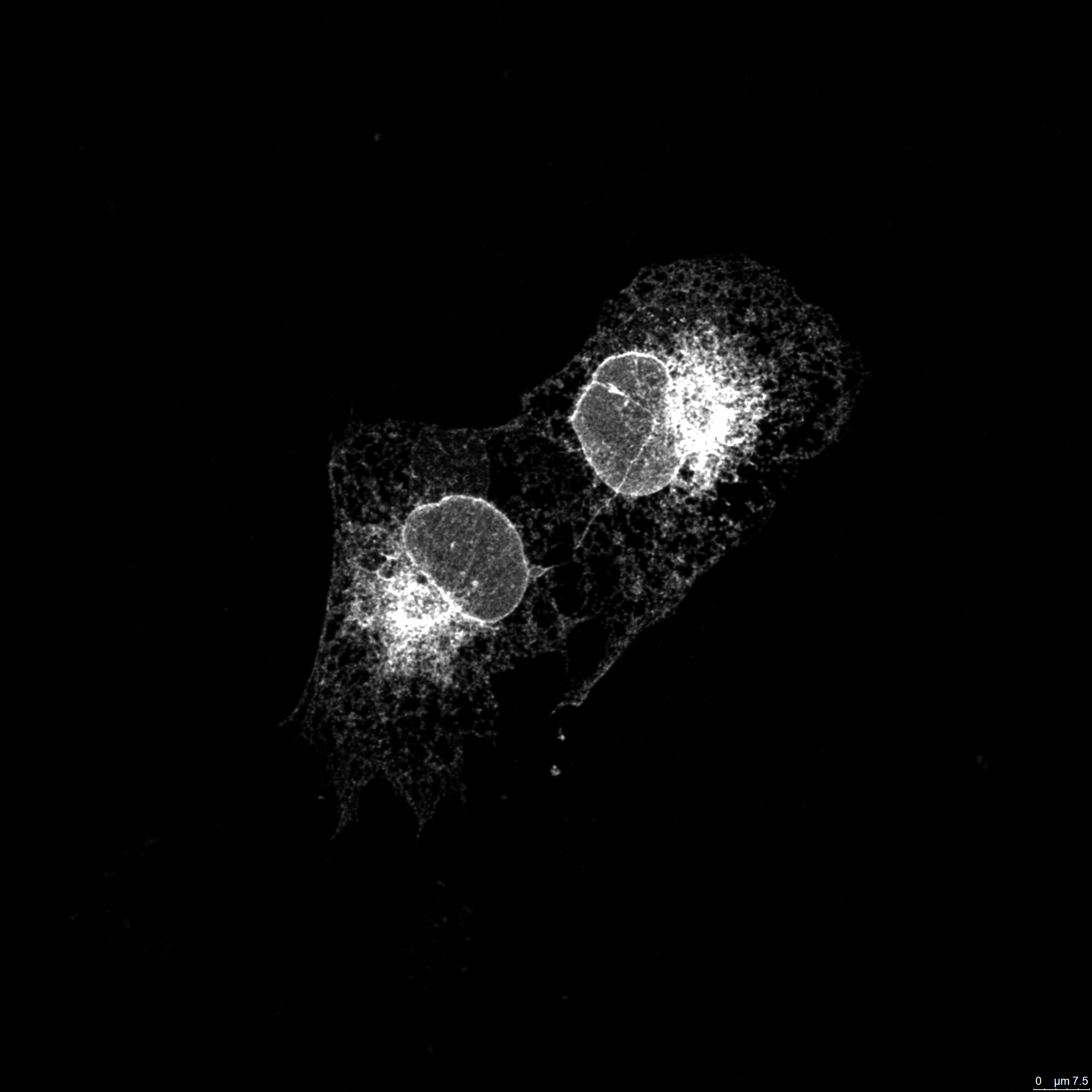

Supplement: Supplementary file 20 — Figure EV7 Source Data [file 44318_2025_654_MOESM20_ESM.zip › EV Figure 7/EV7H/EV7H-1-WT-UBAC2-mCherry.tif]

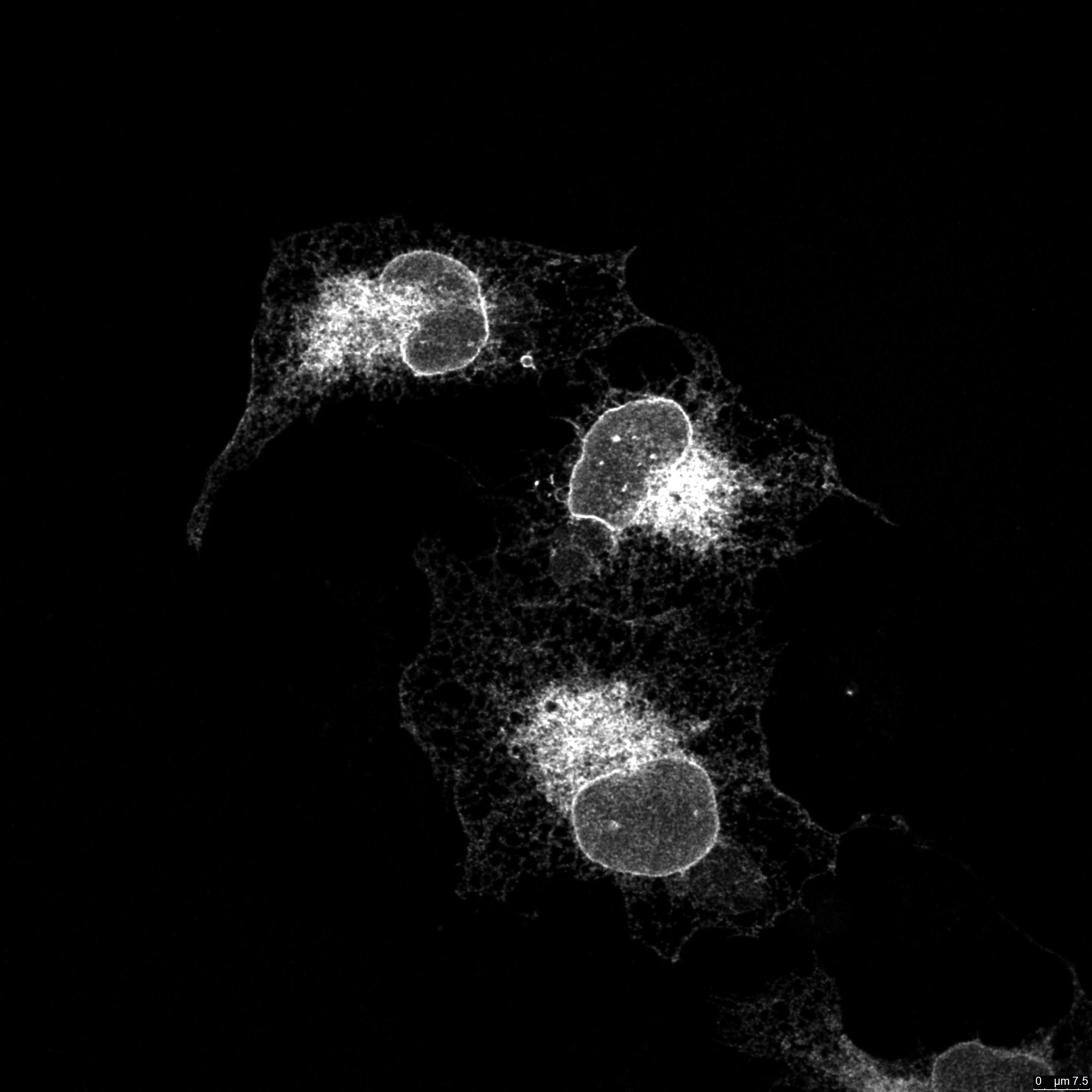

Supplement: Supplementary file 20 — Figure EV7 Source Data [file 44318_2025_654_MOESM20_ESM.zip › EV Figure 7/EV7H/EV7H-2-KO-UBAC2-mCherry.tif]

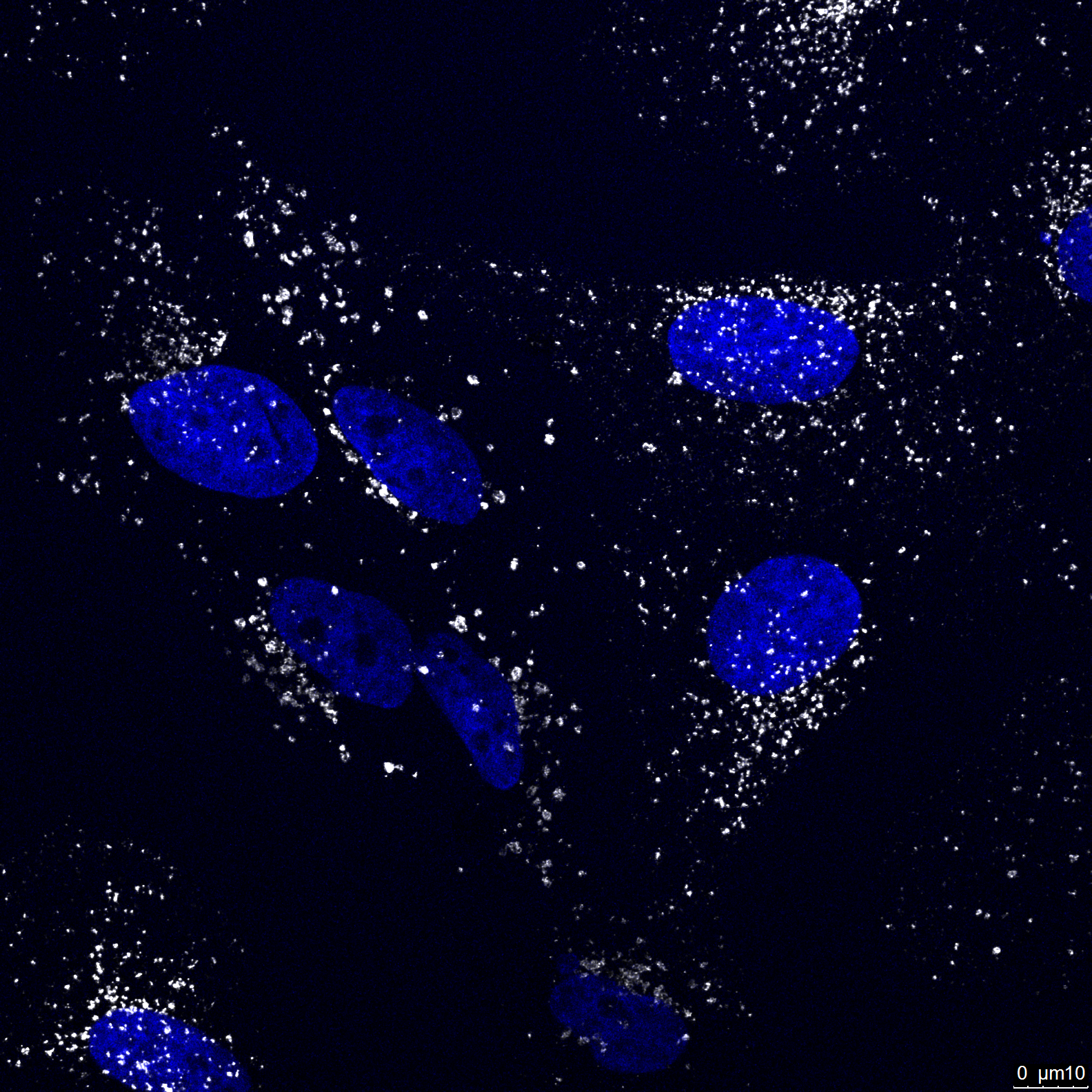

Supplement: Supplementary file 20 — Figure EV7 Source Data [file 44318_2025_654_MOESM20_ESM.zip › EV Figure 7/EV7F/EV7F-1-shNC-PLA.tif]

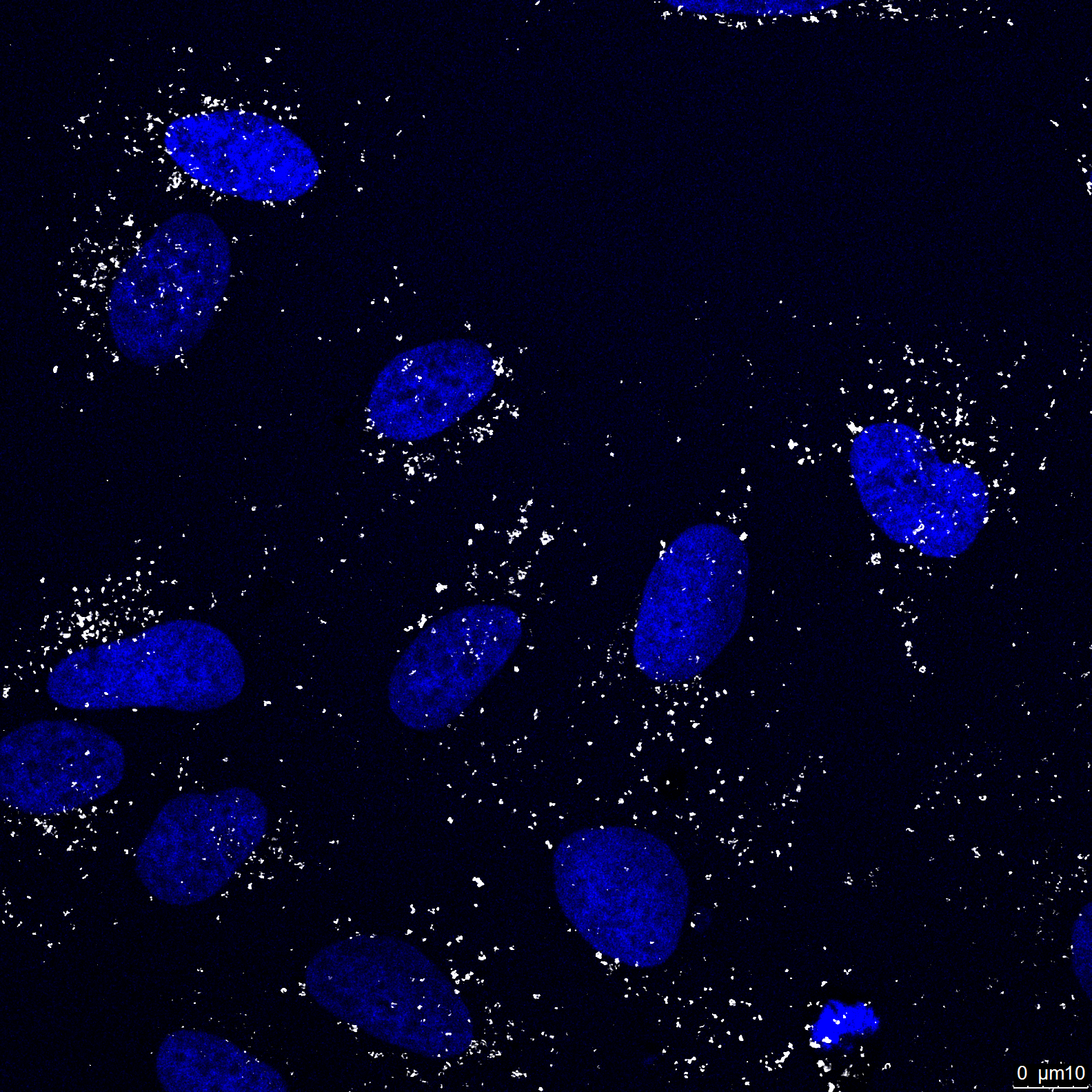

Supplement: Supplementary file 20 — Figure EV7 Source Data [file 44318_2025_654_MOESM20_ESM.zip › EV Figure 7/EV7F/EV7F-2-shUBAC2-PLA.tif]

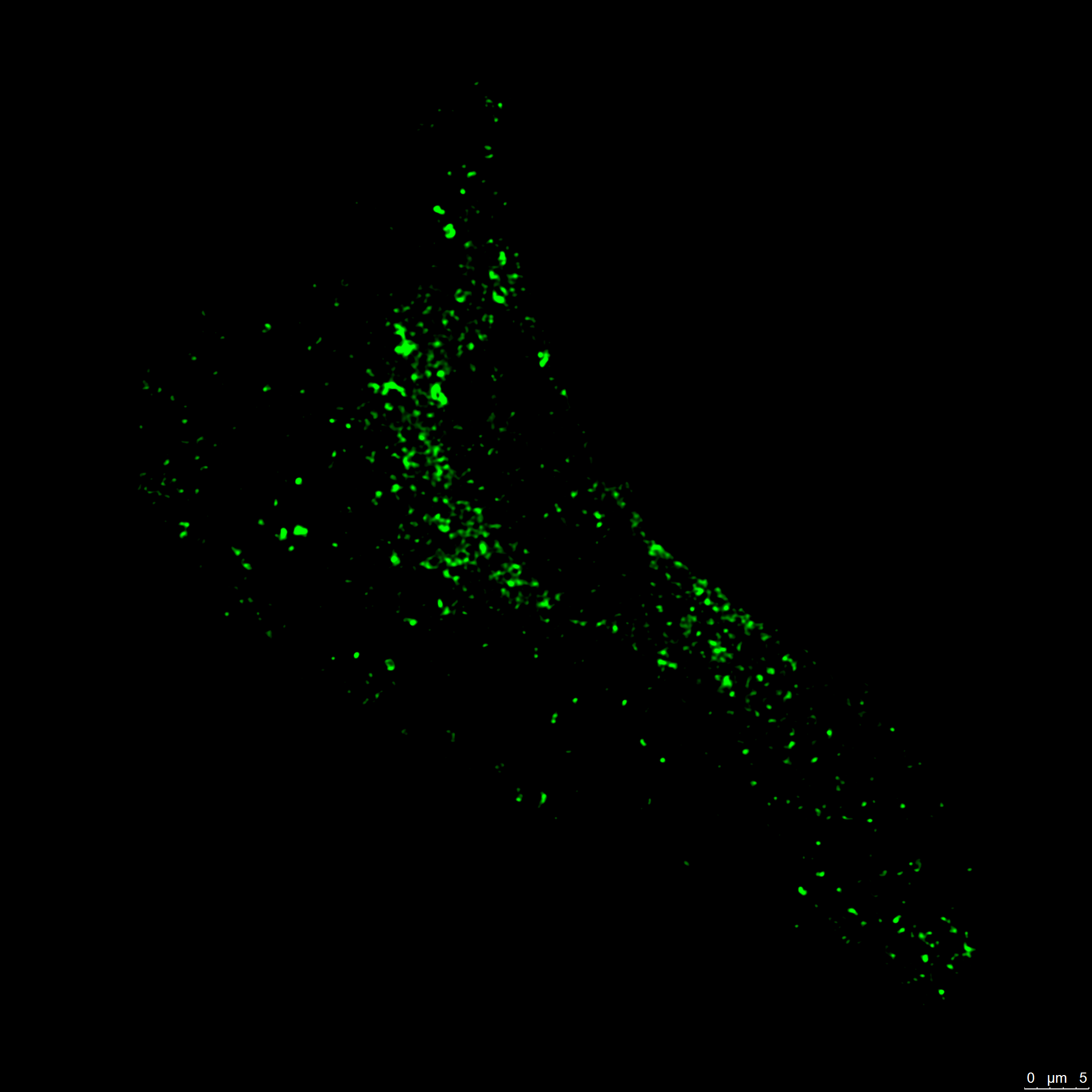

Supplement: Supplementary file 20 — Figure EV7 Source Data [file 44318_2025_654_MOESM20_ESM.zip › EV Figure 7/EV7I/EV7I-2-shUBAC2-AREL1(WT)-EGFP.tif]

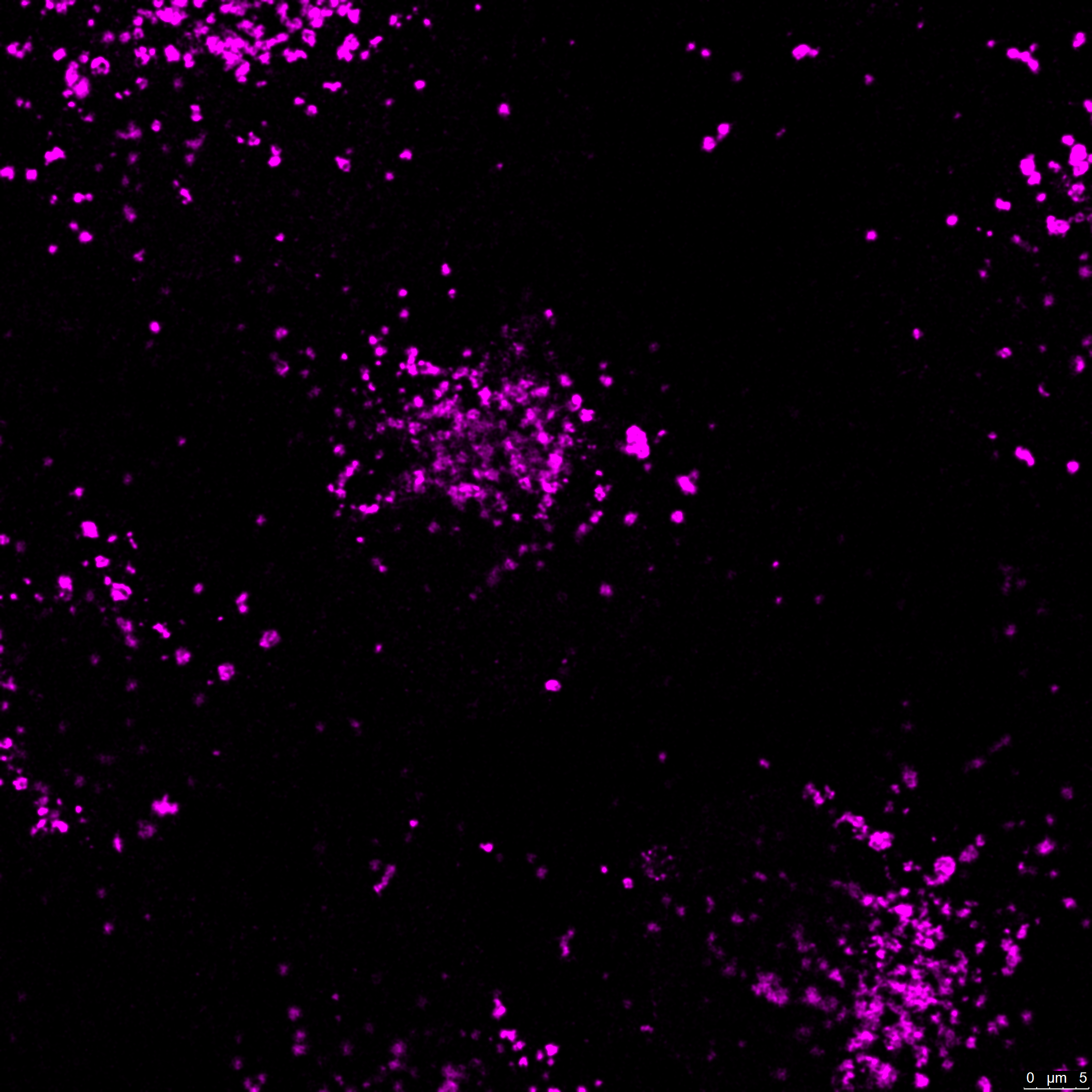

Supplement: Supplementary file 20 — Figure EV7 Source Data [file 44318_2025_654_MOESM20_ESM.zip › EV Figure 7/EV7I/EV7I-1-shNC-AREL1(WT)-EGFP-LAMP1.tif]

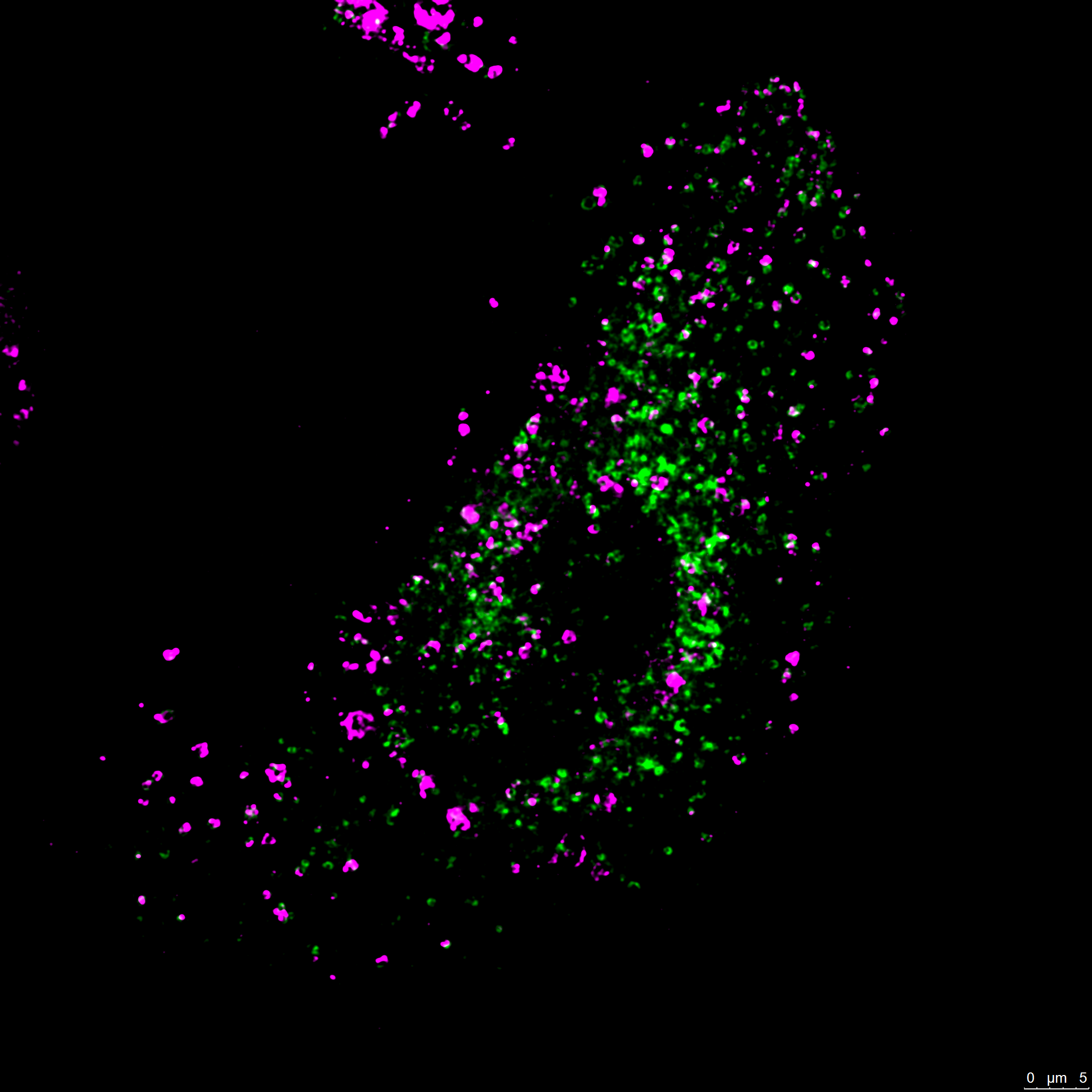

Supplement: Supplementary file 20 — Figure EV7 Source Data [file 44318_2025_654_MOESM20_ESM.zip › EV Figure 7/EV7I/EV7I-3-shUBAC2-AREL1(C790A)-EGFP-merge.tif]

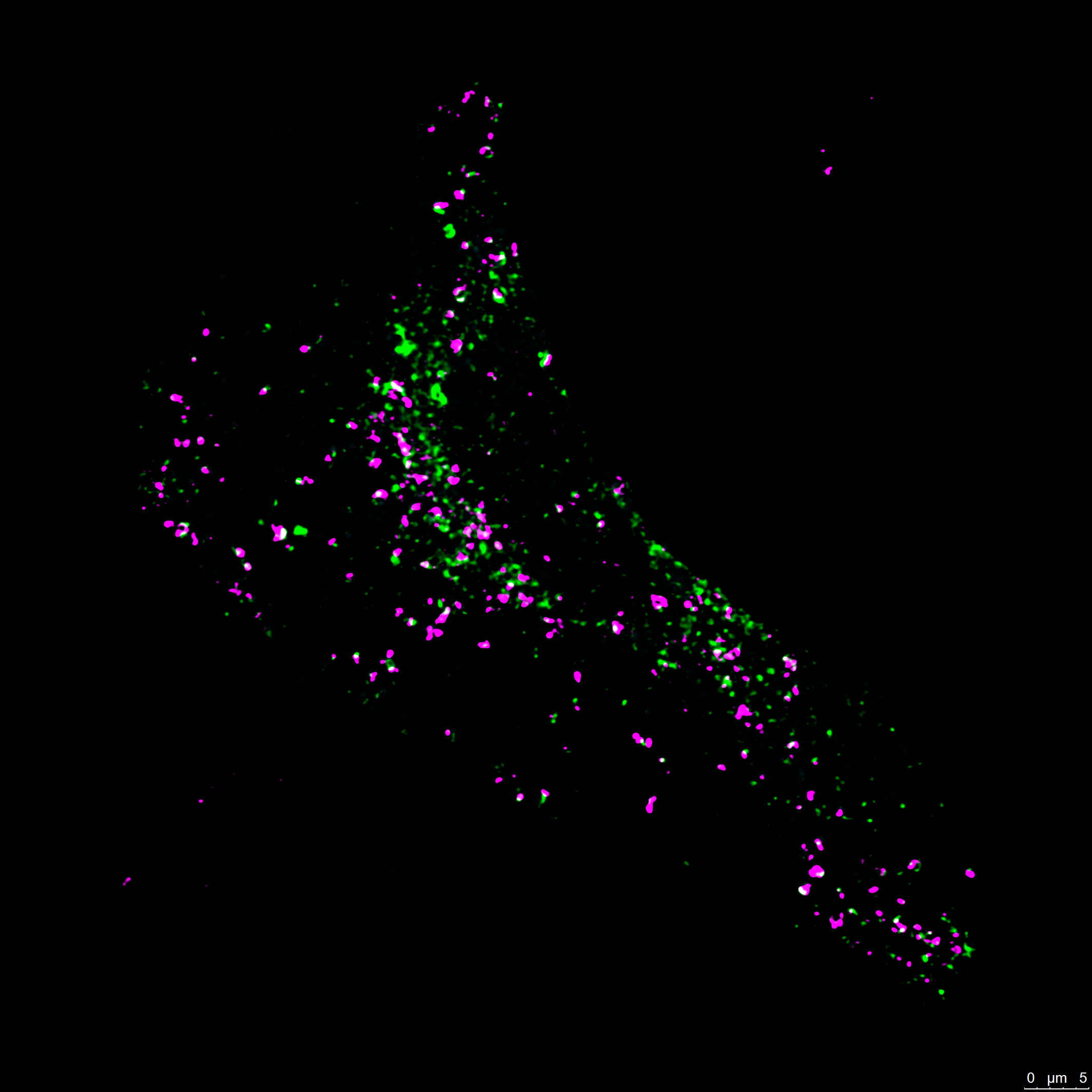

Supplement: Supplementary file 20 — Figure EV7 Source Data [file 44318_2025_654_MOESM20_ESM.zip › EV Figure 7/EV7I/EV7I-2-shUBAC2-AREL1(WT)-EGFP-merge.tif]

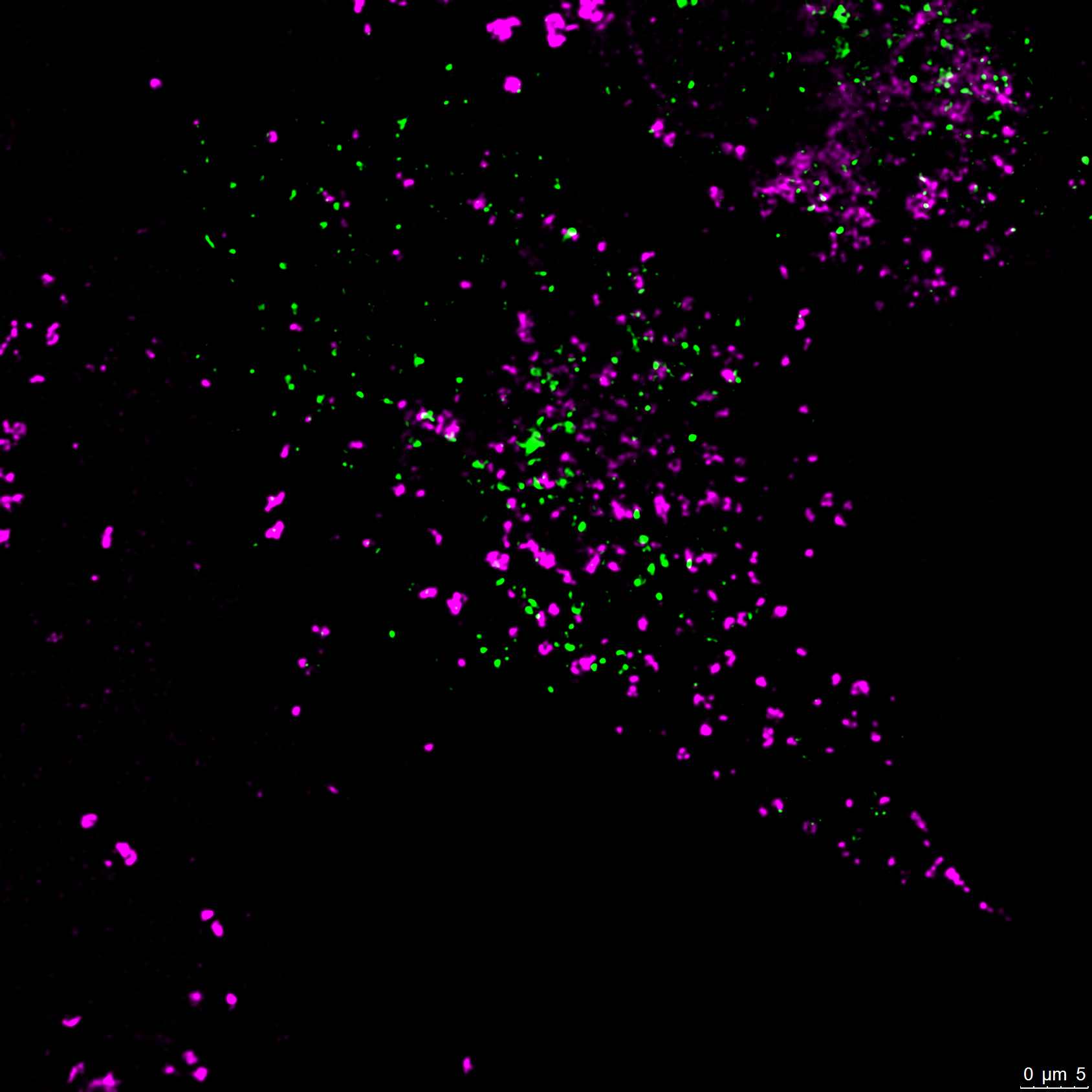

Supplement: Supplementary file 20 — Figure EV7 Source Data [file 44318_2025_654_MOESM20_ESM.zip › EV Figure 7/EV7I/EV7I-4-shUBAC2-AREL1(╬öhinge region)-EGFP-merge.tif]

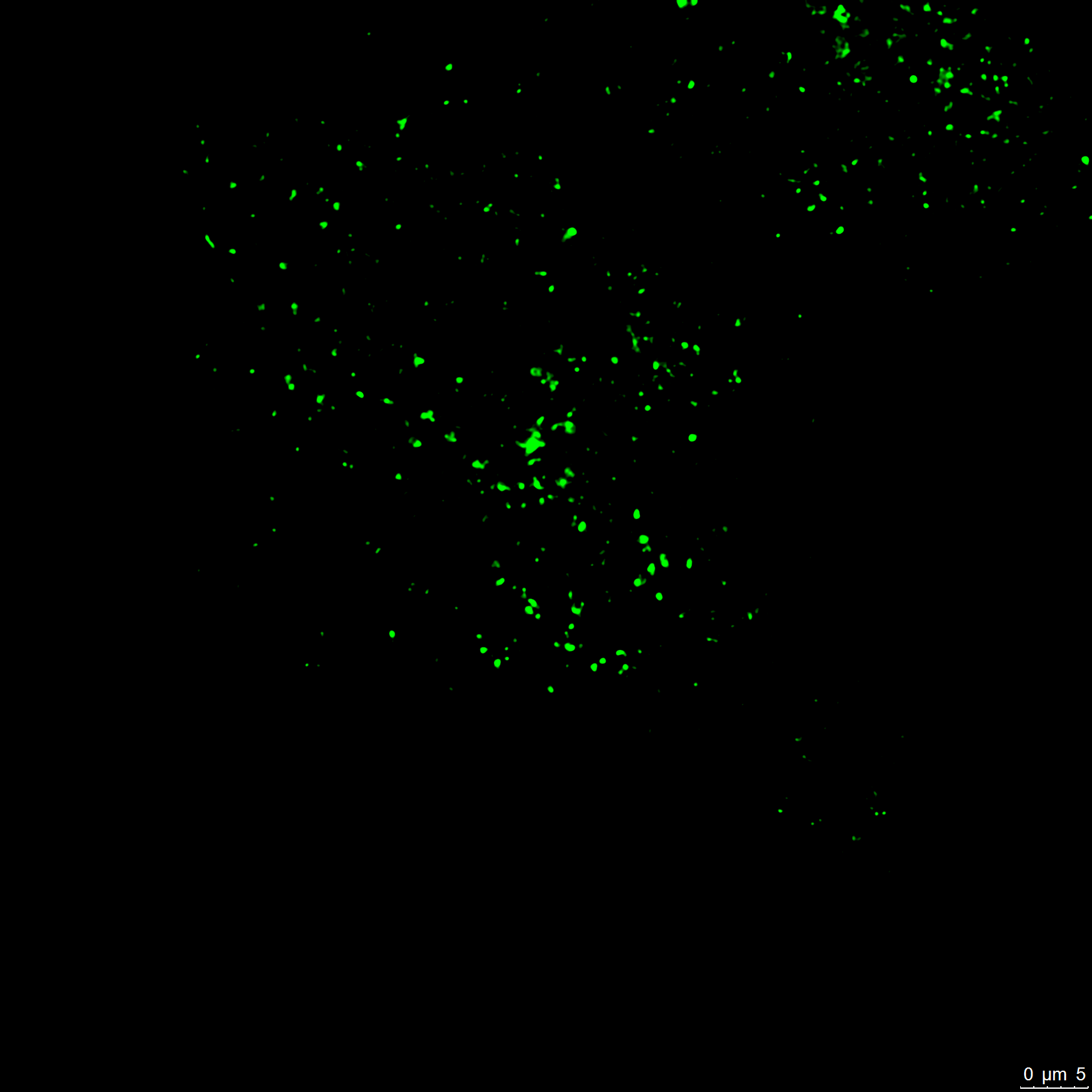

Supplement: Supplementary file 20 — Figure EV7 Source Data [file 44318_2025_654_MOESM20_ESM.zip › EV Figure 7/EV7I/EV7I-4-shUBAC2-AREL1(╬öhinge region)-EGFP.tif]

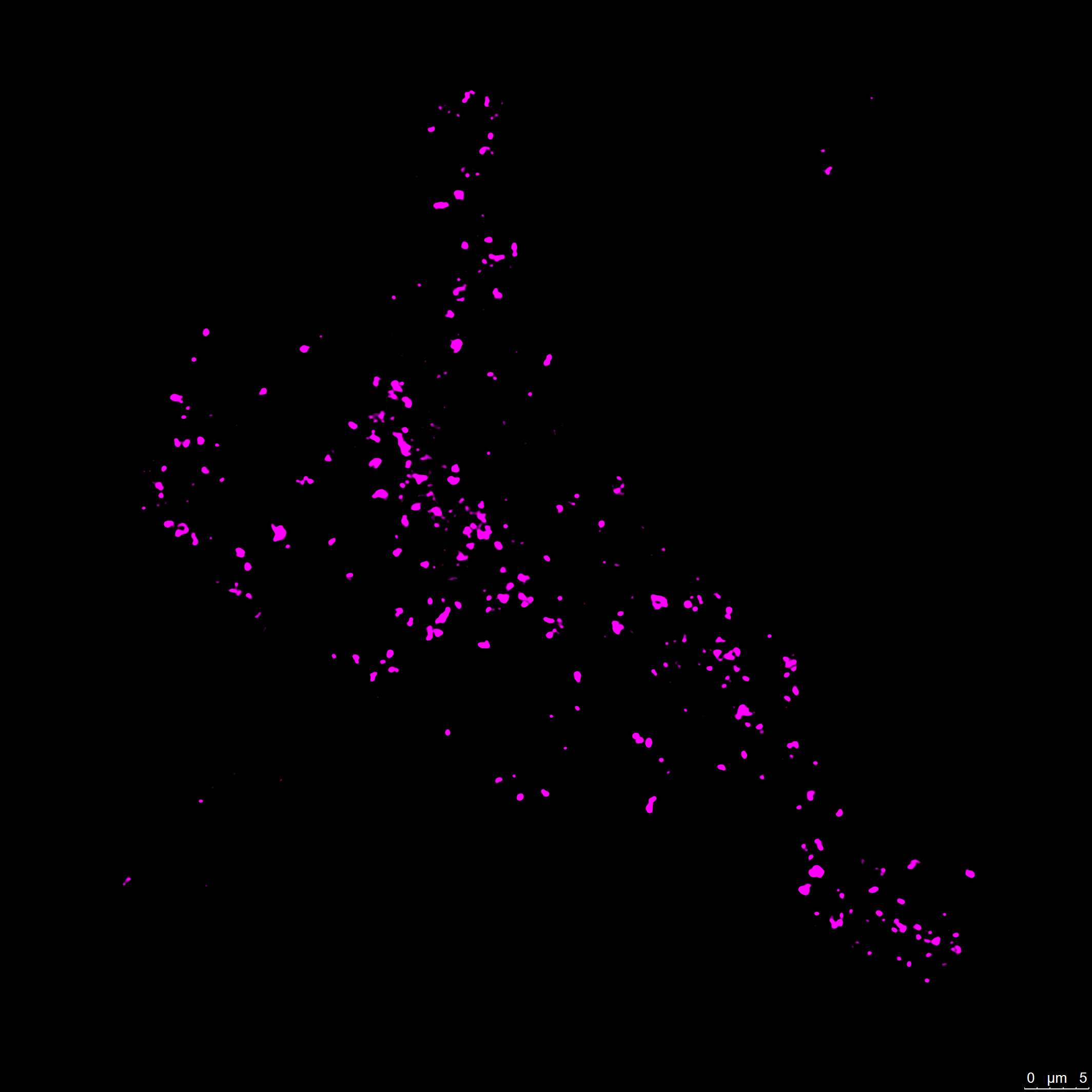

Supplement: Supplementary file 20 — Figure EV7 Source Data [file 44318_2025_654_MOESM20_ESM.zip › EV Figure 7/EV7I/EV7I-2-shUBAC2-AREL1(WT)-EGFP-LAMP1.tif]

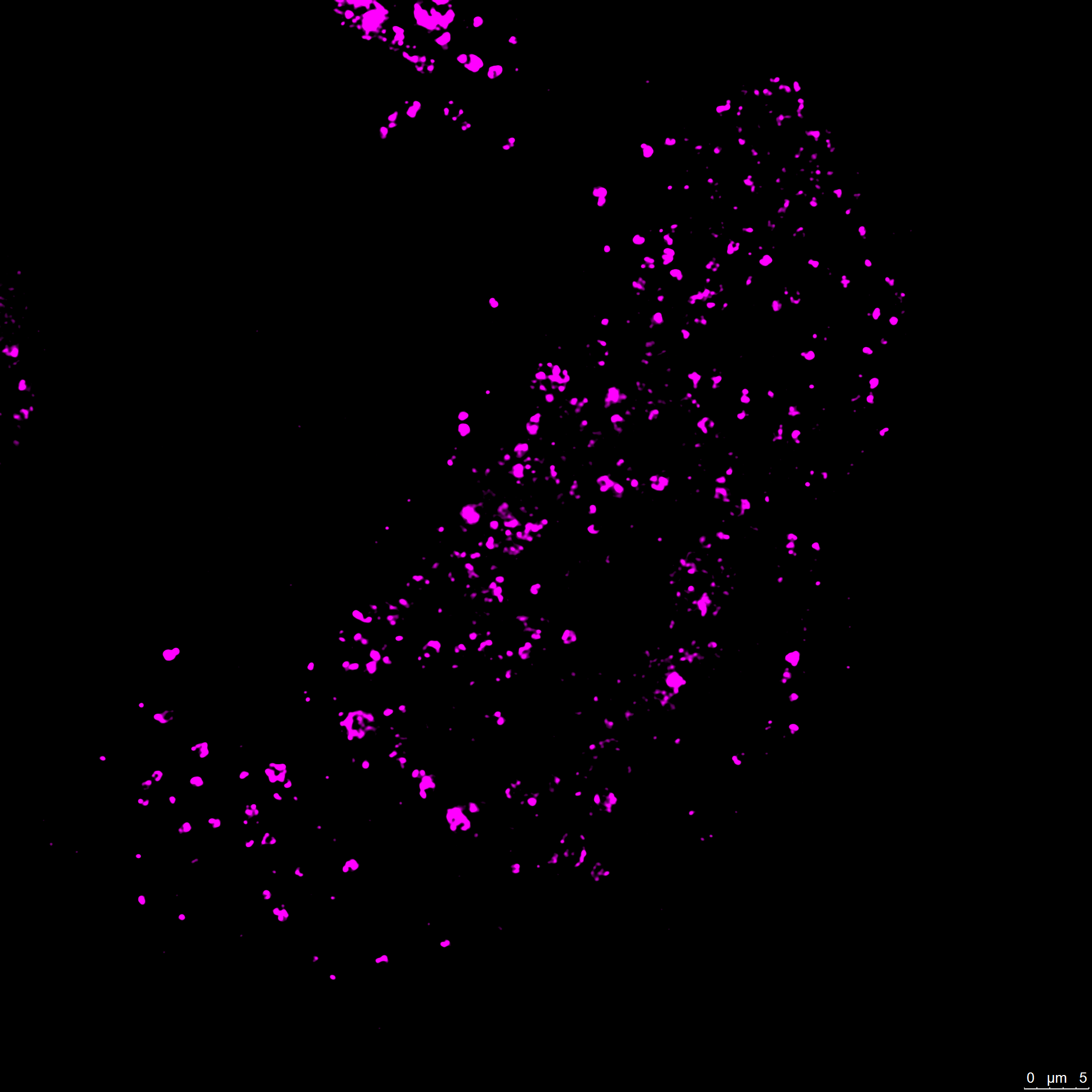

Supplement: Supplementary file 20 — Figure EV7 Source Data [file 44318_2025_654_MOESM20_ESM.zip › EV Figure 7/EV7I/EV7I-3-shUBAC2-AREL1(C790A)-EGFP-LAMP1.tif]

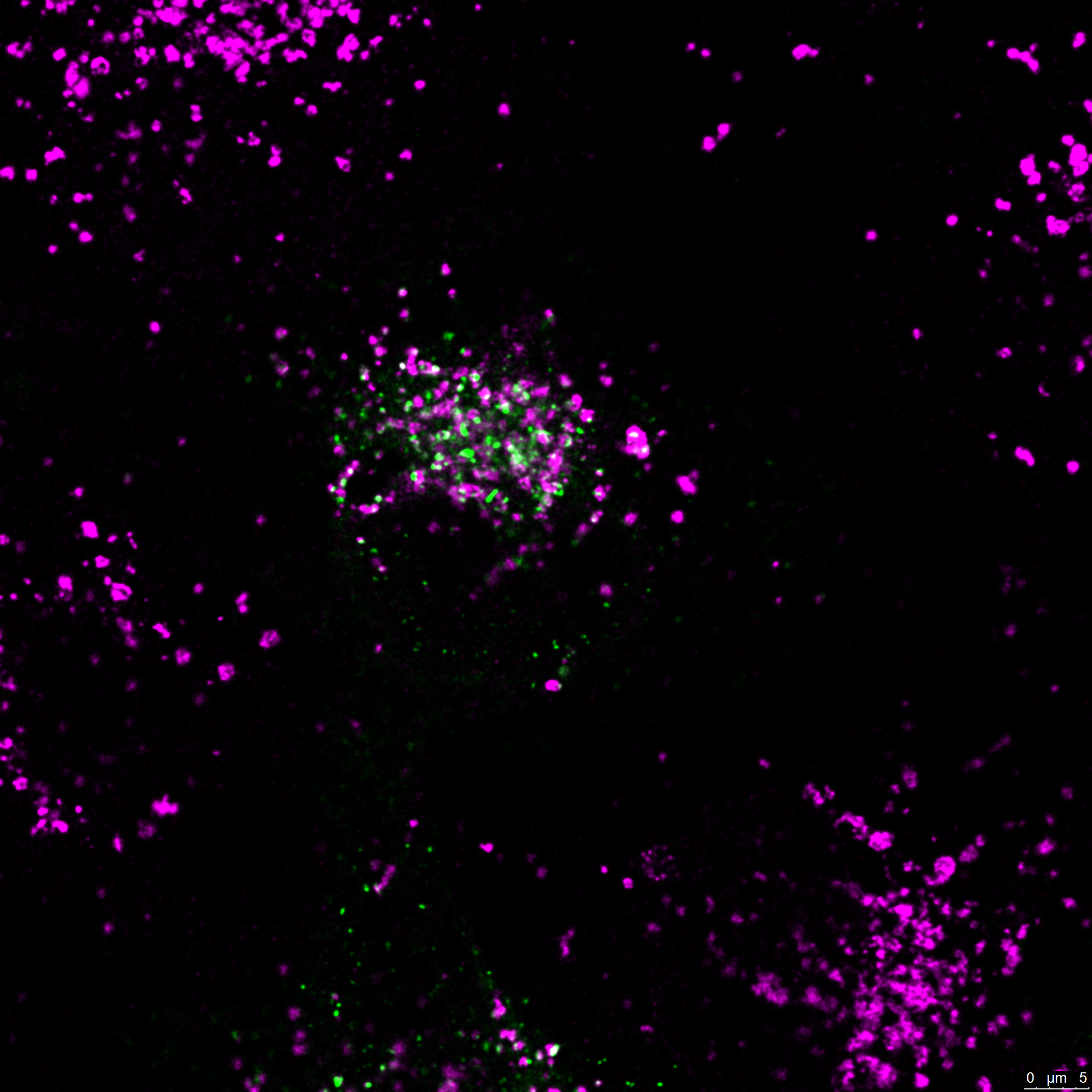

Supplement: Supplementary file 20 — Figure EV7 Source Data [file 44318_2025_654_MOESM20_ESM.zip › EV Figure 7/EV7I/EV7I-1-shNC-AREL1(WT)-EGFP-merge.tif]

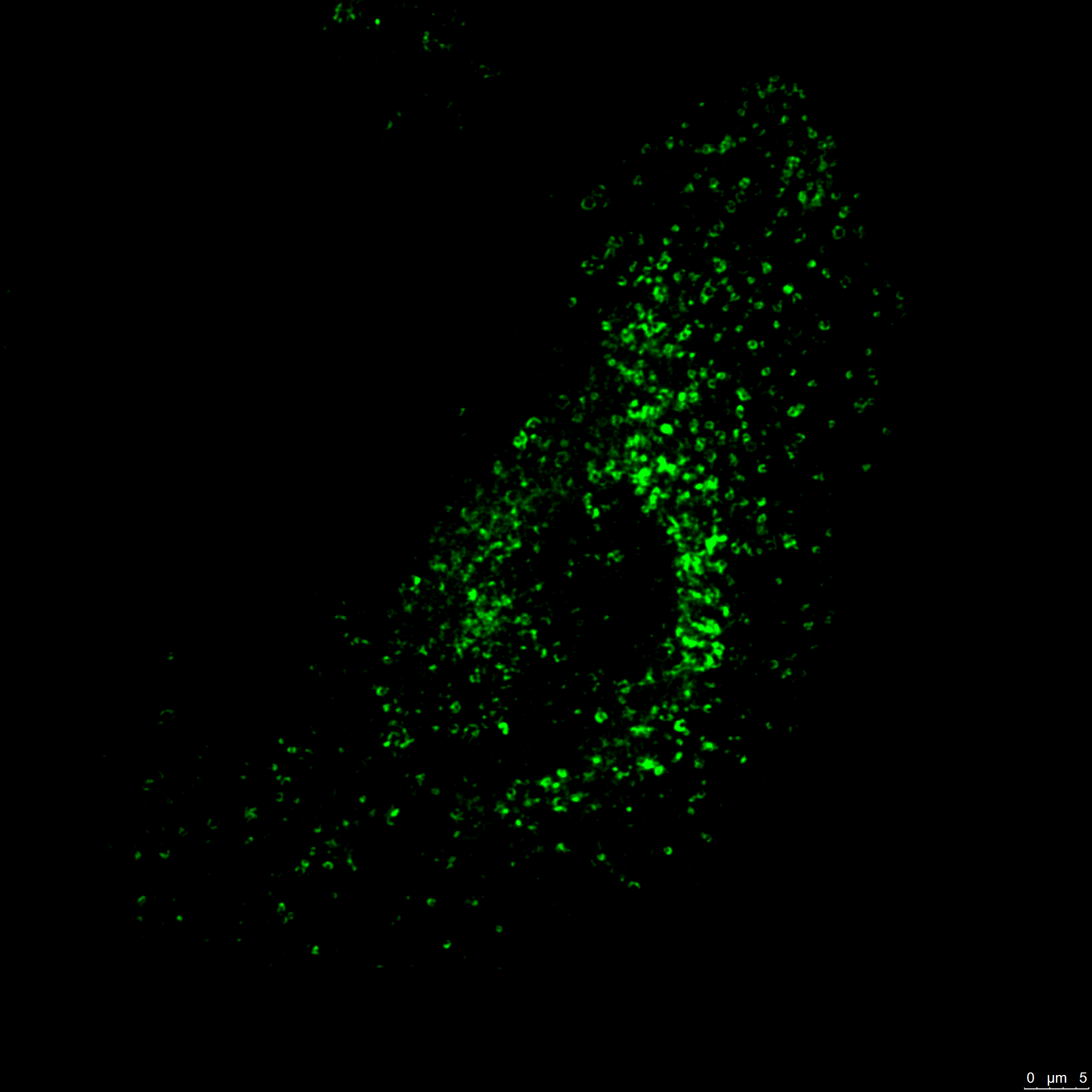

Supplement: Supplementary file 20 — Figure EV7 Source Data [file 44318_2025_654_MOESM20_ESM.zip › EV Figure 7/EV7I/EV7I-3-shUBAC2-AREL1(C790A)-EGFP.tif]

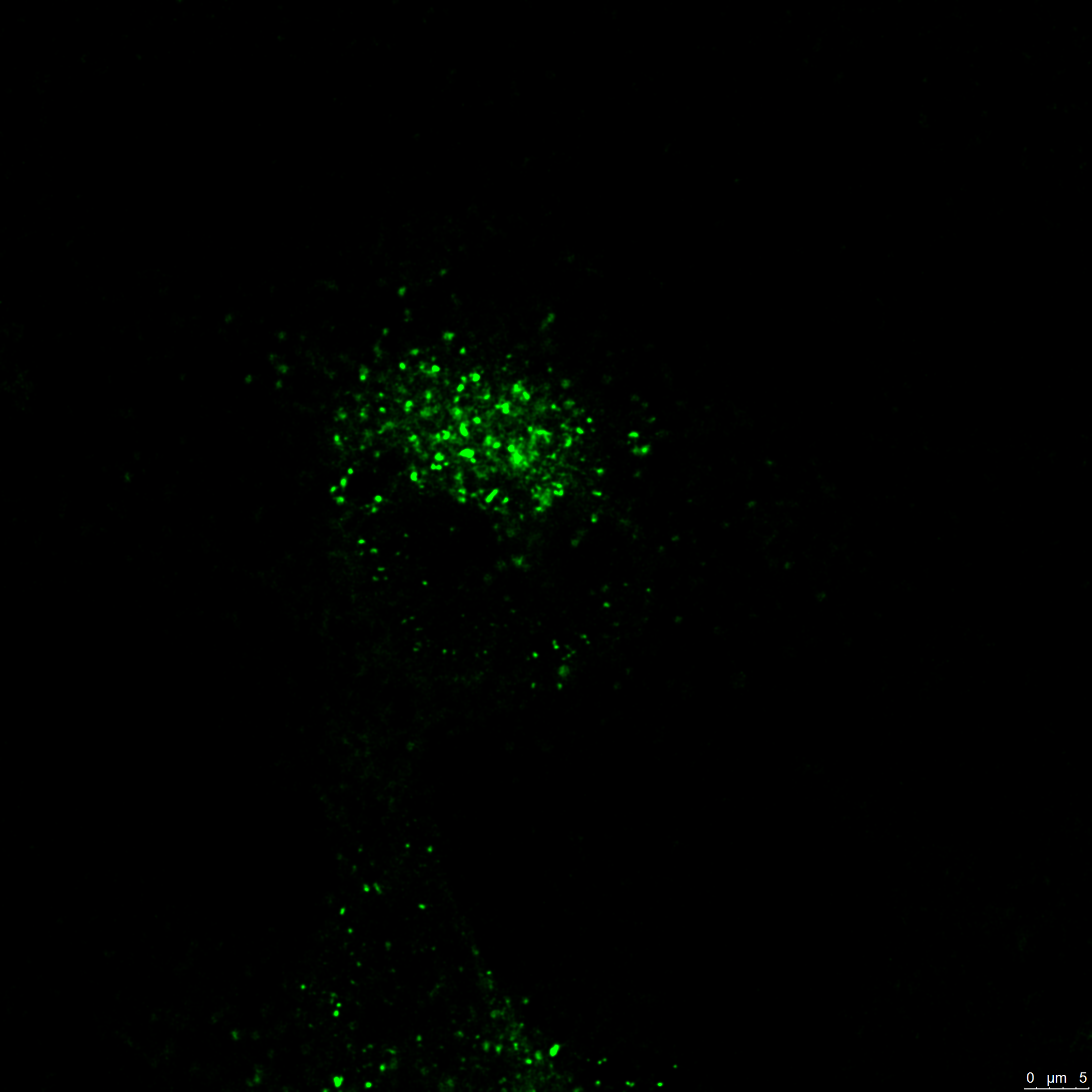

Supplement: Supplementary file 20 — Figure EV7 Source Data [file 44318_2025_654_MOESM20_ESM.zip › EV Figure 7/EV7I/EV7I-1-shNC-AREL1(WT)-EGFP.tif]

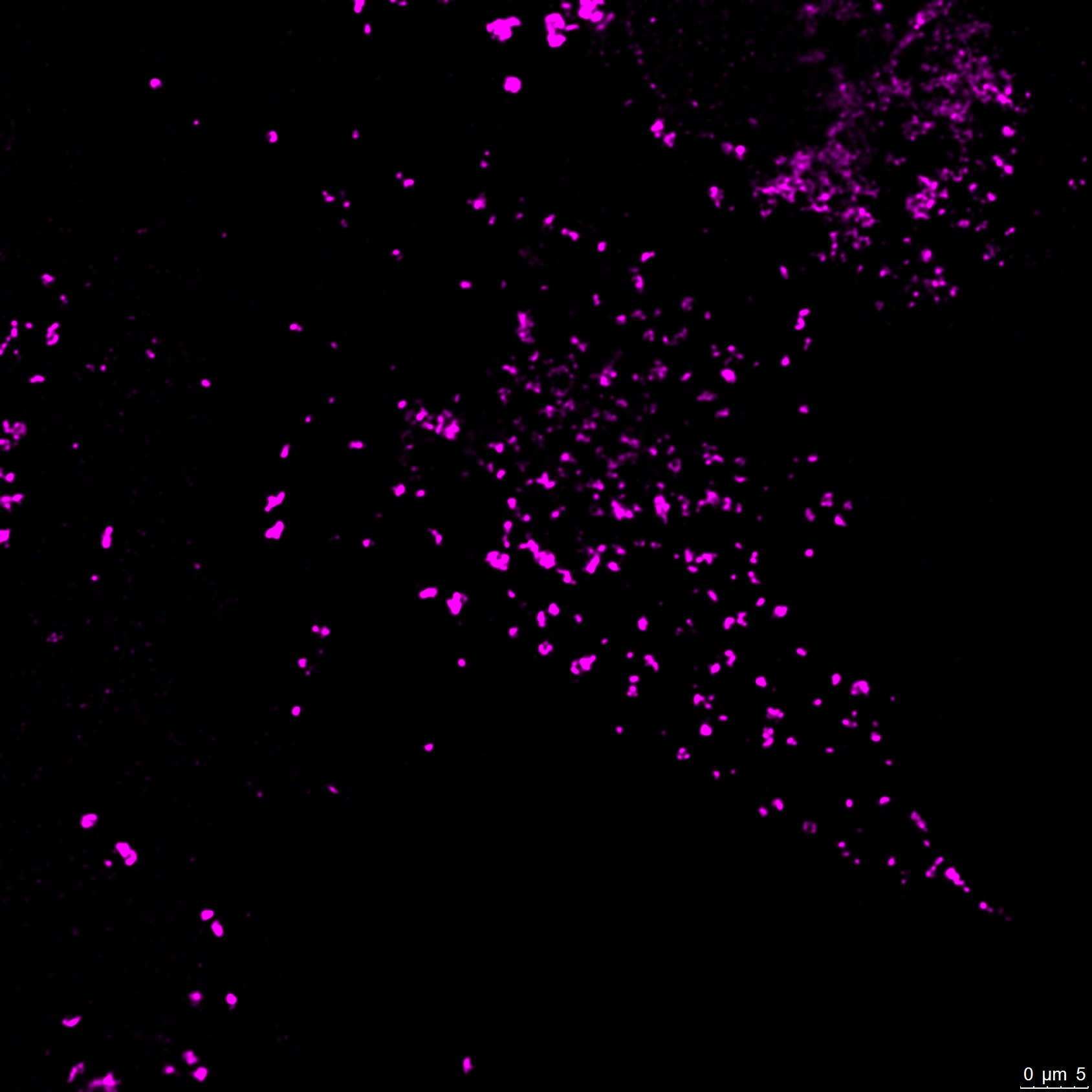

Supplement: Supplementary file 20 — Figure EV7 Source Data [file 44318_2025_654_MOESM20_ESM.zip › EV Figure 7/EV7I/EV7I-4-shUBAC2-AREL1(╬öhinge region)-EGFP-LAMP1.tif]

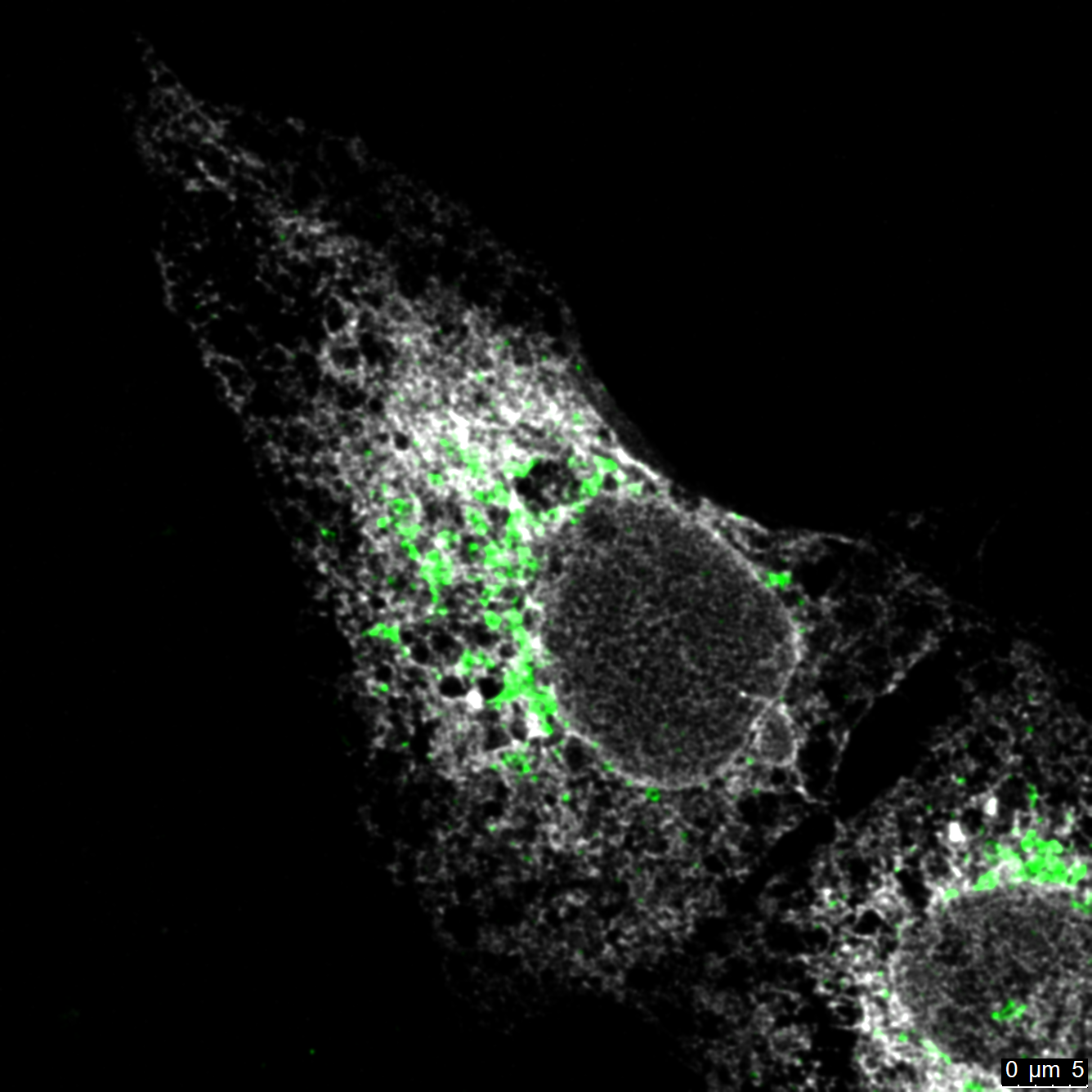

Supplement: Supplementary file 20 — Figure EV7 Source Data [file 44318_2025_654_MOESM20_ESM.zip › EV Figure 7/EV7B/EV7B-2-RHBDD3-EGFP+mCherry-Sec61╬▓ merge.tif]

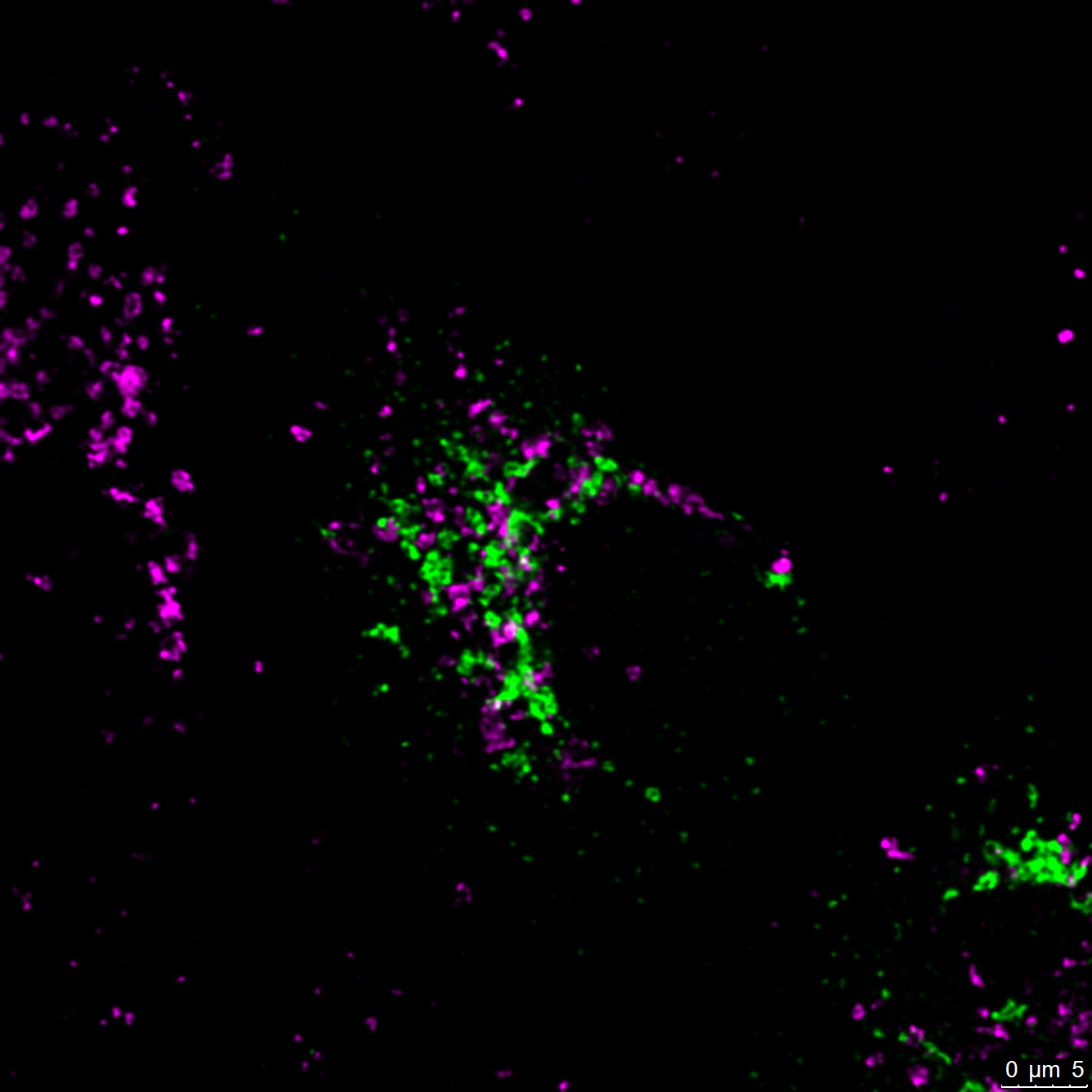

Supplement: Supplementary file 20 — Figure EV7 Source Data [file 44318_2025_654_MOESM20_ESM.zip › EV Figure 7/EV7B/EV7B-2-RHBDD3-EGFP+LAMP1 merge.tif]

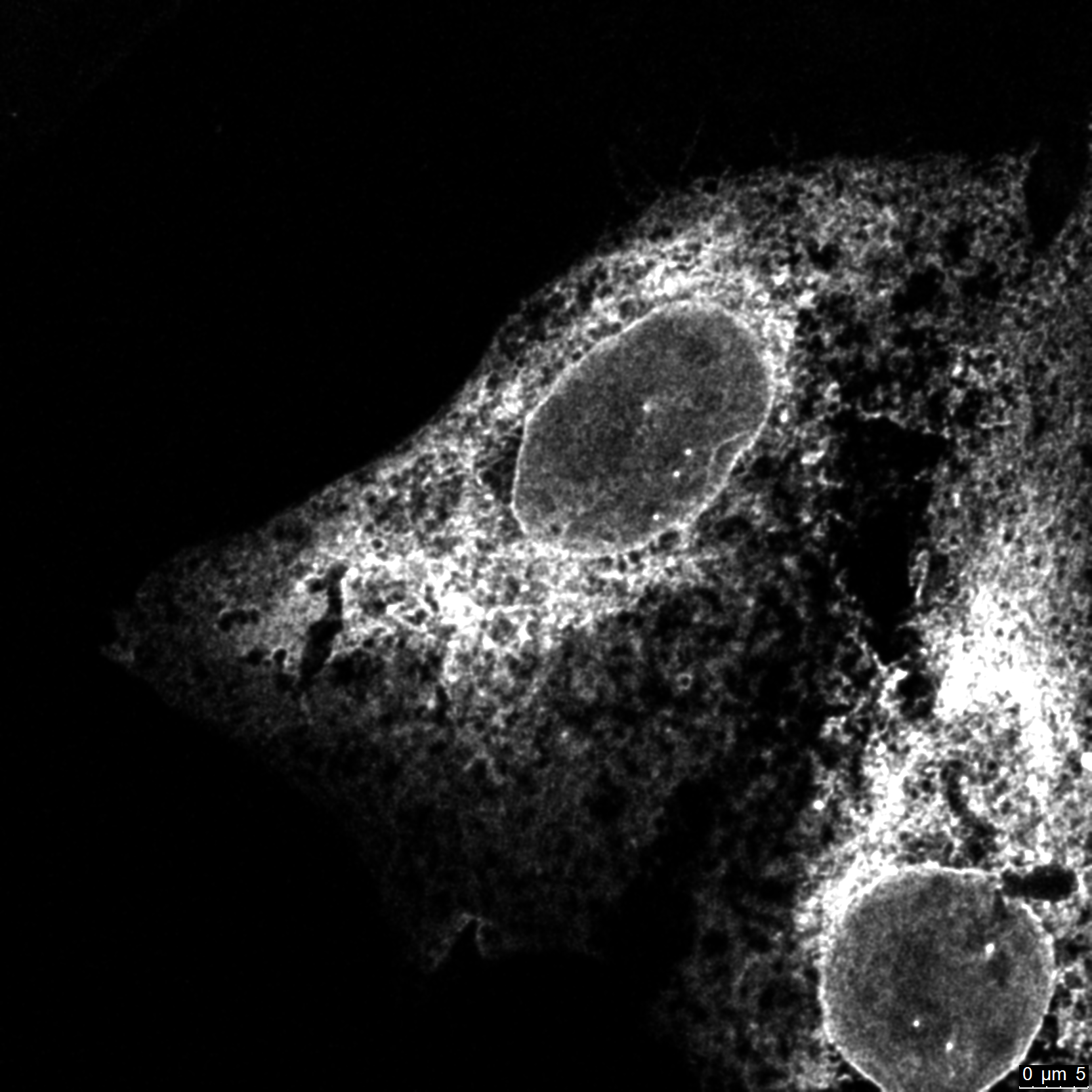

Supplement: Supplementary file 20 — Figure EV7 Source Data [file 44318_2025_654_MOESM20_ESM.zip › EV Figure 7/EV7B/EV7B-1-AUP1-EGFP_mCherry-Sec61╬▓.tif]

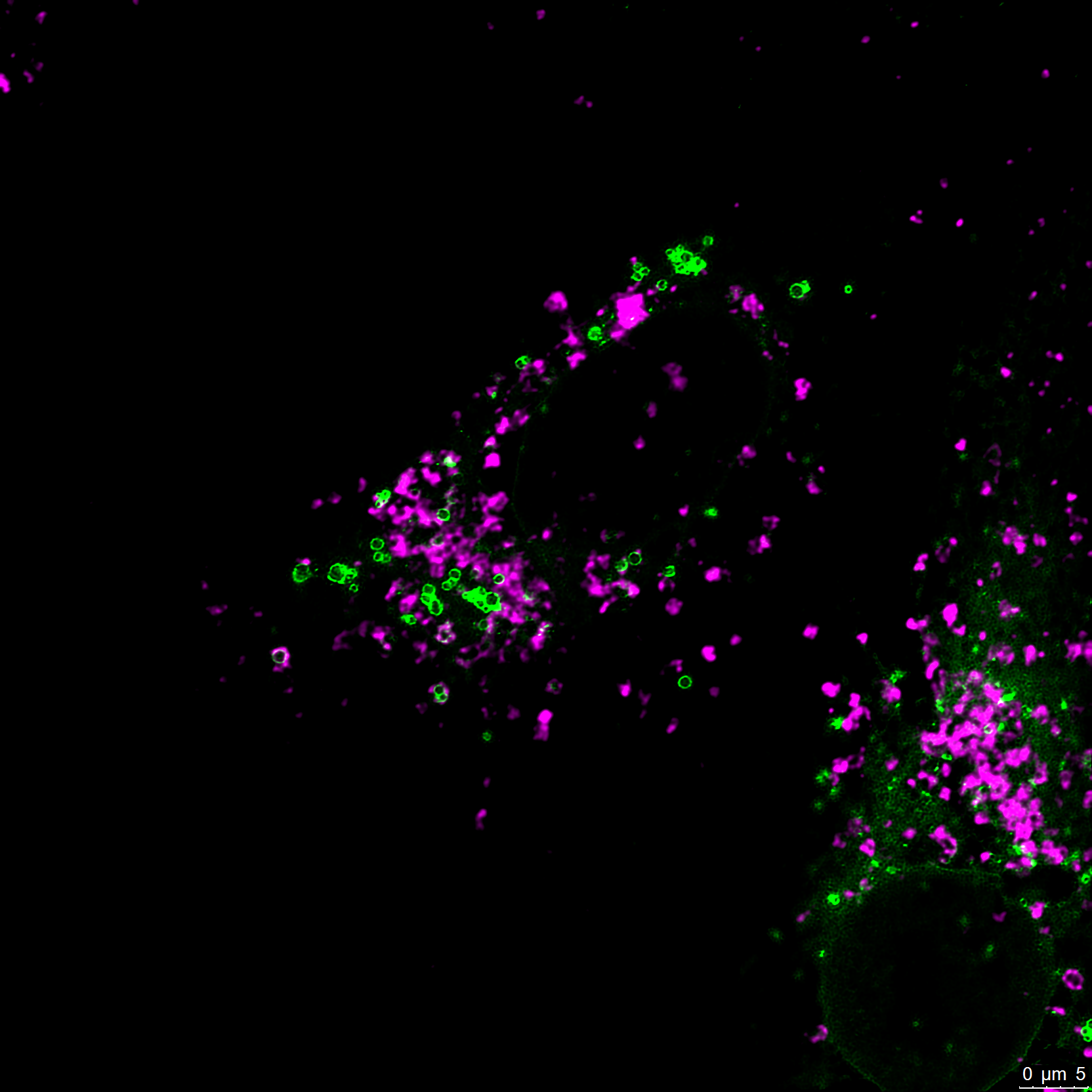

Supplement: Supplementary file 20 — Figure EV7 Source Data [file 44318_2025_654_MOESM20_ESM.zip › EV Figure 7/EV7B/EV7B-1-AUP1-EGFP+LAMP1 merge.tif]

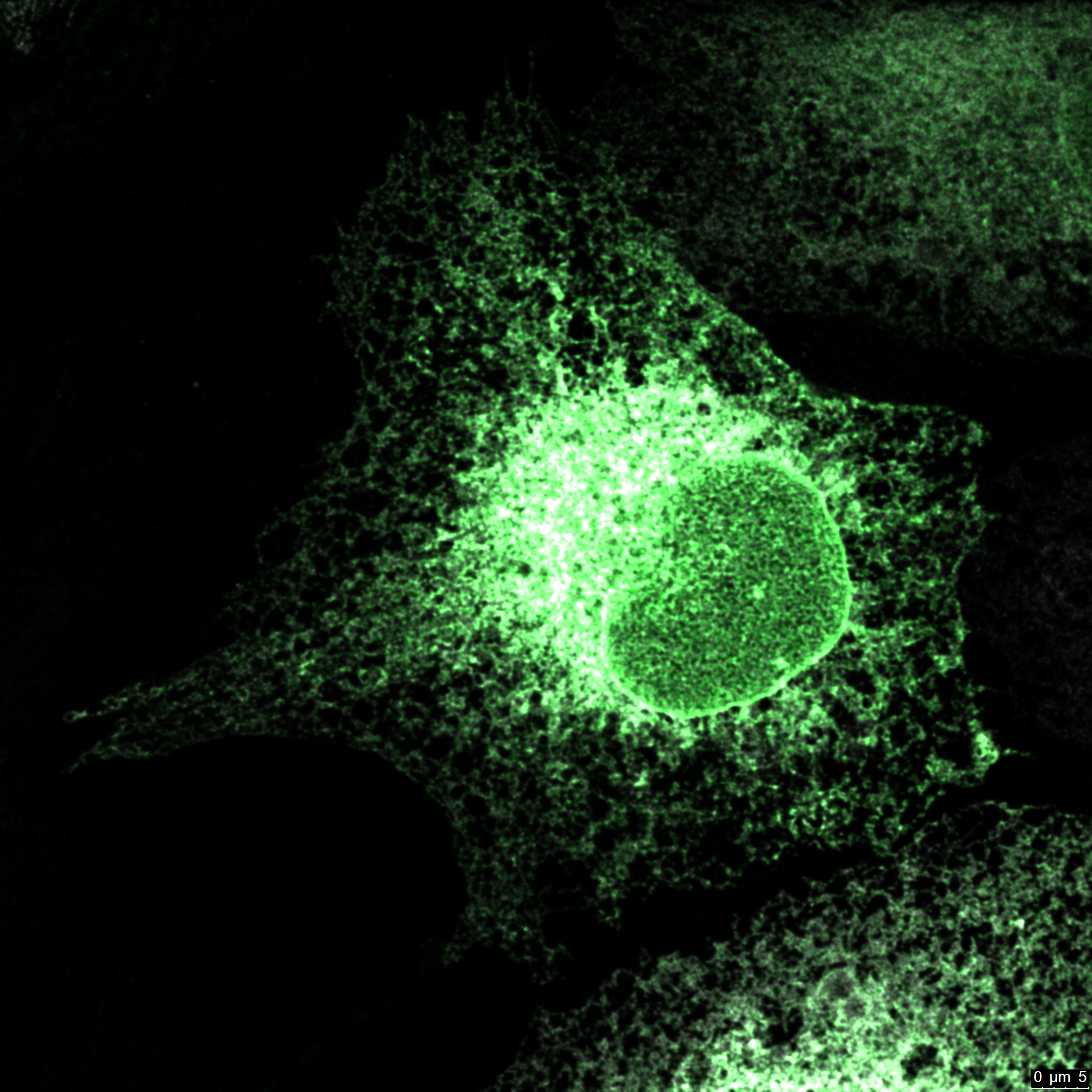

Supplement: Supplementary file 20 — Figure EV7 Source Data [file 44318_2025_654_MOESM20_ESM.zip › EV Figure 7/EV7B/EV7B-3-UBAC2-EGFP+mCherry-Sec61╬▓ merge.tif]

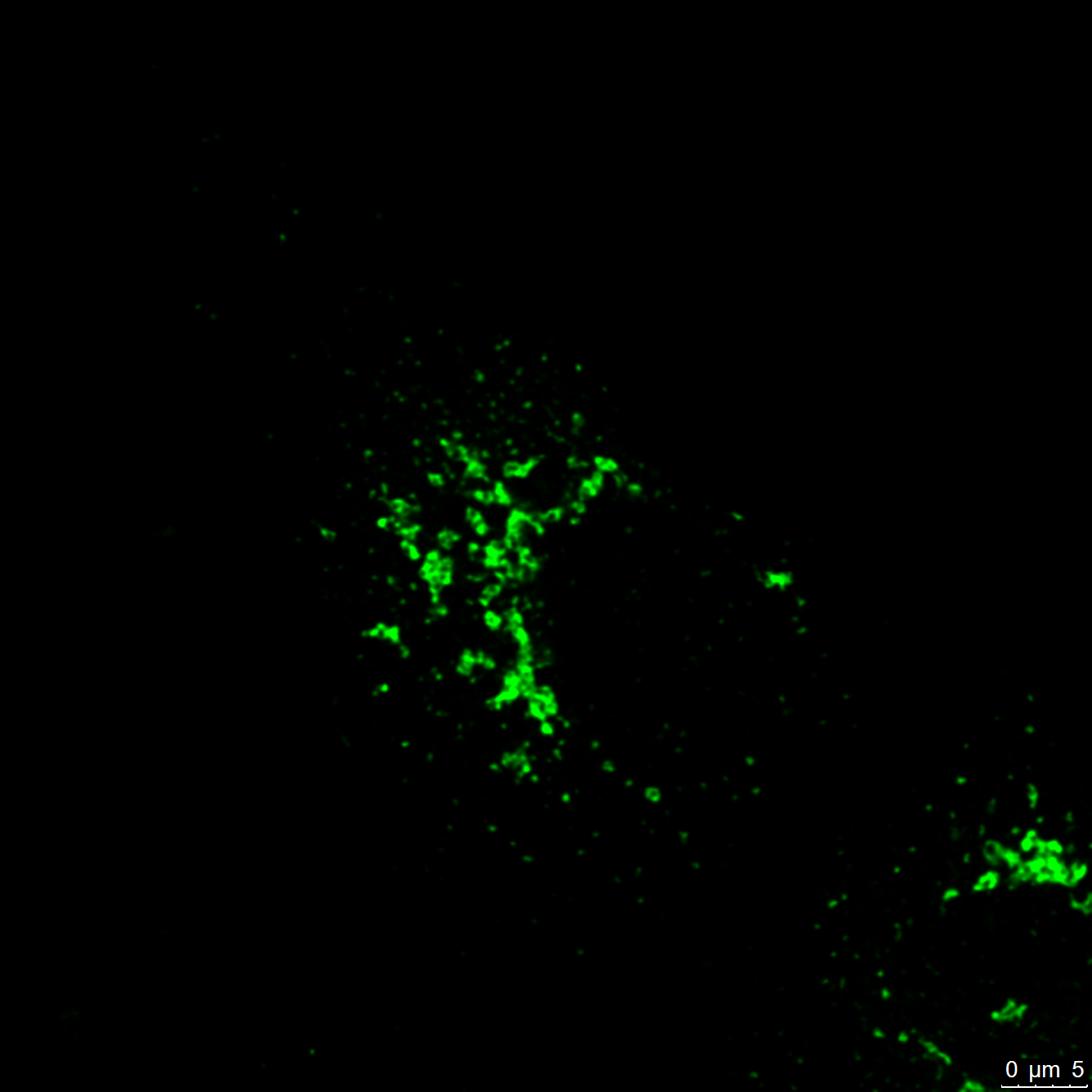

Supplement: Supplementary file 20 — Figure EV7 Source Data [file 44318_2025_654_MOESM20_ESM.zip › EV Figure 7/EV7B/EV7B-2-RHBDD3-EGFP.tif]

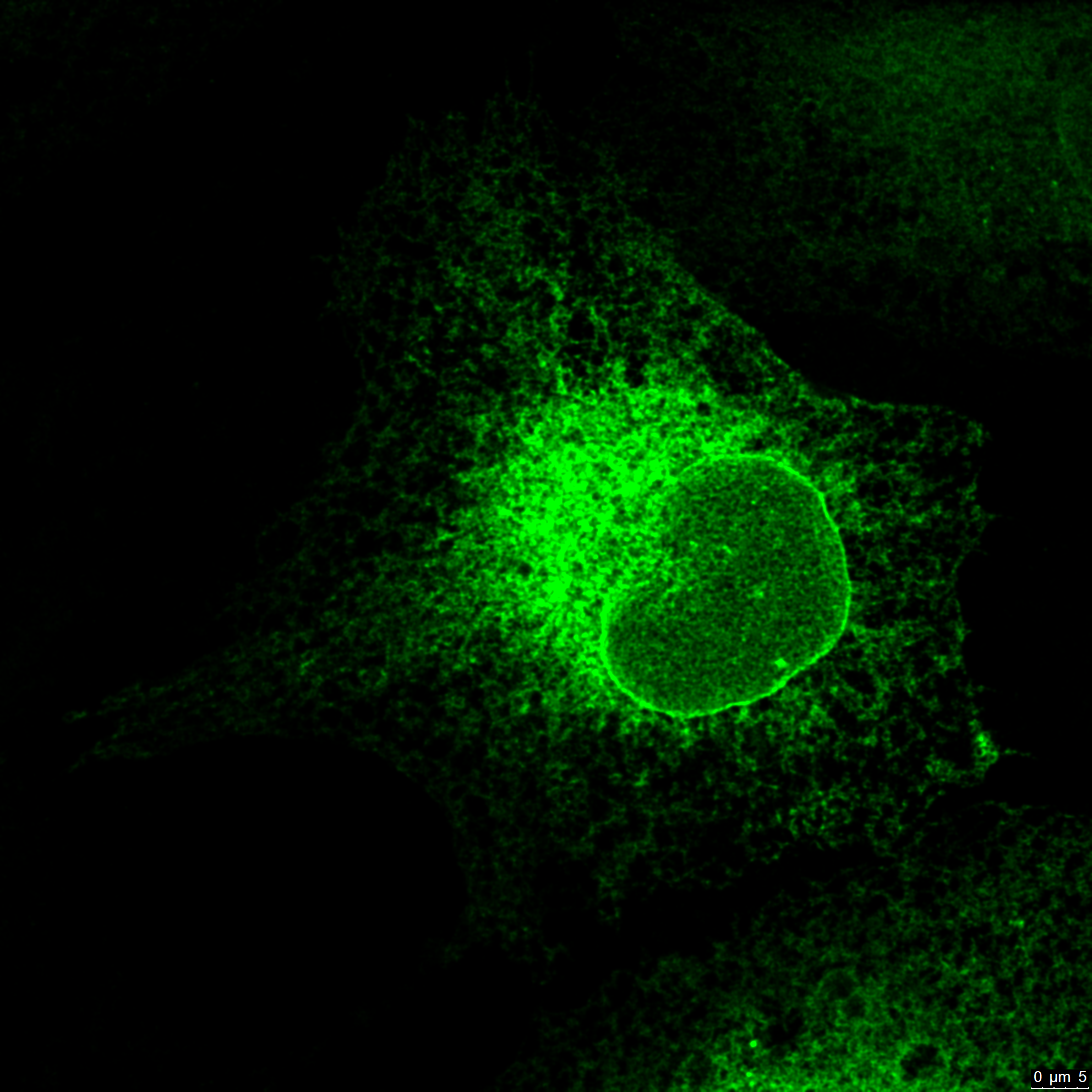

Supplement: Supplementary file 20 — Figure EV7 Source Data [file 44318_2025_654_MOESM20_ESM.zip › EV Figure 7/EV7B/EV7B-3-UBAC2-EGFP.tif]

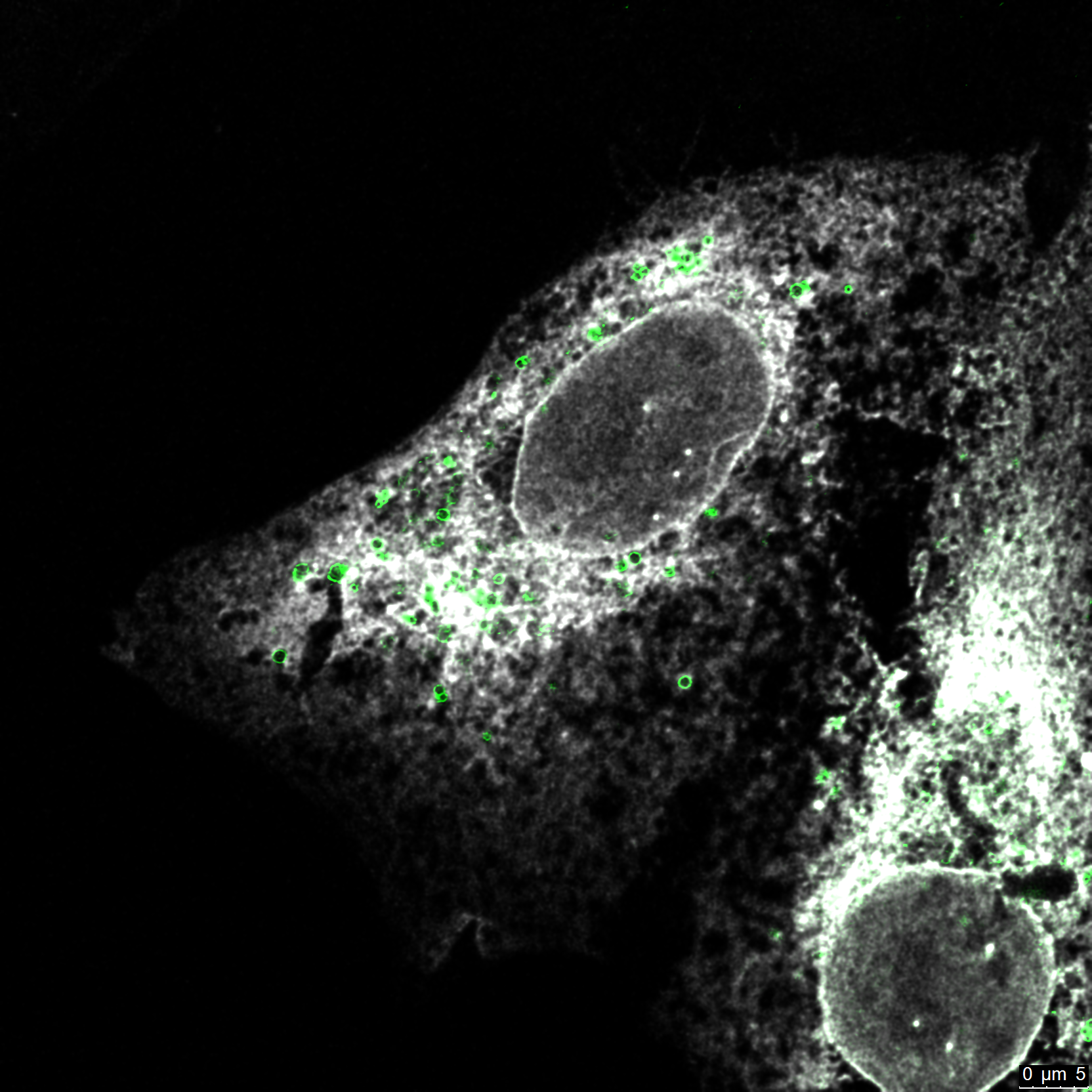

Supplement: Supplementary file 20 — Figure EV7 Source Data [file 44318_2025_654_MOESM20_ESM.zip › EV Figure 7/EV7B/EV7B-1-AUP1-EGFP+mCherry-Sec61╬▓ merge.tif]

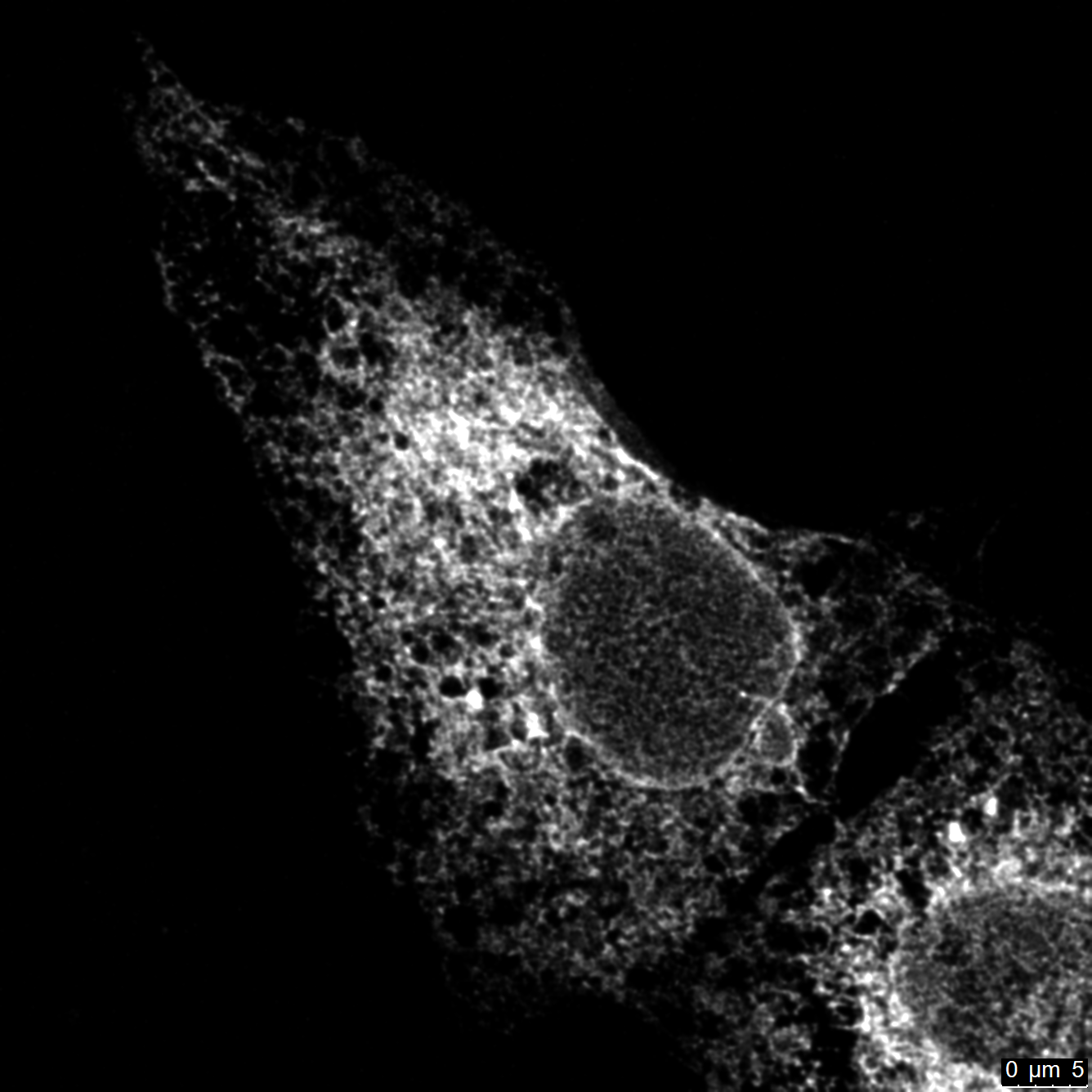

Supplement: Supplementary file 20 — Figure EV7 Source Data [file 44318_2025_654_MOESM20_ESM.zip › EV Figure 7/EV7B/EV7B-2-RHBDD3-EGFP_mCherry-Sec61╬▓.tif]

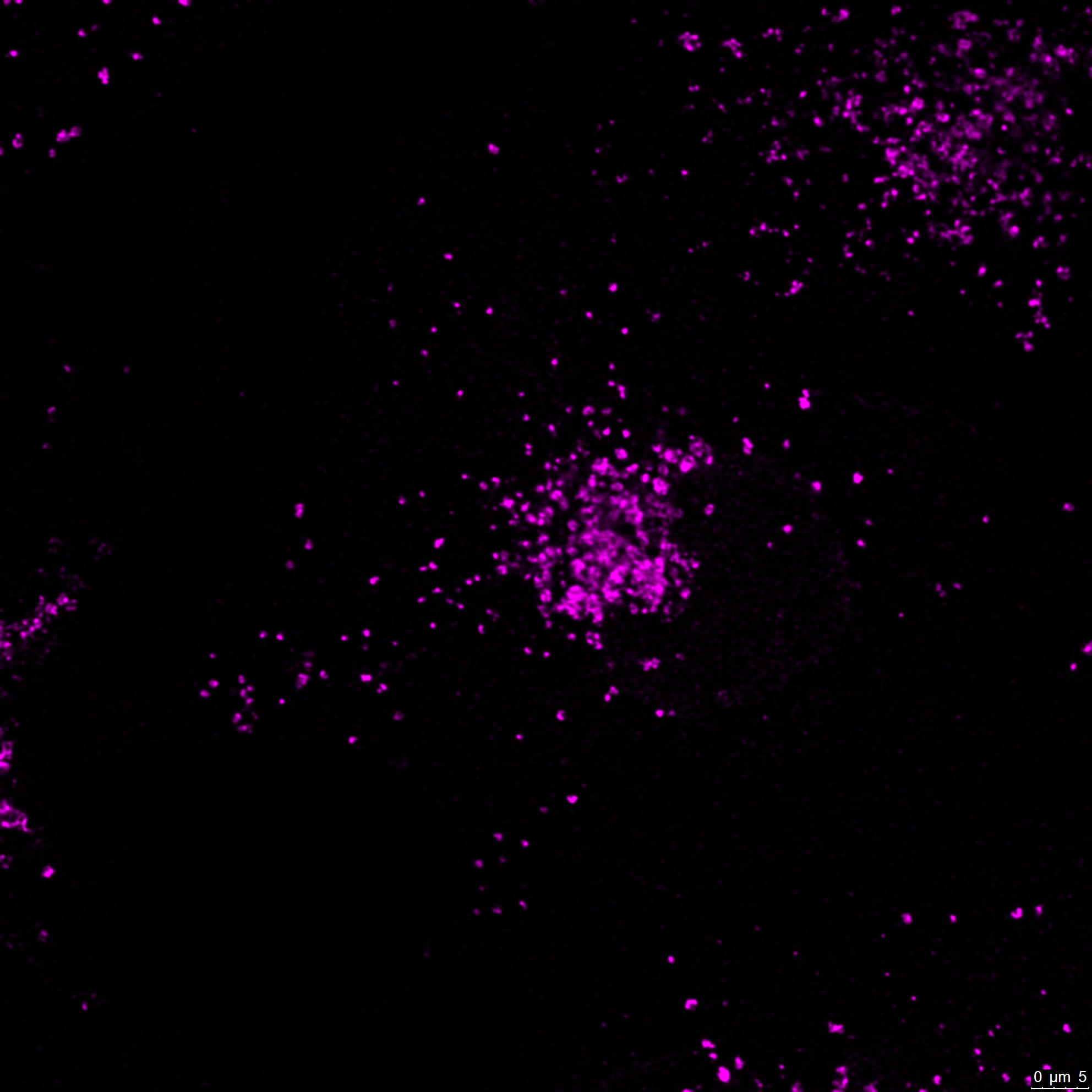

Supplement: Supplementary file 20 — Figure EV7 Source Data [file 44318_2025_654_MOESM20_ESM.zip › EV Figure 7/EV7B/EV7B-3-UBAC2-EGFP_LAMP1.tif]

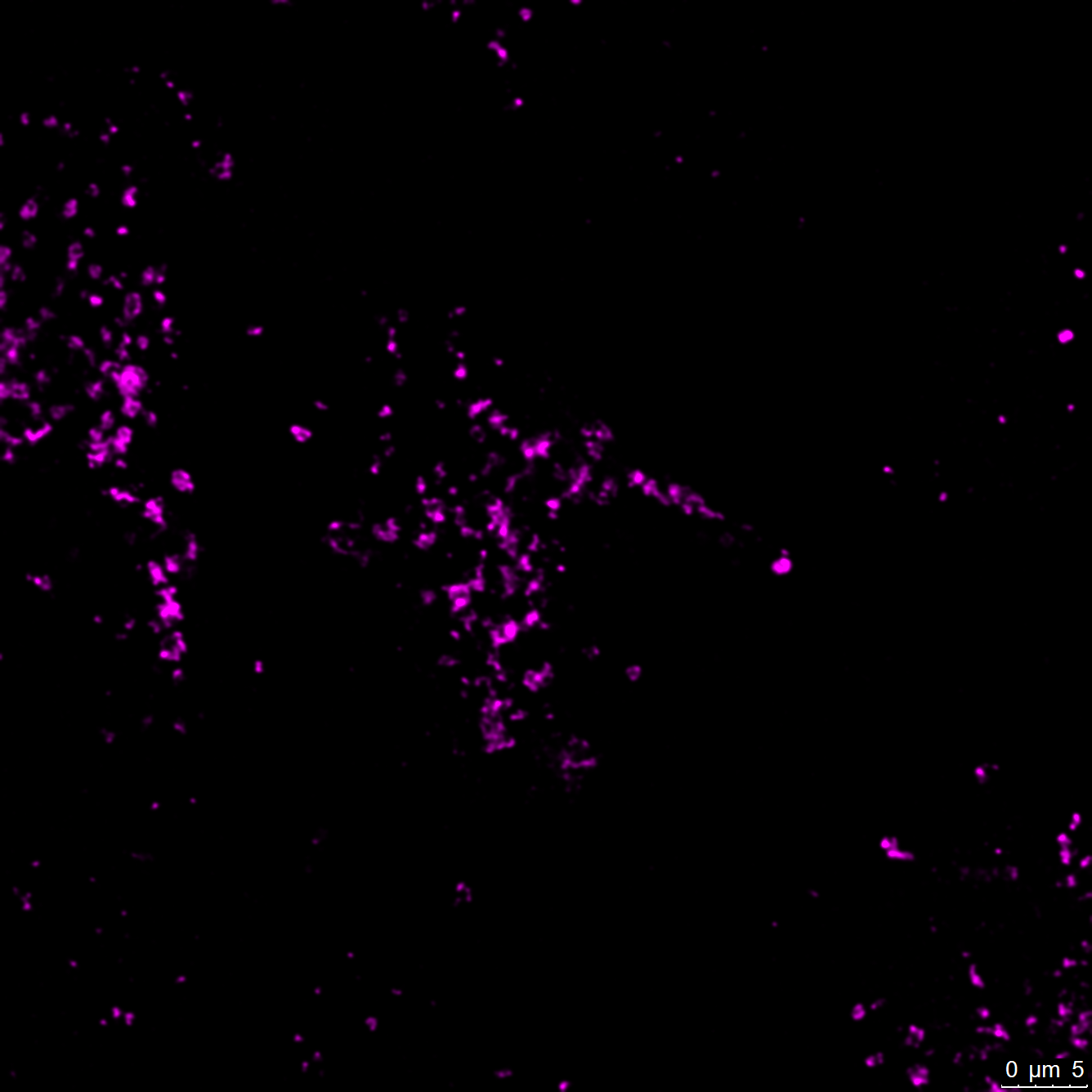

Supplement: Supplementary file 20 — Figure EV7 Source Data [file 44318_2025_654_MOESM20_ESM.zip › EV Figure 7/EV7B/EV7B-2-RHBDD3-EGFP_LAMP1.tif]

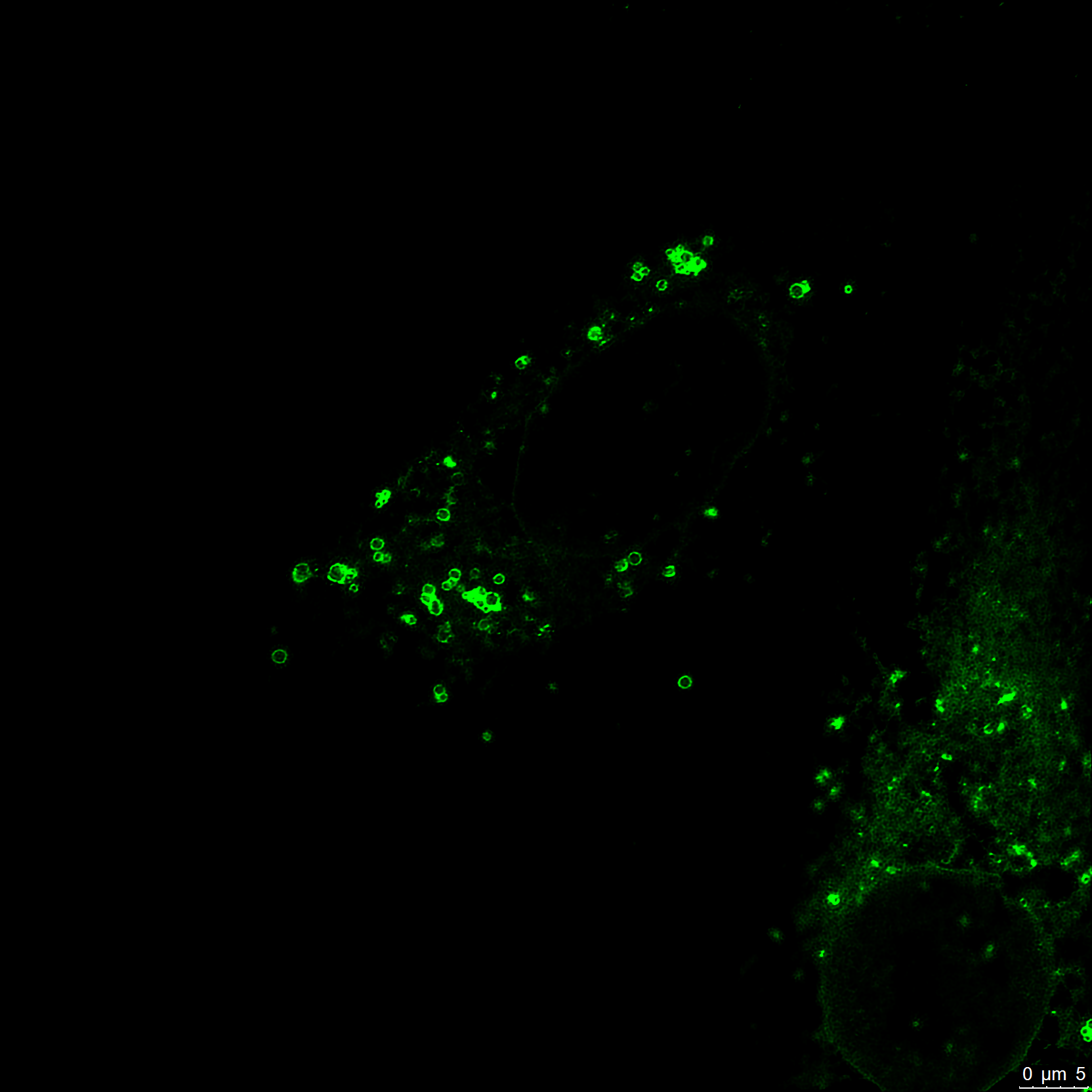

Supplement: Supplementary file 20 — Figure EV7 Source Data [file 44318_2025_654_MOESM20_ESM.zip › EV Figure 7/EV7B/EV7B-1-AUP1-EGFP.tif]

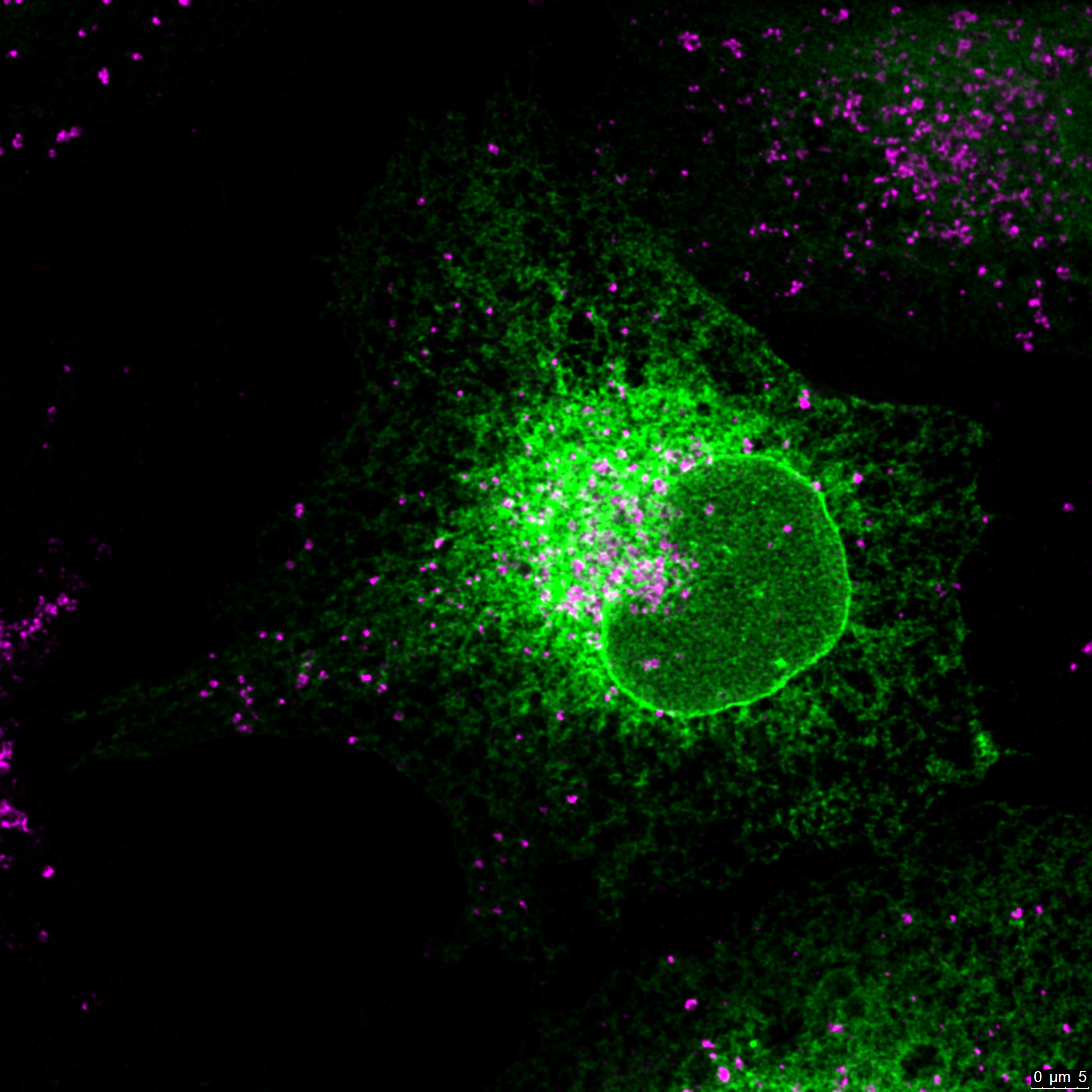

Supplement: Supplementary file 20 — Figure EV7 Source Data [file 44318_2025_654_MOESM20_ESM.zip › EV Figure 7/EV7B/EV7B-3-UBAC2-EGFP+LAMP1 merge.tif]

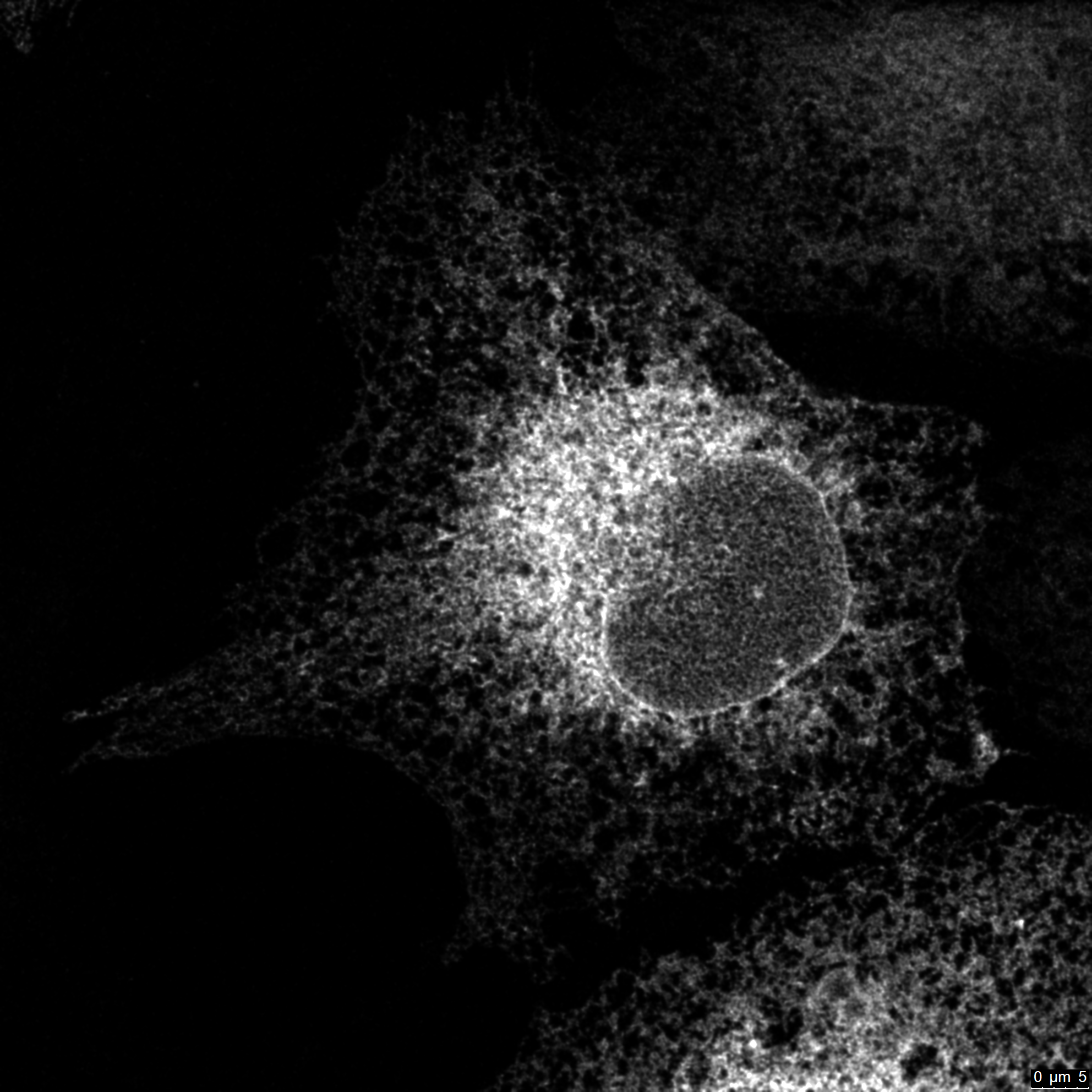

Supplement: Supplementary file 20 — Figure EV7 Source Data [file 44318_2025_654_MOESM20_ESM.zip › EV Figure 7/EV7B/EV7B-3-UBAC2-EGFP_mCherry-Sec61╬▓.tif]

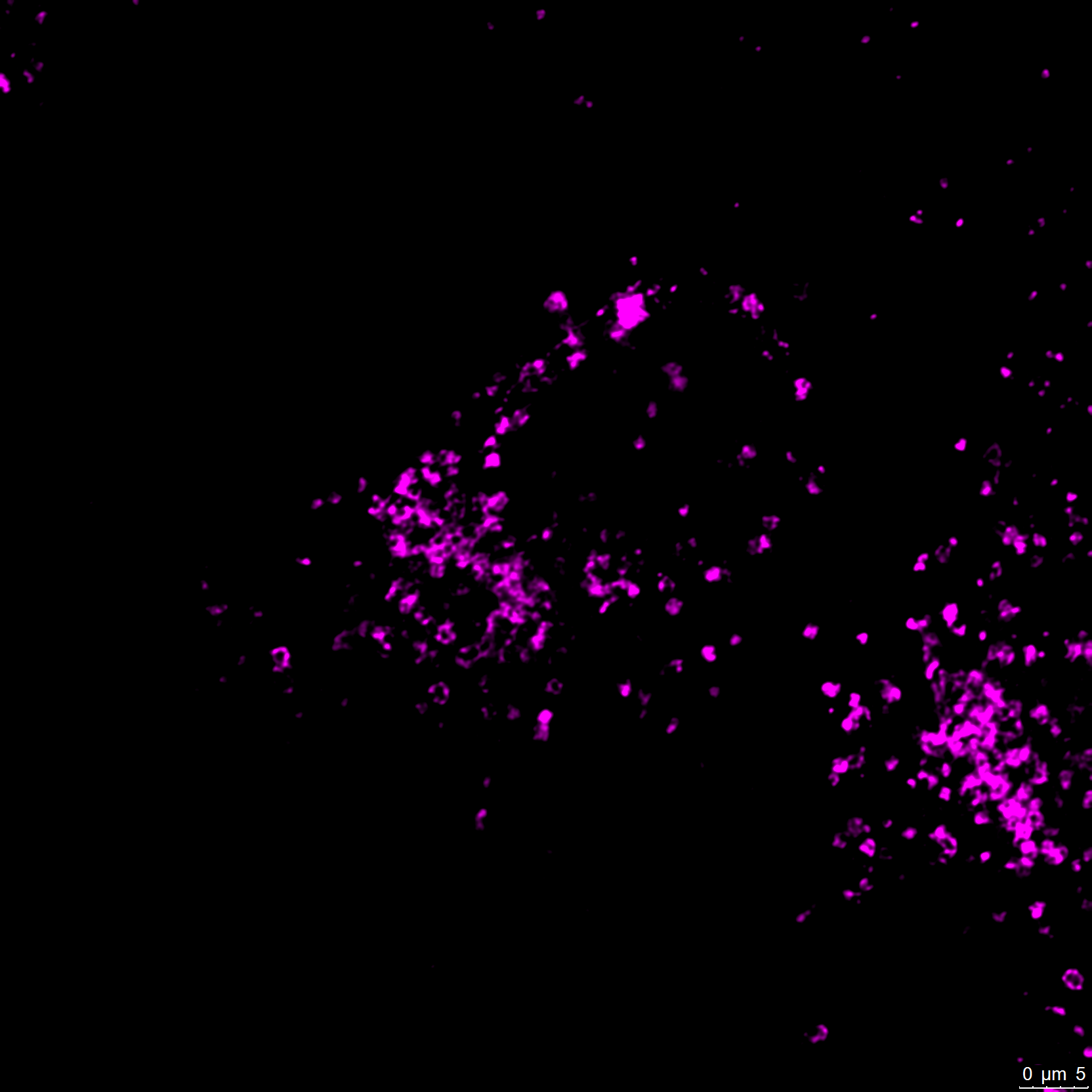

Supplement: Supplementary file 20 — Figure EV7 Source Data [file 44318_2025_654_MOESM20_ESM.zip › EV Figure 7/EV7B/EV7B-1-AUP1-EGFP_LAMP1.tif]

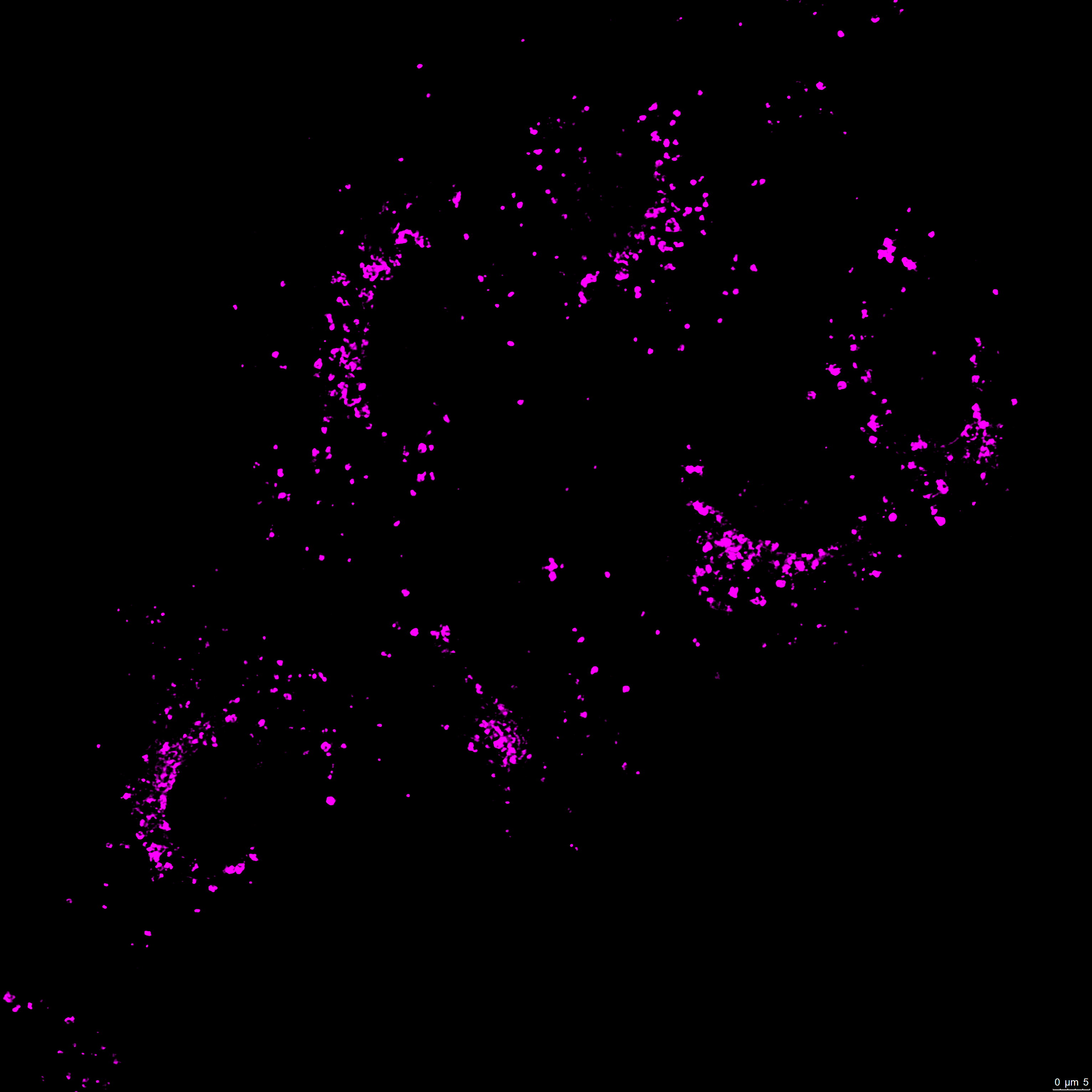

Supplement: Supplementary file 20 — Figure EV7 Source Data [file 44318_2025_654_MOESM20_ESM.zip › EV Figure 7/EV7D/EV7D-3-shRHBDD3-LAMP1.tif]

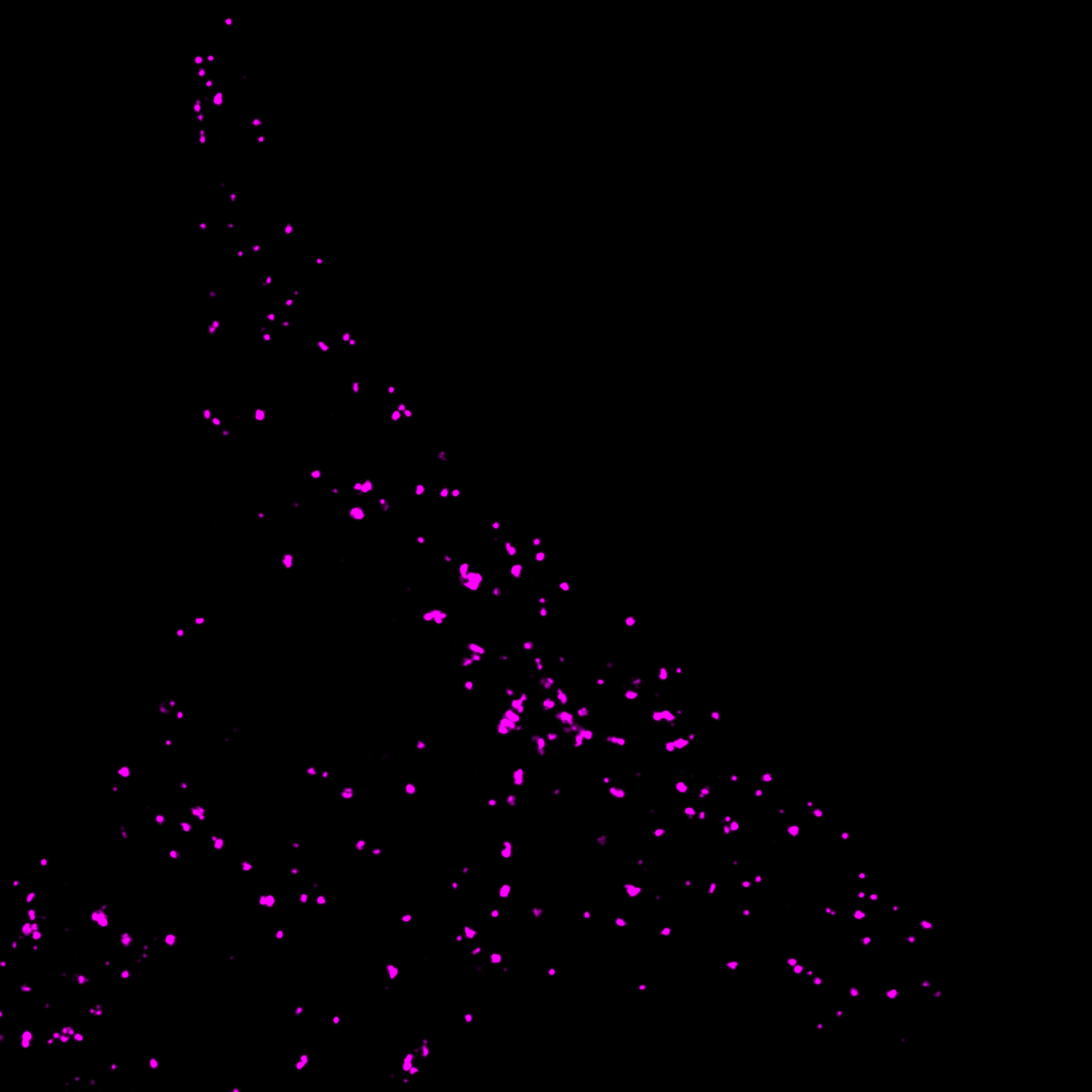

Supplement: Supplementary file 20 — Figure EV7 Source Data [file 44318_2025_654_MOESM20_ESM.zip › EV Figure 7/EV7D/EV7D-4-shUBAC2-LAMP1.tif]

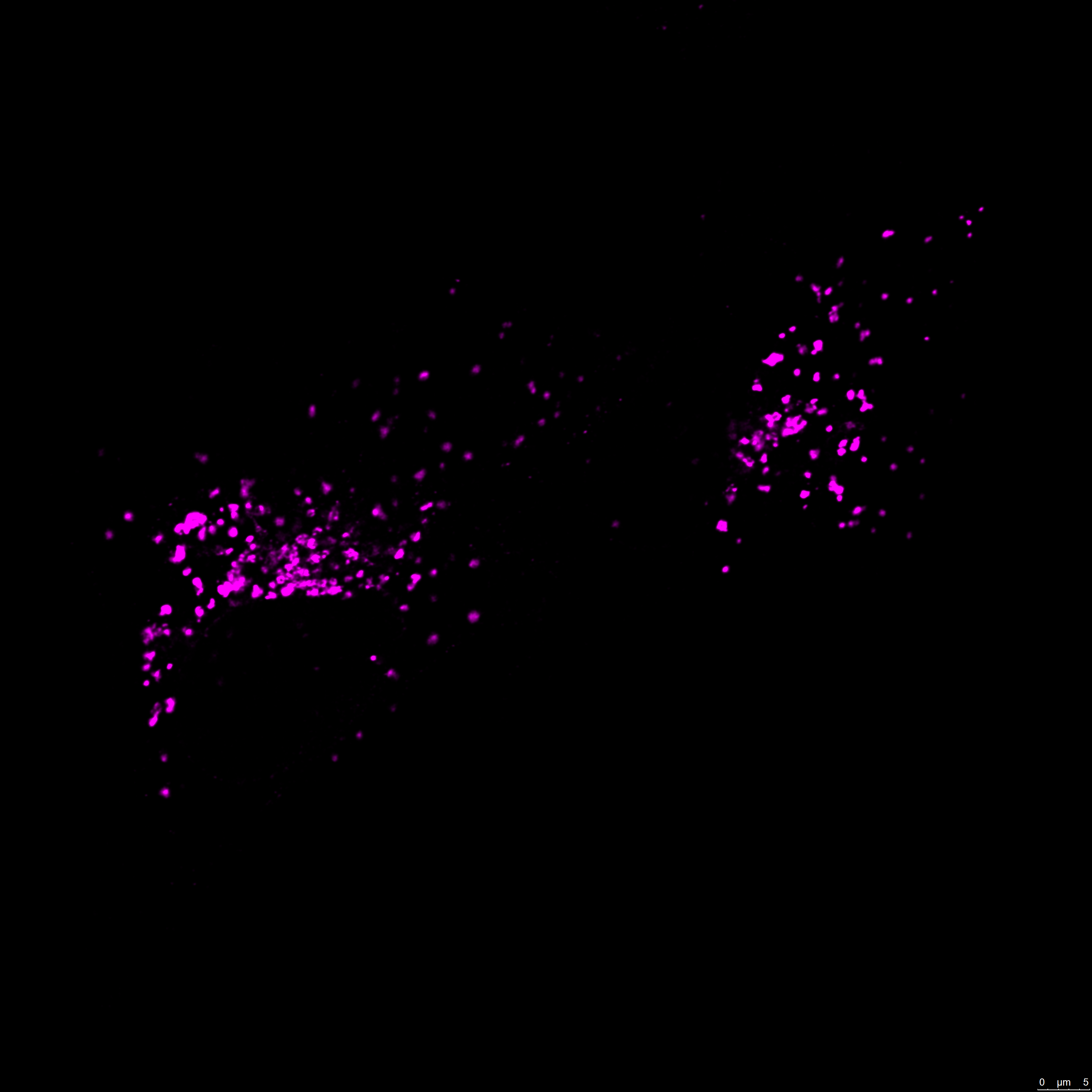

Supplement: Supplementary file 20 — Figure EV7 Source Data [file 44318_2025_654_MOESM20_ESM.zip › EV Figure 7/EV7D/EV7D-1-shNC-LAPM1.tif]

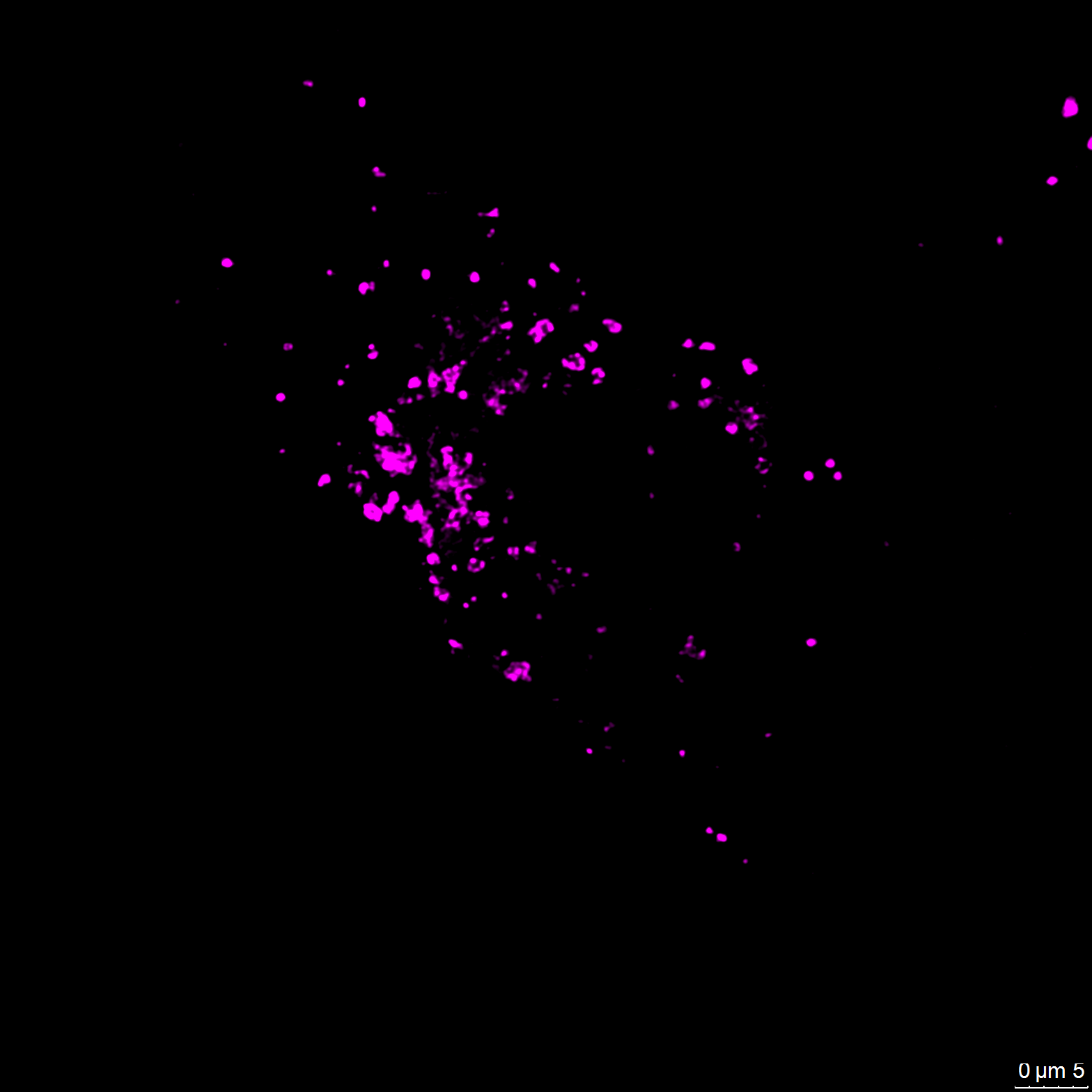

Supplement: Supplementary file 20 — Figure EV7 Source Data [file 44318_2025_654_MOESM20_ESM.zip › EV Figure 7/EV7D/EV7D-2-shAUP1-LAMP1.tif]

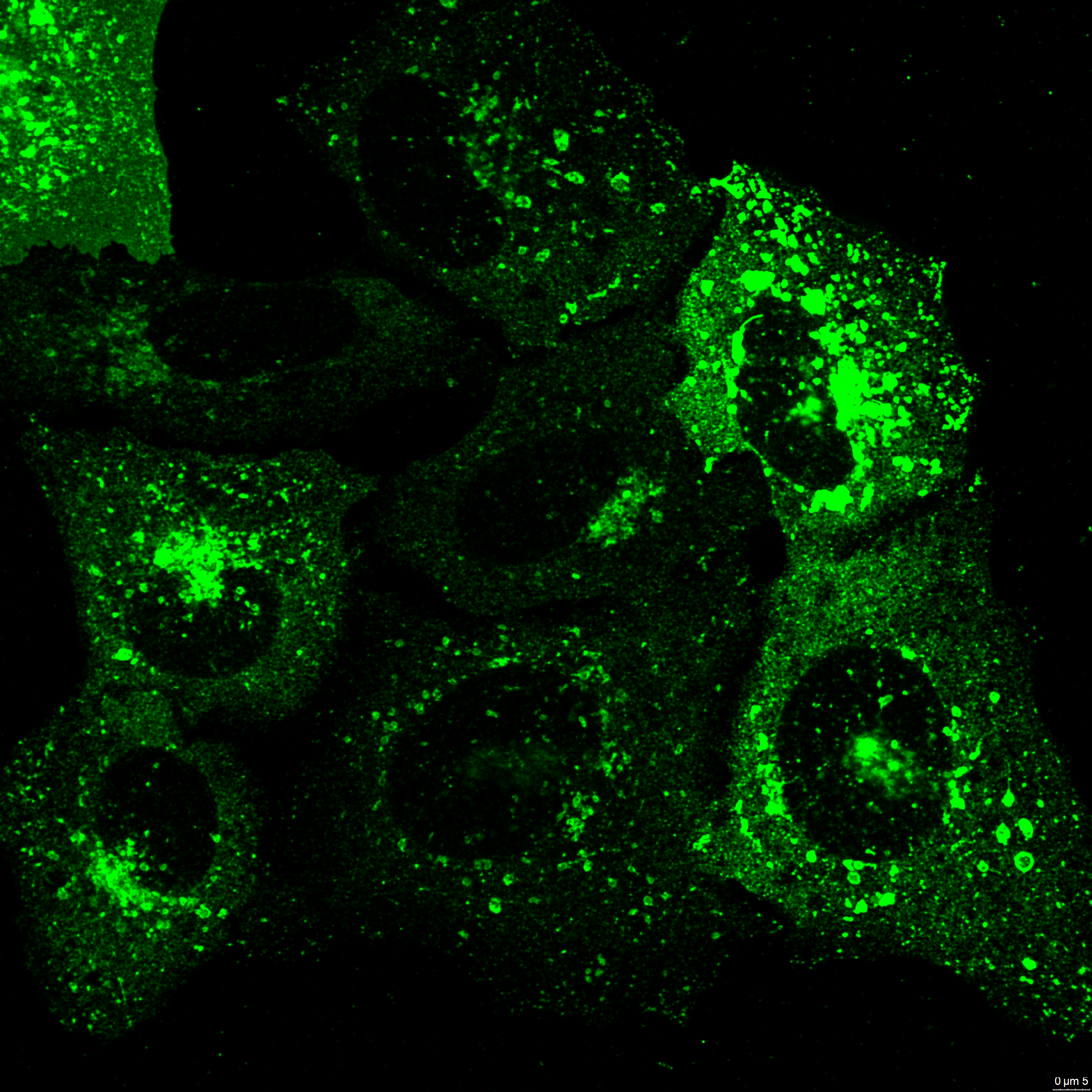

Supplement: Supplementary file 21 — Figure EV8 Source Data [file 44318_2025_654_MOESM21_ESM.zip › EV Figure 8/EV8A/EV8A-LAMP1-EGFP.tif]

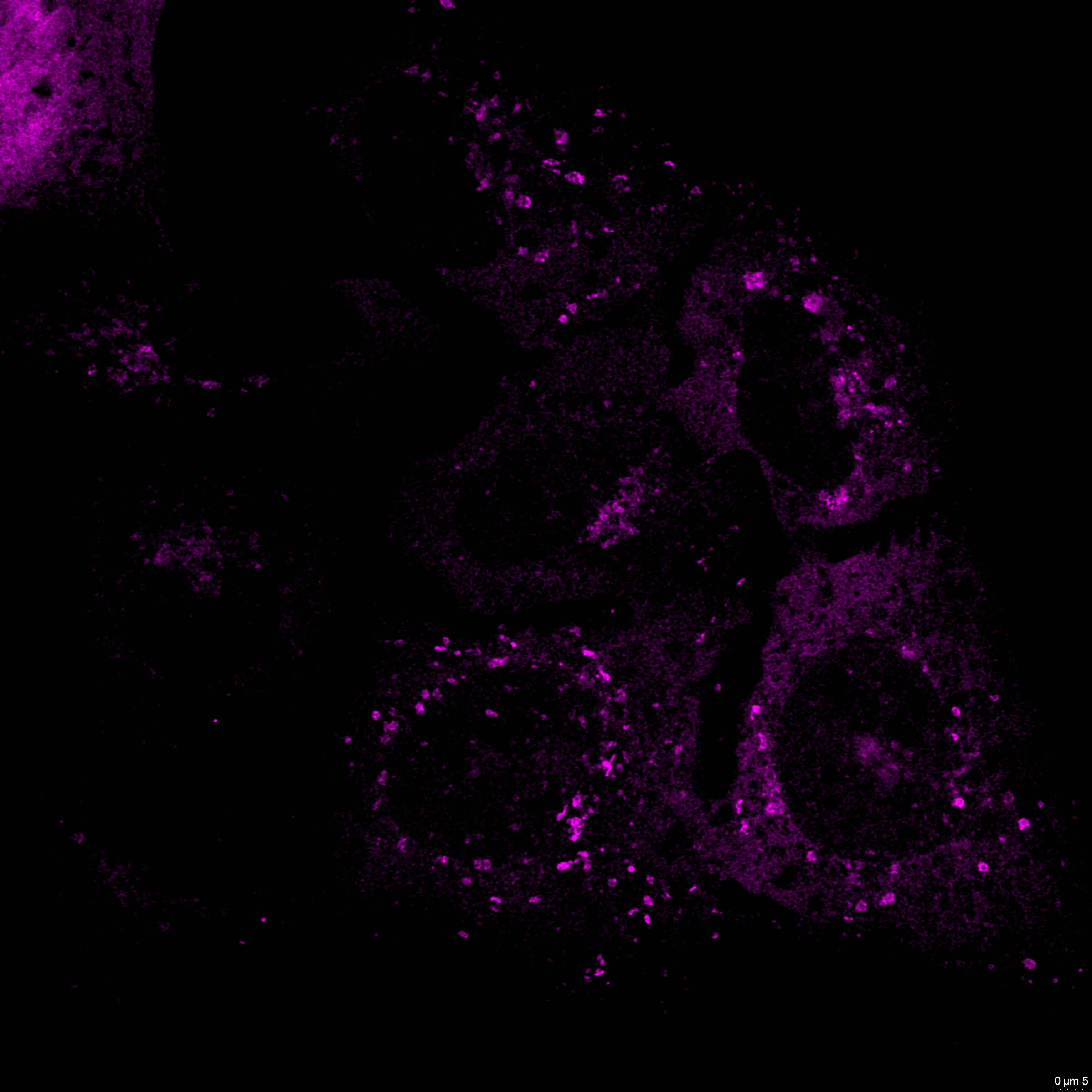

Supplement: Supplementary file 21 — Figure EV8 Source Data [file 44318_2025_654_MOESM21_ESM.zip › EV Figure 8/EV8A/EV8A-V1E1-mCherry.tif]

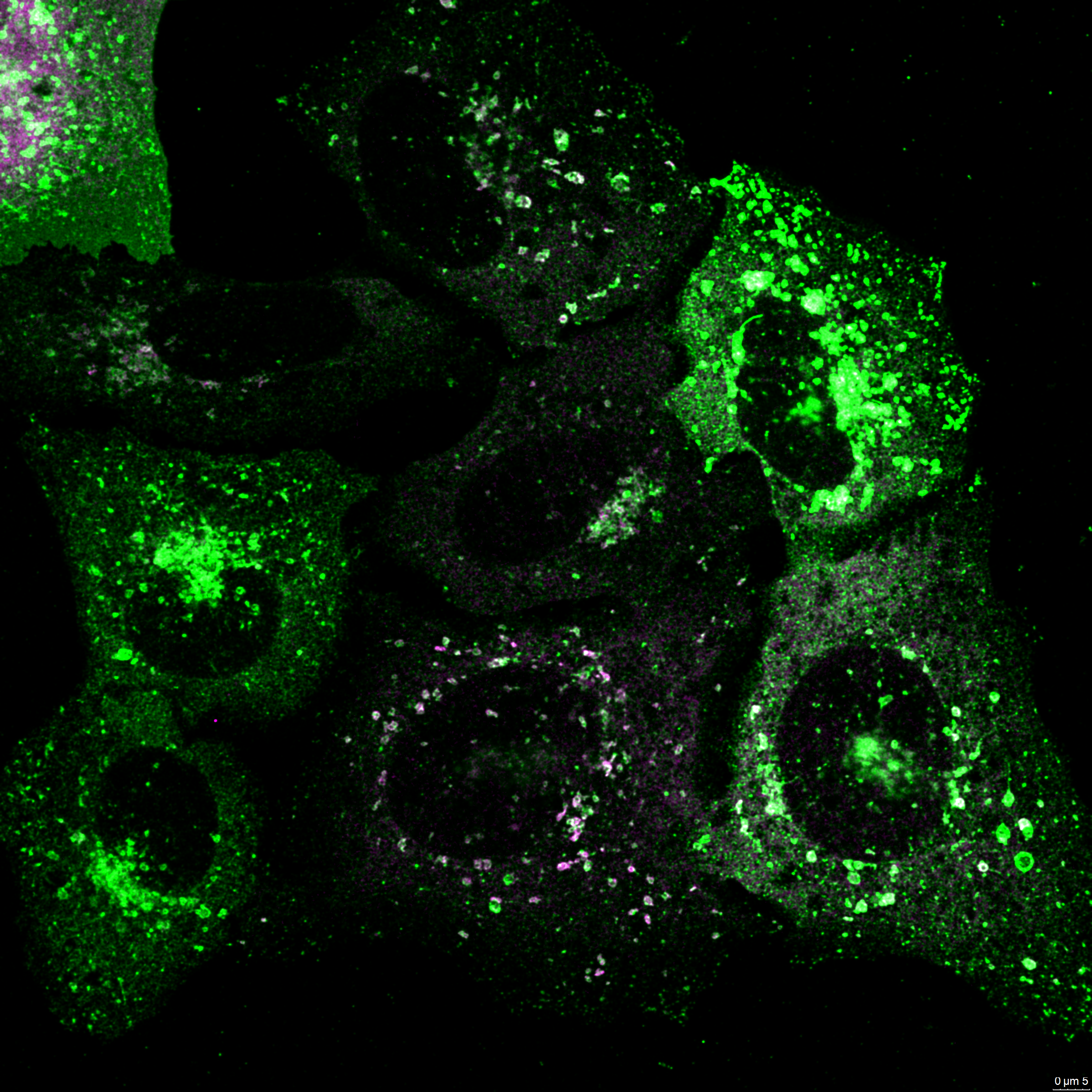

Supplement: Supplementary file 21 — Figure EV8 Source Data [file 44318_2025_654_MOESM21_ESM.zip › EV Figure 8/EV8A/EV8A-V1E1-mCherry+LAMP1-EGFP-merge.tif]

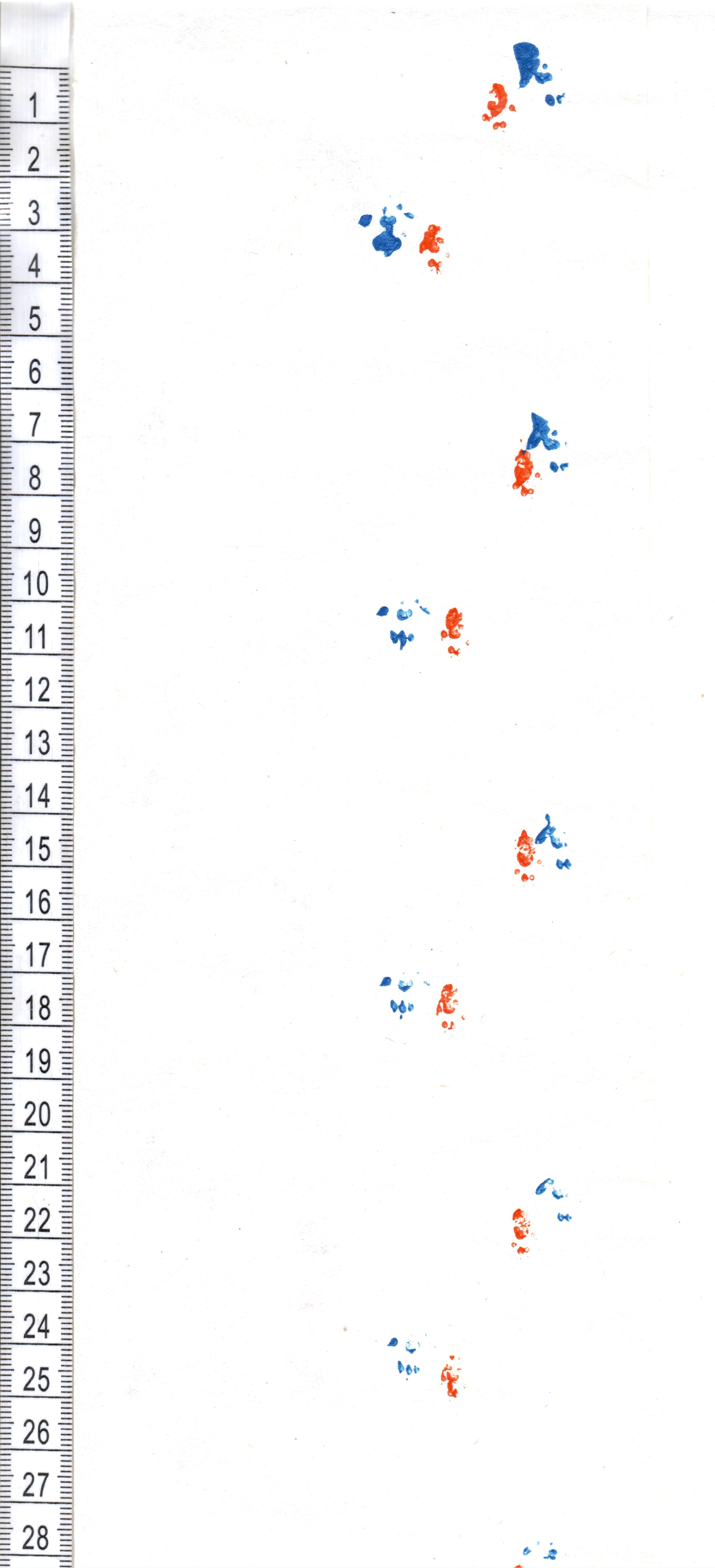

Supplement: Supplementary file 22 — Figure EV9 Source Data [file 44318_2025_654_MOESM22_ESM.zip › EV Figure 9/EV9G/EV9G-1-6 month old male-KO.jpg]

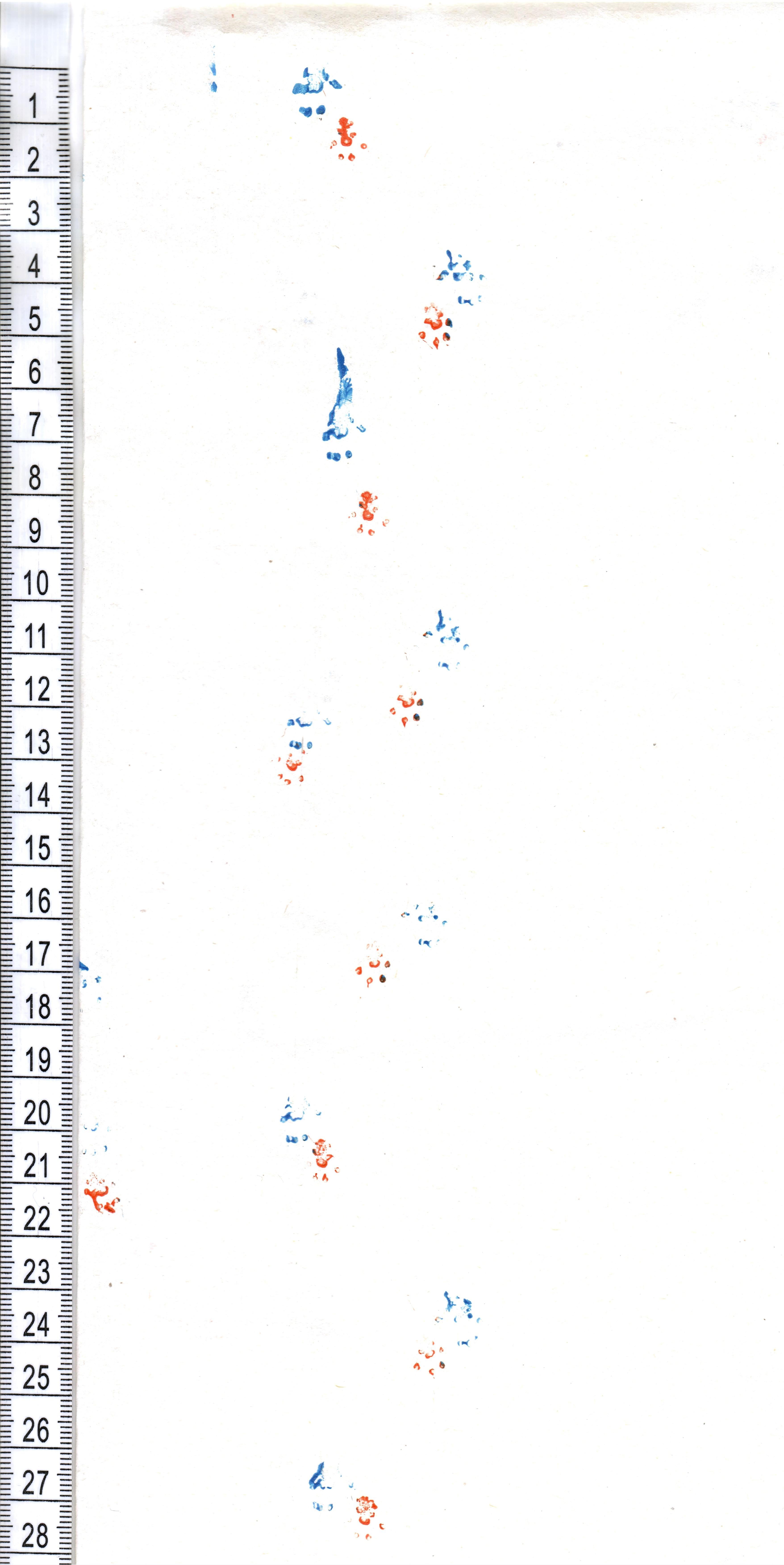

Supplement: Supplementary file 22 — Figure EV9 Source Data [file 44318_2025_654_MOESM22_ESM.zip › EV Figure 9/EV9G/EV9G-2-6 month old male-WT.jpg]

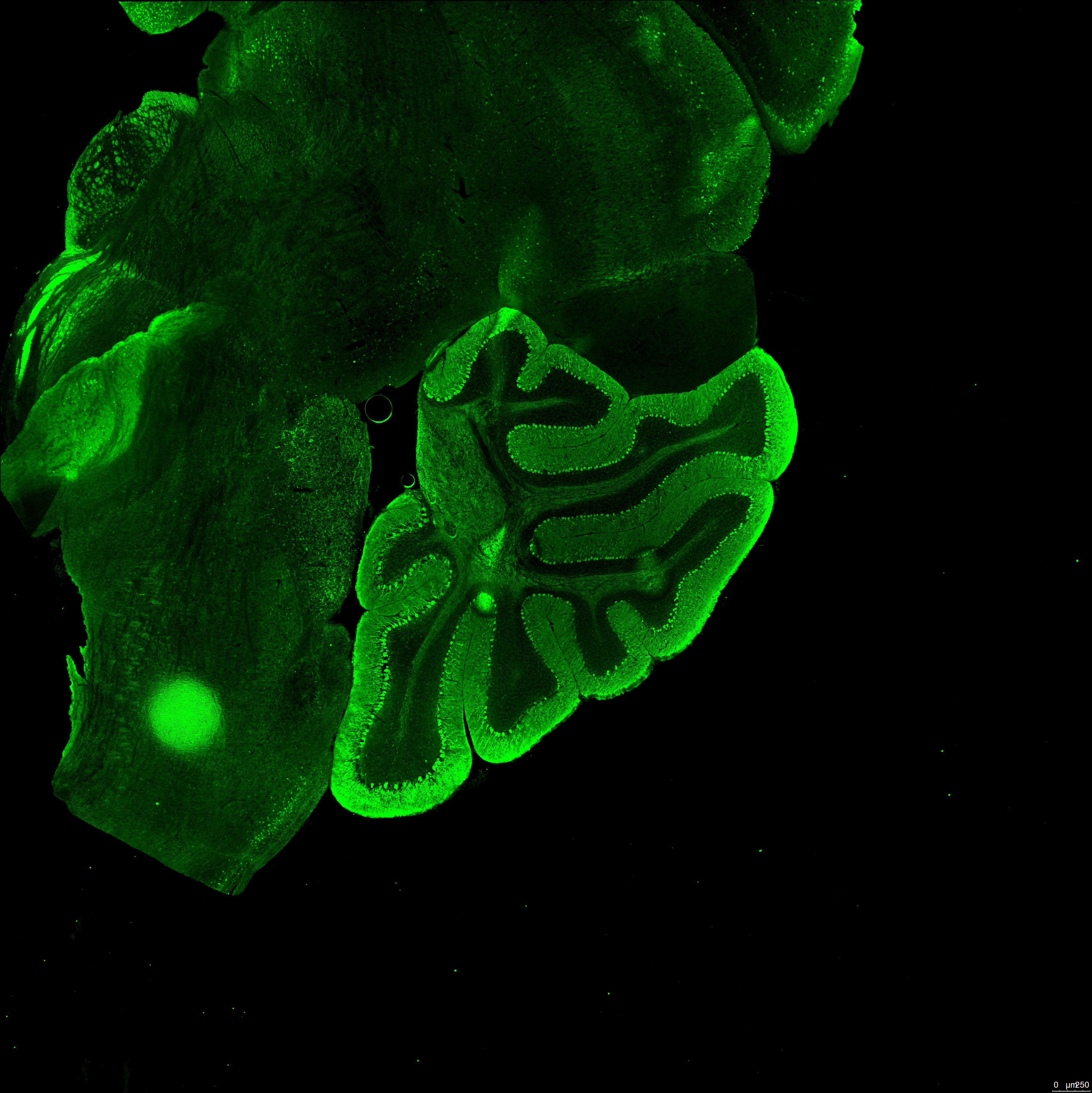

Supplement: Supplementary file 22 — Figure EV9 Source Data [file 44318_2025_654_MOESM22_ESM.zip › EV Figure 9/EV9J/EV9J-1-male 6 month old-WT-Calbindin.tif]

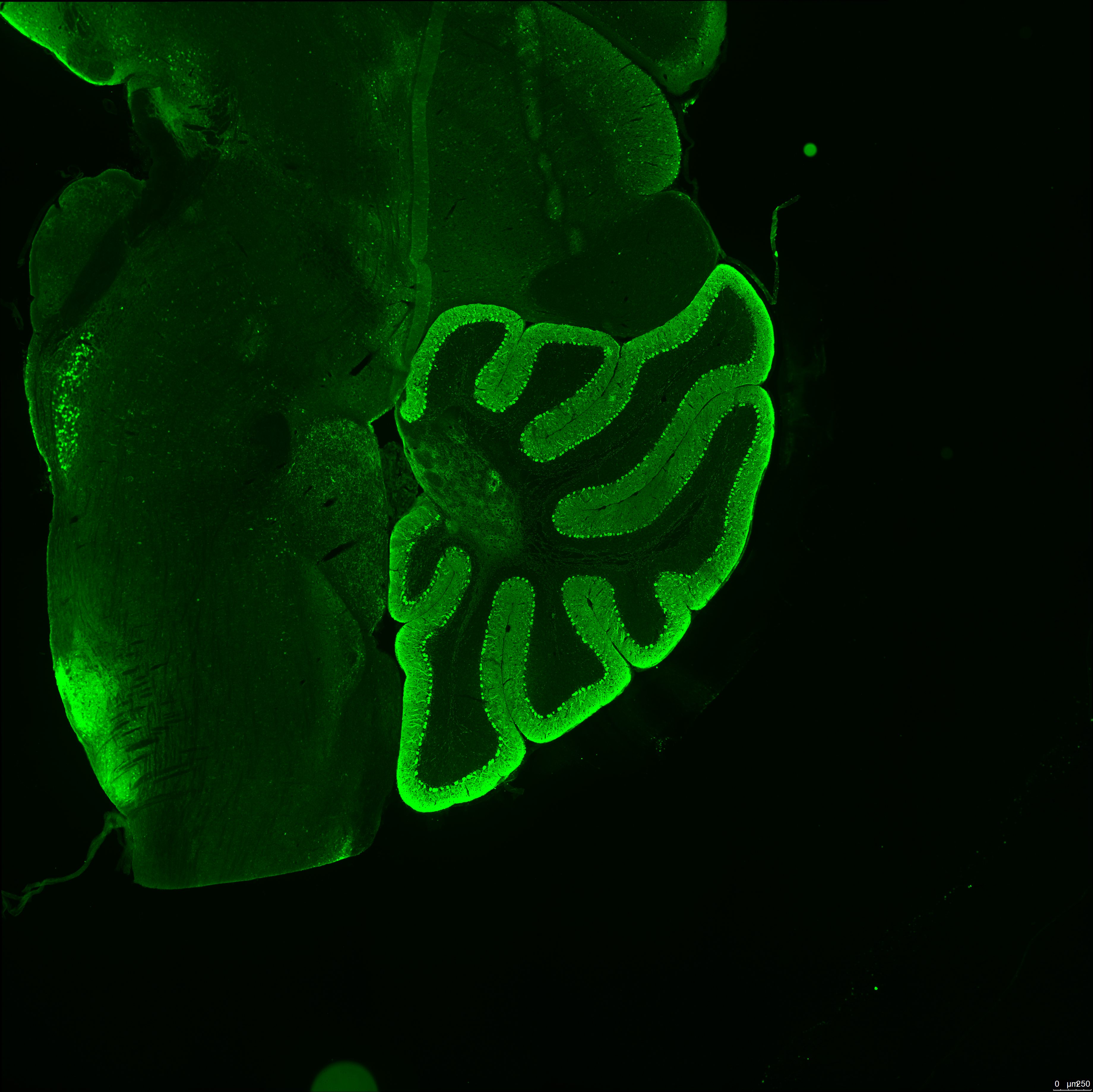

Supplement: Supplementary file 22 — Figure EV9 Source Data [file 44318_2025_654_MOESM22_ESM.zip › EV Figure 9/EV9J/EV9J-2-male 6 month old-KO-Calbindin.tif]

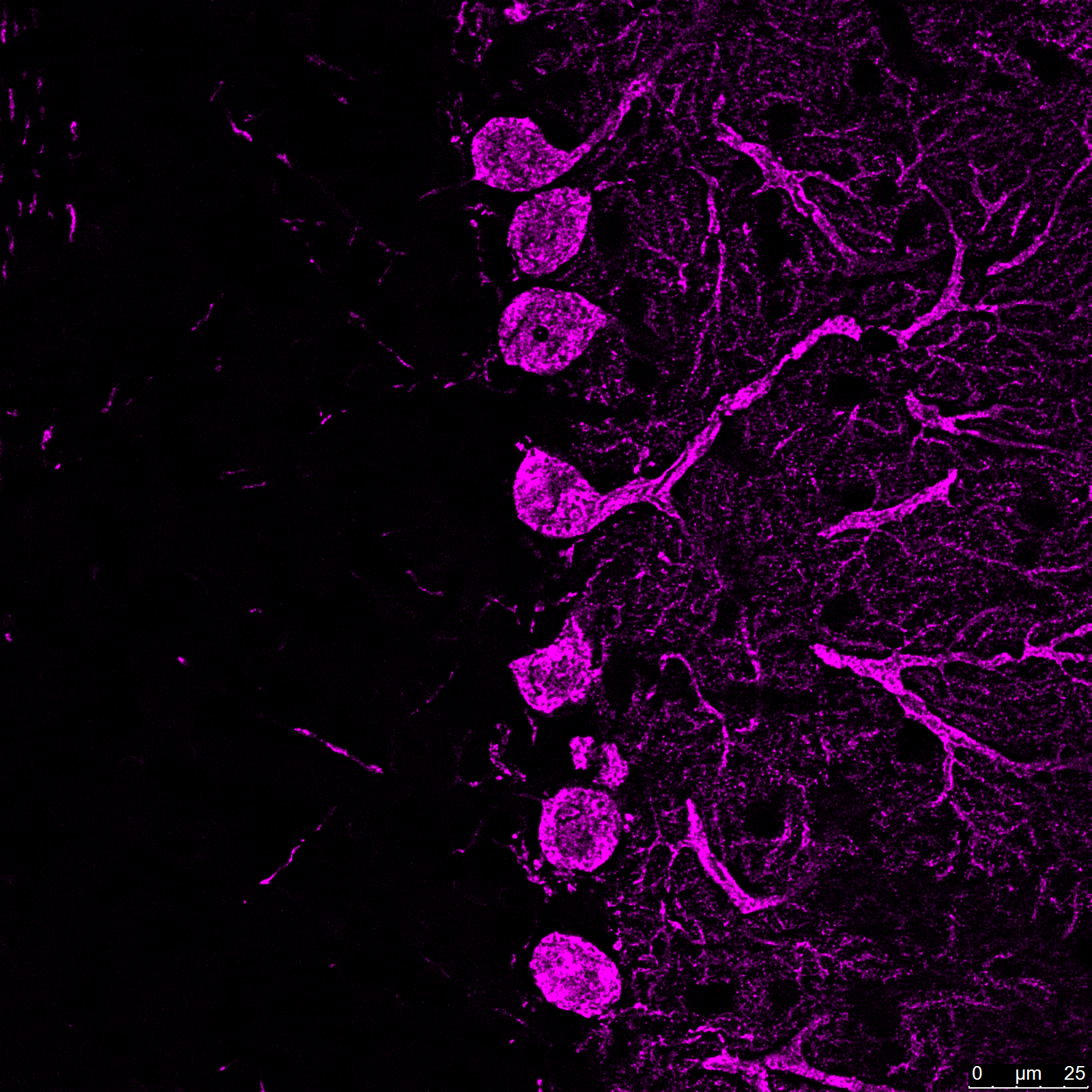

Supplement: Supplementary file 22 — Figure EV9 Source Data [file 44318_2025_654_MOESM22_ESM.zip › EV Figure 9/EV9K/EV9K-1-6 month old male-WT-calbindin.tif]

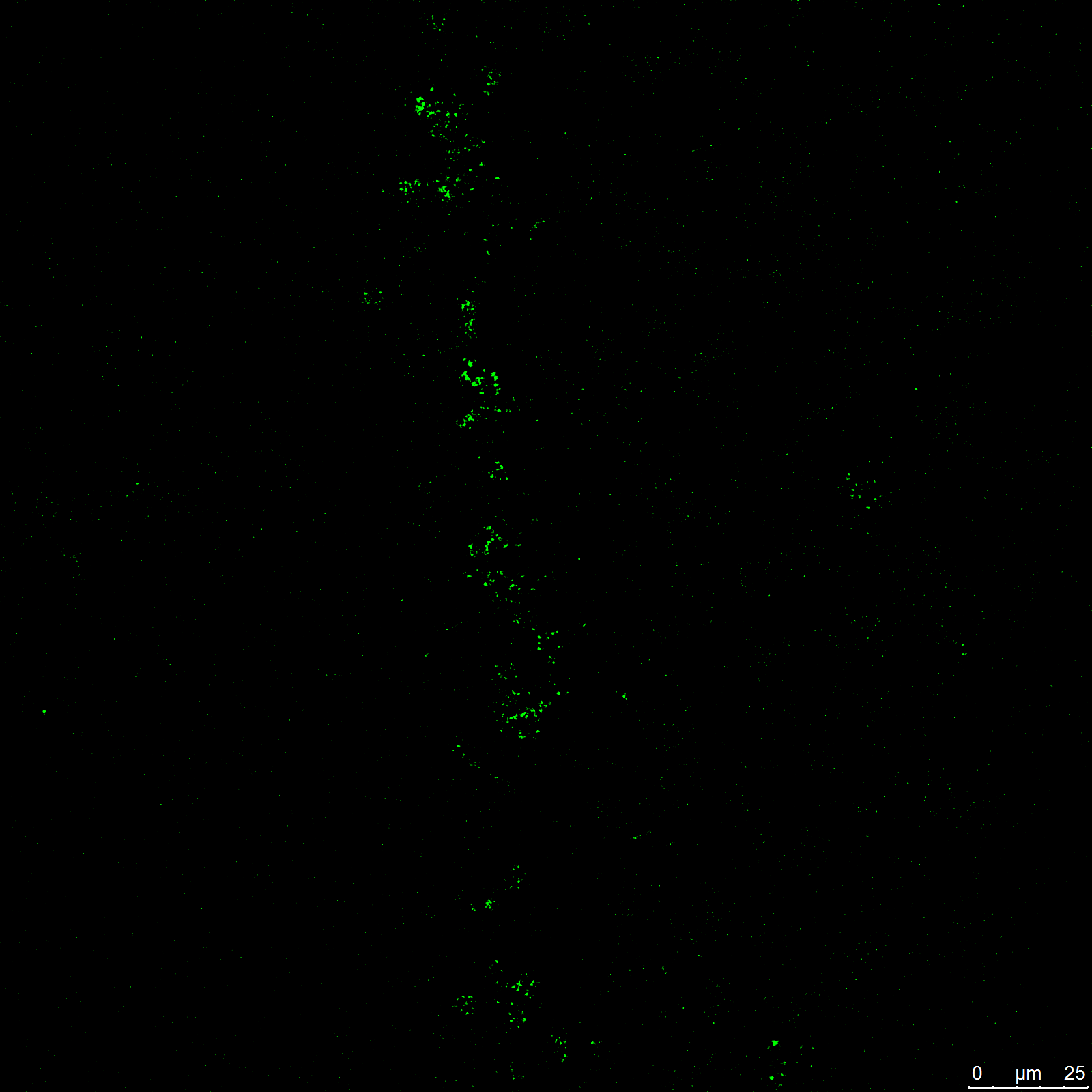

Supplement: Supplementary file 22 — Figure EV9 Source Data [file 44318_2025_654_MOESM22_ESM.zip › EV Figure 9/EV9K/EV9K-6 month old male-KO-lipofuscin.tif]

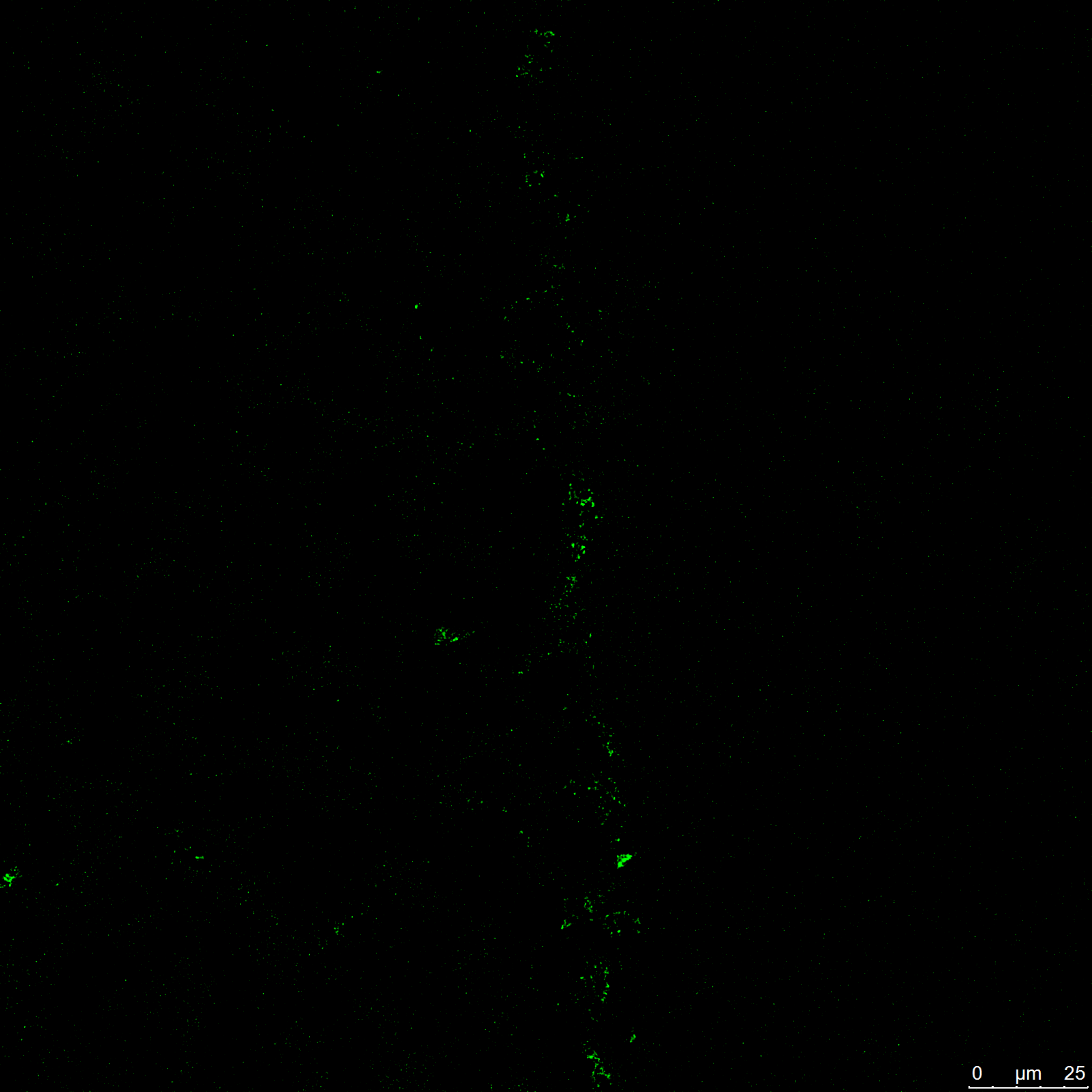

Supplement: Supplementary file 22 — Figure EV9 Source Data [file 44318_2025_654_MOESM22_ESM.zip › EV Figure 9/EV9K/EV9K-1-6 month old male-WT-lipofuscin.tif]
