# Supplementary figures and images for: E3 ligase AREL1 controls perinuclear localization of lysosomes and supports Purkinje cell survival (part 4 of 4)
Source: EMBO J. 2025 Dec 2;45(3):655–91. doi: 10.1038/s44318-025-00654-3 (PMC12864862; doi:10.1038/s44318-025-00654-3)

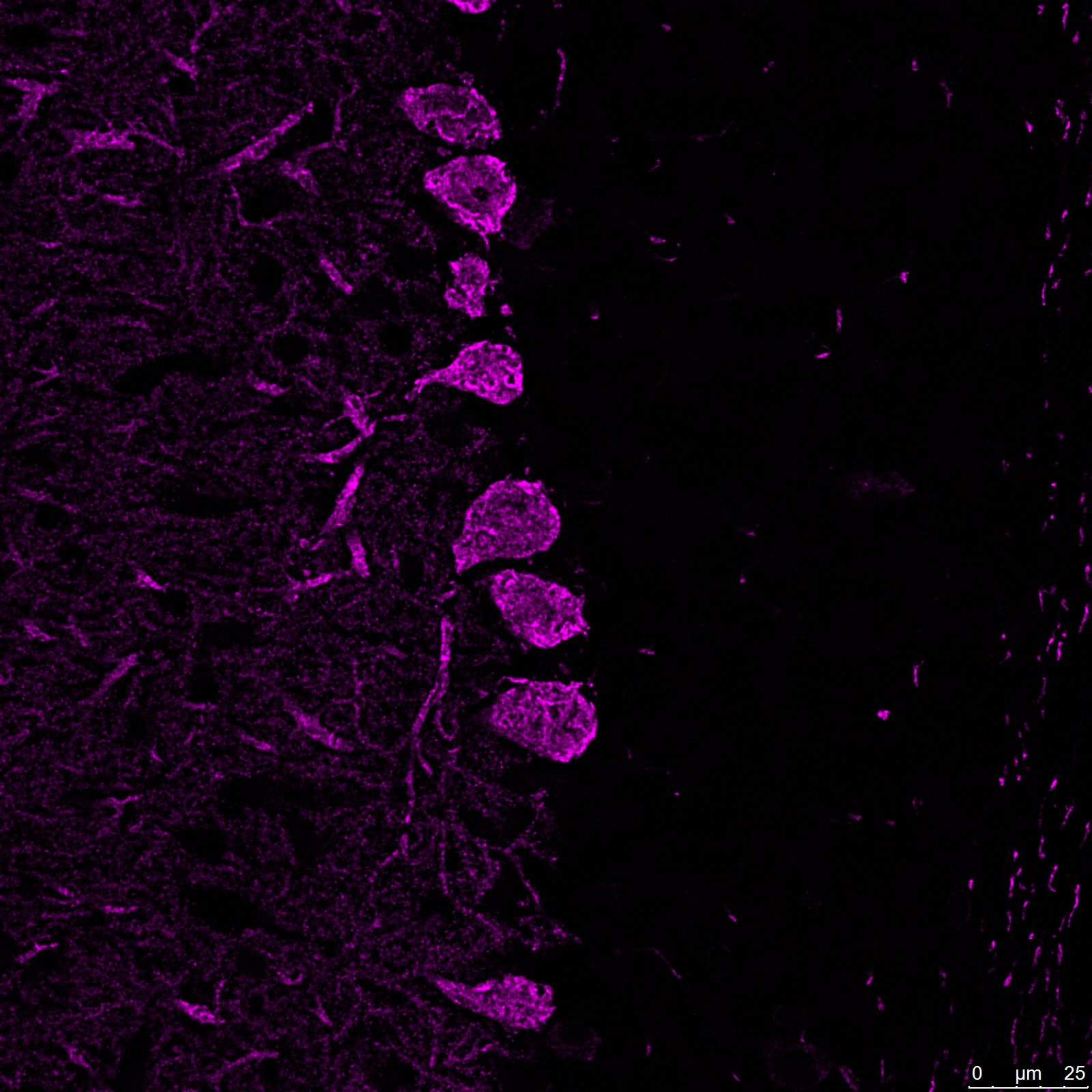

Supplement: Supplementary file 22 — Figure EV9 Source Data [file 44318_2025_654_MOESM22_ESM.zip › EV Figure 9/EV9K/EV9K-2-6 month old male-KO-calbindin.tif]

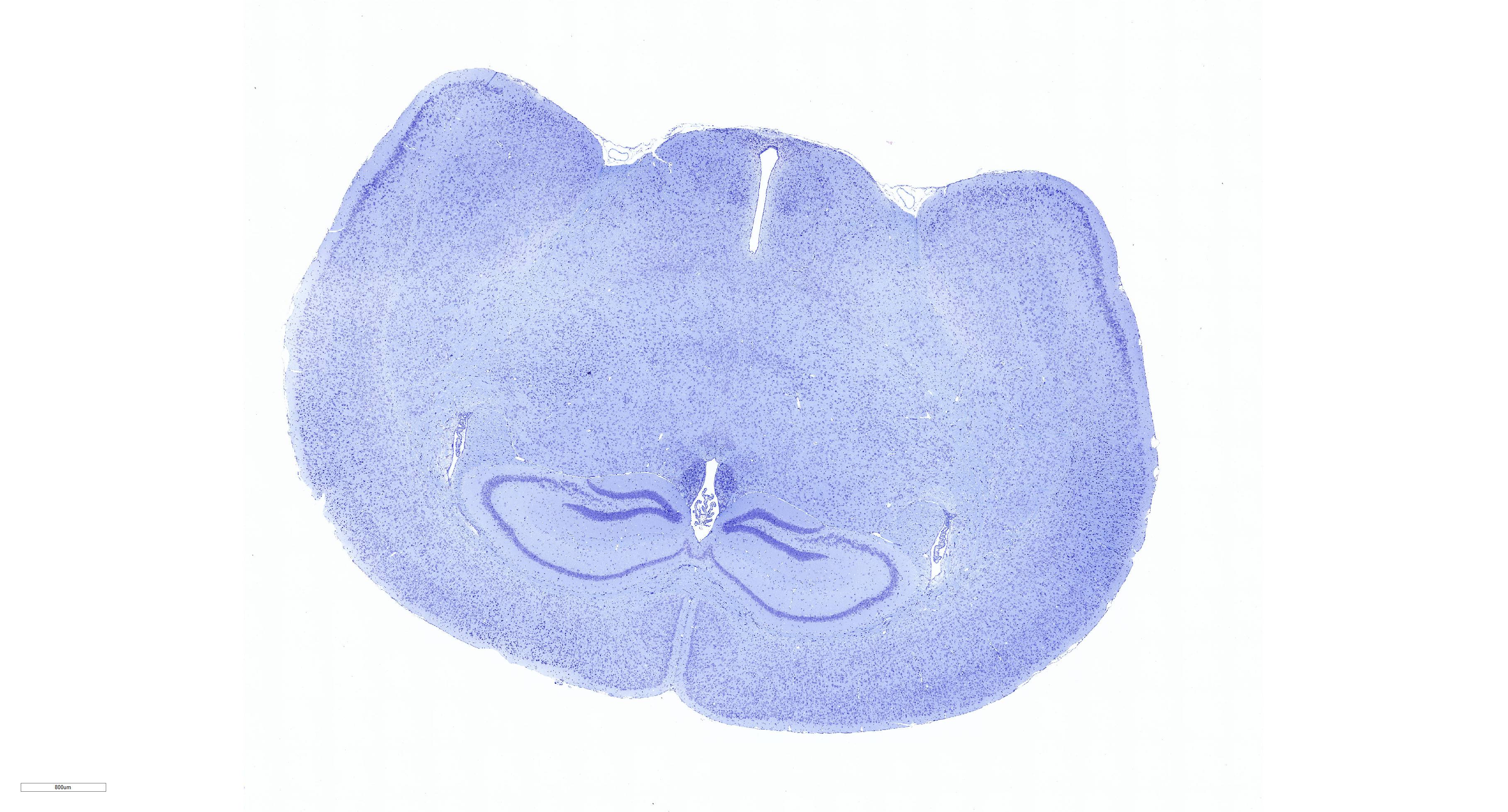

Supplement: Supplementary file 22 — Figure EV9 Source Data [file 44318_2025_654_MOESM22_ESM.zip › EV Figure 9/EV9E/Hippocampus-KO5.jpg]

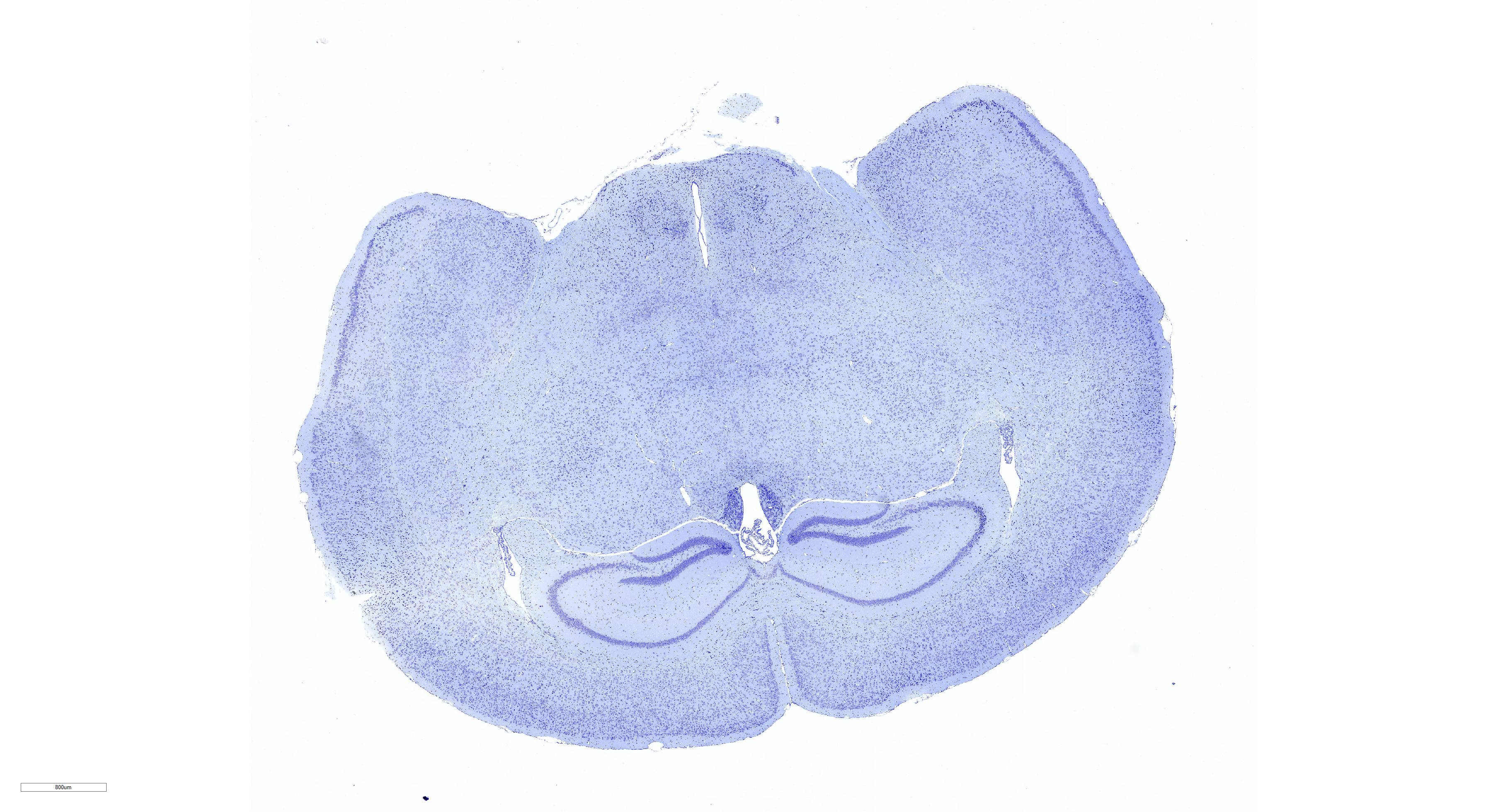

Supplement: Supplementary file 22 — Figure EV9 Source Data [file 44318_2025_654_MOESM22_ESM.zip › EV Figure 9/EV9E/Hippocampus-KO4.jpg]

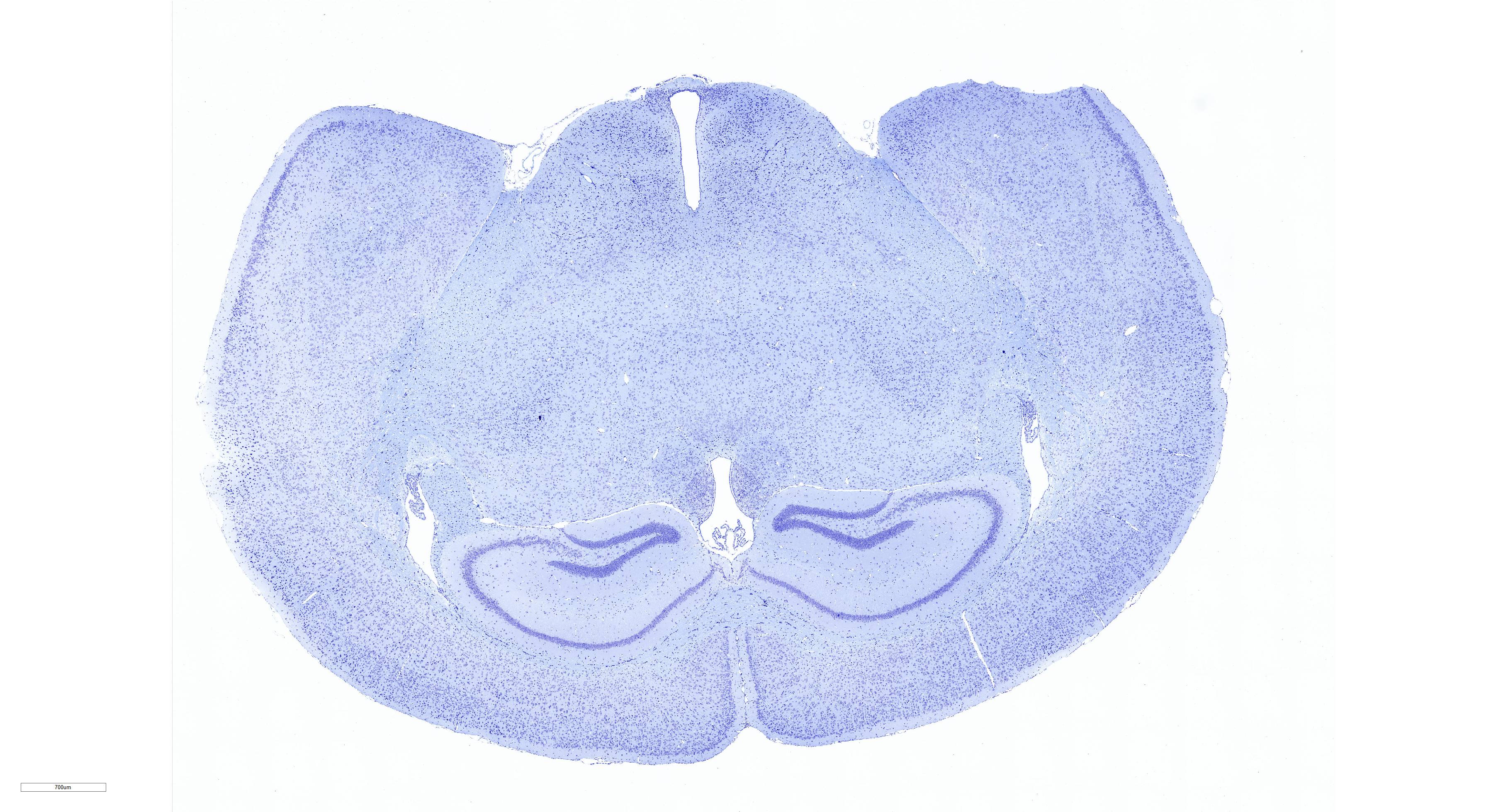

Supplement: Supplementary file 22 — Figure EV9 Source Data [file 44318_2025_654_MOESM22_ESM.zip › EV Figure 9/EV9E/Hippocampus-KO3.jpg]

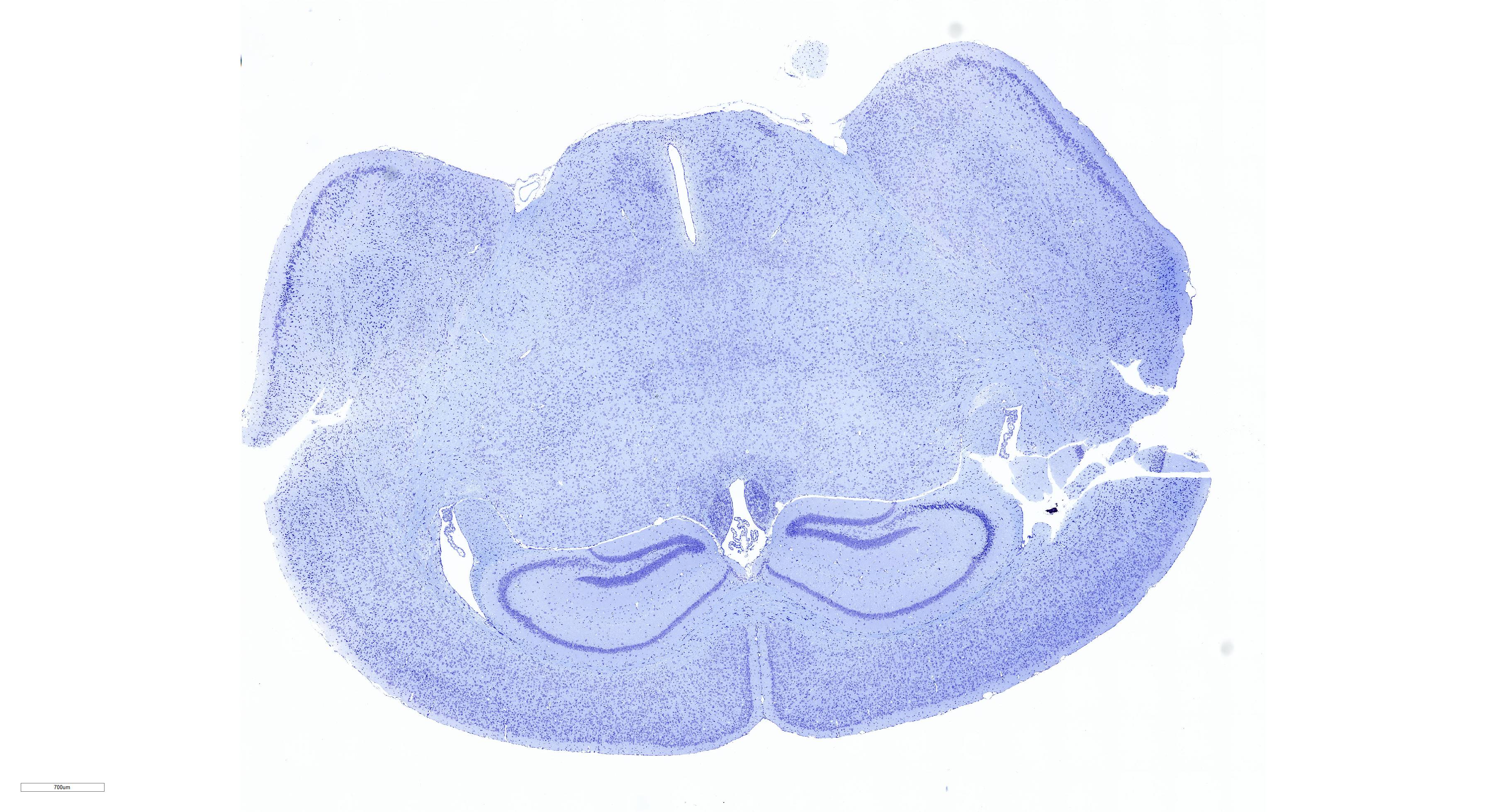

Supplement: Supplementary file 22 — Figure EV9 Source Data [file 44318_2025_654_MOESM22_ESM.zip › EV Figure 9/EV9E/Hippocampus-KO2.jpg]

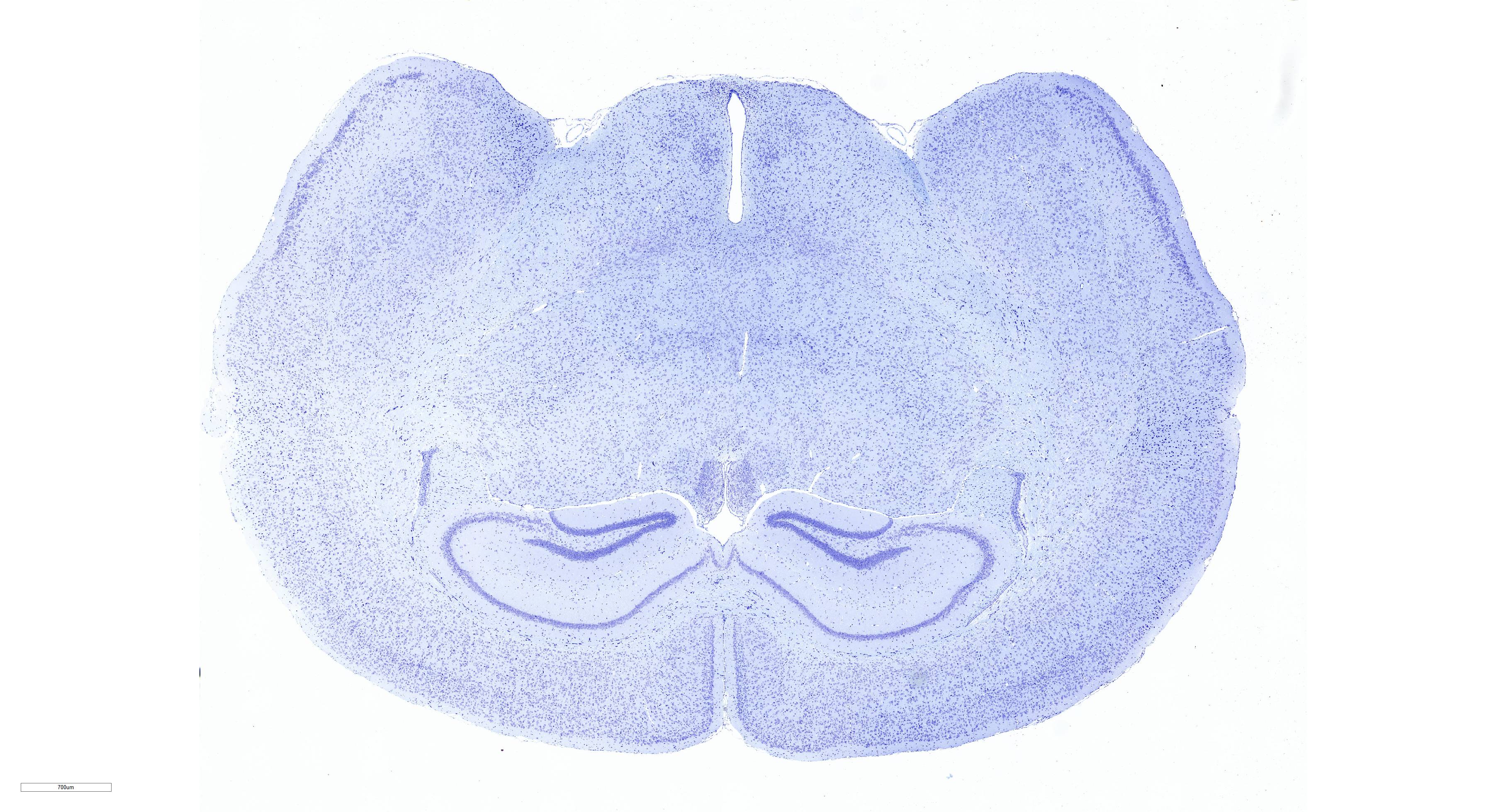

Supplement: Supplementary file 22 — Figure EV9 Source Data [file 44318_2025_654_MOESM22_ESM.zip › EV Figure 9/EV9E/Hippocampus-KO1.jpg]

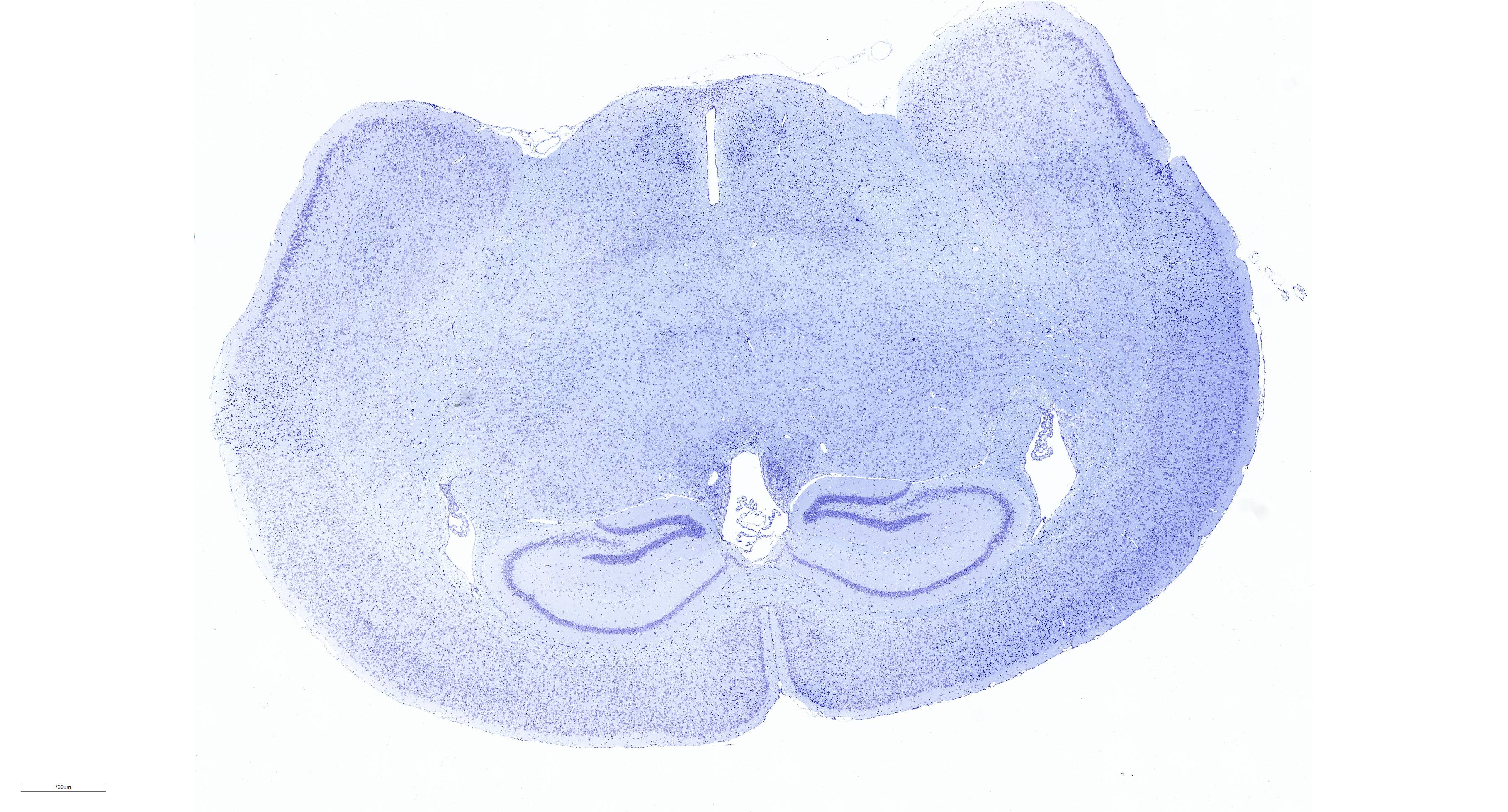

Supplement: Supplementary file 22 — Figure EV9 Source Data [file 44318_2025_654_MOESM22_ESM.zip › EV Figure 9/EV9E/Hippocampus-WT3.jpg]

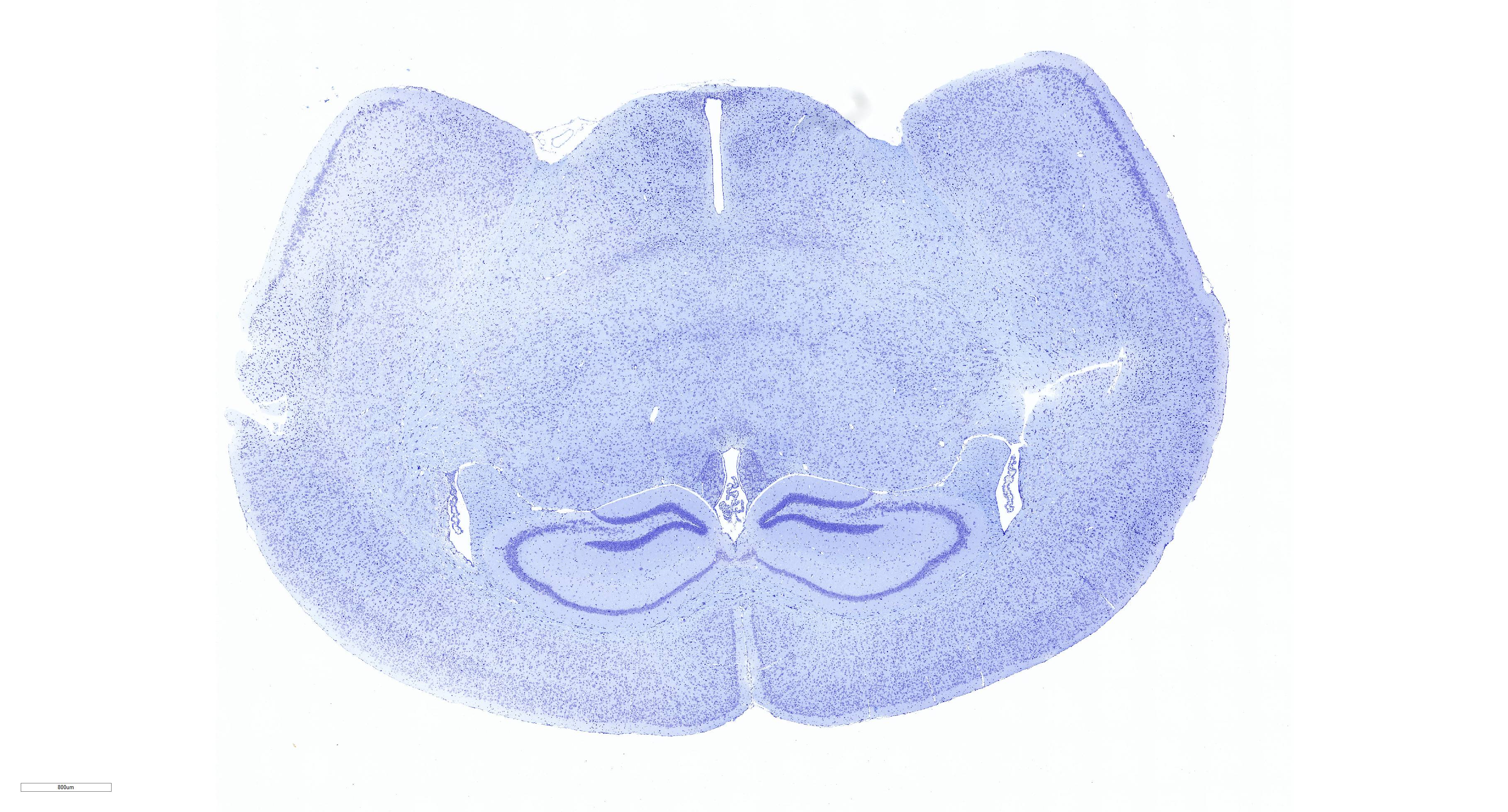

Supplement: Supplementary file 22 — Figure EV9 Source Data [file 44318_2025_654_MOESM22_ESM.zip › EV Figure 9/EV9E/Hippocampus-WT2.jpg]

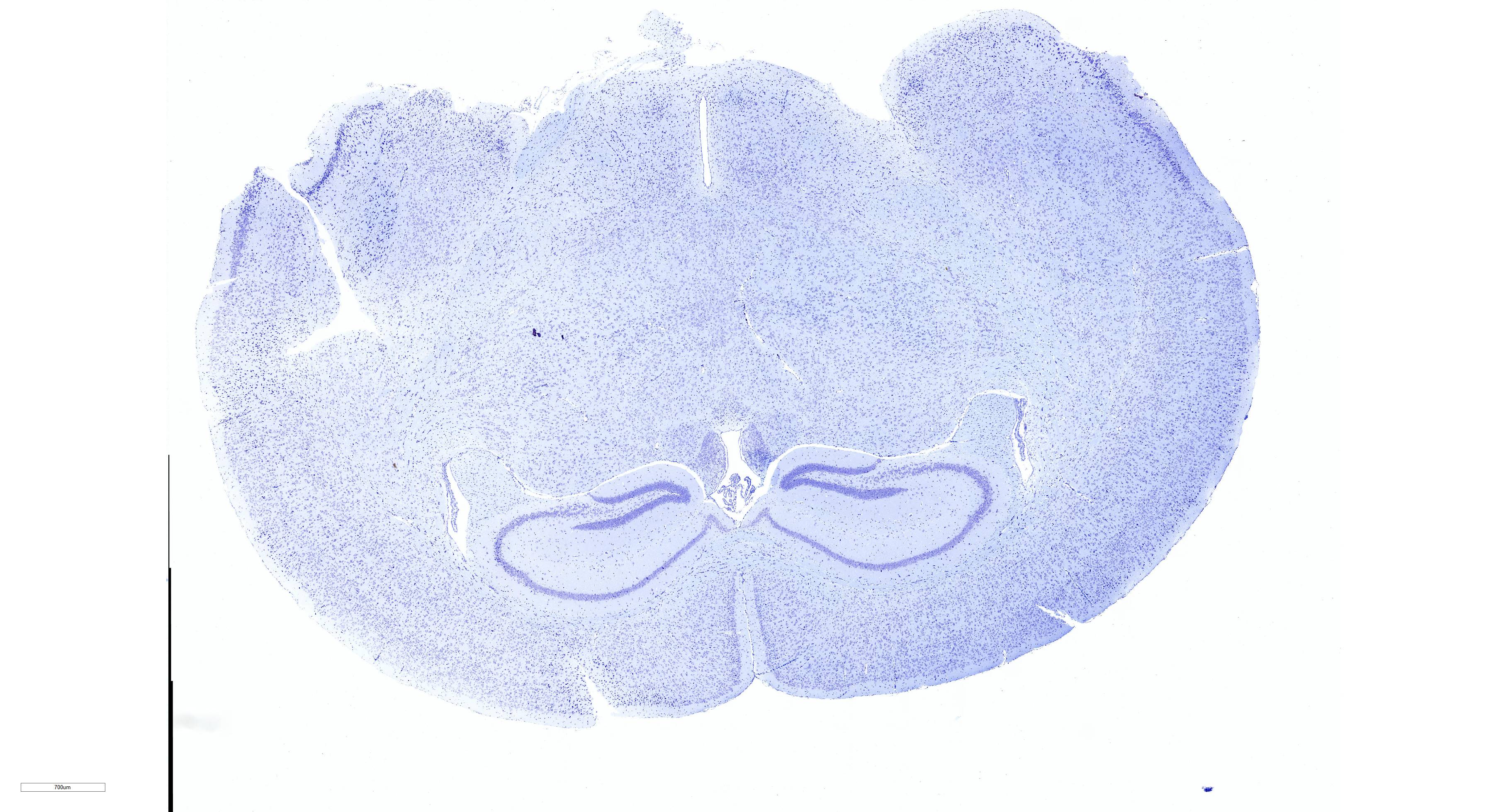

Supplement: Supplementary file 22 — Figure EV9 Source Data [file 44318_2025_654_MOESM22_ESM.zip › EV Figure 9/EV9E/Hippocampus-WT1.jpg]

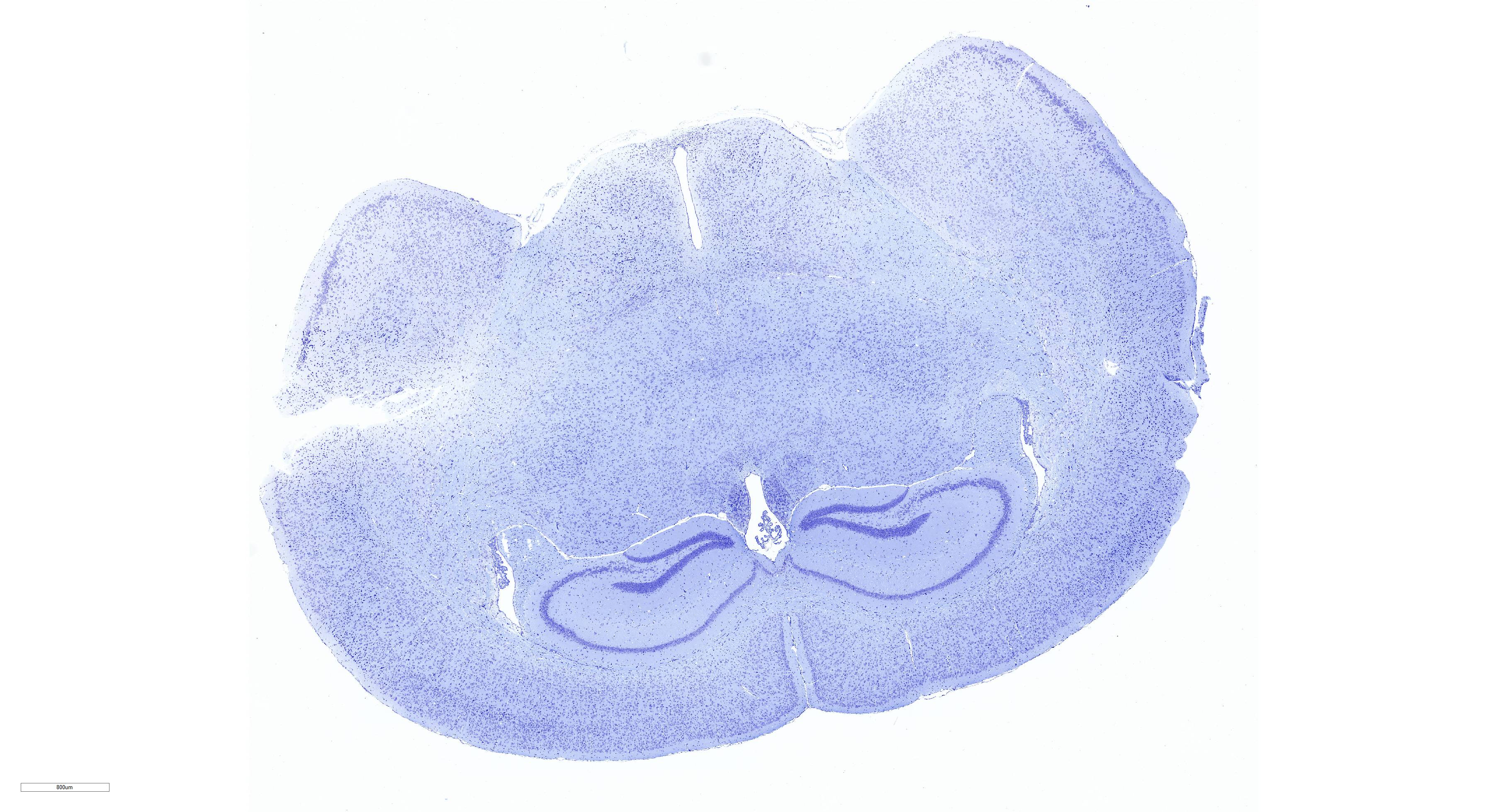

Supplement: Supplementary file 22 — Figure EV9 Source Data [file 44318_2025_654_MOESM22_ESM.zip › EV Figure 9/EV9E/Hippocampus-WT5.jpg]

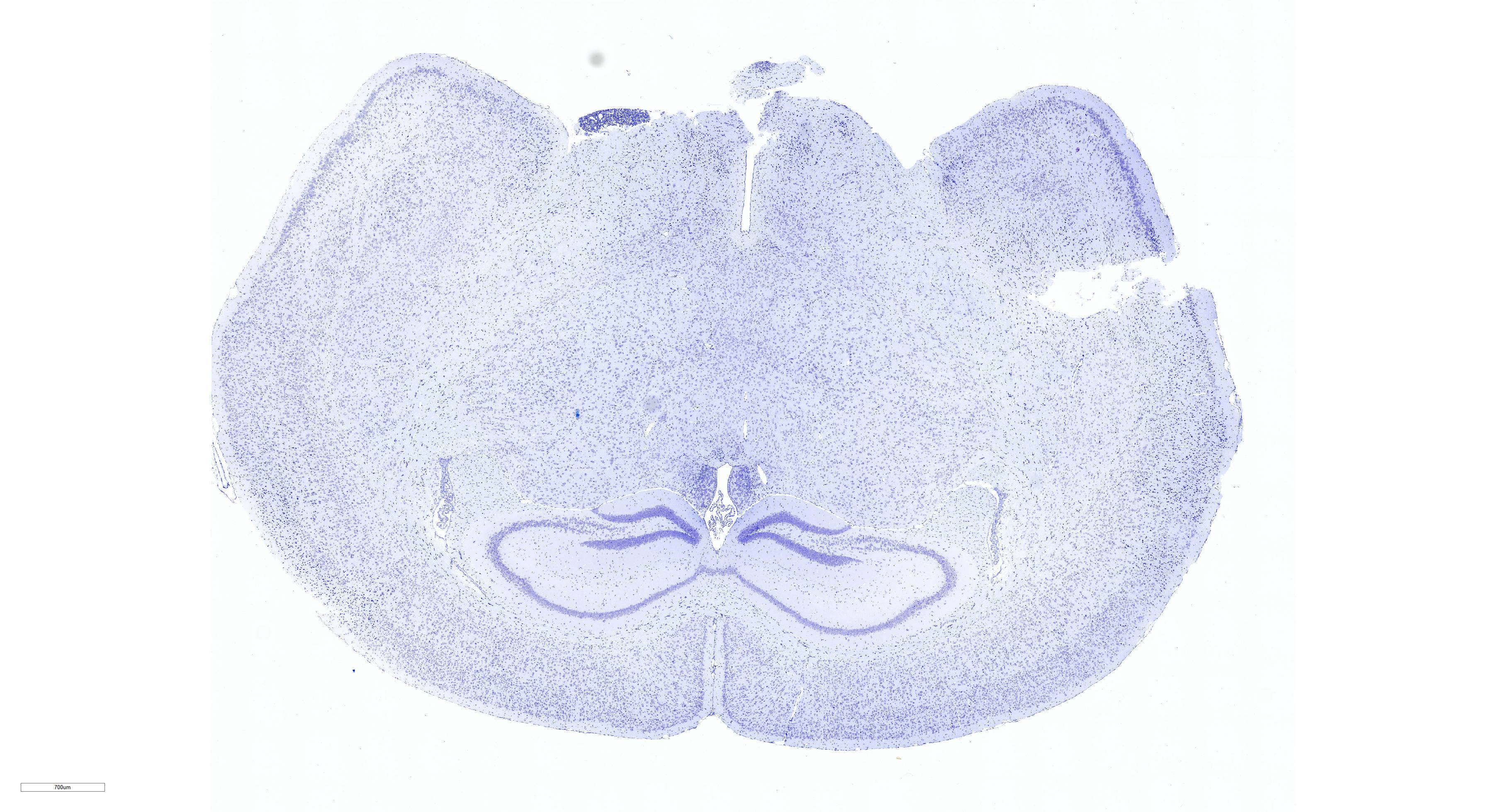

Supplement: Supplementary file 22 — Figure EV9 Source Data [file 44318_2025_654_MOESM22_ESM.zip › EV Figure 9/EV9E/Hippocampus-WT4.jpg]
