# Supplementary material for: E3 ligase AREL1 controls perinuclear localization of lysosomes and supports Purkinje cell survival
Source: EMBO J. 2025 Dec 2;45(3):655–91. doi: 10.1038/s44318-025-00654-3 (PMC12864862; doi:10.1038/s44318-025-00654-3)
Supplement: Supplementary file 23 — Expanded View Figures [file 44318_2025_654_MOESM23_ESM.pdf]

Expanded View Figures

Figure EV1. Identification of proteins at ER–lysosome MCSs using the split-TurboID in proximity labeling assay, related to Fig. 1.

(**A**) Representative confocal images showing U2OS cells transduced with lentiviruses expressing TurboID (C terminus)-EGFP-Sec61β (white) and LAMP1-mCherry-TurboID (N terminus) (green), treated with 100 μM biotin for 4 h and then stained with streptavidin-647 (magenta). Boxed areas are enlarged as numbered and shown on the right. Scale bars, 5 μm (main), 1 μm (inset). (**B**) Workflow of streptavidin affinity purification coupled to mass spectrometry (MS) performed in HEK293T cells. (**C**) Immunoblotting analysis showing biotinylation in post nuclear supernatants (PNS), supernatants (S) and pellets (P) from HEK293T cells transfected as indicated and treated with 100 μM biotin for 4 h. (**D**) Top 18 hits identified at ER–lysosome MCSs. (**E**) Verification of knockdown efficiency of candidates in U2OS cells analyzed by quantitative real-time PCR. Data are presented as mean ± SD ($n = 3$ independent experiments). Unpaired two-tailed Student's *t* test. *P* values from left to right: 0.0002, <0.0001, <0.0001, 0.0002, <0.0001, 0.0001, <0.0001, 0.0009, <0.0001, 0.0001, 0.0002, <0.0001, <0.0001, <0.0001, 0.0003, 0.0004, 0.0002, <0.0001, 0.0003. (**F**) Representative confocal images showing U2OS cells stably expressing shRNAs targeting indicated genes, fixed and immunostained with anti-calnexin and anti-LAMP1 antibodies followed by proximity ligation assay. Scale bars, 10 μm. (**G**) Superplots showing the number of PLA puncta per cell (small dots) and its mean per independent experiment (large dots). Means and error bars (SD) are shown as black bars. # of cells: 21 per condition; from three independent experiments. Unpaired two-tailed Student's *t* test. *P* values from left to right: 0.0057, 0.8821, 0.0119, 0.0006, 0.0038, 0.9349, 0.7540, 0.5507, 0.8070, 0.7957, 0.5766, 0.0211, 0.0125, 0.5876, 0.0177, 0.0071, 0.6837, 0.0155, 0.0146. (**H**) SnapGene images showing that the exon 4 of *AREL1* genome was targeted by sgRNA (top) and Sanger sequencing of *AREL1* knockout (KO) U2OS cells, in which an extra T insertion resulted in premature termination of protein translation (bottom). (**I**) Verification of *AREL1* knockout in U2OS cells by immunoblotting. (**J**) Immunoblotting analysis showing biotinylation in WT and $AREL1^{-/-}$ U2OS cells were transfected as indicated and treated with indicated concentrations of biotin for 4 h. (**K**) Representative confocal images showing PLA signals in WT and $AREL1^{-/-}$ U2OS cells. Cells were fixed and immunostained with anti-calnexin and anti-LAMP1 antibodies followed by proximity ligation assay. Scale bars, 10 μm. (**L**) Superplots showing the number of PLA puncta per cell (small dots) and its mean per independent experiment (large dots). Means and error bars (SD) are shown as black bars. # of cells: WT, 82 and $AREL1^{-/-}$, 76; from 3 independent experiments. Unpaired two-tailed Student's *t* test. WT cells vs $AREL1^{-/-}$ cells, $P < 0.0001$. (**M**) Knockdown efficiency of *AREL1* in HEK293T cells analyzed by quantitative real-time PCR. Data are presented as mean ± SD ($n = 3$ independent experiments). Unpaired two-tailed Student's *t* test. shNC cells vs sh*AREL1* cells, $P = 0.0015$. (**N**) Representative confocal images showing PLA signals in shNC and sh*AREL1* HEK293T cells. Cells were fixed and immunostained with anti-calnexin and anti-LAMP1 antibodies followed by proximity ligation assay. Scale bars, 10 μm. (**O**) Superplots showing the number of PLA puncta per cell (small dots) and its mean per independent experiment (large dots). Means and error bars (SD) are shown as black bars. # of cells: shNC, 60 and sh*AREL1*, 60; from 3 independent experiments. Unpaired two-tailed Student's *t* test. shNC cells vs sh*AREL1* cells, $P < 0.0001$ Source data are available online for this figure.

 © The Author(s)

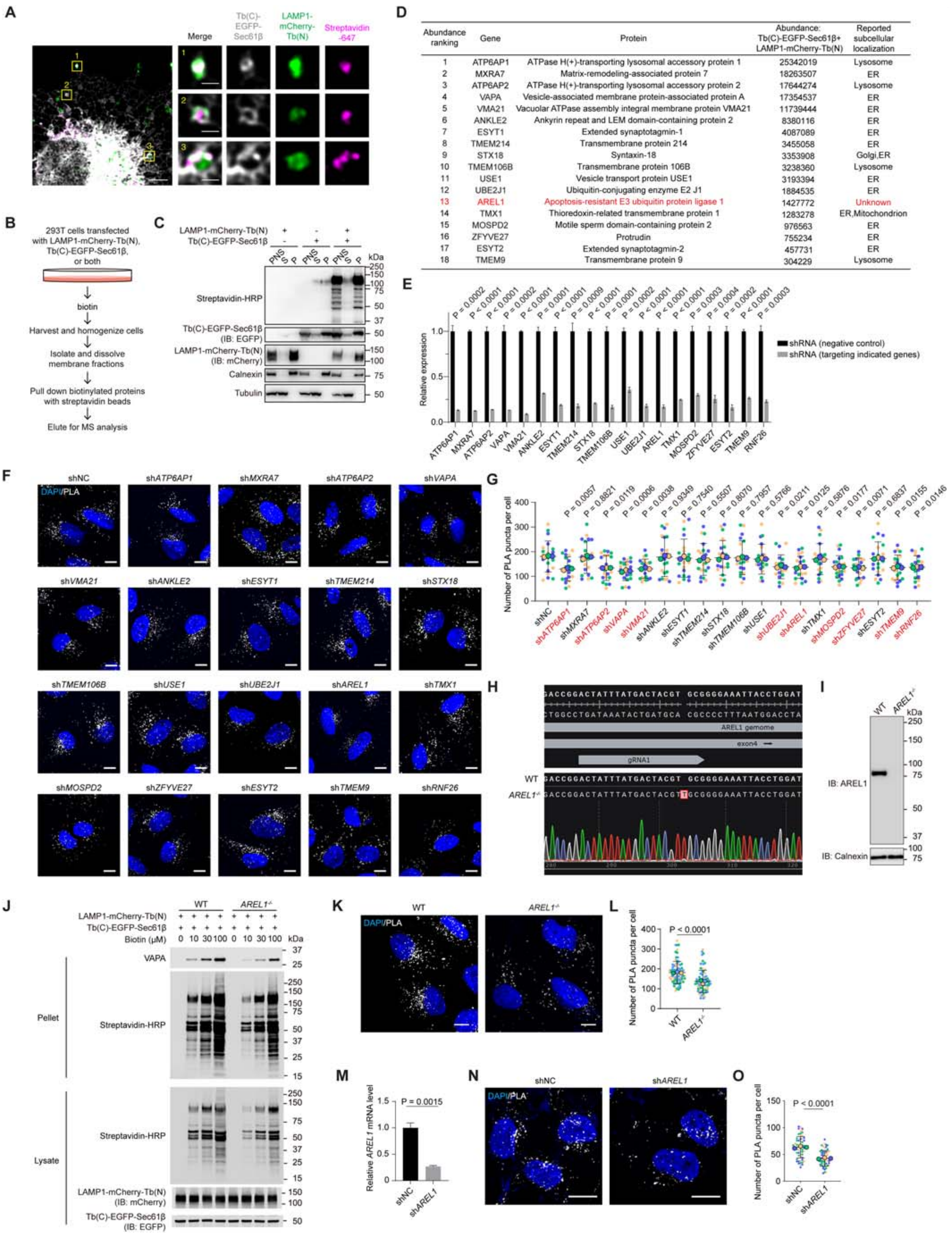

Abundance ranking	Gene	Protein	Abundance: Tb(C)-EGFP-Sec61β+ LAMP1-mCherry-Tb(N)	Reported subcellular localization
1	ATP6AP1	ATPase H(+)-transporting lysosomal accessory protein 1	25342019	Lysosome
2	MXRA7	Matrix-remodeling-associated protein 7	18263507	ER
3	ATP6AP2	ATPase H(+)-transporting lysosomal accessory protein 2	17644274	Lysosome
4	VAPA	Vesicle-associated membrane protein-associated protein A	17354537	ER
5	VMA21	Vacuolar ATPase assembly integral membrane protein VMA21	11739444	ER
6	ANKLE2	Ankyrin repeat and LEM domain-containing protein 2	8380116	ER
7	ESYT1	Extended synaptotagmin-1	4087089	ER
8	TMEM214	Transmembrane protein 214	3455058	ER
9	STX18	Syntaxin-18	3353908	Golgi,ER
10	TMEM106B	Transmembrane protein 106B	3238360	Lysosome
11	USE1	Vesicle transport protein USE1	3193394	ER
12	UBE2J1	Ubiquitin-conjugating enzyme E2 J1	1884535	ER
13	AREL1	Apoptosis-resistant E3 ubiquitin protein ligase 1	1427772	Unknown
14	TMX1	Thioredoxin-related transmembrane protein 1	1283278	ER,Mitochondrion
15	MOSPD2	Motile sperm domain-containing protein 2	976563	ER
16	ZFYVE27	Protrudin	755234	ER
17	ESYT2	Extended synaptotagmin-2	457731	ER
18	TMEM9	Transmembrane protein 9	304229	Lysosome

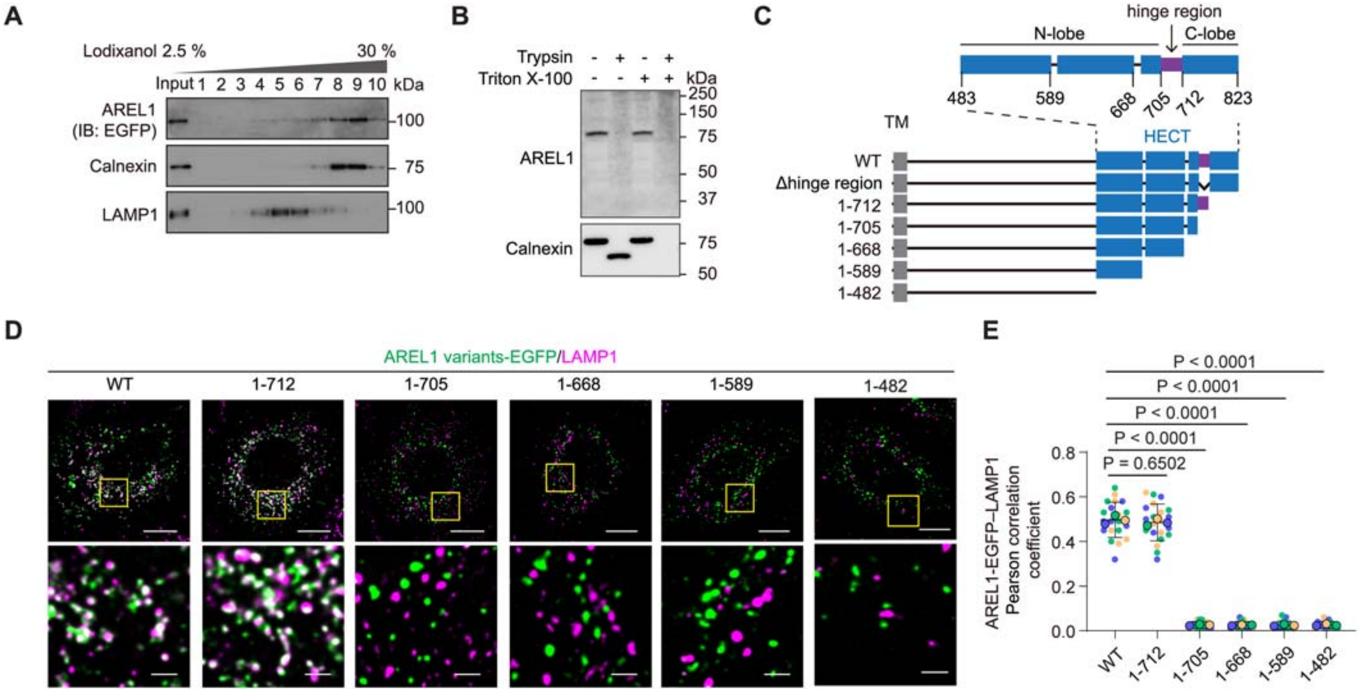

Figure EV2. AREL1 is an ER protein and interacts with lysosomes via the hinge region, related to Fig. 1.

(**A**) Immunoblotting analysis of membrane fractions isolated from U2OS cells transfected with the plasmid expressing AREL1-EGFP and subjected to iodixanol density gradient centrifugation. (**B**) Immunoblotting analysis of U2OS cell lysates treated with (+) or without (−) 0.1 µg/ µl trypsin or 0.1% Triton X-100. (**C**) Schematic illustration of AREL1 variants. (**D**) Representative confocal images showing the localization of AREL1 variants relative to lysosomes. U2OS cells were transduced with lentiviruses expressing indicated AREL1 variants tagged with EGFP and immunostained with anti-LAMP1 antibody. Boxed areas are enlarged and shown on the bottom. Scale bars, 10 µm (main), 2 µm (inset). (**E**) Superplots showing Pearson's correlation coefficient for AREL1 variants and LAMP1 per cell (small dots) and its mean per independent experiment (large dots). Means and error bars (SD) are shown as black bars. # of cells: 21 for cells expressing AREL1(WT)-EGFP, AREL1(1-712)-EGFP, AREL1(1-705)-EGFP, AREL1(1-668)-EGFP, AREL1(1-589)-EGFP and AREL1(1–482)-EGFP, respectively; from 3 independent experiments. Unpaired two-tailed Student's *t* test. AREL1(WT)-EGFP vs AREL1(1-712)-EGFP, $P = 0.6502$; AREL1(WT)-EGFP vs AREL1(1-705)-EGFP, $P < 0.0001$; AREL1(WT)-EGFP vs AREL1(1-668)-EGFP, $P < 0.0001$; AREL1(WT)-EGFP vs AREL1(1-589)-EGFP, $P < 0.0001$; AREL1(WT)-EGFP vs AREL1(1–482)-EGFP, $P < 0.0001$ Source data are available online for this figure.

 © The Author(s)

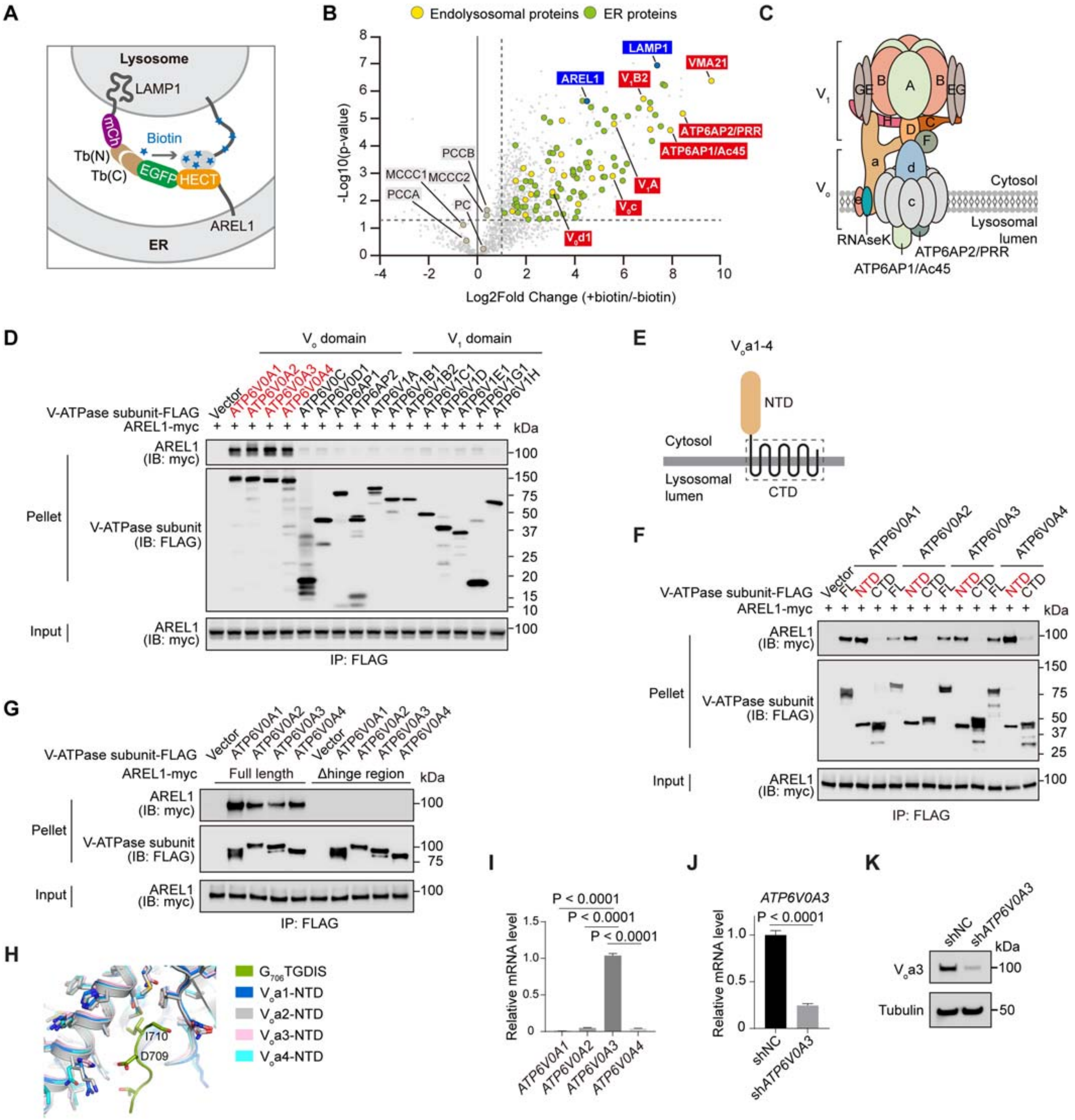
A
Lysosome
LAMP1
mCh
Biotin
Tb(N)
Tb(C)
EGFP
HECT
AREL1
ER
B
Endolysosomal proteins
ER proteins
-Log10(p-value)
Log2Fold Change (+biotin/-biotin)
LAMP1
VMA21
AREL1
ATP6AP2/PRR
ATP6AP1/Ac45
PCCB
MCCC1
MCCC2
PCCA
PC
C
Cytosol
Lysosomal lumen
RNAseK
ATP6AP2/PRR
ATP6AP1/Ac45
D
V-ATPase subunit-FLAG
AREL1-myc
Vector
ATP6V0A1
ATP6V0A2
ATP6V0A3
ATP6V0A4
ATP6V0C
ATP6V0D1
ATP6AP1
ATP6AP2
ATP6V1A
ATP6V1B1
ATP6V1B2
ATP6V1C1
ATP6V1D
ATP6V1E1
ATP6V1G1
ATP6V1H
kDa
AREL1 (IB: myc)
Pellet
V-ATPase subunit (IB: FLAG)
Input
IP: FLAG
E
NTD
Cytosol
Lysosomal lumen
CTD
F
Vector
FL
NTD
CTD
IP: FLAG
G
Full length
Δhinge region
H
I710
D709
I
Relative mRNA level
P < 0.0001
J
ATP6V0A3
shNC
shATP6V0A3
K
Tubulin

Figure EV3. The hinge region of AREL1 interacts with the amino-terminal domain of V_oa subunit of V-ATPase, related to Fig. 1.

(**A**) Schematic illustration of the split-TurboID-based proximity labeling assay to identify AREL1 binding partners. Tb(N), TurboID (N terminus); Tb(C), TurboID (C terminus); mCh, mCherry. (**B**) Volcano plot showing proteins enriched at AREL1-mediated ER–lysosome membrane contact sites. U2OS cells expressing LAMP1-mCherry-TurboID (N terminus) and TurboID (C terminus)-EGFP-AREL1 were treated with (+) or without (−) 100 μM biotin for 4 h. Biotinylated membrane proteins were enriched and subjected to mass spectrometry analysis. PC (Pyruvate carboxylase), PCCA (Propionyl-CoA carboxylase subunit a), PCCB (Propionyl-CoA carboxylase subunit b), MCCC1 (Methylcrotonoyl-CoA carboxylase subunit a) and MCCC2 (Methylcrotonoyl-CoA carboxylase beta chain) were served as the internal references for normalization. Four independent experiments were performed. A two-sample *t* test was conducted to calculate *p*-values using the implemented function in Perseus. Significantly enriched (Log_2(Fold Change) > 1 and $-Log_{10}$(*P* value) >1.3) endolysosomal proteins and ER proteins are in yellow and green dots, respectively, with each specific V-ATPase subunit highlighted. AREL1 and LAMP1 as the baits are displayed as blue dots. (**C**) Schematic illustration of the mammalian V-ATPase. (**D**) Co-immunoprecipitation (IP) analysis of HEK293T cells transfected as indicated. (**E**) Schematic illustration of human V_oa isoforms 1–4. NTD, N-terminal domain; CTD, C-terminal domain. (**F**, **G**) Co-IP analysis of HEK293T cells transfected as indicated. (**H**) AlphaFold 3-predicted interactions between the hinge region of AREL1 (G_{706}TGDIS) and the NTDs of four V_oa isoforms. The NTDs of V_oa1-4 are in marine, gray, lightpink, cyan, respectively, and G_{706}TGDIS is in splitpea. (**I**) Relative expression levels of indicated *ATP6V0A* in U2OS cells analyzed by quantitative real-time PCR. Data are presented as mean ± SD ($n = 6$ independent experiments). Unpaired two-tailed Student's *t* test. *ATP6V0A1* vs *ATP6V0A3*, $P < 0.0001$; *ATP6V0A2* vs *ATP6V0A3*, $P < 0.0001$; *ATP6V0A4* vs *ATP6V0A3*, $P < 0.0001$. (**J**) Verification of knockdown efficiency of *ATP6V0A3* in U2OS cells analyzed by quantitative real-time PCR. Data are presented as mean ± SD ($n = 3$ independent experiments). Unpaired two-tailed Student's *t* test. shNC cells vs sh*ATP6V0A3* cells, $P < 0.0001$. (**K**) Verification of *ATP6V0A3* knockdown in U2OS cells by immunoblotting Source data are available online for this figure.

 © The Author(s)

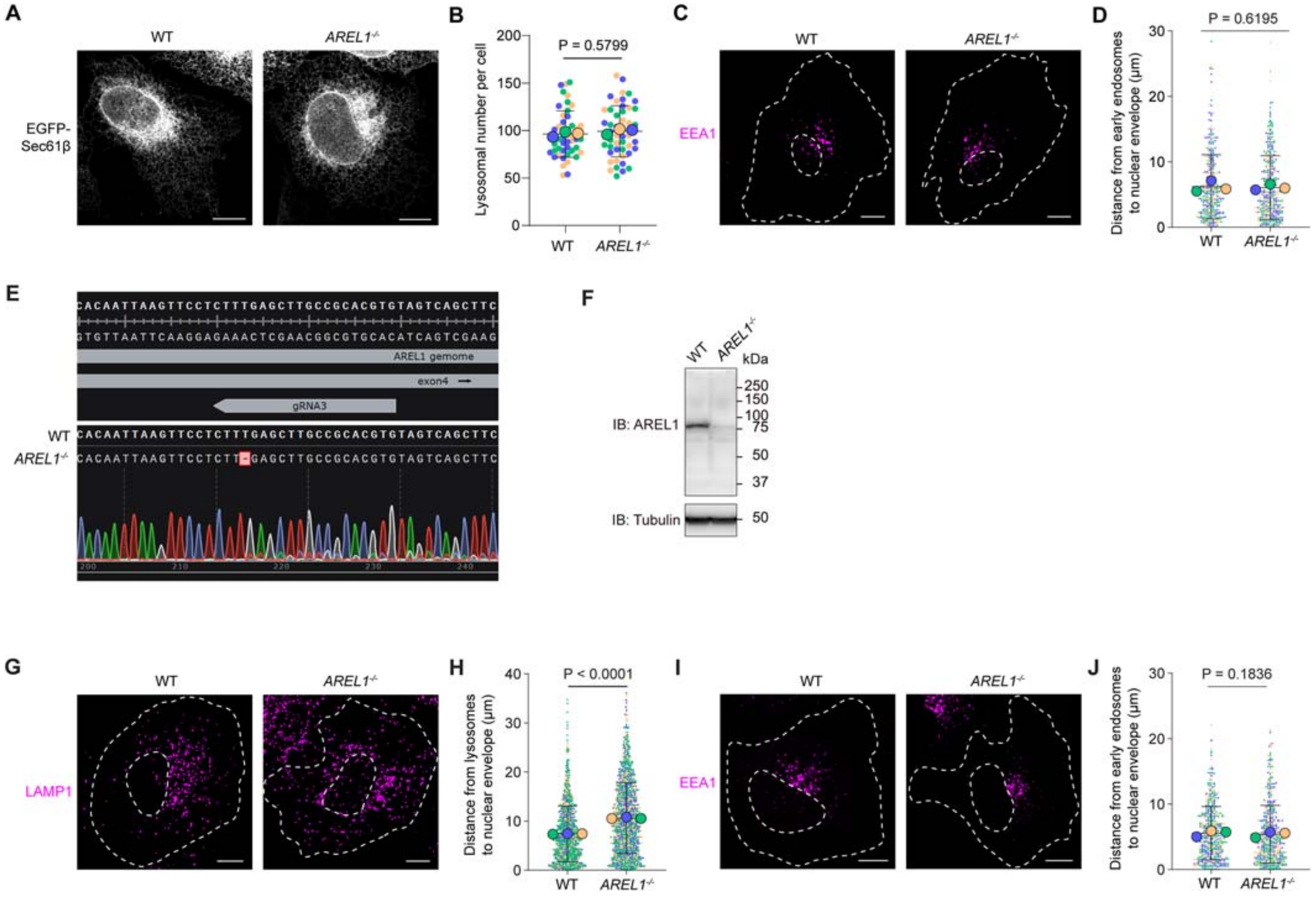

Figure EV4. The effects of AREL1 deficiency on ER morphology, lysosomal numbers, as well as the distribution of lysosomes and early endosomes, related to Fig. 2.

(**A**) Representative confocal images showing ER distribution in WT and *AREL1*$^{-/-}$ U2OS cells. Scale bars, 10 µm. (**B**) Superplots showing the number of lysosomes per cell (small dots) and its mean per independent experiment (large dots). Means and error bars (SD) are shown as black bars. # of cells: WT, 60 and *AREL1*$^{-/-}$, 60; from 3 independent experiments. Unpaired two-tailed Student's *t* test. WT cells vs *AREL1*$^{-/-}$ cells, $P = 0.5799$. (**C**) Representative confocal images showing WT and *AREL1*$^{-/-}$ U2OS cells immunostained with anti-EEA1 antibody. Cell contour and nucleus are outlined using white dashed lines. Scale bars, 10 µm. (**D**) Superplots showing the distance from early endosomes to the nuclear envelope (small dots) and its mean per independent experiment (large dots). Means and error bars (SD) are shown as black bars. # of cells (# of early endosomes): WT, 9 (369) and *AREL1*$^{-/-}$, 9 (410); from 3 independent experiments. Mann–Whitney *U* test. WT cells vs *AREL1*$^{-/-}$ cells, $P = 0.6195$. (**E**) SnapGene images showing that the exon 4 of *AREL1* genome was targeted by sgRNA (top) and Sanger sequencing of *AREL1* knockout (KO) HeLa cells, in which a T deletion resulted in premature termination of protein translation (bottom). (**F**) Verification of *AREL1* knockout in HeLa cells by immunoblotting. (**G**) Representative confocal images showing WT and *AREL1*$^{-/-}$ HeLa cells immunostained with anti-LAMP1 antibody. Cell contour and nucleus are outlined using white dashed lines. Scale bars, 10 µm. (**H**) Superplots showing the distance from lysosomes to the nuclear envelope (small dots) and its mean per independent experiment (large dots). Means and error bars (SD) are shown as black bars. # of cells (# of lysosomes): WT, 11 (1353) and *AREL1*$^{-/-}$, 11 (1566); from 3 independent experiments. Mann–Whitney *U* test. WT cells vs *AREL1*$^{-/-}$ cells, $P < 0.0001$. (**I**) Representative confocal images showing WT and *AREL1*$^{-/-}$ HeLa cells immunostained with anti-EEA1 antibody. Cell contour and nucleus are outlined using white dashed lines. Scale bars, 10 µm. (**J**) Superplots showing the distance from early endosomes to the nuclear envelope (small dots) and its mean per independent experiment (large dots). Means and error bars (SD) are shown as black bars. # of cells (# of early endosomes): WT, 9 (531) and *AREL1*$^{-/-}$, 9 (528); from 3 independent experiments. Mann–Whitney *U* test. WT cells vs *AREL1*$^{-/-}$ cells, $P = 0.1836$ Source data are available online for this figure.

© The Author(s)

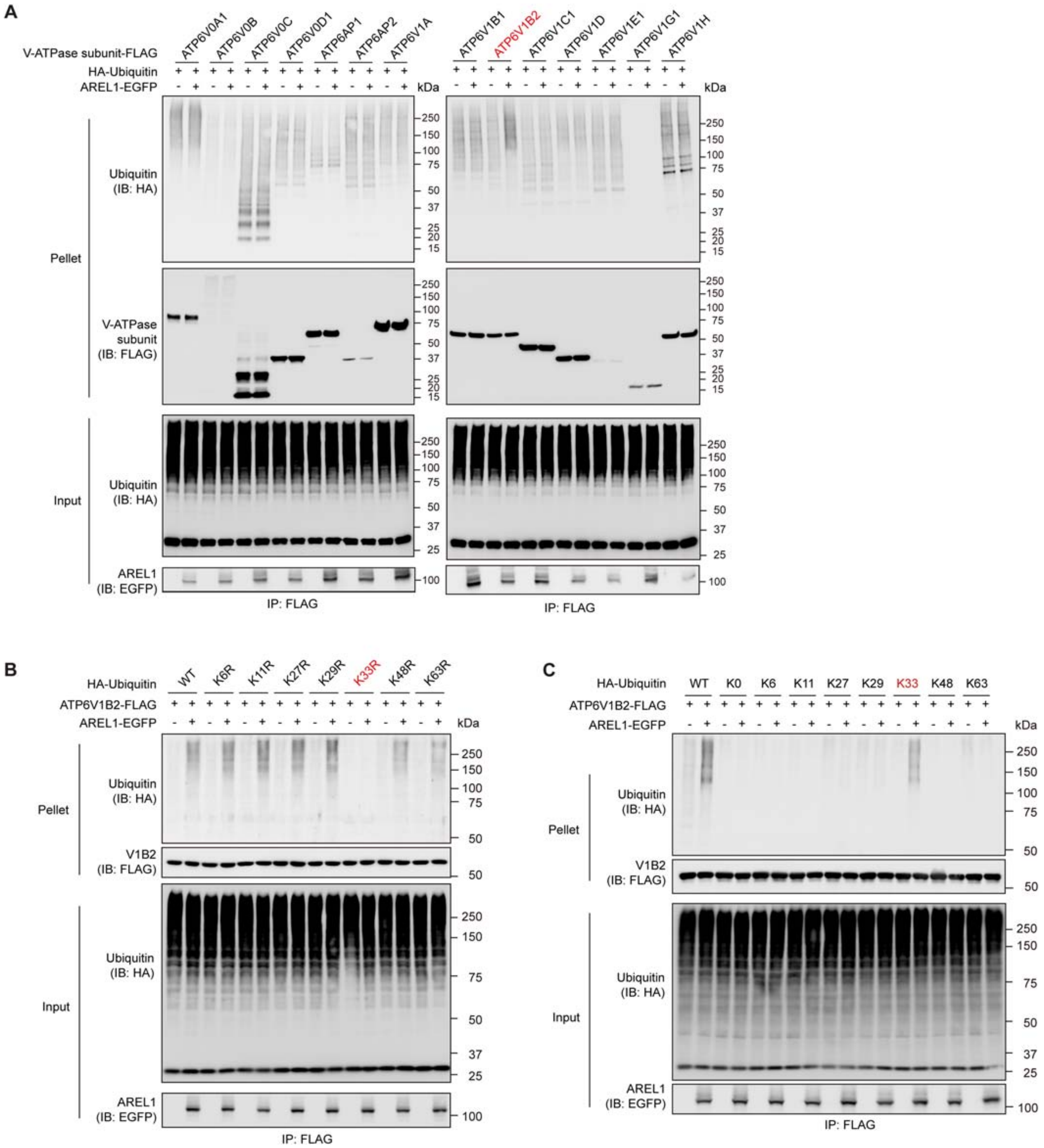

Figure EV5. AREL1 selectively catalyzes K33-linked ubiquitylation of the V_1B2 subunit of the V-ATPase, related to Fig. 4.

(**A–C**) HEK293T cells were transfected as indicated and subjected to IP with anti-FLAG beads followed by immunoblotting to analyze ubiquitylation Source data are available online for this figure.

 © The Author(s)

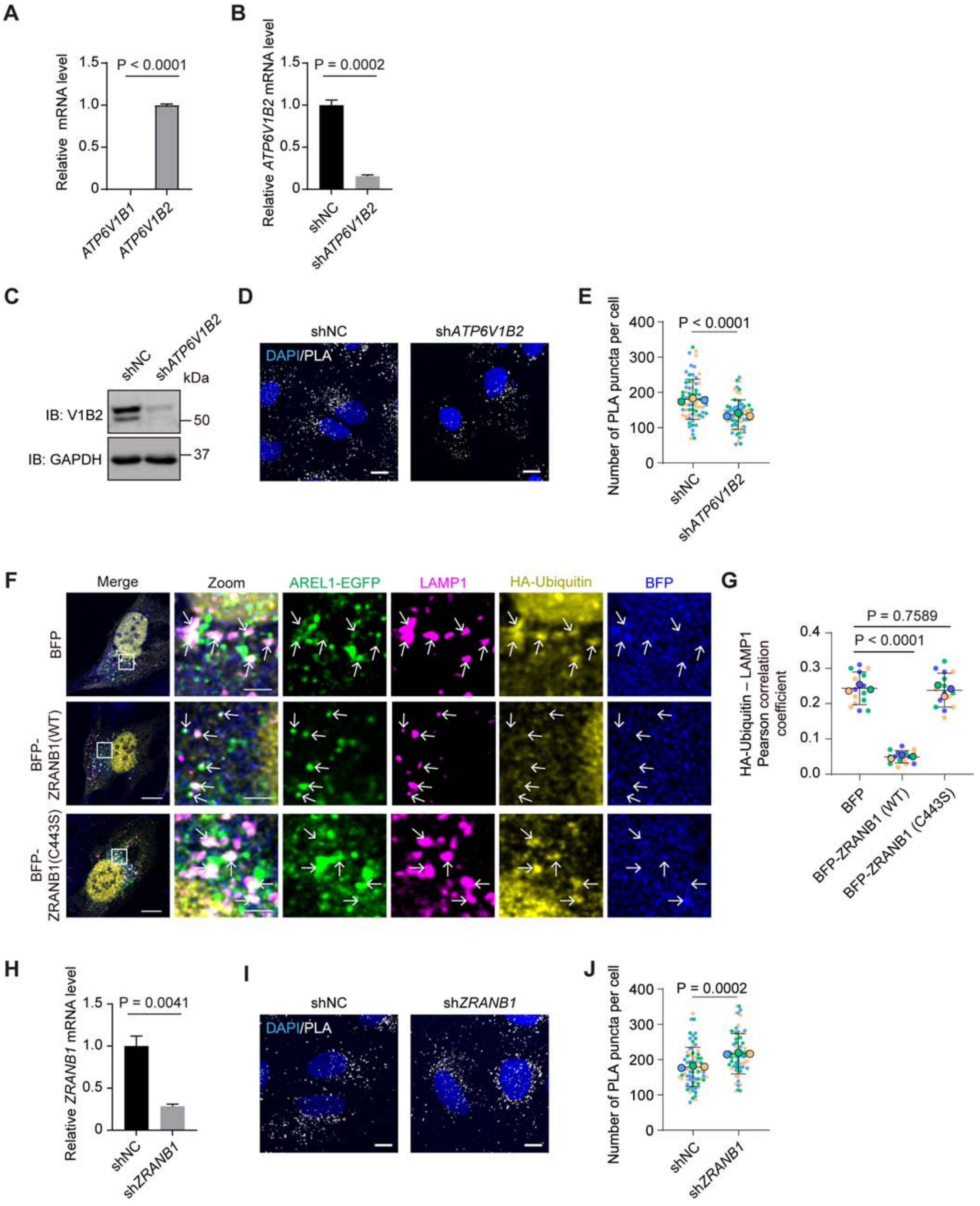
A
Relative mRNA level
P < 0.0001
ATP6V1B1
ATP6V1B2
B
Relative ATP6V1B2 mRNA level
P = 0.0002
shNC
shATP6V1B2
C
shNC
shATP6V1B2
kDa
IB: V1B2
50
IB: GAPDH
37
D
shNC
shATP6V1B2
DAPI/PLA
E
Number of PLA puncta per cell
P < 0.0001
shNC
shATP6V1B2
F
Merge
Zoom
AREL1-EGFP
LAMP1
HA-Ubiquitin
BFP
BFP
BFP-ZRANB1(WT)
BFP-ZRANB1(C443S)
G
HA-Ubiquitin – LAMP1 Pearson correlation coefficient
P = 0.7589
P < 0.0001
BFP
BFP-ZRANB1 (WT)
BFP-ZRANB1 (C443S)
H
Relative ZRANB1 mRNA level
P = 0.0041
shNC
shZRANB1
I
shNC
shZRANB1
DAPI/PLA
J
Number of PLA puncta per cell
P = 0.0002
shNC
shZRANB1

Figure EV6. Characterization of the effects of *ATP6V1B2* and *ZRANB1* deficiency on lysosomal contacts with the ER and those of ZRANB1 overexpression on lysosomal ubiquitin signals, related to Fig. 4.

(**A**) Relative expression levels of *ATP6V1B1* and *ATP6V1B2* in U2OS cells analyzed by quantitative real-time PCR. Data are presented as mean ± SD ($n = 3$ independent experiments). Unpaired two-tailed Student's *t* test. *ATP6V1B1* vs *ATP6V1B2*, $P < 0.0001$. (**B**) Verification of knockdown efficiency of *ATP6V1B2* in U2OS cells analyzed by quantitative real-time PCR. Data are presented as mean ± SD ($n = 3$ independent experiments). Unpaired two-tailed Student's *t* test. shNC cells vs sh*ATP6V1B2* cells, $P = 0.0002$. (**C**) Verification of *ATP6V1B2* knockdown in U2OS cells by immunoblotting. (**D**) Representative confocal images showing PLA signals in shNC and sh*ATP6V1B2* U2OS cells. Cells were fixed and immunostained with anti-calnexin and anti-LAMP1 antibodies followed by proximity ligation assay. Scale bars, 10 μm. (**E**) Superplots showing the number of PLA puncta per cell (small dots) and its mean per independent experiment (large dots). Means and error bars (SD) are shown as black bars. # of cells: shNC, 71 and sh*ATP6V1B2*, 69; from 3 independent experiments. Unpaired two-tailed Student's *t* test. shNC cells vs sh*ATP6V1B2* cells, $P < 0.0001$. (**F**) Representative confocal images showing U2OS cells transduced with lentiviruses expressing AREL1-EGFP, HA-tagged ubiquitin, together with those expressing blue fluorescent protein (BFP) alone or with BFP-tagged ZRANB1 variants, followed by immunostaining with anti-LAMP1 and anti-HA tag antibodies. Boxed areas are enlarged on the right. White arrows indicate contacts between AREL1-EGFP and LAMP1. Scale bars, 10 μm. (**G**) Superplots showing Pearson's correlation coefficient for HA-ubiquitin and LAMP1 per cell (small dots) and its mean per independent experiment (large dots). Means and error bars (SD) are shown as black bars. # of cells: 15 for U2OS cells expressing BFP, BFP-ZRANB1(WT), and BFP-ZRANB1(C443S), respectively; from 3 independent experiments. Unpaired two-tailed Student's *t* test. Cells expressing BFP vs cells expressing BFP-ZRANB1(WT), $P < 0.0001$; Cells expressing BFP vs cells expressing BFP-ZRANB1(C443S), $P = 0.7589$. (**H**) Verification of knockdown efficiency of *ZRANB1* in U2OS cells analyzed by quantitative real-time PCR. Data are presented as mean ± SD ($n = 3$ independent experiments). Unpaired two-tailed Student's *t* test. shNC cells vs sh*ZRANB1* cells, $P = 0.0041$. (**I**) Representative confocal images showing PLA signals in shNC and sh*ZRANB1* U2OS cells. Cells were fixed and immunostained with anti-calnexin and anti-LAMP1 antibodies followed by proximity ligation assay. Scale bars, 10 μm. (**J**) Superplots showing the number of PLA puncta per cell (small dots) and its mean per independent experiment (large dots). Means and error bars (SD) are shown as black bars. # of cells: shNC, 73 and sh*ZRANB1*, 74; from 3 independent experiments. Unpaired two-tailed Student's *t* test. shNC cells vs sh*ZRANB1* cells, $P = 0.0002$ Source data are available online for this figure.

 © The Author(s)

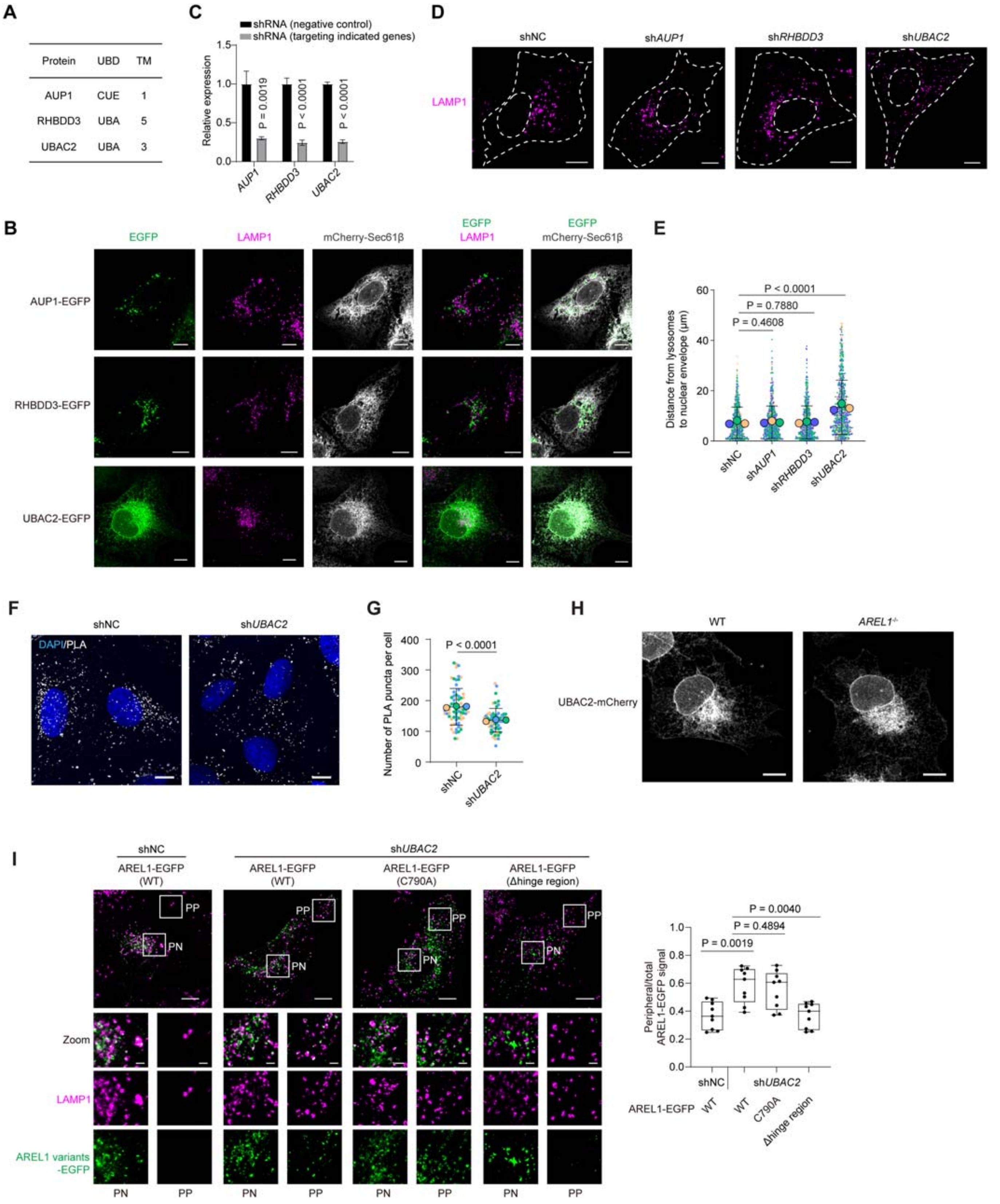
A
Protein UBD TM
AUP1 CUE 1
RHBDD3 UBA 5
UBAC2 UBA 3
C
shRNA (negative control)
shRNA (targeting indicated genes)
Relative expression
P = 0.0019
P < 0.0001
P < 0.0001
AUP1
RHBDD3
UBAC2
D
shNC
shAUP1
shRHBDD3
shUBAC2
LAMP1
B
EGFP
LAMP1
mCherry-Sec61β
AUP1-EGFP
RHBDD3-EGFP
UBAC2-EGFP
E
Distance from lysosomes to nuclear envelope (μm)
P < 0.0001
P = 0.7880
P = 0.4608
F
DAPI/PLA
G
Number of PLA puncta per cell
P < 0.0001
H
WT
AREL1-/-
UBAC2-mCherry
I
AREL1-EGFP (WT)
AREL1-EGFP (C790A)
AREL1-EGFP (Δhinge region)
PP
PN
Zoom
AREL1 variants -EGFP
Peripheral/total AREL1-EGFP signal
P = 0.0040
P = 0.4894
P = 0.0019
AREL1-EGFP
Δhinge region

Figure EV7. The effects of UBAC2 deficiency on lysosomal positioning, ER–lysosome MCSs, and AREL1 subcellular distribution, related to Fig. 5.

(**A**) List of ubiquitin-binding proteins with transmembrane domains. CUE coupling of ubiquitin conjugation to ER degradation, UBA Ubiquitin-associated, UBD ubiquitin-binding domain, TM transmembrane. (**B**) Representative confocal images showing the localization of AUP1, RHBDD3 and UBAC2. U2OS cells were transduced with lentiviruses expressing mCherry-Sec61β together with AUP1-EGFP, RHBDD3-EGFP, or UBAC2-EGFP and then immunostained with anti-LAMP1 antibody. Scale bars, 10 µm. (**C**) Verification of knockdown efficiency of *AUP1, RHBDD3 and UBAC2* in U2OS cells analyzed by quantitative real-time PCR. *P* values from left to right: 0.0019, <0.0001, <0.0001. (**D**) Representative confocal images showing the distribution of lysosomes in U2OS cells transduced with lentiviruses encoding negative control shRNA (shNC) and shRNA against indicated genes. Cell contour and nucleus are outlined using white dashed lines. Scale bars, 10 µm. (**E**) Superplots showing the distance from lysosomes to the nuclear envelope (small dots) and its mean per independent experiment (large dots). Means and error bars (SD) are shown as black bars. # of cells (# of lysosomes): shNC, 9 (678); sh*AUP1*, 9 (732); sh*RHBDD3*, 9 (677) and sh*UBAC2*, 9 (770); from 3 independent experiments. Mann–Whitney *U* test. shNC cells vs sh*AUP1* cells, $P = 0.4608$; shNC cells vs sh*RHBDD3* cells, $P = 0.7880$; shNC cells vs sh*UBAC2* cells, $P < 0.0001$. (**F**) Representative images showing PLA signals in shNC and sh*UBAC2* U2OS cells. Cells were fixed and immunostained with anti-calnexin and anti-LAMP1 antibodies followed by proximity ligation assay. Scale bars, 10 µm. (**G**) Superplots showing the number of PLA puncta per cell (small dots) and its mean per independent experiment (large dots). Means and error bars (SD) are shown as black bars. # of cells: shNC, 69 and sh*UBAC2*, 67; from 3 independent experiments. Unpaired two-tailed Student's *t* test. shNC cells vs sh*UBAC2* cells, $P < 0.0001$. (**H**) Representative confocal images showing the distribution of UBAC2-mCherry in WT and $AREL1^{-/-}$ U2OS cells. Scale bars, 10 µm. (**I**) Representative confocal images showing the localization of AREL1-EGFP variants and lysosomes in shNC and sh*UBAC2* U2OS cells. Boxed areas are enlarged on the bottom. Scale bars, 10 µm (main), 2 µm (inset). Right is box plots showing relative AREL1-EGFP signal in the peripheral region (3 outermost shells over 5 total shells per cell). Data are presented as median with interquartile range. Each box-and-whisker consists of the 25th quantile (the upper border of box), median (horizontal line inside the box), 75th quantile (the lower border of box), and vertical lines extending to the minimum and maximum values. # of cells: 9 for shNC cells expressing AREL1(WT)-EGFP, sh*UBAC2* cells expressing AREL1(WT)-EGFP, sh*UBAC2* cells expressing AREL1(C790A)-EGFP and sh*UBAC2* cells expressing AREL1(hinge region)-EGFP, respectively; from 3 independent experiments. Mann–Whitney *U* test. shNC cells expressing AREL1(WT)-EGFP vs sh*UBAC2* cells expressing AREL1(WT)-EGFP, $P = 0.0019$; sh*UBAC2* cells expressing AREL1(WT)-EGFP vs sh*UBAC2* cells expressing AREL1(C790A)-EGFP, $P = 0.4894$; sh*UBAC2* cells expressing AREL1(WT)-EGFP vs sh*UBAC2* cells expressing AREL1(hinge region)-EGFP, $P = 0.0040$ Source data are available online for this figure.

 © The Author(s)

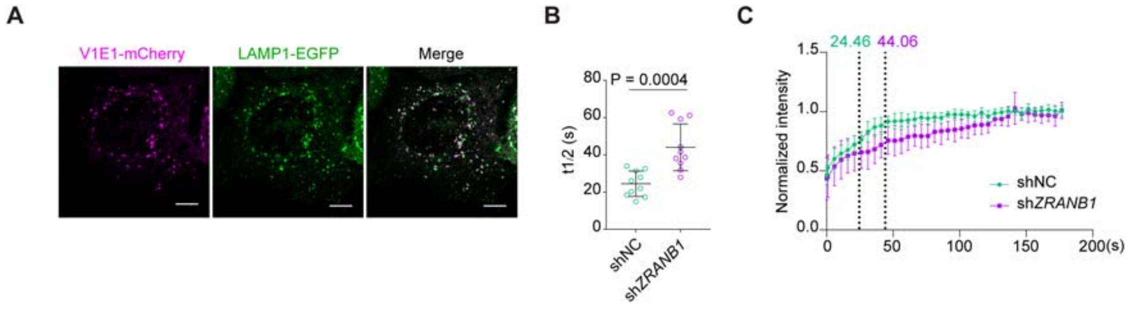

Figure EV8. The effects of ZRANB1 deficiency on V-ATPase assembly.

(**A**) Representative confocal images showing the localization of V_1E1-mCherry in U2OS cell. Scale bar, 10 µm. (**B**) Quantification of half times of fluorescence recovery ($\tau_{1/2}$) of V_1E1-mCherry. Data are presented as mean ±SD. # of cells: shNC, 9 and sh*ZRANB1*, 9; from 2 independent experiments. Unpaired two-tailed Student's *t* test. shNC cells vs sh*ZRANB1* cells, $P = 0.0004$. (**C**) Recovery curves of V_1E1-mCherry normalized to the fluorescence signal after 180 s. Data are presented as mean ± SD. # of cells: shNC, 10 and sh*ZRANB1*, 10; from 2 independent experiments Source data are available online for this figure.

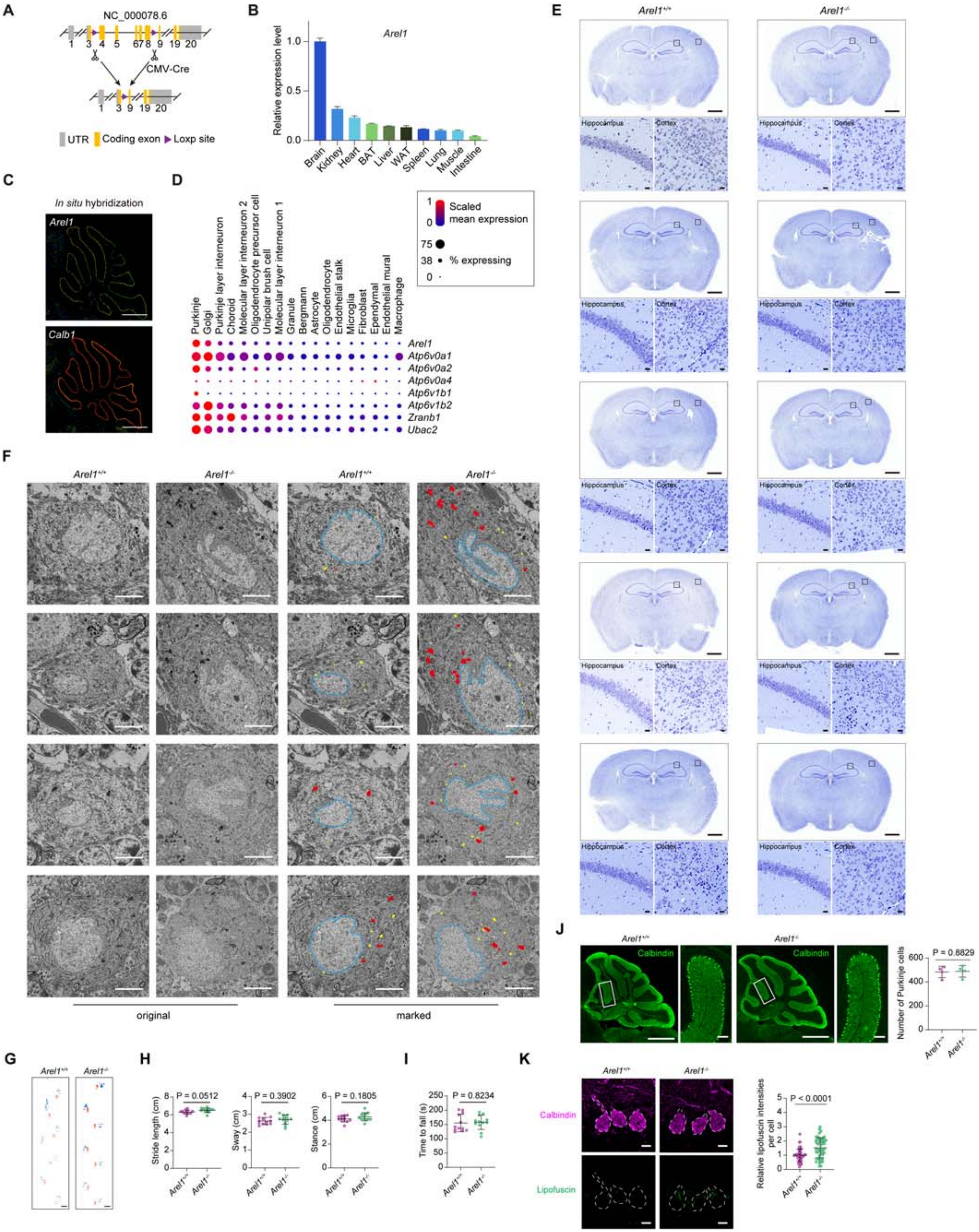
A
NC_000078.6
CMV-Cre
UTR Coding exon Loxp site
B
Arel1
Relative expression level
Brain Kidney Heart BAT Liver WAT Spleen Lung Muscle Intestine
C
In situ hybridization
Arel1
Calb1
D
Scaled mean expression
% expressing
Purkinje Golgi Purkinje layer interneuron Choroid Molecular layer interneuron 2 Oligodendrocyte precursor cell Unipolar brush cell Molecular layer interneuron 1 Granule Bergmann Astrocyte Oligodendrocyte Endothelial stalk Microglia Fibroblast Ependymal Endothelial mural Macrophage
Arel1 Atp6v0a1 Atp6v0a2 Atp6v0a4 Atp6v1b1 Atp6v1b2 Zranb1 Ubac2
E
Hippocampus
Cortex
F
original
marked
G
H
P = 0.0512
P = 0.3902
P = 0.1805
Stride length (cm)
Sway (cm)
Stance (cm)
I
P = 0.8234
Time to fall (s)
J
Calbindin
P = 0.8829
Number of Purkinje cells
K
Calbindin
Lipofuscin
P < 0.0001
Relative lipofuscin intensities per cell

 © The Author(s)

Figure EV9. Characterization of AREL1 expression in mouse tissues and *Arel1*$^{-/-}$ mouse phenotypes, related to Fig. 6.

(**A**) *Arel1* knockout strategy. *Arel1*$^{floxed/floxed}$ mice were crossed with CMV-Cre transgenic mice to generate *Arel1*$^{-/-}$ mice. (**B**) Quantitative real-time PCR analysis showing *Arel1* mRNA levels in different mouse tissues. Data are presented as means ± SD (n = 3 biological replicates of the pooled samples from three 2-month-old C57/BL6J mice). (**C**) In situ hybridization image data retrieved from the Allen Mouse Brain Atlas (https://mouse.brain-map.org/) showing *Arel1* and *Calb1* expression in mouse cerebellum. Scale bars, 1 mm. (**D**) Dot plot showing expression of indicated genes in 18 cell types of adult mouse cerebellum. Data were retrieved from the work by Kozareva et al, 2021 and analyzed in Single Cell Portal. Dot size encodes percentage of cells expressing the gene, and color encodes the average per cell gene expression level. (**E**) Representative Nissl staining of coronal brain sections from five 12-month-old *Arel1*$^{+/+}$ and *Arel1*$^{-/-}$ male mice per genotype. The hippocampal CA1 region and cerebral cortex are enlarged and shown on the bottom. Scale bars, 1 mm (main), 100 µm (inset). (**F**) Transmission electron micrographs (original and color-labeled) showing Purkinje cells in 12-month-old *Arel1*$^{+/+}$ and *Arel1*$^{-/-}$ male mice. Nuclear contours are outlined by blue lines and lysosomes and lipofuscin granules marked in yellow and red, respectively. Scale bars, 5 µm. (**G**) Representative footprints of 6-month-old *Arel1*$^{+/+}$ and *Arel1*$^{-/-}$ male mice. Scale bars, 2 cm. (**H**) Stride length, sway and stance of 6-month-old *Arel1*$^{+/+}$ and *Arel1*$^{-/-}$ male mice. Data are presented as mean ± SD ($n = 12$ for both *Arel1*$^{+/+}$ and *Arel1*$^{-/-}$ mice). Unpaired two-tailed Student's t test. Stride length in *Arel1*$^{+/+}$ mice vs stride length in *Arel1*$^{-/-}$ mice, $P = 0.0512$; sway in *Arel1*$^{+/+}$ mice vs sway in *Arel1*$^{-/-}$ mice, $P = 0.3902$; stance in *Arel1*$^{+/+}$ mice vs stance in *Arel1*$^{-/-}$ mice, $P = 0.1805$. (**I**) Time to fall off the rods of 6-month-old *Arel1*$^{+/+}$ and *Arel1*$^{-/-}$ male mice. Data are presented as mean± SD ($n = 12$ for both *Arel1*$^{+/+}$ and *Arel1*$^{-/-}$ mice). Unpaired two-tailed Student's t test. *Arel1*$^{+/+}$ mice vs *Arel1*$^{-/-}$ mice, $P = 0.8234$. (**J**) Representative confocal images showing calbindin expression in the cerebellum of 6-month-old *Arel1*$^{+/+}$ and *Arel1*$^{-/-}$ male mice. Boxed areas are enlarged and shown on the right. Scale bars, 1 mm (main), and 50 µm (inset). Quantification of calbindin-positive Purkinje cells is presented as mean ±SD ($n = 4$ mice per genotype). Unpaired two-tailed Student's t test. *Arel1*$^{+/+}$ mice vs *Arel1*$^{-/-}$ mice, $P = 0.8829$. (**K**) Representative confocal images showing calbindin staining (magenta) and lipofuscin autofluorescence (green) in Purkinje cells of 6-month-old *Arel1*$^{+/+}$ and *Arel1*$^{-/-}$ male mice. Scale bars, 10 µm (main). Quantification of lipofuscin autofluorescence in calbindin-positive Purkinje cells is presented as mean ± SD ($n = 53$ and 50 cells for *Arel1*$^{+/+}$ and *Arel1*$^{-/-}$ male mice, respectively). Unpaired two-tailed Student's t test. *Arel1*$^{+/+}$ mice vs *Arel1*$^{-/-}$ mice, $P < 0.0001$ Source data are available online for this figure.

© The Author(s)
